# The burden of primary liver cancer caused by specific etiologies from 1990 to 2019 at the global, regional, and national levels

**DOI:** 10.1002/cam4.4530

**Published:** 2022-01-06

**Authors:** Jianqing Yang, Guangdong Pan, Linjing Guan, Zhipeng Liu, Yongrong Wu, Zhen Liu, Wuchang Lu, Shuai Li, Honglai Xu, Guoqing Ouyang

**Affiliations:** ^1^ Department of Hepatobiliary Surgery Liuzhou People’s Hospital affiliated to Guangxi Medical University Liuzhou China; ^2^ Department of Hepatobiliary Ultrasound Liuzhou People’s Hospital affiliated to Guangxi Medical University Liuzhou China; ^3^ Department of Hepatobiliary Surgery Changsha Hospital of Traditional Chinese Medicine Changsha China

**Keywords:** disability‐adjusted life years, global burden of disease, incidence, liver cancer, mortality

## Abstract

**Background:**

Liver cancer is one of the most common cancers worldwide. We aimed to report the burden of liver cancer at the global, regional, and national levels in 204 countries from 1990 to 2019, stratified by etiology, sex, age, and sociodemographic index (SDI).

**Methods:**

Data of mortality, incidence, and disability‐adjusted life years (DALYs) of liver cancer and its etiology were available from the Global Burden of Diseases, Injuries, and Risk Factors (GBD) Study 2019. The trends in the liver cancer burden were assessed by the annual percentage change. All estimates are presented as numbers and age‐standardized rates (ASRs) per 100,000 population, with uncertainty intervals (UIs).

**Results:**

Globally, 484,577 (95% UI 444,091–525,798) mortalities, 534,364 (486,550–588,639) incident cases, and 12,528,422 (11,400,671–13,687,675) disability‐adjusted life years (DALYs) due to liver cancer occurred in 2019. The ASRs were 5.95 (5.44–6.44), 6.51 (5.95–7.16), and 151.08 (137.53–164.8) per 100,000 population for the mortalities, incidences, and DALYs, respectively. From 1990 to 2019, the numbers increased, whereas the ASRs decreased. Hepatitis B and Hepatitis C are the major causes of liver cancer mortality. The liver cancer mortality in 2019 increased with age, peaking at 65–69 and 70–74 age group in males and females, respectively, and the number was higher in males than in females. Generally, there were nonlinear associations between the ASR and SDIs values at the regional and national levels. China had the highest numbers of mortalities, incident cases, and DALYs, whereas Mongolia has the highest ASR in 2019.

**Conclusion:**

Liver cancer remains a major public health issue worldwide, but etiological and geographical variations exist. It is necessary to increase awareness of the population regarding liver cancer, its etiologies and the importance of early detection, and diagnosis and treatment.

## Graphical abstract

Liver cancer is one of the most common cancers worldwide. We aim to report the burden of liver cancer at the global, regional, and national levels in 204 countries from 1990 to 2019, stratified by etiology, sex, age, and sociodemographic index (SDI). Liver cancer remains a major public health issue worldwide, but there are etiological and geographical variations. Increasing the awareness of the population regarding liver cancer, its etiologies and the importance of early detection, and diagnosis and treatment are needed.

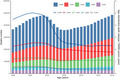

## 1 INTRODUCTION

Liver cancer was the sixth most commonly diagnosed cancer and fourth leading cause of cancer‐related mortality globally in 2018, with 841,000 incident cases and 782,000 mortalities.^1^ The most common type of primary liver cancer is hepatocellular carcinoma, followed by intrahepatic cholangiocarcinoma and other rare types (sarcoma, hemangioendothelioma, etc.). The prognosis of liver cancer is poor, with an overall 5‐year survival rate of only 19.6%.^2^ In addition, the burden of liver cancer continues to increase despite substantial efforts to prevent it.^3^, ^4^, ^5^, ^6^

The etiologies of liver cancer include hepatitis B virus (HBV), hepatitis C virus (HCV), alcohol consumption, nonalcoholic fatty liver disease (NAFLD), and other causes (aflatoxins and microcystins). Among them, HBV and HCV were the primary risk factors for liver cancer.^5^ Previous study demonstrated that the burden of liver cancer varied considerably across geography, sex, age, and etiology.^5^, ^6^, ^7^, ^8^ For example, HBV is the main cause of liver cancer in developing countries (such as China and India), whereas HCV and alcohol consumption are the main risk factors for liver cancer in developed countries (such as the United Kingdom and United States).^5^, ^7^, ^9^ The disease burden of liver cancer in males was believed to be twofold to threefold higher than that in females,^5^, ^9^ however in some regions, the burden of liver cancer caused by HCV and nonalcoholic steatohepatitis (NASH) was higher in females.^5^

In recent years, the trends in the incidence and mortality due to liver cancer have been assessed by several studies, and the results suggested that liver cancer is still a major public concern.^5^, ^6^, ^7^, ^8^, ^9^ However, previous studies only included 195 countries, and no updated global studies on liver cancer have been published since the 2017 estimates. To provide comparable, comprehensive, and up‐to‐date details, this study presents estimates of numbers and age‐standardized rates (ASRs) of incidence, mortality, and disability‐adjusted life years (DALYs) for liver cancer in 204 countries and territories from 1990 to 2019; stratified by etiology, age, sex, and sociodemographic index (SDI). To our knowledge, this study is the first to investigate the association between the trend of liver cancer burden and SDI at the regional and national levels from 1990 to 2019.

## 2 METHODS

### 2.1 Data sources

Data on liver cancer mortality, incidence, and DALYs stratified by region, country, sex, age, and etiology were collected from the Global Burden of Diseases, Injuries, and Risk Factors (GBD) study 2019.^10^ Data were available from 204 countries and territories, which were divided into 21 regions based on the GBD study. The detailed methodology of the estimation of the burden of liver cancer and the latest updates have been described extensively in GBD 2019 papers.^10^, ^11^ Briefly, all available sources of information, including published researches, survey data, census data, surveillance system data, vital statistics, and other health‐related data sources, were gathered to estimate the liver cancer burden. The codes for liver cancer from the International Classification of Diseases (ICD) version 10 (C22–C22.8, D13.4) and version 9 (155–155.1, 155.3–155.9, and 211.5) were used. The etiology of liver cancer was divided into HBV, HCV, alcohol use, NASH, and other causes.^10^

### 2.2 Statistical analysis

The age‐standardized incidence rate (ASIR), age‐standardized mortality rate (ASMR), and age‐standardized DALYs rate (ASDR) across five different etiologies were used to quantify the trends in the global liver cancer. The annual percentage change in each trend was also evaluated in this study, and liver cancer burden trends were considered to be increasing or decreasing based on a positive or negative percentage change value, respectively. The 2.5^th^ and 97.5^th^ centiles of the ordered draws were determined as the 95% uncertainty intervals (UIs).

To determine the shape of the curve of the association between the liver cancer burden in terms of mortality, incidence, DALYs, and SDIs for 21 regions and 195 countries and territories, smoothing spline models were employed.^12^ Although the corresponding linear models were lower than *R* ^2^ of smoothing splines, more focus was paid to the shape of dose–response relationships rather than the fit of models. The SDI is a value ranging from 0 (worst) to 1.0 (best), which is a composite indicator of lag‐distributed income per capita (LDI) and gross domestic product per capita that has been smoothed over the preceding.

10 years, average years of schooling for the population older than 15 years, and total fertility rate under the age of 25.^10^, ^11^ All statistical analyses were performed with R software version 3.6.3 and visualized using the ggplot2 3.3.0 package.^13^ The differences between sexes were compared with an unpaired *t*‐test. A *p* value <0.05 was considered statistically significant.

## 3 RESULTS

### 3.1 Global burden of liver cancer

**FIGURE 1:**
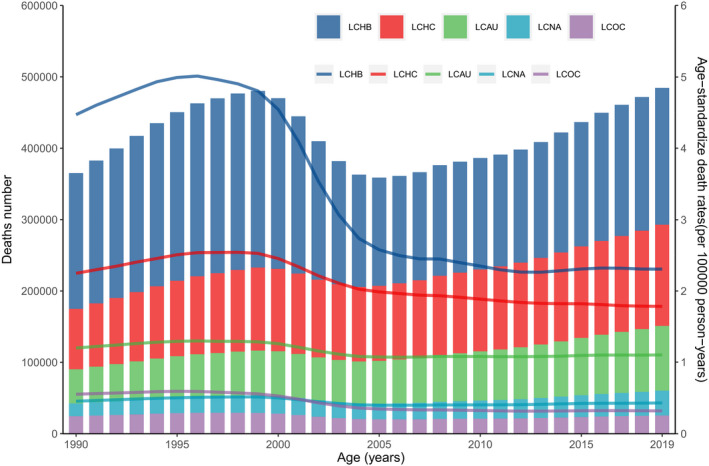
Number of mortality and ASMR at the global level by etiology of primary liver cancer, 1990–2019. LCHB, liver cancer due to hepatitis B. LCHC, liver cancer due to hepatitis C. LCAU, liver cancer due to alcohol use. LCNA, liver cancer due to NASH. LCOC, liver cancer due to other cause. ASMR, age‐standardized mortality rate

**FIGURE 2:**
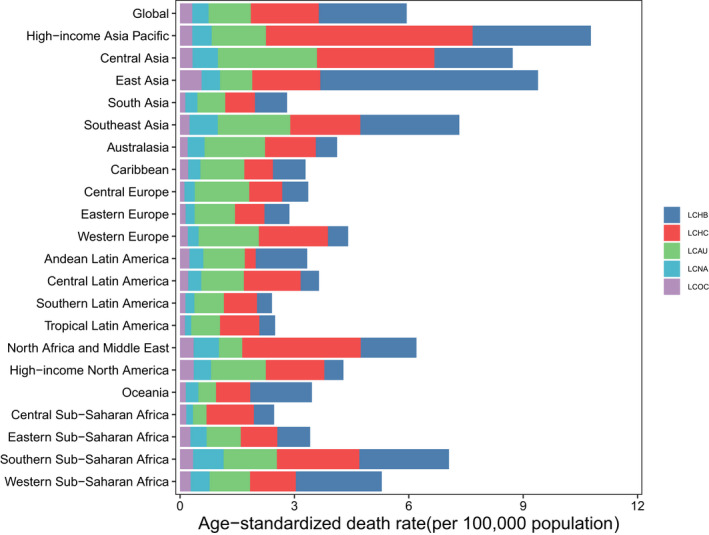
Age‐standardized mortality rate for liver cancer, by region and etiology, 2019. LCHB, liver cancer due to hepatitis B. LCHC, liver cancer due to hepatitis C. LCAU, liver cancer due to alcohol use. LCNA, liver cancer due to NASH. LCOC, liver cancer due to other cause

Globally, there were 484,577 (95% UI 444,091–525,798) mortality caused by liver cancer in 2019; the number increased by 32.68% from 365,215 (329,967–405,773) in 1990. The ASMR at the global level decreased by 33.40% from 8.93 (8.09 to 9.90) in 1990 to 5.95 (5.44–6.44) in 2019 per 100,000 population (Table 1, Figures 1 and 2). The incidence of liver cancer in both sexes increased by 43.11% from 373,390 (335,890–415,748) in 1990 to 534,364 (486,550–588,639) in 2019. The global ASIR of liver cancer was 8.98 (8.10–9.97) per 100,000 population in 1990, which decreased to 6.51 (5.95–7.16) per 100,000 population in 2019 (Table 1; Figures S1 and S2). In 2019, liver cancer caused 12,528,422 (11,400,671–13,687,675) DALYs, which was an 11.08% increase from the 11,278,630 (10,062,526–12,677, 403) DALYs in 1990. Similarly, the ASDR showed a decreasing trend from 258.37 (230.90–290.13) in 1990 to 151.08 (137.53–164.8) in 2019 per 100,000 population (Table 1; Figures S3 and S4).

**TABLE 1:** Death, incident cases, and disability‐adjusted life years (DALYs) for liver cancer in 2019 and percentage change in age‐standardized rates (ASRs) per 100,000 population from 1990 to 2019 by Global Burden of Disease regions

Characteristics	Death (95% uncertainty interval)			Incidence (95% uncertainty interval)			DALYs (95% uncertainty interval)		
	Counts	ASR per 100,000 population (95% UI)	Percentage change in ASRs per 100,000 population (95% UI)	Counts	ASR per 100,000 population (95% UI)	Percentage change in ASRs per 100,000 population (95% UI)	Counts	ASR per 100,000 population (95% UI)	Percentage change in ASRs per 100,000 population (95% UI)
Global	484,577 (444,091 to 525,798)	5.9 (5.4 to 6.4)	−33.4 (−41.9 to −23.2)	534,364 (486,550 to 588,639)	6.5 (5.9 to 7.2)	−27.5 (−37.3 to −15.7)	12,528,422 (11,400,671 to 13,687,675)	151.1 (137.5 to 164.8)	−41.5 (−49.8 to −31.5)
Sex									
Male	333,673 (299,581 to 368,334)	8.7 (7.9 to 9.6)	−32.3 (−42.7 to −19.3)	376,483 (335,003 to 421,982)	9.7 (8.7 to 10.8)	−25.7 (−37 to −10.1)	9,048,723 (8,022,502 to 10,072,046)	225.3 (200.4 to 250.2)	−40.4 (−50.4 to −27.8)
Female	150,904 (134,123 to 167,013)	3.5 (3.1 to 3.8)	−35 (−46.3 to −22.4)	157,881 (140,436 to 176,052)	3.6 (3.2 to 4)	−30.5 (−42.7 to −17.2)	3,479,699 (3,108,771 to 3,866,969)	81.3 (72.7 to 90.3)	−43.3 (−54.2 to −31.2)
Regions									
Andean Latin America	1840 (1510 to 2232)	3.3 (2.7 to 4)	−36.2 (−49.1 to −20.6)	1735 (1419 to 2114)	3.1 (2.5 to 3.8)	−36.4 (−49.6 to −20.1)	44,340 (35,812 to 54,428)	77.3 (62.6 to 94.7)	−40.5 (−53.3 to −24.9)
Australasia	2006 (1832 to 2174)	4.1 (3.8 to 4.5)	107.8 (91.4 to 124.8)	2160 (1752 to 2667)	4.6 (3.7 to 5.7)	124.1 (82.3 to 176.8)	43,655 (40,249 to 47,404)	98.1 (90.3 to 106.4)	94.8 (77.6 to 112)
Caribbean	1695 (1418 to 2005)	3.3 (2.8 to 3.9)	−47.7 (−55.7 to −38)	1628 (1353 to 1938)	3.2 (2.6 to 3.8)	−46.7 (−55.1 to −36.4)	41,276 (33,562 to 50,616)	80.7 (65.6 to 99.2)	−46.8 (−55.7 to −35.5)
Central Asia	6191 (5387 to 7076)	8.7 (7.6 to 9.9)	169.6 (129.8 to 214.6)	6109 (5296 to 7001)	8.3 (7.2 to 9.4)	164.3 (124.9 to 209.7)	172,830 (148,859 to 200,042)	213.5 (184.9 to 244.5)	150.2 (111.1 to 196.2)
Central Europe	7202 (6218 to 8327)	3.4 (2.9 to 3.9)	−39.9 (−48.2 to −30.6)	6906 (5994 to 7986)	3.3 (2.9 to 3.8)	−37.4 (−45.7 to −27.8)	156,614 (133,681 to 182,107)	79.1 (67.7 to 92.3)	−39.9 (−48.7 to −30.1)
Central Latin America	8416 (7357 to 9750)	3.6 (3.2 to 4.2)	−2.4 (−14.5 to 12.1)	7987 (6880 to 9272)	3.4 (3 to 4)	−1.9 (−14 to 13.4)	197,475 (171,637 to 231,238)	82.8 (72.1 to 97)	−7.2 (−19.2 to 7.7)
Central Sub‐Saharan Africa	1394 (1108 to 1753)	2.5 (2 to 3.1)	−13.3 (−31.8 to 11.5)	1364 (1080 to 1715)	2.3 (1.8 to 2.9)	−12.6 (−32.3 to 15.1)	51,448 (38,555 to 67,260)	65.3 (51.7 to 82.1)	−15.5 (−36 to 12.1)
East Asia	193,864 (163,848 to 228,758)	9.4 (8 to 11)	−63.2 (−70.9 to −53)	217,171 (181,403 to 257,464)	10.4 (8.8 to 12.3)	−58.7 (−67.7 to −47.3)	5,491,479 (4,590,535 to 6,534,290)	263.4 (221.3 to 312.2)	−65.1 (−72.8 to −55)
Eastern Europe	9676 (8506 to 11,122)	2.9 (2.5 to 3.3)	85 (64.9 to 108.3)	9407 (8199 to 10,735)	2.8 (2.5 to 3.2)	87.1 (66 to 110)	234,701 (205,032 to 273,291)	74.9 (65.3 to 86.6)	72 (51.5 to 95.4)
Eastern Sub‐Saharan Africa	5677 (4683 to 6919)	3.4 (2.9 to 4.2)	8.4 (−10.7 to 32.4)	5439 (4462 to 6714)	3.1 (2.6 to 3.8)	6.6 (−13.2 to 30.7)	187,944 (149,325 to 232,670)	85.5 (70.2 to 105.1)	3 (−20.1 to 30.4)
High‐income Asia Pacific	49,685 (43,778 to 53,504)	10.8 (9.8 to 11.5)	−7.2 (−14.4 to −0.9)	67,946 (58,134 to 77,642)	15.6 (13.5 to 17.7)	13 (−1.7 to 28.2)	92,0379 (842,591 to 983,716)	238.6 (220.6 to 255.5)	−19.3 (−25.4 to −13.2)
High‐income North America	26,479 (23,637 to 28,913)	4.3 (3.8 to 4.7)	111.4 (89.3 to 130.2)	31,008 (25,713 to 36,961)	5.2 (4.3 to 6.2)	134.8 (94.1 to 179.3)	608,194 (543,851 to 664,431)	105.5 (94.5 to 115.2)	107 (84.9 to 126.2)
North Africa and Middle East	26,432 (21,211 to 32,611)	6.2 (5.1 to 7.6)	−3 (−24.7 to 29.5)	27,546 (22,113 to 33,841)	6.3 (5.1 to 7.7)	3.5 (−19.3 to 37.7)	731,622 (578,678 to 923,575)	153.3 (121.9 to 189.8)	−4.8 (−27.1 to 27.8)
Oceania	233 (195 to 277)	3.5 (2.9 to 4.1)	−10.1 (−27.2 to 10.6)	234 (195 to 278)	3.3 (2.8 to 3.9)	−10.3 (−27.3 to 10.3)	7093 (5872 to 8495)	85.4 (71.3 to 101.6)	−13.1 (−29.3 to 7)
South Asia	38,650 (33,517 to 44,561)	2.8 (2.4 to 3.2)	−0.5 (−17.2 to 19.7)	37,733 (32,783 to 43,281)	2.7 (2.3 to 3.1)	0.1 (−17.5 to 20.1)	1,085,515 (943,943 to 1,244,802)	71.3 (62 to 81.8)	−2.3 (−17.8 to 15.7)
Southeast Asia	42,862 (35,326 to 51,520)	7.3 (6.1 to 8.8)	8.3 (−13 to 33.8)	42,800 (35,218 to 52,129)	7.1 (5.9 to 8.6)	10 (−11.2 to 37.7)	1,149,098 (943,489 to 1,384,243)	177.5 (146.6 to 213.5)	−1.5 (−20.9 to 21.4)
Southern Latin America	2027 (1897 to 2152)	2.4 (2.3 to 2.6)	46.1 (32 to 64.5)	1939 (1524 to 2424)	2.3 (1.8 to 2.9)	49.4 (16.5 to 90.4)	43,534 (40,967 to 46,273)	53.6 (50.4 to 57)	37 (23.3 to 54.3)
Southern Sub‐Saharan Africa	4040 (3618 to 4540)	7.1 (6.3 to 7.9)	4.7 (−39.9 to 58.4)	4016 (3581 to 4521)	6.8 (6.1 to 7.6)	4.7 (−40 to 58.8)	122,195 (108,238 to 137,902)	188.8 (168.3 to 213)	3.9 (−40.7 to 57)
Tropical Latin America	5939 (5543 to 6239)	2.5 (2.3 to 2.6)	19.1 (12.3 to 26.4)	5667 (5335 to 5956)	2.4 (2.2 to 2.5)	19.7 (13.3 to 27.3)	142,719 (135,353 to 150,317)	58.6 (55.4 to 61.7)	12.4 (6.1 to 19.3)
Western Europe	40,296 (37,224 to 42,876)	4.4 (4.1 to 4.7)	28.5 (21.4 to 36.3)	45,859 (39,837 to 52,739)	5.3 (4.6 to 6.1)	49.5 (29.8 to 72.9)	787,717 (738,440 to 836,208)	98.5 (93 to 104.5)	22 (15.2 to 29.9)
Western Sub‐Saharan Africa	9972 (8360 to 11,564)	5.3 (4.5 to 6)	−8.9 (−26.3 to 11.3)	9709 (8164 to 11,417)	4.9 (4.2 to 5.7)	−9.5 (−27.6 to 10.5)	308,593 (252,949 to 365,495)	130.8 (109.4 to 152.3)	−13.6 (−31 to 5.3)

**FIGURE 3:**
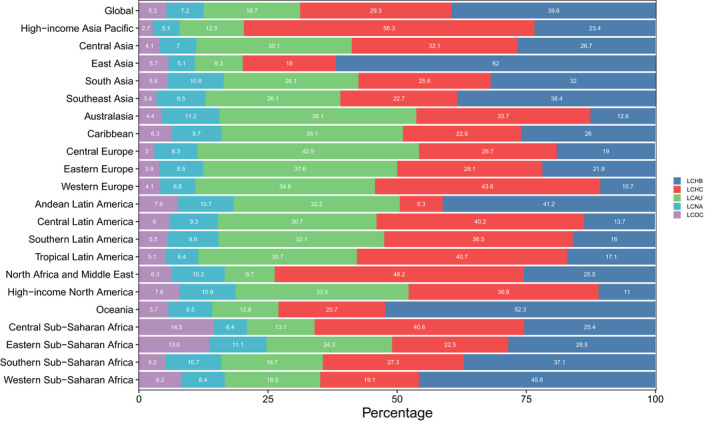
Contribution of LCHB, LCHC, LCAU, LCNA, and LCOC to primary liver cancer mortality, both sexes, globally and by region, 2019. LCHB, liver cancer due to hepatitis B. LCHC, liver cancer due to hepatitis C. LCAU, liver cancer due to alcohol use. LCNA, liver cancer due to NASH. LCOC, liver cancer due to other cause

In 2019, approximately 39.57% of liver cancer‐related mortalities in both sexes were attributed to hepatitis B, 29.26% were attributed to hepatitis C, 18.73% were attributed to alcohol use, 7.17% were attributed to NASH, and 5.27% were attributed to other causes (Figure 3). Liver cancer due to hepatitis B (LCHB) caused 191,737 (161,861–223,727) mortalities in 2019, which was only a 0.76% increase from 190,291 (162,332–222,448) (Table S1). In contrast, the ASMR of LCHB decreased by 48.4% from 4.47 (3.82–5.22) per 100,000 population in 1990 to 2.31 (1.95–2.69) per 100,000 population in 2019 (Table S1). There were 84,665 (73,797–96,590) liver cancer due to hepatitis C (LCHC)‐related mortalities in 1990 and 141,811 (121,787–161,828) in 2019, with an ASMR of 2.25 (1.97–2.54) per 100,000 population in 1990 and 1.78 (1.53–2.04) per 100,000 population in 2019, this rate decreased by 20.63% from 1990 to 2019 (Table S1). Liver cancer due to alcohol use (LCAU) caused 47,858 (38,590–58,606) mortalities in 1990 and 90,741 (73,349–109,402) mortalities in 2019. The ASMR of LCAU remained stable, changing from 1.20 (0.97–1.46) per 100,000 population in 1990 to 1.10 (0.89–1.33) per 100,000 population in 2019 (Table S1). There were 17,800 (14,647–21,515) liver cancer due to NASH (LCNA)‐related mortalities in 1990 and 34,729 (28,395–43,182) in 2019, with an ASMR of 0.46 (0.36–0.55) per 100,000 population in 1990 and 0.43 (0.35–0.53) per 100,000 population in 2019 (Table S1). This rate also remained stable from 1990 to 2019. Liver cancer due to other causes (LCOC) caused almost 24,599 (20,584–29,473) mortalities in 1990 and 25,560 (21,229–30,491) mortalities in 2019. The ASMR of LCOC decreased by 42.17% from 0.55 (0.46–0.66) per 100,000 population in 1990 to 0.32 (0.27–0.38) per 100,000 population in 2019 (Table S1, Figures 1 and 2).

### 3.2 Regional burden of liver cancer

At the regional level, the 2019 ASMR and ASIR of liver cancer in high‐income Asia Pacific were 10.78 (9.77–11.53) and 15.56 (13.46–17.74) per 100,000 population, respectively, which ranked the first among the 21 GBD regions in 2019 (Figure 2; Figure S2). In addition, the highest ASDR of liver cancer per 100,000 population was found in East Asia (263.40 [221.29–312.17]) in 2019 (Figure S4). The most pronounced increases in the ASMR, ASIR, and ASDR were observed in Central Asia, followed by the high‐income North America and Australasia from 1990 to 2019. East Asia had the largest decreases in the ASMR, ASIR, and ASDR during the past 30 years (Tables S1–S3).

In 2019, the highest ASMR of LCHB for both sexes was found in East Asia (5.71 [4.68–6.92]). In contrast, the lowest ASMR of LCHB was observed in Southern Latin America (0.39 [0.27–0.56]) (Table S1). In addition, the hepatitis B was the leading cause of liver cancer in East Asia, Oceania, and Western Sub‐Saharan Africa, accounting for 62%, 52.3%, and 46.8% of total liver cancer deaths in 2019, respectively (Figures 2 and 3).

The highest ASMR of LCHC in 2019 was detected in the high‐income Asia Pacific (5.42 [4.70–5.99]). In contrast, Andean Latin America (0.28 [0.18–0.43]) had the lowest ASMR of LCHC. In addition, >40% of cancer‐related mortalities due to hepatitis C were found in 6 of the 21 regions, namely high‐income Asia Pacific, North Africa and Middle East, Western Europe, Tropical Latin America, Central Sub‐Saharan Africa, and Central Latin America (Table S1; Figures 2 and 3).

Central Asia (2.60 [1.86–3.39]) had the highest ASMR of LCAU in both sexes in 2019. However, Central Sub‐Saharan Africa (0.35 [0.23–0.51]) showed the lowest ASMR. Moreover, the proportion of mortalities attributed to LCAU in Central Europe, Australasia, Eastern Europe, and Caribbean all exceeded 35% (Table S1; Figures 2 and 3).

The sum of the proportion of mortalities caused by liver cancer in 2019 that were attributable to NASH and other causes was no greater than 15%. Among all 21 GBD regions, the highest proportion attributable to NASH was in Central Sub‐Saharan Africa (14.5%), and the highest proportion attributable to other cause was in Australasia (11.2%). The highest ASMR of LCNA was in Southern Sub‐Saharan Africa (0.80 [0.65–0.97]). Meanwhile, East Asia (0.56 [0.46–0.68]) had the highest ASMR of LCOC in 2019 (Table S1; Figures 2 and 3).

### 3.3 National burden of liver cancer

**FIGURE 4:**
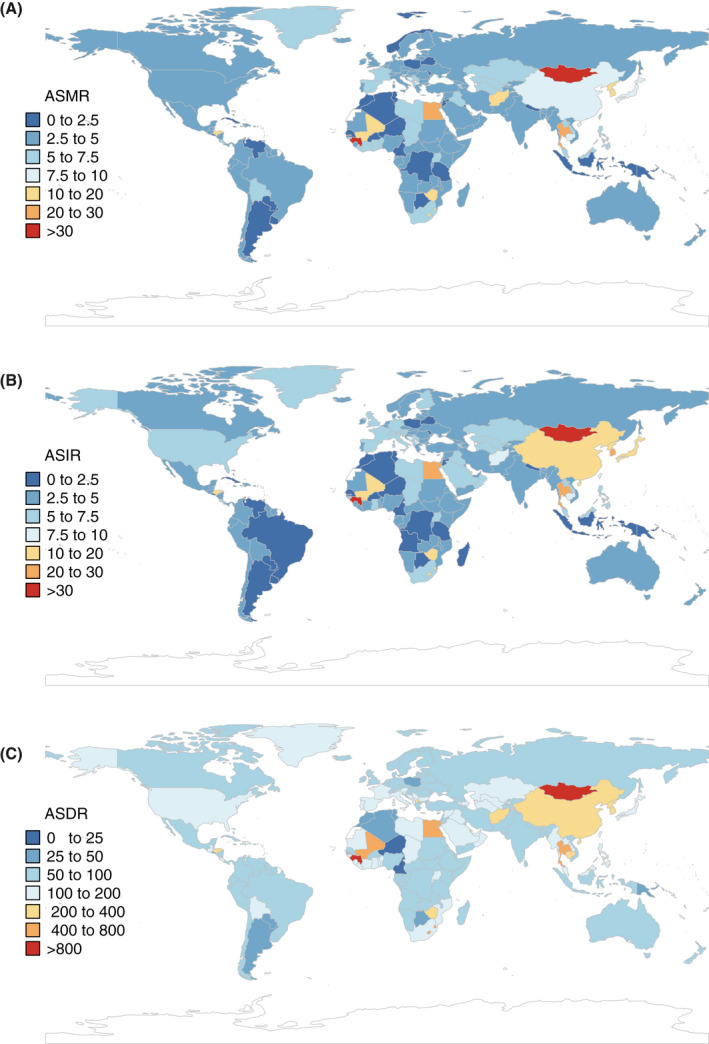
The global age‐standardized rate of primary liver cancer per 100,000 populations in 2019, by country and territory. (A) ASMR in 2019; (B) ASIR in 2019; and (C) ASDR in 2019. ASMR, age‐standardized mortality rate. ASIR, age‐standardized incidence rate. ASDR, age‐standardized DALYs rate

At the national level, China had the highest numbers of mortalities (187,700 [158,262–222,767]), incident cases (210,462 [174,832–251,195]), and DALYs (5,325,461 [4,425,687–6,374,588]), accounting for nearly half of the global totals in 2019 (Table S4–S6). The estimated ASMR of liver cancer ranged from 115.22 to 0.64 per 100,000 population in 2019. Mongolia had the highest ASMR, ASIR, and ASDR of liver cancer in 2019. In contrast, Niger showed the lowest ASMR, ASIR, and ASDR due to live cancer (Figure 4). In addition, the largest increases in the ASMR, ASIR, and ASDR over the past 30 years were in Cabo Verde. Poland showed the most pronounced decreases from 1990 to 2019 (Figure S7). For India, Indonesia, and Pakistan, three heavily populated countries, the ASMR was (2% [−17% to 23%]), (−7% [−25% to 16%]), and (0 [−27% to 48%]) from 1990 to 2019, respectively (Table S4–S6).

The country with the highest ASMR of LCHB in 2019 was in Mongolia (28.23 [18.92–40.83]), whereas the Sweden (0.18 [0.14–0.24]) had the lowest ASMR for LCHB. From 1990 to 2019, the countries with the largest increases and decreases in the ASMR of LCHB were the same as those for all liver cancer, regardless of etiology, which ranged from −76.65% to 897.72% (Table S4; Figures S8A and S9A).

The highest ASMR of LCHC in 2019 was found in Mongolia (40.31 [28.58–53.27]). In contrast, Cameroon (0.12 [0.08–0.18]) showed the lowest ASMR for LCHC. The most pronounced increases in the ASMR of LCHC were found in Cabo Verde, whereas the most pronounce decreases were also detected in Poland between 1990 and 2019. (Table S4; Figures S10A and S11A).

In 2019, the country with the highest ASMR of LCAU was in Mongolia (34.20 [23.11–47.83]). In contrast, the lowest ASMR of LCAU was found in three African countries: Niger (0.11 [0.06–0.16]), Cameroon (0.15 [0.09–0.23]), and Tunisia (0.21 [0.11–0.38]). The countries with the greatest increases and decreases in the ASDR of LCAU were the same as those for LCHB and LCHC, and the percent changes in the ASMR ranged from −72.61% to 1132.01% (Table S4; Figures S12A and S13A).

With regard to LCNA and LCOC, the highest ASMR was also in Mongolia; whereas the lowest ASMR was found in Niger. The largest increases in ASMR for LCNA and LCOC were detected in Cabo Verde, whereas the largest decreases in ASMR were found in Poland (Table S4; Figures S14A, S15A, S16A, and S17A).

Detailed information on incident cases, mortality, DALYs, ASIR, ASDR, and percent change for each etiology by global, region, and country are described in online supplementary 1 (Table S1–S6; Figures S7–S17).

### 3.4 Age and sex patterns

**FIGURE 5:**
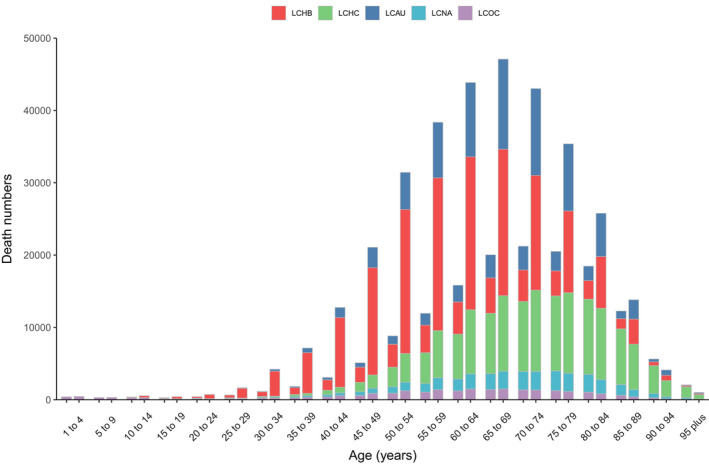
Global primary liver cancer mortality by etiology and age for females and males, 2019. For each group, the left column showed case data in female and the right column shows data in male. LCHB, liver cancer due to hepatitis B. LCHC, liver cancer due to hepatitis C. LCAU, liver cancer due to alcohol use. LCNA, liver cancer due to NASH. LCOC, liver cancer due to other cause

In 2019, the global number of mortalities from liver cancer was higher in males than in females across all age groups except the group older than 90 years (Figure 5). Similar patterns were detected for incidence and DALYs (Figures S18 and S19). The number of deaths from liver cancer in 2019 increased with increasing age, peaking at the group aged 65–69 years and 70–74 years in males and females, respectively, and then decreased with older age. The lowest number of mortalities was found in patients younger than 30 years. The five etiologies of liver cancer exhibited age‐related patterns, although the number of mortalities was higher in males than in females regardless of etiology. The number of mortalities from LCHB peaked in the group aged 60–64 years in males and in the group aged 65–69 years in females, which was the youngest peak among the five etiologies. LCHC and LCNA shared the same peak in the group aged 70–74 years in males, and LCAU and LCOC also shared the same peak in the group aged 65–69 years. For females, the number of deaths from LCHC, LCAU, LCNA, and LCOC peaked in the groups aged 80–84, 70–74, 75–79, and 65–69 years, respectively. (Figure 5).

### 3.5 Burden of liver cancer by sociodemographic Index

**FIGURE 6:**
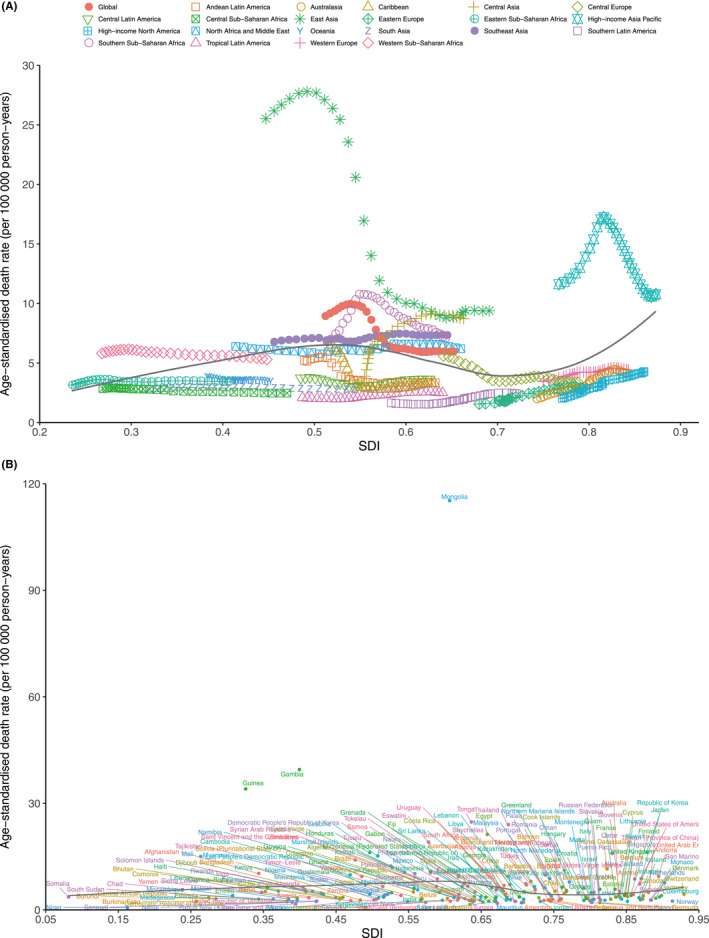
ASMR of primary liver cancer by SDI: (A) ASMR in global and 21 GBD regions, 1990–2019. (B) ASMR in 204 countries and territories, 2019. Expected values based on sociodemographic index and disease rates in all locations are shown as the black line. ASMR, age‐standardized mortality rate; GBD, global burden of diseases, injuries, and risk factors study; SDI, sociodemographic index

Generally, nonlinear associations between the ASDR of liver cancer and the SDI from 1990 to 2019 were observed at the global and regional levels. The highest and lowest ASDRs were observed when the SDI values were 0.52 and 0.71, respectively; the ASDR then decreased and increased with improvement in the SDI. At the regional level, the observed burden of liver cancer in East Asia, high‐income Asia Pacific, Southern Sub‐Saharan Africa, and Southeast Asia was higher than the expected level based on the SDIs between 1990 and 2019. The burden of liver cancer in East Asia, high‐income Asia Pacific, and Southern Sub‐Saharan Africa initially increased and then decreased as the SDI improved over time. At the global level, the observed burden of liver cancer was higher than expected level based on the SDIs from 1990 to 2019 (Figure 6A).

At the national level, a nonlinear association was also found between the ASMR and the SDI value. Mongolia, Gambia, Guinea, and many other countries had a higher than expected ASMR, whereas Niger, Cameroon, Botswana, and many other countries had a lower than expected ASMR based on the SDI (Figure 6B). Nonlinear associations between the SDI and ASIR and ASDR of liver cancer were also observed (Figures S20 and S21).

## 4 DISCUSSION

In this study, we present the most up‐to‐date estimates of the numbers and ASRs of liver cancer mortality, incidence, and DALYs stratified by etiology in 204 countries and territories from 1990 to 2019. Globally, there were approximately 0.49 million mortalities, 0.53 million incident cases, and 12.53 million DALYs in 2019. The trends in ASRs for liver cancer continuously decreased from 1990 to 2019, although they varied according to sex, age, etiology, region, and country. From 1990 to 2019, the number of mortalities of LCHB, LCHC, LCAU, LCNA, and LCOC were all increased, while the ASMR decreased, possibly due to population growth and aging. The increased absolute case numbers may increase the burden of health‐care worker and lead to low quality healthcare, such as inaccurate diagnosis, unnecessary or inappropriate treatment, and medication errors. These situations are prevalent in low‐ and middle‐income countries. Therefore, more resources should be allocated to improve health‐care quality.

The temporal trends in ASR of liver cancer varied across the five etiologies and the world. Consistent with previous studies, HBV and HCV are still the primary causes of liver cancer burden.^5^, ^7^ In 2017, the proportion of liver cancer‐related mortalities due to HBV and HCV was 68.2%,^5^ and this proportion was relatively stable in 2019, at 68.83%. Previous study reported that HBV is endemic in East Asia, and 68.3% of the total global deaths due to LCHB occur there.^14^ Our study also found that China is the top contributor to mortalities due to LCHB, given that it has the largest population. However, the highest ASMR of LCHB was found in neighboring Mongolia. From 1990 to 2019, the percentage change in mortalities, incident cases, and ASRs in China were all decreased, in contrast, in Mongolia these values increased. This is mainly because China initiated a series of HBV programs to address the problem of HBV, such as providing free‐of‐charge HBV vaccination to all newborns since 2005 and conducting a “catch‐up HBV vaccination” program in 2009 for children aged 8–15 years.^8^, ^15^ Although Mongolia introduced an HBV vaccination program for newborns and children under 1 year old in 1991, the burden of LCHB has remained relatively high, mainly because of the limited coverage of the HBV vaccine and lack of adequate medical instruments and equipment.^15^, ^16^, ^17^ Fortunately, the WHO has recommended HBV vaccination as a routine immunization during infancy; 189 countries had introduced the HBV vaccine for infants by the end of 2019, and the global hepatitis B third dose immunization coverage was estimated to be 85%.^18^ In addition, adequate medical instruments and equipment, which improve the ability to diagnose chronic hepatitis and treat HBV, must be provided to reduce the burden of LCHB.

Previous study suggested that HCV is the dominant risk factor for liver cancer in developed countries.^5^, ^7^, ^14^ In the present study, we found that HCV was the predominant etiology not only in developed regions but also in developing regions, such as the high‐income Asia Pacific (Japan) and North Africa and Middle East (Egypt), suggesting that prevention measures should be given priority in some developing countries. This is especially true in Mongolia, which has the highest ASIR and ASDR of LCHC. Unlike HBV, there is currently no effective vaccine to prevent new or re‐infection cases of HCV,^19^ and more prevention measures are needed for LCHC. Strategies to protect against HCV infection include reducing unsafe injections (e.g., reusing unsterilized syringes or needles) and unsafe blood transfusions.^19^, ^20^ In addition, more resources should be allocated to develop an effective HCV vaccine. Although HCV is now curable with direct‐acting antivirals, the high cost of the drugs, drug resistance, and reinfection are key barriers to reducing the burden of HCV around the world, especially in low‐income countries.^20^, ^21^, ^22^ We expect the burden of LCHC to rapidly decrease in the future if more prevention strategies are implemented by countries to meet the goals set by the WHO to eliminate HCV as a public health threat by 2030.^23^

In addition to HBV and HCV, other risk factors for liver cancer include alcohol consumption, NASH, metabolic syndrome, diabetes, obesity, aflatoxin B1, tobacco use, and dietary factors.^24^ Our study found that alcohol use is the third primary cause of liver cancer. Although the ASIR and ASDR remained stable globally, some developed countries, such as the United States and the United Kingdom, had an increasing trend, and this trend is expected to continue increase in the coming years, as previously reported.^25^ This finding highlights the need for policies aimed at reducing harmful alcohol consumption.^6^, ^25^

According to a previous study, the incidence of LCNA has increased in the last few decades, especially in high‐income countries, in parallel with the increasing prevalence of obesity.^26^, ^27^, ^28^ Our study confirmed these results, and most countries, such as Australia, Ireland, and the United Kingdom, had an increasing trend in the ASIR for LCNA from 1990 to 2019. Despite its increased incidence, the diagnosis of NASH remains challenging due to its asymptomatic course, and most patients are diagnosed in the advanced stage of the disease.^29^ Fortunately, we found that the ASDR of LCNA decreased from 1990 to 2019, which may be attributable to the surveillance program that enabled earlier detection of LCNA. However, there has been debate about the cost‐effectiveness and potential harm of this program, owing to over investigation of false‐positive results.^30^, ^31^ Since most NASH is due to obesity, maintaining a healthy lifestyle through exercise and diet is strongly recommended.^31^

In the GBD 2017 study, NASH was estimated as an independent risk factor and was not included in the group of “other causes”.^32^ All etiologies other than the four separately investigated etiologies above were included in the “other causes” group in the GBD 2019 study; these other etiologies included aflatoxin B1 and smoking. Compared with previous GBD study,^5^, ^7^, ^9^ the ASIR of LCOC showed a decreasing trend for the first time in the past 30 years. In addition, mortality exhibited a decreasing trend in the present study, as was also observed in the GBD 2017 study.^5^ This result suggests that aflatoxin exposure and smoking may be reduced to some extent due to AFB1 eradication programs and tobacco control policies.^33^, ^34^ However, more prevention measures should be implemented since the number of incident cases of LCOC continues to increase.

There is a clear sex‐based difference in liver cancer; in general, males have a twofold to fourfold higher incidence of liver cancer than females.^24^ In this study, we found that the numbers of incident cases and deaths in males were 2.4‐fold and 2.2‐fold higher than those in females, respectively. The differences between the two sexes can be attributed to differences in sex steroid hormones, epigenetics, immune responses, and lifestyles (such as alcohol use and smoking, which are more prevalent in males).^35^, ^36^ In addition, the estimated incidence and mortality peaked in relatively old age groups, and the burden of liver cancer has been gradually increasing among populations older than 60 years.^5^, ^6^, ^24^ The highest burden was among males aged 65–69 years and females aged 70–74 years. Hence, these specific groups should be targeted by prevention, management, and treatment policies.

Previous studies have investigated the association between the national level of development, as represented by the Human Development Index, and the incidence rate of liver cancer.^7^ In the present study, we investigated the associations of liver cancer mortality, incidence, and DALYs with the SDI values of regions and countries for the first time. Our study found a nonlinear association between the burden of liver cancer and the SDI values of regions and countries. However, the association between the burden of liver cancer and the SDI value should not be considered in isolation. In fact, the burden of liver cancer is not constrained to developed or less developed regions or countries, and a relatively high burden of liver cancer was observed in regions or countries with a range of SDI values. Furthermore, the observed value and expected levels in each country and region should be compared to judge the effectiveness of prevention programs.

Several limitations of this study should be noted. First, this study was a secondary analysis of data from the GBD study 2019, and as with issues existing in many GBD study, the accuracy and robustness of the results mainly depend on the quality and quantity of input data in modeling. Second, the GBD 2019 study only evaluated the major causes of liver cancer, and the burdens of other and multiple etiologies of liver cancer were not included in this study due to the lack of relevant data. These issues should be investigated in a future GBD study. Third, the effects of prevention and management programs in different countries or regions were not considered, and substantial variations might exist between countries with the same SDI values. Finally, due to the lack of relevant data, the burdens associated with various histological subtypes of liver cancer were not assessed in the current study.

## 5 CONCLUSION

Liver cancer remains a major public health issue worldwide, but there are etiological and geographical variations in the burden of liver cancer. From 1990 to 2019, the global ASRs of incidence, mortality, and DALYs for LCHB, LCHC, and LCOC decreased, and the ASMRs of LCAU and LCNA remained stable. Although the major causes of liver cancer are preventable and treatable, it is necessary to increase the awareness in the population of liver cancer, its etiologies and the importance of early detection, and diagnosis and treatment are needed to reduce the liver cancer burden in the future. The results of our study provide insight into the most up‐to‐date knowledge of the trends in the burden and causes of liver cancer, which may be useful to policy makers who are seeking to develop more cost‐effective and targeted prevention strategies.

## CONFLICT OF INTEREST

The authors declare no conflict of interest.

## AUTHORS CONTRIBUTION

GQOY, JQY, HLX, and GDP conceived, designed, and refined the study protocol. GQOY, GDP, LJG, ZPL, YRW, ZL, WCL, SL, and HLX were involved in the data collection. GQOY and JQY analyzed the data. GQOY and GDP drafted the manuscript. All authors were involved in the critical review of the results and have contributed to, read, and approved the final manuscript.

## ETHICS APPROVAL AND CONSENT TO PARTICIPATE

This study is an analysis of the burden of liver cancer and the data were obtained from the GBD database, so ethics approval and consent to participate were not necessary for this paper.

## CONSENT FOR PUBLICATION

Not applicable.

## Supporting information

Supplementary Material1

Supplementary Material2

## Data Availability

The datasets generated for this study can be found in the GBD at http://ghdx.healthdata.org/gbd‐results‐tool.
