# Supplementary material for: The burden of primary liver cancer caused by specific etiologies from 1990 to 2019 at the global, regional, and national levels
Source: Cancer Med. 2022 Jan 6;11(5):1357–70. doi: 10.1002/cam4.4530 (PMC8894689; doi:10.1002/cam4.4530)
Supplement: Supplementary file 1 — Supplementary Material1 [file CAM4-11-1357-s002.pdf]

Additional file 1

The burden of primary liver cancer caused by specific etiologies from 1990 to 2019 at the Global, Regional, and National Level: results from the Global Burden of Disease Study 2019

List of supplemental Results

Liver cancer due to hepatitis B ……………………………………………………...…2-3

Liver cancer due to hepatitis C………………………………………….…....……….3-4

Liver cancer due to alcohol use…………………………………..……………………….4-5

Liver cancer due to NASH…………………………………....……………………….5-7

Liver cancer due to other cause…………………………………..……………………….7-8

List of supplemental Table

Table S1. The mortality of liver cancer and underlying etiologies between 1990 and 2019 at global, regional level, both sexes ……………………………………………...……..9-19

Table S2. The incidence of liver cancer and underlying etiologies between 1990 and 2019 at global, regional level, both sexes……………………………………………………....19-29

Table S3. The disability-adjusted life years (DALYs) of liver cancer and underlying etiologies between 1990 and 2019 at global, regional level, both sexes……………………………..29-39

Table S4. The mortality of liver cancer and underlying etiologies between 1990 and 2019 at national level, both sexes…………………………………………………………..…..39-129

Table S5. The incidence of liver cancer and underlying etiologies between 1990 and 2019 at national level, both sexes………………………………………………………………130-232

Table S6. The disability-adjusted life years (DALYs) of liver cancer and underlying etiologies between 1990 and 2019 at national level, both sexes………………………………..232-334

Results

Liver cancer due to hepatitis B

In 2019, liver cancer due to hepatitis B(LCHB) accounted for 41.0% of total incident cases globally (Fig.S5). The number of incident cases of LCHB increased by 10.47% from 198,108 in 1990 to 218,855 in 2019, with an age-standardized incidence rate (ASIR) of 4.60 in 1990 per 100,000 population and 2.62 per 100,000 population in 2019, this rate decreased by 42.95% from 1990 to 2019(Fig.S2). At the regional level, the highest ASIR of LCHB per 100,000 population were observed in East Asia, followed by High-income Asia Pacific and Southeast Asia in 2019(Fig.S2). The highest percent changes in ASIR of LCHB was in Central Asia, followed by High-income North America and Australasia from 1990 to 2019. The countries with the highest ASIR of LCHB estimates were Mongolia, Gambia, and Guinea in 2019(Fig.S8B). The most pronounced increased of LCHB ASIR was observed in Cabo Verde, Uzbekistan, and Armenia (Fig.S9B).

The percentage of Liver cancer due to hepatitis B (LCHB) of total Disability-Adjusted Life Years (DALYs) was 46.3% in 2019(Fig.S6). In 2019, the number of DALYs for LCHB decreased by 10.55% from 6,480,909 in 1990 to 5,797,099 in 2019(Fig.S3). The age-standardized DALYs rates (ASDRs) were 145.82 per 100,000 population in 1990 and 69.15 per 100,000 population in 2019, which was decreased by 52,59%

from 1990 to 2019(Fig.S4). The highest ASDR of LCHB per 100,000 population were detected in East Asia, High-income Asia Pacific, and Southern Sub-Saharan Africa in 2019(Fig.S4). The largest increased of ASDR of LCHB were Central Asia, High-income North America, and Australasia between 1990 and 2019. The countries with the highest ASDR of LCHB were Mongolia, Gambia, and Guinea in 2019(Fig.S8C). The most pronounced increased ASDR of LCHB was observed in Cabo Verde, Uzbekistan, and Turkmenistan (Fig.S9C).

Liver cancer due to hepatitis C

hepatitis C (LCHC) makes up 28.5% of total liver cancer incident cases globally in 2019(Fig.S5). The number of incident cases of LCHC increased by 80.68% from 84249 in 1990 to 152225 in 2019, with an ASIR of 2.19 per 100,000 population in 1990 and 1.90 per 100,000 population in 2019, this rate decreased by 13.27% from 1990 to 2019(Fig.S2). At the regions level, High-income Asia Pacific, North Africa and Middle East, and Central Asia showed the highest ASIR of LCHC (Fig.S2). The most pronounced increase of ASIR was in Central Asia, Australasia, and High-income North America. The top three highest ASIRs of LCHC were Mongolia, Egypt, and Japan (Fig.S10B). The largest increased of LCHC ASIR was observed in Cabo Verde, Uzbekistan, and Armenia between

1990 and 2019 (Fig.S11B).

In 2019, hepatitis C account for 23.0% of total liver cancer DALYs globally (Fig.S6). The number of DALYs of LCHB increased by 43.65% from 2,003,448 in 1990 to 2,878,024 in 2019. The ASDR of LCHC was 49.70 per 100,000 population in 1990 and 34.99 per 100,000 population in 2019, which decreased by 29.59% from 1990 to 2019(Fig.S4). At the regions level, the highest ASDR of LCHB were observed in East Asia, followed by High-income Asia Pacific and Central Asia (Fig.S4). The most pronounced increases of ASDR for LCHC was in Central Asia, followed by Australasia and High-income North America. Mongolia, Egypt, and Honduras were countries with the highest ASDR of LCHC in 2019(Fig.S10C). Cabo Verde, Uzbekistan, and Armenia showed the most pronounced increase of LCHC ASDR (Fig.S11C).

Liver cancer due to alcohol use

In 2019, approximately 18.4% total liver cancer incident cases were due to alcohol use globally (Fig.S5). The number of incident case of liver cancer due to alcohol use (LCAU) increased by 103.69% from 48,338 in 1990 to 98,463 in 2019, with an ASIR decreased by 0.44% from 1.20 per 100,000 population in 1990 to 1.19 per 100,000 population in 2019.

Central Asia, High-income Asia Pacific, and Western Europe were the countries with highest ASIR of LCAU (Fig.S2). The highest increases in LCAU ASIR were Central Asia, High-income North America, and Eastern Europe. With respect to countries, the highest ASIR of LCAU was detected in Mongolia, Gambia, and Thailand in 2019(Fig.S12B), with the highest increases was found in Cabo Verde, Uzbekistan, and Armenia (Fig.S13B).

Globally, alcohol use accounted for 17.4% of total liver cancer DALYs in 2019(Fig.S6). The absolute number of DALYs of LCAU increased by 71.10% from 1,274,808 in 1990 to 2,181,197 in 2019, with an ASDR of 30.59 per 100,000 populations in 1990 and 26.07 per 100,000 populations in 2019, this rate decreased by 14.79% from 1990 to 2019(Fig.S4). Across the 21 GBD regions, Central Asia, Southeast Asia, and Australasia were regions with the highest ASDR of LCAU (Fig.S4). The most pronounced increased ASDR was in Central Asia, High-income North America, and Eastern Europe. The highest ASDR of LCAU was found in Mongolia, Gambia, Thailand, whereas the largest increase was observed in Cabo Verde, Uzbekistan, and Armenia (Fig.S12C; Fig.S13C).

Liver cancer due to NASH

Globally in 2019, about 6.8% of total liver cancer incident cases was

ascribed to NASH (Fig.S5). LCNA incident cases increased by 105.34% from 17,697 in 1990 and 36,339 in 2019. The ASIR was 0.44 per 100,000 populations in 1990, which increased by 1.19% from 0.45 per 100,000 populations in 2019(Fig.S2). For 21 GBD regions, the most prominent ASIR of LCNA were seen in Southern Sub-Saharan Africa, followed by High-income Asia Pacific and Southeast Asia (Fig.S2). The largest increase in ASIR of LCNA was in Central Asia, followed by Australasia and High-income North America from. The top three highest ASIR countries were in Mongolia, Gambia, and Qatar in 2019(Fig.S14B)., and the most pronounced increases ASIR of LCNA was Cabo Verde, Uzbekistan, and Armenia (Fig.S15B).

In 2019, the number of LCNA DALYs was 795,806, which accounted for 6.4% of total liver cancer DALYs, and this number increased 66.16% from 478939 in 1990(Fig.S6). The ASDR was 11.35 per 100,000 populations in 1990 and 9.64 per 100,000 populations in 2019, which decreased by 15.09% from 1990 to 2019(Fig.S4). The highest ASDR of LCNA were Southern Sub-Saharan Africa, Southeast Asia and North Africa and Middle East (Fig.S4). Central Asia, Australasia, and High-income North America showed the largest increase from 1990 to 2019. The greatest increases in ASDR of LCNA was in Cabo Verde, Uzbekistan, and Armenia between 1990 and 2019(Fig.S14C), whereas the highest ASDR

of LCNA was found in Mongolia, Gambia, and Guinea in 2019(Fig.S15C).

Liver cancer due to other cause

In 2019, the percentage of liver cancer due to other cause (LCOC) is 5.3% of total incident cases globally (Fig.S5). A total of 28,482 new cases occurred globally in 2019, which increased by 13.94% from 24,998 incident cases in 1990. The ASIR decreased by 36.49% from 0.56 per 100,000 populations in 1990 to 0.35 per 100,000 populations in 2019(Fig.S2). Across the GBD regions, the highest ASIR of LCOC per 100,000 population were observed in East Asia, followed by High-income Asia Pacific and High-income North America (Fig.S2). The most pronounced increases in ASIR of LCOC was in Australasia, Central Asia, and High-income North America. In 2019, Mongolia, Gambia, and Guinea had the highest ASIR of LCOC (Fig.S16B). In addition, Cabo Verde, Uzbekistan, and Eswatini showed the largest increase in ASIR of LCOC (Fig.S17B).

Globally, liver cancer due to other cause accounted for 7.0% of total DALYs in 2019(Fig.S6). The number of DALYs of LCOC decreased from 1,040,525 in 1990 and 876,295 in 2019, which was decreased by 15.78%. The estimated ASDR of LCOC was 20.89 in 1990 and 11.24 in 2019, and

this rate decreased by 46.2% from 1990 to 2019(Fig.S4). The highest ASDR of LCOC per 100,000 population were found in East Asia, followed by North Africa and Middle East and Southern Sub-Saharan Africa (Fig.S4). The most pronounced increase in ASDR of LCOC was in Australasia, High-income North America, and Central Asia between 1990 and 2019. Mongolia, Gambia, and Guinea were the countries with highest ASDR of LCOC (Fig.S16C). Cabo Verde, Uzbekistan, and Armenia showed the largest increased LCOC ASDR (Fig.S17C).

Table S1. The mortality of liver cancer and underlying etiologies between 1990 and 2019 at global, regional level, both sexes							
**regions**	**Causes**	**Cases in 1990**	**Cases in 2019**	**Change in absolute number (95% UI)**	**ASRs in 1990**	**ASRs in 2019**	**change in ASR per 100 000 population (95% UI)**
Global	Liver cancer	365215(329967 to 405774)	484577(444091 to 525798)	0.33(0.15 to 0.54)	8.93(8.09 to 9.9)	5.95(5.44 to 6.44)	-0.05(-0.17 to 0.07)
Global	Liver cancer due to alcohol use	47858(38590 to 58606)	90740(73349 to 109402)	0.9(0.72 to 1.11)	1.2(0.97 to 1.46)	1.1(0.89 to 1.33)	-0.42(-0.51 to -0.32)
Global	Liver cancer due to hepatitis B	190291(162332 to 222448)	191736(161861 to 223727)	0.01(-0.17 to 0.25)	4.47(3.82 to 5.22)	2.31(1.95 to 2.69)	-0.48(-0.58 to -0.37)
Global	Liver cancer due to hepatitis C	84665(73797 to 96590)	141811(121787 to 161828)	0.67(0.5 to 0.88)	2.25(1.97 to 2.54)	1.78(1.53 to 2.04)	-0.21(-0.29 to -0.11)
Global	Liver cancer due to NASH	17800(14647 to 21515)	34729(28395 to 43182)	0.95(0.7 to 1.23)	0.46(0.38 to 0.55)	0.43(0.35 to 0.53)	-0.08(-0.16 to 0.02)
Global	Liver cancer due to other causes	24599(20584 to 29473)	25560(21229 to 30491)	0.04(-0.13 to 0.23)	0.55(0.46 to 0.66)	0.32(0.27 to 0.38)	-0.33(-0.42 to -0.23)
Andean Latin America	Liver cancer	1075(946 to 1211)	1840(1510 to 2232)	0.71(0.36 to 1.14)	5.23(4.6 to 5.87)	3.34(2.73 to 4.03)	-0.16(-0.34 to 0.06)
Andean Latin America	Liver cancer due to alcohol use	314(222 to 417)	593(408 to 823)	0.89(0.47 to 1.42)	1.61(1.15 to 2.14)	1.09(0.75 to 1.51)	-0.43(-0.55 to -0.29)
Andean Latin America	Liver cancer due to hepatitis B	504(400 to 622)	758(548 to 996)	0.5(0.18 to 0.89)	2.37(1.84 to 2.98)	1.35(0.97 to 1.78)	-0.31(-0.45 to -0.14)
Andean Latin America	Liver cancer due to hepatitis C	75(47 to 111)	152(94 to 232)	1.02(0.6 to 1.56)	0.41(0.25 to 0.61)	0.28(0.18 to 0.43)	-0.33(-0.47 to -0.14)
Andean Latin America	Liver cancer due to NASH	83(58 to 116)	198(135 to 282)	1.37(0.84 to 2.01)	0.44(0.3 to 0.62)	0.37(0.25 to 0.52)	-0.37(-0.5 to -0.18)

Andean Latin America	Liver cancer due to other causes	98(76 to 128)	139(97 to 191)	0.41(0.11 to 0.82)	0.39(0.28 to 0.53)	0.25(0.17 to 0.34)	-0.36(-0.49 to -0.21)
Australasia	Liver cancer	464(445 to 481)	2006(1832 to 2174)	3.33(2.98 to 3.67)	1.98(1.9 to 2.06)	4.12(3.8 to 4.46)	1.08(0.91 to 1.25)
Australasia	Liver cancer due to alcohol use	197(154 to 235)	765(596 to 934)	2.88(2.46 to 3.32)	0.83(0.66 to 0.99)	1.58(1.24 to 1.93)	1.91(1.5 to 2.31)
Australasia	Liver cancer due to hepatitis B	71(51 to 95)	252(179 to 350)	2.56(2.14 to 2.97)	0.31(0.23 to 0.41)	0.56(0.4 to 0.77)	0.93(0.69 to 1.17)
Australasia	Liver cancer due to hepatitis C	137(105 to 173)	677(512 to 863)	3.93(3.47 to 4.45)	0.59(0.45 to 0.73)	1.33(1 to 1.69)	0.82(0.62 to 1.02)
Australasia	Liver cancer due to NASH	36(27 to 49)	224(163 to 300)	5.22(4.27 to 6.19)	0.15(0.11 to 0.21)	0.45(0.33 to 0.59)	1.27(1.07 to 1.49)
Australasia	Liver cancer due to other causes	22(18 to 29)	88(64 to 118)	2.9(2.3 to 3.47)	0.1(0.08 to 0.13)	0.2(0.15 to 0.26)	0.91(0.7 to 1.12)
Caribbean	Liver cancer	1636(1521 to 1735)	1695(1418 to 2005)	0.04(-0.12 to 0.22)	6.29(5.85 to 6.68)	3.29(2.76 to 3.89)	-0.5(-0.59 to -0.38)
Caribbean	Liver cancer due to alcohol use	535(399 to 685)	594(428 to 796)	0.11(-0.08 to 0.34)	2.06(1.53 to 2.64)	1.15(0.83 to 1.54)	-0.52(-0.59 to -0.44)
Caribbean	Liver cancer due to hepatitis B	454(338 to 598)	441(307 to 609)	-0.03(-0.2 to 0.18)	1.7(1.26 to 2.25)	0.85(0.6 to 1.18)	-0.44(-0.54 to -0.33)
Caribbean	Liver cancer due to hepatitis C	391(277 to 520)	388(263 to 537)	-0.01(-0.16 to 0.16)	1.56(1.11 to 2.07)	0.75(0.51 to 1.04)	-0.41(-0.5 to -0.29)
Caribbean	Liver cancer due to NASH	138(98 to 190)	165(112 to 234)	0.19(-0.01 to 0.42)	0.54(0.38 to 0.75)	0.32(0.22 to 0.45)	-0.49(-0.58 to -0.39)
Caribbean	Liver cancer due to other causes	118(88 to 155)	107(75 to 149)	-0.1(-0.25 to 0.07)	0.42(0.31 to 0.56)	0.21(0.15 to 0.3)	-0.48(-0.56 to -0.38)
Central Asia	Liver cancer	1507(1348 to 1663)	6191(5387 to 7076)	3.11(2.47 to 3.82)	3.24(2.89 to 3.58)	8.72(7.63 to 9.88)	1.7(1.3 to 2.15)

Central Asia	Liver cancer due to alcohol use	404(281 to 532)	1863(1315 to 2505)	3.61(2.85 to 4.5)	0.88(0.61 to 1.16)	2.6(1.86 to 3.39)	1.3(0.91 to 1.77)
Central Asia	Liver cancer due to hepatitis B	431(315 to 589)	1651(1177 to 2214)	2.83(2.15 to 3.59)	0.89(0.64 to 1.22)	2.05(1.47 to 2.81)	1.75(1.34 to 2.24)
Central Asia	Liver cancer due to hepatitis C	494(353 to 637)	1990(1422 to 2599)	3.03(2.44 to 3.74)	1.12(0.82 to 1.43)	3.08(2.25 to 3.95)	1.95(1.48 to 2.48)
Central Asia	Liver cancer due to NASH	82(57 to 117)	436(307 to 616)	4.33(3.5 to 5.26)	0.18(0.13 to 0.27)	0.67(0.47 to 0.95)	2.61(2.05 to 3.25)
Central Asia	Liver cancer due to other causes	97(72 to 124)	251(179 to 349)	1.58(0.91 to 2.55)	0.16(0.12 to 0.21)	0.33(0.23 to 0.45)	1.09(0.64 to 1.72)
Central Europe	Liver cancer	8114(7830 to 8322)	7202(6218 to 8327)	-0.11(-0.23 to 0.02)	5.6(5.38 to 5.75)	3.36(2.9 to 3.9)	-0.46(-0.54 to -0.37)
Central Europe	Liver cancer due to alcohol use	3159(2626 to 3672)	3092(2400 to 3851)	-0.02(-0.16 to 0.13)	2.13(1.78 to 2.47)	1.43(1.11 to 1.77)	-0.45(-0.53 to -0.36)
Central Europe	Liver cancer due to hepatitis B	1864(1457 to 2357)	1371(974 to 1905)	-0.26(-0.38 to -0.13)	1.27(1 to 1.6)	0.68(0.5 to 0.94)	-0.33(-0.42 to -0.22)
Central Europe	Liver cancer due to hepatitis C	2203(1750 to 2707)	1926(1408 to 2566)	-0.13(-0.25 to 0.02)	1.57(1.27 to 1.9)	0.86(0.63 to 1.15)	-0.4(-0.48 to -0.31)
Central Europe	Liver cancer due to NASH	595(465 to 754)	597(430 to 818)	0(-0.15 to 0.17)	0.42(0.33 to 0.53)	0.27(0.2 to 0.37)	-0.35(-0.45 to -0.25)
Central Europe	Liver cancer due to other causes	293(231 to 365)	215(151 to 297)	-0.27(-0.38 to -0.13)	0.21(0.17 to 0.26)	0.12(0.08 to 0.15)	-0.46(-0.53 to -0.37)
Central Latin America	Liver cancer	3074(2858 to 3233)	8416(7357 to 9750)	1.74(1.4 to 2.15)	3.74(3.46 to 3.94)	3.65(3.18 to 4.22)	-0.18(-0.29 to -0.03)
Central Latin America	Liver cancer due to alcohol use	861(662 to 1058)	2587(2003 to 3264)	2.01(1.59 to 2.5)	1.06(0.82 to 1.31)	1.12(0.86 to 1.41)	-0.05(-0.16 to 0.09)
Central Latin America	Liver cancer due to hepatitis B	537(409 to 698)	1156(859 to 1596)	1.15(0.85 to 1.54)	0.59(0.44 to 0.78)	0.48(0.36 to 0.67)	0.05(-0.09 to 0.22)

Central Latin America	Liver cancer due to hepatitis C	1190(971 to 1414)	3385(2743 to 4109)	1.85(1.5 to 2.28)	1.57(1.29 to 1.85)	1.49(1.21 to 1.8)	-0.02(-0.14 to 0.12)
Central Latin America	Liver cancer due to NASH	219(168 to 284)	784(612 to 1014)	2.58(2.09 to 3.12)	0.27(0.21 to 0.36)	0.34(0.27 to 0.44)	0.25(0.08 to 0.44)
Central Latin America	Liver cancer due to other causes	267(227 to 314)	504(388 to 629)	0.88(0.61 to 1.24)	0.24(0.19 to 0.3)	0.21(0.17 to 0.27)	-0.1(-0.23 to 0.05)
Central Sub-Saharan Africa	Liver cancer	717(598 to 854)	1394(1108 to 1753)	0.95(0.47 to 1.57)	2.85(2.43 to 3.29)	2.47(1.99 to 3.07)	-0.2(-0.39 to 0.06)
Central Sub-Saharan Africa	Liver cancer due to alcohol use	85(55 to 123)	183(117 to 268)	1.16(0.63 to 1.91)	0.39(0.26 to 0.55)	0.35(0.23 to 0.51)	-0.12(-0.31 to 0.12)
Central Sub-Saharan Africa	Liver cancer due to hepatitis B	179(129 to 244)	355(243 to 518)	0.98(0.46 to 1.7)	0.66(0.47 to 0.92)	0.53(0.36 to 0.77)	-0.09(-0.3 to 0.2)
Central Sub-Saharan Africa	Liver cancer due to hepatitis C	272(200 to 350)	565(400 to 746)	1.08(0.58 to 1.72)	1.41(1.08 to 1.74)	1.24(0.92 to 1.6)	0.02(-0.2 to 0.32)
Central Sub-Saharan Africa	Liver cancer due to NASH	36(25 to 53)	89(59 to 131)	1.44(0.82 to 2.3)	0.17(0.12 to 0.25)	0.18(0.12 to 0.26)	-0.21(-0.48 to 0.14)
Central Sub-Saharan Africa	Liver cancer due to other causes	144(97 to 215)	202(119 to 325)	0.41(-0.24 to 1.35)	0.21(0.15 to 0.28)	0.16(0.11 to 0.24)	-0.13(-0.32 to 0.12)
East Asia	Liver cancer	237005(202341 to 279889)	193864(163848 to 228758)	-0.18(-0.36 to 0.05)	25.52(21.98 to 29.94)	9.39(7.98 to 11.03)	-0.64(-0.72 to -0.53)
East Asia	Liver cancer due to alcohol use	17909(13365 to 24174)	17969(13152 to 23272)	0(-0.22 to 0.29)	1.96(1.47 to 2.62)	0.84(0.62 to 1.08)	-0.64(-0.71 to -0.55)
East Asia	Liver cancer due to hepatitis B	155312(128853 to 186543)	120124(97659 to 146171)	-0.23(-0.41 to 0.03)	15.9(13.23 to 19.08)	5.71(4.68 to 6.92)	-0.57(-0.66 to -0.45)
East Asia	Liver cancer due to hepatitis C	38160(31683 to 46292)	34878(28702 to 41388)	-0.09(-0.27 to 0.14)	4.92(4.14 to 5.89)	1.78(1.49 to 2.1)	-0.67(-0.73 to -0.58)

East Asia	Liver cancer due to NASH	9076(7223 to 11293)	9793(7869 to 12103)	0.08(-0.14 to 0.35)	1.05(0.84 to 1.29)	0.49(0.39 to 0.6)	-0.54(-0.62 to -0.42)
East Asia	Liver cancer due to other causes	16548(13471 to 20344)	11100(8925 to 13553)	-0.33(-0.47 to -0.15)	1.7(1.38 to 2.08)	0.57(0.46 to 0.68)	-0.63(-0.71 to -0.53)
Eastern Europe	Liver cancer	4224(4055 to 4412)	9676(8506 to 11122)	1.29(1.04 to 1.58)	1.55(1.49 to 1.62)	2.87(2.51 to 3.29)	0.85(0.65 to 1.08)
Eastern Europe	Liver cancer due to alcohol use	1415(1222 to 1603)	3641(3001 to 4381)	1.57(1.26 to 1.95)	0.5(0.43 to 0.57)	1.06(0.87 to 1.28)	1.14(0.92 to 1.39)
Eastern Europe	Liver cancer due to hepatitis B	1070(904 to 1269)	2115(1680 to 2647)	0.98(0.72 to 1.26)	0.38(0.33 to 0.45)	0.65(0.52 to 0.81)	0.15(-0.07 to 0.4)
Eastern Europe	Liver cancer due to hepatitis C	1161(998 to 1321)	2719(2283 to 3211)	1.34(1.09 to 1.6)	0.43(0.37 to 0.48)	0.77(0.65 to 0.91)	0.7(0.48 to 0.95)
Eastern Europe	Liver cancer due to NASH	302(255 to 359)	820(678 to 1004)	1.71(1.43 to 2.03)	0.11(0.09 to 0.13)	0.24(0.2 to 0.29)	0.8(0.62 to 1)
Eastern Europe	Liver cancer due to other causes	276(248 to 305)	380(307 to 462)	0.38(0.18 to 0.59)	0.13(0.11 to 0.14)	0.15(0.11 to 0.18)	1.11(0.86 to 1.43)
Eastern Sub-Saharan Africa	Liver cancer	2537(2076 to 3145)	5677(4683 to 6919)	1.24(0.74 to 1.82)	3.15(2.63 to 3.99)	3.41(2.85 to 4.15)	0.08(-0.11 to 0.32)
Eastern Sub-Saharan Africa	Liver cancer due to alcohol use	589(419 to 843)	1380(980 to 1929)	1.34(0.9 to 1.91)	0.82(0.59 to 1.16)	0.89(0.64 to 1.23)	0.01(-0.18 to 0.26)
Eastern Sub-Saharan Africa	Liver cancer due to hepatitis B	716(532 to 979)	1616(1189 to 2158)	1.26(0.79 to 1.87)	0.85(0.62 to 1.17)	0.86(0.62 to 1.16)	0.11(-0.09 to 0.37)
Eastern Sub-Saharan Africa	Liver cancer due to hepatitis C	547(388 to 730)	1276(952 to 1637)	1.33(0.83 to 1.93)	0.86(0.63 to 1.13)	0.96(0.73 to 1.21)	0.1(-0.09 to 0.34)
Eastern Sub-Saharan Africa	Liver cancer due to NASH	240(177 to 328)	631(458 to 842)	1.63(1.13 to 2.27)	0.35(0.26 to 0.48)	0.43(0.31 to 0.57)	0.22(0.03 to 0.48)

Eastern Sub-Saharan Africa	Liver cancer due to other causes	445(317 to 624)	774(580 to 995)	0.74(0.09 to 1.52)	0.28(0.2 to 0.36)	0.27(0.21 to 0.35)	-0.01(-0.27 to 0.3)
High-income Asia Pacific	Liver cancer	23589(22762 to 24374)	49685(43778 to 53504)	1.11(0.89 to 1.26)	11.62(11.18 to 12)	10.78(9.77 to 11.53)	0.22(0.05 to 0.41)
High-income Asia Pacific	Liver cancer due to alcohol use	3045(2529 to 3605)	6217(4956 to 7731)	1.04(0.77 to 1.33)	1.47(1.23 to 1.74)	1.42(1.13 to 1.77)	-0.2(-0.27 to -0.15)
High-income Asia Pacific	Liver cancer due to hepatitis B	5274(4589 to 6031)	11624(9714 to 13638)	1.2(0.88 to 1.54)	2.54(2.21 to 2.9)	3.1(2.62 to 3.6)	-0.04(-0.15 to 0.09)
High-income Asia Pacific	Liver cancer due to hepatitis C	13591(12740 to 14365)	27963(23725 to 30944)	1.06(0.79 to 1.22)	6.76(6.33 to 7.14)	5.42(4.7 to 5.99)	0.06(-0.07 to 0.2)
High-income Asia Pacific	Liver cancer due to NASH	962(806 to 1144)	2521(1921 to 3191)	1.62(1.21 to 2.04)	0.48(0.4 to 0.57)	0.51(0.39 to 0.64)	-0.11(-0.22 to -0.01)
High-income Asia Pacific	Liver cancer due to other causes	718(604 to 845)	1360(1067 to 1690)	0.89(0.6 to 1.2)	0.36(0.31 to 0.42)	0.32(0.26 to 0.39)	-0.07(-0.14 to -0.01)
High-income North America	Liver cancer	7074(6778 to 7245)	26479(23637 to 28913)	2.74(2.36 to 3.07)	2.03(1.95 to 2.07)	4.29(3.83 to 4.68)	1.11(0.89 to 1.3)
High-income North America	Liver cancer due to alcohol use	2225(1968 to 2468)	8863(7227 to 10431)	2.98(2.47 to 3.43)	0.64(0.56 to 0.7)	1.44(1.18 to 1.69)	0.88(0.65 to 1.09)
High-income North America	Liver cancer due to hepatitis B	897(771 to 1041)	2914(2351 to 3588)	2.25(1.85 to 2.62)	0.27(0.23 to 0.31)	0.5(0.41 to 0.62)	1.13(0.92 to 1.32)
High-income North America	Liver cancer due to hepatitis C	2551(2276 to 2798)	9754(8361 to 11230)	2.82(2.44 to 3.15)	0.72(0.65 to 0.79)	1.53(1.32 to 1.77)	1.26(0.97 to 1.51)
High-income North America	Liver cancer due to NASH	722(612 to 846)	2889(2354 to 3496)	3(2.58 to 3.34)	0.2(0.17 to 0.23)	0.45(0.37 to 0.55)	1.28(1.04 to 1.47)

High-income North America	Liver cancer due to other causes	680(592 to 771)	2059(1707 to 2440)	2.03(1.72 to 2.32)	0.21(0.18 to 0.23)	0.36(0.3 to 0.42)	0.73(0.57 to 0.89)
North Africa and Middle East	Liver cancer	10913(9575 to 12227)	26432(21211 to 32611)	1.42(0.87 to 2.24)	6.39(5.55 to 7.19)	6.2(5.06 to 7.62)	0.28(-0.02 to 0.76)
North Africa and Middle East	Liver cancer due to alcohol use	968(641 to 1378)	2571(1655 to 3905)	1.66(1.05 to 2.54)	0.58(0.39 to 0.82)	0.61(0.39 to 0.92)	-0.06(-0.26 to 0.26)
North Africa and Middle East	Liver cancer due to hepatitis B	3153(2480 to 3938)	6736(5017 to 8807)	1.14(0.72 to 1.69)	1.72(1.34 to 2.17)	1.46(1.08 to 1.92)	-0.15(-0.31 to 0.07)
North Africa and Middle East	Liver cancer due to hepatitis C	5110(4035 to 6230)	12740(9255 to 17062)	1.49(0.82 to 2.53)	3.2(2.53 to 3.89)	3.11(2.31 to 4.09)	-0.03(-0.29 to 0.35)
North Africa and Middle East	Liver cancer due to NASH	832(599 to 1173)	2720(1890 to 3930)	2.27(1.48 to 3.53)	0.52(0.37 to 0.73)	0.67(0.47 to 0.94)	0.05(-0.19 to 0.39)
North Africa and Middle East	Liver cancer due to other causes	850(654 to 1095)	1665(1185 to 2331)	0.96(0.52 to 1.62)	0.38(0.28 to 0.5)	0.36(0.25 to 0.5)	-0.03(-0.25 to 0.29)
Oceania	Liver cancer	112(94 to 131)	233(195 to 277)	1.07(0.69 to 1.55)	3.85(3.25 to 4.47)	3.46(2.93 to 4.09)	-0.15(-0.32 to 0.04)
Oceania	Liver cancer due to alcohol use	13(8 to 19)	30(19 to 45)	1.28(0.79 to 1.86)	0.47(0.3 to 0.69)	0.46(0.3 to 0.68)	-0.09(-0.25 to 0.12)
Oceania	Liver cancer due to hepatitis B	62(48 to 77)	122(94 to 157)	0.97(0.58 to 1.42)	1.91(1.46 to 2.4)	1.62(1.23 to 2.08)	-0.01(-0.21 to 0.24)
Oceania	Liver cancer due to hepatitis C	23(15 to 31)	48(32 to 69)	1.12(0.75 to 1.62)	0.99(0.67 to 1.32)	0.9(0.62 to 1.23)	-0.12(-0.27 to 0.08)
Oceania	Liver cancer due to NASH	8(5 to 12)	20(13 to 28)	1.47(1.03 to 2.05)	0.31(0.21 to 0.44)	0.33(0.23 to 0.47)	-0.1(-0.27 to 0.11)
Oceania	Liver cancer due to other causes	7(5 to 9)	13(10 to 18)	0.95(0.6 to 1.38)	0.17(0.12 to 0.24)	0.15(0.11 to 0.21)	0.07(-0.12 to 0.31)

South Asia	Liver cancer	15854(13379 to 18088)	38650(33517 to 44561)	1.44(1.04 to 1.89)	2.82(2.33 to 3.27)	2.81(2.43 to 3.24)	0.15(-0.07 to 0.37)
South Asia	Liver cancer due to alcohol use	3593(2789 to 4444)	10076(8092 to 12256)	1.8(1.28 to 2.47)	0.66(0.51 to 0.82)	0.73(0.59 to 0.89)	-0.02(-0.17 to 0.13)
South Asia	Liver cancer due to hepatitis B	5788(4757 to 6878)	12362(10136 to 14956)	1.14(0.75 to 1.57)	0.93(0.74 to 1.11)	0.84(0.69 to 1.03)	-0.01(-0.17 to 0.2)
South Asia	Liver cancer due to hepatitis C	3742(2891 to 4605)	9905(8086 to 11943)	1.65(1.14 to 2.19)	0.81(0.63 to 1.01)	0.78(0.64 to 0.94)	-0.09(-0.26 to 0.11)
South Asia	Liver cancer due to NASH	1404(1115 to 1724)	4157(3350 to 5054)	1.96(1.41 to 2.52)	0.28(0.21 to 0.34)	0.32(0.25 to 0.39)	-0.04(-0.24 to 0.16)
South Asia	Liver cancer due to other causes	1327(1137 to 1535)	2152(1815 to 2523)	0.62(0.34 to 0.94)	0.14(0.12 to 0.16)	0.14(0.12 to 0.16)	0.1(-0.11 to 0.36)
Southeast Asia	Liver cancer	17574(15676 to 19279)	42862(35326 to 51520)	1.44(0.96 to 2)	6.76(6.03 to 7.45)	7.33(6.08 to 8.79)	0.08(-0.13 to 0.34)
Southeast Asia	Liver cancer due to alcohol use	3622(2679 to 4771)	11175(7880 to 15392)	2.08(1.36 to 2.93)	1.44(1.08 to 1.88)	1.9(1.34 to 2.59)	-0.08(-0.31 to 0.17)
Southeast Asia	Liver cancer due to hepatitis B	7780(6337 to 9326)	16439(12182 to 21724)	1.11(0.64 to 1.69)	2.75(2.2 to 3.33)	2.6(1.93 to 3.43)	-0.06(-0.26 to 0.2)
Southeast Asia	Liver cancer due to hepatitis C	3929(2991 to 4958)	9709(7328 to 12702)	1.47(1.03 to 2.01)	1.78(1.39 to 2.21)	1.84(1.39 to 2.39)	0.03(-0.15 to 0.26)
Southeast Asia	Liver cancer due to NASH	1244(942 to 1634)	4080(2896 to 5630)	2.28(1.59 to 3.15)	0.52(0.39 to 0.7)	0.75(0.53 to 1.03)	0.32(0.02 to 0.68)
Southeast Asia	Liver cancer due to other causes	997(764 to 1346)	1459(1091 to 1934)	0.46(0 to 0.98)	0.27(0.21 to 0.35)	0.25(0.18 to 0.33)	0.42(0.13 to 0.8)
Southern Latin America	Liver cancer	755(683 to 829)	2027(1897 to 2152)	1.68(1.42 to 2.03)	1.65(1.49 to 1.81)	2.41(2.26 to 2.56)	0.28(0.12 to 0.46)
Southern Latin America	Liver cancer due to alcohol use	251(179 to 328)	652(472 to 832)	1.6(1.26 to 1.99)	0.54(0.39 to 0.71)	0.77(0.56 to 0.98)	0.84(0.62 to 1.12)

Southern Latin America	Liver cancer due to hepatitis B	136(94 to 194)	323(223 to 467)	1.38(1.08 to 1.73)	0.29(0.2 to 0.41)	0.39(0.27 to 0.56)	0.46(0.32 to 0.65)
Southern Latin America	Liver cancer due to hepatitis C	258(189 to 339)	739(544 to 935)	1.87(1.55 to 2.26)	0.58(0.43 to 0.75)	0.87(0.64 to 1.1)	0.35(0.19 to 0.54)
Southern Latin America	Liver cancer due to NASH	58(39 to 85)	200(137 to 285)	2.48(2.02 to 3.03)	0.13(0.09 to 0.19)	0.24(0.16 to 0.33)	0.5(0.34 to 0.69)
Southern Latin America	Liver cancer due to other causes	52(40 to 69)	112(79 to 154)	1.14(0.83 to 1.47)	0.11(0.08 to 0.15)	0.14(0.1 to 0.19)	0.43(0.26 to 0.65)
Southern Sub-Saharan Africa	Liver cancer	1913(1349 to 3140)	4040(3618 to 4540)	1.11(0.21 to 2.19)	6.74(4.71 to 11.07)	7.05(6.31 to 7.91)	0.22(-0.27 to 0.83)
Southern Sub-Saharan Africa	Liver cancer due to alcohol use	363(219 to 695)	795(631 to 989)	1.19(0.11 to 2.54)	1.31(0.79 to 2.5)	1.4(1.11 to 1.71)	0.05(-0.4 to 0.58)
Southern Sub-Saharan Africa	Liver cancer due to hepatitis B	744(498 to 1298)	1497(1251 to 1788)	1.01(0.07 to 2.13)	2.36(1.56 to 4.13)	2.35(1.95 to 2.81)	-0.01(-0.46 to 0.55)
Southern Sub-Saharan Africa	Liver cancer due to hepatitis C	514(351 to 776)	1103(905 to 1315)	1.15(0.39 to 2.1)	2.07(1.41 to 3.1)	2.16(1.8 to 2.57)	0.05(-0.32 to 0.52)
Southern Sub-Saharan Africa	Liver cancer due to NASH	177(116 to 287)	434(351 to 529)	1.45(0.48 to 2.65)	0.66(0.43 to 1.07)	0.8(0.65 to 0.97)	0.07(-0.46 to 0.73)
Southern Sub-Saharan Africa	Liver cancer due to other causes	115(84 to 176)	212(172 to 265)	0.83(0.16 to 1.59)	0.34(0.24 to 0.53)	0.34(0.28 to 0.42)	-0.01(-0.38 to 0.42)
Tropical Latin America	Liver cancer	1885(1803 to 1949)	5939(5543 to 6239)	2.15(1.97 to 2.35)	2.09(1.99 to 2.17)	2.5(2.32 to 2.62)	0.02(-0.04 to 0.1)
Tropical Latin America	Liver cancer due to alcohol use	517(447 to 591)	1822(1566 to 2068)	2.52(2.3 to 2.77)	0.58(0.5 to 0.65)	0.76(0.65 to 0.86)	0.2(0.13 to 0.28)
Tropical Latin America	Liver cancer due to hepatitis B	409(352 to 469)	1018(857 to 1196)	1.49(1.32 to 1.69)	0.41(0.35 to 0.47)	0.41(0.35 to 0.49)	0.31(0.23 to 0.41)

Tropical Latin America	Liver cancer due to hepatitis C	704(626 to 788)	2419(2115 to 2702)	2.44(2.23 to 2.66)	0.86(0.77 to 0.96)	1.03(0.9 to 1.15)	0.35(0.27 to 0.43)
Tropical Latin America	Liver cancer due to NASH	104(88 to 121)	380(321 to 449)	2.67(2.43 to 2.92)	0.12(0.1 to 0.14)	0.16(0.14 to 0.19)	0.02(-0.06 to 0.1)
Tropical Latin America	Liver cancer due to other causes	151(136 to 167)	300(261 to 343)	0.99(0.8 to 1.18)	0.13(0.11 to 0.15)	0.13(0.11 to 0.15)	0.19(0.12 to 0.26)
Western Europe	Liver cancer	19883(19160 to 20419)	40296(37224 to 42876)	1.03(0.91 to 1.15)	3.43(3.31 to 3.52)	4.41(4.1 to 4.68)	0.29(0.21 to 0.36)
Western Europe	Liver cancer due to alcohol use	6916(5606 to 8168)	14022(11220 to 16807)	1.03(0.87 to 1.19)	1.19(0.97 to 1.4)	1.58(1.27 to 1.88)	0.53(0.42 to 0.65)
Western Europe	Liver cancer due to hepatitis B	2437(1848 to 3191)	4299(3127 to 5823)	0.76(0.6 to 0.93)	0.45(0.34 to 0.58)	0.53(0.39 to 0.71)	0.19(0.09 to 0.29)
Western Europe	Liver cancer due to hepatitis C	8581(7308 to 9927)	17568(14479 to 20788)	1.05(0.89 to 1.19)	1.45(1.24 to 1.68)	1.81(1.49 to 2.15)	0.25(0.17 to 0.33)
Western Europe	Liver cancer due to NASH	1115(843 to 1473)	2755(2010 to 3732)	1.47(1.25 to 1.69)	0.19(0.14 to 0.25)	0.29(0.21 to 0.38)	0.33(0.23 to 0.43)
Western Europe	Liver cancer due to other causes	834(640 to 1080)	1652(1222 to 2213)	0.98(0.81 to 1.16)	0.16(0.13 to 0.2)	0.2(0.16 to 0.26)	0.27(0.18 to 0.37)
Western Sub-Saharan Africa	Liver cancer	5308(4553 to 6165)	9972(8360 to 11564)	0.88(0.51 to 1.29)	5.81(4.99 to 6.76)	5.29(4.48 to 6.04)	-0.15(-0.34 to 0.05)
Western Sub-Saharan Africa	Liver cancer due to alcohol use	878(636 to 1206)	1849(1327 to 2450)	1.11(0.61 to 1.65)	1.03(0.75 to 1.42)	1.06(0.77 to 1.4)	-0.11(-0.26 to 0.08)
Western Sub-Saharan Africa	Liver cancer due to hepatitis B	2473(1966 to 3031)	4563(3567 to 5703)	0.85(0.44 to 1.29)	2.65(2.1 to 3.26)	2.26(1.74 to 2.82)	0.02(-0.21 to 0.28)
Western Sub-Saharan Africa	Liver cancer due to hepatitis C	1033(745 to 1350)	1906(1372 to 2462)	0.85(0.52 to 1.22)	1.34(0.99 to 1.72)	1.2(0.88 to 1.51)	-0.15(-0.31 to 0.02)
Western Sub-Saharan	Liver cancer due to NASH	367(271 to 488)	838(627 to 1118)	1.28(0.85 to 1.82)	0.45(0.33 to 0.6)	0.5(0.37 to 0.66)	0.1(-0.11 to 0.35)

Africa							
Western Sub-Saharan Africa	Liver cancer due to other causes	558(447 to 695)	816(621 to 1049)	0.46(0.11 to 0.85)	0.33(0.26 to 0.42)	0.28(0.21 to 0.37)	-0.09(-0.26 to 0.11)

Table S2. The incidence of liver cancer and underlying etiologies between 1990 and 2019 at global, regional level, both sexes

**regions**	**Causes**	**Cases in 1990**	**Cases in 2019**	**Change in absolute number (95% UI)**	**ASRs in 1990**	**ASRs in 2019**	**change in ASR per 100 000 population (95% UI)**
Global	Liver cancer	373390(335890 to 415748)	534364(486550 to 588639)	0.43(0.23 to 0.67)	8.98(8.1 to 9.97)	6.51(5.95 to 7.16)	-0.27(-0.37 to -0.16)
Global	Liver cancer due to alcohol use	48338(38845 to 59086)	98463(79034 to 120127)	1.04(0.8 to 1.3)	1.2(0.96 to 1.47)	1.19(0.96 to 1.45)	0(-0.12 to 0.12)
Global	Liver cancer due to hepatitis B	198107(165914 to 232200)	218855(186488 to 254886)	0.1(-0.11 to 0.37)	4.6(3.86 to 5.39)	2.62(2.24 to 3.05)	-0.43(-0.54 to -0.29)
Global	Liver cancer due to hepatitis C	84249(72741 to 96021)	152225(131581 to 174627)	0.81(0.62 to 1.04)	2.19(1.9 to 2.49)	1.9(1.64 to 2.17)	-0.13(-0.22 to -0.02)
Global	Liver cancer due to NASH	17697(14528 to 21175)	36339(29494 to 44855)	1.05(0.79 to 1.35)	0.44(0.36 to 0.53)	0.45(0.37 to 0.55)	0.01(-0.11 to 0.15)
Global	Liver cancer due to other causes	24998(20963 to 29798)	28482(23574 to 34082)	0.14(-0.04 to 0.34)	0.56(0.47 to 0.67)	0.35(0.29 to 0.42)	-0.36(-0.46 to -0.26)
Andean Latin America	Liver cancer	1036(910 to 1168)	1735(1419 to 2114)	0.67(0.33 to 1.11)	4.9(4.31 to 5.52)	3.11(2.55 to 3.78)	-0.36(-0.5 to -0.2)
Andean Latin America	Liver cancer due to alcohol use	295(207 to 394)	549(376 to 755)	0.86(0.45 to 1.39)	1.49(1.05 to 1.99)	1(0.69 to 1.38)	-0.33(-0.47 to -0.14)

Andean Latin America	Liver cancer due to hepatitis B	502(401 to 617)	740(539 to 971)	0.48(0.15 to 0.86)	2.29(1.8 to 2.85)	1.31(0.95 to 1.71)	-0.43(-0.55 to -0.28)
Andean Latin America	Liver cancer due to hepatitis C	68(42 to 101)	133(81 to 204)	0.96(0.55 to 1.5)	0.36(0.22 to 0.53)	0.25(0.15 to 0.38)	-0.32(-0.46 to -0.14)
Andean Latin America	Liver cancer due to NASH	77(54 to 108)	177(122 to 252)	1.29(0.77 to 1.92)	0.39(0.27 to 0.55)	0.33(0.22 to 0.46)	-0.17(-0.35 to 0.06)
Andean Latin America	Liver cancer due to other causes	95(71 to 122)	135(95 to 182)	0.42(0.12 to 0.83)	0.37(0.26 to 0.5)	0.24(0.16 to 0.32)	-0.36(-0.49 to -0.17)
Australasia	Liver cancer	476(457 to 494)	2160(1752 to 2667)	3.54(2.7 to 4.59)	2.05(1.97 to 2.13)	4.59(3.72 to 5.68)	1.24(0.82 to 1.77)
Australasia	Liver cancer due to alcohol use	202(160 to 241)	845(616 to 1132)	3.18(2.33 to 4.21)	0.86(0.68 to 1.02)	1.79(1.31 to 2.42)	1.09(0.67 to 1.63)
Australasia	Liver cancer due to hepatitis B	77(57 to 102)	295(202 to 424)	2.83(2.04 to 3.81)	0.34(0.25 to 0.44)	0.68(0.47 to 0.97)	1.02(0.61 to 1.54)
Australasia	Liver cancer due to hepatitis C	134(102 to 170)	689(489 to 947)	4.14(3.16 to 5.34)	0.57(0.44 to 0.72)	1.39(0.99 to 1.91)	1.43(0.96 to 1.98)
Australasia	Liver cancer due to NASH	36(26 to 48)	231(157 to 330)	5.46(4.09 to 7.23)	0.15(0.11 to 0.2)	0.47(0.32 to 0.67)	2.11(1.47 to 2.96)
Australasia	Liver cancer due to other causes	27(21 to 33)	100(70 to 142)	2.77(1.98 to 3.7)	0.13(0.1 to 0.16)	0.25(0.18 to 0.34)	0.96(0.57 to 1.45)
Caribbean	Liver cancer	1563(1452 to 1657)	1628(1353 to 1938)	0.04(-0.12 to 0.24)	5.94(5.52 to 6.3)	3.16(2.63 to 3.77)	-0.47(-0.55 to -0.36)
Caribbean	Liver cancer due to alcohol use	507(374 to 651)	569(406 to 769)	0.12(-0.08 to 0.35)	1.94(1.43 to 2.49)	1.1(0.79 to 1.49)	-0.43(-0.53 to -0.31)
Caribbean	Liver cancer due to hepatitis B	454(340 to 594)	445(310 to 618)	-0.02(-0.2 to 0.2)	1.68(1.25 to 2.22)	0.86(0.6 to 1.2)	-0.49(-0.58 to -0.37)
Caribbean	Liver cancer due to hepatitis C	355(249 to 474)	352(239 to 498)	-0.01(-0.16 to 0.16)	1.4(0.99 to 1.86)	0.68(0.46 to 0.96)	-0.51(-0.59 to -0.43)
Caribbean	Liver cancer due to NASH	129(91 to 177)	154(105 to 219)	0.2(0 to 0.43)	0.5(0.35 to 0.68)	0.3(0.2 to 0.42)	-0.4(-0.5 to -0.28)

Caribbean	Liver cancer due to other causes	117(88 to 153)	108(77 to 150)	-0.08(-0.24 to 0.09)	0.41(0.3 to 0.55)	0.22(0.16 to 0.3)	-0.47(-0.56 to -0.36)
Central Asia	Liver cancer	1493(1329 to 1658)	6109(5296 to 7001)	3.09(2.45 to 3.83)	3.13(2.79 to 3.47)	8.27(7.22 to 9.41)	1.64(1.25 to 2.1)
Central Asia	Liver cancer due to alcohol use	394(274 to 520)	1834(1288 to 2494)	3.66(2.88 to 4.53)	0.85(0.59 to 1.12)	2.47(1.77 to 3.26)	1.92(1.44 to 2.45)
Central Asia	Liver cancer due to hepatitis B	443(326 to 605)	1696(1206 to 2273)	2.83(2.14 to 3.59)	0.9(0.65 to 1.22)	2.05(1.47 to 2.78)	1.28(0.89 to 1.74)
Central Asia	Liver cancer due to hepatitis C	466(330 to 607)	1894(1332 to 2494)	3.06(2.46 to 3.78)	1.04(0.74 to 1.34)	2.81(2.04 to 3.63)	1.7(1.28 to 2.18)
Central Asia	Liver cancer due to NASH	78(54 to 111)	420(294 to 594)	4.4(3.55 to 5.36)	0.17(0.12 to 0.25)	0.61(0.43 to 0.87)	2.56(2 to 3.19)
Central Asia	Liver cancer due to other causes	112(79 to 159)	265(187 to 370)	1.37(0.61 to 2.43)	0.17(0.12 to 0.24)	0.33(0.24 to 0.46)	0.92(0.38 to 1.61)
Central Europe	Liver cancer	7699(7443 to 7902)	6906(5994 to 7986)	-0.1(-0.22 to 0.03)	5.25(5.06 to 5.39)	3.29(2.85 to 3.81)	-0.37(-0.46 to -0.28)
Central Europe	Liver cancer due to alcohol use	3012(2494 to 3504)	2985(2315 to 3704)	-0.01(-0.15 to 0.15)	2.02(1.67 to 2.34)	1.4(1.09 to 1.74)	-0.31(-0.4 to -0.2)
Central Europe	Liver cancer due to hepatitis B	1845(1446 to 2314)	1372(983 to 1908)	-0.26(-0.37 to -0.12)	1.25(0.99 to 1.57)	0.7(0.51 to 0.96)	-0.44(-0.52 to -0.34)
Central Europe	Liver cancer due to hepatitis C	2008(1586 to 2481)	1777(1296 to 2390)	-0.12(-0.24 to 0.03)	1.4(1.12 to 1.71)	0.81(0.59 to 1.08)	-0.42(-0.51 to -0.33)
Central Europe	Liver cancer due to NASH	547(428 to 695)	555(397 to 767)	0.01(-0.14 to 0.18)	0.38(0.3 to 0.48)	0.26(0.19 to 0.35)	-0.33(-0.42 to -0.21)
Central Europe	Liver cancer due to other causes	288(227 to 356)	217(153 to 298)	-0.25(-0.36 to -0.12)	0.21(0.17 to 0.26)	0.13(0.09 to 0.17)	-0.39(-0.48 to -0.29)
Central Latin America	Liver cancer	2970(2764 to 3123)	7987(6880 to 9272)	1.69(1.35 to 2.11)	3.49(3.24 to 3.68)	3.43(2.96 to 3.97)	-0.02(-0.14 to 0.13)
Central Latin America	Liver cancer due to alcohol use	827(641 to 1017)	2470(1914 to 3112)	1.99(1.59 to 2.5)	1(0.77 to 1.23)	1.06(0.82 to 1.33)	0.06(-0.08 to 0.23)

Central Latin America	Liver cancer due to hepatitis B	551(423 to 711)	1165(864 to 1608)	1.12(0.81 to 1.52)	0.58(0.43 to 0.77)	0.48(0.36 to 0.67)	-0.17(-0.29 to -0.01)
Central Latin America	Liver cancer due to hepatitis C	1102(900 to 1309)	3097(2487 to 3786)	1.81(1.46 to 2.25)	1.41(1.16 to 1.68)	1.35(1.08 to 1.65)	-0.04(-0.16 to 0.1)
Central Latin America	Liver cancer due to NASH	209(161 to 270)	734(567 to 961)	2.51(2.05 to 3.06)	0.25(0.19 to 0.33)	0.32(0.24 to 0.41)	0.26(0.1 to 0.45)
Central Latin America	Liver cancer due to other causes	281(236 to 332)	520(405 to 648)	0.85(0.54 to 1.19)	0.24(0.19 to 0.29)	0.22(0.17 to 0.27)	-0.08(-0.21 to 0.08)
Central Sub-Saharan Africa	Liver cancer	687(563 to 827)	1364(1080 to 1715)	0.99(0.49 to 1.65)	2.63(2.2 to 3.08)	2.3(1.84 to 2.86)	-0.13(-0.32 to 0.15)
Central Sub-Saharan Africa	Liver cancer due to alcohol use	82(53 to 121)	180(114 to 266)	1.19(0.63 to 1.98)	0.36(0.24 to 0.51)	0.33(0.21 to 0.48)	-0.08(-0.29 to 0.23)
Central Sub-Saharan Africa	Liver cancer due to hepatitis B	188(134 to 256)	375(258 to 542)	0.99(0.45 to 1.75)	0.66(0.46 to 0.91)	0.53(0.36 to 0.78)	-0.19(-0.39 to 0.09)
Central Sub-Saharan Africa	Liver cancer due to hepatitis C	261(189 to 339)	542(378 to 718)	1.07(0.55 to 1.76)	1.27(0.97 to 1.6)	1.12(0.82 to 1.47)	-0.12(-0.32 to 0.14)
Central Sub-Saharan Africa	Liver cancer due to NASH	37(25 to 53)	90(60 to 133)	1.43(0.78 to 2.31)	0.16(0.11 to 0.23)	0.16(0.11 to 0.24)	0.02(-0.22 to 0.35)
Central Sub-Saharan Africa	Liver cancer due to other causes	118(71 to 190)	177(107 to 293)	0.51(-0.14 to 1.46)	0.18(0.13 to 0.25)	0.15(0.1 to 0.22)	-0.18(-0.45 to 0.15)
East Asia	Liver cancer	241531(204188 to 284759)	217171(181403 to 257464)	-0.1(-0.3 to 0.16)	25.26(21.46 to 29.74)	10.43(8.76 to 12.3)	-0.59(-0.68 to -0.47)
East Asia	Liver cancer due to alcohol use	17943(13016 to 23872)	19766(14715 to 26282)	0.1(-0.13 to 0.41)	1.92(1.42 to 2.55)	0.92(0.69 to 1.21)	-0.52(-0.62 to -0.39)
East Asia	Liver cancer due to hepatitis B	161255(132170 to 194343)	138506(112428 to 167396)	-0.14(-0.35 to 0.14)	16.16(13.32 to 19.41)	6.57(5.35 to 7.95)	-0.59(-0.69 to -0.46)

East Asia	Liver cancer due to hepatitis C	36425(29958 to 44391)	35912(29625 to 42897)	-0.01(-0.22 to 0.24)	4.48(3.72 to 5.41)	1.79(1.48 to 2.12)	-0.6(-0.68 to -0.5)
East Asia	Liver cancer due to NASH	9022(7065 to 11091)	10459(8179 to 12962)	0.16(-0.08 to 0.45)	1(0.79 to 1.22)	0.51(0.4 to 0.63)	-0.49(-0.59 to -0.37)
East Asia	Liver cancer due to other causes	16886(13814 to 20858)	12528(10141 to 15478)	-0.26(-0.41 to -0.07)	1.69(1.38 to 2.09)	0.64(0.52 to 0.78)	-0.62(-0.7 to -0.53)
Eastern Europe	Liver cancer	4139(3971 to 4327)	9407(8199 to 10735)	1.27(1.02 to 1.56)	1.52(1.45 to 1.59)	2.84(2.46 to 3.24)	0.87(0.66 to 1.1)
Eastern Europe	Liver cancer due to alcohol use	1374(1188 to 1565)	3555(2922 to 4240)	1.59(1.26 to 1.96)	0.48(0.42 to 0.55)	1.04(0.86 to 1.24)	1.15(0.88 to 1.46)
Eastern Europe	Liver cancer due to hepatitis B	1095(933 to 1294)	2162(1716 to 2694)	0.97(0.72 to 1.24)	0.4(0.34 to 0.46)	0.68(0.55 to 0.85)	0.72(0.51 to 0.96)
Eastern Europe	Liver cancer due to hepatitis C	1076(923 to 1235)	2509(2067 to 3006)	1.33(1.1 to 1.59)	0.39(0.34 to 0.44)	0.71(0.59 to 0.85)	0.83(0.65 to 1.04)
Eastern Europe	Liver cancer due to NASH	285(242 to 339)	768(629 to 925)	1.69(1.4 to 2.01)	0.1(0.09 to 0.12)	0.23(0.19 to 0.27)	1.17(0.94 to 1.43)
Eastern Europe	Liver cancer due to other causes	308(261 to 376)	413(315 to 530)	0.34(0.05 to 0.65)	0.14(0.12 to 0.18)	0.17(0.12 to 0.25)	0.22(-0.12 to 0.62)
Eastern Sub-Saharan Africa	Liver cancer	2444(1992 to 3027)	5439(4462 to 6714)	1.23(0.72 to 1.82)	2.93(2.44 to 3.7)	3.13(2.6 to 3.81)	0.07(-0.13 to 0.31)
Eastern Sub-Saharan Africa	Liver cancer due to alcohol use	565(404 to 806)	1317(927 to 1848)	1.33(0.88 to 1.89)	0.76(0.55 to 1.09)	0.83(0.58 to 1.14)	0.09(-0.1 to 0.33)
Eastern Sub-Saharan Africa	Liver cancer due to hepatitis B	733(548 to 998)	1646(1203 to 2198)	1.25(0.76 to 1.88)	0.83(0.61 to 1.15)	0.83(0.61 to 1.13)	0(-0.19 to 0.26)
Eastern Sub-Saharan Africa	Liver cancer due to hepatitis C	515(362 to 686)	1166(864 to 1504)	1.26(0.78 to 1.87)	0.77(0.56 to 1.02)	0.83(0.63 to 1.06)	0.08(-0.12 to 0.33)
Eastern Sub-Saharan Africa	Liver cancer due to NASH	234(171 to 317)	601(438 to 807)	1.57(1.05 to 2.22)	0.32(0.24 to 0.44)	0.38(0.28 to 0.52)	0.19(-0.01 to 0.46)

Eastern Sub-Saharan Africa	Liver cancer due to other causes	396(274 to 567)	709(521 to 941)	0.79(0.15 to 1.55)	0.25(0.18 to 0.34)	0.25(0.19 to 0.32)	-0.01(-0.27 to 0.29)
High-income Asia Pacific	Liver cancer	28212(27268 to 29085)	67946(58134 to 77642)	1.41(1.08 to 1.75)	13.77(13.3 to 14.19)	15.56(13.46 to 17.74)	0.13(-0.02 to 0.28)
High-income Asia Pacific	Liver cancer due to alcohol use	3661(3043 to 4341)	8717(6689 to 11065)	1.38(0.99 to 1.81)	1.76(1.47 to 2.08)	2.07(1.58 to 2.67)	0.18(-0.02 to 0.39)
High-income Asia Pacific	Liver cancer due to hepatitis B	6234(5470 to 7074)	16844(13559 to 20453)	1.7(1.22 to 2.24)	3(2.63 to 3.4)	4.73(3.84 to 5.69)	0.58(0.3 to 0.89)
High-income Asia Pacific	Liver cancer due to hepatitis C	16300(15267 to 17273)	37118(30231 to 43419)	1.28(0.9 to 1.63)	8(7.5 to 8.47)	7.54(6.29 to 8.91)	-0.06(-0.2 to 0.09)
High-income Asia Pacific	Liver cancer due to NASH	1129(945 to 1334)	3357(2562 to 4359)	1.97(1.46 to 2.54)	0.56(0.47 to 0.66)	0.71(0.55 to 0.92)	0.28(0.09 to 0.5)
High-income Asia Pacific	Liver cancer due to other causes	890(749 to 1042)	1910(1456 to 2402)	1.15(0.77 to 1.53)	0.45(0.38 to 0.52)	0.5(0.39 to 0.62)	0.11(-0.04 to 0.28)
High-income North America	Liver cancer	7533(7276 to 7697)	31008(25713 to 36961)	3.12(2.41 to 3.9)	2.2(2.14 to 2.25)	5.18(4.28 to 6.18)	1.35(0.94 to 1.79)
High-income North America	Liver cancer due to alcohol use	2393(2121 to 2655)	10672(8401 to 13357)	3.46(2.58 to 4.46)	0.69(0.62 to 0.77)	1.77(1.39 to 2.22)	1.55(1.04 to 2.13)
High-income North America	Liver cancer due to hepatitis B	1021(881 to 1178)	3658(2790 to 4737)	2.58(1.89 to 3.36)	0.31(0.27 to 0.36)	0.66(0.5 to 0.85)	1.12(0.72 to 1.58)
High-income North America	Liver cancer due to hepatitis C	2601(2332 to 2850)	10988(8872 to 13360)	3.22(2.53 to 3.98)	0.74(0.67 to 0.82)	1.77(1.43 to 2.15)	1.37(0.98 to 1.79)
High-income North America	Liver cancer due to NASH	728(622 to 851)	3185(2499 to 3943)	3.37(2.68 to 4.11)	0.2(0.18 to 0.24)	0.52(0.41 to 0.64)	1.52(1.12 to 1.95)

High-income North America	Liver cancer due to other causes	790(695 to 881)	2506(2000 to 3096)	2.17(1.66 to 2.73)	0.25(0.22 to 0.28)	0.47(0.38 to 0.57)	0.86(0.57 to 1.17)
North Africa and Middle East	Liver cancer	10734(9445 to 11997)	27546(22113 to 33841)	1.57(0.99 to 2.43)	6.08(5.28 to 6.81)	6.29(5.13 to 7.71)	0.04(-0.19 to 0.38)
North Africa and Middle East	Liver cancer due to alcohol use	943(623 to 1343)	2617(1683 to 4007)	1.77(1.12 to 2.71)	0.55(0.37 to 0.78)	0.61(0.4 to 0.91)	0.1(-0.14 to 0.47)
North Africa and Middle East	Liver cancer due to hepatitis B	3203(2537 to 3974)	7259(5421 to 9466)	1.27(0.82 to 1.83)	1.69(1.32 to 2.13)	1.53(1.14 to 2.01)	-0.09(-0.26 to 0.14)
North Africa and Middle East	Liver cancer due to hepatitis C	4934(3888 to 6011)	12952(9358 to 17369)	1.63(0.91 to 2.7)	2.98(2.37 to 3.63)	3.08(2.28 to 4.07)	0.03(-0.24 to 0.44)
North Africa and Middle East	Liver cancer due to NASH	812(587 to 1143)	2872(2003 to 4128)	2.54(1.69 to 3.85)	0.48(0.35 to 0.68)	0.68(0.48 to 0.97)	0.41(0.08 to 0.92)
North Africa and Middle East	Liver cancer due to other causes	843(644 to 1080)	1846(1318 to 2574)	1.19(0.7 to 1.87)	0.37(0.28 to 0.48)	0.38(0.27 to 0.54)	0.04(-0.18 to 0.38)
Oceania	Liver cancer	113(95 to 132)	234(195 to 278)	1.06(0.68 to 1.55)	3.65(3.08 to 4.25)	3.28(2.77 to 3.89)	-0.1(-0.27 to 0.1)
Oceania	Liver cancer due to alcohol use	13(8 to 19)	29(19 to 44)	1.29(0.8 to 1.86)	0.44(0.28 to 0.65)	0.44(0.28 to 0.64)	-0.01(-0.21 to 0.24)
Oceania	Liver cancer due to hepatitis B	64(50 to 79)	126(97 to 162)	0.97(0.58 to 1.42)	1.88(1.45 to 2.35)	1.59(1.22 to 2.04)	-0.15(-0.32 to 0.04)
Oceania	Liver cancer due to hepatitis C	22(14 to 30)	46(31 to 66)	1.11(0.74 to 1.62)	0.88(0.6 to 1.19)	0.8(0.55 to 1.1)	-0.09(-0.26 to 0.12)
Oceania	Liver cancer due to NASH	8(5 to 12)	19(13 to 28)	1.45(1.01 to 2.03)	0.28(0.19 to 0.41)	0.3(0.21 to 0.43)	0.07(-0.12 to 0.31)
Oceania	Liver cancer due to other causes	7(5 to 9)	14(10 to 18)	0.95(0.6 to 1.39)	0.17(0.12 to 0.23)	0.15(0.1 to 0.21)	-0.12(-0.27 to 0.08)

South Asia	Liver cancer	15679(13213 to 18045)	37733(32783 to 43281)	1.41(0.98 to 1.88)	2.66(2.2 to 3.08)	2.66(2.3 to 3.05)	0(-0.17 to 0.2)
South Asia	Liver cancer due to alcohol use	3511(2731 to 4391)	9738(7744 to 11997)	1.77(1.23 to 2.44)	0.63(0.48 to 0.78)	0.69(0.55 to 0.85)	0.1(-0.11 to 0.37)
South Asia	Liver cancer due to hepatitis B	5981(4870 to 7122)	12558(10250 to 15244)	1.1(0.69 to 1.54)	0.92(0.73 to 1.1)	0.84(0.68 to 1.02)	-0.09(-0.27 to 0.11)
South Asia	Liver cancer due to hepatitis C	3554(2753 to 4410)	9249(7525 to 11101)	1.6(1.12 to 2.14)	0.73(0.56 to 0.9)	0.7(0.58 to 0.84)	-0.03(-0.22 to 0.18)
South Asia	Liver cancer due to NASH	1383(1110 to 1688)	3970(3227 to 4908)	1.87(1.36 to 2.43)	0.25(0.2 to 0.31)	0.29(0.24 to 0.36)	0.15(-0.07 to 0.37)
South Asia	Liver cancer due to other causes	1249(1041 to 1520)	2217(1848 to 2750)	0.77(0.5 to 1.1)	0.13(0.11 to 0.16)	0.14(0.12 to 0.17)	0.03(-0.12 to 0.21)
Southeast Asia	Liver cancer	17307(15374 to 18982)	42800(35218 to 52129)	1.47(0.99 to 2.1)	6.43(5.69 to 7.06)	7.07(5.87 to 8.61)	0.1(-0.11 to 0.38)
Southeast Asia	Liver cancer due to alcohol use	3550(2610 to 4678)	11132(7772 to 15451)	2.14(1.41 to 3.05)	1.37(1.02 to 1.81)	1.84(1.3 to 2.54)	0.34(0.04 to 0.72)
Southeast Asia	Liver cancer due to hepatitis B	7993(6570 to 9557)	17138(12887 to 22480)	1.14(0.67 to 1.75)	2.73(2.19 to 3.29)	2.64(1.97 to 3.45)	-0.03(-0.25 to 0.23)
Southeast Asia	Liver cancer due to hepatitis C	3689(2799 to 4647)	9145(6792 to 12022)	1.48(1.03 to 2.04)	1.6(1.24 to 1.99)	1.67(1.25 to 2.17)	0.04(-0.15 to 0.27)
Southeast Asia	Liver cancer due to NASH	1211(914 to 1598)	3973(2823 to 5485)	2.28(1.6 to 3.17)	0.49(0.36 to 0.64)	0.7(0.49 to 0.97)	0.44(0.14 to 0.83)
Southeast Asia	Liver cancer due to other causes	864(658 to 1132)	1412(1046 to 1910)	0.63(0.19 to 1.15)	0.24(0.18 to 0.3)	0.23(0.17 to 0.31)	-0.04(-0.25 to 0.2)
Southern Latin America	Liver cancer	721(652 to 791)	1939(1524 to 2424)	1.69(1.1 to 2.43)	1.56(1.41 to 1.71)	2.33(1.83 to 2.92)	0.49(0.17 to 0.9)
Southern Latin America	Liver cancer due to alcohol use	239(170 to 312)	629(418 to 874)	1.63(1.02 to 2.45)	0.51(0.37 to 0.66)	0.75(0.5 to 1.04)	0.47(0.13 to 0.93)

Southern Latin America	Liver cancer due to hepatitis B	137(96 to 190)	327(214 to 493)	1.39(0.83 to 2.13)	0.29(0.21 to 0.41)	0.4(0.26 to 0.61)	0.38(0.06 to 0.82)
Southern Latin America	Liver cancer due to hepatitis C	237(173 to 312)	681(459 to 934)	1.87(1.25 to 2.65)	0.52(0.39 to 0.68)	0.8(0.55 to 1.1)	0.53(0.2 to 0.94)
Southern Latin America	Liver cancer due to NASH	53(36 to 78)	186(123 to 272)	2.49(1.72 to 3.51)	0.12(0.08 to 0.17)	0.22(0.15 to 0.32)	0.88(0.47 to 1.42)
Southern Latin America	Liver cancer due to other causes	55(42 to 71)	116(78 to 167)	1.13(0.64 to 1.72)	0.11(0.09 to 0.15)	0.15(0.1 to 0.21)	0.34(0.05 to 0.69)
Southern Sub-Saharan Africa	Liver cancer	1908(1345 to 3153)	4016(3581 to 4521)	1.1(0.19 to 2.18)	6.47(4.52 to 10.72)	6.77(6.07 to 7.61)	0.05(-0.4 to 0.59)
Southern Sub-Saharan Africa	Liver cancer due to alcohol use	358(215 to 689)	782(622 to 985)	1.19(0.12 to 2.55)	1.26(0.75 to 2.41)	1.34(1.07 to 1.67)	0.07(-0.45 to 0.74)
Southern Sub-Saharan Africa	Liver cancer due to hepatitis B	776(518 to 1351)	1559(1297 to 1866)	1.01(0.07 to 2.14)	2.39(1.56 to 4.14)	2.39(1.98 to 2.85)	0(-0.46 to 0.56)
Southern Sub-Saharan Africa	Liver cancer due to hepatitis C	480(330 to 720)	1036(839 to 1246)	1.16(0.38 to 2.12)	1.86(1.27 to 2.79)	1.96(1.62 to 2.35)	0.05(-0.33 to 0.53)
Southern Sub-Saharan Africa	Liver cancer due to NASH	173(116 to 281)	421(342 to 511)	1.43(0.45 to 2.62)	0.61(0.4 to 1)	0.75(0.61 to 0.92)	0.22(-0.27 to 0.82)
Southern Sub-Saharan Africa	Liver cancer due to other causes	120(87 to 185)	218(175 to 272)	0.81(0.15 to 1.55)	0.34(0.24 to 0.53)	0.34(0.27 to 0.42)	-0.01(-0.37 to 0.41)
Tropical Latin America	Liver cancer	1846(1774 to 1903)	5667(5335 to 5956)	2.07(1.9 to 2.27)	1.97(1.88 to 2.04)	2.36(2.22 to 2.48)	0.2(0.13 to 0.27)
Tropical Latin America	Liver cancer due to alcohol use	502(433 to 573)	1750(1509 to 1985)	2.49(2.27 to 2.74)	0.54(0.47 to 0.62)	0.72(0.62 to 0.82)	0.32(0.24 to 0.42)
Tropical Latin America	Liver cancer due to hepatitis B	423(366 to 484)	1029(868 to 1212)	1.43(1.27 to 1.62)	0.41(0.35 to 0.47)	0.42(0.35 to 0.49)	0.02(-0.04 to 0.1)

Tropical Latin America	Liver cancer due to hepatitis C	658(585 to 733)	2218(1939 to 2486)	2.37(2.18 to 2.59)	0.78(0.69 to 0.87)	0.94(0.82 to 1.05)	0.2(0.14 to 0.29)
Tropical Latin America	Liver cancer due to NASH	100(86 to 117)	358(304 to 421)	2.57(2.34 to 2.81)	0.11(0.09 to 0.13)	0.15(0.13 to 0.18)	0.36(0.28 to 0.44)
Tropical Latin America	Liver cancer due to other causes	163(145 to 185)	312(269 to 358)	0.91(0.72 to 1.09)	0.13(0.12 to 0.15)	0.14(0.12 to 0.16)	0.04(-0.04 to 0.13)
Western Europe	Liver cancer	20184(19491 to 20702)	45859(39837 to 52739)	1.27(0.98 to 1.61)	3.55(3.43 to 3.64)	5.31(4.59 to 6.12)	0.5(0.3 to 0.73)
Western Europe	Liver cancer due to alcohol use	7128(5804 to 8386)	16556(12857 to 20520)	1.32(0.98 to 1.71)	1.25(1.02 to 1.47)	1.94(1.5 to 2.4)	0.55(0.33 to 0.82)
Western Europe	Liver cancer due to hepatitis B	2642(2021 to 3413)	5341(3865 to 7410)	1.02(0.73 to 1.39)	0.5(0.38 to 0.63)	0.71(0.52 to 0.97)	0.43(0.22 to 0.7)
Western Europe	Liver cancer due to hepatitis C	8401(7130 to 9778)	18947(15193 to 23282)	1.26(0.98 to 1.59)	1.43(1.22 to 1.67)	2.05(1.63 to 2.55)	0.43(0.25 to 0.65)
Western Europe	Liver cancer due to NASH	1096(836 to 1446)	3008(2184 to 4144)	1.75(1.36 to 2.18)	0.19(0.14 to 0.24)	0.33(0.24 to 0.45)	0.77(0.53 to 1.05)
Western Europe	Liver cancer due to other causes	917(720 to 1160)	2007(1462 to 2689)	1.19(0.89 to 1.54)	0.19(0.15 to 0.23)	0.28(0.22 to 0.37)	0.5(0.3 to 0.73)
Western Sub-Saharan Africa	Liver cancer	5114(4328 to 5960)	9709(8164 to 11417)	0.9(0.52 to 1.31)	5.45(4.6 to 6.32)	4.93(4.19 to 5.72)	-0.09(-0.28 to 0.11)
Western Sub-Saharan Africa	Liver cancer due to alcohol use	840(589 to 1133)	1769(1275 to 2345)	1.11(0.65 to 1.61)	0.96(0.69 to 1.3)	0.98(0.71 to 1.29)	0.02(-0.21 to 0.26)
Western Sub-Saharan Africa	Liver cancer due to hepatitis B	2489(1979 to 3047)	4614(3604 to 5785)	0.85(0.45 to 1.3)	2.57(2.03 to 3.15)	2.17(1.67 to 2.72)	-0.15(-0.34 to 0.04)
Western Sub-Saharan Africa	Liver cancer due to hepatitis C	963(675 to 1266)	1774(1268 to 2303)	0.84(0.5 to 1.24)	1.2(0.86 to 1.56)	1.07(0.78 to 1.37)	-0.11(-0.27 to 0.07)
Western Sub-Saharan	Liver cancer due to NASH	351(260 to 468)	800(594 to 1070)	1.28(0.84 to 1.82)	0.41(0.3 to 0.55)	0.45(0.33 to 0.61)	0.09(-0.12 to 0.33)

Africa							
Western Sub-Saharan Africa	Liver cancer due to other causes	471(364 to 599)	753(559 to 977)	0.6(0.24 to 1.03)	0.3(0.23 to 0.38)	0.26(0.2 to 0.34)	-0.13(-0.29 to 0.05)

Table S3. The disability-adjusted life years (DALYs) of liver cancer and underlying etiologies between 1990 and 2019 at global, regional level, both sexes

**regions**	**Causes**	**Cases in 1990**	**Cases in 2019**	**Change in absolute number (95% UI)**	**ASRs in 1990**	**ASRs in 2019**	**change in ASR per 100 000 population (95% UI)**
Global	Liver cancer	11278630(10062526 to 12677403)	12528422(11400671 to 13687675)	0.11(-0.05 to 0.31)	258.37(230.9 to 290.13)	151.08(137.53 to 164.82)	-0.42(-0.5 to -0.32)
Global	Liver cancer due to alcohol use	1274808(1016105 to 1563126)	2181197(1756692 to 2653361)	0.71(0.54 to 0.91)	30.59(24.49 to 37.59)	26.07(21.07 to 31.63)	-0.15(-0.23 to -0.05)
Global	Liver cancer due to hepatitis B	6480909(5536716 to 7595880)	5797099(4911756 to 6771065)	-0.11(-0.27 to 0.11)	145.84(124.6 to 171.24)	69.15(58.71 to 80.61)	-0.53(-0.61 to -0.41)
Global	Liver cancer due to hepatitis C	2003448(1731331 to 2320933)	2878024(2439911 to 3323494)	0.44(0.29 to 0.62)	49.7(42.99 to 57.44)	34.99(29.71 to 40.28)	-0.3(-0.37 to -0.2)
Global	Liver cancer due to NASH	478939(392589 to 577854)	795806(657291 to 975790)	0.66(0.44 to 0.91)	11.35(9.33 to 13.71)	9.64(7.98 to 11.75)	-0.15(-0.26 to -0.03)
Global	Liver cancer due to other causes	1040525(884154 to 1237284)	876295(748509 to 1015967)	-0.16(-0.31 to 0)	20.89(17.67 to 24.92)	11.24(9.61 to 13.02)	-0.46(-0.55 to -0.36)
Andean Latin America	Liver cancer	30637(27050 to 34452)	44340(35812 to 54428)	0.45(0.14 to 0.83)	129.83(113.96 to 146.3)	77.27(62.62 to 94.73)	-0.4(-0.53 to -0.25)

Andean Latin America	Liver cancer due to alcohol use	7513(5217 to 10033)	12772(8626 to 17767)	0.7(0.31 to 1.21)	36.05(25.15 to 48.32)	22.81(15.43 to 31.67)	-0.37(-0.51 to -0.18)
Andean Latin America	Liver cancer due to hepatitis B	15209(12469 to 18358)	20097(14816 to 26072)	0.32(0.02 to 0.7)	63.94(51.38 to 78.43)	34.48(25.2 to 44.9)	-0.46(-0.59 to -0.31)
Andean Latin America	Liver cancer due to hepatitis C	1556(958 to 2355)	2735(1649 to 4224)	0.76(0.38 to 1.25)	7.85(4.78 to 11.82)	4.98(3 to 7.68)	-0.37(-0.5 to -0.2)
Andean Latin America	Liver cancer due to NASH	1939(1383 to 2692)	3903(2692 to 5532)	1.01(0.55 to 1.59)	9.06(6.28 to 12.75)	6.98(4.8 to 9.87)	-0.23(-0.41 to -0.01)
Andean Latin America	Liver cancer due to other causes	4421(3529 to 5514)	4834(3565 to 6371)	0.09(-0.19 to 0.48)	12.94(9.96 to 16.54)	8.03(5.91 to 10.61)	-0.38(-0.53 to -0.19)
Australasia	Liver cancer	11484(11085 to 11938)	43655(40249 to 47404)	2.8(2.47 to 3.13)	50.35(48.58 to 52.27)	98.07(90.33 to 106.39)	0.95(0.78 to 1.12)
Australasia	Liver cancer due to alcohol use	4842(3885 to 5762)	17277(13218 to 21069)	2.57(2.18 to 2.97)	20.88(16.84 to 24.91)	38.34(29.3 to 46.83)	0.84(0.64 to 1.04)
Australasia	Liver cancer due to hepatitis B	2109(1584 to 2737)	6675(4863 to 9174)	2.17(1.78 to 2.54)	9.4(7.11 to 12.2)	16.29(12.09 to 22.02)	0.73(0.53 to 0.94)
Australasia	Liver cancer due to hepatitis C	2937(2206 to 3777)	13000(9662 to 16800)	3.43(3.03 to 3.87)	12.62(9.48 to 16.24)	27.49(20.36 to 35.58)	1.18(0.99 to 1.4)
Australasia	Liver cancer due to NASH	805(598 to 1075)	4400(3218 to 5852)	4.46(3.65 to 5.28)	3.47(2.59 to 4.58)	9.56(7.1 to 12.59)	1.76(1.36 to 2.16)
Australasia	Liver cancer due to other causes	791(666 to 958)	2303(1756 to 2989)	1.91(1.47 to 2.33)	3.98(3.4 to 4.7)	6.39(5.13 to 7.99)	0.61(0.41 to 0.8)
Caribbean	Liver cancer	41326(38233 to 44105)	41276(33562 to 50616)	0(-0.17 to 0.2)	151.48(140.38 to 161.07)	80.66(65.64 to 99.23)	-0.47(-0.56 to -0.35)
Caribbean	Liver cancer due to alcohol use	12568(9233 to 16135)	13740(9786 to 18707)	0.09(-0.1 to 0.33)	47.44(34.92 to 61.04)	26.53(18.91 to 36.1)	-0.44(-0.54 to -0.32)
Caribbean	Liver cancer due to hepatitis B	13418(10259 to 16982)	12735(8872 to 17486)	-0.05(-0.23 to 0.17)	48.27(36.72 to 62.09)	24.82(17.32 to 34.1)	-0.49(-0.58 to -0.35)

Caribbean	Liver cancer due to hepatitis C	7948(5458 to 10745)	7613(4943 to 11100)	-0.04(-0.2 to 0.14)	30.67(21.14 to 41.41)	14.7(9.58 to 21.4)	-0.52(-0.6 to -0.43)
Caribbean	Liver cancer due to NASH	3084(2235 to 4179)	3523(2423 to 4983)	0.14(-0.06 to 0.37)	11.49(8.19 to 15.61)	6.84(4.71 to 9.68)	-0.4(-0.51 to -0.28)
Caribbean	Liver cancer due to other causes	4308(3317 to 5718)	3666(2655 to 4985)	-0.15(-0.31 to 0.02)	13.61(10.37 to 17.88)	7.76(5.66 to 10.48)	-0.43(-0.54 to -0.31)
Central Asia	Liver cancer	43272(38393 to 48032)	172830(148859 to 200042)	2.99(2.35 to 3.78)	85.32(75.7 to 94.97)	213.45(184.93 to 244.51)	1.5(1.11 to 1.96)
Central Asia	Liver cancer due to alcohol use	10701(7288 to 14241)	50544(35195 to 69123)	3.72(2.93 to 4.62)	22.28(15.44 to 29.34)	62.98(44.48 to 84.41)	1.83(1.34 to 2.35)
Central Asia	Liver cancer due to hepatitis B	13358(9941 to 18092)	53555(38489 to 71466)	3.01(2.26 to 3.85)	26.36(19.6 to 35.55)	60.59(43.66 to 80.9)	1.3(0.9 to 1.78)
Central Asia	Liver cancer due to hepatitis C	11664(8059 to 15413)	47951(32811 to 64423)	3.11(2.47 to 3.84)	24.99(17.55 to 32.65)	64.25(45.62 to 83.96)	1.57(1.17 to 2.05)
Central Asia	Liver cancer due to NASH	2025(1398 to 2920)	11173(7786 to 15816)	4.52(3.63 to 5.52)	4.26(2.91 to 6.07)	14.63(10.39 to 20.48)	2.44(1.88 to 3.05)
Central Asia	Liver cancer due to other causes	5525(4094 to 6827)	9606(7042 to 13036)	0.74(0.18 to 1.7)	7.43(5.54 to 9.29)	11(8.11 to 14.88)	0.48(0.05 to 1.17)
Central Europe	Liver cancer	194212(188478 to 199058)	156614(133681 to 182107)	-0.19(-0.31 to -0.06)	131.74(127.71 to 135.03)	79.13(67.67 to 92.27)	-0.4(-0.49 to -0.3)
Central Europe	Liver cancer due to alcohol use	75210(61708 to 87797)	68127(52706 to 85040)	-0.09(-0.22 to 0.06)	50.01(41.33 to 58.29)	33.33(25.65 to 41.65)	-0.33(-0.43 to -0.22)
Central Europe	Liver cancer due to hepatitis B	52117(41318 to 65020)	34875(25572 to 47996)	-0.33(-0.43 to -0.21)	35.59(28.56 to 44.18)	19.05(14.11 to 25.94)	-0.46(-0.55 to -0.37)
Central Europe	Liver cancer due to hepatitis C	45286(34783 to 56454)	36480(25653 to 49748)	-0.19(-0.32 to -0.05)	30.6(23.91 to 37.83)	17.19(12.07 to 23.31)	-0.44(-0.53 to -0.34)
Central Europe	Liver cancer due to NASH	12691(9836 to 16186)	11614(8351 to 16062)	-0.08(-0.23 to 0.07)	8.67(6.82 to 10.98)	5.69(4.14 to 7.82)	-0.34(-0.44 to -0.23)

Central Europe	Liver cancer due to other causes	8907(7382 to 10715)	5517(4024 to 7456)	-0.38(-0.48 to -0.26)	6.87(5.83 to 8.05)	3.87(2.99 to 4.91)	-0.44(-0.52 to -0.34)
Central Latin America	Liver cancer	84956(79529 to 89037)	197475(171637 to 231238)	1.32(1.01 to 1.71)	89.22(83.1 to 93.77)	82.82(72.13 to 97)	-0.07(-0.19 to 0.08)
Central Latin America	Liver cancer due to alcohol use	21900(16814 to 27046)	60088(46433 to 76237)	1.74(1.35 to 2.21)	25.04(19.41 to 30.93)	25.19(19.55 to 31.92)	0.01(-0.14 to 0.18)
Central Latin America	Liver cancer due to hepatitis B	17688(13819 to 22377)	33051(24719 to 44936)	0.87(0.58 to 1.23)	17.19(13.26 to 22.13)	13.37(9.94 to 18.25)	-0.22(-0.34 to -0.07)
Central Latin America	Liver cancer due to hepatitis C	26636(21251 to 32163)	68790(54760 to 85626)	1.58(1.25 to 2.02)	32.12(25.84 to 38.66)	29.32(23.41 to 36.37)	-0.09(-0.2 to 0.06)
Central Latin America	Liver cancer due to NASH	5602(4381 to 7243)	17414(13626 to 22466)	2.11(1.67 to 2.6)	6.11(4.66 to 7.91)	7.31(5.72 to 9.42)	0.2(0.03 to 0.39)
Central Latin America	Liver cancer due to other causes	13130(11627 to 14796)	18132(14558 to 22586)	0.38(0.12 to 0.73)	8.76(7.48 to 10.17)	7.63(6.11 to 9.53)	-0.13(-0.27 to 0.05)
Central Sub-Saharan Africa	Liver cancer	28800(23045 to 36322)	51448(38555 to 67260)	0.79(0.24 to 1.44)	77.32(64.82 to 91.3)	65.34(51.74 to 82.14)	-0.15(-0.36 to 0.12)
Central Sub-Saharan Africa	Liver cancer due to alcohol use	2382(1512 to 3473)	5307(3288 to 7931)	1.23(0.65 to 2.03)	9.44(6.13 to 13.53)	8.76(5.55 to 12.82)	-0.07(-0.3 to 0.24)
Central Sub-Saharan Africa	Liver cancer due to hepatitis B	6843(5005 to 9079)	13951(9795 to 19601)	1.04(0.45 to 1.81)	20.73(14.95 to 28.2)	16.87(11.52 to 24.67)	-0.19(-0.4 to 0.11)
Central Sub-Saharan Africa	Liver cancer due to hepatitis C	7240(5222 to 9496)	14904(10198 to 20036)	1.06(0.54 to 1.77)	30.68(22.81 to 39.24)	26.75(18.78 to 35.32)	-0.13(-0.33 to 0.15)
Central Sub-Saharan Africa	Liver cancer due to NASH	1144(787 to 1622)	2792(1863 to 4163)	1.44(0.77 to 2.36)	4.18(2.89 to 5.96)	4.2(2.81 to 6.23)	0.01(-0.25 to 0.36)
Central Sub-Saharan Africa	Liver cancer due to other causes	11189(7032 to 17281)	14494(7638 to 24239)	0.3(-0.36 to 1.32)	12.29(8.57 to 17.93)	8.75(5.24 to 13.88)	-0.29(-0.6 to 0.15)

East Asia	Liver cancer	7723663(6580547 to 9129280)	5491479(4590535 to 6534290)	-0.29(-0.45 to -0.08)	755.38(644.72 to 893.3)	263.4(221.29 to 312.17)	-0.65(-0.73 to -0.55)
East Asia	Liver cancer due to alcohol use	520098(383773 to 701233)	465518(338175 to 605921)	-0.1(-0.31 to 0.16)	53.18(39.36 to 71.15)	21.28(15.68 to 27.47)	-0.6(-0.69 to -0.48)
East Asia	Liver cancer due to hepatitis B	5357704(4436313 to 6458092)	3694477(3004762 to 4530774)	-0.31(-0.48 to -0.08)	510.48(421.91 to 615.25)	176.37(143.94 to 215.63)	-0.65(-0.74 to -0.54)
East Asia	Liver cancer due to hepatitis C	934519(765542 to 1145647)	751414(609844 to 900127)	-0.2(-0.36 to 0.01)	104.78(86.75 to 127.13)	35.67(29.24 to 42.58)	-0.66(-0.73 to -0.58)
East Asia	Liver cancer due to NASH	262963(210092 to 327079)	238268(190065 to 295673)	-0.09(-0.28 to 0.14)	26.9(21.46 to 33.35)	11.38(9.19 to 14.04)	-0.58(-0.66 to -0.47)
East Asia	Liver cancer due to other causes	648380(535334 to 782314)	341802(276408 to 414280)	-0.47(-0.58 to -0.34)	60.04(49.45 to 72.64)	18.71(15.53 to 22.15)	-0.69(-0.75 to -0.61)
Eastern Europe	Liver cancer	115457(110529 to 121300)	234701(205032 to 273291)	1.03(0.79 to 1.32)	43.53(41.62 to 45.75)	74.86(65.32 to 86.64)	0.72(0.51 to 0.95)
Eastern Europe	Liver cancer due to alcohol use	36057(30819 to 41309)	88163(72048 to 107245)	1.45(1.13 to 1.83)	12.65(10.87 to 14.5)	26.55(21.61 to 32.58)	1.1(0.83 to 1.43)
Eastern Europe	Liver cancer due to hepatitis B	32537(27719 to 38161)	61326(48867 to 76782)	0.88(0.64 to 1.18)	11.94(10.22 to 13.88)	20.39(16.49 to 25.25)	0.71(0.48 to 0.97)
Eastern Europe	Liver cancer due to hepatitis C	25141(21212 to 29396)	54373(44568 to 65512)	1.16(0.93 to 1.42)	8.89(7.56 to 10.28)	15.78(13.01 to 18.96)	0.78(0.58 to 0.99)
Eastern Europe	Liver cancer due to NASH	7064(6005 to 8380)	17528(14513 to 21469)	1.48(1.2 to 1.79)	2.57(2.2 to 3.02)	5.4(4.49 to 6.51)	1.1(0.86 to 1.37)
Eastern Europe	Liver cancer due to other causes	14657(13199 to 16257)	13310(10139 to 17264)	-0.09(-0.28 to 0.14)	7.48(6.71 to 8.35)	6.74(4.85 to 9.52)	-0.1(-0.34 to 0.21)
Eastern Sub-Saharan Africa	Liver cancer	91016(71408 to 113690)	187944(149325 to 232670)	1.06(0.48 to 1.75)	82.98(68.12 to 103.03)	85.45(70.21 to 105.1)	0.03(-0.2 to 0.3)

Eastern Sub-Saharan Africa	Liver cancer due to alcohol use	15750(11197 to 22670)	36993(26188 to 51882)	1.35(0.86 to 1.97)	19.57(13.93 to 28.03)	21.06(15.01 to 29.5)	0.08(-0.14 to 0.34)
Eastern Sub-Saharan Africa	Liver cancer due to hepatitis B	24832(18662 to 33058)	56994(41663 to 75257)	1.3(0.76 to 2)	25.23(18.93 to 33.97)	25.29(18.47 to 33.97)	0(-0.21 to 0.28)
Eastern Sub-Saharan Africa	Liver cancer due to hepatitis C	13519(9455 to 18300)	29609(21757 to 38928)	1.19(0.67 to 1.82)	17.99(12.78 to 24.06)	18.7(13.85 to 24.23)	0.04(-0.19 to 0.31)
Eastern Sub-Saharan Africa	Liver cancer due to NASH	6721(4937 to 9013)	17271(12422 to 23262)	1.57(0.99 to 2.3)	7.96(5.89 to 10.8)	9.36(6.74 to 12.57)	0.18(-0.06 to 0.47)
Eastern Sub-Saharan Africa	Liver cancer due to other causes	30194(20663 to 44640)	47076(33911 to 62851)	0.56(-0.09 to 1.4)	12.23(8.75 to 16.9)	11.04(8.26 to 14.1)	-0.1(-0.42 to 0.3)
High-income Asia Pacific	Liver cancer	614780(594969 to 637246)	920379(842591 to 983716)	0.5(0.37 to 0.61)	295.64(285.8 to 306.34)	238.61(220.55 to 255.49)	-0.19(-0.25 to -0.13)
High-income Asia Pacific	Liver cancer due to alcohol use	80506(66759 to 96637)	125755(98264 to 157731)	0.56(0.36 to 0.79)	38.33(31.86 to 46.03)	32.92(25.61 to 41.92)	-0.14(-0.25 to -0.02)
High-income Asia Pacific	Liver cancer due to hepatitis B	165438(144524 to 188504)	286993(242797 to 334849)	0.73(0.46 to 1.02)	79.27(69.37 to 90.03)	88.63(75.76 to 102.37)	0.12(-0.06 to 0.3)
High-income Asia Pacific	Liver cancer due to hepatitis C	324640(302997 to 346247)	438136(381840 to 484032)	0.35(0.22 to 0.45)	155.81(145.47 to 165.97)	98.19(87.14 to 109.03)	-0.37(-0.41 to -0.33)
High-income Asia Pacific	Liver cancer due to NASH	22944(19094 to 27156)	42252(32816 to 53751)	0.84(0.58 to 1.14)	11.09(9.26 to 13.1)	10.04(7.92 to 12.73)	-0.09(-0.2 to 0.03)
High-income Asia Pacific	Liver cancer due to other causes	21251(17857 to 24657)	27244(21677 to 33506)	0.28(0.1 to 0.47)	11.14(9.5 to 12.79)	8.83(7.25 to 10.55)	-0.21(-0.3 to -0.11)
High-income North America	Liver cancer	167210(163014 to 170516)	608194(543851 to 664431)	2.64(2.25 to 2.97)	50.96(49.8 to 51.95)	105.46(94.47 to 115.21)	1.07(0.85 to 1.26)

High-income North America	Liver cancer due to alcohol use	52711(46742 to 58668)	209495(171086 to 246890)	2.97(2.45 to 3.43)	15.79(13.97 to 17.56)	35.65(29.18 to 42.17)	1.26(0.96 to 1.51)
High-income North America	Liver cancer due to hepatitis B	25416(22042 to 29113)	77145(62363 to 95405)	2.04(1.63 to 2.43)	7.92(6.88 to 9.08)	14.4(11.75 to 17.58)	0.82(0.58 to 1.03)
High-income North America	Liver cancer due to hepatitis C	53809(48012 to 59242)	209530(177240 to 243188)	2.89(2.48 to 3.25)	15.95(14.21 to 17.6)	34.63(29.28 to 40.27)	1.17(0.95 to 1.37)
High-income North America	Liver cancer due to NASH	14751(12698 to 17258)	58599(47529 to 70828)	2.97(2.54 to 3.34)	4.3(3.72 to 4.99)	9.81(8.02 to 11.76)	1.28(1.04 to 1.49)
High-income North America	Liver cancer due to other causes	20522(18396 to 22684)	53425(44901 to 62895)	1.6(1.32 to 1.88)	7(6.32 to 7.7)	10.97(9.39 to 12.63)	0.57(0.42 to 0.72)
North Africa and Middle East	Liver cancer	319736(284681 to 355483)	731622(578678 to 923575)	1.29(0.76 to 2.04)	161.07(141.87 to 179.7)	153.33(121.85 to 189.79)	-0.05(-0.27 to 0.28)
North Africa and Middle East	Liver cancer due to alcohol use	25514(16970 to 36861)	67530(42111 to 104202)	1.65(1.01 to 2.57)	13.95(9.24 to 19.83)	14.62(9.28 to 22.5)	0.05(-0.2 to 0.41)
North Africa and Middle East	Liver cancer due to hepatitis B	100271(80419 to 123896)	209881(156888 to 270076)	1.09(0.67 to 1.62)	48.82(38.7 to 60.35)	40.97(30.53 to 53.59)	-0.16(-0.33 to 0.05)
North Africa and Middle East	Liver cancer due to hepatitis C	131171(102796 to 161854)	322058(224228 to 441692)	1.46(0.74 to 2.53)	73.15(57.41 to 89.18)	70.9(50.55 to 96.51)	-0.03(-0.3 to 0.38)
North Africa and Middle East	Liver cancer due to NASH	22189(16211 to 30720)	70428(47529 to 103959)	2.17(1.4 to 3.36)	11.92(8.56 to 16.71)	15.25(10.46 to 22.23)	0.28(-0.03 to 0.76)
North Africa and Middle East	Liver cancer due to other causes	40590(31202 to 53609)	61725(45328 to 82688)	0.52(0.11 to 1.06)	13.23(10.2 to 17.01)	11.59(8.48 to 15.64)	-0.12(-0.31 to 0.15)
Oceania	Liver cancer	3557(3003 to 4124)	7093(5872 to 8495)	0.99(0.63 to 1.45)	98.31(82.63 to 114.22)	85.4(71.26 to 101.58)	-0.13(-0.29 to 0.07)
Oceania	Liver cancer due to alcohol use	360(225 to 544)	806(502 to 1220)	1.24(0.76 to 1.8)	11.15(7.12 to 16.59)	10.76(6.77 to	-0.04(-0.23 to 0.21)

						16.02)	
Oceania	Liver cancer due to hepatitis B	2071(1642 to 2543)	3967(3118 to 5063)	0.91(0.54 to 1.35)	54.57(42.7 to 67.68)	44.83(34.76 to 57.75)	-0.18(-0.34 to 0.01)
Oceania	Liver cancer due to hepatitis C	559(356 to 797)	1145(741 to 1666)	1.05(0.68 to 1.56)	19.62(12.92 to 27.26)	17.32(11.62 to 24.59)	-0.12(-0.28 to 0.09)
Oceania	Liver cancer due to NASH	228(160 to 332)	536(366 to 756)	1.35(0.91 to 1.91)	6.94(4.77 to 10.11)	7.15(4.91 to 10.17)	0.03(-0.15 to 0.27)
Oceania	Liver cancer due to other causes	338(263 to 429)	639(492 to 820)	0.89(0.5 to 1.35)	6.02(4.48 to 7.93)	5.35(4.04 to 7.1)	-0.11(-0.28 to 0.09)
South Asia	Liver cancer	512899(446428 to 575148)	1085515(943943 to 1244802)	1.12(0.79 to 1.5)	72.93(61.8 to 82.99)	71.28(61.99 to 81.83)	-0.02(-0.18 to 0.16)
South Asia	Liver cancer due to alcohol use	98371(76333 to 122362)	257774(205959 to 313411)	1.62(1.12 to 2.22)	16(12.39 to 19.7)	17.42(13.98 to 21.15)	0.09(-0.12 to 0.34)
South Asia	Liver cancer due to hepatitis B	197535(165277 to 231473)	386907(320870 to 462553)	0.96(0.62 to 1.35)	27.26(22.55 to 32.2)	24.32(20.14 to 29.26)	-0.11(-0.26 to 0.07)
South Asia	Liver cancer due to hepatitis C	93282(72908 to 113744)	228543(185704 to 274694)	1.45(0.99 to 1.94)	16.63(12.91 to 20.45)	16.16(13.17 to 19.45)	-0.03(-0.22 to 0.17)
South Asia	Liver cancer due to NASH	39853(32346 to 48187)	104694(84461 to 127877)	1.63(1.18 to 2.13)	6.32(5.07 to 7.72)	7.08(5.71 to 8.65)	0.12(-0.08 to 0.33)
South Asia	Liver cancer due to other causes	83859(69688 to 100752)	107597(90920 to 126810)	0.28(0.02 to 0.58)	6.71(5.74 to 7.81)	6.3(5.33 to 7.41)	-0.06(-0.22 to 0.12)
Southeast Asia	Liver cancer	551810(492855 to 604067)	1149098(943489 to 1384243)	1.08(0.67 to 1.56)	180.27(161.24 to 197.51)	177.53(146.55 to 213.53)	-0.02(-0.21 to 0.21)
Southeast Asia	Liver cancer due to alcohol use	100244(73479 to 131729)	283538(197789 to 397258)	1.83(1.17 to 2.63)	35.97(26.4 to 47.61)	44.14(31.11 to 61.31)	0.23(-0.06 to 0.57)
Southeast Asia	Liver cancer due to hepatitis B	265658(221085 to 311776)	501601(381028 to 649563)	0.89(0.47 to 1.38)	83.21(68.46 to 99.32)	73.04(55.46 to 94.77)	-0.12(-0.31 to 0.11)

Southeast Asia	Liver cancer due to hepatitis C	93497(70039 to 120869)	208013(153539 to 277671)	1.22(0.83 to 1.71)	36.72(27.83 to 46.73)	34.99(26.18 to 45.54)	-0.05(-0.22 to 0.16)
Southeast Asia	Liver cancer due to NASH	34038(26190 to 43895)	96201(68849 to 131532)	1.83(1.23 to 2.6)	12.11(9.2 to 15.91)	15.6(11.2 to 21.42)	0.29(0.02 to 0.63)
Southeast Asia	Liver cancer due to other causes	58373(42479 to 86640)	59745(46576 to 76265)	0.02(-0.37 to 0.46)	12.25(9.23 to 17.22)	9.76(7.64 to 12.48)	-0.2(-0.47 to 0.1)
Southern Latin America	Liver cancer	18439(16686 to 20246)	43534(40967 to 46273)	1.36(1.12 to 1.66)	39.13(35.43 to 42.9)	53.59(50.41 to 57)	0.37(0.23 to 0.54)
Southern Latin America	Liver cancer due to alcohol use	5884(4225 to 7653)	14210(10372 to 18194)	1.42(1.11 to 1.8)	12.48(8.94 to 16.24)	17.23(12.55 to 22.13)	0.38(0.21 to 0.6)
Southern Latin America	Liver cancer due to hepatitis B	3839(2751 to 5265)	8240(5718 to 11528)	1.15(0.88 to 1.48)	8.13(5.81 to 11.18)	10.34(7.22 to 14.41)	0.27(0.12 to 0.46)
Southern Latin America	Liver cancer due to hepatitis C	5376(3865 to 7140)	14000(10127 to 18143)	1.6(1.32 to 1.98)	11.6(8.45 to 15.32)	16.77(12.09 to 21.72)	0.45(0.29 to 0.64)
Southern Latin America	Liver cancer due to NASH	1238(879 to 1788)	3892(2732 to 5494)	2.14(1.74 to 2.64)	2.66(1.89 to 3.83)	4.7(3.33 to 6.55)	0.77(0.54 to 1.03)
Southern Latin America	Liver cancer due to other causes	2102(1739 to 2539)	3192(2447 to 4126)	0.52(0.29 to 0.76)	4.25(3.5 to 5.17)	4.55(3.62 to 5.7)	0.07(-0.06 to 0.22)
Southern Sub-Saharan Africa	Liver cancer	59594(42648 to 98286)	122195(108238 to 137902)	1.05(0.16 to 2.09)	181.69(128.88 to 299.99)	188.75(168.33 to 212.96)	0.04(-0.41 to 0.57)
Southern Sub-Saharan Africa	Liver cancer due to alcohol use	10381(6277 to 20061)	22485(17681 to 28412)	1.17(0.1 to 2.5)	34.07(20.45 to 65.93)	36.1(28.32 to 45.65)	0.06(-0.46 to 0.72)
Southern Sub-Saharan Africa	Liver cancer due to hepatitis B	27134(18555 to 46538)	53648(45000 to 63856)	0.98(0.05 to 2.1)	76.92(51.68 to 133.76)	76.74(64.35 to 91.52)	0(-0.47 to 0.56)

Southern Sub-Saharan Africa	Liver cancer due to hepatitis C	12098(8379 to 18190)	26002(20921 to 31675)	1.15(0.38 to 2.07)	43.14(29.51 to 65.08)	45.28(36.64 to 54.58)	0.05(-0.33 to 0.51)
Southern Sub-Saharan Africa	Liver cancer due to NASH	5044(3430 to 8152)	11863(9617 to 14500)	1.35(0.42 to 2.5)	15.83(10.54 to 25.93)	19.09(15.54 to 23.25)	0.21(-0.28 to 0.8)
Southern Sub-Saharan Africa	Liver cancer due to other causes	4937(3719 to 7390)	8196(6668 to 10110)	0.66(0.08 to 1.27)	11.73(8.56 to 17.81)	11.54(9.43 to 14.17)	-0.02(-0.37 to 0.37)
Tropical Latin America	Liver cancer	54246(52380 to 55933)	142719(135353 to 150317)	1.63(1.48 to 1.8)	52.15(50.03 to 53.82)	58.61(55.4 to 61.74)	0.12(0.06 to 0.19)
Tropical Latin America	Liver cancer due to alcohol use	13665(11821 to 15693)	43653(37574 to 49995)	2.19(1.98 to 2.43)	13.96(12.08 to 15.99)	17.62(15.17 to 20.12)	0.26(0.18 to 0.35)
Tropical Latin America	Liver cancer due to hepatitis B	13823(12005 to 15694)	29658(25272 to 34517)	1.15(1 to 1.32)	12.35(10.69 to 14.12)	11.84(10.09 to 13.78)	-0.04(-0.1 to 0.03)
Tropical Latin America	Liver cancer due to hepatitis C	16317(14342 to 18322)	50058(43578 to 56548)	2.07(1.88 to 2.27)	17.8(15.77 to 19.91)	20.6(17.92 to 23.21)	0.16(0.09 to 0.23)
Tropical Latin America	Liver cancer due to NASH	2767(2387 to 3182)	8680(7415 to 10207)	2.14(1.93 to 2.35)	2.75(2.36 to 3.18)	3.56(3.05 to 4.18)	0.29(0.22 to 0.37)
Tropical Latin America	Liver cancer due to other causes	7674(6937 to 8547)	10669(9387 to 12121)	0.39(0.21 to 0.59)	5.29(4.78 to 5.86)	4.98(4.34 to 5.67)	-0.06(-0.17 to 0.07)
Western Europe	Liver cancer	439140(426685 to 449642)	787717(738440 to 836208)	0.79(0.69 to 0.91)	80.71(78.49 to 82.56)	98.5(92.97 to 104.52)	0.22(0.15 to 0.3)
Western Europe	Liver cancer due to alcohol use	157173(128094 to 186139)	289150(232244 to 345094)	0.84(0.71 to 0.99)	28.43(23.14 to 33.8)	35.94(28.71 to 43.06)	0.26(0.18 to 0.36)
Western Europe	Liver cancer due to hepatitis B	64808(49636 to 82612)	103296(76204 to 137069)	0.59(0.46 to 0.75)	12.68(9.89 to 16)	14.6(11.09 to 18.96)	0.15(0.06 to 0.26)
Western Europe	Liver cancer due to hepatitis C	171902(144089 to 202575)	308839(250962 to 370796)	0.8(0.67 to 0.92)	30.27(25.27 to 35.8)	36.01(28.98 to 43.6)	0.19(0.11 to 0.27)

Western Europe	Liver cancer due to NASH	22267(17087 to 29384)	48876(36569 to 65750)	1.19(1.02 to 1.39)	3.96(3.05 to 5.17)	5.83(4.42 to 7.83)	0.47(0.36 to 0.59)
Western Europe	Liver cancer due to other causes	22990(18803 to 28416)	37555(28774 to 48614)	0.63(0.49 to 0.78)	5.36(4.57 to 6.38)	6.11(5 to 7.51)	0.14(0.04 to 0.23)
Western Sub-Saharan Africa	Liver cancer	172397(145973 to 199308)	308593(252949 to 365495)	0.79(0.42 to 1.19)	151.29(129.74 to 176.29)	130.76(109.41 to 152.3)	-0.14(-0.31 to 0.05)
Western Sub-Saharan Africa	Liver cancer due to alcohol use	22976(16264 to 31523)	48270(33594 to 64822)	1.1(0.6 to 1.64)	24.6(17.52 to 33.86)	24.15(17.06 to 32.26)	-0.02(-0.24 to 0.22)
Western Sub-Saharan Africa	Liver cancer due to hepatitis B	79100(63043 to 96415)	148026(115874 to 184845)	0.87(0.46 to 1.33)	74.29(59 to 91.51)	61.52(47.79 to 76.81)	-0.17(-0.35 to 0.02)
Western Sub-Saharan Africa	Liver cancer due to hepatitis C	24352(17171 to 32302)	44831(31310 to 58275)	0.84(0.51 to 1.22)	27.8(19.84 to 36.74)	24.11(17.12 to 31.46)	-0.13(-0.29 to 0.04)
Western Sub-Saharan Africa	Liver cancer due to NASH	9581(7148 to 12602)	21899(16225 to 29199)	1.29(0.84 to 1.82)	10.07(7.4 to 13.4)	10.69(7.96 to 14.43)	0.06(-0.14 to 0.3)
Western Sub-Saharan Africa	Liver cancer due to other causes	36386(28126 to 47076)	45567(32472 to 59726)	0.25(-0.13 to 0.68)	14.53(11.6 to 18.12)	10.28(7.83 to 13.33)	-0.29(-0.46 to -0.11)

Table S4. The mortality of liver cancer and underlying etiologies between 1990 and 2019 at national level, both sexes

**regions**	**Causes**	**Cases in 1990**	**Cases in 2019**	**Change in absolute number (95% UI)**	**ASRs in 1990**	**ASRs in 2019**	**change in ASR per 100 000 population (95% UI)**
Afghanistan	Liver cancer	851(657 to 1089)	1376(1041 to 1789)	0.62(0.16 to 1.24)	11.97(9.38 to 15.15)	10.27(7.98 to 12.93)	-0.14(-0.37 to 0.16)

Afghanistan	Liver cancer due to alcohol use	71(42 to 112)	103(63 to 159)	0.45(0.02 to 1.08)	1.01(0.6 to 1.56)	0.88(0.53 to 1.33)	-0.13(-0.38 to 0.19)
Afghanistan	Liver cancer due to hepatitis B	333(232 to 462)	572(398 to 798)	0.72(0.2 to 1.48)	4.42(3.11 to 6.12)	3.6(2.51 to 4.96)	-0.18(-0.42 to 0.13)
Afghanistan	Liver cancer due to hepatitis C	318(214 to 442)	451(300 to 635)	0.42(0.02 to 0.99)	4.81(3.32 to 6.52)	4.13(2.91 to 5.72)	-0.14(-0.37 to 0.14)
Afghanistan	Liver cancer due to NASH	63(41 to 94)	113(72 to 164)	0.81(0.28 to 1.6)	0.9(0.6 to 1.34)	0.9(0.57 to 1.34)	0(-0.28 to 0.37)
Afghanistan	Liver cancer due to other causes	67(45 to 97)	136(95 to 194)	1.03(0.45 to 1.93)	0.83(0.55 to 1.22)	0.75(0.49 to 1.1)	-0.1(-0.36 to 0.27)
Albania	Liver cancer	233(217 to 249)	294(215 to 392)	0.26(-0.09 to 0.68)	11.62(10.81 to 12.44)	6.84(5.04 to 9.09)	-0.41(-0.57 to -0.22)
Albania	Liver cancer due to alcohol use	68(50 to 89)	111(70 to 163)	0.63(0.16 to 1.25)	3.42(2.48 to 4.47)	2.53(1.6 to 3.68)	-0.26(-0.48 to 0.01)
Albania	Liver cancer due to hepatitis B	70(51 to 92)	68(42 to 103)	-0.03(-0.31 to 0.31)	3.22(2.34 to 4.25)	1.64(1.04 to 2.44)	-0.49(-0.63 to -0.31)
Albania	Liver cancer due to hepatitis C	67(47 to 88)	81(51 to 120)	0.21(-0.12 to 0.64)	3.62(2.61 to 4.72)	1.85(1.19 to 2.73)	-0.49(-0.62 to -0.31)
Albania	Liver cancer due to NASH	17(12 to 23)	24(15 to 38)	0.45(0.04 to 0.99)	0.88(0.61 to 1.24)	0.56(0.35 to 0.86)	-0.36(-0.53 to -0.13)
Albania	Liver cancer due to other causes	11(8 to 15)	10(6 to 15)	-0.12(-0.38 to 0.18)	0.49(0.34 to 0.67)	0.26(0.17 to 0.38)	-0.48(-0.62 to -0.3)
Algeria	Liver cancer	209(170 to 258)	688(533 to 874)	2.29(1.38 to 3.4)	1.81(1.48 to 2.2)	2.2(1.72 to 2.78)	0.21(-0.11 to 0.59)
Algeria	Liver cancer due to alcohol use	19(12 to 30)	72(43 to 111)	2.71(1.56 to 4.23)	0.17(0.1 to 0.25)	0.23(0.13 to 0.35)	0.36(-0.05 to 0.89)
Algeria	Liver cancer due to hepatitis B	77(54 to 105)	235(162 to 330)	2.05(1.17 to 3.19)	0.61(0.43 to 0.82)	0.68(0.46 to 0.96)	0.13(-0.2 to 0.55)

Algeria	Liver cancer due to hepatitis C	73(50 to 100)	256(178 to 350)	2.5(1.5 to 3.71)	0.75(0.54 to 0.97)	0.89(0.64 to 1.21)	0.2(-0.12 to 0.55)
Algeria	Liver cancer due to NASH	17(11 to 26)	76(48 to 114)	3.4(2.08 to 5.15)	0.16(0.1 to 0.24)	0.25(0.16 to 0.37)	0.56(0.12 to 1.11)
Algeria	Liver cancer due to other causes	22(17 to 28)	49(34 to 68)	1.21(0.6 to 1.97)	0.13(0.09 to 0.18)	0.14(0.1 to 0.2)	0.1(-0.17 to 0.44)
American Samoa	Liver cancer	1(1 to 2)	3(3 to 4)	1.56(0.96 to 2.42)	5.69(4.37 to 6.94)	7.02(5.85 to 8.45)	0.23(-0.05 to 0.63)
American Samoa	Liver cancer due to alcohol use	0(0 to 0)	0(0 to 1)	1.68(1 to 2.67)	0.62(0.38 to 0.95)	0.78(0.49 to 1.17)	0.26(-0.06 to 0.68)
American Samoa	Liver cancer due to hepatitis B	1(1 to 1)	2(1 to 2)	1.49(0.83 to 2.45)	2.84(1.97 to 3.75)	3.58(2.73 to 4.65)	0.26(-0.05 to 0.72)
American Samoa	Liver cancer due to hepatitis C	0(0 to 0)	1(0 to 1)	1.6(1.01 to 2.4)	1.37(0.9 to 1.88)	1.53(1.05 to 2.08)	0.12(-0.13 to 0.44)
American Samoa	Liver cancer due to NASH	0(0 to 0)	0(0 to 1)	2.01(1.27 to 3.02)	0.61(0.4 to 0.88)	0.84(0.59 to 1.2)	0.38(0.05 to 0.82)
American Samoa	Liver cancer due to other causes	0(0 to 0)	0(0 to 0)	1.07(0.53 to 1.77)	0.25(0.16 to 0.36)	0.28(0.19 to 0.4)	0.14(-0.15 to 0.56)
Andorra	Liver cancer	5(4 to 7)	13(10 to 17)	1.65(0.76 to 2.85)	9.38(7.05 to 12.68)	9.44(7.14 to 12.24)	0.01(-0.33 to 0.45)
Andorra	Liver cancer due to alcohol use	2(1 to 3)	5(3 to 7)	1.37(0.56 to 2.54)	3.9(2.66 to 5.72)	3.73(2.45 to 5.15)	-0.04(-0.37 to 0.41)
Andorra	Liver cancer due to hepatitis B	1(0 to 1)	2(1 to 2)	1.28(0.51 to 2.4)	1.18(0.72 to 1.83)	1.12(0.7 to 1.75)	-0.05(-0.38 to 0.4)
Andorra	Liver cancer due to hepatitis C	2(1 to 3)	5(3 to 7)	2.06(1.01 to 3.51)	3.33(2.19 to 4.98)	3.51(2.33 to 4.97)	0.05(-0.3 to 0.53)
Andorra	Liver cancer due to NASH	0(0 to 0)	1(1 to 1)	2.36(1.16 to 4.1)	0.53(0.32 to 0.84)	0.65(0.4 to 1.01)	0.22(-0.21 to 0.78)

Andorra	Liver cancer due to other causes	0(0 to 0)	1(0 to 1)	1.53(0.6 to 2.88)	0.43(0.27 to 0.65)	0.43(0.27 to 0.66)	-0.01(-0.35 to 0.51)
Angola	Liver cancer	117(91 to 150)	302(232 to 385)	1.58(0.8 to 2.65)	2.75(2.15 to 3.43)	2.59(2.09 to 3.23)	-0.06(-0.3 to 0.29)
Angola	Liver cancer due to alcohol use	14(8 to 21)	46(29 to 69)	2.36(1.26 to 3.97)	0.38(0.23 to 0.57)	0.44(0.28 to 0.65)	0.17(-0.2 to 0.76)
Angola	Liver cancer due to hepatitis B	31(21 to 43)	76(50 to 109)	1.48(0.68 to 2.66)	0.65(0.44 to 0.93)	0.55(0.37 to 0.79)	-0.16(-0.41 to 0.2)
Angola	Liver cancer due to hepatitis C	45(31 to 61)	114(81 to 155)	1.53(0.82 to 2.55)	1.39(1 to 1.84)	1.26(0.93 to 1.65)	-0.1(-0.33 to 0.24)
Angola	Liver cancer due to NASH	6(4 to 8)	19(12 to 28)	2.29(1.26 to 3.73)	0.16(0.1 to 0.24)	0.18(0.12 to 0.27)	0.16(-0.17 to 0.63)
Angola	Liver cancer due to other causes	22(13 to 37)	47(27 to 77)	1.15(-0.06 to 3.44)	0.18(0.12 to 0.27)	0.16(0.11 to 0.24)	-0.11(-0.5 to 0.44)
Antigua and Barbuda	Liver cancer	4(4 to 5)	3(2 to 3)	-0.36(-0.46 to -0.24)	8.11(7.35 to 8.96)	2.85(2.44 to 3.31)	-0.65(-0.7 to -0.58)
Antigua and Barbuda	Liver cancer due to alcohol use	1(1 to 2)	1(1 to 1)	-0.29(-0.42 to -0.12)	2.6(1.88 to 3.39)	1(0.72 to 1.35)	-0.61(-0.68 to -0.52)
Antigua and Barbuda	Liver cancer due to hepatitis B	1(1 to 2)	1(0 to 1)	-0.44(-0.54 to -0.31)	2.44(1.76 to 3.28)	0.69(0.48 to 0.97)	-0.72(-0.77 to -0.66)
Antigua and Barbuda	Liver cancer due to hepatitis C	1(1 to 1)	1(0 to 1)	-0.4(-0.5 to -0.28)	1.94(1.34 to 2.62)	0.71(0.49 to 0.98)	-0.64(-0.7 to -0.57)
Antigua and Barbuda	Liver cancer due to NASH	0(0 to 1)	0(0 to 0)	-0.25(-0.4 to -0.05)	0.63(0.44 to 0.9)	0.28(0.19 to 0.4)	-0.55(-0.64 to -0.45)
Antigua and Barbuda	Liver cancer due to other causes	0(0 to 0)	0(0 to 0)	-0.4(-0.52 to -0.24)	0.5(0.35 to 0.68)	0.17(0.12 to 0.24)	-0.65(-0.72 to -0.57)
Argentina	Liver cancer	481(419 to 545)	1172(1089 to 1268)	1.44(1.1 to 1.89)	1.51(1.32 to 1.7)	2.15(2 to 2.33)	0.43(0.24 to 0.69)

Argentina	Liver cancer due to alcohol use	166(116 to 220)	385(274 to 497)	1.32(0.9 to 1.84)	0.51(0.36 to 0.68)	0.7(0.5 to 0.91)	0.38(0.13 to 0.69)
Argentina	Liver cancer due to hepatitis B	82(55 to 117)	188(129 to 270)	1.29(0.9 to 1.8)	0.25(0.17 to 0.36)	0.35(0.24 to 0.5)	0.4(0.16 to 0.69)
Argentina	Liver cancer due to hepatitis C	164(118 to 221)	425(313 to 539)	1.59(1.22 to 2.1)	0.53(0.39 to 0.7)	0.77(0.57 to 0.98)	0.46(0.26 to 0.74)
Argentina	Liver cancer due to NASH	36(24 to 54)	110(75 to 158)	2.08(1.56 to 2.81)	0.11(0.08 to 0.17)	0.2(0.14 to 0.28)	0.75(0.46 to 1.16)
Argentina	Liver cancer due to other causes	33(25 to 44)	65(46 to 89)	0.95(0.61 to 1.34)	0.1(0.08 to 0.13)	0.13(0.09 to 0.17)	0.24(0.04 to 0.46)
Armenia	Liver cancer	29(28 to 31)	289(240 to 343)	8.87(7.15 to 10.79)	1.13(1.06 to 1.21)	7.07(5.88 to 8.38)	5.26(4.17 to 6.46)
Armenia	Liver cancer due to alcohol use	7(5 to 9)	85(59 to 117)	11.38(8.8 to 14.39)	0.27(0.19 to 0.36)	2.06(1.44 to 2.8)	6.63(5.1 to 8.44)
Armenia	Liver cancer due to hepatitis B	7(5 to 9)	57(38 to 83)	7.49(5.75 to 9.47)	0.24(0.17 to 0.33)	1.4(0.94 to 2.03)	4.78(3.65 to 6.05)
Armenia	Liver cancer due to hepatitis C	11(8 to 13)	114(81 to 150)	9.72(7.83 to 11.87)	0.45(0.34 to 0.55)	2.78(2 to 3.62)	5.23(4.1 to 6.44)
Armenia	Liver cancer due to NASH	2(1 to 2)	23(16 to 34)	13.44(10.28 to 17.12)	0.07(0.05 to 0.1)	0.57(0.39 to 0.83)	7.51(5.78 to 9.68)
Armenia	Liver cancer due to other causes	3(3 to 4)	10(6 to 14)	1.8(0.8 to 3.39)	0.1(0.08 to 0.13)	0.26(0.17 to 0.37)	1.49(0.66 to 2.81)
Australia	Liver cancer	382(365 to 399)	1726(1566 to 1886)	3.52(3.12 to 3.92)	1.96(1.87 to 2.04)	4.2(3.83 to 4.59)	1.15(0.96 to 1.34)
Australia	Liver cancer due to alcohol use	163(124 to 200)	659(497 to 823)	3.03(2.55 to 3.52)	0.82(0.63 to 1.01)	1.62(1.22 to 2.01)	0.96(0.73 to 1.2)
Australia	Liver cancer due to hepatitis B	57(39 to 81)	210(143 to 302)	2.71(2.21 to 3.2)	0.3(0.21 to 0.41)	0.55(0.38 to 0.79)	0.88(0.63 to 1.11)

Australia	Liver cancer due to hepatitis C	115(84 to 149)	589(431 to 763)	4.14(3.6 to 4.74)	0.59(0.43 to 0.76)	1.37(1 to 1.78)	1.34(1.12 to 1.59)
Australia	Liver cancer due to NASH	29(21 to 42)	193(134 to 264)	5.56(4.43 to 6.79)	0.15(0.11 to 0.21)	0.45(0.32 to 0.62)	2.03(1.56 to 2.55)
Australia	Liver cancer due to other causes	18(13 to 24)	75(52 to 103)	3.14(2.45 to 3.83)	0.1(0.08 to 0.13)	0.2(0.14 to 0.27)	1.01(0.73 to 1.3)
Austria	Liver cancer	387(368 to 405)	799(714 to 886)	1.06(0.86 to 1.3)	3.3(3.14 to 3.46)	4.5(4.04 to 4.99)	0.37(0.23 to 0.52)
Austria	Liver cancer due to alcohol use	203(167 to 236)	420(336 to 506)	1.07(0.81 to 1.36)	1.74(1.44 to 2.02)	2.4(1.94 to 2.9)	0.38(0.2 to 0.58)
Austria	Liver cancer due to hepatitis B	28(19 to 41)	50(32 to 75)	0.75(0.47 to 1.02)	0.26(0.18 to 0.37)	0.31(0.2 to 0.46)	0.17(-0.01 to 0.35)
Austria	Liver cancer due to hepatitis C	121(89 to 158)	256(187 to 342)	1.12(0.86 to 1.38)	0.99(0.73 to 1.28)	1.37(0.99 to 1.85)	0.39(0.22 to 0.56)
Austria	Liver cancer due to NASH	17(12 to 25)	41(27 to 62)	1.4(1.04 to 1.81)	0.14(0.1 to 0.2)	0.22(0.15 to 0.33)	0.59(0.36 to 0.86)
Austria	Liver cancer due to other causes	18(13 to 25)	33(22 to 48)	0.87(0.59 to 1.22)	0.17(0.13 to 0.22)	0.21(0.14 to 0.29)	0.24(0.06 to 0.45)
Azerbaijan	Liver cancer	51(46 to 56)	365(280 to 484)	6.18(4.37 to 8.94)	1.02(0.92 to 1.14)	4.37(3.31 to 5.94)	3.28(2.16 to 5.13)
Azerbaijan	Liver cancer due to alcohol use	11(8 to 15)	100(62 to 152)	8.03(5.46 to 12.19)	0.24(0.16 to 0.32)	1.16(0.72 to 1.76)	3.92(2.5 to 6.33)
Azerbaijan	Liver cancer due to hepatitis B	12(8 to 16)	92(61 to 137)	6.75(4.76 to 9.84)	0.23(0.16 to 0.31)	0.94(0.62 to 1.39)	3.11(2.02 to 4.8)
Azerbaijan	Liver cancer due to hepatitis C	18(13 to 23)	129(85 to 188)	6.18(4.18 to 9.07)	0.4(0.3 to 0.5)	1.72(1.13 to 2.58)	3.32(2.05 to 5.29)
Azerbaijan	Liver cancer due to NASH	3(2 to 4)	28(18 to 44)	8.62(5.92 to 12.72)	0.06(0.04 to 0.09)	0.37(0.23 to 0.6)	4.79(3.02 to 7.56)

Azerbaijan	Liver cancer due to other causes	7(6 to 9)	16(11 to 24)	1.3(0.61 to 2.39)	0.1(0.08 to 0.12)	0.19(0.13 to 0.29)	0.96(0.42 to 1.89)
Bahamas	Liver cancer	13(12 to 15)	12(10 to 15)	-0.07(-0.26 to 0.19)	8.47(7.48 to 9.48)	3.21(2.66 to 3.96)	-0.62(-0.7 to -0.52)
Bahamas	Liver cancer due to alcohol use	5(4 to 6)	4(3 to 6)	-0.13(-0.32 to 0.13)	3.16(2.33 to 4.07)	1.09(0.75 to 1.49)	-0.66(-0.73 to -0.55)
Bahamas	Liver cancer due to hepatitis B	4(3 to 5)	4(3 to 5)	-0.11(-0.31 to 0.16)	2.4(1.76 to 3.26)	0.87(0.62 to 1.21)	-0.64(-0.72 to -0.53)
Bahamas	Liver cancer due to hepatitis C	3(2 to 3)	3(2 to 4)	0.04(-0.17 to 0.3)	1.76(1.23 to 2.39)	0.72(0.48 to 1)	-0.59(-0.67 to -0.49)
Bahamas	Liver cancer due to NASH	1(1 to 1)	1(1 to 2)	0.2(-0.05 to 0.55)	0.66(0.46 to 0.92)	0.32(0.22 to 0.47)	-0.51(-0.61 to -0.37)
Bahamas	Liver cancer due to other causes	1(1 to 1)	1(1 to 1)	-0.1(-0.3 to 0.15)	0.49(0.35 to 0.68)	0.2(0.14 to 0.28)	-0.6(-0.69 to -0.48)
Bahrain	Liver cancer	9(7 to 11)	39(30 to 50)	3.3(2.19 to 4.85)	6.11(5.14 to 7.17)	5.23(4.12 to 6.53)	-0.15(-0.36 to 0.12)
Bahrain	Liver cancer due to alcohol use	1(1 to 2)	4(2 to 7)	2.98(1.82 to 4.65)	0.71(0.44 to 1.06)	0.52(0.31 to 0.81)	-0.26(-0.46 to 0.03)
Bahrain	Liver cancer due to hepatitis B	3(3 to 5)	15(10 to 21)	3.2(2.03 to 4.92)	1.88(1.33 to 2.6)	1.4(0.95 to 2.02)	-0.25(-0.45 to 0.02)
Bahrain	Liver cancer due to hepatitis C	3(2 to 4)	13(9 to 18)	3.29(2.16 to 4.76)	2.52(1.82 to 3.25)	2.25(1.6 to 2.96)	-0.11(-0.33 to 0.17)
Bahrain	Liver cancer due to NASH	1(1 to 1)	5(3 to 7)	4.46(2.98 to 6.49)	0.68(0.45 to 1.02)	0.77(0.51 to 1.16)	0.13(-0.17 to 0.56)
Bahrain	Liver cancer due to other causes	1(0 to 1)	2(1 to 3)	2.77(1.78 to 4.04)	0.33(0.22 to 0.48)	0.27(0.18 to 0.42)	-0.17(-0.37 to 0.12)
Bangladesh	Liver cancer	1740(1404 to 2117)	3521(2772 to 4396)	1.02(0.51 to 1.76)	3.24(2.68 to 3.91)	2.75(2.17 to 3.42)	-0.15(-0.37 to 0.12)

Bangladesh	Liver cancer due to alcohol use	330(221 to 471)	742(457 to 1089)	1.25(0.59 to 2.14)	0.7(0.46 to 1)	0.58(0.35 to 0.84)	-0.18(-0.41 to 0.15)
Bangladesh	Liver cancer due to hepatitis B	518(373 to 701)	779(538 to 1120)	0.5(0.07 to 1.11)	0.84(0.6 to 1.15)	0.56(0.39 to 0.82)	-0.33(-0.52 to -0.08)
Bangladesh	Liver cancer due to hepatitis C	562(403 to 760)	1502(1055 to 1991)	1.67(0.96 to 2.62)	1.3(0.94 to 1.75)	1.24(0.89 to 1.61)	-0.04(-0.29 to 0.27)
Bangladesh	Liver cancer due to NASH	111(77 to 155)	274(181 to 402)	1.47(0.75 to 2.42)	0.21(0.15 to 0.3)	0.22(0.14 to 0.32)	0.01(-0.26 to 0.36)
Bangladesh	Liver cancer due to other causes	219(153 to 313)	225(170 to 299)	0.03(-0.32 to 0.54)	0.19(0.14 to 0.26)	0.15(0.12 to 0.2)	-0.19(-0.42 to 0.16)
Barbados	Liver cancer	9(6 to 11)	13(11 to 16)	0.55(0.12 to 1.24)	2.91(2.16 to 3.72)	2.74(2.29 to 3.27)	-0.06(-0.32 to 0.36)
Barbados	Liver cancer due to alcohol use	3(2 to 4)	5(4 to 7)	0.74(0.19 to 1.68)	0.96(0.6 to 1.45)	1(0.7 to 1.33)	0.05(-0.28 to 0.62)
Barbados	Liver cancer due to hepatitis B	2(1 to 3)	3(2 to 4)	0.35(-0.06 to 1.07)	0.77(0.48 to 1.17)	0.6(0.43 to 0.86)	-0.21(-0.46 to 0.2)
Barbados	Liver cancer due to hepatitis C	2(1 to 3)	3(2 to 5)	0.48(0.11 to 1.03)	0.69(0.45 to 1.02)	0.64(0.44 to 0.91)	-0.07(-0.3 to 0.27)
Barbados	Liver cancer due to NASH	1(1 to 1)	1(1 to 2)	0.75(0.3 to 1.47)	0.27(0.17 to 0.4)	0.29(0.2 to 0.43)	0.11(-0.18 to 0.55)
Barbados	Liver cancer due to other causes	1(0 to 1)	1(1 to 1)	0.27(-0.07 to 0.74)	0.23(0.16 to 0.32)	0.2(0.14 to 0.27)	-0.12(-0.34 to 0.19)
Belarus	Liver cancer	167(158 to 176)	377(275 to 504)	1.26(0.65 to 2.03)	1.31(1.24 to 1.38)	2.41(1.76 to 3.24)	0.84(0.35 to 1.46)
Belarus	Liver cancer due to alcohol use	61(45 to 75)	162(105 to 235)	1.67(0.86 to 2.73)	0.46(0.35 to 0.58)	1.01(0.66 to 1.48)	1.18(0.52 to 2.08)
Belarus	Liver cancer due to hepatitis B	37(26 to 50)	72(44 to 113)	0.97(0.36 to 1.77)	0.28(0.2 to 0.38)	0.47(0.29 to 0.74)	0.68(0.16 to 1.37)

Belarus	Liver cancer due to hepatitis C	47(34 to 61)	99(64 to 142)	1.11(0.62 to 1.72)	0.37(0.27 to 0.47)	0.61(0.4 to 0.87)	0.65(0.26 to 1.14)
Belarus	Liver cancer due to NASH	12(8 to 16)	29(18 to 44)	1.49(0.85 to 2.3)	0.09(0.06 to 0.13)	0.18(0.11 to 0.28)	0.99(0.48 to 1.66)
Belarus	Liver cancer due to other causes	11(8 to 13)	15(10 to 21)	0.35(-0.02 to 0.83)	0.11(0.08 to 0.13)	0.13(0.1 to 0.18)	0.24(-0.1 to 0.69)
Belgium	Liver cancer	457(432 to 479)	865(786 to 943)	0.89(0.72 to 1.08)	2.93(2.78 to 3.07)	3.78(3.45 to 4.12)	0.29(0.17 to 0.42)
Belgium	Liver cancer due to alcohol use	164(123 to 207)	326(243 to 416)	0.98(0.74 to 1.24)	1.05(0.79 to 1.31)	1.47(1.1 to 1.87)	0.4(0.23 to 0.57)
Belgium	Liver cancer due to hepatitis B	55(37 to 79)	96(64 to 139)	0.74(0.54 to 0.96)	0.38(0.26 to 0.53)	0.47(0.32 to 0.67)	0.25(0.1 to 0.41)
Belgium	Liver cancer due to hepatitis C	188(144 to 233)	349(265 to 438)	0.85(0.66 to 1.06)	1.18(0.9 to 1.45)	1.42(1.07 to 1.81)	0.21(0.09 to 0.34)
Belgium	Liver cancer due to NASH	28(18 to 40)	58(38 to 84)	1.08(0.79 to 1.46)	0.17(0.12 to 0.25)	0.24(0.16 to 0.35)	0.38(0.19 to 0.64)
Belgium	Liver cancer due to other causes	21(14 to 30)	36(23 to 52)	0.72(0.46 to 1)	0.15(0.11 to 0.21)	0.18(0.12 to 0.24)	0.16(-0.01 to 0.34)
Belize	Liver cancer	6(5 to 7)	9(7 to 10)	0.45(0.22 to 0.71)	6.33(5.76 to 6.94)	3.21(2.75 to 3.7)	-0.49(-0.57 to -0.4)
Belize	Liver cancer due to alcohol use	2(1 to 2)	3(2 to 4)	0.65(0.34 to 1)	1.96(1.41 to 2.55)	1.13(0.82 to 1.48)	-0.42(-0.53 to -0.3)
Belize	Liver cancer due to hepatitis B	2(1 to 2)	2(2 to 3)	0.5(0.23 to 0.82)	1.68(1.24 to 2.26)	0.83(0.6 to 1.14)	-0.51(-0.6 to -0.4)
Belize	Liver cancer due to hepatitis C	1(1 to 2)	2(1 to 3)	0.22(0 to 0.47)	1.68(1.19 to 2.24)	0.74(0.5 to 1.02)	-0.56(-0.64 to -0.47)
Belize	Liver cancer due to NASH	1(0 to 1)	1(1 to 1)	0.63(0.29 to 1.04)	0.56(0.38 to 0.79)	0.32(0.22 to 0.46)	-0.42(-0.53 to -0.28)

Belize	Liver cancer due to other causes	1(0 to 1)	1(0 to 1)	0.11(-0.11 to 0.36)	0.47(0.34 to 0.62)	0.2(0.14 to 0.27)	-0.57(-0.66 to -0.48)
Benin	Liver cancer	144(116 to 182)	255(190 to 343)	0.77(0.26 to 1.5)	6.96(5.58 to 8.87)	5.12(3.89 to 6.79)	-0.26(-0.47 to 0.03)
Benin	Liver cancer due to alcohol use	24(16 to 37)	46(29 to 72)	0.9(0.34 to 1.72)	1.25(0.82 to 1.93)	1(0.62 to 1.55)	-0.2(-0.43 to 0.14)
Benin	Liver cancer due to hepatitis B	72(53 to 96)	120(82 to 167)	0.66(0.14 to 1.47)	3.42(2.52 to 4.6)	2.22(1.53 to 3.08)	-0.35(-0.55 to -0.07)
Benin	Liver cancer due to hepatitis C	26(16 to 37)	44(28 to 63)	0.73(0.29 to 1.37)	1.41(0.91 to 1.98)	1.08(0.71 to 1.52)	-0.23(-0.42 to 0.04)
Benin	Liver cancer due to NASH	10(7 to 15)	24(16 to 36)	1.34(0.67 to 2.27)	0.53(0.35 to 0.77)	0.53(0.35 to 0.8)	0(-0.27 to 0.38)
Benin	Liver cancer due to other causes	12(9 to 17)	21(15 to 31)	0.75(0.16 to 1.5)	0.35(0.24 to 0.48)	0.28(0.18 to 0.41)	-0.2(-0.42 to 0.12)
Bermuda	Liver cancer	5(4 to 5)	3(2 to 3)	-0.41(-0.52 to -0.27)	7.49(6.74 to 8.28)	2.05(1.7 to 2.51)	-0.73(-0.78 to -0.66)
Bermuda	Liver cancer due to alcohol use	2(1 to 2)	1(1 to 1)	-0.42(-0.54 to -0.26)	2.89(2.16 to 3.67)	0.79(0.57 to 1.06)	-0.73(-0.78 to -0.66)
Bermuda	Liver cancer due to hepatitis B	1(1 to 2)	1(0 to 1)	-0.46(-0.58 to -0.31)	1.78(1.28 to 2.5)	0.49(0.33 to 0.69)	-0.73(-0.78 to -0.66)
Bermuda	Liver cancer due to hepatitis C	1(1 to 1)	1(0 to 1)	-0.39(-0.51 to -0.22)	1.71(1.18 to 2.3)	0.45(0.29 to 0.64)	-0.74(-0.79 to -0.68)
Bermuda	Liver cancer due to NASH	0(0 to 1)	0(0 to 0)	-0.3(-0.46 to -0.09)	0.71(0.49 to 1)	0.22(0.15 to 0.32)	-0.69(-0.76 to -0.6)
Bermuda	Liver cancer due to other causes	0(0 to 0)	0(0 to 0)	-0.5(-0.62 to -0.36)	0.4(0.28 to 0.56)	0.11(0.07 to 0.15)	-0.73(-0.79 to -0.65)
Bhutan	Liver cancer	6(4 to 9)	18(12 to 25)	2(1.07 to 3.5)	2.29(1.55 to 3.33)	3.27(2.29 to 4.61)	0.43(0 to 1.06)

Bhutan	Liver cancer due to alcohol use	2(1 to 3)	5(3 to 8)	2.12(1.04 to 3.72)	0.66(0.4 to 1.09)	0.94(0.54 to 1.48)	0.41(-0.06 to 1.07)
Bhutan	Liver cancer due to hepatitis B	2(1 to 3)	4(3 to 7)	1.4(0.57 to 2.8)	0.63(0.37 to 1.03)	0.75(0.44 to 1.17)	0.19(-0.2 to 0.8)
Bhutan	Liver cancer due to hepatitis C	2(1 to 2)	6(4 to 9)	2.72(1.54 to 4.47)	0.71(0.44 to 1.1)	1.12(0.7 to 1.73)	0.57(0.12 to 1.25)
Bhutan	Liver cancer due to NASH	0(0 to 1)	2(1 to 3)	3.13(1.81 to 5.23)	0.18(0.11 to 0.28)	0.32(0.19 to 0.51)	0.83(0.28 to 1.66)
Bhutan	Liver cancer due to other causes	1(0 to 1)	1(1 to 1)	0.67(-0.02 to 2.77)	0.1(0.05 to 0.16)	0.14(0.09 to 0.21)	0.38(-0.08 to 1.4)
Bolivia (Plurinational State of)	Liver cancer	169(128 to 222)	427(319 to 550)	1.53(0.82 to 2.47)	5.29(4.04 to 6.95)	5.03(3.77 to 6.43)	-0.05(-0.3 to 0.29)
Bolivia (Plurinational State of)	Liver cancer due to alcohol use	45(28 to 66)	132(86 to 191)	1.96(1.14 to 3.17)	1.47(0.94 to 2.18)	1.57(1.02 to 2.27)	0.07(-0.22 to 0.47)
Bolivia (Plurinational State of)	Liver cancer due to hepatitis B	81(58 to 109)	183(124 to 254)	1.26(0.58 to 2.2)	2.45(1.74 to 3.32)	2.08(1.43 to 2.87)	-0.15(-0.4 to 0.18)
Bolivia (Plurinational State of)	Liver cancer due to hepatitis C	13(7 to 22)	36(20 to 59)	1.74(0.96 to 2.82)	0.48(0.27 to 0.81)	0.47(0.27 to 0.75)	-0.03(-0.3 to 0.34)
Bolivia (Plurinational State of)	Liver cancer due to NASH	13(8 to 22)	42(26 to 67)	2.24(1.28 to 3.68)	0.46(0.28 to 0.75)	0.53(0.34 to 0.83)	0.17(-0.16 to 0.65)
Bolivia (Plurinational State of)	Liver cancer due to other causes	18(12 to 27)	35(22 to 51)	0.97(0.36 to 1.8)	0.43(0.27 to 0.69)	0.38(0.24 to 0.58)	-0.12(-0.36 to 0.23)

Bosnia and Herzegovina	Liver cancer	233(217 to 251)	481(385 to 601)	1.06(0.64 to 1.59)	5.78(5.38 to 6.2)	8.01(6.4 to 10)	0.38(0.1 to 0.74)
Bosnia and Herzegovina	Liver cancer due to alcohol use	77(55 to 100)	168(114 to 232)	1.18(0.67 to 1.87)	1.89(1.39 to 2.47)	2.73(1.86 to 3.77)	0.44(0.11 to 0.87)
Bosnia and Herzegovina	Liver cancer due to hepatitis B	70(50 to 93)	99(64 to 151)	0.43(0.08 to 0.85)	1.59(1.15 to 2.13)	1.69(1.12 to 2.54)	0.07(-0.18 to 0.36)
Bosnia and Herzegovina	Liver cancer due to hepatitis C	62(43 to 84)	150(103 to 214)	1.41(0.84 to 2.1)	1.67(1.17 to 2.2)	2.5(1.74 to 3.5)	0.5(0.18 to 0.89)
Bosnia and Herzegovina	Liver cancer due to NASH	15(10 to 22)	47(31 to 70)	2.06(1.28 to 3.06)	0.41(0.28 to 0.59)	0.78(0.52 to 1.15)	0.92(0.46 to 1.49)
Bosnia and Herzegovina	Liver cancer due to other causes	9(6 to 13)	17(10 to 25)	0.85(0.34 to 1.44)	0.22(0.16 to 0.31)	0.3(0.19 to 0.44)	0.34(0.01 to 0.75)
Botswana	Liver cancer	6(3 to 13)	22(15 to 30)	2.74(0.62 to 6.91)	1.03(0.5 to 2.27)	1.53(1.13 to 2.02)	0.49(-0.32 to 2.09)
Botswana	Liver cancer due to alcohol use	1(0 to 3)	5(3 to 7)	3.33(0.7 to 9.24)	0.19(0.07 to 0.54)	0.32(0.2 to 0.5)	0.75(-0.28 to 3.09)
Botswana	Liver cancer due to hepatitis B	2(1 to 6)	9(6 to 13)	2.85(0.51 to 7.91)	0.35(0.14 to 0.92)	0.51(0.33 to 0.75)	0.46(-0.41 to 2.38)
Botswana	Liver cancer due to hepatitis C	2(1 to 3)	5(3 to 8)	2.18(0.65 to 5.18)	0.35(0.17 to 0.66)	0.46(0.3 to 0.64)	0.32(-0.3 to 1.56)
Botswana	Liver cancer due to NASH	0(0 to 1)	2(1 to 3)	3.28(1.1 to 7.75)	0.09(0.04 to 0.2)	0.16(0.1 to 0.24)	0.74(-0.12 to 2.54)
Botswana	Liver cancer due to other causes	0(0 to 1)	1(1 to 2)	2.2(0.7 to 4.71)	0.05(0.03 to 0.11)	0.07(0.05 to 0.11)	0.38(-0.29 to 1.66)
Brazil	Liver cancer	1830(1751 to 1894)	5822(5434 to 6127)	2.18(2 to 2.38)	2.09(1.98 to 2.17)	2.5(2.33 to 2.64)	0.2(0.13 to 0.27)

Brazil	Liver cancer due to alcohol use	499(433 to 569)	1782(1533 to 2023)	2.57(2.34 to 2.84)	0.57(0.49 to 0.65)	0.76(0.65 to 0.86)	0.33(0.25 to 0.43)
Brazil	Liver cancer due to hepatitis B	399(345 to 456)	998(840 to 1170)	1.5(1.32 to 1.7)	0.41(0.35 to 0.47)	0.42(0.35 to 0.49)	0.02(-0.04 to 0.1)
Brazil	Liver cancer due to hepatitis C	684(609 to 763)	2374(2082 to 2653)	2.47(2.27 to 2.71)	0.86(0.77 to 0.96)	1.04(0.91 to 1.15)	0.2(0.13 to 0.29)
Brazil	Liver cancer due to NASH	101(86 to 118)	374(316 to 440)	2.7(2.46 to 2.97)	0.12(0.1 to 0.14)	0.16(0.14 to 0.19)	0.36(0.27 to 0.44)
Brazil	Liver cancer due to other causes	147(132 to 162)	294(256 to 337)	1.01(0.82 to 1.2)	0.13(0.12 to 0.15)	0.13(0.12 to 0.15)	0.02(-0.06 to 0.11)
Brunei Darussalam	Liver cancer	9(8 to 11)	35(29 to 41)	2.69(1.91 to 3.74)	10.13(8.33 to 12.08)	11.53(9.82 to 13.38)	0.14(-0.08 to 0.43)
Brunei Darussalam	Liver cancer due to alcohol use	1(1 to 1)	3(2 to 5)	2.61(1.68 to 3.87)	0.95(0.58 to 1.44)	1.06(0.65 to 1.57)	0.11(-0.15 to 0.46)
Brunei Darussalam	Liver cancer due to hepatitis B	5(4 to 6)	18(13 to 23)	2.64(1.77 to 3.84)	4.36(3.21 to 5.78)	4.91(3.67 to 6.4)	0.13(-0.12 to 0.48)
Brunei Darussalam	Liver cancer due to hepatitis C	3(2 to 4)	10(7 to 14)	2.76(1.96 to 3.81)	3.86(2.66 to 5.23)	4.39(3.24 to 5.71)	0.14(-0.08 to 0.44)
Brunei Darussalam	Liver cancer due to NASH	0(0 to 1)	2(1 to 3)	3.1(2.12 to 4.42)	0.57(0.37 to 0.85)	0.71(0.47 to 1.04)	0.25(-0.03 to 0.62)
Brunei Darussalam	Liver cancer due to other causes	0(0 to 1)	1(1 to 2)	2.55(1.73 to 3.6)	0.39(0.25 to 0.58)	0.47(0.3 to 0.66)	0.19(-0.08 to 0.53)
Bulgaria	Liver cancer	807(757 to 861)	646(516 to 794)	-0.2(-0.36 to 0)	6.43(6.04 to 6.86)	4.62(3.68 to 5.71)	-0.28(-0.43 to -0.1)
Bulgaria	Liver cancer due to alcohol use	334(254 to 413)	295(213 to 398)	-0.12(-0.31 to 0.13)	2.59(2 to 3.19)	2.08(1.49 to 2.81)	-0.2(-0.38 to 0.03)
Bulgaria	Liver cancer due to hepatitis B	198(140 to 270)	126(83 to 189)	-0.36(-0.51 to -0.18)	1.6(1.16 to 2.15)	1(0.67 to 1.47)	-0.38(-0.52 to -0.21)

Bulgaria	Liver cancer due to hepatitis C	191(129 to 260)	155(103 to 227)	-0.19(-0.36 to 0.02)	1.55(1.09 to 2.05)	1.05(0.69 to 1.51)	-0.33(-0.46 to -0.16)
Bulgaria	Liver cancer due to NASH	57(39 to 81)	50(33 to 74)	-0.12(-0.32 to 0.15)	0.46(0.32 to 0.65)	0.35(0.23 to 0.5)	-0.25(-0.43 to -0.05)
Bulgaria	Liver cancer due to other causes	26(18 to 37)	19(12 to 28)	-0.29(-0.46 to -0.06)	0.23(0.16 to 0.31)	0.16(0.1 to 0.22)	-0.31(-0.47 to -0.11)
Burkina Faso	Liver cancer	153(121 to 189)	248(189 to 317)	0.62(0.2 to 1.15)	3.25(2.63 to 3.93)	2.37(1.8 to 2.96)	-0.27(-0.47 to -0.03)
Burkina Faso	Liver cancer due to alcohol use	20(12 to 29)	34(21 to 49)	0.7(0.16 to 1.42)	0.48(0.3 to 0.69)	0.4(0.25 to 0.58)	-0.18(-0.43 to 0.17)
Burkina Faso	Liver cancer due to hepatitis B	59(42 to 79)	89(62 to 122)	0.5(0.05 to 1.08)	1.31(0.93 to 1.74)	0.87(0.6 to 1.19)	-0.34(-0.54 to -0.1)
Burkina Faso	Liver cancer due to hepatitis C	36(24 to 49)	51(34 to 71)	0.44(0.04 to 0.98)	0.99(0.69 to 1.33)	0.68(0.45 to 0.94)	-0.31(-0.5 to -0.07)
Burkina Faso	Liver cancer due to NASH	9(6 to 13)	18(12 to 26)	1.05(0.48 to 1.83)	0.22(0.15 to 0.33)	0.21(0.14 to 0.31)	-0.05(-0.3 to 0.29)
Burkina Faso	Liver cancer due to other causes	30(18 to 47)	56(35 to 88)	0.88(0.04 to 2.23)	0.25(0.17 to 0.34)	0.22(0.15 to 0.3)	-0.12(-0.41 to 0.28)
Burundi	Liver cancer	97(71 to 129)	144(99 to 225)	0.48(-0.06 to 1.22)	3.94(2.93 to 5.15)	3.11(2.17 to 4.8)	-0.21(-0.48 to 0.15)
Burundi	Liver cancer due to alcohol use	30(19 to 44)	39(21 to 69)	0.31(-0.19 to 0.99)	1.29(0.83 to 1.91)	0.89(0.51 to 1.58)	-0.3(-0.56 to 0.04)
Burundi	Liver cancer due to hepatitis B	26(17 to 37)	42(25 to 71)	0.61(-0.02 to 1.54)	1.02(0.67 to 1.45)	0.79(0.46 to 1.33)	-0.22(-0.52 to 0.21)
Burundi	Liver cancer due to hepatitis C	19(11 to 29)	30(18 to 46)	0.57(0.04 to 1.38)	0.91(0.57 to 1.34)	0.81(0.5 to 1.22)	-0.11(-0.39 to 0.29)
Burundi	Liver cancer due to NASH	9(6 to 14)	15(9 to 25)	0.62(0.03 to 1.42)	0.42(0.27 to 0.65)	0.37(0.23 to 0.6)	-0.12(-0.42 to 0.32)

Burundi	Liver cancer due to other causes	13(8 to 21)	19(13 to 28)	0.4(-0.2 to 1.37)	0.31(0.2 to 0.46)	0.24(0.16 to 0.38)	-0.21(-0.52 to 0.18)
C??te d'Ivoire	Liver cancer	331(249 to 426)	537(388 to 742)	0.62(0.17 to 1.19)	7.93(6.08 to 9.91)	5.06(3.77 to 6.83)	-0.36(-0.53 to -0.15)
C??te d'Ivoire	Liver cancer due to alcohol use	58(35 to 89)	105(64 to 165)	0.81(0.28 to 1.54)	1.55(0.94 to 2.36)	1.08(0.67 to 1.67)	-0.3(-0.5 to -0.05)
C??te d'Ivoire	Liver cancer due to hepatitis B	184(129 to 245)	271(182 to 385)	0.48(0.02 to 1.07)	4.05(2.9 to 5.43)	2.28(1.55 to 3.27)	-0.44(-0.59 to -0.24)
C??te d'Ivoire	Liver cancer due to hepatitis C	43(27 to 64)	79(48 to 118)	0.82(0.33 to 1.41)	1.39(0.9 to 2)	0.96(0.62 to 1.42)	-0.31(-0.48 to -0.1)
C??te d'Ivoire	Liver cancer due to NASH	20(13 to 30)	45(29 to 69)	1.21(0.57 to 2.03)	0.58(0.37 to 0.87)	0.49(0.32 to 0.76)	-0.16(-0.39 to 0.12)
C??te d'Ivoire	Liver cancer due to other causes	26(18 to 36)	37(24 to 53)	0.43(-0.02 to 0.99)	0.35(0.24 to 0.5)	0.25(0.16 to 0.37)	-0.3(-0.49 to -0.06)
Cabo Verde	Liver cancer	3(2 to 3)	53(44 to 64)	17.87(14.02 to 22.97)	1.17(1.01 to 1.35)	12.34(10.2 to 14.87)	9.52(7.42 to 12.43)
Cabo Verde	Liver cancer due to alcohol use	0(0 to 1)	10(7 to 14)	20.6(15.66 to 27.26)	0.2(0.13 to 0.28)	2.4(1.62 to 3.32)	11.32(8.64 to 14.92)
Cabo Verde	Liver cancer due to hepatitis B	1(1 to 2)	24(19 to 31)	18.46(14.15 to 24.34)	0.54(0.41 to 0.69)	5.36(4.04 to 6.98)	8.98(6.86 to 11.76)
Cabo Verde	Liver cancer due to hepatitis C	1(0 to 1)	10(7 to 14)	14.89(11.24 to 20.66)	0.27(0.18 to 0.37)	2.56(1.68 to 3.63)	8.5(6.26 to 11.94)
Cabo Verde	Liver cancer due to NASH	0(0 to 0)	6(4 to 8)	21.66(15.96 to 29.65)	0.11(0.07 to 0.15)	1.41(0.96 to 2.09)	12.41(9.03 to 17.11)
Cabo Verde	Liver cancer due to other causes	0(0 to 0)	3(2 to 4)	12.57(9.34 to 17.38)	0.06(0.05 to 0.09)	0.6(0.41 to 0.86)	8.19(5.99 to 11.46)
Cambodia	Liver cancer	676(523 to 899)	1184(925 to 1462)	0.75(0.15 to 1.55)	11.62(9.34 to 13.98)	9.86(7.85 to 12.03)	-0.15(-0.38 to 0.19)

Cambodia	Liver cancer due to alcohol use	55(35 to 83)	187(119 to 272)	2.41(1.44 to 3.79)	1.25(0.8 to 1.91)	1.61(1.03 to 2.32)	0.29(-0.07 to 0.81)
Cambodia	Liver cancer due to hepatitis B	225(168 to 297)	389(274 to 545)	0.73(0.21 to 1.41)	3.95(2.89 to 5.31)	2.95(2.05 to 4.14)	-0.25(-0.47 to 0.03)
Cambodia	Liver cancer due to hepatitis C	188(131 to 250)	415(296 to 550)	1.2(0.57 to 2.22)	4.67(3.32 to 6.02)	3.92(2.84 to 5.08)	-0.16(-0.39 to 0.2)
Cambodia	Liver cancer due to NASH	30(20 to 43)	75(49 to 110)	1.5(0.74 to 2.63)	0.64(0.42 to 0.93)	0.66(0.43 to 0.97)	0.02(-0.25 to 0.49)
Cambodia	Liver cancer due to other causes	178(85 to 357)	118(68 to 201)	-0.34(-0.74 to 0.6)	1.11(0.62 to 2)	0.73(0.44 to 1.19)	-0.34(-0.72 to 0.36)
Cameroon	Liver cancer	31(21 to 46)	85(61 to 115)	1.77(0.76 to 3.09)	0.69(0.48 to 1.04)	0.69(0.51 to 0.92)	0(-0.35 to 0.46)
Cameroon	Liver cancer due to alcohol use	5(3 to 10)	17(10 to 26)	2.05(0.86 to 3.65)	0.13(0.07 to 0.23)	0.15(0.09 to 0.23)	0.13(-0.3 to 0.7)
Cameroon	Liver cancer due to hepatitis B	16(10 to 25)	42(28 to 60)	1.67(0.61 to 3.09)	0.33(0.21 to 0.52)	0.31(0.21 to 0.44)	-0.06(-0.42 to 0.41)
Cameroon	Liver cancer due to hepatitis C	5(3 to 8)	12(7 to 18)	1.46(0.65 to 2.51)	0.13(0.08 to 0.2)	0.12(0.08 to 0.18)	-0.07(-0.36 to 0.32)
Cameroon	Liver cancer due to NASH	3(2 to 4)	8(5 to 12)	2.1(1.01 to 3.51)	0.06(0.04 to 0.1)	0.07(0.05 to 0.11)	0.14(-0.24 to 0.65)
Cameroon	Liver cancer due to other causes	2(1 to 3)	6(4 to 9)	2.11(1.07 to 3.45)	0.03(0.02 to 0.05)	0.04(0.02 to 0.05)	0.09(-0.29 to 0.55)
Canada	Liver cancer	618(591 to 642)	2668(2374 to 2952)	3.31(2.85 to 3.76)	1.92(1.83 to 1.99)	3.91(3.48 to 4.32)	1.04(0.83 to 1.25)
Canada	Liver cancer due to alcohol use	350(299 to 398)	1477(1184 to 1748)	3.23(2.67 to 3.75)	1.07(0.92 to 1.22)	2.17(1.75 to 2.56)	1.02(0.76 to 1.27)
Canada	Liver cancer due to hepatitis B	49(34 to 71)	171(112 to 257)	2.51(1.98 to 3.08)	0.15(0.11 to 0.22)	0.27(0.18 to 0.4)	0.76(0.53 to 1.01)

Canada	Liver cancer due to hepatitis C	114(80 to 155)	521(356 to 734)	3.58(3.04 to 4.2)	0.35(0.25 to 0.48)	0.73(0.5 to 1.02)	1.07(0.84 to 1.32)
Canada	Liver cancer due to NASH	66(47 to 93)	355(247 to 499)	4.36(3.57 to 5.2)	0.2(0.15 to 0.28)	0.5(0.36 to 0.7)	1.46(1.12 to 1.83)
Canada	Liver cancer due to other causes	40(30 to 53)	143(101 to 203)	2.59(1.98 to 3.24)	0.13(0.1 to 0.17)	0.24(0.17 to 0.32)	0.77(0.53 to 1.06)
Central African Republic	Liver cancer	48(33 to 67)	75(48 to 113)	0.57(0.15 to 1.11)	3.82(2.71 to 5.18)	3.42(2.27 to 5.01)	-0.1(-0.32 to 0.19)
Central African Republic	Liver cancer due to alcohol use	7(4 to 11)	11(6 to 20)	0.58(0.14 to 1.25)	0.61(0.36 to 0.94)	0.54(0.3 to 0.92)	-0.12(-0.36 to 0.25)
Central African Republic	Liver cancer due to hepatitis B	14(9 to 21)	22(12 to 36)	0.53(0.08 to 1.13)	1.01(0.63 to 1.51)	0.82(0.47 to 1.35)	-0.19(-0.4 to 0.1)
Central African Republic	Liver cancer due to hepatitis C	18(11 to 26)	29(17 to 44)	0.66(0.2 to 1.29)	1.72(1.13 to 2.46)	1.62(1.01 to 2.37)	-0.06(-0.29 to 0.26)
Central African Republic	Liver cancer due to NASH	3(2 to 4)	5(3 to 8)	0.82(0.31 to 1.54)	0.24(0.14 to 0.37)	0.24(0.14 to 0.39)	0.01(-0.27 to 0.38)
Central African Republic	Liver cancer due to other causes	7(4 to 11)	9(5 to 14)	0.3(-0.19 to 1.06)	0.24(0.15 to 0.36)	0.2(0.12 to 0.32)	-0.17(-0.42 to 0.18)
Chad	Liver cancer	190(148 to 243)	325(246 to 421)	0.71(0.3 to 1.27)	6.54(5.05 to 8.35)	5.68(4.37 to 7.29)	-0.13(-0.33 to 0.15)
Chad	Liver cancer due to alcohol use	27(16 to 43)	48(29 to 75)	0.77(0.28 to 1.45)	0.98(0.58 to 1.52)	0.92(0.56 to 1.41)	-0.05(-0.31 to 0.31)
Chad	Liver cancer due to hepatitis B	97(68 to 132)	167(117 to 226)	0.71(0.26 to 1.32)	3.31(2.32 to 4.49)	2.76(1.93 to 3.79)	-0.17(-0.37 to 0.12)
Chad	Liver cancer due to hepatitis C	37(23 to 53)	57(36 to 83)	0.54(0.18 to 1.07)	1.42(0.91 to 2.01)	1.21(0.77 to 1.72)	-0.15(-0.34 to 0.12)

Chad	Liver cancer due to NASH	13(9 to 20)	25(16 to 37)	0.86(0.38 to 1.47)	0.49(0.31 to 0.72)	0.48(0.31 to 0.72)	-0.01(-0.26 to 0.3)
Chad	Liver cancer due to other causes	15(10 to 21)	28(19 to 38)	0.86(0.34 to 1.61)	0.34(0.23 to 0.47)	0.3(0.2 to 0.43)	-0.11(-0.32 to 0.18)
Chile	Liver cancer	218(204 to 235)	727(664 to 791)	2.33(1.97 to 2.72)	2.21(2.06 to 2.37)	3.02(2.76 to 3.29)	0.37(0.22 to 0.53)
Chile	Liver cancer due to alcohol use	69(50 to 89)	228(164 to 296)	2.33(1.87 to 2.89)	0.69(0.5 to 0.9)	0.94(0.68 to 1.22)	0.37(0.19 to 0.59)
Chile	Liver cancer due to hepatitis B	44(31 to 61)	114(77 to 166)	1.63(1.23 to 2.04)	0.42(0.29 to 0.59)	0.48(0.33 to 0.69)	0.13(-0.02 to 0.31)
Chile	Liver cancer due to hepatitis C	73(53 to 93)	266(192 to 344)	2.63(2.19 to 3.14)	0.77(0.57 to 0.98)	1.1(0.8 to 1.42)	0.42(0.26 to 0.61)
Chile	Liver cancer due to NASH	17(12 to 24)	77(53 to 110)	3.47(2.77 to 4.22)	0.18(0.13 to 0.26)	0.32(0.22 to 0.45)	0.78(0.52 to 1.07)
Chile	Liver cancer due to other causes	16(12 to 21)	41(29 to 58)	1.59(1.14 to 2.11)	0.15(0.11 to 0.2)	0.18(0.13 to 0.25)	0.24(0.05 to 0.46)
China	Liver cancer	232449(197395 to 275388)	187700(158262 to 222767)	-0.19(-0.37 to 0.05)	25.99(22.29 to 30.55)	9.41(7.95 to 11.13)	-0.64(-0.71 to -0.53)
China	Liver cancer due to alcohol use	17526(13039 to 23669)	17436(12814 to 22545)	-0.01(-0.23 to 0.29)	1.99(1.49 to 2.67)	0.85(0.63 to 1.09)	-0.57(-0.67 to -0.45)
China	Liver cancer due to hepatitis B	152614(126398 to 183861)	116998(94646 to 142974)	-0.23(-0.42 to 0.03)	16.22(13.47 to 19.53)	5.76(4.68 to 7.02)	-0.64(-0.73 to -0.53)
China	Liver cancer due to hepatitis C	37163(30837 to 45223)	33079(27212 to 39258)	-0.11(-0.29 to 0.12)	4.99(4.19 to 5.99)	1.75(1.45 to 2.07)	-0.65(-0.72 to -0.56)
China	Liver cancer due to NASH	8866(7044 to 11080)	9401(7549 to 11640)	0.06(-0.16 to 0.34)	1.06(0.85 to 1.31)	0.48(0.39 to 0.59)	-0.54(-0.63 to -0.43)
China	Liver cancer due to other causes	16281(13227 to 20051)	10786(8661 to 13163)	-0.34(-0.47 to -0.16)	1.73(1.41 to 2.13)	0.57(0.46 to 0.69)	-0.67(-0.74 to -0.59)

Colombia	Liver cancer	523(495 to 549)	1396(1071 to 1789)	1.67(1.06 to 2.42)	3.02(2.83 to 3.18)	2.65(2.03 to 3.4)	-0.12(-0.32 to 0.13)
Colombia	Liver cancer due to alcohol use	148(108 to 190)	440(292 to 632)	1.97(1.19 to 2.91)	0.87(0.64 to 1.12)	0.84(0.56 to 1.21)	-0.04(-0.29 to 0.25)
Colombia	Liver cancer due to hepatitis B	111(83 to 148)	231(147 to 347)	1.08(0.54 to 1.74)	0.57(0.42 to 0.79)	0.44(0.28 to 0.66)	-0.24(-0.43 to 0)
Colombia	Liver cancer due to hepatitis C	190(144 to 234)	541(372 to 750)	1.85(1.21 to 2.67)	1.2(0.92 to 1.47)	1.02(0.69 to 1.41)	-0.16(-0.35 to 0.09)
Colombia	Liver cancer due to NASH	31(22 to 44)	110(70 to 168)	2.53(1.62 to 3.72)	0.19(0.13 to 0.27)	0.21(0.13 to 0.32)	0.11(-0.17 to 0.46)
Colombia	Liver cancer due to other causes	42(34 to 52)	73(49 to 105)	0.74(0.28 to 1.31)	0.18(0.14 to 0.23)	0.15(0.1 to 0.21)	-0.17(-0.37 to 0.09)
Comoros	Liver cancer	8(4 to 13)	14(10 to 23)	0.92(0.31 to 2.49)	3.31(1.87 to 5.51)	3.01(2.12 to 4.65)	-0.09(-0.35 to 0.55)
Comoros	Liver cancer due to alcohol use	1(1 to 3)	3(2 to 6)	0.92(0.31 to 2.36)	0.68(0.34 to 1.42)	0.61(0.34 to 1.16)	-0.11(-0.39 to 0.49)
Comoros	Liver cancer due to hepatitis B	2(1 to 4)	4(2 to 8)	0.81(0.19 to 2.91)	1.02(0.48 to 1.87)	0.84(0.49 to 1.44)	-0.18(-0.45 to 0.63)
Comoros	Liver cancer due to hepatitis C	2(1 to 3)	4(2 to 6)	1.12(0.5 to 2.38)	0.89(0.52 to 1.42)	0.86(0.54 to 1.31)	-0.04(-0.31 to 0.43)
Comoros	Liver cancer due to NASH	1(0 to 2)	2(1 to 3)	1.3(0.57 to 2.9)	0.44(0.25 to 0.75)	0.46(0.28 to 0.74)	0.05(-0.26 to 0.7)
Comoros	Liver cancer due to other causes	1(0 to 1)	1(1 to 2)	0.43(-0.12 to 1.83)	0.28(0.15 to 0.45)	0.25(0.16 to 0.38)	-0.11(-0.4 to 0.61)
Congo	Liver cancer	47(35 to 64)	79(56 to 108)	0.66(0.21 to 1.34)	4.28(3.25 to 5.7)	3.14(2.33 to 4.24)	-0.27(-0.45 to 0)
Congo	Liver cancer due to alcohol use	7(4 to 11)	13(8 to 20)	0.88(0.33 to 1.83)	0.65(0.38 to 1.01)	0.52(0.32 to 0.79)	-0.2(-0.42 to 0.17)

Congo	Liver cancer due to hepatitis B	13(8 to 19)	20(13 to 31)	0.6(0.1 to 1.37)	1.01(0.64 to 1.55)	0.66(0.42 to 1)	-0.35(-0.53 to -0.06)
Congo	Liver cancer due to hepatitis C	20(13 to 28)	33(22 to 49)	0.71(0.25 to 1.36)	2.09(1.47 to 2.88)	1.55(1.08 to 2.19)	-0.26(-0.45 to -0.01)
Congo	Liver cancer due to NASH	3(2 to 4)	6(4 to 9)	1.12(0.48 to 2.01)	0.28(0.18 to 0.43)	0.26(0.16 to 0.39)	-0.1(-0.36 to 0.23)
Congo	Liver cancer due to other causes	6(3 to 8)	6(4 to 9)	0.13(-0.3 to 0.76)	0.24(0.17 to 0.34)	0.16(0.1 to 0.23)	-0.35(-0.54 to -0.08)
Cook Islands	Liver cancer	2(1 to 2)	3(2 to 3)	0.57(0.22 to 1.02)	13.6(11.34 to 15.98)	11.13(9.03 to 13.51)	-0.18(-0.36 to 0.04)
Cook Islands	Liver cancer due to alcohol use	0(0 to 0)	0(0 to 1)	1.23(0.66 to 1.99)	1.61(1 to 2.33)	1.82(1.17 to 2.63)	0.13(-0.14 to 0.5)
Cook Islands	Liver cancer due to hepatitis B	1(1 to 1)	1(1 to 2)	0.35(0.01 to 0.81)	6.88(5.16 to 8.98)	5.18(3.82 to 6.86)	-0.25(-0.44 to 0.01)
Cook Islands	Liver cancer due to hepatitis C	0(0 to 1)	1(0 to 1)	0.6(0.26 to 1.05)	3.24(2.16 to 4.38)	2.39(1.61 to 3.34)	-0.26(-0.41 to -0.07)
Cook Islands	Liver cancer due to NASH	0(0 to 0)	0(0 to 0)	1.13(0.63 to 1.79)	1.28(0.88 to 1.81)	1.34(0.91 to 1.87)	0.05(-0.19 to 0.37)
Cook Islands	Liver cancer due to other causes	0(0 to 0)	0(0 to 0)	0.2(-0.1 to 0.6)	0.58(0.39 to 0.82)	0.41(0.26 to 0.59)	-0.3(-0.47 to -0.08)
Costa Rica	Liver cancer	100(92 to 106)	269(208 to 341)	1.7(1.08 to 2.45)	5.74(5.29 to 6.13)	5.3(4.11 to 6.74)	-0.08(-0.29 to 0.18)
Costa Rica	Liver cancer due to alcohol use	33(24 to 42)	93(62 to 131)	1.8(1.14 to 2.67)	1.93(1.41 to 2.47)	1.82(1.24 to 2.58)	-0.06(-0.28 to 0.23)
Costa Rica	Liver cancer due to hepatitis B	17(12 to 23)	38(25 to 59)	1.31(0.75 to 2.01)	0.89(0.63 to 1.24)	0.74(0.47 to 1.14)	-0.17(-0.36 to 0.09)
Costa Rica	Liver cancer due to hepatitis C	37(27 to 46)	100(68 to 137)	1.74(1.13 to 2.53)	2.21(1.66 to 2.8)	1.99(1.35 to 2.72)	-0.1(-0.3 to 0.17)

Costa Rica	Liver cancer due to NASH	7(5 to 10)	24(15 to 37)	2.46(1.61 to 3.51)	0.41(0.28 to 0.57)	0.48(0.31 to 0.72)	0.18(-0.12 to 0.54)
Costa Rica	Liver cancer due to other causes	6(5 to 8)	13(9 to 19)	1.12(0.56 to 1.81)	0.3(0.22 to 0.41)	0.26(0.17 to 0.39)	-0.13(-0.34 to 0.12)
Croatia	Liver cancer	204(189 to 221)	310(245 to 391)	0.52(0.18 to 0.95)	3.22(2.98 to 3.47)	3.53(2.77 to 4.48)	0.1(-0.15 to 0.41)
Croatia	Liver cancer due to alcohol use	89(69 to 108)	146(103 to 197)	0.64(0.26 to 1.15)	1.38(1.08 to 1.69)	1.66(1.17 to 2.22)	0.2(-0.09 to 0.56)
Croatia	Liver cancer due to hepatitis B	47(33 to 64)	61(39 to 90)	0.29(-0.03 to 0.7)	0.73(0.52 to 0.98)	0.74(0.49 to 1.11)	0.02(-0.23 to 0.34)
Croatia	Liver cancer due to hepatitis C	42(29 to 58)	62(40 to 89)	0.46(0.14 to 0.88)	0.69(0.47 to 0.93)	0.67(0.43 to 0.96)	-0.03(-0.24 to 0.23)
Croatia	Liver cancer due to NASH	18(12 to 25)	31(20 to 46)	0.75(0.32 to 1.31)	0.29(0.2 to 0.4)	0.34(0.22 to 0.5)	0.17(-0.11 to 0.53)
Croatia	Liver cancer due to other causes	8(6 to 12)	11(7 to 16)	0.27(-0.03 to 0.66)	0.14(0.1 to 0.19)	0.13(0.09 to 0.19)	-0.05(-0.26 to 0.22)
Cuba	Liver cancer	695(655 to 728)	464(375 to 573)	-0.33(-0.46 to -0.19)	6.71(6.31 to 7.03)	2.43(1.95 to 3)	-0.64(-0.71 to -0.56)
Cuba	Liver cancer due to alcohol use	227(169 to 289)	173(119 to 236)	-0.24(-0.4 to -0.05)	2.18(1.63 to 2.77)	0.9(0.62 to 1.23)	-0.59(-0.68 to -0.48)
Cuba	Liver cancer due to hepatitis B	187(138 to 250)	107(72 to 155)	-0.43(-0.55 to -0.29)	1.81(1.33 to 2.4)	0.58(0.39 to 0.83)	-0.68(-0.75 to -0.61)
Cuba	Liver cancer due to hepatitis C	175(125 to 233)	111(74 to 158)	-0.36(-0.49 to -0.22)	1.7(1.2 to 2.23)	0.56(0.37 to 0.8)	-0.67(-0.73 to -0.59)
Cuba	Liver cancer due to NASH	61(43 to 85)	49(32 to 74)	-0.21(-0.38 to 0.02)	0.59(0.41 to 0.82)	0.25(0.16 to 0.38)	-0.57(-0.67 to -0.45)
Cuba	Liver cancer due to other causes	44(32 to 60)	23(15 to 34)	-0.48(-0.6 to -0.33)	0.43(0.31 to 0.58)	0.13(0.09 to 0.19)	-0.69(-0.75 to -0.61)

Cyprus	Liver cancer	26(22 to 31)	65(56 to 75)	1.47(0.99 to 2.14)	3.4(2.87 to 3.9)	3.42(2.95 to 3.94)	0.01(-0.17 to 0.25)
Cyprus	Liver cancer due to alcohol use	10(7 to 13)	24(18 to 32)	1.49(0.94 to 2.33)	1.18(0.84 to 1.59)	1.24(0.9 to 1.61)	0.05(-0.18 to 0.37)
Cyprus	Liver cancer due to hepatitis B	4(2 to 5)	7(5 to 11)	0.97(0.53 to 1.53)	0.46(0.31 to 0.67)	0.39(0.26 to 0.57)	-0.16(-0.34 to 0.07)
Cyprus	Liver cancer due to hepatitis C	10(7 to 13)	26(19 to 33)	1.61(1.09 to 2.43)	1.38(1 to 1.78)	1.4(1.05 to 1.79)	0.02(-0.17 to 0.29)
Cyprus	Liver cancer due to NASH	1(1 to 2)	4(3 to 6)	1.91(1.23 to 2.92)	0.19(0.13 to 0.28)	0.23(0.15 to 0.33)	0.16(-0.09 to 0.5)
Cyprus	Liver cancer due to other causes	1(1 to 2)	3(2 to 4)	1.14(0.68 to 1.76)	0.18(0.12 to 0.25)	0.17(0.11 to 0.24)	-0.07(-0.26 to 0.18)
Czechia	Liver cancer	627(594 to 655)	632(522 to 777)	0.01(-0.18 to 0.26)	4.52(4.29 to 4.73)	2.99(2.45 to 3.68)	-0.34(-0.46 to -0.17)
Czechia	Liver cancer due to alcohol use	289(231 to 344)	309(229 to 403)	0.07(-0.15 to 0.34)	2.06(1.65 to 2.45)	1.44(1.07 to 1.88)	-0.3(-0.44 to -0.12)
Czechia	Liver cancer due to hepatitis B	132(93 to 184)	107(70 to 161)	-0.19(-0.38 to 0.03)	0.98(0.7 to 1.34)	0.54(0.36 to 0.8)	-0.44(-0.57 to -0.29)
Czechia	Liver cancer due to hepatitis C	145(100 to 195)	151(101 to 216)	0.04(-0.18 to 0.3)	1.04(0.73 to 1.38)	0.69(0.45 to 0.98)	-0.34(-0.47 to -0.19)
Czechia	Liver cancer due to NASH	41(28 to 59)	48(31 to 72)	0.18(-0.07 to 0.49)	0.29(0.2 to 0.42)	0.22(0.15 to 0.33)	-0.24(-0.4 to -0.04)
Czechia	Liver cancer due to other causes	19(13 to 27)	17(11 to 25)	-0.11(-0.31 to 0.14)	0.15(0.11 to 0.21)	0.09(0.06 to 0.13)	-0.38(-0.5 to -0.2)
Democratic People's Republic of Korea	Liver cancer	2531(1944 to 3304)	3313(2499 to 4332)	0.31(-0.11 to 0.81)	14.77(11.51 to 19.02)	10.2(7.75 to 13.3)	-0.31(-0.52 to -0.06)

Democratic People's Republic of Korea	Liver cancer due to alcohol use	181(107 to 295)	271(156 to 435)	0.49(0.01 to 1.13)	1.07(0.65 to 1.7)	0.83(0.48 to 1.31)	-0.23(-0.48 to 0.08)
Democratic People's Republic of Korea	Liver cancer due to hepatitis B	1576(1140 to 2156)	1936(1347 to 2687)	0.23(-0.2 to 0.8)	8.58(6.22 to 11.52)	5.84(4.08 to 8.1)	-0.32(-0.55 to -0.02)
Democratic People's Republic of Korea	Liver cancer due to hepatitis C	495(312 to 713)	756(492 to 1071)	0.53(0.11 to 1.07)	3.49(2.26 to 4.87)	2.44(1.6 to 3.41)	-0.3(-0.49 to -0.07)
Democratic People's Republic of Korea	Liver cancer due to NASH	110(70 to 169)	162(100 to 250)	0.47(0.01 to 1.03)	0.71(0.45 to 1.08)	0.51(0.31 to 0.79)	-0.29(-0.5 to -0.04)
Democratic People's Republic of Korea	Liver cancer due to other causes	169(108 to 245)	188(116 to 279)	0.11(-0.24 to 0.58)	0.92(0.6 to 1.32)	0.59(0.38 to 0.86)	-0.36(-0.55 to -0.09)
Democratic Republic of the Congo	Liver cancer	477(385 to 586)	887(676 to 1161)	0.86(0.31 to 1.6)	2.67(2.2 to 3.18)	2.28(1.77 to 2.94)	-0.14(-0.37 to 0.19)
Democratic Republic of the Congo	Liver cancer due to alcohol use	53(34 to 78)	104(62 to 158)	0.97(0.37 to 1.87)	0.35(0.23 to 0.51)	0.29(0.18 to 0.44)	-0.16(-0.41 to 0.18)
Democratic Republic of the Congo	Liver cancer due to hepatitis B	116(79 to 162)	225(147 to 337)	0.94(0.33 to 1.83)	0.62(0.42 to 0.87)	0.49(0.32 to 0.76)	-0.2(-0.43 to 0.14)

Democratic Republic of the Congo	Liver cancer due to hepatitis C	178(129 to 233)	366(257 to 498)	1.06(0.47 to 1.93)	1.33(1.01 to 1.68)	1.17(0.85 to 1.55)	-0.11(-0.34 to 0.22)
Democratic Republic of the Congo	Liver cancer due to NASH	24(16 to 35)	55(36 to 86)	1.32(0.61 to 2.35)	0.16(0.11 to 0.24)	0.16(0.1 to 0.24)	-0.01(-0.28 to 0.37)
Democratic Republic of the Congo	Liver cancer due to other causes	107(68 to 169)	137(71 to 240)	0.28(-0.38 to 1.5)	0.21(0.15 to 0.3)	0.16(0.1 to 0.25)	-0.23(-0.53 to 0.22)
Denmark	Liver cancer	155(147 to 163)	370(333 to 407)	1.39(1.14 to 1.67)	1.91(1.82 to 2.01)	3.25(2.93 to 3.57)	0.7(0.52 to 0.89)
Denmark	Liver cancer due to alcohol use	63(48 to 78)	147(109 to 187)	1.34(1.01 to 1.7)	0.77(0.59 to 0.95)	1.28(0.95 to 1.63)	0.66(0.43 to 0.9)
Denmark	Liver cancer due to hepatitis B	19(13 to 28)	47(31 to 69)	1.42(1.11 to 1.78)	0.26(0.18 to 0.36)	0.45(0.3 to 0.65)	0.76(0.53 to 1.01)
Denmark	Liver cancer due to hepatitis C	57(42 to 73)	137(100 to 177)	1.4(1.13 to 1.7)	0.68(0.5 to 0.86)	1.15(0.85 to 1.49)	0.7(0.51 to 0.91)
Denmark	Liver cancer due to NASH	9(6 to 13)	24(16 to 35)	1.66(1.28 to 2.15)	0.11(0.07 to 0.15)	0.2(0.14 to 0.29)	0.9(0.64 to 1.24)
Denmark	Liver cancer due to other causes	7(5 to 11)	17(11 to 24)	1.23(0.88 to 1.59)	0.1(0.08 to 0.14)	0.16(0.12 to 0.23)	0.57(0.33 to 0.81)
Djibouti	Liver cancer	5(3 to 9)	20(13 to 34)	2.97(1.73 to 4.7)	3.37(2.23 to 5.79)	3.49(2.29 to 5.59)	0.04(-0.25 to 0.42)
Djibouti	Liver cancer due to alcohol use	1(1 to 2)	5(2 to 9)	3.46(2.02 to 5.56)	0.79(0.4 to 1.68)	0.84(0.46 to 1.62)	0.07(-0.25 to 0.53)
Djibouti	Liver cancer due to hepatitis B	2(1 to 4)	7(4 to 12)	2.85(1.55 to 4.93)	1.01(0.55 to 1.97)	0.98(0.55 to 1.74)	-0.03(-0.32 to 0.39)
Djibouti	Liver cancer due to hepatitis C	1(1 to 2)	4(2 to 7)	3.41(2.13 to 5.2)	0.89(0.55 to 1.44)	0.92(0.57 to 1.47)	0.04(-0.24 to 0.38)

Djibouti	Liver cancer due to NASH	0(0 to 1)	2(1 to 4)	3.91(2.39 to 6.13)	0.4(0.22 to 0.7)	0.48(0.28 to 0.8)	0.18(-0.15 to 0.64)
Djibouti	Liver cancer due to other causes	1(1 to 1)	2(1 to 3)	1.56(0.49 to 3.09)	0.28(0.17 to 0.45)	0.27(0.16 to 0.43)	-0.04(-0.34 to 0.39)
Dominica	Liver cancer	7(6 to 7)	3(2 to 4)	-0.54(-0.64 to -0.42)	9.13(8.01 to 10.31)	3.39(2.76 to 4.16)	-0.63(-0.71 to -0.53)
Dominica	Liver cancer due to alcohol use	2(2 to 3)	1(1 to 2)	-0.5(-0.61 to -0.35)	2.99(2.15 to 3.9)	1.22(0.84 to 1.7)	-0.59(-0.68 to -0.47)
Dominica	Liver cancer due to hepatitis B	2(1 to 2)	1(0 to 1)	-0.56(-0.66 to -0.43)	2.47(1.78 to 3.36)	0.84(0.56 to 1.19)	-0.66(-0.74 to -0.56)
Dominica	Liver cancer due to hepatitis C	2(1 to 2)	1(0 to 1)	-0.58(-0.67 to -0.46)	2.28(1.63 to 3.09)	0.79(0.52 to 1.11)	-0.66(-0.73 to -0.56)
Dominica	Liver cancer due to NASH	1(0 to 1)	0(0 to 0)	-0.48(-0.6 to -0.31)	0.78(0.54 to 1.1)	0.34(0.23 to 0.49)	-0.57(-0.67 to -0.43)
Dominica	Liver cancer due to other causes	0(0 to 1)	0(0 to 0)	-0.6(-0.7 to -0.47)	0.6(0.42 to 0.82)	0.2(0.14 to 0.29)	-0.66(-0.74 to -0.55)
Dominican Republic	Liver cancer	159(139 to 181)	453(310 to 678)	1.84(0.87 to 3.37)	4.16(3.61 to 4.73)	4.92(3.4 to 7.23)	0.18(-0.21 to 0.82)
Dominican Republic	Liver cancer due to alcohol use	49(36 to 64)	158(95 to 265)	2.21(1.04 to 4.11)	1.35(0.98 to 1.77)	1.73(1.04 to 2.87)	0.28(-0.18 to 1.05)
Dominican Republic	Liver cancer due to hepatitis B	49(37 to 64)	125(74 to 209)	1.54(0.6 to 3.15)	1.18(0.87 to 1.57)	1.3(0.77 to 2.15)	0.1(-0.3 to 0.78)
Dominican Republic	Liver cancer due to hepatitis C	33(23 to 44)	98(62 to 152)	1.96(0.98 to 3.3)	1(0.72 to 1.33)	1.12(0.72 to 1.72)	0.12(-0.24 to 0.6)
Dominican Republic	Liver cancer due to NASH	11(8 to 16)	41(25 to 63)	2.65(1.4 to 4.37)	0.31(0.21 to 0.44)	0.45(0.28 to 0.7)	0.46(-0.03 to 1.14)
Dominican Republic	Liver cancer due to other causes	16(13 to 20)	31(21 to 46)	0.89(0.3 to 1.69)	0.32(0.24 to 0.42)	0.32(0.21 to 0.49)	0.01(-0.29 to 0.42)

Ecuador	Liver cancer	162(145 to 179)	539(430 to 692)	2.33(1.55 to 3.32)	3.03(2.69 to 3.38)	3.71(2.97 to 4.73)	0.22(-0.06 to 0.57)
Ecuador	Liver cancer due to alcohol use	39(28 to 53)	162(108 to 232)	3.1(2.12 to 4.43)	0.78(0.55 to 1.07)	1.11(0.75 to 1.59)	0.43(0.09 to 0.87)
Ecuador	Liver cancer due to hepatitis B	77(62 to 94)	222(156 to 303)	1.87(1.16 to 2.76)	1.41(1.1 to 1.73)	1.49(1.04 to 2.03)	0.06(-0.2 to 0.38)
Ecuador	Liver cancer due to hepatitis C	13(8 to 19)	47(29 to 71)	2.7(1.74 to 3.84)	0.27(0.17 to 0.4)	0.35(0.21 to 0.52)	0.28(-0.04 to 0.66)
Ecuador	Liver cancer due to NASH	16(11 to 23)	68(45 to 99)	3.22(2.1 to 4.59)	0.33(0.23 to 0.46)	0.49(0.32 to 0.71)	0.49(0.11 to 0.98)
Ecuador	Liver cancer due to other causes	16(12 to 21)	40(27 to 57)	1.51(0.85 to 2.27)	0.25(0.18 to 0.33)	0.27(0.18 to 0.39)	0.1(-0.18 to 0.42)
Egypt	Liver cancer	5198(4128 to 6224)	13590(9715 to 18632)	1.61(0.74 to 3.12)	17.43(13.67 to 20.93)	21.25(15.44 to 28.92)	0.22(-0.18 to 0.89)
Egypt	Liver cancer due to alcohol use	384(226 to 597)	1200(656 to 2084)	2.13(0.95 to 4.03)	1.27(0.75 to 1.97)	1.8(1 to 3.09)	0.41(-0.1 to 1.27)
Egypt	Liver cancer due to hepatitis B	762(511 to 1120)	1774(1060 to 2859)	1.33(0.52 to 2.66)	2.26(1.5 to 3.33)	2.41(1.46 to 3.89)	0.07(-0.3 to 0.69)
Egypt	Liver cancer due to hepatitis C	3360(2517 to 4136)	8629(5849 to 12345)	1.57(0.68 to 3.05)	11.9(8.94 to 14.63)	14.05(9.83 to 19.71)	0.18(-0.21 to 0.83)
Egypt	Liver cancer due to NASH	325(212 to 492)	1233(736 to 1991)	2.79(1.49 to 5.05)	1.12(0.72 to 1.73)	1.96(1.18 to 3.09)	0.74(0.15 to 1.77)
Egypt	Liver cancer due to other causes	367(269 to 503)	755(474 to 1170)	1.06(0.34 to 2.08)	0.87(0.62 to 1.22)	1.03(0.63 to 1.62)	0.18(-0.19 to 0.74)
El Salvador	Liver cancer	106(98 to 114)	127(96 to 164)	0.19(-0.11 to 0.55)	3.55(3.26 to 3.84)	2.12(1.61 to 2.76)	-0.4(-0.55 to -0.22)
El Salvador	Liver cancer due to alcohol use	27(19 to 35)	39(25 to 56)	0.48(0.1 to 0.97)	0.91(0.65 to 1.19)	0.67(0.43 to 0.96)	-0.27(-0.46 to -0.03)

El Salvador	Liver cancer due to hepatitis B	20(15 to 27)	18(11 to 28)	-0.09(-0.36 to 0.22)	0.63(0.45 to 0.87)	0.31(0.19 to 0.47)	-0.51(-0.65 to -0.34)
El Salvador	Liver cancer due to hepatitis C	43(33 to 53)	51(35 to 71)	0.19(-0.12 to 0.57)	1.5(1.15 to 1.87)	0.84(0.57 to 1.18)	-0.44(-0.59 to -0.26)
El Salvador	Liver cancer due to NASH	8(5 to 11)	12(7 to 18)	0.52(0.11 to 1.04)	0.26(0.18 to 0.38)	0.19(0.12 to 0.3)	-0.26(-0.45 to -0.01)
El Salvador	Liver cancer due to other causes	9(7 to 12)	7(4 to 10)	-0.27(-0.48 to -0.01)	0.24(0.18 to 0.33)	0.11(0.07 to 0.17)	-0.53(-0.66 to -0.37)
Equatorial Guinea	Liver cancer	7(5 to 9)	17(10 to 25)	1.42(0.31 to 2.93)	3.24(2.44 to 4.21)	3.48(2.04 to 5.04)	0.07(-0.41 to 0.69)
Equatorial Guinea	Liver cancer due to alcohol use	1(1 to 1)	3(1 to 5)	2.23(0.65 to 4.6)	0.44(0.26 to 0.67)	0.62(0.32 to 1.02)	0.42(-0.25 to 1.44)
Equatorial Guinea	Liver cancer due to hepatitis B	2(1 to 3)	5(2 to 7)	1.5(0.32 to 3.24)	0.79(0.52 to 1.17)	0.77(0.41 to 1.25)	-0.03(-0.49 to 0.61)
Equatorial Guinea	Liver cancer due to hepatitis C	3(2 to 4)	6(4 to 10)	1.23(0.19 to 2.57)	1.62(1.14 to 2.2)	1.61(0.93 to 2.47)	0(-0.46 to 0.57)
Equatorial Guinea	Liver cancer due to NASH	0(0 to 1)	1(1 to 2)	2.76(1.04 to 5.1)	0.2(0.12 to 0.32)	0.32(0.17 to 0.52)	0.58(-0.13 to 1.5)
Equatorial Guinea	Liver cancer due to other causes	1(1 to 1)	1(1 to 2)	0.54(-0.2 to 1.77)	0.2(0.14 to 0.28)	0.16(0.09 to 0.26)	-0.18(-0.54 to 0.33)
Eritrea	Liver cancer	36(24 to 55)	88(60 to 129)	1.46(0.69 to 2.53)	3.4(2.31 to 5.24)	3.32(2.36 to 4.73)	-0.02(-0.31 to 0.4)
Eritrea	Liver cancer due to alcohol use	7(4 to 13)	19(11 to 32)	1.56(0.76 to 2.8)	0.76(0.42 to 1.36)	0.74(0.42 to 1.24)	-0.03(-0.31 to 0.41)
Eritrea	Liver cancer due to hepatitis B	12(8 to 19)	28(17 to 44)	1.26(0.51 to 2.35)	1.02(0.63 to 1.63)	0.87(0.52 to 1.37)	-0.15(-0.41 to 0.25)
Eritrea	Liver cancer due to hepatitis C	7(4 to 13)	20(12 to 30)	1.78(0.87 to 3.27)	0.92(0.53 to 1.53)	0.98(0.62 to 1.44)	0.06(-0.27 to 0.61)

Eritrea	Liver cancer due to NASH	3(2 to 6)	10(6 to 17)	1.94(0.93 to 3.54)	0.4(0.23 to 0.68)	0.45(0.27 to 0.71)	0.11(-0.25 to 0.69)
Eritrea	Liver cancer due to other causes	5(3 to 8)	11(7 to 16)	1.07(0.28 to 2.32)	0.3(0.18 to 0.47)	0.29(0.19 to 0.43)	-0.02(-0.34 to 0.49)
Estonia	Liver cancer	43(40 to 46)	95(73 to 118)	1.2(0.71 to 1.75)	2.12(1.98 to 2.27)	3.61(2.76 to 4.54)	0.7(0.31 to 1.14)
Estonia	Liver cancer due to alcohol use	15(11 to 19)	42(29 to 55)	1.69(1.02 to 2.52)	0.75(0.56 to 0.94)	1.6(1.13 to 2.12)	1.14(0.6 to 1.81)
Estonia	Liver cancer due to hepatitis B	10(7 to 14)	16(10 to 23)	0.55(0.15 to 1.01)	0.51(0.36 to 0.69)	0.67(0.43 to 0.99)	0.33(-0.01 to 0.73)
Estonia	Liver cancer due to hepatitis C	12(9 to 16)	26(17 to 36)	1.14(0.64 to 1.76)	0.59(0.42 to 0.77)	0.91(0.59 to 1.29)	0.54(0.17 to 0.94)
Estonia	Liver cancer due to NASH	3(2 to 5)	8(5 to 13)	1.6(0.92 to 2.46)	0.16(0.11 to 0.22)	0.3(0.19 to 0.45)	0.9(0.43 to 1.5)
Estonia	Liver cancer due to other causes	2(1 to 3)	3(2 to 4)	0.43(0 to 0.97)	0.12(0.08 to 0.16)	0.13(0.08 to 0.18)	0.09(-0.24 to 0.55)
Eswatini	Liver cancer	18(11 to 35)	118(34 to 215)	5.6(0.16 to 14.93)	5.95(3.91 to 11.21)	19.09(5.98 to 33.88)	2.21(-0.39 to 6.45)
Eswatini	Liver cancer due to alcohol use	3(2 to 8)	27(7 to 57)	7.49(0.19 to 20.58)	1.1(0.56 to 2.59)	4.58(1.14 to 9.27)	3.17(-0.38 to 9.12)
Eswatini	Liver cancer due to hepatitis B	7(4 to 16)	52(12 to 100)	6.22(0 to 18.25)	2.09(1.15 to 4.48)	7.43(1.82 to 14.12)	2.55(-0.49 to 8.09)
Eswatini	Liver cancer due to hepatitis C	5(3 to 8)	23(8 to 43)	3.84(0.26 to 9.48)	1.88(1.16 to 3.2)	4.46(1.64 to 8.17)	1.37(-0.33 to 4.01)
Eswatini	Liver cancer due to NASH	2(1 to 3)	11(3 to 20)	5.52(0.47 to 14.05)	0.59(0.33 to 1.07)	1.87(0.62 to 3.44)	2.17(-0.24 to 6.12)
Eswatini	Liver cancer due to other causes	1(1 to 2)	5(2 to 10)	3.61(0.13 to 10.03)	0.29(0.17 to 0.52)	0.75(0.26 to 1.46)	1.54(-0.37 to 4.87)

Ethiopia	Liver cancer	668(461 to 961)	1226(973 to 1547)	0.84(0.07 to 1.84)	3.13(2.24 to 4.22)	3.02(2.4 to 3.84)	-0.04(-0.4 to 0.43)
Ethiopia	Liver cancer due to alcohol use	134(89 to 192)	264(194 to 357)	0.96(0.15 to 2.11)	0.7(0.47 to 0.96)	0.69(0.51 to 0.93)	-0.01(-0.4 to 0.53)
Ethiopia	Liver cancer due to hepatitis B	153(102 to 227)	274(204 to 367)	0.79(0 to 1.85)	0.67(0.45 to 0.96)	0.6(0.44 to 0.81)	-0.1(-0.47 to 0.38)
Ethiopia	Liver cancer due to hepatitis C	200(136 to 284)	422(328 to 537)	1.11(0.31 to 2.18)	1.21(0.86 to 1.65)	1.21(0.95 to 1.55)	0(-0.34 to 0.47)
Ethiopia	Liver cancer due to NASH	54(36 to 80)	118(91 to 155)	1.17(0.31 to 2.36)	0.29(0.2 to 0.41)	0.31(0.24 to 0.41)	0.05(-0.33 to 0.55)
Ethiopia	Liver cancer due to other causes	126(76 to 218)	148(112 to 196)	0.18(-0.42 to 1.2)	0.26(0.17 to 0.41)	0.2(0.16 to 0.26)	-0.23(-0.57 to 0.26)
Fiji	Liver cancer	22(18 to 27)	47(36 to 60)	1.13(0.51 to 2.07)	5.95(4.79 to 7.33)	6.23(4.86 to 7.83)	0.05(-0.24 to 0.49)
Fiji	Liver cancer due to alcohol use	3(2 to 4)	6(4 to 10)	1.47(0.73 to 2.64)	0.73(0.45 to 1.11)	0.84(0.51 to 1.31)	0.15(-0.19 to 0.68)
Fiji	Liver cancer due to hepatitis B	12(9 to 16)	24(17 to 34)	0.99(0.37 to 1.89)	2.94(2.17 to 3.86)	2.94(2.1 to 4.07)	0(-0.3 to 0.44)
Fiji	Liver cancer due to hepatitis C	4(3 to 6)	10(6 to 14)	1.2(0.59 to 2.17)	1.48(0.98 to 2.11)	1.5(1.02 to 2.14)	0.01(-0.25 to 0.43)
Fiji	Liver cancer due to NASH	2(1 to 3)	5(3 to 7)	1.69(0.89 to 2.82)	0.51(0.33 to 0.75)	0.67(0.43 to 0.98)	0.3(-0.07 to 0.88)
Fiji	Liver cancer due to other causes	1(1 to 2)	2(1 to 3)	0.83(0.27 to 1.69)	0.28(0.18 to 0.4)	0.28(0.18 to 0.42)	0.03(-0.27 to 0.5)
Finland	Liver cancer	205(193 to 218)	509(462 to 560)	1.48(1.24 to 1.76)	2.85(2.67 to 3.03)	4.03(3.68 to 4.42)	0.41(0.28 to 0.58)
Finland	Liver cancer due to alcohol use	75(56 to 94)	193(141 to 245)	1.58(1.23 to 2.01)	1.03(0.77 to 1.29)	1.54(1.14 to 1.94)	0.49(0.3 to 0.73)

Finland	Liver cancer due to hepatitis B	27(18 to 39)	57(37 to 86)	1.1(0.77 to 1.47)	0.39(0.27 to 0.56)	0.51(0.35 to 0.73)	0.29(0.1 to 0.49)
Finland	Liver cancer due to hepatitis C	81(60 to 102)	203(152 to 257)	1.52(1.21 to 1.87)	1.1(0.83 to 1.38)	1.52(1.13 to 1.94)	0.39(0.23 to 0.57)
Finland	Liver cancer due to NASH	13(8 to 19)	36(23 to 53)	1.78(1.32 to 2.36)	0.17(0.12 to 0.25)	0.27(0.18 to 0.4)	0.57(0.32 to 0.86)
Finland	Liver cancer due to other causes	10(7 to 14)	21(14 to 32)	1.08(0.72 to 1.55)	0.15(0.11 to 0.21)	0.19(0.13 to 0.27)	0.25(0.07 to 0.5)
France	Liver cancer	4009(3813 to 4199)	7792(6826 to 8775)	0.94(0.7 to 1.19)	4.94(4.7 to 5.16)	5.8(5.1 to 6.56)	0.18(0.03 to 0.33)
France	Liver cancer due to alcohol use	1508(1103 to 1914)	2558(1802 to 3413)	0.7(0.43 to 1)	1.87(1.38 to 2.36)	1.97(1.4 to 2.61)	0.05(-0.11 to 0.24)
France	Liver cancer due to hepatitis B	530(356 to 757)	897(586 to 1331)	0.69(0.43 to 1)	0.69(0.47 to 0.98)	0.76(0.5 to 1.11)	0.1(-0.07 to 0.28)
France	Liver cancer due to hepatitis C	1605(1202 to 2056)	3504(2649 to 4375)	1.18(0.87 to 1.55)	1.92(1.44 to 2.5)	2.45(1.84 to 3.11)	0.28(0.1 to 0.48)
France	Liver cancer due to NASH	203(137 to 302)	509(333 to 744)	1.51(1.07 to 1.97)	0.24(0.16 to 0.36)	0.36(0.24 to 0.53)	0.49(0.23 to 0.78)
France	Liver cancer due to other causes	164(114 to 236)	325(215 to 469)	0.97(0.63 to 1.36)	0.22(0.15 to 0.3)	0.27(0.18 to 0.37)	0.23(0.03 to 0.45)
Gabon	Liver cancer	21(15 to 27)	36(24 to 50)	0.73(0.13 to 1.58)	3.59(2.67 to 4.57)	3.54(2.33 to 5.01)	-0.02(-0.35 to 0.47)
Gabon	Liver cancer due to alcohol use	3(2 to 5)	6(3 to 11)	0.93(0.18 to 1.98)	0.6(0.35 to 0.91)	0.64(0.34 to 1.1)	0.07(-0.33 to 0.67)
Gabon	Liver cancer due to hepatitis B	4(3 to 6)	8(5 to 13)	0.8(0.13 to 1.87)	0.72(0.46 to 1.04)	0.68(0.4 to 1.1)	-0.05(-0.4 to 0.46)
Gabon	Liver cancer due to hepatitis C	9(7 to 13)	16(10 to 24)	0.7(0.1 to 1.58)	1.84(1.31 to 2.42)	1.77(1.15 to 2.59)	-0.04(-0.37 to 0.47)

Gabon	Liver cancer due to NASH	1(1 to 2)	3(2 to 4)	1.38(0.5 to 2.69)	0.23(0.15 to 0.34)	0.3(0.17 to 0.47)	0.3(-0.16 to 1.04)
Gabon	Liver cancer due to other causes	2(1 to 3)	2(1 to 3)	0.01(-0.39 to 0.66)	0.21(0.13 to 0.3)	0.16(0.1 to 0.23)	-0.24(-0.51 to 0.17)
Gambia	Liver cancer	122(90 to 161)	410(296 to 538)	2.37(1.29 to 3.9)	30.76(23.19 to 40.07)	39.51(29.01 to 50.99)	0.28(-0.14 to 0.84)
Gambia	Liver cancer due to alcohol use	20(12 to 30)	75(44 to 112)	2.81(1.47 to 4.65)	5.4(3.45 to 8.02)	7.7(4.5 to 11.55)	0.43(-0.06 to 1.07)
Gambia	Liver cancer due to hepatitis B	70(50 to 96)	222(155 to 312)	2.18(1.08 to 3.8)	17.14(12.17 to 23.1)	20.34(14.26 to 28.33)	0.19(-0.22 to 0.77)
Gambia	Liver cancer due to hepatitis C	14(8 to 20)	53(33 to 78)	2.84(1.62 to 4.51)	4.46(2.85 to 6.4)	6.04(3.83 to 8.89)	0.35(-0.06 to 0.88)
Gambia	Liver cancer due to NASH	8(5 to 11)	33(21 to 49)	3.33(1.93 to 5.25)	2.21(1.42 to 3.22)	3.43(2.23 to 5.16)	0.55(0.06 to 1.19)
Gambia	Liver cancer due to other causes	11(7 to 15)	28(19 to 38)	1.52(0.64 to 2.83)	1.56(1.02 to 2.22)	2(1.34 to 2.89)	0.29(-0.1 to 0.84)
Georgia	Liver cancer	71(64 to 78)	210(174 to 255)	1.98(1.42 to 2.65)	1.2(1.08 to 1.32)	3.63(2.97 to 4.42)	2.03(1.46 to 2.75)
Georgia	Liver cancer due to alcohol use	19(13 to 25)	71(50 to 95)	2.78(1.99 to 3.84)	0.3(0.21 to 0.4)	1.22(0.85 to 1.63)	2.99(2.15 to 4.12)
Georgia	Liver cancer due to hepatitis B	16(11 to 22)	47(32 to 67)	1.91(1.3 to 2.67)	0.26(0.18 to 0.36)	0.87(0.6 to 1.25)	2.35(1.65 to 3.29)
Georgia	Liver cancer due to hepatitis C	26(20 to 34)	71(51 to 95)	1.7(1.21 to 2.28)	0.46(0.35 to 0.58)	1.16(0.83 to 1.54)	1.53(1.04 to 2.1)
Georgia	Liver cancer due to NASH	5(3 to 7)	15(11 to 22)	2.07(1.43 to 2.92)	0.09(0.06 to 0.13)	0.26(0.18 to 0.37)	1.93(1.33 to 2.75)
Georgia	Liver cancer due to other causes	5(3 to 6)	6(4 to 9)	0.41(-0.1 to 1.24)	0.09(0.06 to 0.12)	0.13(0.09 to 0.18)	0.49(-0.08 to 1.5)

Germany	Liver cancer	3082(2842 to 3314)	7743(7109 to 8362)	1.51(1.26 to 1.79)	2.43(2.24 to 2.6)	4.02(3.7 to 4.33)	0.66(0.48 to 0.84)
Germany	Liver cancer due to alcohol use	1330(1071 to 1598)	3549(2848 to 4234)	1.67(1.29 to 2.07)	1.05(0.85 to 1.25)	1.88(1.53 to 2.24)	0.8(0.55 to 1.08)
Germany	Liver cancer due to hepatitis B	353(250 to 493)	693(458 to 1017)	0.96(0.64 to 1.34)	0.29(0.21 to 0.4)	0.4(0.27 to 0.59)	0.37(0.15 to 0.61)
Germany	Liver cancer due to hepatitis C	978(755 to 1220)	2413(1784 to 3079)	1.47(1.15 to 1.8)	0.75(0.58 to 0.93)	1.17(0.87 to 1.49)	0.56(0.36 to 0.76)
Germany	Liver cancer due to NASH	230(160 to 323)	673(458 to 973)	1.93(1.47 to 2.45)	0.17(0.12 to 0.24)	0.33(0.23 to 0.46)	0.89(0.61 to 1.2)
Germany	Liver cancer due to other causes	191(136 to 266)	415(280 to 582)	1.17(0.8 to 1.58)	0.16(0.12 to 0.22)	0.24(0.17 to 0.32)	0.44(0.23 to 0.68)
Ghana	Liver cancer	399(307 to 535)	990(734 to 1266)	1.48(0.63 to 2.47)	6.21(4.81 to 8.22)	6(4.59 to 7.59)	-0.03(-0.37 to 0.36)
Ghana	Liver cancer due to alcohol use	73(46 to 115)	211(136 to 309)	1.87(0.8 to 3.22)	1.23(0.77 to 1.9)	1.36(0.89 to 1.98)	0.11(-0.31 to 0.62)
Ghana	Liver cancer due to hepatitis B	211(152 to 294)	503(353 to 685)	1.38(0.49 to 2.49)	3.11(2.23 to 4.28)	2.83(1.99 to 3.86)	-0.09(-0.43 to 0.3)
Ghana	Liver cancer due to hepatitis C	60(38 to 88)	138(86 to 204)	1.28(0.57 to 2.25)	1.16(0.75 to 1.66)	1.02(0.66 to 1.46)	-0.12(-0.4 to 0.23)
Ghana	Liver cancer due to NASH	24(16 to 37)	76(51 to 112)	2.12(1.09 to 3.39)	0.43(0.27 to 0.65)	0.51(0.34 to 0.76)	0.2(-0.21 to 0.69)
Ghana	Liver cancer due to other causes	30(22 to 40)	63(44 to 86)	1.11(0.45 to 1.92)	0.28(0.2 to 0.39)	0.28(0.19 to 0.41)	0(-0.29 to 0.34)
Greece	Liver cancer	351(329 to 375)	782(714 to 850)	1.23(1.02 to 1.44)	2.3(2.16 to 2.44)	3.21(2.95 to 3.47)	0.39(0.27 to 0.53)
Greece	Liver cancer due to alcohol use	120(89 to 154)	266(197 to 342)	1.22(0.96 to 1.56)	0.76(0.57 to 0.98)	1.1(0.82 to 1.41)	0.44(0.26 to 0.63)

Greece	Liver cancer due to hepatitis B	119(87 to 152)	249(182 to 333)	1.1(0.84 to 1.38)	0.78(0.58 to 0.99)	1.13(0.85 to 1.48)	0.45(0.28 to 0.62)
Greece	Liver cancer due to hepatitis C	69(48 to 94)	166(115 to 227)	1.39(1.06 to 1.79)	0.46(0.33 to 0.61)	0.59(0.41 to 0.8)	0.27(0.12 to 0.45)
Greece	Liver cancer due to NASH	25(17 to 37)	65(43 to 96)	1.57(1.13 to 2.03)	0.17(0.11 to 0.24)	0.24(0.16 to 0.34)	0.44(0.24 to 0.68)
Greece	Liver cancer due to other causes	18(12 to 25)	36(23 to 53)	1(0.66 to 1.39)	0.13(0.09 to 0.17)	0.16(0.11 to 0.22)	0.23(0.04 to 0.43)
Greenland	Liver cancer	2(1 to 2)	4(3 to 6)	1.64(0.97 to 2.57)	4.6(4.06 to 5.31)	6.35(5.03 to 7.96)	0.38(0.05 to 0.8)
Greenland	Liver cancer due to alcohol use	1(1 to 1)	2(1 to 3)	1.75(0.97 to 2.76)	1.88(1.41 to 2.41)	2.68(1.86 to 3.67)	0.42(0.05 to 0.91)
Greenland	Liver cancer due to hepatitis B	0(0 to 0)	1(0 to 1)	1.11(0.48 to 1.95)	0.61(0.41 to 0.93)	0.76(0.47 to 1.15)	0.24(-0.09 to 0.66)
Greenland	Liver cancer due to hepatitis C	0(0 to 1)	1(1 to 2)	1.86(1.12 to 2.79)	1.26(0.89 to 1.71)	1.74(1.15 to 2.52)	0.38(0.05 to 0.79)
Greenland	Liver cancer due to NASH	0(0 to 0)	0(0 to 1)	2.12(1.31 to 3.23)	0.43(0.3 to 0.62)	0.67(0.44 to 0.95)	0.55(0.17 to 1.05)
Greenland	Liver cancer due to other causes	0(0 to 0)	0(0 to 1)	1.07(0.45 to 1.87)	0.41(0.29 to 0.56)	0.5(0.32 to 0.74)	0.21(-0.12 to 0.62)
Grenada	Liver cancer	7(6 to 8)	3(3 to 4)	-0.5(-0.57 to -0.41)	9.4(8.39 to 10.41)	3.13(2.76 to 3.54)	-0.67(-0.71 to -0.61)
Grenada	Liver cancer due to alcohol use	2(2 to 3)	1(1 to 2)	-0.42(-0.52 to -0.3)	3.16(2.28 to 4.1)	1.19(0.88 to 1.54)	-0.62(-0.68 to -0.55)
Grenada	Liver cancer due to hepatitis B	2(1 to 2)	1(1 to 1)	-0.52(-0.6 to -0.42)	2.58(1.91 to 3.46)	0.74(0.52 to 1.02)	-0.71(-0.76 to -0.66)
Grenada	Liver cancer due to hepatitis C	2(1 to 2)	1(1 to 1)	-0.59(-0.66 to -0.5)	2.32(1.62 to 3.12)	0.73(0.5 to 1)	-0.69(-0.74 to -0.63)

Grenada	Liver cancer due to NASH	1(0 to 1)	0(0 to 0)	-0.45(-0.55 to -0.32)	0.71(0.49 to 1.01)	0.29(0.19 to 0.41)	-0.6(-0.67 to -0.51)
Grenada	Liver cancer due to other causes	0(0 to 1)	0(0 to 0)	-0.58(-0.66 to -0.48)	0.63(0.45 to 0.86)	0.19(0.13 to 0.26)	-0.7(-0.76 to -0.63)
Guam	Liver cancer	3(3 to 4)	11(9 to 14)	2.73(1.87 to 3.85)	3.91(3.35 to 4.54)	5.79(4.71 to 7.05)	0.48(0.16 to 0.9)
Guam	Liver cancer due to alcohol use	0(0 to 1)	1(1 to 2)	3.07(2.03 to 4.49)	0.46(0.29 to 0.68)	0.73(0.45 to 1.1)	0.58(0.18 to 1.09)
Guam	Liver cancer due to hepatitis B	2(1 to 2)	6(5 to 8)	2.78(1.85 to 4.01)	1.99(1.56 to 2.54)	3.33(2.54 to 4.24)	0.68(0.29 to 1.22)
Guam	Liver cancer due to hepatitis C	1(0 to 1)	2(1 to 3)	2.36(1.59 to 3.31)	0.92(0.63 to 1.27)	0.96(0.63 to 1.4)	0.04(-0.2 to 0.32)
Guam	Liver cancer due to NASH	0(0 to 0)	1(1 to 2)	3.34(2.29 to 4.68)	0.38(0.26 to 0.55)	0.57(0.39 to 0.84)	0.51(0.14 to 0.97)
Guam	Liver cancer due to other causes	0(0 to 0)	0(0 to 1)	1.66(1.04 to 2.46)	0.16(0.11 to 0.22)	0.19(0.13 to 0.27)	0.22(-0.06 to 0.58)
Guatemala	Liver cancer	353(312 to 394)	521(413 to 653)	0.48(0.15 to 0.93)	10.05(8.95 to 11.17)	4.81(3.84 to 6.01)	-0.52(-0.63 to -0.38)
Guatemala	Liver cancer due to alcohol use	85(59 to 114)	130(85 to 188)	0.53(0.15 to 1.04)	2.44(1.68 to 3.26)	1.2(0.78 to 1.73)	-0.51(-0.63 to -0.35)
Guatemala	Liver cancer due to hepatitis B	72(52 to 97)	88(58 to 128)	0.22(-0.07 to 0.6)	1.75(1.24 to 2.43)	0.74(0.48 to 1.1)	-0.58(-0.67 to -0.44)
Guatemala	Liver cancer due to hepatitis C	141(106 to 178)	219(157 to 294)	0.55(0.21 to 1.01)	4.5(3.46 to 5.61)	2.13(1.55 to 2.85)	-0.53(-0.63 to -0.4)
Guatemala	Liver cancer due to NASH	24(17 to 34)	46(30 to 67)	0.94(0.46 to 1.59)	0.71(0.48 to 1.01)	0.43(0.28 to 0.64)	-0.39(-0.54 to -0.2)
Guatemala	Liver cancer due to other causes	31(24 to 40)	39(27 to 54)	0.25(-0.07 to 0.68)	0.65(0.48 to 0.89)	0.31(0.21 to 0.44)	-0.53(-0.65 to -0.38)

Guinea	Liver cancer	1076(895 to 1295)	1935(1348 to 2506)	0.8(0.2 to 1.49)	31.8(26.71 to 38.1)	34.05(23.98 to 44.01)	0.07(-0.29 to 0.48)
Guinea	Liver cancer due to alcohol use	168(109 to 252)	327(189 to 493)	0.94(0.23 to 1.79)	5.16(3.36 to 7.57)	6.14(3.54 to 9.17)	0.19(-0.24 to 0.7)
Guinea	Liver cancer due to hepatitis B	574(429 to 738)	1033(693 to 1417)	0.8(0.17 to 1.56)	16.82(12.73 to 21.71)	17.49(11.6 to 23.83)	0.04(-0.33 to 0.46)
Guinea	Liver cancer due to hepatitis C	179(118 to 254)	296(182 to 436)	0.65(0.16 to 1.25)	6(3.94 to 8.41)	6.04(3.67 to 8.9)	0.01(-0.28 to 0.36)
Guinea	Liver cancer due to NASH	71(47 to 103)	144(91 to 218)	1.01(0.43 to 1.8)	2.28(1.53 to 3.31)	2.75(1.73 to 4.23)	0.21(-0.14 to 0.65)
Guinea	Liver cancer due to other causes	83(59 to 113)	136(92 to 196)	0.63(0.07 to 1.47)	1.55(1.12 to 2.15)	1.64(1.08 to 2.38)	0.05(-0.26 to 0.44)
Guinea-Bissau	Liver cancer	35(25 to 50)	47(34 to 65)	0.34(-0.06 to 0.88)	8.34(6.09 to 11.61)	6.36(4.67 to 8.51)	-0.24(-0.45 to 0.05)
Guinea-Bissau	Liver cancer due to alcohol use	6(3 to 9)	8(5 to 12)	0.37(-0.05 to 1.01)	1.44(0.87 to 2.3)	1.15(0.71 to 1.75)	-0.21(-0.45 to 0.14)
Guinea-Bissau	Liver cancer due to hepatitis B	19(13 to 27)	24(16 to 34)	0.27(-0.13 to 0.83)	4.26(2.89 to 6.06)	2.91(1.99 to 4.1)	-0.32(-0.52 to -0.03)
Guinea-Bissau	Liver cancer due to hepatitis C	6(3 to 9)	8(5 to 12)	0.4(0.02 to 0.92)	1.62(0.99 to 2.5)	1.36(0.84 to 2.09)	-0.16(-0.37 to 0.14)
Guinea-Bissau	Liver cancer due to NASH	2(1 to 4)	4(2 to 6)	0.69(0.19 to 1.35)	0.62(0.38 to 0.95)	0.61(0.37 to 0.94)	-0.02(-0.29 to 0.35)
Guinea-Bissau	Liver cancer due to other causes	3(2 to 4)	3(2 to 5)	0.25(-0.14 to 0.82)	0.41(0.26 to 0.62)	0.34(0.21 to 0.52)	-0.17(-0.41 to 0.16)
Guyana	Liver cancer	26(23 to 30)	19(15 to 25)	-0.26(-0.45 to -0.03)	6.86(5.94 to 7.84)	3.2(2.53 to 4.03)	-0.53(-0.65 to -0.4)
Guyana	Liver cancer due to alcohol use	9(6 to 12)	7(5 to 9)	-0.25(-0.45 to -0.02)	2.42(1.75 to 3.18)	1.11(0.76 to 1.54)	-0.54(-0.65 to -0.4)

Guyana	Liver cancer due to hepatitis B	8(6 to 11)	5(4 to 8)	-0.3(-0.5 to -0.06)	1.85(1.32 to 2.54)	0.82(0.55 to 1.18)	-0.55(-0.67 to -0.41)
Guyana	Liver cancer due to hepatitis C	5(4 to 8)	4(3 to 6)	-0.24(-0.42 to -0.02)	1.62(1.12 to 2.23)	0.75(0.49 to 1.06)	-0.53(-0.64 to -0.4)
Guyana	Liver cancer due to NASH	2(1 to 3)	2(1 to 3)	-0.07(-0.31 to 0.24)	0.52(0.36 to 0.75)	0.3(0.2 to 0.44)	-0.42(-0.56 to -0.23)
Guyana	Liver cancer due to other causes	2(2 to 3)	1(1 to 2)	-0.35(-0.53 to -0.12)	0.46(0.32 to 0.63)	0.21(0.14 to 0.31)	-0.54(-0.67 to -0.38)
Haiti	Liver cancer	225(139 to 310)	297(181 to 445)	0.32(-0.06 to 0.82)	7.11(4.41 to 9.95)	4.42(2.67 to 6.66)	-0.38(-0.55 to -0.15)
Haiti	Liver cancer due to alcohol use	70(41 to 111)	96(51 to 163)	0.37(-0.04 to 0.91)	2.25(1.28 to 3.65)	1.47(0.8 to 2.5)	-0.35(-0.53 to -0.09)
Haiti	Liver cancer due to hepatitis B	68(40 to 108)	91(50 to 152)	0.34(-0.07 to 0.9)	1.95(1.14 to 3.09)	1.22(0.68 to 2.08)	-0.37(-0.56 to -0.13)
Haiti	Liver cancer due to hepatitis C	52(29 to 84)	65(35 to 106)	0.25(-0.11 to 0.74)	1.9(1.09 to 3.14)	1.1(0.6 to 1.73)	-0.42(-0.59 to -0.2)
Haiti	Liver cancer due to NASH	14(8 to 23)	20(10 to 33)	0.4(0 to 0.99)	0.49(0.27 to 0.82)	0.32(0.17 to 0.52)	-0.35(-0.53 to -0.08)
Haiti	Liver cancer due to other causes	21(12 to 35)	25(14 to 39)	0.19(-0.17 to 0.68)	0.52(0.29 to 0.84)	0.31(0.17 to 0.49)	-0.41(-0.57 to -0.18)
Honduras	Liver cancer	242(93 to 349)	934(431 to 1372)	2.87(1.97 to 4.52)	11.92(4.4 to 17.38)	16.14(7.41 to 23.5)	0.35(0.04 to 0.98)
Honduras	Liver cancer due to alcohol use	63(20 to 105)	267(111 to 433)	3.23(2.17 to 5.53)	3.18(0.96 to 5.36)	4.55(1.87 to 7.31)	0.43(0.07 to 1.25)
Honduras	Liver cancer due to hepatitis B	50(18 to 85)	159(63 to 275)	2.15(1.3 to 3.49)	2.26(0.74 to 3.86)	2.53(0.98 to 4.37)	0.12(-0.17 to 0.64)
Honduras	Liver cancer due to hepatitis C	94(31 to 148)	376(154 to 605)	3.01(2.07 to 4.89)	4.99(1.63 to 7.85)	6.82(2.82 to 10.85)	0.37(0.05 to 1.03)

Honduras	Liver cancer due to NASH	16(5 to 28)	78(30 to 137)	3.97(2.67 to 6.33)	0.79(0.24 to 1.42)	1.38(0.52 to 2.42)	0.74(0.29 to 1.61)
Honduras	Liver cancer due to other causes	19(9 to 29)	55(22 to 92)	1.94(1.11 to 3.06)	0.7(0.27 to 1.17)	0.86(0.34 to 1.46)	0.22(-0.1 to 0.75)
Hungary	Liver cancer	1005(959 to 1047)	512(423 to 622)	-0.49(-0.58 to -0.38)	6.82(6.51 to 7.11)	2.65(2.18 to 3.23)	-0.61(-0.68 to -0.52)
Hungary	Liver cancer due to alcohol use	448(357 to 534)	219(158 to 289)	-0.51(-0.61 to -0.39)	2.98(2.39 to 3.55)	1.12(0.8 to 1.48)	-0.63(-0.7 to -0.53)
Hungary	Liver cancer due to hepatitis B	212(149 to 292)	103(69 to 151)	-0.51(-0.61 to -0.39)	1.47(1.06 to 1.99)	0.58(0.39 to 0.83)	-0.61(-0.69 to -0.51)
Hungary	Liver cancer due to hepatitis C	241(168 to 322)	131(88 to 184)	-0.46(-0.56 to -0.34)	1.65(1.16 to 2.16)	0.64(0.43 to 0.9)	-0.61(-0.68 to -0.52)
Hungary	Liver cancer due to NASH	72(50 to 102)	44(30 to 64)	-0.38(-0.52 to -0.22)	0.49(0.35 to 0.69)	0.22(0.15 to 0.31)	-0.55(-0.65 to -0.43)
Hungary	Liver cancer due to other causes	31(21 to 43)	15(9 to 21)	-0.53(-0.63 to -0.4)	0.23(0.17 to 0.31)	0.09(0.06 to 0.12)	-0.61(-0.69 to -0.51)
Iceland	Liver cancer	5(5 to 6)	16(14 to 18)	1.93(1.54 to 2.39)	1.88(1.72 to 2.04)	2.84(2.51 to 3.2)	0.51(0.31 to 0.75)
Iceland	Liver cancer due to alcohol use	2(1 to 2)	6(4 to 7)	2.32(1.79 to 2.96)	0.6(0.43 to 0.78)	1.04(0.76 to 1.35)	0.73(0.46 to 1.06)
Iceland	Liver cancer due to hepatitis B	1(1 to 1)	2(1 to 3)	1.65(1.27 to 2.11)	0.3(0.21 to 0.43)	0.42(0.29 to 0.61)	0.41(0.2 to 0.65)
Iceland	Liver cancer due to hepatitis C	2(2 to 3)	6(4 to 8)	1.78(1.38 to 2.23)	0.74(0.56 to 0.93)	1.03(0.76 to 1.35)	0.4(0.2 to 0.63)
Iceland	Liver cancer due to NASH	0(0 to 1)	1(1 to 2)	2.14(1.57 to 2.82)	0.13(0.09 to 0.18)	0.2(0.13 to 0.3)	0.61(0.33 to 0.96)
Iceland	Liver cancer due to other causes	0(0 to 0)	1(0 to 1)	1.31(0.88 to 1.78)	0.11(0.08 to 0.15)	0.14(0.1 to 0.2)	0.25(0.03 to 0.49)

India	Liver cancer	11859(9876 to 13742)	30709(25884 to 36351)	1.59(1.11 to 2.14)	2.7(2.21 to 3.15)	2.75(2.32 to 3.27)	0.02(-0.17 to 0.23)
India	Liver cancer due to alcohol use	2908(2213 to 3672)	8577(6764 to 10583)	1.95(1.34 to 2.7)	0.69(0.52 to 0.87)	0.76(0.61 to 0.94)	0.11(-0.12 to 0.39)
India	Liver cancer due to hepatitis B	4898(3919 to 5898)	10784(8690 to 13220)	1.2(0.76 to 1.72)	1.01(0.8 to 1.22)	0.91(0.74 to 1.12)	-0.1(-0.28 to 0.11)
India	Liver cancer due to hepatitis C	2031(1592 to 2531)	6292(4887 to 7983)	2.1(1.45 to 2.74)	0.57(0.45 to 0.7)	0.61(0.47 to 0.77)	0.07(-0.15 to 0.29)
India	Liver cancer due to NASH	1137(898 to 1404)	3520(2777 to 4361)	2.1(1.5 to 2.76)	0.29(0.23 to 0.36)	0.33(0.26 to 0.41)	0.13(-0.09 to 0.36)
India	Liver cancer due to other causes	885(750 to 1031)	1537(1259 to 1864)	0.74(0.42 to 1.11)	0.13(0.11 to 0.15)	0.13(0.11 to 0.16)	-0.02(-0.18 to 0.15)
Indonesia	Liver cancer	2554(2192 to 2891)	4721(3974 to 5403)	0.85(0.46 to 1.32)	2.62(2.25 to 3.03)	2.45(2.08 to 2.76)	-0.07(-0.25 to 0.16)
Indonesia	Liver cancer due to alcohol use	417(330 to 521)	878(678 to 1098)	1.1(0.62 to 1.67)	0.44(0.35 to 0.55)	0.43(0.34 to 0.53)	-0.02(-0.24 to 0.23)
Indonesia	Liver cancer due to hepatitis B	574(474 to 689)	974(750 to 1222)	0.7(0.31 to 1.16)	0.5(0.41 to 0.61)	0.43(0.34 to 0.53)	-0.13(-0.31 to 0.09)
Indonesia	Liver cancer due to hepatitis C	1072(902 to 1289)	2163(1784 to 2521)	1.02(0.59 to 1.54)	1.29(1.08 to 1.54)	1.22(1.02 to 1.39)	-0.05(-0.25 to 0.18)
Indonesia	Liver cancer due to NASH	213(170 to 261)	462(372 to 564)	1.17(0.72 to 1.73)	0.23(0.18 to 0.28)	0.25(0.2 to 0.29)	0.08(-0.14 to 0.34)
Indonesia	Liver cancer due to other causes	278(207 to 400)	244(202 to 291)	-0.12(-0.43 to 0.26)	0.16(0.13 to 0.22)	0.12(0.1 to 0.14)	-0.29(-0.5 to -0.03)
Iran (Islamic Republic of)	Liver cancer	1076(889 to 1258)	2501(2272 to 2750)	1.32(0.86 to 1.96)	4.57(3.7 to 5.55)	3.64(3.29 to 4.02)	-0.2(-0.38 to 0.04)
Iran (Islamic Republic of)	Liver cancer due to alcohol use	118(85 to 159)	271(205 to 355)	1.29(0.88 to 1.95)	0.5(0.36 to 0.65)	0.4(0.3 to 0.52)	-0.2(-0.35 to 0.03)

Iran (Islamic Republic of)	Liver cancer due to hepatitis B	458(377 to 540)	974(834 to 1128)	1.13(0.76 to 1.69)	1.72(1.41 to 2.03)	1.32(1.13 to 1.55)	-0.23(-0.37 to -0.02)
Iran (Islamic Republic of)	Liver cancer due to hepatitis C	272(209 to 355)	720(607 to 840)	1.65(0.95 to 2.59)	1.42(1.08 to 1.86)	1.12(0.94 to 1.31)	-0.21(-0.43 to 0.08)
Iran (Islamic Republic of)	Liver cancer due to NASH	119(90 to 152)	354(297 to 423)	1.98(1.22 to 2.99)	0.57(0.42 to 0.75)	0.54(0.45 to 0.64)	-0.06(-0.32 to 0.27)
Iran (Islamic Republic of)	Liver cancer due to other causes	110(90 to 133)	183(157 to 215)	0.67(0.35 to 1.06)	0.37(0.29 to 0.47)	0.26(0.22 to 0.3)	-0.3(-0.46 to -0.09)
Iraq	Liver cancer	358(275 to 444)	1336(1018 to 1687)	2.73(1.64 to 4.25)	4.58(3.53 to 5.66)	6.07(4.72 to 7.47)	0.33(-0.05 to 0.84)
Iraq	Liver cancer due to alcohol use	32(20 to 50)	129(78 to 195)	2.98(1.72 to 4.73)	0.43(0.26 to 0.66)	0.6(0.37 to 0.91)	0.39(-0.04 to 0.99)
Iraq	Liver cancer due to hepatitis B	133(93 to 183)	488(331 to 691)	2.67(1.51 to 4.3)	1.6(1.11 to 2.21)	1.97(1.34 to 2.76)	0.23(-0.15 to 0.75)
Iraq	Liver cancer due to hepatitis C	127(88 to 175)	480(326 to 666)	2.76(1.66 to 4.32)	1.8(1.27 to 2.42)	2.43(1.7 to 3.31)	0.35(-0.03 to 0.87)
Iraq	Liver cancer due to NASH	33(22 to 48)	144(93 to 214)	3.39(2.08 to 5.34)	0.44(0.29 to 0.65)	0.69(0.45 to 1.01)	0.56(0.1 to 1.23)
Iraq	Liver cancer due to other causes	33(24 to 43)	96(66 to 142)	1.96(1.09 to 3.15)	0.31(0.2 to 0.43)	0.38(0.25 to 0.56)	0.23(-0.14 to 0.72)
Ireland	Liver cancer	61(57 to 65)	257(230 to 283)	3.24(2.72 to 3.76)	1.47(1.37 to 1.57)	3.4(3.04 to 3.75)	1.31(1.04 to 1.59)
Ireland	Liver cancer due to alcohol use	22(16 to 28)	95(70 to 123)	3.39(2.71 to 4.11)	0.51(0.38 to 0.66)	1.26(0.93 to 1.61)	1.45(1.08 to 1.85)
Ireland	Liver cancer due to hepatitis B	8(5 to 11)	30(20 to 44)	2.84(2.3 to 3.41)	0.19(0.13 to 0.27)	0.41(0.28 to 0.6)	1.12(0.81 to 1.44)
Ireland	Liver cancer due to hepatitis C	24(18 to 30)	101(76 to 129)	3.24(2.69 to 3.91)	0.58(0.44 to 0.72)	1.31(0.99 to 1.68)	1.27(0.99 to 1.61)

Ireland	Liver cancer due to NASH	4(3 to 6)	19(13 to 28)	3.88(3.08 to 4.8)	0.09(0.07 to 0.14)	0.25(0.17 to 0.37)	1.66(1.25 to 2.14)
Ireland	Liver cancer due to other causes	4(3 to 5)	12(8 to 17)	2.42(1.83 to 3.08)	0.09(0.07 to 0.12)	0.17(0.12 to 0.23)	0.9(0.6 to 1.26)
Israel	Liver cancer	130(117 to 143)	324(295 to 354)	1.5(1.21 to 1.85)	2.65(2.39 to 2.92)	2.79(2.55 to 3.04)	0.05(-0.06 to 0.2)
Israel	Liver cancer due to alcohol use	30(21 to 41)	84(59 to 112)	1.78(1.35 to 2.28)	0.61(0.42 to 0.83)	0.73(0.51 to 0.97)	0.2(0.02 to 0.42)
Israel	Liver cancer due to hepatitis B	20(14 to 30)	45(30 to 66)	1.25(0.95 to 1.63)	0.41(0.29 to 0.61)	0.41(0.28 to 0.59)	-0.01(-0.14 to 0.15)
Israel	Liver cancer due to hepatitis C	61(47 to 76)	149(116 to 185)	1.45(1.13 to 1.82)	1.26(0.97 to 1.55)	1.25(0.97 to 1.56)	0(-0.12 to 0.14)
Israel	Liver cancer due to NASH	10(6 to 15)	27(18 to 39)	1.72(1.26 to 2.27)	0.2(0.13 to 0.29)	0.23(0.16 to 0.33)	0.15(-0.03 to 0.36)
Israel	Liver cancer due to other causes	8(6 to 12)	19(13 to 27)	1.24(0.91 to 1.67)	0.17(0.12 to 0.24)	0.17(0.12 to 0.24)	-0.01(-0.15 to 0.18)
Italy	Liver cancer	5829(5642 to 5960)	6919(6225 to 7495)	0.19(0.09 to 0.27)	6.5(6.29 to 6.64)	4.8(4.36 to 5.17)	-0.26(-0.32 to -0.21)
Italy	Liver cancer due to alcohol use	1596(1378 to 1809)	1724(1450 to 2008)	0.08(-0.04 to 0.2)	1.77(1.53 to 2)	1.25(1.05 to 1.45)	-0.29(-0.36 to -0.22)
Italy	Liver cancer due to hepatitis B	687(573 to 824)	654(536 to 799)	-0.05(-0.15 to 0.05)	0.8(0.68 to 0.96)	0.52(0.43 to 0.64)	-0.35(-0.41 to -0.29)
Italy	Liver cancer due to hepatitis C	3121(2853 to 3391)	4032(3535 to 4452)	0.29(0.17 to 0.38)	3.43(3.13 to 3.73)	2.66(2.34 to 2.93)	-0.22(-0.28 to -0.17)
Italy	Liver cancer due to NASH	270(227 to 322)	341(275 to 414)	0.26(0.14 to 0.39)	0.3(0.25 to 0.35)	0.22(0.18 to 0.27)	-0.24(-0.31 to -0.19)
Italy	Liver cancer due to other causes	154(130 to 183)	167(138 to 203)	0.08(-0.02 to 0.18)	0.2(0.17 to 0.23)	0.14(0.12 to 0.16)	-0.3(-0.34 to -0.25)

Jamaica	Liver cancer	74(69 to 79)	82(65 to 101)	0.1(-0.14 to 0.4)	4.1(3.84 to 4.39)	2.75(2.19 to 3.41)	-0.33(-0.48 to -0.15)
Jamaica	Liver cancer due to alcohol use	24(18 to 31)	27(19 to 38)	0.15(-0.12 to 0.45)	1.32(0.99 to 1.72)	0.93(0.64 to 1.28)	-0.3(-0.46 to -0.11)
Jamaica	Liver cancer due to hepatitis B	21(15 to 28)	21(14 to 30)	0.01(-0.23 to 0.33)	1.19(0.87 to 1.57)	0.71(0.48 to 1)	-0.4(-0.55 to -0.22)
Jamaica	Liver cancer due to hepatitis C	18(13 to 24)	20(13 to 28)	0.09(-0.15 to 0.38)	0.99(0.7 to 1.3)	0.65(0.43 to 0.94)	-0.34(-0.49 to -0.17)
Jamaica	Liver cancer due to NASH	6(4 to 9)	8(5 to 12)	0.37(0.03 to 0.79)	0.33(0.23 to 0.46)	0.28(0.18 to 0.42)	-0.16(-0.37 to 0.09)
Jamaica	Liver cancer due to other causes	5(4 to 7)	5(4 to 7)	-0.02(-0.26 to 0.26)	0.28(0.2 to 0.38)	0.18(0.12 to 0.25)	-0.35(-0.51 to -0.16)
Japan	Liver cancer	19987(19306 to 20460)	34515(29605 to 37430)	0.73(0.53 to 0.85)	11.67(11.25 to 11.96)	8.78(7.8 to 9.42)	-0.25(-0.31 to -0.21)
Japan	Liver cancer due to alcohol use	2592(2204 to 2998)	3562(2919 to 4194)	0.37(0.21 to 0.56)	1.49(1.27 to 1.72)	0.99(0.83 to 1.16)	-0.34(-0.39 to -0.28)
Japan	Liver cancer due to hepatitis B	2934(2546 to 3385)	3368(2760 to 4091)	0.15(0 to 0.31)	1.71(1.49 to 1.96)	1.05(0.89 to 1.24)	-0.39(-0.44 to -0.34)
Japan	Liver cancer due to hepatitis C	13060(12273 to 13760)	25052(21086 to 27540)	0.92(0.68 to 1.07)	7.63(7.16 to 8.03)	6.08(5.3 to 6.63)	-0.2(-0.28 to -0.15)
Japan	Liver cancer due to NASH	806(683 to 946)	1650(1259 to 2030)	1.05(0.72 to 1.38)	0.47(0.4 to 0.55)	0.4(0.32 to 0.48)	-0.16(-0.26 to -0.07)
Japan	Liver cancer due to other causes	596(510 to 689)	883(710 to 1082)	0.48(0.24 to 0.72)	0.36(0.31 to 0.42)	0.26(0.22 to 0.3)	-0.29(-0.36 to -0.22)
Jordan	Liver cancer	40(32 to 50)	141(113 to 177)	2.52(1.55 to 3.94)	3.21(2.52 to 4.02)	2.38(1.91 to 2.97)	-0.26(-0.47 to 0.05)
Jordan	Liver cancer due to alcohol use	4(2 to 6)	16(9 to 25)	3.19(1.83 to 5.18)	0.32(0.19 to 0.48)	0.27(0.16 to 0.42)	-0.15(-0.43 to 0.25)

Jordan	Liver cancer due to hepatitis B	16(12 to 22)	52(36 to 72)	2.24(1.29 to 3.73)	1.12(0.8 to 1.56)	0.77(0.53 to 1.08)	-0.32(-0.52 to 0)
Jordan	Liver cancer due to hepatitis C	13(8 to 18)	46(31 to 62)	2.58(1.51 to 4.11)	1.22(0.8 to 1.68)	0.88(0.61 to 1.19)	-0.28(-0.49 to 0.04)
Jordan	Liver cancer due to NASH	4(3 to 6)	18(12 to 26)	3.6(2.26 to 5.59)	0.34(0.21 to 0.51)	0.32(0.21 to 0.48)	-0.06(-0.34 to 0.38)
Jordan	Liver cancer due to other causes	4(3 to 5)	10(7 to 14)	1.7(1.01 to 2.65)	0.21(0.14 to 0.31)	0.14(0.1 to 0.21)	-0.32(-0.51 to -0.04)
Kazakhstan	Liver cancer	453(425 to 483)	1116(956 to 1291)	1.46(1.08 to 1.88)	3.57(3.34 to 3.82)	6.54(5.63 to 7.5)	0.83(0.56 to 1.13)
Kazakhstan	Liver cancer due to alcohol use	140(101 to 178)	360(254 to 470)	1.57(1.14 to 2.08)	1.1(0.8 to 1.4)	2.07(1.49 to 2.72)	0.88(0.57 to 1.26)
Kazakhstan	Liver cancer due to hepatitis B	116(83 to 156)	257(177 to 364)	1.23(0.85 to 1.65)	0.86(0.61 to 1.17)	1.4(0.96 to 1.98)	0.62(0.35 to 0.92)
Kazakhstan	Liver cancer due to hepatitis C	147(108 to 188)	372(259 to 502)	1.52(1.14 to 1.96)	1.23(0.9 to 1.55)	2.3(1.62 to 3.05)	0.88(0.6 to 1.19)
Kazakhstan	Liver cancer due to NASH	26(18 to 38)	85(57 to 125)	2.23(1.66 to 2.9)	0.22(0.15 to 0.31)	0.52(0.35 to 0.77)	1.42(1 to 1.91)
Kazakhstan	Liver cancer due to other causes	24(17 to 32)	42(29 to 57)	0.7(0.19 to 1.41)	0.17(0.12 to 0.22)	0.24(0.17 to 0.33)	0.44(0.05 to 0.96)
Kenya	Liver cancer	224(156 to 392)	724(491 to 1056)	2.24(1.56 to 3.1)	2.65(1.82 to 4.67)	3.34(2.28 to 4.81)	0.26(0 to 0.56)
Kenya	Liver cancer due to alcohol use	56(34 to 103)	183(112 to 285)	2.25(1.52 to 3.12)	0.71(0.43 to 1.3)	0.86(0.54 to 1.33)	0.22(-0.05 to 0.53)
Kenya	Liver cancer due to hepatitis B	62(39 to 110)	208(132 to 312)	2.36(1.56 to 3.28)	0.67(0.42 to 1.18)	0.81(0.52 to 1.2)	0.22(-0.06 to 0.52)
Kenya	Liver cancer due to hepatitis C	47(32 to 80)	155(102 to 227)	2.29(1.61 to 3.12)	0.66(0.46 to 1.13)	0.86(0.58 to 1.23)	0.29(0.04 to 0.58)

Kenya	Liver cancer due to NASH	29(20 to 50)	107(72 to 158)	2.65(1.87 to 3.57)	0.38(0.26 to 0.64)	0.53(0.36 to 0.77)	0.42(0.13 to 0.75)
Kenya	Liver cancer due to other causes	29(23 to 45)	72(49 to 105)	1.46(0.8 to 2.29)	0.23(0.17 to 0.38)	0.27(0.18 to 0.39)	0.18(-0.07 to 0.49)
Kiribati	Liver cancer	6(4 to 7)	9(7 to 11)	0.56(0.1 to 1.16)	14.29(11.14 to 17.77)	11.95(9.29 to 15.08)	-0.16(-0.4 to 0.15)
Kiribati	Liver cancer due to alcohol use	1(0 to 1)	1(1 to 1)	0.59(0.06 to 1.32)	1.42(0.86 to 2.18)	1.21(0.72 to 1.78)	-0.15(-0.43 to 0.23)
Kiribati	Liver cancer due to hepatitis B	3(2 to 4)	5(4 to 7)	0.5(0.03 to 1.11)	7.71(5.69 to 10.33)	5.97(4.3 to 8.02)	-0.23(-0.46 to 0.08)
Kiribati	Liver cancer due to hepatitis C	1(1 to 2)	2(1 to 3)	0.59(0.12 to 1.26)	3.36(2.28 to 4.69)	3(1.93 to 4.33)	-0.11(-0.36 to 0.24)
Kiribati	Liver cancer due to NASH	0(0 to 1)	1(1 to 1)	0.87(0.31 to 1.63)	1.13(0.74 to 1.69)	1.17(0.77 to 1.76)	0.03(-0.26 to 0.45)
Kiribati	Liver cancer due to other causes	0(0 to 0)	1(0 to 1)	0.64(0.16 to 1.26)	0.66(0.44 to 0.94)	0.6(0.4 to 0.9)	-0.09(-0.35 to 0.25)
Kuwait	Liver cancer	17(15 to 19)	54(42 to 68)	2.15(1.44 to 3)	2.76(2.45 to 3.09)	2.36(1.85 to 2.96)	-0.15(-0.33 to 0.07)
Kuwait	Liver cancer due to alcohol use	2(1 to 2)	6(3 to 9)	2.43(1.52 to 3.61)	0.29(0.18 to 0.43)	0.26(0.15 to 0.4)	-0.1(-0.34 to 0.19)
Kuwait	Liver cancer due to hepatitis B	7(6 to 9)	20(13 to 28)	1.67(1 to 2.5)	1.02(0.75 to 1.34)	0.73(0.48 to 1.07)	-0.28(-0.46 to -0.06)
Kuwait	Liver cancer due to hepatitis C	5(3 to 6)	18(12 to 24)	2.68(1.82 to 3.7)	0.98(0.7 to 1.25)	0.89(0.63 to 1.2)	-0.09(-0.3 to 0.14)
Kuwait	Liver cancer due to NASH	2(1 to 2)	7(5 to 11)	3.41(2.28 to 4.79)	0.31(0.21 to 0.44)	0.35(0.23 to 0.53)	0.14(-0.15 to 0.49)
Kuwait	Liver cancer due to other causes	2(1 to 2)	3(2 to 5)	1.15(0.63 to 1.73)	0.17(0.12 to 0.23)	0.13(0.09 to 0.19)	-0.25(-0.42 to -0.04)

Kyrgyzstan	Liver cancer	41(37 to 44)	117(100 to 136)	1.89(1.42 to 2.38)	1.32(1.21 to 1.42)	2.7(2.31 to 3.09)	1.05(0.72 to 1.41)
Kyrgyzstan	Liver cancer due to alcohol use	10(7 to 13)	35(25 to 47)	2.5(1.82 to 3.35)	0.34(0.24 to 0.45)	0.81(0.57 to 1.08)	1.4(0.94 to 1.96)
Kyrgyzstan	Liver cancer due to hepatitis B	9(6 to 12)	27(18 to 37)	1.92(1.33 to 2.61)	0.29(0.21 to 0.4)	0.54(0.38 to 0.77)	0.86(0.49 to 1.26)
Kyrgyzstan	Liver cancer due to hepatitis C	15(11 to 19)	42(31 to 55)	1.82(1.34 to 2.37)	0.52(0.39 to 0.65)	1.06(0.77 to 1.37)	1.04(0.68 to 1.42)
Kyrgyzstan	Liver cancer due to NASH	2(2 to 3)	7(5 to 11)	2.28(1.59 to 3.06)	0.08(0.05 to 0.11)	0.18(0.12 to 0.26)	1.34(0.89 to 1.91)
Kyrgyzstan	Liver cancer due to other causes	4(2 to 6)	6(4 to 8)	0.4(-0.15 to 1.51)	0.09(0.06 to 0.12)	0.11(0.08 to 0.15)	0.22(-0.2 to 0.93)
Lao People's Democratic Republic	Liver cancer	223(153 to 303)	308(228 to 398)	0.39(-0.1 to 1.1)	10.47(7.33 to 14.06)	7.11(5.4 to 9.01)	-0.32(-0.54 to -0.02)
Lao People's Democratic Republic	Liver cancer due to alcohol use	45(26 to 71)	72(45 to 109)	0.62(0.05 to 1.48)	2.12(1.28 to 3.3)	1.69(1.1 to 2.51)	-0.2(-0.48 to 0.2)
Lao People's Democratic Republic	Liver cancer due to hepatitis B	99(62 to 147)	129(86 to 181)	0.3(-0.2 to 1.06)	4.31(2.71 to 6.29)	2.64(1.79 to 3.7)	-0.39(-0.61 to -0.06)
Lao People's Democratic Republic	Liver cancer due to hepatitis C	55(32 to 86)	74(47 to 104)	0.33(-0.09 to 0.96)	2.98(1.78 to 4.46)	1.98(1.31 to 2.75)	-0.34(-0.53 to -0.05)
Lao People's Democratic Republic	Liver cancer due to NASH	15(8 to 24)	23(15 to 35)	0.6(0.05 to 1.4)	0.74(0.43 to 1.16)	0.58(0.37 to 0.85)	-0.21(-0.47 to 0.14)

Lao People's Democratic Republic	Liver cancer due to other causes	9(5 to 16)	11(7 to 16)	0.16(-0.35 to 0.91)	0.33(0.19 to 0.53)	0.21(0.14 to 0.31)	-0.35(-0.59 to -0.03)
Latvia	Liver cancer	60(56 to 64)	106(89 to 127)	0.76(0.45 to 1.11)	1.7(1.59 to 1.81)	2.7(2.25 to 3.29)	0.59(0.3 to 0.91)
Latvia	Liver cancer due to alcohol use	21(16 to 27)	45(33 to 59)	1.12(0.68 to 1.63)	0.59(0.45 to 0.75)	1.16(0.84 to 1.52)	0.95(0.55 to 1.45)
Latvia	Liver cancer due to hepatitis B	14(10 to 19)	19(12 to 27)	0.31(0.03 to 0.64)	0.4(0.29 to 0.55)	0.53(0.36 to 0.77)	0.33(0.05 to 0.67)
Latvia	Liver cancer due to hepatitis C	17(12 to 23)	30(20 to 41)	0.73(0.38 to 1.12)	0.47(0.34 to 0.62)	0.69(0.46 to 0.95)	0.45(0.16 to 0.78)
Latvia	Liver cancer due to NASH	5(3 to 6)	9(6 to 14)	1.04(0.57 to 1.6)	0.13(0.09 to 0.18)	0.22(0.15 to 0.33)	0.75(0.36 to 1.22)
Latvia	Liver cancer due to other causes	3(2 to 4)	3(2 to 5)	0.13(-0.17 to 0.56)	0.1(0.07 to 0.14)	0.1(0.07 to 0.14)	0.01(-0.3 to 0.53)
Lebanon	Liver cancer	79(65 to 98)	155(114 to 216)	0.95(0.35 to 1.88)	3.56(2.95 to 4.32)	2.97(2.19 to 4.14)	-0.17(-0.42 to 0.22)
Lebanon	Liver cancer due to alcohol use	10(6 to 15)	20(11 to 33)	0.98(0.29 to 2.08)	0.46(0.29 to 0.68)	0.38(0.22 to 0.63)	-0.16(-0.44 to 0.26)
Lebanon	Liver cancer due to hepatitis B	42(31 to 54)	76(52 to 107)	0.8(0.21 to 1.67)	1.8(1.36 to 2.31)	1.45(1 to 2.06)	-0.2(-0.46 to 0.19)
Lebanon	Liver cancer due to hepatitis C	17(11 to 25)	36(23 to 58)	1.14(0.4 to 2.4)	0.85(0.56 to 1.21)	0.7(0.44 to 1.11)	-0.18(-0.45 to 0.28)
Lebanon	Liver cancer due to NASH	6(4 to 8)	15(10 to 24)	1.67(0.71 to 3.15)	0.28(0.18 to 0.4)	0.29(0.18 to 0.47)	0.06(-0.32 to 0.64)
Lebanon	Liver cancer due to other causes	5(3 to 6)	8(5 to 12)	0.66(0.12 to 1.49)	0.18(0.12 to 0.26)	0.15(0.09 to 0.22)	-0.2(-0.45 to 0.18)
Lesotho	Liver cancer	53(33 to 111)	197(80 to 312)	2.71(-0.1 to 7)	5.42(3.38 to 11.16)	15.17(6.45 to 23.71)	1.8(-0.27 to 4.82)

Lesotho	Liver cancer due to alcohol use	10(5 to 24)	46(15 to 78)	3.64(-0.09 to 9.46)	1(0.49 to 2.44)	3.56(1.22 to 6.01)	2.57(-0.28 to 6.92)
Lesotho	Liver cancer due to hepatitis B	21(11 to 49)	81(27 to 141)	2.92(-0.24 to 8.38)	1.94(1.03 to 4.51)	5.62(1.95 to 9.5)	1.9(-0.41 to 5.6)
Lesotho	Liver cancer due to hepatitis C	15(9 to 28)	44(20 to 74)	1.88(-0.02 to 4.96)	1.76(1.05 to 3.13)	3.97(1.91 to 6.49)	1.26(-0.19 to 3.5)
Lesotho	Liver cancer due to NASH	4(2 to 8)	17(7 to 29)	2.97(0.18 to 7.42)	0.46(0.25 to 0.88)	1.4(0.62 to 2.34)	2.04(-0.03 to 5.21)
Lesotho	Liver cancer due to other causes	3(2 to 5)	9(4 to 15)	2.01(-0.12 to 5.37)	0.26(0.15 to 0.47)	0.62(0.27 to 1.07)	1.37(-0.28 to 4.02)
Liberia	Liver cancer	81(64 to 104)	108(77 to 157)	0.34(-0.07 to 0.9)	7.22(5.74 to 9.41)	5.29(3.89 to 7.78)	-0.27(-0.48 to 0.02)
Liberia	Liver cancer due to alcohol use	15(9 to 24)	19(11 to 31)	0.29(-0.13 to 0.92)	1.35(0.84 to 2.11)	1.01(0.61 to 1.65)	-0.25(-0.48 to 0.11)
Liberia	Liver cancer due to hepatitis B	41(30 to 55)	54(35 to 85)	0.33(-0.1 to 1.02)	3.57(2.6 to 4.78)	2.38(1.57 to 3.65)	-0.33(-0.54 to -0.02)
Liberia	Liver cancer due to hepatitis C	14(9 to 20)	18(11 to 28)	0.31(-0.05 to 0.83)	1.39(0.91 to 1.94)	1.09(0.67 to 1.64)	-0.22(-0.42 to 0.07)
Liberia	Liver cancer due to NASH	6(4 to 9)	10(6 to 16)	0.7(0.19 to 1.49)	0.56(0.37 to 0.84)	0.55(0.35 to 0.85)	-0.03(-0.31 to 0.38)
Liberia	Liver cancer due to other causes	6(4 to 8)	7(4 to 10)	0.22(-0.17 to 0.77)	0.35(0.24 to 0.49)	0.26(0.16 to 0.39)	-0.26(-0.46 to 0.02)
Libya	Liver cancer	101(76 to 130)	256(190 to 351)	1.54(0.64 to 2.81)	5.37(4.06 to 6.94)	5.05(3.79 to 6.86)	-0.06(-0.38 to 0.42)
Libya	Liver cancer due to alcohol use	9(5 to 14)	24(14 to 40)	1.71(0.73 to 3.17)	0.5(0.3 to 0.79)	0.5(0.28 to 0.8)	0(-0.35 to 0.52)
Libya	Liver cancer due to hepatitis B	39(27 to 54)	97(64 to 144)	1.48(0.54 to 2.87)	1.93(1.33 to 2.7)	1.71(1.14 to 2.5)	-0.12(-0.43 to 0.36)

Libya	Liver cancer due to hepatitis C	33(22 to 48)	84(55 to 123)	1.56(0.63 to 2.81)	1.96(1.29 to 2.83)	1.83(1.21 to 2.66)	-0.06(-0.4 to 0.39)
Libya	Liver cancer due to NASH	11(7 to 16)	33(22 to 51)	2.11(1.02 to 3.7)	0.6(0.38 to 0.91)	0.69(0.44 to 1.06)	0.14(-0.26 to 0.71)
Libya	Liver cancer due to other causes	9(6 to 13)	18(12 to 27)	0.9(0.23 to 1.87)	0.38(0.25 to 0.56)	0.33(0.22 to 0.5)	-0.13(-0.44 to 0.31)
Lithuania	Liver cancer	74(70 to 79)	169(134 to 206)	1.28(0.81 to 1.77)	1.66(1.56 to 1.76)	3.03(2.38 to 3.7)	0.82(0.44 to 1.23)
Lithuania	Liver cancer due to alcohol use	26(19 to 33)	76(53 to 99)	1.87(1.19 to 2.65)	0.58(0.43 to 0.73)	1.36(0.96 to 1.78)	1.33(0.76 to 1.98)
Lithuania	Liver cancer due to hepatitis B	18(13 to 24)	30(20 to 45)	0.73(0.33 to 1.15)	0.39(0.29 to 0.53)	0.61(0.4 to 0.9)	0.54(0.18 to 0.94)
Lithuania	Liver cancer due to hepatitis C	21(15 to 28)	45(30 to 62)	1.1(0.66 to 1.58)	0.47(0.34 to 0.6)	0.73(0.48 to 1)	0.56(0.24 to 0.91)
Lithuania	Liver cancer due to NASH	5(4 to 8)	13(9 to 19)	1.44(0.91 to 2.06)	0.12(0.08 to 0.17)	0.22(0.14 to 0.32)	0.84(0.43 to 1.32)
Lithuania	Liver cancer due to other causes	4(2 to 5)	5(3 to 7)	0.37(0 to 0.89)	0.1(0.06 to 0.13)	0.11(0.08 to 0.15)	0.12(-0.2 to 0.63)
Luxembourg	Liver cancer	16(15 to 18)	36(30 to 45)	1.24(0.84 to 1.75)	2.96(2.75 to 3.2)	3.62(2.94 to 4.5)	0.22(0 to 0.5)
Luxembourg	Liver cancer due to alcohol use	7(5 to 8)	14(10 to 19)	1.1(0.64 to 1.66)	1.2(0.91 to 1.48)	1.41(1 to 1.91)	0.18(-0.08 to 0.49)
Luxembourg	Liver cancer due to hepatitis B	2(1 to 3)	4(2 to 6)	1.03(0.62 to 1.54)	0.37(0.25 to 0.53)	0.41(0.26 to 0.63)	0.13(-0.09 to 0.41)
Luxembourg	Liver cancer due to hepatitis C	6(4 to 8)	14(10 to 20)	1.42(0.98 to 1.93)	1.07(0.8 to 1.38)	1.37(0.98 to 1.88)	0.28(0.05 to 0.55)
Luxembourg	Liver cancer due to NASH	1(1 to 1)	2(2 to 4)	1.57(1.04 to 2.24)	0.17(0.11 to 0.25)	0.24(0.15 to 0.35)	0.39(0.12 to 0.75)

Luxembourg	Liver cancer due to other causes	1(1 to 1)	2(1 to 2)	1.17(0.73 to 1.7)	0.15(0.11 to 0.2)	0.18(0.12 to 0.25)	0.16(-0.06 to 0.44)
Madagascar	Liver cancer	168(121 to 287)	289(196 to 435)	0.71(0.21 to 1.4)	3.07(2.12 to 5.36)	2.62(1.8 to 4.02)	-0.15(-0.4 to 0.18)
Madagascar	Liver cancer due to alcohol use	37(19 to 79)	64(35 to 117)	0.73(0.18 to 1.6)	0.75(0.38 to 1.59)	0.63(0.34 to 1.15)	-0.16(-0.41 to 0.23)
Madagascar	Liver cancer due to hepatitis B	56(35 to 100)	96(57 to 159)	0.7(0.14 to 1.46)	0.97(0.59 to 1.75)	0.74(0.43 to 1.25)	-0.24(-0.47 to 0.08)
Madagascar	Liver cancer due to hepatitis C	33(20 to 52)	59(36 to 91)	0.82(0.29 to 1.57)	0.73(0.45 to 1.17)	0.69(0.45 to 1.05)	-0.05(-0.32 to 0.32)
Madagascar	Liver cancer due to NASH	17(10 to 28)	33(20 to 53)	0.95(0.35 to 1.74)	0.35(0.21 to 0.6)	0.34(0.2 to 0.54)	-0.02(-0.3 to 0.37)
Madagascar	Liver cancer due to other causes	25(16 to 39)	36(24 to 52)	0.41(-0.06 to 1.16)	0.27(0.18 to 0.41)	0.22(0.14 to 0.33)	-0.19(-0.42 to 0.13)
Malawi	Liver cancer	166(123 to 228)	243(191 to 301)	0.47(0.05 to 1.05)	3.42(2.62 to 4.81)	3.04(2.43 to 3.74)	-0.11(-0.37 to 0.21)
Malawi	Liver cancer due to alcohol use	29(17 to 51)	52(34 to 74)	0.77(0.16 to 1.6)	0.77(0.45 to 1.33)	0.76(0.5 to 1.08)	-0.02(-0.34 to 0.41)
Malawi	Liver cancer due to hepatitis B	44(29 to 69)	61(41 to 87)	0.4(-0.07 to 1.05)	0.99(0.63 to 1.61)	0.75(0.5 to 1.1)	-0.24(-0.5 to 0.08)
Malawi	Liver cancer due to hepatitis C	27(17 to 41)	45(30 to 63)	0.7(0.24 to 1.37)	0.81(0.52 to 1.18)	0.77(0.52 to 1.07)	-0.05(-0.29 to 0.29)
Malawi	Liver cancer due to NASH	14(9 to 22)	26(17 to 38)	0.83(0.31 to 1.54)	0.4(0.25 to 0.6)	0.4(0.27 to 0.58)	0.02(-0.26 to 0.39)
Malawi	Liver cancer due to other causes	52(29 to 88)	59(41 to 79)	0.13(-0.36 to 0.92)	0.46(0.3 to 0.67)	0.35(0.26 to 0.47)	-0.24(-0.5 to 0.11)
Malaysia	Liver cancer	532(456 to 622)	1628(1240 to 2103)	2.06(1.21 to 3.21)	5.78(4.93 to 6.75)	6.22(4.78 to 7.94)	0.08(-0.22 to 0.45)

Malaysia	Liver cancer due to alcohol use	67(43 to 99)	223(137 to 348)	2.34(1.31 to 3.74)	0.76(0.49 to 1.14)	0.85(0.52 to 1.31)	0.12(-0.21 to 0.57)
Malaysia	Liver cancer due to hepatitis B	318(252 to 386)	922(655 to 1218)	1.89(1.02 to 3.02)	3.28(2.57 to 4.07)	3.36(2.39 to 4.44)	0.02(-0.28 to 0.41)
Malaysia	Liver cancer due to hepatitis C	86(55 to 123)	264(169 to 395)	2.06(1.28 to 3.19)	1.07(0.69 to 1.49)	1.12(0.73 to 1.65)	0.05(-0.22 to 0.42)
Malaysia	Liver cancer due to NASH	43(30 to 62)	180(117 to 267)	3.13(1.96 to 4.83)	0.51(0.34 to 0.74)	0.73(0.48 to 1.08)	0.43(0.03 to 0.99)
Malaysia	Liver cancer due to other causes	17(12 to 23)	40(26 to 59)	1.36(0.71 to 2.23)	0.15(0.1 to 0.22)	0.15(0.1 to 0.22)	-0.03(-0.3 to 0.34)
Maldives	Liver cancer	6(4 to 9)	16(13 to 20)	1.72(0.74 to 3.22)	7.33(5.14 to 10.73)	5.63(4.46 to 6.89)	-0.23(-0.49 to 0.15)
Maldives	Liver cancer due to alcohol use	1(1 to 2)	4(2 to 5)	1.88(0.78 to 3.54)	1.53(0.88 to 2.62)	1.26(0.82 to 1.83)	-0.17(-0.47 to 0.27)
Maldives	Liver cancer due to hepatitis B	3(2 to 5)	7(5 to 9)	1.44(0.5 to 2.98)	3.06(1.9 to 4.88)	2.08(1.45 to 2.84)	-0.32(-0.56 to 0.05)
Maldives	Liver cancer due to hepatitis C	1(1 to 2)	4(3 to 5)	1.97(0.83 to 3.75)	2(1.15 to 3.25)	1.58(1.04 to 2.2)	-0.21(-0.47 to 0.22)
Maldives	Liver cancer due to NASH	0(0 to 1)	2(1 to 2)	2.85(1.34 to 5.24)	0.53(0.31 to 0.91)	0.56(0.37 to 0.86)	0.05(-0.32 to 0.65)
Maldives	Liver cancer due to other causes	0(0 to 0)	0(0 to 1)	0.96(0.22 to 2.15)	0.2(0.12 to 0.36)	0.15(0.1 to 0.22)	-0.26(-0.52 to 0.12)
Mali	Liver cancer	711(585 to 852)	1372(1011 to 1818)	0.93(0.4 to 1.61)	15.7(12.98 to 18.83)	15.03(11.25 to 19.52)	-0.04(-0.3 to 0.28)
Mali	Liver cancer due to alcohol use	106(65 to 158)	228(139 to 351)	1.14(0.46 to 2.05)	2.46(1.53 to 3.65)	2.59(1.59 to 3.9)	0.05(-0.28 to 0.46)
Mali	Liver cancer due to hepatitis B	280(198 to 370)	535(346 to 775)	0.91(0.32 to 1.64)	6.03(4.31 to 8.08)	5.43(3.47 to 7.84)	-0.1(-0.37 to 0.24)

Mali	Liver cancer due to hepatitis C	218(147 to 302)	426(277 to 606)	0.95(0.44 to 1.58)	5.5(3.82 to 7.37)	5.28(3.56 to 7.22)	-0.04(-0.28 to 0.24)
Mali	Liver cancer due to NASH	38(25 to 55)	91(58 to 140)	1.42(0.66 to 2.28)	0.91(0.61 to 1.33)	1.06(0.68 to 1.59)	0.17(-0.18 to 0.58)
Mali	Liver cancer due to other causes	68(48 to 96)	93(62 to 133)	0.35(-0.14 to 1.07)	0.8(0.57 to 1.13)	0.67(0.44 to 0.99)	-0.16(-0.41 to 0.16)
Malta	Liver cancer	7(6 to 8)	20(18 to 23)	1.93(1.49 to 2.41)	1.64(1.5 to 1.78)	2.2(1.93 to 2.49)	0.34(0.15 to 0.56)
Malta	Liver cancer due to alcohol use	2(2 to 3)	7(5 to 10)	2.18(1.61 to 2.82)	0.53(0.38 to 0.68)	0.78(0.56 to 1.02)	0.48(0.22 to 0.78)
Malta	Liver cancer due to hepatitis B	1(1 to 1)	2(2 to 4)	1.41(1.01 to 1.88)	0.23(0.16 to 0.34)	0.29(0.19 to 0.42)	0.24(0.04 to 0.47)
Malta	Liver cancer due to hepatitis C	3(2 to 4)	8(6 to 11)	1.9(1.45 to 2.45)	0.68(0.51 to 0.86)	0.85(0.63 to 1.1)	0.26(0.07 to 0.49)
Malta	Liver cancer due to NASH	0(0 to 1)	2(1 to 2)	2.46(1.79 to 3.3)	0.1(0.07 to 0.15)	0.16(0.11 to 0.23)	0.53(0.26 to 0.89)
Malta	Liver cancer due to other causes	0(0 to 1)	1(1 to 1)	1.33(0.89 to 1.84)	0.1(0.07 to 0.14)	0.12(0.09 to 0.17)	0.21(0.01 to 0.44)
Marshall Islands	Liver cancer	2(2 to 3)	4(3 to 5)	0.86(0.34 to 1.55)	12.14(9.19 to 15.86)	10.57(7.64 to 14.31)	-0.13(-0.36 to 0.19)
Marshall Islands	Liver cancer due to alcohol use	0(0 to 0)	0(0 to 1)	0.99(0.38 to 1.85)	1.44(0.83 to 2.34)	1.35(0.8 to 2.18)	-0.06(-0.33 to 0.3)
Marshall Islands	Liver cancer due to hepatitis B	1(1 to 2)	2(1 to 3)	0.87(0.3 to 1.62)	6.42(4.5 to 9.11)	5.38(3.58 to 7.88)	-0.16(-0.4 to 0.15)
Marshall Islands	Liver cancer due to hepatitis C	0(0 to 1)	1(0 to 1)	0.71(0.21 to 1.36)	2.88(1.84 to 4.3)	2.47(1.52 to 3.65)	-0.14(-0.38 to 0.16)
Marshall Islands	Liver cancer due to NASH	0(0 to 0)	0(0 to 0)	1.1(0.47 to 1.97)	0.89(0.56 to 1.36)	0.92(0.59 to 1.4)	0.04(-0.26 to 0.42)

Marshall Islands	Liver cancer due to other causes	0(0 to 0)	0(0 to 0)	0.68(0.2 to 1.34)	0.52(0.32 to 0.76)	0.45(0.27 to 0.67)	-0.14(-0.37 to 0.19)
Mauritania	Liver cancer	75(60 to 91)	90(66 to 117)	0.21(-0.13 to 0.63)	7.31(5.92 to 8.82)	4.43(3.3 to 5.68)	-0.39(-0.56 to -0.19)
Mauritania	Liver cancer due to alcohol use	10(6 to 16)	13(8 to 20)	0.27(-0.09 to 0.76)	1.04(0.66 to 1.59)	0.67(0.4 to 1.03)	-0.35(-0.53 to -0.12)
Mauritania	Liver cancer due to hepatitis B	39(29 to 50)	43(29 to 61)	0.11(-0.24 to 0.55)	3.71(2.79 to 4.77)	2.02(1.39 to 2.83)	-0.46(-0.62 to -0.26)
Mauritania	Liver cancer due to hepatitis C	14(8 to 20)	17(11 to 25)	0.26(-0.07 to 0.68)	1.51(0.96 to 2.13)	0.94(0.61 to 1.36)	-0.37(-0.52 to -0.16)
Mauritania	Liver cancer due to NASH	7(4 to 10)	11(7 to 16)	0.63(0.16 to 1.23)	0.68(0.46 to 1)	0.55(0.35 to 0.81)	-0.19(-0.4 to 0.1)
Mauritania	Liver cancer due to other causes	5(3 to 6)	6(4 to 8)	0.19(-0.21 to 0.67)	0.36(0.24 to 0.51)	0.23(0.15 to 0.35)	-0.35(-0.55 to -0.11)
Mauritius	Liver cancer	14(13 to 15)	34(26 to 44)	1.46(0.87 to 2.28)	1.9(1.75 to 2.04)	2(1.55 to 2.57)	0.06(-0.19 to 0.4)
Mauritius	Liver cancer due to alcohol use	3(2 to 4)	8(5 to 12)	1.8(1.01 to 2.95)	0.4(0.28 to 0.54)	0.47(0.3 to 0.7)	0.16(-0.15 to 0.63)
Mauritius	Liver cancer due to hepatitis B	5(4 to 7)	11(7 to 16)	1.06(0.5 to 1.81)	0.7(0.53 to 0.9)	0.64(0.43 to 0.93)	-0.08(-0.33 to 0.24)
Mauritius	Liver cancer due to hepatitis C	4(3 to 5)	10(6 to 14)	1.67(1.04 to 2.45)	0.55(0.39 to 0.7)	0.59(0.39 to 0.83)	0.07(-0.18 to 0.37)
Mauritius	Liver cancer due to NASH	1(1 to 2)	4(3 to 6)	2.24(1.42 to 3.33)	0.18(0.12 to 0.25)	0.24(0.16 to 0.36)	0.35(0.01 to 0.81)
Mauritius	Liver cancer due to other causes	1(0 to 1)	1(1 to 1)	0.64(0.2 to 1.17)	0.07(0.05 to 0.09)	0.07(0.04 to 0.09)	-0.06(-0.28 to 0.23)
Mexico	Liver cancer	1010(972 to 1039)	4183(3606 to 4792)	3.14(2.57 to 3.76)	2.43(2.31 to 2.5)	3.69(3.18 to 4.22)	0.52(0.32 to 0.74)

Mexico	Liver cancer due to alcohol use	262(228 to 298)	1277(1024 to 1562)	3.88(3.12 to 4.69)	0.64(0.56 to 0.73)	1.11(0.89 to 1.36)	0.74(0.47 to 1.02)
Mexico	Liver cancer due to hepatitis B	139(119 to 165)	476(374 to 597)	2.42(1.9 to 3)	0.3(0.26 to 0.36)	0.4(0.32 to 0.5)	0.32(0.12 to 0.53)
Mexico	Liver cancer due to hepatitis C	417(376 to 458)	1736(1460 to 2060)	3.16(2.61 to 3.8)	1.1(0.99 to 1.2)	1.56(1.31 to 1.86)	0.42(0.24 to 0.64)
Mexico	Liver cancer due to NASH	83(71 to 96)	431(349 to 525)	4.22(3.51 to 5.04)	0.2(0.18 to 0.24)	0.38(0.31 to 0.47)	0.87(0.62 to 1.15)
Mexico	Liver cancer due to other causes	109(98 to 120)	263(219 to 313)	1.43(1.06 to 1.83)	0.18(0.16 to 0.2)	0.23(0.19 to 0.27)	0.28(0.11 to 0.47)
Micronesia (Federated States of)	Liver cancer	5(4 to 7)	8(5 to 11)	0.41(-0.1 to 1.1)	11.58(8.62 to 14.99)	10.7(7.24 to 14.74)	-0.08(-0.38 to 0.33)
Micronesia (Federated States of)	Liver cancer due to alcohol use	1(0 to 1)	1(1 to 2)	0.45(-0.09 to 1.23)	1.4(0.81 to 2.24)	1.34(0.75 to 2.1)	-0.04(-0.37 to 0.43)
Micronesia (Federated States of)	Liver cancer due to hepatitis B	3(2 to 4)	4(3 to 6)	0.41(-0.14 to 1.15)	5.95(4.04 to 8.28)	5.31(3.29 to 7.72)	-0.11(-0.44 to 0.33)
Micronesia (Federated States of)	Liver cancer due to hepatitis C	1(1 to 2)	1(1 to 2)	0.33(-0.14 to 0.99)	2.77(1.72 to 4.09)	2.53(1.54 to 3.79)	-0.09(-0.38 to 0.32)
Micronesia (Federated States of)	Liver cancer due to NASH	0(0 to 1)	1(0 to 1)	0.65(0.06 to 1.53)	0.96(0.61 to 1.49)	1.08(0.65 to 1.67)	0.12(-0.25 to 0.7)
Micronesia (Federated States of)	Liver cancer due to other causes	0(0 to 0)	0(0 to 1)	0.24(-0.19 to 0.81)	0.5(0.31 to 0.74)	0.44(0.26 to 0.72)	-0.11(-0.41 to 0.33)

Monaco	Liver cancer	2(2 to 3)	7(6 to 9)	2.2(1.39 to 3.26)	3.24(2.53 to 4)	7.63(6.05 to 9.39)	1.35(0.74 to 2.18)
Monaco	Liver cancer due to alcohol use	1(1 to 1)	2(2 to 3)	2.24(1.43 to 3.38)	1.11(0.74 to 1.53)	2.68(1.84 to 3.71)	1.41(0.78 to 2.29)
Monaco	Liver cancer due to hepatitis B	0(0 to 0)	1(1 to 1)	1.84(1.08 to 2.89)	0.48(0.31 to 0.73)	1.02(0.66 to 1.5)	1.11(0.54 to 1.96)
Monaco	Liver cancer due to hepatitis C	1(1 to 1)	3(2 to 4)	2.22(1.39 to 3.36)	1.23(0.84 to 1.71)	2.93(2.06 to 3.93)	1.38(0.76 to 2.26)
Monaco	Liver cancer due to NASH	0(0 to 0)	1(0 to 1)	2.57(1.56 to 3.91)	0.23(0.15 to 0.36)	0.63(0.4 to 0.96)	1.68(0.95 to 2.67)
Monaco	Liver cancer due to other causes	0(0 to 0)	0(0 to 0)	2.08(1.28 to 3.23)	0.18(0.12 to 0.26)	0.37(0.24 to 0.54)	1.05(0.51 to 1.76)
Mongolia	Liver cancer	693(556 to 838)	2365(1819 to 3039)	2.41(1.49 to 3.72)	66.77(54.18 to 79.9)	115.23(91.48 to 142.48)	0.73(0.3 to 1.3)
Mongolia	Liver cancer due to alcohol use	183(116 to 258)	729(477 to 1054)	2.98(1.81 to 4.51)	17.55(11.24 to 24.44)	34.2(23.11 to 47.83)	0.95(0.4 to 1.68)
Mongolia	Liver cancer due to hepatitis B	237(165 to 327)	705(468 to 1024)	1.98(1.1 to 3.23)	21.66(15.15 to 30.17)	28.23(18.92 to 40.83)	0.3(-0.05 to 0.8)
Mongolia	Liver cancer due to hepatitis C	215(146 to 299)	697(473 to 971)	2.24(1.4 to 3.46)	21.95(15.12 to 29.98)	40.31(28.58 to 53.28)	0.84(0.38 to 1.47)
Mongolia	Liver cancer due to NASH	35(22 to 53)	154(98 to 232)	3.43(2.2 to 5.07)	3.51(2.3 to 5.19)	8.72(5.64 to 12.87)	1.48(0.82 to 2.38)
Mongolia	Liver cancer due to other causes	23(15 to 35)	80(51 to 121)	2.48(1.47 to 3.92)	2.1(1.34 to 3.17)	3.78(2.39 to 5.74)	0.8(0.27 to 1.52)
Montenegro	Liver cancer	39(32 to 45)	60(48 to 74)	0.55(0.17 to 1.16)	6.26(5.17 to 7.22)	6.09(4.93 to 7.48)	-0.03(-0.26 to 0.36)

Montenegro	Liver cancer due to alcohol use	16(12 to 21)	25(18 to 34)	0.55(0.12 to 1.25)	2.65(1.96 to 3.41)	2.53(1.8 to 3.42)	-0.04(-0.3 to 0.37)
Montenegro	Liver cancer due to hepatitis B	9(6 to 13)	12(8 to 18)	0.34(-0.01 to 0.88)	1.42(0.98 to 2.01)	1.28(0.85 to 1.84)	-0.1(-0.32 to 0.24)
Montenegro	Liver cancer due to hepatitis C	9(6 to 13)	15(10 to 22)	0.68(0.26 to 1.33)	1.51(1.02 to 2.07)	1.55(1.07 to 2.18)	0.03(-0.23 to 0.43)
Montenegro	Liver cancer due to NASH	3(2 to 4)	5(3 to 7)	0.88(0.37 to 1.67)	0.45(0.3 to 0.64)	0.52(0.35 to 0.75)	0.16(-0.14 to 0.65)
Montenegro	Liver cancer due to other causes	1(1 to 2)	2(1 to 3)	0.31(-0.04 to 0.81)	0.23(0.17 to 0.33)	0.21(0.14 to 0.3)	-0.1(-0.32 to 0.21)
Morocco	Liver cancer	258(189 to 318)	652(497 to 800)	1.52(0.8 to 2.48)	2.07(1.48 to 2.57)	2.31(1.77 to 2.79)	0.11(-0.2 to 0.51)
Morocco	Liver cancer due to alcohol use	28(17 to 44)	76(45 to 117)	1.69(0.85 to 2.88)	0.22(0.13 to 0.35)	0.26(0.16 to 0.41)	0.19(-0.18 to 0.7)
Morocco	Liver cancer due to hepatitis B	100(70 to 138)	230(153 to 326)	1.3(0.6 to 2.31)	0.74(0.51 to 1.02)	0.74(0.49 to 1.06)	0.01(-0.29 to 0.43)
Morocco	Liver cancer due to hepatitis C	88(57 to 122)	231(154 to 314)	1.61(0.91 to 2.62)	0.79(0.51 to 1.07)	0.88(0.6 to 1.19)	0.12(-0.18 to 0.53)
Morocco	Liver cancer due to NASH	23(14 to 35)	76(49 to 112)	2.25(1.26 to 3.58)	0.2(0.12 to 0.3)	0.28(0.18 to 0.41)	0.42(0.01 to 0.97)
Morocco	Liver cancer due to other causes	19(13 to 26)	40(25 to 59)	1.11(0.53 to 1.92)	0.13(0.08 to 0.18)	0.13(0.09 to 0.2)	0.05(-0.24 to 0.47)
Mozambique	Liver cancer	139(101 to 225)	459(319 to 609)	2.29(0.52 to 4.25)	2.3(1.66 to 3.71)	3.99(2.79 to 5.24)	0.73(-0.21 to 1.82)
Mozambique	Liver cancer due to alcohol use	26(14 to 52)	99(58 to 146)	2.79(0.44 to 5.66)	0.48(0.27 to 0.95)	0.99(0.57 to 1.45)	1.06(-0.22 to 2.57)
Mozambique	Liver cancer due to hepatitis B	56(34 to 98)	183(116 to 261)	2.29(0.33 to 4.66)	0.88(0.54 to 1.55)	1.49(0.95 to 2.15)	0.7(-0.34 to 1.87)

Mozambique	Liver cancer due to hepatitis C	22(13 to 35)	61(36 to 90)	1.77(0.52 to 3.35)	0.48(0.29 to 0.76)	0.72(0.45 to 1.07)	0.51(-0.16 to 1.35)
Mozambique	Liver cancer due to NASH	13(8 to 21)	44(29 to 66)	2.3(0.78 to 4.17)	0.26(0.16 to 0.43)	0.47(0.31 to 0.7)	0.77(-0.03 to 1.78)
Mozambique	Liver cancer due to other causes	22(14 to 36)	72(45 to 112)	2.22(0.63 to 5.02)	0.2(0.13 to 0.3)	0.32(0.22 to 0.46)	0.59(-0.07 to 1.5)
Myanmar	Liver cancer	679(492 to 998)	1955(1629 to 2325)	1.88(0.94 to 3.05)	3.03(2.23 to 4.41)	4.44(3.76 to 5.26)	0.46(0.01 to 1.02)
Myanmar	Liver cancer due to alcohol use	117(71 to 198)	394(254 to 572)	2.36(1.24 to 3.93)	0.54(0.33 to 0.89)	0.89(0.59 to 1.27)	0.66(0.13 to 1.39)
Myanmar	Liver cancer due to hepatitis B	249(161 to 382)	645(457 to 891)	1.59(0.65 to 2.72)	1.04(0.68 to 1.58)	1.35(0.95 to 1.88)	0.3(-0.14 to 0.84)
Myanmar	Liver cancer due to hepatitis C	197(122 to 305)	594(409 to 794)	2.01(1.11 to 3.27)	1.02(0.66 to 1.51)	1.46(1.02 to 1.93)	0.43(0.02 to 0.99)
Myanmar	Liver cancer due to NASH	56(34 to 88)	204(137 to 298)	2.65(1.46 to 4.18)	0.27(0.17 to 0.43)	0.48(0.33 to 0.71)	0.77(0.22 to 1.45)
Myanmar	Liver cancer due to other causes	59(35 to 106)	119(82 to 170)	1.02(0.05 to 2.42)	0.16(0.1 to 0.26)	0.25(0.17 to 0.35)	0.58(-0.09 to 1.49)
Namibia	Liver cancer	14(9 to 25)	49(37 to 64)	2.45(0.86 to 5.06)	1.98(1.25 to 3.47)	3.46(2.66 to 4.39)	0.75(-0.03 to 1.99)
Namibia	Liver cancer due to alcohol use	3(1 to 6)	12(7 to 17)	3.31(1.08 to 7.21)	0.38(0.18 to 0.84)	0.84(0.54 to 1.2)	1.24(0.1 to 3.25)
Namibia	Liver cancer due to hepatitis B	5(3 to 10)	17(11 to 24)	2.41(0.63 to 5.8)	0.65(0.34 to 1.32)	1.08(0.72 to 1.55)	0.67(-0.19 to 2.25)
Namibia	Liver cancer due to hepatitis C	4(3 to 7)	13(9 to 19)	2.04(0.88 to 3.88)	0.67(0.42 to 1.09)	1.05(0.71 to 1.44)	0.56(-0.03 to 1.45)
Namibia	Liver cancer due to NASH	1(1 to 2)	4(3 to 6)	2.57(1.08 to 4.96)	0.18(0.1 to 0.32)	0.32(0.21 to 0.47)	0.82(0.09 to 1.98)

Namibia	Liver cancer due to other causes	1(1 to 2)	3(2 to 4)	1.95(0.77 to 3.94)	0.11(0.06 to 0.18)	0.17(0.11 to 0.25)	0.61(-0.05 to 1.64)
Nauru	Liver cancer	0(0 to 1)	0(0 to 1)	-0.02(-0.3 to 0.39)	10.89(7.88 to 13.83)	9.39(6.7 to 12.73)	-0.14(-0.36 to 0.16)
Nauru	Liver cancer due to alcohol use	0(0 to 0)	0(0 to 0)	0.05(-0.28 to 0.54)	1.31(0.79 to 2.01)	1.24(0.71 to 1.9)	-0.05(-0.33 to 0.34)
Nauru	Liver cancer due to hepatitis B	0(0 to 0)	0(0 to 0)	-0.04(-0.33 to 0.41)	5.66(3.84 to 7.66)	4.6(3.09 to 6.58)	-0.19(-0.41 to 0.15)
Nauru	Liver cancer due to hepatitis C	0(0 to 0)	0(0 to 0)	-0.09(-0.35 to 0.28)	2.53(1.67 to 3.58)	2.18(1.38 to 3.28)	-0.14(-0.36 to 0.14)
Nauru	Liver cancer due to NASH	0(0 to 0)	0(0 to 0)	0.14(-0.19 to 0.6)	0.91(0.59 to 1.34)	0.94(0.6 to 1.45)	0.03(-0.23 to 0.42)
Nauru	Liver cancer due to other causes	0(0 to 0)	0(0 to 0)	-0.03(-0.27 to 0.29)	0.48(0.31 to 0.72)	0.42(0.26 to 0.68)	-0.12(-0.35 to 0.19)
Nepal	Liver cancer	188(142 to 242)	490(354 to 701)	1.61(0.75 to 2.56)	1.97(1.53 to 2.47)	2.36(1.71 to 3.39)	0.2(-0.18 to 0.64)
Nepal	Liver cancer due to alcohol use	43(29 to 62)	134(79 to 220)	2.1(0.97 to 3.56)	0.51(0.34 to 0.73)	0.64(0.38 to 1.04)	0.26(-0.2 to 0.89)
Nepal	Liver cancer due to hepatitis B	48(32 to 69)	110(67 to 174)	1.29(0.46 to 2.29)	0.48(0.31 to 0.68)	0.49(0.3 to 0.78)	0.03(-0.34 to 0.46)
Nepal	Liver cancer due to hepatitis C	53(34 to 76)	164(109 to 253)	2.12(1.12 to 3.35)	0.69(0.46 to 0.97)	0.87(0.58 to 1.3)	0.25(-0.13 to 0.71)
Nepal	Liver cancer due to NASH	13(9 to 20)	48(30 to 74)	2.57(1.43 to 4.08)	0.16(0.1 to 0.24)	0.24(0.15 to 0.37)	0.49(0.02 to 1.09)
Nepal	Liver cancer due to other causes	31(19 to 52)	34(24 to 53)	0.1(-0.38 to 1.13)	0.13(0.09 to 0.2)	0.13(0.09 to 0.2)	-0.03(-0.39 to 0.66)
Netherlands	Liver cancer	275(259 to 289)	939(855 to 1016)	2.41(2.16 to 2.7)	1.39(1.31 to 1.46)	2.75(2.53 to 2.97)	0.98(0.84 to 1.14)

Netherlands	Liver cancer due to alcohol use	111(88 to 135)	379(289 to 470)	2.4(2.03 to 2.79)	0.56(0.44 to 0.67)	1.11(0.86 to 1.36)	0.99(0.79 to 1.21)
Netherlands	Liver cancer due to hepatitis B	34(23 to 48)	105(70 to 151)	2.08(1.72 to 2.52)	0.18(0.12 to 0.25)	0.34(0.24 to 0.48)	0.91(0.7 to 1.15)
Netherlands	Liver cancer due to hepatitis C	94(70 to 120)	330(244 to 418)	2.52(2.16 to 2.93)	0.46(0.35 to 0.59)	0.92(0.69 to 1.17)	0.99(0.8 to 1.21)
Netherlands	Liver cancer due to NASH	20(14 to 29)	80(54 to 113)	2.96(2.38 to 3.61)	0.1(0.07 to 0.14)	0.23(0.16 to 0.32)	1.29(0.97 to 1.64)
Netherlands	Liver cancer due to other causes	16(12 to 22)	46(31 to 64)	1.9(1.4 to 2.32)	0.09(0.07 to 0.12)	0.16(0.12 to 0.21)	0.71(0.46 to 0.92)
New Zealand	Liver cancer	82(76 to 88)	280(259 to 302)	2.44(2.08 to 2.84)	2.12(1.98 to 2.28)	3.72(3.44 to 4)	0.75(0.58 to 0.95)
New Zealand	Liver cancer due to alcohol use	34(29 to 38)	106(92 to 120)	2.16(1.79 to 2.59)	0.87(0.75 to 0.98)	1.41(1.23 to 1.6)	0.63(0.43 to 0.84)
New Zealand	Liver cancer due to hepatitis B	14(12 to 17)	42(35 to 51)	1.98(1.63 to 2.37)	0.38(0.31 to 0.45)	0.61(0.51 to 0.73)	0.62(0.44 to 0.83)
New Zealand	Liver cancer due to hepatitis C	23(20 to 26)	88(76 to 101)	2.86(2.44 to 3.34)	0.59(0.51 to 0.68)	1.11(0.95 to 1.27)	0.89(0.7 to 1.11)
New Zealand	Liver cancer due to NASH	7(6 to 8)	31(26 to 37)	3.71(3.17 to 4.28)	0.17(0.14 to 0.2)	0.4(0.34 to 0.47)	1.36(1.11 to 1.63)
New Zealand	Liver cancer due to other causes	4(4 to 5)	13(11 to 15)	1.94(1.64 to 2.25)	0.12(0.11 to 0.14)	0.19(0.17 to 0.22)	0.58(0.44 to 0.74)
Nicaragua	Liver cancer	51(45 to 58)	172(140 to 210)	2.37(1.63 to 3.24)	3.24(2.81 to 3.7)	4.1(3.37 to 4.96)	0.27(-0.02 to 0.6)
Nicaragua	Liver cancer due to alcohol use	14(9 to 18)	53(36 to 75)	2.89(1.9 to 4.3)	0.92(0.62 to 1.24)	1.26(0.85 to 1.75)	0.37(0.02 to 0.84)
Nicaragua	Liver cancer due to hepatitis B	10(7 to 14)	26(17 to 37)	1.59(0.95 to 2.39)	0.56(0.38 to 0.81)	0.55(0.36 to 0.81)	-0.02(-0.26 to 0.27)

Nicaragua	Liver cancer due to hepatitis C	19(14 to 24)	68(49 to 90)	2.6(1.81 to 3.52)	1.34(0.99 to 1.71)	1.72(1.27 to 2.25)	0.29(0.01 to 0.61)
Nicaragua	Liver cancer due to NASH	3(2 to 5)	14(10 to 21)	3.42(2.38 to 4.71)	0.21(0.15 to 0.31)	0.35(0.24 to 0.51)	0.65(0.26 to 1.15)
Nicaragua	Liver cancer due to other causes	5(4 to 6)	11(8 to 15)	1.04(0.54 to 1.6)	0.21(0.16 to 0.28)	0.22(0.15 to 0.31)	0.06(-0.18 to 0.35)
Niger	Liver cancer	20(16 to 25)	51(38 to 68)	1.54(0.82 to 2.43)	0.71(0.55 to 0.87)	0.65(0.49 to 0.84)	-0.08(-0.32 to 0.21)
Niger	Liver cancer due to alcohol use	3(2 to 4)	8(5 to 12)	1.61(0.83 to 2.74)	0.11(0.07 to 0.17)	0.11(0.06 to 0.16)	-0.05(-0.31 to 0.33)
Niger	Liver cancer due to hepatitis B	11(8 to 15)	26(18 to 37)	1.37(0.68 to 2.31)	0.37(0.27 to 0.48)	0.31(0.21 to 0.43)	-0.15(-0.38 to 0.16)
Niger	Liver cancer due to hepatitis C	3(2 to 5)	9(6 to 13)	1.72(1.02 to 2.63)	0.15(0.1 to 0.22)	0.14(0.09 to 0.2)	-0.03(-0.27 to 0.27)
Niger	Liver cancer due to NASH	1(1 to 2)	4(2 to 6)	1.99(1.14 to 3.04)	0.05(0.03 to 0.07)	0.05(0.03 to 0.08)	0.07(-0.22 to 0.44)
Niger	Liver cancer due to other causes	2(1 to 2)	4(3 to 6)	1.92(1.02 to 3.2)	0.03(0.02 to 0.05)	0.03(0.02 to 0.05)	0.02(-0.25 to 0.37)
Nigeria	Liver cancer	1646(1263 to 2063)	2920(2284 to 3704)	0.77(0.3 to 1.44)	3.57(2.71 to 4.54)	3.57(2.87 to 4.44)	0(-0.26 to 0.39)
Nigeria	Liver cancer due to alcohol use	295(207 to 405)	614(451 to 812)	1.08(0.48 to 2.01)	0.7(0.5 to 0.96)	0.81(0.61 to 1.07)	0.16(-0.17 to 0.64)
Nigeria	Liver cancer due to hepatitis B	646(475 to 842)	1129(841 to 1480)	0.75(0.24 to 1.48)	1.39(1.01 to 1.81)	1.3(0.99 to 1.7)	-0.06(-0.32 to 0.33)
Nigeria	Liver cancer due to hepatitis C	334(241 to 449)	608(459 to 781)	0.82(0.3 to 1.58)	0.88(0.64 to 1.18)	0.86(0.66 to 1.07)	-0.03(-0.29 to 0.39)
Nigeria	Liver cancer due to NASH	132(94 to 177)	287(218 to 375)	1.18(0.58 to 2.07)	0.33(0.23 to 0.44)	0.38(0.3 to 0.49)	0.18(-0.13 to 0.66)

Nigeria	Liver cancer due to other causes	240(183 to 312)	282(191 to 377)	0.18(-0.24 to 0.63)	0.26(0.2 to 0.33)	0.21(0.16 to 0.27)	-0.21(-0.42 to 0.07)
Niue	Liver cancer	0(0 to 0)	0(0 to 0)	-0.13(-0.36 to 0.19)	8.32(6.66 to 10.36)	7.34(5.67 to 9.36)	-0.12(-0.36 to 0.2)
Niue	Liver cancer due to alcohol use	0(0 to 0)	0(0 to 0)	0.02(-0.28 to 0.44)	1.02(0.63 to 1.52)	1.03(0.65 to 1.54)	0(-0.29 to 0.43)
Niue	Liver cancer due to hepatitis B	0(0 to 0)	0(0 to 0)	-0.14(-0.39 to 0.21)	4.21(3.07 to 5.51)	3.54(2.52 to 4.84)	-0.16(-0.41 to 0.19)
Niue	Liver cancer due to hepatitis C	0(0 to 0)	0(0 to 0)	-0.22(-0.43 to 0.06)	1.99(1.33 to 2.81)	1.66(1.09 to 2.4)	-0.17(-0.39 to 0.12)
Niue	Liver cancer due to NASH	0(0 to 0)	0(0 to 0)	0.07(-0.23 to 0.45)	0.73(0.48 to 1.1)	0.81(0.52 to 1.19)	0.12(-0.2 to 0.53)
Niue	Liver cancer due to other causes	0(0 to 0)	0(0 to 0)	-0.23(-0.45 to 0.08)	0.37(0.24 to 0.53)	0.31(0.19 to 0.46)	-0.17(-0.41 to 0.18)
North Macedonia	Liver cancer	168(151 to 185)	280(222 to 355)	0.66(0.28 to 1.14)	9.13(8.14 to 10.05)	8.82(7.01 to 11.1)	-0.03(-0.25 to 0.23)
North Macedonia	Liver cancer due to alcohol use	61(45 to 79)	105(70 to 148)	0.71(0.27 to 1.25)	3.3(2.44 to 4.21)	3.2(2.15 to 4.47)	-0.03(-0.28 to 0.25)
North Macedonia	Liver cancer due to hepatitis B	44(31 to 60)	63(41 to 94)	0.43(0.07 to 0.88)	2.28(1.62 to 3.11)	1.97(1.3 to 2.9)	-0.14(-0.34 to 0.12)
North Macedonia	Liver cancer due to hepatitis C	44(31 to 60)	78(51 to 112)	0.76(0.35 to 1.28)	2.52(1.77 to 3.34)	2.55(1.73 to 3.55)	0.01(-0.22 to 0.29)
North Macedonia	Liver cancer due to NASH	12(8 to 16)	24(16 to 36)	1.07(0.56 to 1.75)	0.67(0.46 to 0.95)	0.79(0.52 to 1.16)	0.19(-0.1 to 0.58)
North Macedonia	Liver cancer due to other causes	7(5 to 9)	10(6 to 15)	0.42(0.05 to 0.88)	0.36(0.26 to 0.5)	0.32(0.21 to 0.47)	-0.12(-0.33 to 0.16)
Northern Mariana Islands	Liver cancer	1(1 to 2)	4(3 to 5)	2.08(1.25 to 3.16)	6.93(5.45 to 8.53)	7.84(6.5 to 9.45)	0.13(-0.12 to 0.46)

Northern Mariana Islands	Liver cancer due to alcohol use	0(0 to 0)	1(0 to 1)	2.84(1.71 to 4.39)	0.77(0.48 to 1.15)	0.98(0.63 to 1.43)	0.27(-0.03 to 0.67)
Northern Mariana Islands	Liver cancer due to hepatitis B	1(1 to 1)	3(2 to 3)	1.96(1.08 to 3.24)	3.53(2.52 to 4.67)	4.26(3.21 to 5.54)	0.21(-0.1 to 0.59)
Northern Mariana Islands	Liver cancer due to hepatitis C	0(0 to 0)	1(0 to 1)	2.24(1.42 to 3.32)	1.64(1.08 to 2.25)	1.54(1 to 2.13)	-0.06(-0.26 to 0.18)
Northern Mariana Islands	Liver cancer due to NASH	0(0 to 0)	0(0 to 1)	2.36(1.41 to 3.62)	0.7(0.48 to 1.02)	0.8(0.54 to 1.15)	0.14(-0.12 to 0.46)
Northern Mariana Islands	Liver cancer due to other causes	0(0 to 0)	0(0 to 0)	0.99(0.34 to 1.8)	0.29(0.19 to 0.43)	0.26(0.17 to 0.38)	-0.1(-0.31 to 0.17)
Norway	Liver cancer	103(98 to 107)	232(207 to 261)	1.25(1.02 to 1.54)	1.54(1.47 to 1.6)	2.45(2.2 to 2.76)	0.59(0.43 to 0.8)
Norway	Liver cancer due to alcohol use	32(28 to 36)	77(64 to 91)	1.41(1.14 to 1.81)	0.48(0.42 to 0.54)	0.82(0.69 to 0.97)	0.72(0.52 to 1.01)
Norway	Liver cancer due to hepatitis B	15(12 to 17)	29(23 to 36)	0.96(0.76 to 1.25)	0.24(0.21 to 0.29)	0.33(0.27 to 0.41)	0.37(0.22 to 0.57)
Norway	Liver cancer due to hepatitis C	44(39 to 49)	98(84 to 113)	1.23(1.02 to 1.49)	0.62(0.56 to 0.7)	0.99(0.85 to 1.13)	0.58(0.43 to 0.77)
Norway	Liver cancer due to NASH	7(6 to 8)	16(13 to 20)	1.43(1.19 to 1.71)	0.1(0.08 to 0.11)	0.17(0.14 to 0.2)	0.76(0.58 to 0.96)
Norway	Liver cancer due to other causes	6(5 to 7)	12(10 to 14)	1.02(0.83 to 1.25)	0.1(0.09 to 0.11)	0.14(0.12 to 0.16)	0.37(0.25 to 0.53)
Oman	Liver cancer	30(21 to 40)	83(67 to 103)	1.78(0.95 to 3.07)	4.36(3.03 to 5.79)	4.75(3.98 to 5.69)	0.09(-0.23 to 0.63)
Oman	Liver cancer due to alcohol use	3(2 to 4)	9(5 to 13)	2.06(1.07 to 3.66)	0.43(0.24 to 0.67)	0.52(0.33 to 0.81)	0.21(-0.19 to 0.93)

Oman	Liver cancer due to hepatitis B	13(8 to 19)	36(25 to 50)	1.83(0.93 to 3.34)	1.61(1.01 to 2.38)	1.6(1.13 to 2.24)	-0.01(-0.32 to 0.5)
Oman	Liver cancer due to hepatitis C	10(6 to 14)	23(16 to 32)	1.44(0.71 to 2.58)	1.73(1.1 to 2.47)	1.76(1.23 to 2.3)	0.02(-0.29 to 0.53)
Oman	Liver cancer due to NASH	2(1 to 3)	9(6 to 13)	3.35(1.99 to 5.55)	0.34(0.19 to 0.54)	0.59(0.4 to 0.87)	0.76(0.19 to 1.73)
Oman	Liver cancer due to other causes	3(2 to 4)	6(5 to 9)	1.31(0.74 to 2.18)	0.26(0.17 to 0.38)	0.29(0.2 to 0.4)	0.1(-0.2 to 0.56)
Pakistan	Liver cancer	2060(1428 to 2752)	3912(3165 to 4833)	0.9(0.39 to 1.81)	3.47(2.31 to 4.72)	3.46(2.75 to 4.3)	0(-0.27 to 0.48)
Pakistan	Liver cancer due to alcohol use	310(195 to 435)	618(462 to 826)	0.99(0.4 to 2.19)	0.54(0.34 to 0.76)	0.54(0.41 to 0.72)	0.01(-0.28 to 0.59)
Pakistan	Liver cancer due to hepatitis B	321(217 to 447)	684(528 to 883)	1.13(0.5 to 2.28)	0.5(0.33 to 0.71)	0.51(0.39 to 0.66)	0(-0.29 to 0.54)
Pakistan	Liver cancer due to hepatitis C	1094(705 to 1511)	1941(1486 to 2483)	0.77(0.27 to 1.66)	2.01(1.28 to 2.79)	1.94(1.48 to 2.5)	-0.03(-0.29 to 0.42)
Pakistan	Liver cancer due to NASH	142(93 to 203)	314(243 to 411)	1.2(0.55 to 2.32)	0.25(0.16 to 0.36)	0.29(0.22 to 0.38)	0.16(-0.16 to 0.7)
Pakistan	Liver cancer due to other causes	192(145 to 263)	355(278 to 449)	0.85(0.27 to 1.69)	0.17(0.13 to 0.23)	0.18(0.14 to 0.23)	0.05(-0.23 to 0.48)
Palau	Liver cancer	1(1 to 1)	2(2 to 3)	1.22(0.48 to 2.4)	9.78(6.71 to 13.43)	9.79(7.59 to 12.53)	0(-0.32 to 0.5)
Palau	Liver cancer due to alcohol use	0(0 to 0)	0(0 to 0)	1.39(0.56 to 2.8)	1.29(0.73 to 2.11)	1.36(0.85 to 2.04)	0.05(-0.3 to 0.65)
Palau	Liver cancer due to hepatitis B	1(0 to 1)	1(1 to 2)	1.18(0.42 to 2.41)	5.74(3.72 to 8.18)	5.55(4.02 to 7.45)	-0.03(-0.36 to 0.49)
Palau	Liver cancer due to hepatitis C	0(0 to 0)	0(0 to 0)	1.12(0.43 to 2.18)	1.74(1.06 to 2.6)	1.69(1.11 to 2.38)	-0.03(-0.33 to 0.43)

Palau	Liver cancer due to NASH	0(0 to 0)	0(0 to 0)	1.78(0.85 to 3.31)	0.68(0.4 to 1.07)	0.88(0.56 to 1.3)	0.29(-0.11 to 0.93)
Palau	Liver cancer due to other causes	0(0 to 0)	0(0 to 0)	0.92(0.26 to 1.84)	0.33(0.2 to 0.51)	0.31(0.2 to 0.47)	-0.06(-0.37 to 0.4)
Palestine	Liver cancer	74(55 to 99)	142(118 to 172)	0.91(0.36 to 1.68)	9(6.8 to 11.92)	6.61(5.56 to 7.88)	-0.26(-0.48 to 0.02)
Palestine	Liver cancer due to alcohol use	6(4 to 10)	13(8 to 20)	1.07(0.44 to 2.01)	0.78(0.44 to 1.2)	0.62(0.38 to 0.93)	-0.21(-0.45 to 0.14)
Palestine	Liver cancer due to hepatitis B	25(17 to 36)	50(37 to 67)	1.03(0.43 to 1.89)	2.81(1.88 to 4.08)	2.02(1.46 to 2.75)	-0.28(-0.49 to 0.02)
Palestine	Liver cancer due to hepatitis C	31(21 to 43)	55(39 to 71)	0.79(0.23 to 1.51)	3.99(2.75 to 5.47)	2.89(2.13 to 3.75)	-0.28(-0.5 to 0.02)
Palestine	Liver cancer due to NASH	7(4 to 11)	14(10 to 20)	1.09(0.44 to 1.99)	0.85(0.52 to 1.31)	0.7(0.48 to 1.02)	-0.17(-0.44 to 0.18)
Palestine	Liver cancer due to other causes	6(4 to 9)	10(7 to 14)	0.7(0.19 to 1.43)	0.57(0.35 to 0.89)	0.39(0.26 to 0.55)	-0.32(-0.53 to -0.02)
Panama	Liver cancer	52(48 to 56)	126(95 to 162)	1.44(0.85 to 2.2)	3.45(3.18 to 3.72)	3.06(2.32 to 3.93)	-0.11(-0.33 to 0.16)
Panama	Liver cancer due to alcohol use	16(12 to 20)	44(29 to 63)	1.76(1.04 to 2.66)	1.07(0.78 to 1.38)	1.06(0.71 to 1.54)	-0.01(-0.26 to 0.32)
Panama	Liver cancer due to hepatitis B	10(7 to 13)	18(12 to 27)	0.91(0.4 to 1.58)	0.59(0.42 to 0.83)	0.44(0.28 to 0.66)	-0.26(-0.46 to -0.01)
Panama	Liver cancer due to hepatitis C	19(14 to 24)	47(32 to 65)	1.43(0.83 to 2.18)	1.34(1.01 to 1.69)	1.13(0.77 to 1.59)	-0.16(-0.37 to 0.09)
Panama	Liver cancer due to NASH	3(2 to 5)	10(7 to 16)	2.11(1.29 to 3.18)	0.23(0.15 to 0.33)	0.25(0.16 to 0.38)	0.12(-0.18 to 0.49)
Panama	Liver cancer due to other causes	4(3 to 5)	7(5 to 10)	0.83(0.37 to 1.4)	0.21(0.16 to 0.29)	0.17(0.11 to 0.24)	-0.2(-0.4 to 0.07)

Papua New Guinea	Liver cancer	25(19 to 32)	67(51 to 87)	1.67(0.93 to 2.68)	1.52(1.19 to 1.93)	1.67(1.31 to 2.12)	0.1(-0.2 to 0.48)
Papua New Guinea	Liver cancer due to alcohol use	3(2 to 5)	9(5 to 14)	1.9(1 to 3.26)	0.19(0.12 to 0.3)	0.23(0.15 to 0.37)	0.21(-0.16 to 0.73)
Papua New Guinea	Liver cancer due to hepatitis B	12(9 to 17)	32(22 to 45)	1.59(0.83 to 2.64)	0.69(0.48 to 0.94)	0.72(0.5 to 1)	0.06(-0.25 to 0.45)
Papua New Guinea	Liver cancer due to hepatitis C	6(4 to 9)	16(10 to 24)	1.67(0.94 to 2.66)	0.47(0.3 to 0.66)	0.51(0.34 to 0.73)	0.09(-0.19 to 0.46)
Papua New Guinea	Liver cancer due to NASH	1(1 to 2)	4(3 to 7)	2.1(1.24 to 3.25)	0.1(0.06 to 0.16)	0.13(0.08 to 0.2)	0.27(-0.07 to 0.71)
Papua New Guinea	Liver cancer due to other causes	2(1 to 2)	5(3 to 7)	1.52(0.9 to 2.33)	0.07(0.05 to 0.1)	0.07(0.05 to 0.11)	0.09(-0.17 to 0.45)
Paraguay	Liver cancer	55(47 to 62)	117(88 to 152)	1.15(0.58 to 1.92)	2.47(2.13 to 2.84)	2.19(1.64 to 2.82)	-0.12(-0.35 to 0.19)
Paraguay	Liver cancer due to alcohol use	18(13 to 24)	40(25 to 57)	1.17(0.54 to 2.03)	0.85(0.59 to 1.11)	0.74(0.47 to 1.07)	-0.13(-0.38 to 0.22)
Paraguay	Liver cancer due to hepatitis B	9(7 to 13)	21(13 to 32)	1.19(0.55 to 2.11)	0.4(0.27 to 0.57)	0.36(0.23 to 0.56)	-0.09(-0.36 to 0.29)
Paraguay	Liver cancer due to hepatitis C	20(15 to 26)	44(30 to 61)	1.22(0.63 to 2)	0.96(0.71 to 1.24)	0.85(0.58 to 1.17)	-0.12(-0.35 to 0.19)
Paraguay	Liver cancer due to NASH	3(2 to 4)	7(4 to 10)	1.45(0.79 to 2.35)	0.12(0.08 to 0.18)	0.12(0.08 to 0.19)	-0.01(-0.28 to 0.36)
Paraguay	Liver cancer due to other causes	4(3 to 5)	6(4 to 9)	0.46(0.04 to 0.96)	0.14(0.1 to 0.18)	0.11(0.07 to 0.15)	-0.23(-0.44 to 0.05)
Peru	Liver cancer	744(641 to 854)	874(651 to 1150)	0.17(-0.17 to 0.63)	6.21(5.34 to 7.13)	2.74(2.04 to 3.61)	-0.56(-0.69 to -0.39)
Peru	Liver cancer due to alcohol use	230(162 to 308)	300(194 to 424)	0.3(-0.08 to 0.81)	2.03(1.43 to 2.72)	0.95(0.61 to 1.35)	-0.53(-0.67 to -0.35)

Peru	Liver cancer due to hepatitis B	346(268 to 439)	353(239 to 506)	0.02(-0.29 to 0.44)	2.79(2.12 to 3.58)	1.1(0.74 to 1.58)	-0.61(-0.73 to -0.45)
Peru	Liver cancer due to hepatitis C	50(30 to 74)	69(42 to 107)	0.4(-0.03 to 0.98)	0.46(0.28 to 0.68)	0.22(0.13 to 0.34)	-0.53(-0.67 to -0.34)
Peru	Liver cancer due to NASH	54(37 to 78)	87(54 to 127)	0.61(0.11 to 1.29)	0.48(0.32 to 0.69)	0.27(0.17 to 0.4)	-0.43(-0.61 to -0.2)
Peru	Liver cancer due to other causes	65(49 to 84)	64(43 to 93)	-0.01(-0.3 to 0.4)	0.45(0.31 to 0.62)	0.2(0.13 to 0.29)	-0.55(-0.68 to -0.37)
Philippines	Liver cancer	3151(2362 to 3839)	5280(4269 to 6454)	0.68(0.22 to 1.43)	10.04(7.53 to 12.27)	6.58(5.35 to 7.98)	-0.35(-0.52 to -0.05)
Philippines	Liver cancer due to alcohol use	658(445 to 873)	1244(929 to 1628)	0.89(0.32 to 1.9)	2.18(1.48 to 2.87)	1.57(1.18 to 2.03)	-0.28(-0.5 to 0.1)
Philippines	Liver cancer due to hepatitis B	1588(1160 to 2010)	2364(1849 to 2975)	0.49(0.06 to 1.19)	4.52(3.25 to 5.75)	2.69(2.1 to 3.37)	-0.4(-0.57 to -0.11)
Philippines	Liver cancer due to hepatitis C	580(424 to 735)	1079(858 to 1341)	0.86(0.4 to 1.66)	2.33(1.74 to 2.92)	1.56(1.26 to 1.92)	-0.33(-0.49 to -0.07)
Philippines	Liver cancer due to NASH	199(150 to 257)	406(321 to 507)	1.04(0.53 to 1.86)	0.71(0.53 to 0.91)	0.55(0.43 to 0.68)	-0.22(-0.42 to 0.09)
Philippines	Liver cancer due to other causes	126(103 to 151)	187(153 to 230)	0.49(0.18 to 0.89)	0.31(0.24 to 0.38)	0.21(0.17 to 0.26)	-0.31(-0.46 to -0.08)
Poland	Liver cancer	3242(3102 to 3341)	1455(1224 to 1719)	-0.55(-0.62 to -0.47)	7.57(7.21 to 7.81)	2.06(1.73 to 2.44)	-0.73(-0.77 to -0.68)
Poland	Liver cancer due to alcohol use	1167(1033 to 1305)	650(529 to 800)	-0.44(-0.54 to -0.32)	2.67(2.37 to 2.99)	0.91(0.74 to 1.13)	-0.66(-0.72 to -0.58)
Poland	Liver cancer due to hepatitis B	719(613 to 841)	260(204 to 336)	-0.64(-0.7 to -0.56)	1.66(1.43 to 1.93)	0.39(0.31 to 0.5)	-0.77(-0.81 to -0.72)
Poland	Liver cancer due to hepatitis C	965(839 to 1089)	376(297 to 460)	-0.61(-0.67 to -0.54)	2.31(2.03 to 2.59)	0.51(0.41 to 0.62)	-0.78(-0.81 to -0.74)

Poland	Liver cancer due to NASH	266(225 to 311)	124(99 to 157)	-0.53(-0.6 to -0.45)	0.63(0.53 to 0.74)	0.17(0.14 to 0.22)	-0.73(-0.77 to -0.68)
Poland	Liver cancer due to other causes	126(108 to 145)	44(35 to 54)	-0.65(-0.71 to -0.59)	0.3(0.26 to 0.35)	0.07(0.06 to 0.09)	-0.76(-0.79 to -0.72)
Portugal	Liver cancer	264(250 to 279)	1050(962 to 1136)	2.98(2.62 to 3.35)	1.92(1.82 to 2.03)	4.59(4.19 to 4.97)	1.39(1.17 to 1.62)
Portugal	Liver cancer due to alcohol use	101(76 to 125)	409(303 to 514)	3.05(2.57 to 3.59)	0.71(0.55 to 0.88)	1.83(1.36 to 2.3)	1.57(1.26 to 1.89)
Portugal	Liver cancer due to hepatitis B	35(24 to 50)	125(84 to 183)	2.55(2.11 to 3.03)	0.27(0.19 to 0.37)	0.63(0.43 to 0.91)	1.37(1.07 to 1.67)
Portugal	Liver cancer due to hepatitis C	99(75 to 126)	404(302 to 512)	3.08(2.64 to 3.55)	0.72(0.56 to 0.91)	1.63(1.21 to 2.09)	1.27(1.04 to 1.52)
Portugal	Liver cancer due to NASH	15(10 to 22)	68(46 to 98)	3.59(2.96 to 4.37)	0.11(0.08 to 0.15)	0.28(0.19 to 0.4)	1.61(1.24 to 2.02)
Portugal	Liver cancer due to other causes	14(10 to 19)	43(29 to 62)	2.1(1.56 to 2.62)	0.12(0.09 to 0.15)	0.22(0.15 to 0.3)	0.84(0.54 to 1.15)
Puerto Rico	Liver cancer	255(239 to 269)	195(146 to 250)	-0.24(-0.42 to -0.02)	6.97(6.5 to 7.36)	2.74(2.04 to 3.55)	-0.61(-0.7 to -0.49)
Puerto Rico	Liver cancer due to alcohol use	90(66 to 114)	67(44 to 96)	-0.25(-0.45 to -0.01)	2.42(1.79 to 3.07)	0.95(0.62 to 1.36)	-0.61(-0.71 to -0.48)
Puerto Rico	Liver cancer due to hepatitis B	65(46 to 87)	43(27 to 64)	-0.33(-0.51 to -0.12)	1.78(1.29 to 2.39)	0.67(0.43 to 0.99)	-0.62(-0.72 to -0.5)
Puerto Rico	Liver cancer due to hepatitis C	60(41 to 80)	48(31 to 69)	-0.2(-0.39 to 0.01)	1.66(1.15 to 2.2)	0.62(0.4 to 0.91)	-0.63(-0.72 to -0.52)
Puerto Rico	Liver cancer due to NASH	27(18 to 37)	27(18 to 40)	0.02(-0.25 to 0.34)	0.72(0.51 to 1.01)	0.36(0.23 to 0.54)	-0.5(-0.63 to -0.33)
Puerto Rico	Liver cancer due to other causes	14(10 to 20)	10(6 to 14)	-0.33(-0.51 to -0.12)	0.39(0.27 to 0.54)	0.15(0.1 to 0.21)	-0.62(-0.72 to -0.5)

Qatar	Liver cancer	14(11 to 19)	91(63 to 124)	5.27(3.01 to 8.49)	17.37(12.99 to 22.54)	15.88(11.76 to 20.79)	-0.09(-0.37 to 0.3)
Qatar	Liver cancer due to alcohol use	2(1 to 2)	10(6 to 17)	5.92(3.28 to 10.17)	1.87(1.08 to 2.96)	1.8(1.02 to 2.9)	-0.04(-0.38 to 0.5)
Qatar	Liver cancer due to hepatitis B	6(4 to 9)	37(24 to 57)	5.13(2.78 to 8.79)	5.44(3.54 to 8.22)	4.36(2.71 to 6.73)	-0.2(-0.48 to 0.19)
Qatar	Liver cancer due to hepatitis C	4(3 to 6)	26(16 to 38)	4.93(2.96 to 7.77)	6.94(4.47 to 9.83)	6.48(4.41 to 9.04)	-0.07(-0.35 to 0.3)
Qatar	Liver cancer due to NASH	2(1 to 2)	12(7 to 19)	6.65(4.04 to 10.51)	2.23(1.38 to 3.44)	2.53(1.56 to 3.92)	0.13(-0.22 to 0.64)
Qatar	Liver cancer due to other causes	1(1 to 1)	5(3 to 8)	4.37(2.52 to 7.1)	0.89(0.54 to 1.35)	0.71(0.42 to 1.16)	-0.2(-0.46 to 0.15)
Republic of Korea	Liver cancer	3413(2831 to 4174)	14477(12937 to 16039)	3.24(2.33 to 4.42)	11.04(9.25 to 13.4)	16.2(14.47 to 17.94)	0.47(0.16 to 0.86)
Republic of Korea	Liver cancer due to alcohol use	439(281 to 648)	2599(1791 to 3641)	4.92(3.44 to 6.78)	1.47(0.95 to 2.18)	2.88(2 to 4.03)	0.96(0.5 to 1.54)
Republic of Korea	Liver cancer due to hepatitis B	2220(1771 to 2766)	7876(6389 to 9396)	2.55(1.69 to 3.6)	6.65(5.29 to 8.21)	8.76(7.19 to 10.42)	0.32(0.02 to 0.7)
Republic of Korea	Liver cancer due to hepatitis C	487(326 to 693)	2707(1879 to 3574)	4.55(3.16 to 6.41)	1.98(1.35 to 2.75)	3.08(2.18 to 4.04)	0.56(0.19 to 1.02)
Republic of Korea	Liver cancer due to NASH	149(98 to 214)	837(565 to 1222)	4.62(3.1 to 6.71)	0.56(0.38 to 0.81)	0.94(0.64 to 1.37)	0.68(0.26 to 1.22)
Republic of Korea	Liver cancer due to other causes	117(78 to 166)	458(299 to 654)	2.92(1.82 to 4.32)	0.38(0.25 to 0.55)	0.53(0.36 to 0.74)	0.4(0.05 to 0.83)
Republic of Moldova	Liver cancer	74(70 to 79)	138(117 to 162)	0.85(0.58 to 1.17)	1.7(1.6 to 1.81)	2.4(2.03 to 2.81)	0.41(0.2 to 0.64)
Republic of Moldova	Liver cancer due to alcohol use	38(31 to 44)	67(53 to 84)	0.79(0.49 to 1.14)	0.85(0.71 to 0.99)	1.16(0.92 to 1.45)	0.36(0.13 to 0.62)

Republic of Moldova	Liver cancer due to hepatitis B	14(10 to 20)	24(16 to 35)	0.65(0.35 to 1.03)	0.32(0.23 to 0.44)	0.42(0.29 to 0.61)	0.34(0.1 to 0.62)
Republic of Moldova	Liver cancer due to hepatitis C	15(11 to 20)	33(23 to 46)	1.21(0.82 to 1.63)	0.36(0.26 to 0.49)	0.57(0.39 to 0.79)	0.56(0.3 to 0.82)
Republic of Moldova	Liver cancer due to NASH	4(3 to 5)	9(6 to 13)	1.62(1.07 to 2.27)	0.09(0.06 to 0.12)	0.16(0.11 to 0.23)	0.89(0.52 to 1.34)
Republic of Moldova	Liver cancer due to other causes	4(2 to 5)	4(3 to 6)	0.08(-0.25 to 0.6)	0.08(0.05 to 0.12)	0.08(0.06 to 0.11)	-0.01(-0.31 to 0.45)
Romania	Liver cancer	504(452 to 565)	1112(907 to 1344)	1.21(0.77 to 1.7)	1.85(1.66 to 2.06)	3.08(2.5 to 3.75)	0.67(0.33 to 1.03)
Romania	Liver cancer due to alcohol use	206(153 to 258)	499(360 to 650)	1.42(0.9 to 1.99)	0.74(0.56 to 0.91)	1.37(0.98 to 1.79)	0.86(0.46 to 1.32)
Romania	Liver cancer due to hepatitis B	119(84 to 160)	209(141 to 305)	0.77(0.38 to 1.22)	0.43(0.31 to 0.57)	0.63(0.43 to 0.9)	0.48(0.16 to 0.85)
Romania	Liver cancer due to hepatitis C	128(90 to 172)	284(193 to 394)	1.22(0.78 to 1.73)	0.48(0.35 to 0.64)	0.74(0.5 to 1.03)	0.52(0.23 to 0.86)
Romania	Liver cancer due to NASH	32(22 to 46)	88(59 to 129)	1.75(1.14 to 2.43)	0.12(0.09 to 0.17)	0.23(0.16 to 0.34)	0.94(0.5 to 1.4)
Romania	Liver cancer due to other causes	20(15 to 26)	33(21 to 47)	0.64(0.27 to 1.11)	0.08(0.06 to 0.1)	0.11(0.08 to 0.15)	0.34(0.07 to 0.68)
Russian Federation	Liver cancer	3101(2963 to 3268)	6826(5812 to 8145)	1.2(0.9 to 1.56)	1.76(1.68 to 1.85)	2.97(2.53 to 3.55)	0.69(0.46 to 0.96)
Russian Federation	Liver cancer due to alcohol use	1041(905 to 1185)	2550(2034 to 3200)	1.45(1.06 to 1.92)	0.57(0.5 to 0.65)	1.09(0.87 to 1.37)	0.91(0.61 to 1.27)
Russian Federation	Liver cancer due to hepatitis B	816(694 to 965)	1503(1150 to 1935)	0.84(0.56 to 1.2)	0.45(0.39 to 0.53)	0.68(0.53 to 0.88)	0.5(0.27 to 0.79)
Russian Federation	Liver cancer due to hepatitis C	823(708 to 937)	1913(1585 to 2318)	1.32(1.01 to 1.65)	0.47(0.41 to 0.53)	0.8(0.66 to 0.97)	0.7(0.48 to 0.94)

Russian Federation	Liver cancer due to NASH	218(185 to 259)	588(478 to 728)	1.7(1.35 to 2.11)	0.12(0.11 to 0.15)	0.25(0.21 to 0.31)	1.03(0.76 to 1.32)
Russian Federation	Liver cancer due to other causes	204(181 to 229)	271(216 to 336)	0.33(0.13 to 0.54)	0.14(0.13 to 0.16)	0.15(0.11 to 0.19)	0.05(-0.14 to 0.25)
Rwanda	Liver cancer	162(117 to 225)	286(219 to 373)	0.76(0.11 to 1.68)	5.36(3.93 to 7.14)	4.72(3.78 to 5.98)	-0.12(-0.4 to 0.28)
Rwanda	Liver cancer due to alcohol use	48(31 to 73)	86(57 to 125)	0.77(0.14 to 1.73)	1.7(1.12 to 2.51)	1.46(1 to 2.08)	-0.14(-0.42 to 0.26)
Rwanda	Liver cancer due to hepatitis B	46(30 to 69)	79(51 to 115)	0.73(0.01 to 1.79)	1.4(0.92 to 2.07)	1.13(0.74 to 1.62)	-0.19(-0.5 to 0.24)
Rwanda	Liver cancer due to hepatitis C	32(19 to 51)	59(40 to 84)	0.87(0.24 to 1.81)	1.28(0.79 to 1.93)	1.19(0.82 to 1.67)	-0.07(-0.35 to 0.34)
Rwanda	Liver cancer due to NASH	16(9 to 24)	32(20 to 46)	1.03(0.3 to 2.14)	0.57(0.36 to 0.88)	0.58(0.38 to 0.82)	0(-0.32 to 0.46)
Rwanda	Liver cancer due to other causes	21(13 to 34)	30(20 to 45)	0.45(-0.29 to 1.57)	0.41(0.26 to 0.64)	0.36(0.24 to 0.52)	-0.12(-0.48 to 0.39)
Saint Kitts and Nevis	Liver cancer	5(5 to 6)	2(2 to 3)	-0.51(-0.6 to -0.4)	13.47(12.28 to 14.87)	3.88(3.28 to 4.57)	-0.71(-0.76 to -0.65)
Saint Kitts and Nevis	Liver cancer due to alcohol use	2(1 to 2)	1(1 to 1)	-0.48(-0.58 to -0.34)	4.15(3.01 to 5.4)	1.32(0.93 to 1.8)	-0.68(-0.74 to -0.61)
Saint Kitts and Nevis	Liver cancer due to hepatitis B	1(1 to 2)	1(0 to 1)	-0.48(-0.61 to -0.32)	3.81(2.82 to 4.97)	1.01(0.68 to 1.43)	-0.73(-0.79 to -0.67)
Saint Kitts and Nevis	Liver cancer due to hepatitis C	1(1 to 2)	1(0 to 1)	-0.59(-0.67 to -0.49)	3.43(2.46 to 4.5)	0.93(0.62 to 1.28)	-0.73(-0.77 to -0.67)
Saint Kitts and Nevis	Liver cancer due to NASH	0(0 to 1)	0(0 to 0)	-0.49(-0.6 to -0.33)	1.19(0.84 to 1.68)	0.4(0.28 to 0.58)	-0.66(-0.73 to -0.58)
Saint Kitts and Nevis	Liver cancer due to other causes	0(0 to 0)	0(0 to 0)	-0.59(-0.7 to -0.45)	0.89(0.63 to 1.23)	0.22(0.14 to 0.32)	-0.75(-0.81 to -0.68)

Saint Lucia	Liver cancer	6(5 to 6)	5(4 to 6)	-0.14(-0.29 to 0.04)	6.45(5.92 to 7.05)	2.29(1.91 to 2.73)	-0.64(-0.71 to -0.57)
Saint Lucia	Liver cancer due to alcohol use	2(2 to 3)	2(1 to 2)	-0.08(-0.25 to 0.15)	2.34(1.77 to 2.97)	0.9(0.66 to 1.17)	-0.62(-0.69 to -0.53)
Saint Lucia	Liver cancer due to hepatitis B	1(1 to 2)	1(1 to 2)	-0.2(-0.35 to -0.02)	1.66(1.23 to 2.2)	0.55(0.38 to 0.76)	-0.67(-0.73 to -0.6)
Saint Lucia	Liver cancer due to hepatitis C	1(1 to 2)	1(1 to 1)	-0.18(-0.32 to -0.01)	1.55(1.11 to 2.05)	0.51(0.35 to 0.72)	-0.67(-0.73 to -0.6)
Saint Lucia	Liver cancer due to NASH	0(0 to 1)	0(0 to 1)	0.05(-0.16 to 0.32)	0.49(0.34 to 0.69)	0.21(0.15 to 0.3)	-0.57(-0.65 to -0.47)
Saint Lucia	Liver cancer due to other causes	0(0 to 1)	0(0 to 0)	-0.34(-0.47 to -0.17)	0.41(0.3 to 0.56)	0.13(0.09 to 0.18)	-0.69(-0.75 to -0.61)
Saint Vincent and the Grenadines	Liver cancer	6(5 to 6)	4(4 to 5)	-0.24(-0.36 to -0.11)	7.61(7 to 8.26)	3.13(2.72 to 3.61)	-0.59(-0.65 to -0.52)
Saint Vincent and the Grenadines	Liver cancer due to alcohol use	2(1 to 2)	2(1 to 2)	-0.09(-0.25 to 0.1)	2.43(1.78 to 3.16)	1.19(0.88 to 1.52)	-0.51(-0.59 to -0.41)
Saint Vincent and the Grenadines	Liver cancer due to hepatitis B	2(1 to 2)	1(1 to 1)	-0.31(-0.42 to -0.16)	2.18(1.6 to 2.91)	0.81(0.58 to 1.09)	-0.63(-0.69 to -0.55)
Saint Vincent and the Grenadines	Liver cancer due to hepatitis C	1(1 to 2)	1(1 to 1)	-0.34(-0.45 to -0.23)	1.9(1.36 to 2.5)	0.67(0.46 to 0.93)	-0.65(-0.7 to -0.59)
Saint Vincent and the Grenadines	Liver cancer due to NASH	0(0 to 1)	0(0 to 1)	-0.12(-0.28 to 0.07)	0.6(0.42 to 0.85)	0.29(0.2 to 0.41)	-0.52(-0.61 to -0.42)

Saint Vincent and the Grenadines	Liver cancer due to other causes	0(0 to 1)	0(0 to 0)	-0.42(-0.54 to -0.3)	0.5(0.37 to 0.66)	0.18(0.12 to 0.24)	-0.65(-0.71 to -0.58)
Samoa	Liver cancer	6(4 to 7)	8(6 to 10)	0.37(-0.01 to 0.83)	6.19(4.93 to 7.8)	5.06(3.91 to 6.33)	-0.18(-0.4 to 0.08)
Samoa	Liver cancer due to alcohol use	1(0 to 1)	1(1 to 1)	0.35(-0.02 to 0.87)	0.82(0.49 to 1.24)	0.67(0.41 to 1)	-0.18(-0.4 to 0.11)
Samoa	Liver cancer due to hepatitis B	3(2 to 4)	4(3 to 6)	0.35(-0.07 to 0.89)	3.33(2.4 to 4.36)	2.66(1.93 to 3.59)	-0.2(-0.43 to 0.1)
Samoa	Liver cancer due to hepatitis C	1(1 to 2)	1(1 to 2)	0.39(0.04 to 0.84)	1.29(0.84 to 1.86)	1.07(0.69 to 1.55)	-0.17(-0.37 to 0.07)
Samoa	Liver cancer due to NASH	0(0 to 1)	1(0 to 1)	0.54(0.12 to 1.13)	0.5(0.33 to 0.75)	0.46(0.3 to 0.68)	-0.08(-0.31 to 0.24)
Samoa	Liver cancer due to other causes	0(0 to 0)	0(0 to 0)	0.23(-0.13 to 0.72)	0.25(0.16 to 0.36)	0.2(0.13 to 0.29)	-0.2(-0.42 to 0.1)
San Marino	Liver cancer	1(1 to 1)	2(1 to 3)	1.56(0.62 to 2.82)	2.07(1.71 to 2.47)	2.75(1.8 to 3.98)	0.33(-0.17 to 1.03)
San Marino	Liver cancer due to alcohol use	0(0 to 0)	1(0 to 1)	1.54(0.57 to 2.85)	0.76(0.54 to 1.01)	1.05(0.6 to 1.63)	0.39(-0.16 to 1.14)
San Marino	Liver cancer due to hepatitis B	0(0 to 0)	0(0 to 0)	1.2(0.34 to 2.41)	0.28(0.19 to 0.43)	0.35(0.19 to 0.58)	0.24(-0.26 to 0.97)
San Marino	Liver cancer due to hepatitis C	0(0 to 0)	1(0 to 1)	1.68(0.72 to 2.95)	0.77(0.54 to 1.02)	1(0.61 to 1.53)	0.31(-0.16 to 0.96)
San Marino	Liver cancer due to NASH	0(0 to 0)	0(0 to 0)	1.94(0.82 to 3.53)	0.13(0.08 to 0.19)	0.19(0.11 to 0.32)	0.49(-0.08 to 1.31)
San Marino	Liver cancer due to other causes	0(0 to 0)	0(0 to 0)	1.24(0.43 to 2.33)	0.13(0.09 to 0.17)	0.15(0.1 to 0.23)	0.19(-0.22 to 0.75)
Sao Tome and Principe	Liver cancer	2(1 to 2)	3(2 to 4)	0.81(0.21 to 1.54)	2.6(2.05 to 3.13)	2.8(1.89 to 3.69)	0.08(-0.28 to 0.49)

Sao Tome and Principe	Liver cancer due to alcohol use	0(0 to 0)	1(0 to 1)	1.17(0.37 to 2.14)	0.45(0.29 to 0.64)	0.61(0.35 to 0.92)	0.35(-0.13 to 0.91)
Sao Tome and Principe	Liver cancer due to hepatitis B	1(1 to 1)	1(1 to 2)	0.79(0.13 to 1.67)	1.26(0.9 to 1.64)	1.24(0.78 to 1.75)	-0.02(-0.36 to 0.42)
Sao Tome and Principe	Liver cancer due to hepatitis C	0(0 to 0)	0(0 to 1)	0.53(0.06 to 1.18)	0.53(0.36 to 0.73)	0.53(0.32 to 0.82)	0(-0.31 to 0.4)
Sao Tome and Principe	Liver cancer due to NASH	0(0 to 0)	0(0 to 0)	1.05(0.4 to 1.99)	0.22(0.15 to 0.33)	0.29(0.18 to 0.44)	0.28(-0.13 to 0.86)
Sao Tome and Principe	Liver cancer due to other causes	0(0 to 0)	0(0 to 0)	0.48(0.03 to 1.18)	0.14(0.1 to 0.18)	0.14(0.09 to 0.2)	0.03(-0.27 to 0.45)
Saudi Arabia	Liver cancer	330(235 to 434)	733(552 to 958)	1.22(0.44 to 2.49)	5.91(4.21 to 7.67)	4.9(3.86 to 6.25)	-0.17(-0.45 to 0.28)
Saudi Arabia	Liver cancer due to alcohol use	25(15 to 41)	59(34 to 95)	1.33(0.48 to 2.84)	0.47(0.27 to 0.76)	0.42(0.24 to 0.67)	-0.12(-0.44 to 0.44)
Saudi Arabia	Liver cancer due to hepatitis B	140(91 to 198)	287(192 to 413)	1.05(0.27 to 2.41)	2.22(1.44 to 3.17)	1.54(1.01 to 2.25)	-0.31(-0.55 to 0.13)
Saudi Arabia	Liver cancer due to hepatitis C	113(72 to 160)	246(163 to 345)	1.18(0.39 to 2.35)	2.31(1.48 to 3.26)	1.97(1.36 to 2.71)	-0.15(-0.44 to 0.29)
Saudi Arabia	Liver cancer due to NASH	32(19 to 48)	102(67 to 154)	2.19(1.07 to 4.1)	0.62(0.37 to 0.94)	0.76(0.49 to 1.15)	0.22(-0.2 to 0.93)
Saudi Arabia	Liver cancer due to other causes	20(14 to 28)	38(24 to 58)	0.87(0.25 to 1.84)	0.28(0.17 to 0.42)	0.22(0.13 to 0.34)	-0.22(-0.49 to 0.2)
Senegal	Liver cancer	80(63 to 95)	167(124 to 212)	1.09(0.51 to 1.84)	2.39(1.92 to 2.83)	2.21(1.65 to 2.78)	-0.08(-0.33 to 0.25)
Senegal	Liver cancer due to alcohol use	10(6 to 15)	21(13 to 32)	1.12(0.46 to 1.97)	0.33(0.21 to 0.47)	0.3(0.18 to 0.45)	-0.08(-0.36 to 0.28)
Senegal	Liver cancer due to hepatitis B	47(36 to 58)	96(67 to 128)	1.06(0.42 to 1.82)	1.4(1.08 to 1.74)	1.23(0.87 to 1.62)	-0.12(-0.38 to 0.2)

Senegal	Liver cancer due to hepatitis C	7(4 to 10)	15(9 to 23)	1.21(0.56 to 2.06)	0.24(0.15 to 0.35)	0.23(0.14 to 0.35)	-0.05(-0.32 to 0.3)
Senegal	Liver cancer due to NASH	8(5 to 11)	20(13 to 29)	1.57(0.78 to 2.63)	0.26(0.17 to 0.38)	0.29(0.19 to 0.43)	0.11(-0.22 to 0.55)
Senegal	Liver cancer due to other causes	9(6 to 12)	16(11 to 22)	0.75(0.11 to 1.55)	0.16(0.11 to 0.22)	0.16(0.1 to 0.23)	-0.03(-0.32 to 0.35)
Serbia	Liver cancer	700(582 to 808)	885(701 to 1107)	0.26(-0.04 to 0.64)	6.27(5.22 to 7.21)	5.49(4.36 to 6.87)	-0.13(-0.33 to 0.14)
Serbia	Liver cancer due to alcohol use	250(180 to 326)	334(225 to 462)	0.33(-0.02 to 0.79)	2.19(1.57 to 2.85)	2.03(1.37 to 2.79)	-0.08(-0.31 to 0.22)
Serbia	Liver cancer due to hepatitis B	163(111 to 230)	158(98 to 242)	-0.03(-0.29 to 0.31)	1.4(0.96 to 1.94)	1.04(0.67 to 1.56)	-0.26(-0.45 to -0.01)
Serbia	Liver cancer due to hepatitis C	224(155 to 309)	305(209 to 427)	0.36(0 to 0.81)	2.09(1.48 to 2.84)	1.87(1.3 to 2.56)	-0.11(-0.33 to 0.17)
Serbia	Liver cancer due to NASH	41(26 to 60)	65(41 to 97)	0.6(0.16 to 1.19)	0.39(0.25 to 0.56)	0.4(0.26 to 0.58)	0.03(-0.23 to 0.4)
Serbia	Liver cancer due to other causes	22(15 to 32)	23(14 to 36)	0.08(-0.21 to 0.47)	0.2(0.14 to 0.29)	0.16(0.1 to 0.23)	-0.23(-0.42 to 0.02)
Seychelles	Liver cancer	5(4 to 5)	6(5 to 7)	0.38(0.13 to 0.7)	8.01(6.98 to 9.25)	5.7(4.81 to 6.72)	-0.29(-0.42 to -0.14)
Seychelles	Liver cancer due to alcohol use	1(1 to 1)	2(1 to 2)	0.71(0.31 to 1.18)	1.65(1.1 to 2.38)	1.45(0.97 to 2.02)	-0.12(-0.32 to 0.11)
Seychelles	Liver cancer due to hepatitis B	2(1 to 2)	2(2 to 3)	0.29(0.01 to 0.66)	3.27(2.47 to 4.31)	2.03(1.48 to 2.71)	-0.38(-0.51 to -0.22)
Seychelles	Liver cancer due to hepatitis C	1(1 to 2)	1(1 to 2)	0.22(0 to 0.49)	2.1(1.48 to 2.83)	1.44(1 to 1.93)	-0.32(-0.44 to -0.17)
Seychelles	Liver cancer due to NASH	0(0 to 1)	1(0 to 1)	0.54(0.23 to 0.9)	0.75(0.52 to 1.04)	0.63(0.43 to 0.89)	-0.16(-0.34 to 0.03)

Seychelles	Liver cancer due to other causes	0(0 to 0)	0(0 to 0)	0.23(-0.01 to 0.52)	0.23(0.16 to 0.33)	0.16(0.11 to 0.22)	-0.33(-0.45 to -0.17)
Sierra Leone	Liver cancer	122(92 to 157)	181(135 to 239)	0.48(0.06 to 1.08)	6.24(4.76 to 8.03)	4.94(3.79 to 6.39)	-0.21(-0.43 to 0.1)
Sierra Leone	Liver cancer due to alcohol use	22(14 to 34)	32(19 to 48)	0.44(-0.01 to 1.08)	1.17(0.75 to 1.8)	0.93(0.58 to 1.4)	-0.2(-0.45 to 0.14)
Sierra Leone	Liver cancer due to hepatitis B	60(42 to 82)	86(59 to 123)	0.43(-0.02 to 1.07)	3.06(2.13 to 4.19)	2.21(1.52 to 3.14)	-0.28(-0.5 to 0.02)
Sierra Leone	Liver cancer due to hepatitis C	22(14 to 31)	32(20 to 47)	0.49(0.06 to 1.07)	1.23(0.79 to 1.8)	1.05(0.66 to 1.51)	-0.15(-0.39 to 0.19)
Sierra Leone	Liver cancer due to NASH	9(5 to 13)	16(10 to 23)	0.83(0.29 to 1.56)	0.47(0.3 to 0.69)	0.47(0.3 to 0.71)	0.01(-0.28 to 0.4)
Sierra Leone	Liver cancer due to other causes	10(6 to 14)	15(10 to 22)	0.57(0.03 to 1.36)	0.32(0.22 to 0.45)	0.28(0.18 to 0.42)	-0.11(-0.38 to 0.28)
Singapore	Liver cancer	180(169 to 191)	658(586 to 732)	2.65(2.31 to 3.04)	8.27(7.75 to 8.76)	8.68(7.67 to 9.66)	0.05(-0.05 to 0.16)
Singapore	Liver cancer due to alcohol use	13(9 to 19)	53(33 to 80)	3.01(2.35 to 3.78)	0.61(0.39 to 0.9)	0.69(0.43 to 1.04)	0.12(-0.06 to 0.33)
Singapore	Liver cancer due to hepatitis B	115(97 to 132)	362(283 to 443)	2.16(1.77 to 2.55)	5.01(4.17 to 5.88)	4.65(3.62 to 5.71)	-0.07(-0.18 to 0.04)
Singapore	Liver cancer due to hepatitis C	41(27 to 55)	193(129 to 260)	3.76(3.1 to 4.54)	2.08(1.43 to 2.8)	2.65(1.77 to 3.55)	0.27(0.1 to 0.47)
Singapore	Liver cancer due to NASH	7(4 to 10)	32(21 to 49)	3.96(3.12 to 4.98)	0.33(0.22 to 0.49)	0.44(0.28 to 0.67)	0.36(0.14 to 0.62)
Singapore	Liver cancer due to other causes	5(4 to 7)	18(11 to 26)	2.34(1.68 to 3.08)	0.24(0.17 to 0.33)	0.25(0.16 to 0.36)	0.02(-0.16 to 0.22)
Slovakia	Liver cancer	279(261 to 298)	312(243 to 395)	0.12(-0.13 to 0.45)	4.66(4.37 to 4.98)	3.39(2.64 to 4.28)	-0.27(-0.43 to -0.06)

Slovakia	Liver cancer due to alcohol use	123(96 to 149)	142(99 to 193)	0.15(-0.14 to 0.52)	2.04(1.61 to 2.46)	1.52(1.05 to 2.05)	-0.26(-0.44 to -0.02)
Slovakia	Liver cancer due to hepatitis B	64(46 to 86)	59(37 to 89)	-0.07(-0.31 to 0.23)	1.07(0.78 to 1.45)	0.66(0.42 to 0.99)	-0.38(-0.54 to -0.18)
Slovakia	Liver cancer due to hepatitis C	64(45 to 86)	77(50 to 111)	0.2(-0.08 to 0.53)	1.07(0.76 to 1.44)	0.82(0.54 to 1.18)	-0.23(-0.41 to -0.02)
Slovakia	Liver cancer due to NASH	18(12 to 25)	24(16 to 37)	0.36(0.03 to 0.8)	0.3(0.21 to 0.41)	0.26(0.17 to 0.4)	-0.12(-0.33 to 0.17)
Slovakia	Liver cancer due to other causes	9(7 to 13)	9(6 to 14)	0(-0.25 to 0.3)	0.16(0.12 to 0.22)	0.12(0.08 to 0.17)	-0.29(-0.45 to -0.08)
Slovenia	Liver cancer	74(57 to 93)	222(171 to 283)	2.01(1.07 to 3.26)	3(2.31 to 3.82)	5.14(3.93 to 6.61)	0.71(0.18 to 1.44)
Slovenia	Liver cancer due to alcohol use	29(20 to 40)	88(60 to 124)	2.07(1.11 to 3.44)	1.16(0.8 to 1.6)	2.06(1.39 to 2.91)	0.77(0.21 to 1.56)
Slovenia	Liver cancer due to hepatitis B	17(11 to 26)	45(29 to 68)	1.59(0.71 to 2.81)	0.71(0.47 to 1.05)	1.13(0.71 to 1.7)	0.58(0.05 to 1.32)
Slovenia	Liver cancer due to hepatitis C	19(13 to 29)	61(40 to 87)	2.16(1.18 to 3.36)	0.79(0.52 to 1.16)	1.33(0.86 to 1.9)	0.68(0.16 to 1.34)
Slovenia	Liver cancer due to NASH	5(3 to 8)	21(12 to 32)	2.79(1.56 to 4.4)	0.22(0.14 to 0.33)	0.45(0.28 to 0.7)	1.05(0.41 to 1.9)
Slovenia	Liver cancer due to other causes	3(2 to 4)	6(4 to 10)	1.5(0.71 to 2.57)	0.11(0.07 to 0.16)	0.16(0.1 to 0.24)	0.44(0.01 to 1.02)
Solomon Islands	Liver cancer	11(8 to 14)	19(15 to 23)	0.77(0.25 to 1.49)	6.81(5.07 to 8.82)	5.52(4.46 to 6.68)	-0.19(-0.41 to 0.14)
Solomon Islands	Liver cancer due to alcohol use	1(1 to 2)	2(1 to 3)	0.88(0.28 to 1.83)	0.76(0.45 to 1.22)	0.66(0.4 to 0.98)	-0.13(-0.4 to 0.29)
Solomon Islands	Liver cancer due to hepatitis B	6(4 to 8)	10(8 to 13)	0.67(0.16 to 1.36)	3.7(2.57 to 4.98)	2.73(2.05 to 3.57)	-0.26(-0.47 to 0.06)

Solomon Islands	Liver cancer due to hepatitis C	2(1 to 3)	3(2 to 5)	0.86(0.3 to 1.71)	1.44(0.87 to 2.08)	1.27(0.86 to 1.75)	-0.12(-0.35 to 0.25)
Solomon Islands	Liver cancer due to NASH	1(0 to 1)	2(1 to 2)	1.14(0.46 to 2.15)	0.51(0.33 to 0.81)	0.5(0.34 to 0.72)	-0.01(-0.29 to 0.41)
Solomon Islands	Liver cancer due to other causes	1(1 to 1)	2(1 to 3)	0.84(0.22 to 1.58)	0.41(0.28 to 0.58)	0.36(0.26 to 0.49)	-0.11(-0.35 to 0.23)
Somalia	Liver cancer	100(61 to 173)	248(160 to 434)	1.48(0.71 to 2.66)	3.76(2.35 to 6.53)	3.61(2.35 to 6.4)	-0.04(-0.31 to 0.38)
Somalia	Liver cancer due to alcohol use	19(10 to 42)	46(25 to 88)	1.39(0.62 to 2.54)	0.82(0.44 to 1.75)	0.74(0.4 to 1.43)	-0.1(-0.37 to 0.29)
Somalia	Liver cancer due to hepatitis B	37(21 to 72)	89(52 to 168)	1.4(0.63 to 2.65)	1.24(0.68 to 2.38)	1.13(0.67 to 2.12)	-0.08(-0.36 to 0.35)
Somalia	Liver cancer due to hepatitis C	19(10 to 34)	51(29 to 89)	1.61(0.81 to 2.8)	0.96(0.55 to 1.58)	0.99(0.59 to 1.76)	0.03(-0.26 to 0.45)
Somalia	Liver cancer due to NASH	10(5 to 17)	26(15 to 46)	1.65(0.83 to 2.98)	0.44(0.24 to 0.74)	0.45(0.26 to 0.78)	0.03(-0.27 to 0.48)
Somalia	Liver cancer due to other causes	14(8 to 23)	36(22 to 58)	1.53(0.56 to 3.15)	0.31(0.16 to 0.51)	0.3(0.18 to 0.5)	-0.03(-0.34 to 0.43)
South Africa	Liver cancer	1312(867 to 2258)	2638(2328 to 2995)	1.01(0.1 to 2.2)	6.04(3.93 to 10.36)	5.87(5.18 to 6.68)	-0.03(-0.46 to 0.56)
South Africa	Liver cancer due to alcohol use	261(153 to 501)	552(448 to 662)	1.12(0.04 to 2.65)	1.24(0.72 to 2.35)	1.23(1 to 1.47)	-0.01(-0.51 to 0.73)
South Africa	Liver cancer due to hepatitis B	494(318 to 877)	960(808 to 1132)	0.94(-0.02 to 2.22)	2.04(1.28 to 3.65)	1.94(1.63 to 2.29)	-0.05(-0.52 to 0.58)
South Africa	Liver cancer due to hepatitis C	343(222 to 533)	684(580 to 824)	0.99(0.23 to 2.01)	1.8(1.15 to 2.79)	1.7(1.45 to 2.04)	-0.06(-0.42 to 0.44)
South Africa	Liver cancer due to NASH	132(84 to 217)	311(262 to 373)	1.36(0.37 to 2.67)	0.63(0.4 to 1.06)	0.73(0.61 to 0.87)	0.15(-0.34 to 0.8)

South Africa	Liver cancer due to other causes	83(59 to 128)	130(109 to 155)	0.58(-0.02 to 1.26)	0.32(0.22 to 0.51)	0.27(0.23 to 0.32)	-0.15(-0.48 to 0.24)
South Sudan	Liver cancer	82(52 to 147)	115(67 to 198)	0.4(-0.01 to 0.99)	3.26(2.05 to 5.88)	2.97(1.79 to 5.14)	-0.09(-0.34 to 0.26)
South Sudan	Liver cancer due to alcohol use	18(9 to 40)	24(11 to 49)	0.35(-0.07 to 0.96)	0.76(0.37 to 1.65)	0.68(0.33 to 1.41)	-0.11(-0.37 to 0.28)
South Sudan	Liver cancer due to hepatitis B	26(14 to 53)	38(20 to 72)	0.46(-0.01 to 1.17)	1.01(0.55 to 2.02)	0.89(0.46 to 1.69)	-0.12(-0.38 to 0.28)
South Sudan	Liver cancer due to hepatitis C	17(10 to 29)	24(13 to 42)	0.41(-0.01 to 0.97)	0.83(0.49 to 1.41)	0.78(0.44 to 1.3)	-0.07(-0.33 to 0.27)
South Sudan	Liver cancer due to NASH	9(5 to 15)	14(7 to 24)	0.54(0.07 to 1.2)	0.39(0.22 to 0.69)	0.4(0.22 to 0.69)	0.01(-0.27 to 0.41)
South Sudan	Liver cancer due to other causes	12(7 to 18)	15(9 to 22)	0.23(-0.23 to 0.86)	0.27(0.17 to 0.44)	0.23(0.14 to 0.36)	-0.15(-0.41 to 0.21)
Spain	Liver cancer	2189(2069 to 2288)	4973(4472 to 5437)	1.27(1.07 to 1.49)	3.98(3.76 to 4.16)	5.18(4.67 to 5.67)	0.3(0.18 to 0.43)
Spain	Liver cancer due to alcohol use	673(475 to 874)	1324(918 to 1791)	0.97(0.68 to 1.26)	1.21(0.86 to 1.56)	1.45(1 to 1.93)	0.2(0.04 to 0.38)
Spain	Liver cancer due to hepatitis B	230(158 to 330)	467(310 to 685)	1.03(0.74 to 1.36)	0.44(0.31 to 0.62)	0.56(0.37 to 0.81)	0.26(0.09 to 0.46)
Spain	Liver cancer due to hepatitis C	1102(880 to 1341)	2735(2182 to 3275)	1.48(1.21 to 1.79)	1.99(1.6 to 2.42)	2.7(2.1 to 3.3)	0.36(0.22 to 0.51)
Spain	Liver cancer due to NASH	107(71 to 156)	290(191 to 422)	1.71(1.25 to 2.19)	0.19(0.13 to 0.28)	0.29(0.19 to 0.42)	0.49(0.28 to 0.72)
Spain	Liver cancer due to other causes	76(52 to 107)	156(102 to 225)	1.07(0.72 to 1.46)	0.15(0.11 to 0.21)	0.18(0.13 to 0.26)	0.2(0.03 to 0.38)
Sri Lanka	Liver cancer	229(200 to 261)	706(512 to 941)	2.09(1.15 to 3.22)	2.22(1.95 to 2.52)	2.84(2.11 to 3.77)	0.28(-0.11 to 0.74)

Sri Lanka	Liver cancer due to alcohol use	46(30 to 65)	181(110 to 276)	2.97(1.68 to 4.74)	0.45(0.3 to 0.64)	0.7(0.43 to 1.05)	0.55(0.06 to 1.22)
Sri Lanka	Liver cancer due to hepatitis B	98(75 to 126)	252(161 to 372)	1.58(0.75 to 2.61)	0.87(0.65 to 1.14)	0.98(0.64 to 1.44)	0.12(-0.23 to 0.57)
Sri Lanka	Liver cancer due to hepatitis C	54(37 to 73)	181(116 to 266)	2.33(1.35 to 3.7)	0.61(0.43 to 0.82)	0.77(0.5 to 1.12)	0.27(-0.09 to 0.76)
Sri Lanka	Liver cancer due to NASH	19(13 to 27)	69(43 to 109)	2.58(1.47 to 4.07)	0.2(0.14 to 0.29)	0.29(0.18 to 0.45)	0.43(0 to 0.98)
Sri Lanka	Liver cancer due to other causes	12(8 to 17)	23(14 to 34)	0.98(0.32 to 1.9)	0.09(0.06 to 0.12)	0.1(0.06 to 0.14)	0.12(-0.2 to 0.55)
Sudan	Liver cancer	344(202 to 521)	684(414 to 1074)	0.99(0.39 to 1.89)	3.8(2.21 to 5.72)	3.92(2.41 to 6.04)	0.03(-0.28 to 0.53)
Sudan	Liver cancer due to alcohol use	45(22 to 85)	95(49 to 179)	1.13(0.38 to 2.46)	0.5(0.25 to 0.95)	0.57(0.3 to 1.07)	0.15(-0.25 to 0.85)
Sudan	Liver cancer due to hepatitis B	129(75 to 200)	241(133 to 396)	0.88(0.25 to 1.94)	1.35(0.77 to 2.13)	1.27(0.71 to 2.07)	-0.06(-0.36 to 0.48)
Sudan	Liver cancer due to hepatitis C	102(56 to 159)	194(111 to 313)	0.89(0.32 to 1.75)	1.26(0.69 to 1.94)	1.24(0.72 to 1.96)	-0.02(-0.3 to 0.43)
Sudan	Liver cancer due to NASH	34(17 to 56)	89(52 to 144)	1.65(0.78 to 2.85)	0.39(0.2 to 0.64)	0.54(0.31 to 0.86)	0.38(-0.06 to 0.99)
Sudan	Liver cancer due to other causes	35(20 to 53)	65(41 to 103)	0.84(0.3 to 1.57)	0.3(0.17 to 0.45)	0.3(0.18 to 0.49)	0.01(-0.29 to 0.4)
Suriname	Liver cancer	18(16 to 20)	16(13 to 20)	-0.08(-0.27 to 0.15)	6.83(6.1 to 7.57)	2.74(2.21 to 3.38)	-0.6(-0.68 to -0.5)
Suriname	Liver cancer due to alcohol use	6(4 to 7)	6(4 to 8)	-0.01(-0.23 to 0.26)	2.27(1.64 to 2.96)	0.96(0.68 to 1.34)	-0.58(-0.67 to -0.47)
Suriname	Liver cancer due to hepatitis B	5(4 to 7)	4(3 to 6)	-0.18(-0.36 to 0.06)	1.94(1.42 to 2.59)	0.71(0.49 to 1)	-0.63(-0.71 to -0.53)

Suriname	Liver cancer due to hepatitis C	4(3 to 5)	4(2 to 5)	-0.08(-0.28 to 0.15)	1.61(1.14 to 2.17)	0.63(0.43 to 0.9)	-0.61(-0.69 to -0.51)
Suriname	Liver cancer due to NASH	1(1 to 2)	2(1 to 2)	0.14(-0.12 to 0.48)	0.54(0.38 to 0.79)	0.27(0.18 to 0.39)	-0.51(-0.62 to -0.36)
Suriname	Liver cancer due to other causes	1(1 to 2)	1(1 to 1)	-0.23(-0.41 to 0.01)	0.46(0.33 to 0.62)	0.17(0.12 to 0.24)	-0.62(-0.71 to -0.52)
Sweden	Liver cancer	380(358 to 400)	624(573 to 670)	0.64(0.51 to 0.76)	2.52(2.39 to 2.65)	3.02(2.8 to 3.24)	0.2(0.1 to 0.29)
Sweden	Liver cancer due to alcohol use	150(130 to 170)	272(235 to 309)	0.81(0.63 to 1)	1(0.87 to 1.13)	1.35(1.16 to 1.53)	0.35(0.22 to 0.49)
Sweden	Liver cancer due to hepatitis B	21(16 to 27)	33(25 to 42)	0.56(0.41 to 0.7)	0.16(0.12 to 0.2)	0.18(0.14 to 0.24)	0.16(0.04 to 0.27)
Sweden	Liver cancer due to hepatitis C	157(134 to 182)	237(198 to 277)	0.5(0.38 to 0.64)	1(0.86 to 1.16)	1.08(0.91 to 1.27)	0.08(-0.01 to 0.17)
Sweden	Liver cancer due to NASH	29(24 to 36)	49(40 to 60)	0.71(0.55 to 0.87)	0.19(0.15 to 0.22)	0.23(0.19 to 0.28)	0.23(0.12 to 0.35)
Sweden	Liver cancer due to other causes	23(19 to 27)	33(28 to 40)	0.44(0.31 to 0.59)	0.18(0.15 to 0.2)	0.18(0.16 to 0.22)	0.04(-0.05 to 0.13)
Switzerland	Liver cancer	244(231 to 257)	766(685 to 850)	2.13(1.79 to 2.52)	2.36(2.23 to 2.47)	4.43(3.97 to 4.91)	0.88(0.67 to 1.11)
Switzerland	Liver cancer due to alcohol use	107(82 to 129)	306(224 to 389)	1.87(1.45 to 2.32)	1.03(0.78 to 1.25)	1.8(1.32 to 2.29)	0.74(0.5 to 1.02)
Switzerland	Liver cancer due to hepatitis B	30(20 to 44)	93(61 to 136)	2.08(1.68 to 2.57)	0.31(0.21 to 0.45)	0.59(0.39 to 0.85)	0.89(0.64 to 1.2)
Switzerland	Liver cancer due to hepatitis C	84(62 to 109)	289(211 to 377)	2.42(2.04 to 2.89)	0.78(0.57 to 1.02)	1.58(1.15 to 2.08)	1.02(0.79 to 1.28)
Switzerland	Liver cancer due to NASH	13(9 to 19)	47(30 to 69)	2.62(2.07 to 3.2)	0.12(0.08 to 0.17)	0.26(0.17 to 0.38)	1.17(0.85 to 1.51)

Switzerland	Liver cancer due to other causes	10(7 to 15)	31(20 to 45)	2.04(1.53 to 2.56)	0.11(0.08 to 0.15)	0.2(0.14 to 0.28)	0.79(0.51 to 1.08)
Syrian Arab Republic	Liver cancer	264(199 to 333)	502(374 to 667)	0.9(0.29 to 1.91)	5.21(3.84 to 6.69)	4.41(3.35 to 5.77)	-0.15(-0.43 to 0.31)
Syrian Arab Republic	Liver cancer due to alcohol use	23(14 to 36)	48(27 to 77)	1.04(0.3 to 2.28)	0.48(0.29 to 0.75)	0.41(0.24 to 0.65)	-0.15(-0.46 to 0.35)
Syrian Arab Republic	Liver cancer due to hepatitis B	103(73 to 142)	179(118 to 260)	0.74(0.15 to 1.7)	1.83(1.25 to 2.58)	1.4(0.92 to 2.04)	-0.24(-0.49 to 0.18)
Syrian Arab Republic	Liver cancer due to hepatitis C	89(59 to 126)	179(121 to 252)	1(0.34 to 2.08)	2.02(1.31 to 2.82)	1.75(1.22 to 2.4)	-0.13(-0.41 to 0.34)
Syrian Arab Republic	Liver cancer due to NASH	25(16 to 38)	60(38 to 91)	1.38(0.56 to 2.78)	0.53(0.33 to 0.83)	0.55(0.35 to 0.83)	0.03(-0.32 to 0.64)
Syrian Arab Republic	Liver cancer due to other causes	23(17 to 32)	36(23 to 53)	0.55(0.05 to 1.29)	0.35(0.23 to 0.51)	0.29(0.19 to 0.43)	-0.16(-0.44 to 0.25)
Taiwan (Province of China)	Liver cancer	2025(1941 to 2109)	2852(2228 to 3660)	0.41(0.09 to 0.81)	11.98(11.46 to 12.47)	7.27(5.69 to 9.32)	-0.39(-0.53 to -0.22)
Taiwan (Province of China)	Liver cancer due to alcohol use	202(133 to 293)	263(157 to 409)	0.3(-0.04 to 0.78)	1.2(0.8 to 1.74)	0.66(0.4 to 1.02)	-0.45(-0.59 to -0.26)
Taiwan (Province of China)	Liver cancer due to hepatitis B	1122(948 to 1290)	1190(847 to 1621)	0.06(-0.18 to 0.37)	6.34(5.34 to 7.33)	3.08(2.22 to 4.17)	-0.51(-0.62 to -0.37)
Taiwan (Province of China)	Liver cancer due to hepatitis C	502(363 to 642)	1043(721 to 1425)	1.08(0.6 to 1.69)	3.26(2.4 to 4.08)	2.6(1.81 to 3.56)	-0.2(-0.38 to 0.02)
Taiwan (Province of China)	Liver cancer due to NASH	100(69 to 145)	230(148 to 348)	1.3(0.68 to 2.1)	0.62(0.43 to 0.89)	0.58(0.38 to 0.87)	-0.06(-0.29 to 0.26)

Taiwan (Province of China)	Liver cancer due to other causes	99(71 to 136)	127(80 to 192)	0.28(-0.09 to 0.7)	0.57(0.41 to 0.78)	0.35(0.23 to 0.52)	-0.39(-0.56 to -0.19)
Tajikistan	Liver cancer	33(30 to 36)	174(140 to 218)	4.32(3.19 to 5.67)	1.07(0.96 to 1.18)	4.02(3.23 to 5.06)	2.77(1.97 to 3.73)
Tajikistan	Liver cancer due to alcohol use	6(4 to 8)	39(25 to 56)	5.5(3.94 to 7.67)	0.22(0.15 to 0.31)	0.89(0.57 to 1.3)	3.02(2.07 to 4.24)
Tajikistan	Liver cancer due to hepatitis B	5(4 to 7)	36(25 to 51)	5.93(4.29 to 8.13)	0.18(0.12 to 0.24)	0.63(0.42 to 0.89)	2.52(1.67 to 3.51)
Tajikistan	Liver cancer due to hepatitis C	14(11 to 17)	79(58 to 105)	4.77(3.44 to 6.44)	0.52(0.41 to 0.63)	2.1(1.57 to 2.75)	3.05(2.15 to 4.17)
Tajikistan	Liver cancer due to NASH	1(1 to 2)	10(6 to 14)	6.12(4.3 to 8.38)	0.05(0.03 to 0.07)	0.24(0.15 to 0.36)	3.82(2.63 to 5.3)
Tajikistan	Liver cancer due to other causes	7(5 to 8)	11(8 to 16)	0.66(0.2 to 1.62)	0.1(0.08 to 0.12)	0.16(0.12 to 0.24)	0.66(0.2 to 1.34)
Thailand	Liver cancer	7883(6844 to 9011)	24527(18171 to 32511)	2.11(1.25 to 3.26)	21.6(18.83 to 24.52)	24.01(17.88 to 31.65)	0.11(-0.19 to 0.52)
Thailand	Liver cancer due to alcohol use	1918(1305 to 2631)	7386(4775 to 10828)	2.85(1.71 to 4.42)	5.41(3.73 to 7.43)	7.17(4.61 to 10.45)	0.32(-0.06 to 0.86)
Thailand	Liver cancer due to hepatitis B	3882(3073 to 4784)	9765(6627 to 13874)	1.52(0.76 to 2.52)	9.74(7.65 to 12.15)	9.5(6.5 to 13.47)	-0.02(-0.31 to 0.35)
Thailand	Liver cancer due to hepatitis C	1296(874 to 1802)	4319(2729 to 6416)	2.33(1.41 to 3.62)	4.2(2.91 to 5.7)	4.3(2.73 to 6.36)	0.03(-0.25 to 0.39)
Thailand	Liver cancer due to NASH	536(368 to 758)	2427(1528 to 3631)	3.53(2.24 to 5.43)	1.61(1.12 to 2.29)	2.41(1.52 to 3.6)	0.5(0.08 to 1.09)
Thailand	Liver cancer due to other causes	251(175 to 342)	630(379 to 962)	1.51(0.74 to 2.46)	0.64(0.44 to 0.89)	0.63(0.39 to 0.95)	-0.02(-0.3 to 0.34)

Timor-Leste	Liver cancer	23(15 to 33)	53(35 to 74)	1.28(0.52 to 2.37)	7.84(5.35 to 10.86)	6.6(4.53 to 9.09)	-0.16(-0.41 to 0.18)
Timor-Leste	Liver cancer due to alcohol use	4(2 to 6)	11(6 to 18)	1.83(0.86 to 3.18)	1.4(0.8 to 2.26)	1.33(0.72 to 2.16)	-0.05(-0.36 to 0.36)
Timor-Leste	Liver cancer due to hepatitis B	11(7 to 18)	21(12 to 33)	0.91(0.18 to 1.89)	3.28(2.02 to 5.14)	2.54(1.42 to 3.92)	-0.22(-0.49 to 0.13)
Timor-Leste	Liver cancer due to hepatitis C	5(3 to 8)	14(9 to 21)	1.67(0.83 to 2.88)	2.3(1.44 to 3.46)	1.94(1.27 to 2.81)	-0.15(-0.39 to 0.19)
Timor-Leste	Liver cancer due to NASH	2(1 to 2)	4(3 to 7)	1.75(0.82 to 3.06)	0.61(0.36 to 0.95)	0.57(0.34 to 0.85)	-0.06(-0.35 to 0.31)
Timor-Leste	Liver cancer due to other causes	1(1 to 2)	2(1 to 3)	0.73(-0.17 to 1.93)	0.26(0.15 to 0.4)	0.22(0.13 to 0.33)	-0.16(-0.48 to 0.27)
Togo	Liver cancer	88(69 to 111)	192(146 to 251)	1.2(0.56 to 2.05)	6.76(5.41 to 8.54)	5.24(4.09 to 6.74)	-0.22(-0.44 to 0.06)
Togo	Liver cancer due to alcohol use	14(9 to 21)	33(20 to 49)	1.36(0.61 to 2.41)	1.17(0.71 to 1.79)	0.94(0.6 to 1.39)	-0.19(-0.44 to 0.14)
Togo	Liver cancer due to hepatitis B	46(34 to 61)	97(68 to 134)	1.12(0.44 to 2.05)	3.32(2.42 to 4.43)	2.36(1.68 to 3.25)	-0.29(-0.51 to 0)
Togo	Liver cancer due to hepatitis C	15(9 to 21)	33(21 to 47)	1.26(0.63 to 2.13)	1.4(0.94 to 1.95)	1.13(0.74 to 1.6)	-0.19(-0.4 to 0.11)
Togo	Liver cancer due to NASH	6(4 to 9)	17(11 to 26)	1.8(1.01 to 2.91)	0.53(0.35 to 0.78)	0.53(0.35 to 0.81)	0(-0.27 to 0.37)
Togo	Liver cancer due to other causes	7(5 to 10)	13(9 to 18)	0.76(0.23 to 1.47)	0.34(0.24 to 0.47)	0.28(0.19 to 0.4)	-0.17(-0.41 to 0.13)
Tokelau	Liver cancer	0(0 to 0)	0(0 to 0)	-0.15(-0.39 to 0.17)	8.64(6.43 to 11.29)	7.48(5.46 to 10.06)	-0.13(-0.38 to 0.18)
Tokelau	Liver cancer due to alcohol use	0(0 to 0)	0(0 to 0)	-0.09(-0.35 to 0.28)	0.95(0.55 to 1.48)	0.88(0.51 to 1.35)	-0.07(-0.33 to 0.27)

Tokelau	Liver cancer due to hepatitis B	0(0 to 0)	0(0 to 0)	-0.12(-0.38 to 0.23)	4.3(2.87 to 6.05)	3.74(2.43 to 5.33)	-0.13(-0.39 to 0.2)
Tokelau	Liver cancer due to hepatitis C	0(0 to 0)	0(0 to 0)	-0.26(-0.48 to 0.04)	2.24(1.44 to 3.15)	1.78(1.18 to 2.53)	-0.21(-0.43 to 0.11)
Tokelau	Liver cancer due to NASH	0(0 to 0)	0(0 to 0)	0(-0.3 to 0.44)	0.72(0.47 to 1.1)	0.76(0.49 to 1.12)	0.05(-0.26 to 0.51)
Tokelau	Liver cancer due to other causes	0(0 to 0)	0(0 to 0)	-0.26(-0.49 to 0.04)	0.41(0.26 to 0.64)	0.32(0.2 to 0.48)	-0.23(-0.48 to 0.09)
Tonga	Liver cancer	14(9 to 18)	20(14 to 26)	0.45(0.04 to 1.03)	23.93(16.74 to 30.82)	24.74(18.09 to 32.04)	0.03(-0.25 to 0.42)
Tonga	Liver cancer due to alcohol use	2(1 to 3)	3(2 to 4)	0.59(0.11 to 1.24)	2.92(1.66 to 4.54)	3.31(1.96 to 5.14)	0.13(-0.2 to 0.58)
Tonga	Liver cancer due to hepatitis B	8(5 to 11)	11(7 to 15)	0.35(-0.06 to 0.9)	13.1(8.57 to 18.15)	12.87(8.72 to 18.05)	-0.02(-0.31 to 0.38)
Tonga	Liver cancer due to hepatitis C	3(2 to 4)	4(3 to 6)	0.54(0.13 to 1.03)	5.07(3.22 to 7.5)	5.15(3.28 to 7.5)	0.02(-0.24 to 0.34)
Tonga	Liver cancer due to NASH	1(1 to 2)	2(1 to 3)	0.87(0.38 to 1.58)	1.93(1.17 to 2.93)	2.46(1.56 to 3.62)	0.28(-0.06 to 0.73)
Tonga	Liver cancer due to other causes	1(0 to 1)	1(0 to 1)	0.38(0 to 0.94)	0.92(0.57 to 1.38)	0.94(0.57 to 1.44)	0.02(-0.26 to 0.43)
Trinidad and Tobago	Liver cancer	59(55 to 63)	49(37 to 63)	-0.18(-0.39 to 0.08)	7.1(6.63 to 7.57)	2.66(2.03 to 3.46)	-0.63(-0.72 to -0.51)
Trinidad and Tobago	Liver cancer due to alcohol use	19(14 to 24)	17(11 to 24)	-0.11(-0.35 to 0.2)	2.28(1.67 to 2.9)	0.92(0.6 to 1.32)	-0.6(-0.71 to -0.47)
Trinidad and Tobago	Liver cancer due to hepatitis B	17(12 to 22)	12(8 to 18)	-0.28(-0.48 to -0.04)	1.91(1.41 to 2.55)	0.65(0.42 to 0.96)	-0.66(-0.75 to -0.55)
Trinidad and Tobago	Liver cancer due to hepatitis C	14(10 to 18)	11(7 to 17)	-0.18(-0.38 to 0.07)	1.79(1.27 to 2.34)	0.63(0.4 to 0.92)	-0.65(-0.73 to -0.54)

Trinidad and Tobago	Liver cancer due to NASH	5(4 to 7)	5(3 to 8)	0.01(-0.25 to 0.34)	0.65(0.46 to 0.92)	0.3(0.19 to 0.45)	-0.54(-0.67 to -0.4)
Trinidad and Tobago	Liver cancer due to other causes	4(3 to 6)	3(2 to 4)	-0.33(-0.51 to -0.09)	0.48(0.34 to 0.64)	0.17(0.11 to 0.24)	-0.65(-0.74 to -0.53)
Tunisia	Liver cancer	97(75 to 123)	220(155 to 311)	1.28(0.5 to 2.48)	1.98(1.54 to 2.51)	1.8(1.27 to 2.53)	-0.09(-0.4 to 0.38)
Tunisia	Liver cancer due to alcohol use	10(6 to 17)	26(13 to 48)	1.53(0.55 to 3.13)	0.21(0.12 to 0.35)	0.21(0.11 to 0.38)	0(-0.38 to 0.62)
Tunisia	Liver cancer due to hepatitis B	31(21 to 44)	61(36 to 95)	0.98(0.24 to 2.14)	0.58(0.39 to 0.84)	0.48(0.29 to 0.74)	-0.19(-0.49 to 0.3)
Tunisia	Liver cancer due to hepatitis C	43(30 to 59)	106(71 to 155)	1.45(0.61 to 2.84)	0.97(0.67 to 1.28)	0.89(0.6 to 1.3)	-0.07(-0.39 to 0.44)
Tunisia	Liver cancer due to NASH	6(4 to 9)	17(10 to 27)	1.97(0.92 to 3.69)	0.12(0.08 to 0.19)	0.14(0.08 to 0.22)	0.15(-0.24 to 0.82)
Tunisia	Liver cancer due to other causes	6(5 to 9)	10(6 to 15)	0.52(-0.02 to 1.28)	0.1(0.07 to 0.14)	0.08(0.05 to 0.13)	-0.2(-0.46 to 0.19)
Turkey	Liver cancer	1377(1104 to 1666)	2537(2009 to 3121)	0.84(0.37 to 1.55)	3.92(3.13 to 4.69)	2.96(2.36 to 3.63)	-0.24(-0.44 to 0.05)
Turkey	Liver cancer due to alcohol use	163(105 to 241)	337(210 to 515)	1.07(0.47 to 1.96)	0.49(0.31 to 0.73)	0.39(0.24 to 0.6)	-0.19(-0.43 to 0.13)
Turkey	Liver cancer due to hepatitis B	672(506 to 853)	1063(771 to 1448)	0.58(0.11 to 1.24)	1.8(1.34 to 2.3)	1.2(0.86 to 1.63)	-0.34(-0.52 to -0.07)
Turkey	Liver cancer due to hepatitis C	332(216 to 466)	707(477 to 947)	1.13(0.56 to 2.09)	1.05(0.69 to 1.44)	0.86(0.58 to 1.14)	-0.18(-0.4 to 0.19)
Turkey	Liver cancer due to NASH	110(71 to 164)	286(190 to 414)	1.6(0.82 to 2.71)	0.33(0.21 to 0.5)	0.34(0.23 to 0.49)	0.03(-0.27 to 0.46)
Turkey	Liver cancer due to other causes	100(71 to 139)	143(94 to 204)	0.43(0 to 0.95)	0.25(0.16 to 0.35)	0.17(0.12 to 0.24)	-0.29(-0.48 to -0.03)

Turkmenistan	Liver cancer	24(23 to 26)	229(181 to 291)	8.37(6.29 to 11.03)	1.21(1.13 to 1.3)	5.59(4.41 to 7.07)	3.62(2.59 to 4.86)
Turkmenistan	Liver cancer due to alcohol use	5(3 to 6)	69(45 to 98)	13.39(9.69 to 18.08)	0.27(0.18 to 0.36)	1.71(1.14 to 2.43)	5.44(3.84 to 7.41)
Turkmenistan	Liver cancer due to hepatitis B	5(4 to 7)	63(43 to 90)	10.98(8.03 to 15.12)	0.26(0.19 to 0.36)	1.4(0.94 to 1.99)	4.29(2.99 to 6.01)
Turkmenistan	Liver cancer due to hepatitis C	8(6 to 10)	68(45 to 95)	7.22(5.24 to 9.66)	0.49(0.38 to 0.6)	1.79(1.19 to 2.44)	2.63(1.79 to 3.69)
Turkmenistan	Liver cancer due to NASH	1(1 to 2)	16(10 to 24)	11.97(8.39 to 16.53)	0.07(0.05 to 0.1)	0.41(0.27 to 0.62)	4.72(3.19 to 6.62)
Turkmenistan	Liver cancer due to other causes	5(4 to 6)	13(10 to 17)	1.68(1 to 2.7)	0.12(0.1 to 0.14)	0.28(0.21 to 0.38)	1.43(0.87 to 2.2)
Tuvalu	Liver cancer	1(1 to 1)	1(1 to 1)	0.19(-0.13 to 0.68)	10.76(8.16 to 13.96)	8.72(6.51 to 11.52)	-0.19(-0.41 to 0.13)
Tuvalu	Liver cancer due to alcohol use	0(0 to 0)	0(0 to 0)	0.29(-0.09 to 0.88)	1.19(0.7 to 1.89)	1.06(0.63 to 1.66)	-0.12(-0.37 to 0.28)
Tuvalu	Liver cancer due to hepatitis B	0(0 to 1)	0(0 to 1)	0.15(-0.18 to 0.67)	5.58(3.91 to 7.73)	4.43(3.08 to 6.32)	-0.21(-0.43 to 0.13)
Tuvalu	Liver cancer due to hepatitis C	0(0 to 0)	0(0 to 0)	0.18(-0.14 to 0.65)	2.68(1.72 to 3.98)	2.07(1.3 to 3)	-0.23(-0.44 to 0.07)
Tuvalu	Liver cancer due to NASH	0(0 to 0)	0(0 to 0)	0.47(0.06 to 1.08)	0.82(0.51 to 1.25)	0.8(0.51 to 1.19)	-0.03(-0.31 to 0.39)
Tuvalu	Liver cancer due to other causes	0(0 to 0)	0(0 to 0)	0.06(-0.28 to 0.53)	0.49(0.31 to 0.73)	0.37(0.23 to 0.55)	-0.25(-0.48 to 0.08)
Uganda	Liver cancer	329(265 to 399)	974(763 to 1224)	1.96(1.2 to 3.05)	4.75(3.85 to 5.74)	6.39(5.12 to 7.88)	0.34(0.02 to 0.8)
Uganda	Liver cancer due to alcohol use	103(68 to 141)	292(198 to 413)	1.83(1.09 to 2.99)	1.59(1.06 to 2.15)	2.07(1.42 to 2.85)	0.3(-0.02 to 0.79)

Uganda	Liver cancer due to hepatitis B	100(69 to 142)	284(191 to 409)	1.83(1.02 to 3.15)	1.38(0.93 to 1.98)	1.63(1.1 to 2.35)	0.18(-0.14 to 0.69)
Uganda	Liver cancer due to hepatitis C	57(37 to 81)	176(116 to 250)	2.09(1.28 to 3.14)	0.99(0.66 to 1.38)	1.46(0.98 to 2.04)	0.47(0.12 to 0.92)
Uganda	Liver cancer due to NASH	29(19 to 41)	101(69 to 149)	2.55(1.58 to 3.92)	0.46(0.31 to 0.67)	0.76(0.5 to 1.12)	0.63(0.22 to 1.16)
Uganda	Liver cancer due to other causes	41(28 to 56)	121(83 to 175)	1.99(1 to 3.43)	0.33(0.23 to 0.46)	0.48(0.33 to 0.68)	0.47(0.08 to 1.01)
Ukraine	Liver cancer	704(672 to 737)	1965(1665 to 2295)	1.79(1.37 to 2.26)	1.02(0.97 to 1.07)	2.66(2.24 to 3.11)	1.61(1.19 to 2.06)
Ukraine	Liver cancer due to alcohol use	213(183 to 241)	699(561 to 856)	2.29(1.74 to 2.94)	0.29(0.25 to 0.33)	0.92(0.74 to 1.13)	2.13(1.61 to 2.77)
Ukraine	Liver cancer due to hepatitis B	161(136 to 191)	451(355 to 566)	1.8(1.32 to 2.37)	0.23(0.19 to 0.27)	0.64(0.51 to 0.81)	1.83(1.33 to 2.42)
Ukraine	Liver cancer due to hepatitis C	225(196 to 256)	573(467 to 690)	1.55(1.16 to 1.98)	0.32(0.28 to 0.36)	0.73(0.6 to 0.89)	1.27(0.93 to 1.67)
Ukraine	Liver cancer due to NASH	56(47 to 67)	163(129 to 203)	1.9(1.44 to 2.42)	0.08(0.07 to 0.1)	0.21(0.17 to 0.26)	1.68(1.24 to 2.14)
Ukraine	Liver cancer due to other causes	49(40 to 58)	78(62 to 98)	0.59(0.21 to 1.16)	0.1(0.08 to 0.12)	0.15(0.11 to 0.2)	0.54(0.07 to 1.28)
United Arab Emirates	Liver cancer	20(11 to 35)	203(84 to 464)	9.31(4.57 to 16.23)	4.57(2.27 to 8.67)	4.9(2.06 to 11.3)	0.07(-0.38 to 0.65)
United Arab Emirates	Liver cancer due to alcohol use	2(1 to 5)	22(7 to 59)	9.11(4.25 to 15.73)	0.58(0.23 to 1.3)	0.61(0.2 to 1.63)	0.05(-0.43 to 0.68)
United Arab Emirates	Liver cancer due to hepatitis B	10(5 to 18)	106(43 to 245)	9.99(4.68 to 17.96)	1.57(0.71 to 3.26)	1.72(0.68 to 4.2)	0.09(-0.39 to 0.72)
United Arab Emirates	Liver cancer due to hepatitis C	5(2 to 9)	43(16 to 107)	8.23(4.14 to 13.82)	1.7(0.79 to 3.45)	1.7(0.67 to 4.26)	0(-0.41 to 0.52)

United Arab Emirates	Liver cancer due to NASH	2(1 to 3)	20(8 to 51)	11.78(5.91 to 19.89)	0.47(0.22 to 0.95)	0.64(0.25 to 1.62)	0.37(-0.21 to 1.13)
United Arab Emirates	Liver cancer due to other causes	2(1 to 3)	12(5 to 28)	6.23(2.78 to 11.78)	0.25(0.13 to 0.5)	0.23(0.1 to 0.55)	-0.07(-0.45 to 0.43)
United Kingdom	Liver cancer	1681(1609 to 1725)	5155(4749 to 5460)	2.07(1.89 to 2.22)	1.87(1.8 to 1.92)	4.03(3.75 to 4.26)	1.16(1.04 to 1.26)
United Kingdom	Liver cancer due to alcohol use	602(535 to 670)	1822(1598 to 2053)	2.03(1.82 to 2.23)	0.66(0.59 to 0.74)	1.45(1.28 to 1.63)	1.19(1.03 to 1.33)
United Kingdom	Liver cancer due to hepatitis B	215(178 to 257)	608(499 to 730)	1.82(1.63 to 2.01)	0.26(0.22 to 0.31)	0.53(0.44 to 0.63)	1.05(0.92 to 1.17)
United Kingdom	Liver cancer due to hepatitis C	664(590 to 739)	2087(1831 to 2335)	2.14(1.95 to 2.32)	0.72(0.64 to 0.79)	1.55(1.36 to 1.73)	1.16(1.05 to 1.27)
United Kingdom	Liver cancer due to NASH	111(93 to 131)	398(332 to 471)	2.6(2.37 to 2.82)	0.12(0.1 to 0.14)	0.3(0.25 to 0.35)	1.52(1.39 to 1.66)
United Kingdom	Liver cancer due to other causes	89(76 to 102)	241(205 to 283)	1.72(1.56 to 1.88)	0.11(0.1 to 0.13)	0.21(0.18 to 0.24)	0.86(0.76 to 0.96)
United Republic of Tanzania	Liver cancer	271(217 to 332)	639(500 to 813)	1.36(0.78 to 2.08)	2.21(1.83 to 2.69)	2.46(1.98 to 3.02)	0.11(-0.12 to 0.41)
United Republic of Tanzania	Liver cancer due to alcohol use	60(40 to 88)	155(101 to 223)	1.58(0.92 to 2.4)	0.58(0.39 to 0.83)	0.68(0.44 to 0.98)	0.17(-0.11 to 0.53)
United Republic of Tanzania	Liver cancer due to hepatitis B	70(49 to 99)	161(109 to 230)	1.29(0.69 to 2.08)	0.58(0.4 to 0.82)	0.58(0.39 to 0.84)	0.01(-0.24 to 0.33)
United Republic of Tanzania	Liver cancer due to hepatitis C	49(33 to 68)	126(84 to 172)	1.56(1 to 2.31)	0.55(0.38 to 0.74)	0.62(0.42 to 0.84)	0.11(-0.12 to 0.4)
United Republic of Tanzania	Liver cancer due to NASH	26(18 to 37)	75(52 to 108)	1.9(1.22 to 2.81)	0.26(0.18 to 0.38)	0.34(0.23 to 0.48)	0.28(0 to 0.64)

United Republic of Tanzania	Liver cancer due to other causes	65(43 to 98)	123(75 to 190)	0.87(0.08 to 2.2)	0.24(0.17 to 0.32)	0.24(0.17 to 0.34)	0.01(-0.3 to 0.44)
United States of America	Liver cancer	6454(6183 to 6616)	23807(21185 to 26096)	2.69(2.3 to 3.03)	2.04(1.96 to 2.09)	4.33(3.86 to 4.75)	1.12(0.9 to 1.32)
United States of America	Liver cancer due to alcohol use	1875(1656 to 2089)	7384(6001 to 8768)	2.94(2.4 to 3.42)	0.59(0.52 to 0.65)	1.35(1.1 to 1.6)	1.28(0.97 to 1.55)
United States of America	Liver cancer due to hepatitis B	848(735 to 976)	2742(2216 to 3353)	2.24(1.82 to 2.62)	0.28(0.24 to 0.32)	0.53(0.43 to 0.65)	0.9(0.66 to 1.12)
United States of America	Liver cancer due to hepatitis C	2436(2192 to 2666)	9231(7939 to 10567)	2.79(2.4 to 3.13)	0.76(0.68 to 0.83)	1.63(1.4 to 1.87)	1.16(0.94 to 1.35)
United States of America	Liver cancer due to NASH	656(564 to 764)	2534(2081 to 3047)	2.86(2.45 to 3.23)	0.2(0.17 to 0.23)	0.45(0.37 to 0.54)	1.25(1 to 1.45)
United States of America	Liver cancer due to other causes	640(559 to 723)	1915(1597 to 2255)	1.99(1.68 to 2.28)	0.21(0.19 to 0.24)	0.37(0.31 to 0.44)	0.74(0.57 to 0.9)
United States Virgin Islands	Liver cancer	4(3 to 4)	5(4 to 6)	0.35(0.02 to 0.76)	4.29(3.54 to 5.05)	2.6(2.17 to 3.07)	-0.39(-0.54 to -0.21)
United States Virgin Islands	Liver cancer due to alcohol use	1(1 to 2)	2(1 to 2)	0.57(0.15 to 1.1)	1.33(0.94 to 1.81)	0.9(0.64 to 1.22)	-0.33(-0.5 to -0.1)
United States Virgin Islands	Liver cancer due to hepatitis B	1(1 to 1)	1(1 to 2)	0.09(-0.21 to 0.48)	1.18(0.82 to 1.64)	0.66(0.45 to 0.93)	-0.44(-0.59 to -0.24)
United States Virgin Islands	Liver cancer due to hepatitis C	1(1 to 1)	1(1 to 1)	0.35(0.02 to 0.76)	1.05(0.72 to 1.46)	0.58(0.39 to 0.79)	-0.45(-0.58 to -0.29)
United States Virgin Islands	Liver cancer due to NASH	0(0 to 1)	1(0 to 1)	0.74(0.29 to 1.39)	0.44(0.3 to 0.63)	0.33(0.23 to 0.47)	-0.26(-0.44 to 0)

United States Virgin Islands	Liver cancer due to other causes	0(0 to 0)	0(0 to 0)	-0.02(-0.28 to 0.32)	0.28(0.19 to 0.39)	0.14(0.1 to 0.2)	-0.49(-0.62 to -0.34)
Uruguay	Liver cancer	56(49 to 63)	128(116 to 142)	1.29(0.93 to 1.69)	1.43(1.25 to 1.61)	2.36(2.12 to 2.61)	0.65(0.39 to 0.94)
Uruguay	Liver cancer due to alcohol use	16(11 to 22)	38(27 to 50)	1.35(0.88 to 1.91)	0.41(0.29 to 0.55)	0.72(0.51 to 0.94)	0.75(0.41 to 1.17)
Uruguay	Liver cancer due to hepatitis B	11(7 to 16)	21(15 to 30)	0.98(0.61 to 1.42)	0.28(0.19 to 0.41)	0.42(0.29 to 0.59)	0.51(0.22 to 0.84)
Uruguay	Liver cancer due to hepatitis C	21(15 to 27)	49(35 to 62)	1.35(0.98 to 1.75)	0.52(0.38 to 0.67)	0.85(0.61 to 1.1)	0.62(0.37 to 0.88)
Uruguay	Liver cancer due to NASH	5(3 to 7)	13(9 to 19)	1.84(1.32 to 2.47)	0.12(0.08 to 0.17)	0.23(0.16 to 0.33)	0.98(0.63 to 1.41)
Uruguay	Liver cancer due to other causes	4(3 to 5)	7(5 to 10)	0.9(0.56 to 1.27)	0.1(0.07 to 0.13)	0.14(0.1 to 0.19)	0.39(0.16 to 0.63)
Uzbekistan	Liver cancer	113(100 to 123)	1325(1084 to 1587)	10.76(8.28 to 13.62)	0.95(0.86 to 1.02)	6.68(5.58 to 7.83)	6.04(4.79 to 7.5)
Uzbekistan	Liver cancer due to alcohol use	24(17 to 31)	376(253 to 522)	14.86(11.39 to 18.6)	0.22(0.15 to 0.3)	1.87(1.28 to 2.55)	7.51(5.9 to 9.37)
Uzbekistan	Liver cancer due to hepatitis B	25(18 to 33)	368(256 to 523)	13.79(10.55 to 17.41)	0.21(0.15 to 0.29)	1.5(1.04 to 2.11)	6.09(4.7 to 7.74)
Uzbekistan	Liver cancer due to hepatitis C	39(30 to 48)	417(280 to 573)	9.74(7.55 to 12.41)	0.38(0.29 to 0.47)	2.48(1.8 to 3.26)	5.6(4.5 to 6.86)
Uzbekistan	Liver cancer due to NASH	6(4 to 9)	97(63 to 141)	14.64(10.78 to 19.46)	0.06(0.04 to 0.08)	0.55(0.37 to 0.8)	8.35(6.5 to 10.67)
Uzbekistan	Liver cancer due to other causes	19(12 to 25)	67(46 to 94)	2.53(1.04 to 5.5)	0.08(0.05 to 0.11)	0.28(0.19 to 0.4)	2.41(1.17 to 4.41)
Vanuatu	Liver cancer	6(4 to 10)	16(11 to 23)	1.57(0.74 to 2.82)	9.62(6.08 to 14.29)	9.36(6.41 to 13.2)	-0.03(-0.33 to 0.43)

Vanuatu	Liver cancer due to alcohol use	1(0 to 1)	2(1 to 3)	1.78(0.88 to 3.21)	1.17(0.59 to 2.05)	1.22(0.66 to 2.01)	0.04(-0.29 to 0.54)
Vanuatu	Liver cancer due to hepatitis B	4(2 to 6)	9(6 to 14)	1.46(0.61 to 2.71)	5.17(3.12 to 8.08)	4.86(3.15 to 7.21)	-0.06(-0.38 to 0.41)
Vanuatu	Liver cancer due to hepatitis C	1(1 to 2)	3(2 to 5)	1.61(0.81 to 2.88)	2.22(1.28 to 3.66)	2.12(1.27 to 3.15)	-0.05(-0.32 to 0.38)
Vanuatu	Liver cancer due to NASH	0(0 to 1)	1(1 to 2)	2.12(1.14 to 3.63)	0.68(0.37 to 1.12)	0.79(0.47 to 1.21)	0.16(-0.19 to 0.69)
Vanuatu	Liver cancer due to other causes	0(0 to 0)	1(0 to 1)	1.5(0.72 to 2.75)	0.38(0.21 to 0.62)	0.38(0.23 to 0.58)	0.01(-0.32 to 0.52)
Venezuela (Bolivarian Republic of)	Liver cancer	639(608 to 670)	688(531 to 891)	0.08(-0.18 to 0.39)	6.75(6.38 to 7.09)	2.44(1.88 to 3.15)	-0.64(-0.72 to -0.54)
Venezuela (Bolivarian Republic of)	Liver cancer due to alcohol use	213(156 to 268)	244(162 to 347)	0.14(-0.13 to 0.52)	2.27(1.66 to 2.88)	0.85(0.57 to 1.21)	-0.62(-0.72 to -0.5)
Venezuela (Bolivarian Republic of)	Liver cancer due to hepatitis B	109(76 to 151)	101(63 to 157)	-0.07(-0.31 to 0.23)	1.05(0.71 to 1.48)	0.34(0.22 to 0.53)	-0.67(-0.75 to -0.57)
Venezuela (Bolivarian Republic of)	Liver cancer due to hepatitis C	231(173 to 288)	249(169 to 346)	0.08(-0.17 to 0.38)	2.58(1.95 to 3.23)	0.9(0.62 to 1.25)	-0.65(-0.73 to -0.55)
Venezuela (Bolivarian Republic of)	Liver cancer due to NASH	44(31 to 62)	58(38 to 91)	0.31(-0.03 to 0.74)	0.48(0.33 to 0.67)	0.21(0.13 to 0.32)	-0.56(-0.67 to -0.42)
Venezuela (Bolivarian Republic of)	Liver cancer due to other causes	42(32 to 55)	35(23 to 52)	-0.16(-0.38 to 0.1)	0.37(0.27 to 0.51)	0.13(0.08 to 0.18)	-0.66(-0.74 to -0.55)

Viet Nam	Liver cancer	1577(1182 to 1983)	2387(1839 to 2982)	0.51(0.03 to 1.25)	3.97(2.95 to 4.96)	2.75(2.15 to 3.4)	-0.31(-0.52 to 0.05)
Viet Nam	Liver cancer due to alcohol use	287(174 to 444)	572(351 to 842)	1(0.34 to 2.06)	0.72(0.44 to 1.12)	0.66(0.4 to 0.97)	-0.09(-0.4 to 0.41)
Viet Nam	Liver cancer due to hepatitis B	715(488 to 985)	936(634 to 1329)	0.31(-0.13 to 0.98)	1.74(1.19 to 2.4)	1(0.68 to 1.42)	-0.43(-0.61 to -0.13)
Viet Nam	Liver cancer due to hepatitis C	383(213 to 595)	579(333 to 897)	0.51(0.05 to 1.38)	1.03(0.6 to 1.6)	0.72(0.43 to 1.11)	-0.3(-0.51 to 0.1)
Viet Nam	Liver cancer due to NASH	128(79 to 193)	218(139 to 326)	0.7(0.15 to 1.69)	0.34(0.21 to 0.52)	0.27(0.17 to 0.4)	-0.21(-0.45 to 0.25)
Viet Nam	Liver cancer due to other causes	64(45 to 90)	81(55 to 122)	0.27(-0.06 to 0.76)	0.14(0.09 to 0.2)	0.1(0.07 to 0.14)	-0.29(-0.48 to 0)
Yemen	Liver cancer	159(100 to 234)	423(309 to 575)	1.66(0.87 to 2.87)	3.42(2.16 to 5.05)	3.42(2.49 to 4.57)	0(-0.28 to 0.4)
Yemen	Liver cancer due to alcohol use	10(5 to 18)	29(16 to 48)	1.96(1.03 to 3.43)	0.21(0.11 to 0.37)	0.24(0.13 to 0.4)	0.13(-0.22 to 0.66)
Yemen	Liver cancer due to hepatitis B	55(32 to 89)	137(86 to 207)	1.49(0.68 to 2.77)	1.06(0.62 to 1.72)	0.98(0.61 to 1.51)	-0.07(-0.35 to 0.38)
Yemen	Liver cancer due to hepatitis C	68(40 to 108)	184(123 to 264)	1.71(0.91 to 2.85)	1.66(0.98 to 2.6)	1.66(1.13 to 2.33)	0(-0.29 to 0.41)
Yemen	Liver cancer due to NASH	11(6 to 18)	35(21 to 55)	2.18(1.19 to 3.69)	0.25(0.13 to 0.43)	0.3(0.18 to 0.46)	0.16(-0.19 to 0.67)
Yemen	Liver cancer due to other causes	16(9 to 25)	39(25 to 56)	1.48(0.68 to 2.78)	0.24(0.14 to 0.38)	0.25(0.15 to 0.36)	0.03(-0.27 to 0.51)
Zambia	Liver cancer	80(60 to 112)	202(156 to 252)	1.52(0.69 to 2.7)	2.6(1.95 to 3.76)	2.99(2.37 to 3.67)	0.15(-0.25 to 0.64)
Zambia	Liver cancer due to alcohol use	18(11 to 32)	50(33 to 70)	1.82(0.66 to 3.27)	0.69(0.41 to 1.23)	0.83(0.55 to 1.16)	0.21(-0.29 to 0.84)

Zambia	Liver cancer due to hepatitis B	23(15 to 35)	60(41 to 84)	1.64(0.68 to 2.94)	0.71(0.46 to 1.11)	0.77(0.52 to 1.1)	0.09(-0.32 to 0.6)
Zambia	Liver cancer due to hepatitis C	15(9 to 22)	39(25 to 55)	1.68(0.81 to 2.8)	0.64(0.41 to 0.97)	0.75(0.51 to 1.04)	0.17(-0.22 to 0.63)
Zambia	Liver cancer due to NASH	8(5 to 13)	24(16 to 36)	1.95(0.95 to 3.3)	0.32(0.21 to 0.5)	0.41(0.27 to 0.6)	0.27(-0.16 to 0.83)
Zambia	Liver cancer due to other causes	17(10 to 28)	29(20 to 40)	0.7(-0.09 to 2.13)	0.24(0.16 to 0.35)	0.24(0.16 to 0.34)	-0.01(-0.35 to 0.47)
Zimbabwe	Liver cancer	510(396 to 794)	1016(752 to 1381)	0.99(0.34 to 1.76)	12.16(9.51 to 18.38)	14.03(10.58 to 18.67)	0.15(-0.22 to 0.6)
Zimbabwe	Liver cancer due to alcohol use	85(49 to 167)	153(92 to 255)	0.79(0.21 to 1.54)	2.05(1.18 to 3.94)	2.14(1.3 to 3.49)	0.04(-0.29 to 0.47)
Zimbabwe	Liver cancer due to hepatitis B	215(144 to 366)	378(251 to 580)	0.76(0.19 to 1.5)	4.63(3.1 to 7.81)	4.49(2.96 to 6.87)	-0.03(-0.34 to 0.35)
Zimbabwe	Liver cancer due to hepatitis C	145(99 to 209)	333(220 to 473)	1.3(0.6 to 2.23)	3.98(2.82 to 5.67)	5.36(3.65 to 7.57)	0.35(-0.06 to 0.91)
Zimbabwe	Liver cancer due to NASH	38(24 to 61)	88(57 to 133)	1.34(0.6 to 2.35)	0.97(0.62 to 1.58)	1.31(0.84 to 2.02)	0.36(-0.06 to 0.94)
Zimbabwe	Liver cancer due to other causes	27(18 to 42)	64(42 to 92)	1.32(0.57 to 2.33)	0.54(0.35 to 0.84)	0.72(0.45 to 1.1)	0.35(-0.1 to 0.93)

Table S5. The incidence of liver cancer and underlying etiologies between 1990 and 2019 at national level, both sexes

region	Cause	Case in 1990	Case in 2019	Change in absolute number (95% UI)	ASR in 1990	ASR in 2019	change in ASR per 100 000 population (95% UI)
Afghanistan	Liver cancer	843(646 to 1082)	1408(1057 to 1848)	0.67(0.19 to 1.33)	11.48(8.93 to 14.53)	9.82(7.63 to 12.44)	-0.14(-0.38 to 0.16)
Afghanistan	Liver cancer due to alcohol use	69(40 to 107)	102(62 to 158)	0.48(0.04 to 1.17)	0.95(0.56 to 1.47)	0.83(0.5 to 1.26)	-0.13(-0.38 to 0.2)
Afghanistan	Liver cancer due to hepatitis B	339(237 to 473)	606(424 to 850)	0.79(0.24 to 1.59)	4.41(3.12 to 6.09)	3.6(2.49 to 4.97)	-0.18(-0.42 to 0.14)
Afghanistan	Liver cancer due to hepatitis C	304(204 to 428)	439(291 to 622)	0.44(0.02 to 1.04)	4.43(3.04 to 6.04)	3.79(2.62 to 5.31)	-0.14(-0.37 to 0.16)
Afghanistan	Liver cancer due to NASH	62(40 to 93)	116(74 to 168)	0.87(0.31 to 1.7)	0.86(0.57 to 1.28)	0.86(0.54 to 1.26)	0(-0.28 to 0.4)
Afghanistan	Liver cancer due to other causes	69(46 to 100)	145(101 to 208)	1.1(0.48 to 2.03)	0.84(0.55 to 1.22)	0.75(0.49 to 1.09)	-0.1(-0.37 to 0.28)
Albania	Liver cancer	224(209 to 240)	281(205 to 376)	0.26(-0.09 to 0.67)	10.85(10.1 to 11.6)	6.58(4.82 to 8.76)	-0.39(-0.56 to -0.19)
Albania	Liver cancer due to alcohol use	66(48 to 85)	107(67 to 157)	0.63(0.15 to 1.26)	3.21(2.31 to 4.17)	2.44(1.53 to 3.58)	-0.24(-0.47 to 0.04)
Albania	Liver cancer due to hepatitis B	70(52 to 92)	68(42 to 102)	-0.04(-0.32 to 0.31)	3.15(2.29 to 4.16)	1.66(1.06 to 2.45)	-0.47(-0.62 to -0.28)
Albania	Liver cancer due to hepatitis C	61(43 to 81)	74(47 to 111)	0.21(-0.12 to 0.65)	3.23(2.31 to 4.22)	1.69(1.08 to 2.52)	-0.48(-0.61 to -0.29)

Albania	Liver cancer due to NASH	16(11 to 22)	23(14 to 35)	0.44(0.03 to 0.98)	0.79(0.55 to 1.12)	0.52(0.33 to 0.8)	-0.34(-0.52 to -0.09)
Albania	Liver cancer due to other causes	11(8 to 14)	10(6 to 15)	-0.11(-0.38 to 0.2)	0.47(0.33 to 0.63)	0.27(0.18 to 0.39)	-0.43(-0.57 to -0.24)
Algeria	Liver cancer	207(168 to 256)	720(562 to 908)	2.47(1.52 to 3.64)	1.68(1.38 to 2.06)	2.21(1.73 to 2.78)	0.31(-0.04 to 0.72)
Algeria	Liver cancer due to alcohol use	19(11 to 29)	72(43 to 111)	2.87(1.69 to 4.47)	0.16(0.09 to 0.24)	0.22(0.13 to 0.34)	0.44(0 to 1)
Algeria	Liver cancer due to hepatitis B	79(57 to 106)	252(176 to 350)	2.18(1.28 to 3.43)	0.59(0.42 to 0.8)	0.71(0.49 to 0.99)	0.2(-0.14 to 0.64)
Algeria	Liver cancer due to hepatitis C	69(47 to 96)	260(181 to 357)	2.75(1.66 to 4.06)	0.66(0.47 to 0.87)	0.87(0.61 to 1.18)	0.31(-0.05 to 0.71)
Algeria	Liver cancer due to NASH	17(11 to 25)	79(51 to 119)	3.72(2.27 to 5.67)	0.15(0.1 to 0.22)	0.25(0.16 to 0.37)	0.71(0.22 to 1.35)
Algeria	Liver cancer due to other causes	23(17 to 31)	57(40 to 79)	1.43(0.75 to 2.33)	0.13(0.09 to 0.17)	0.16(0.11 to 0.22)	0.24(-0.07 to 0.64)
American Samoa	Liver cancer	1(1 to 2)	3(3 to 4)	1.55(0.93 to 2.4)	5.47(4.19 to 6.72)	6.88(5.69 to 8.35)	0.26(-0.04 to 0.68)
American Samoa	Liver cancer due to alcohol use	0(0 to 0)	0(0 to 1)	1.68(0.98 to 2.68)	0.59(0.35 to 0.91)	0.76(0.48 to 1.13)	0.27(-0.05 to 0.71)
American Samoa	Liver cancer due to hepatitis B	1(1 to 1)	2(1 to 2)	1.48(0.82 to 2.42)	2.83(1.96 to 3.74)	3.64(2.78 to 4.75)	0.28(-0.04 to 0.75)
American Samoa	Liver cancer due to hepatitis C	0(0 to 0)	1(0 to 1)	1.58(1 to 2.42)	1.24(0.81 to 1.72)	1.4(0.95 to 1.91)	0.13(-0.12 to 0.47)

American Samoa	Liver cancer due to NASH	0(0 to 0)	0(0 to 1)	1.98(1.24 to 2.99)	0.57(0.37 to 0.82)	0.8(0.55 to 1.13)	0.4(0.06 to 0.86)
American Samoa	Liver cancer due to other causes	0(0 to 0)	0(0 to 0)	1.08(0.53 to 1.8)	0.24(0.16 to 0.36)	0.28(0.19 to 0.41)	0.17(-0.13 to 0.6)
Andorra	Liver cancer	5(4 to 7)	15(11 to 20)	1.87(0.9 to 3.2)	9.57(7.16 to 12.99)	10.95(8.23 to 14.37)	0.14(-0.23 to 0.67)
Andorra	Liver cancer due to alcohol use	2(2 to 3)	6(4 to 8)	1.65(0.74 to 3.02)	3.99(2.72 to 5.85)	4.37(2.86 to 6.13)	0.1(-0.28 to 0.63)
Andorra	Liver cancer due to hepatitis B	1(0 to 1)	2(1 to 3)	1.57(0.66 to 2.85)	1.3(0.8 to 2)	1.46(0.92 to 2.22)	0.12(-0.27 to 0.66)
Andorra	Liver cancer due to hepatitis C	2(1 to 3)	5(4 to 8)	2.22(1.11 to 3.75)	3.25(2.12 to 4.91)	3.84(2.5 to 5.45)	0.18(-0.22 to 0.72)
Andorra	Liver cancer due to NASH	0(0 to 0)	1(1 to 2)	2.56(1.3 to 4.34)	0.53(0.32 to 0.83)	0.72(0.45 to 1.14)	0.37(-0.11 to 1.01)
Andorra	Liver cancer due to other causes	0(0 to 0)	1(0 to 1)	1.68(0.71 to 3.08)	0.49(0.32 to 0.72)	0.56(0.37 to 0.84)	0.15(-0.25 to 0.71)
Angola	Liver cancer	112(84 to 147)	294(226 to 377)	1.61(0.79 to 2.77)	2.54(1.94 to 3.28)	2.39(1.92 to 2.99)	-0.06(-0.33 to 0.31)
Angola	Liver cancer due to alcohol use	13(8 to 21)	45(29 to 67)	2.39(1.24 to 4.07)	0.35(0.21 to 0.55)	0.41(0.26 to 0.61)	0.18(-0.21 to 0.76)
Angola	Liver cancer due to hepatitis B	32(21 to 46)	80(53 to 115)	1.5(0.61 to 2.72)	0.64(0.42 to 0.93)	0.54(0.36 to 0.78)	-0.15(-0.44 to 0.25)
Angola	Liver cancer due to hepatitis C	43(30 to 60)	110(77 to 149)	1.53(0.77 to 2.62)	1.25(0.88 to 1.69)	1.13(0.82 to 1.48)	-0.1(-0.35 to 0.24)

Angola	Liver cancer due to NASH	6(4 to 9)	19(12 to 28)	2.29(1.21 to 3.85)	0.15(0.09 to 0.22)	0.17(0.11 to 0.25)	0.15(-0.19 to 0.61)
Angola	Liver cancer due to other causes	18(10 to 33)	40(23 to 67)	1.23(0.04 to 3.34)	0.16(0.1 to 0.24)	0.15(0.09 to 0.22)	-0.09(-0.48 to 0.44)
Antigua and Barbuda	Liver cancer	4(4 to 5)	3(2 to 3)	-0.34(-0.45 to -0.21)	7.7(6.99 to 8.51)	2.69(2.29 to 3.13)	-0.65(-0.71 to -0.58)
Antigua and Barbuda	Liver cancer due to alcohol use	1(1 to 2)	1(1 to 1)	-0.27(-0.41 to -0.09)	2.46(1.76 to 3.2)	0.95(0.67 to 1.28)	-0.61(-0.69 to -0.52)
Antigua and Barbuda	Liver cancer due to hepatitis B	1(1 to 2)	1(1 to 1)	-0.42(-0.53 to -0.29)	2.42(1.76 to 3.25)	0.69(0.48 to 0.96)	-0.72(-0.77 to -0.65)
Antigua and Barbuda	Liver cancer due to hepatitis C	1(1 to 1)	1(0 to 1)	-0.38(-0.49 to -0.25)	1.75(1.19 to 2.38)	0.63(0.43 to 0.88)	-0.64(-0.7 to -0.57)
Antigua and Barbuda	Liver cancer due to NASH	0(0 to 0)	0(0 to 0)	-0.22(-0.38 to -0.02)	0.58(0.41 to 0.82)	0.26(0.18 to 0.37)	-0.56(-0.64 to -0.45)
Antigua and Barbuda	Liver cancer due to other causes	0(0 to 0)	0(0 to 0)	-0.38(-0.49 to -0.21)	0.49(0.35 to 0.66)	0.17(0.12 to 0.24)	-0.64(-0.71 to -0.55)
Argentina	Liver cancer	453(395 to 514)	1105(867 to 1381)	1.44(0.88 to 2.17)	1.4(1.22 to 1.59)	2.05(1.61 to 2.56)	0.46(0.12 to 0.89)
Argentina	Liver cancer due to alcohol use	156(109 to 206)	367(242 to 510)	1.34(0.72 to 2.14)	0.48(0.33 to 0.63)	0.68(0.45 to 0.94)	0.41(0.03 to 0.89)
Argentina	Liver cancer due to hepatitis B	81(56 to 115)	187(122 to 284)	1.31(0.72 to 2.08)	0.25(0.17 to 0.35)	0.36(0.23 to 0.54)	0.43(0.06 to 0.92)
Argentina	Liver cancer due to hepatitis C	149(107 to 202)	385(263 to 529)	1.59(0.99 to 2.36)	0.47(0.34 to 0.63)	0.7(0.48 to 0.97)	0.49(0.14 to 0.92)

Argentina	Liver cancer due to NASH	33(22 to 49)	100(64 to 148)	2.08(1.34 to 3.15)	0.1(0.07 to 0.15)	0.18(0.12 to 0.27)	0.79(0.35 to 1.38)
Argentina	Liver cancer due to other causes	34(26 to 45)	66(44 to 95)	0.94(0.46 to 1.54)	0.1(0.08 to 0.14)	0.13(0.09 to 0.19)	0.28(-0.01 to 0.65)
Armenia	Liver cancer	30(27 to 32)	271(225 to 323)	8.18(6.42 to 10.11)	1.09(1.02 to 1.18)	6.6(5.47 to 7.85)	5.05(3.92 to 6.3)
Armenia	Liver cancer due to alcohol use	7(5 to 9)	81(56 to 110)	11.2(8.62 to 14.23)	0.25(0.18 to 0.34)	1.94(1.35 to 2.66)	6.68(5.14 to 8.55)
Armenia	Liver cancer due to hepatitis B	7(5 to 9)	56(37 to 81)	6.88(5.2 to 8.79)	0.25(0.18 to 0.33)	1.39(0.93 to 1.98)	4.58(3.47 to 5.84)
Armenia	Liver cancer due to hepatitis C	10(7 to 12)	103(73 to 137)	9.45(7.6 to 11.61)	0.4(0.3 to 0.5)	2.49(1.78 to 3.27)	5.21(4.08 to 6.46)
Armenia	Liver cancer due to NASH	2(1 to 2)	21(14 to 30)	13.02(9.96 to 16.53)	0.06(0.04 to 0.09)	0.51(0.35 to 0.74)	7.51(5.73 to 9.68)
Armenia	Liver cancer due to other causes	4(3 to 7)	10(6 to 14)	1.21(0.26 to 2.86)	0.13(0.09 to 0.19)	0.27(0.18 to 0.4)	1.09(0.19 to 2.55)
Australia	Liver cancer	384(368 to 400)	1799(1388 to 2309)	3.69(2.65 to 5)	1.98(1.89 to 2.06)	4.52(3.48 to 5.84)	1.29(0.77 to 1.94)
Australia	Liver cancer due to alcohol use	165(126 to 200)	707(486 to 989)	3.3(2.25 to 4.56)	0.83(0.64 to 1.01)	1.78(1.21 to 2.5)	1.13(0.62 to 1.77)
Australia	Liver cancer due to hepatitis B	60(42 to 84)	238(148 to 358)	2.94(1.95 to 4.2)	0.32(0.22 to 0.43)	0.65(0.41 to 0.98)	1.05(0.54 to 1.74)
Australia	Liver cancer due to hepatitis C	110(79 to 143)	581(388 to 825)	4.3(3.12 to 5.74)	0.56(0.41 to 0.72)	1.39(0.93 to 1.97)	1.48(0.92 to 2.15)

Australia	Liver cancer due to NASH	28(20 to 40)	191(122 to 289)	5.74(4.05 to 8.03)	0.14(0.1 to 0.2)	0.46(0.3 to 0.7)	2.21(1.41 to 3.3)
Australia	Liver cancer due to other causes	21(16 to 27)	82(53 to 123)	2.94(1.95 to 4.13)	0.12(0.1 to 0.15)	0.24(0.16 to 0.35)	1.01(0.53 to 1.61)
Austria	Liver cancer	398(379 to 418)	939(753 to 1173)	1.36(0.91 to 1.97)	3.48(3.31 to 3.66)	5.5(4.41 to 6.89)	0.58(0.26 to 1)
Austria	Liver cancer due to alcohol use	210(174 to 245)	499(366 to 663)	1.37(0.87 to 2.05)	1.84(1.53 to 2.14)	2.94(2.14 to 3.9)	0.59(0.25 to 1.04)
Austria	Liver cancer due to hepatitis B	31(21 to 45)	62(38 to 97)	1(0.54 to 1.57)	0.3(0.21 to 0.42)	0.41(0.26 to 0.62)	0.36(0.05 to 0.77)
Austria	Liver cancer due to hepatitis C	119(88 to 155)	289(198 to 412)	1.42(0.93 to 2.05)	0.99(0.73 to 1.29)	1.6(1.1 to 2.3)	0.61(0.28 to 1.03)
Austria	Liver cancer due to NASH	17(12 to 25)	47(29 to 72)	1.75(1.16 to 2.49)	0.14(0.1 to 0.2)	0.26(0.17 to 0.4)	0.86(0.45 to 1.35)
Austria	Liver cancer due to other causes	20(15 to 27)	42(27 to 61)	1.09(0.62 to 1.63)	0.21(0.16 to 0.27)	0.3(0.2 to 0.42)	0.43(0.13 to 0.81)
Azerbaijan	Liver cancer	51(46 to 58)	358(274 to 472)	6.02(4.25 to 8.64)	0.98(0.89 to 1.1)	4.07(3.11 to 5.46)	3.13(2.07 to 4.82)
Azerbaijan	Liver cancer due to alcohol use	11(7 to 15)	98(61 to 150)	8.19(5.57 to 12.36)	0.22(0.15 to 0.3)	1.08(0.66 to 1.65)	3.89(2.5 to 6.24)
Azerbaijan	Liver cancer due to hepatitis B	13(9 to 17)	94(63 to 138)	6.37(4.44 to 9.37)	0.23(0.17 to 0.31)	0.92(0.61 to 1.36)	2.95(1.94 to 4.54)
Azerbaijan	Liver cancer due to hepatitis C	16(12 to 21)	121(79 to 178)	6.37(4.34 to 9.23)	0.36(0.27 to 0.45)	1.52(1.01 to 2.26)	3.25(2.04 to 5.09)
Azerbaijan	Liver cancer due to NASH	3(2 to 4)	27(17 to 41)	8.84(6.16 to 12.74)	0.06(0.04 to 0.08)	0.33(0.21 to 0.53)	4.72(3.08 to 7.31)

Azerbaijan	Liver cancer due to other causes	8(6 to 13)	18(12 to 29)	1.17(0.44 to 2.22)	0.11(0.09 to 0.16)	0.2(0.14 to 0.32)	0.82(0.24 to 1.7)
Bahamas	Liver cancer	13(11 to 15)	12(10 to 15)	-0.06(-0.26 to 0.19)	8.15(7.18 to 9.12)	3.1(2.56 to 3.81)	-0.62(-0.7 to -0.52)
Bahamas	Liver cancer due to alcohol use	5(3 to 6)	4(3 to 6)	-0.13(-0.32 to 0.14)	3.03(2.22 to 3.93)	1.04(0.72 to 1.44)	-0.65(-0.73 to -0.55)
Bahamas	Liver cancer due to hepatitis B	4(3 to 6)	4(3 to 5)	-0.11(-0.31 to 0.16)	2.41(1.78 to 3.25)	0.89(0.63 to 1.24)	-0.63(-0.71 to -0.52)
Bahamas	Liver cancer due to hepatitis C	2(2 to 3)	2(2 to 3)	0.04(-0.17 to 0.31)	1.61(1.1 to 2.21)	0.66(0.44 to 0.91)	-0.59(-0.67 to -0.48)
Bahamas	Liver cancer due to NASH	1(1 to 1)	1(1 to 2)	0.2(-0.06 to 0.56)	0.61(0.43 to 0.86)	0.3(0.21 to 0.44)	-0.51(-0.61 to -0.37)
Bahamas	Liver cancer due to other causes	1(1 to 1)	1(1 to 1)	-0.09(-0.3 to 0.17)	0.49(0.35 to 0.67)	0.2(0.14 to 0.28)	-0.58(-0.68 to -0.46)
Bahrain	Liver cancer	9(7 to 11)	44(34 to 57)	3.96(2.68 to 5.71)	5.62(4.72 to 6.58)	5.53(4.39 to 6.91)	-0.02(-0.25 to 0.31)
Bahrain	Liver cancer due to alcohol use	1(1 to 2)	4(3 to 7)	3.41(2.14 to 5.27)	0.65(0.4 to 0.97)	0.53(0.32 to 0.83)	-0.18(-0.4 to 0.13)
Bahrain	Liver cancer due to hepatitis B	4(3 to 5)	17(11 to 24)	3.67(2.37 to 5.57)	1.8(1.29 to 2.49)	1.5(1.03 to 2.16)	-0.17(-0.38 to 0.14)
Bahrain	Liver cancer due to hepatitis C	3(2 to 4)	15(10 to 21)	4.12(2.75 to 5.89)	2.23(1.6 to 2.91)	2.35(1.67 to 3.14)	0.05(-0.22 to 0.39)
Bahrain	Liver cancer due to NASH	1(1 to 1)	6(4 to 9)	5.51(3.76 to 8.05)	0.61(0.41 to 0.91)	0.83(0.55 to 1.24)	0.34(-0.02 to 0.85)

Bahrain	Liver cancer due to other causes	1(0 to 1)	3(2 to 4)	3.58(2.4 to 5.05)	0.31(0.22 to 0.46)	0.32(0.22 to 0.48)	0.02(-0.22 to 0.36)
Bangladesh	Liver cancer	1742(1384 to 2138)	3405(2661 to 4240)	0.95(0.46 to 1.69)	3.11(2.55 to 3.77)	2.58(2.03 to 3.21)	-0.17(-0.38 to 0.11)
Bangladesh	Liver cancer due to alcohol use	324(216 to 466)	707(441 to 1042)	1.18(0.54 to 2.07)	0.67(0.44 to 0.96)	0.54(0.33 to 0.79)	-0.19(-0.42 to 0.13)
Bangladesh	Liver cancer due to hepatitis B	564(404 to 762)	809(566 to 1142)	0.43(0.01 to 1.03)	0.87(0.62 to 1.18)	0.57(0.39 to 0.82)	-0.34(-0.52 to -0.09)
Bangladesh	Liver cancer due to hepatitis C	535(379 to 730)	1384(973 to 1845)	1.59(0.89 to 2.54)	1.19(0.85 to 1.61)	1.11(0.78 to 1.46)	-0.07(-0.31 to 0.26)
Bangladesh	Liver cancer due to NASH	118(81 to 165)	267(179 to 390)	1.27(0.58 to 2.16)	0.21(0.14 to 0.29)	0.2(0.13 to 0.3)	-0.02(-0.29 to 0.34)
Bangladesh	Liver cancer due to other causes	201(133 to 293)	238(169 to 328)	0.18(-0.19 to 0.79)	0.18(0.13 to 0.25)	0.16(0.11 to 0.22)	-0.12(-0.37 to 0.25)
Barbados	Liver cancer	8(6 to 10)	13(10 to 15)	0.57(0.13 to 1.29)	2.78(2.05 to 3.58)	2.63(2.17 to 3.14)	-0.05(-0.31 to 0.37)
Barbados	Liver cancer due to alcohol use	3(2 to 4)	5(3 to 6)	0.78(0.21 to 1.75)	0.9(0.57 to 1.37)	0.95(0.67 to 1.26)	0.05(-0.28 to 0.63)
Barbados	Liver cancer due to hepatitis B	2(1 to 3)	3(2 to 4)	0.37(-0.06 to 1.1)	0.77(0.49 to 1.16)	0.61(0.43 to 0.86)	-0.21(-0.46 to 0.2)
Barbados	Liver cancer due to hepatitis C	2(1 to 3)	3(2 to 4)	0.52(0.13 to 1.08)	0.62(0.4 to 0.91)	0.58(0.39 to 0.82)	-0.06(-0.3 to 0.29)
Barbados	Liver cancer due to NASH	1(0 to 1)	1(1 to 2)	0.79(0.33 to 1.53)	0.24(0.16 to 0.36)	0.27(0.19 to 0.39)	0.12(-0.17 to 0.57)
Barbados	Liver cancer due to other causes	1(0 to 1)	1(1 to 1)	0.26(-0.07 to 0.73)	0.24(0.16 to 0.36)	0.23(0.15 to	-0.08(-0.28 to 0.24)

						0.32)	
Belarus	Liver cancer	163(154 to 173)	381(275 to 512)	1.33(0.68 to 2.14)	1.29(1.22 to 1.38)	2.49(1.81 to 3.34)	0.93(0.39 to 1.58)
Belarus	Liver cancer due to alcohol use	58(43 to 73)	165(106 to 241)	1.82(0.95 to 2.98)	0.45(0.34 to 0.55)	1.04(0.67 to 1.53)	1.33(0.61 to 2.31)
Belarus	Liver cancer due to hepatitis B	37(27 to 50)	77(46 to 120)	1.04(0.39 to 1.88)	0.29(0.22 to 0.39)	0.52(0.32 to 0.8)	0.77(0.22 to 1.48)
Belarus	Liver cancer due to hepatitis C	43(31 to 57)	94(61 to 137)	1.18(0.65 to 1.81)	0.33(0.24 to 0.43)	0.58(0.37 to 0.85)	0.74(0.31 to 1.25)
Belarus	Liver cancer due to NASH	11(7 to 15)	28(17 to 42)	1.58(0.91 to 2.45)	0.08(0.06 to 0.12)	0.17(0.11 to 0.27)	1.1(0.55 to 1.82)
Belarus	Liver cancer due to other causes	14(10 to 18)	18(12 to 25)	0.3(-0.1 to 0.88)	0.14(0.1 to 0.19)	0.18(0.13 to 0.25)	0.33(-0.09 to 0.95)
Belgium	Liver cancer	443(418 to 467)	928(734 to 1171)	1.09(0.66 to 1.65)	2.89(2.74 to 3.04)	4.27(3.36 to 5.42)	0.48(0.16 to 0.88)
Belgium	Liver cancer due to alcohol use	161(122 to 202)	358(242 to 495)	1.22(0.7 to 1.88)	1.04(0.79 to 1.29)	1.67(1.13 to 2.33)	0.6(0.22 to 1.07)
Belgium	Liver cancer due to hepatitis B	57(40 to 81)	112(71 to 170)	0.95(0.51 to 1.53)	0.4(0.28 to 0.55)	0.59(0.37 to 0.87)	0.46(0.11 to 0.91)
Belgium	Liver cancer due to hepatitis C	176(133 to 218)	356(248 to 484)	1.02(0.61 to 1.55)	1.11(0.84 to 1.37)	1.51(1.05 to 2.08)	0.37(0.09 to 0.74)
Belgium	Liver cancer due to NASH	26(17 to 38)	60(37 to 92)	1.28(0.77 to 1.96)	0.17(0.11 to 0.24)	0.26(0.16 to 0.4)	0.58(0.21 to 1.08)
Belgium	Liver cancer due to other causes	22(16 to 31)	42(27 to 61)	0.87(0.45 to 1.44)	0.17(0.13 to 0.23)	0.23(0.16 to	0.34(0.03 to 0.72)

						0.33)	
Belize	Liver cancer	6(5 to 6)	8(7 to 10)	0.49(0.24 to 0.78)	5.93(5.36 to 6.56)	3.04(2.6 to 3.52)	-0.49(-0.57 to -0.39)
Belize	Liver cancer due to alcohol use	2(1 to 2)	3(2 to 4)	0.68(0.37 to 1.05)	1.83(1.33 to 2.39)	1.06(0.77 to 1.38)	-0.42(-0.53 to -0.3)
Belize	Liver cancer due to hepatitis B	2(1 to 2)	3(2 to 3)	0.53(0.25 to 0.88)	1.65(1.21 to 2.22)	0.82(0.59 to 1.12)	-0.5(-0.59 to -0.4)
Belize	Liver cancer due to hepatitis C	1(1 to 2)	2(1 to 2)	0.26(0.03 to 0.52)	1.49(1.05 to 2.01)	0.66(0.45 to 0.92)	-0.56(-0.63 to -0.46)
Belize	Liver cancer due to NASH	0(0 to 1)	1(1 to 1)	0.69(0.33 to 1.11)	0.51(0.35 to 0.71)	0.3(0.21 to 0.42)	-0.41(-0.53 to -0.27)
Belize	Liver cancer due to other causes	1(0 to 1)	1(0 to 1)	0.18(-0.06 to 0.46)	0.45(0.33 to 0.6)	0.2(0.14 to 0.27)	-0.56(-0.64 to -0.45)
Benin	Liver cancer	138(111 to 175)	249(182 to 337)	0.8(0.27 to 1.57)	6.54(5.24 to 8.36)	4.78(3.57 to 6.37)	-0.27(-0.48 to 0.03)
Benin	Liver cancer due to alcohol use	23(15 to 35)	44(27 to 69)	0.92(0.35 to 1.76)	1.16(0.76 to 1.79)	0.93(0.58 to 1.45)	-0.2(-0.43 to 0.15)
Benin	Liver cancer due to hepatitis B	72(53 to 95)	122(83 to 170)	0.7(0.16 to 1.51)	3.31(2.44 to 4.44)	2.15(1.49 to 3)	-0.35(-0.55 to -0.05)
Benin	Liver cancer due to hepatitis C	23(15 to 34)	41(25 to 58)	0.74(0.28 to 1.42)	1.26(0.81 to 1.78)	0.96(0.62 to 1.35)	-0.24(-0.43 to 0.04)
Benin	Liver cancer due to NASH	10(6 to 14)	23(15 to 35)	1.37(0.68 to 2.35)	0.49(0.32 to 0.71)	0.48(0.32 to 0.73)	0(-0.29 to 0.37)
Benin	Liver cancer due to other causes	10(7 to 15)	19(13 to 28)	0.85(0.21 to 1.69)	0.32(0.22 to 0.45)	0.26(0.17 to 0.39)	-0.19(-0.42 to 0.14)

Bermuda	Liver cancer	4(4 to 5)	3(2 to 3)	-0.39(-0.51 to -0.25)	7.07(6.36 to 7.81)	2.09(1.73 to 2.56)	-0.7(-0.76 to -0.63)
Bermuda	Liver cancer due to alcohol use	2(1 to 2)	1(1 to 1)	-0.39(-0.52 to -0.24)	2.74(2.04 to 3.47)	0.8(0.57 to 1.07)	-0.71(-0.77 to -0.63)
Bermuda	Liver cancer due to hepatitis B	1(1 to 2)	1(0 to 1)	-0.44(-0.57 to -0.29)	1.77(1.29 to 2.45)	0.52(0.36 to 0.74)	-0.7(-0.77 to -0.62)
Bermuda	Liver cancer due to hepatitis C	1(1 to 1)	1(0 to 1)	-0.37(-0.49 to -0.2)	1.52(1.04 to 2.08)	0.42(0.28 to 0.61)	-0.72(-0.78 to -0.65)
Bermuda	Liver cancer due to NASH	0(0 to 1)	0(0 to 0)	-0.28(-0.44 to -0.06)	0.65(0.45 to 0.91)	0.22(0.14 to 0.32)	-0.67(-0.74 to -0.57)
Bermuda	Liver cancer due to other causes	0(0 to 0)	0(0 to 0)	-0.46(-0.59 to -0.31)	0.4(0.29 to 0.54)	0.13(0.09 to 0.18)	-0.66(-0.74 to -0.56)
Bhutan	Liver cancer	6(4 to 9)	17(12 to 24)	1.88(0.96 to 3.35)	2.18(1.47 to 3.2)	3.04(2.11 to 4.29)	0.39(-0.03 to 1.04)
Bhutan	Liver cancer due to alcohol use	2(1 to 3)	5(3 to 8)	2(0.97 to 3.59)	0.63(0.37 to 1.04)	0.87(0.51 to 1.39)	0.37(-0.08 to 1.03)
Bhutan	Liver cancer due to hepatitis B	2(1 to 3)	4(3 to 7)	1.3(0.5 to 2.61)	0.63(0.36 to 1.03)	0.74(0.43 to 1.15)	0.17(-0.22 to 0.78)
Bhutan	Liver cancer due to hepatitis C	1(1 to 2)	5(3 to 8)	2.53(1.43 to 4.23)	0.65(0.39 to 1.01)	1(0.62 to 1.54)	0.53(0.08 to 1.19)
Bhutan	Liver cancer due to NASH	0(0 to 1)	2(1 to 2)	2.87(1.61 to 4.95)	0.16(0.1 to 0.26)	0.29(0.17 to 0.46)	0.78(0.23 to 1.62)
Bhutan	Liver cancer due to other causes	0(0 to 1)	1(1 to 1)	0.88(0.12 to 3.38)	0.09(0.05 to 0.14)	0.14(0.09 to 0.21)	0.45(-0.03 to 1.53)

Bolivia (Plurinational State of)	Liver cancer	162(122 to 213)	399(296 to 520)	1.46(0.77 to 2.43)	4.92(3.72 to 6.43)	4.58(3.41 to 5.93)	-0.07(-0.33 to 0.27)
Bolivia (Plurinational State of)	Liver cancer due to alcohol use	42(26 to 62)	121(78 to 176)	1.9(1.09 to 3.1)	1.35(0.85 to 2.01)	1.42(0.92 to 2.05)	0.05(-0.24 to 0.47)
Bolivia (Plurinational State of)	Liver cancer due to hepatitis B	80(57 to 108)	176(121 to 246)	1.2(0.53 to 2.14)	2.34(1.66 to 3.18)	1.96(1.34 to 2.71)	-0.16(-0.41 to 0.18)
Bolivia (Plurinational State of)	Liver cancer due to hepatitis C	12(6 to 20)	32(17 to 53)	1.66(0.89 to 2.76)	0.42(0.23 to 0.71)	0.4(0.22 to 0.65)	-0.05(-0.32 to 0.32)
Bolivia (Plurinational State of)	Liver cancer due to NASH	12(7 to 21)	38(23 to 60)	2.13(1.2 to 3.57)	0.41(0.25 to 0.68)	0.46(0.29 to 0.73)	0.14(-0.19 to 0.62)
Bolivia (Plurinational State of)	Liver cancer due to other causes	17(11 to 25)	32(21 to 48)	0.94(0.33 to 1.77)	0.4(0.25 to 0.64)	0.35(0.22 to 0.52)	-0.13(-0.38 to 0.21)
Bosnia and Herzegovina	Liver cancer	229(212 to 246)	452(359 to 570)	0.98(0.57 to 1.5)	5.5(5.11 to 5.9)	7.53(5.99 to 9.46)	0.37(0.08 to 0.73)
Bosnia and Herzegovina	Liver cancer due to alcohol use	75(54 to 98)	159(108 to 222)	1.11(0.62 to 1.78)	1.81(1.32 to 2.35)	2.59(1.78 to 3.59)	0.44(0.11 to 0.87)
Bosnia and Herzegovina	Liver cancer due to hepatitis B	70(51 to 94)	97(63 to 147)	0.38(0.04 to 0.79)	1.57(1.14 to 2.09)	1.67(1.11 to 2.5)	0.06(-0.18 to 0.36)
Bosnia and Herzegovina	Liver cancer due to hepatitis C	59(40 to 80)	137(93 to 195)	1.31(0.76 to 1.99)	1.53(1.07 to 2.03)	2.26(1.55 to 3.18)	0.47(0.15 to 0.88)
Bosnia and Herzegovina	Liver cancer due to NASH	15(10 to 21)	43(28 to 64)	1.92(1.18 to 2.88)	0.38(0.26 to 0.55)	0.72(0.48 to 1.06)	0.89(0.43 to 1.46)

Bosnia and Herzegovina	Liver cancer due to other causes	9(6 to 13)	16(10 to 25)	0.77(0.29 to 1.34)	0.22(0.15 to 0.3)	0.29(0.19 to 0.43)	0.35(0.02 to 0.75)
Botswana	Liver cancer	6(3 to 13)	22(15 to 31)	2.81(0.65 to 7.05)	0.97(0.47 to 2.17)	1.47(1.07 to 1.95)	0.52(-0.33 to 2.15)
Botswana	Liver cancer due to alcohol use	1(0 to 3)	5(3 to 7)	3.44(0.73 to 9.48)	0.18(0.07 to 0.5)	0.31(0.19 to 0.48)	0.78(-0.28 to 3.16)
Botswana	Liver cancer due to hepatitis B	2(1 to 6)	9(6 to 14)	2.91(0.53 to 7.99)	0.35(0.14 to 0.92)	0.52(0.34 to 0.76)	0.49(-0.41 to 2.46)
Botswana	Liver cancer due to hepatitis C	2(1 to 3)	5(3 to 7)	2.23(0.67 to 5.25)	0.31(0.15 to 0.6)	0.41(0.27 to 0.59)	0.34(-0.3 to 1.59)
Botswana	Liver cancer due to NASH	0(0 to 1)	2(1 to 3)	3.36(1.13 to 7.88)	0.08(0.04 to 0.18)	0.15(0.09 to 0.23)	0.77(-0.12 to 2.58)
Botswana	Liver cancer due to other causes	0(0 to 1)	1(1 to 2)	2.21(0.71 to 4.71)	0.05(0.03 to 0.11)	0.07(0.05 to 0.11)	0.41(-0.28 to 1.68)
Brazil	Liver cancer	1794(1726 to 1850)	5555(5239 to 5841)	2.1(1.92 to 2.3)	1.96(1.88 to 2.03)	2.37(2.23 to 2.49)	0.21(0.14 to 0.28)
Brazil	Liver cancer due to alcohol use	484(418 to 551)	1712(1483 to 1942)	2.53(2.31 to 2.8)	0.54(0.47 to 0.61)	0.72(0.62 to 0.81)	0.34(0.25 to 0.44)
Brazil	Liver cancer due to hepatitis B	413(358 to 474)	1008(854 to 1188)	1.44(1.27 to 1.63)	0.41(0.35 to 0.47)	0.42(0.35 to 0.49)	0.03(-0.04 to 0.1)
Brazil	Liver cancer due to hepatitis C	640(570 to 712)	2178(1907 to 2436)	2.4(2.21 to 2.63)	0.78(0.69 to 0.86)	0.94(0.82 to 1.05)	0.21(0.15 to 0.29)
Brazil	Liver cancer due to NASH	98(83 to 114)	352(299 to 412)	2.6(2.36 to 2.85)	0.11(0.09 to 0.13)	0.15(0.13 to 0.18)	0.37(0.29 to 0.45)

Brazil	Liver cancer due to other causes	159(141 to 180)	306(265 to 350)	0.92(0.73 to 1.1)	0.13(0.12 to 0.15)	0.14(0.12 to 0.16)	0.05(-0.04 to 0.13)
Brunei Darussalam	Liver cancer	10(8 to 12)	38(32 to 46)	2.97(2.1 to 4.12)	9.68(7.88 to 11.49)	11.75(9.96 to 13.78)	0.21(-0.03 to 0.53)
Brunei Darussalam	Liver cancer due to alcohol use	1(1 to 1)	3(2 to 5)	2.9(1.9 to 4.3)	0.92(0.56 to 1.39)	1.08(0.67 to 1.6)	0.18(-0.11 to 0.55)
Brunei Darussalam	Liver cancer due to hepatitis B	5(4 to 7)	20(15 to 26)	2.91(1.96 to 4.24)	4.38(3.21 to 5.78)	5.29(3.96 to 6.89)	0.21(-0.07 to 0.6)
Brunei Darussalam	Liver cancer due to hepatitis C	3(2 to 4)	11(7 to 15)	3.06(2.17 to 4.23)	3.49(2.36 to 4.77)	4.19(3.04 to 5.52)	0.2(-0.03 to 0.53)
Brunei Darussalam	Liver cancer due to NASH	0(0 to 1)	2(1 to 3)	3.42(2.35 to 4.83)	0.52(0.33 to 0.78)	0.69(0.46 to 1.01)	0.33(0.03 to 0.73)
Brunei Darussalam	Liver cancer due to other causes	0(0 to 1)	2(1 to 2)	2.82(1.94 to 4)	0.38(0.25 to 0.57)	0.49(0.33 to 0.71)	0.3(0.01 to 0.67)
Bulgaria	Liver cancer	782(732 to 836)	614(489 to 758)	-0.22(-0.38 to -0.01)	6.19(5.81 to 6.61)	4.5(3.58 to 5.58)	-0.27(-0.43 to -0.09)
Bulgaria	Liver cancer due to alcohol use	324(245 to 401)	282(204 to 380)	-0.13(-0.33 to 0.12)	2.49(1.92 to 3.08)	2.02(1.46 to 2.75)	-0.19(-0.37 to 0.04)
Bulgaria	Liver cancer due to hepatitis B	198(140 to 270)	125(83 to 188)	-0.37(-0.51 to -0.19)	1.6(1.17 to 2.13)	1.02(0.68 to 1.52)	-0.36(-0.51 to -0.19)
Bulgaria	Liver cancer due to hepatitis C	180(121 to 246)	142(93 to 209)	-0.21(-0.38 to 0)	1.43(1 to 1.92)	0.97(0.64 to 1.41)	-0.32(-0.46 to -0.15)
Bulgaria	Liver cancer due to NASH	54(37 to 77)	46(31 to 68)	-0.14(-0.34 to 0.11)	0.43(0.3 to 0.6)	0.33(0.22 to 0.48)	-0.25(-0.42 to -0.04)

Bulgaria	Liver cancer due to other causes	26(18 to 37)	18(12 to 27)	-0.3(-0.47 to -0.08)	0.23(0.17 to 0.31)	0.16(0.11 to 0.23)	-0.28(-0.45 to -0.06)
Burkina Faso	Liver cancer	143(112 to 179)	233(174 to 302)	0.63(0.19 to 1.18)	2.97(2.38 to 3.62)	2.18(1.66 to 2.75)	-0.26(-0.47 to -0.02)
Burkina Faso	Liver cancer due to alcohol use	19(12 to 28)	32(20 to 47)	0.72(0.17 to 1.45)	0.44(0.28 to 0.64)	0.37(0.23 to 0.53)	-0.17(-0.43 to 0.17)
Burkina Faso	Liver cancer due to hepatitis B	59(43 to 78)	92(64 to 125)	0.54(0.08 to 1.15)	1.25(0.89 to 1.66)	0.84(0.59 to 1.17)	-0.33(-0.53 to -0.08)
Burkina Faso	Liver cancer due to hepatitis C	33(22 to 46)	48(32 to 66)	0.44(0.04 to 0.98)	0.87(0.59 to 1.17)	0.6(0.39 to 0.82)	-0.31(-0.49 to -0.07)
Burkina Faso	Liver cancer due to NASH	8(6 to 12)	17(11 to 25)	1.09(0.51 to 1.92)	0.2(0.14 to 0.29)	0.19(0.13 to 0.29)	-0.04(-0.3 to 0.31)
Burkina Faso	Liver cancer due to other causes	23(13 to 38)	44(25 to 72)	0.89(0.07 to 2.2)	0.21(0.14 to 0.3)	0.18(0.12 to 0.27)	-0.12(-0.41 to 0.26)
Burundi	Liver cancer	94(68 to 124)	141(97 to 221)	0.51(-0.05 to 1.27)	3.69(2.71 to 4.83)	2.89(1.98 to 4.53)	-0.22(-0.49 to 0.16)
Burundi	Liver cancer due to alcohol use	28(18 to 41)	37(21 to 67)	0.33(-0.19 to 1.05)	1.2(0.77 to 1.76)	0.83(0.48 to 1.47)	-0.31(-0.56 to 0.06)
Burundi	Liver cancer due to hepatitis B	27(18 to 38)	44(26 to 74)	0.62(-0.01 to 1.59)	1(0.66 to 1.44)	0.78(0.45 to 1.3)	-0.23(-0.52 to 0.2)
Burundi	Liver cancer due to hepatitis C	17(10 to 27)	28(16 to 43)	0.59(0.04 to 1.42)	0.81(0.5 to 1.22)	0.71(0.44 to 1.09)	-0.13(-0.41 to 0.29)
Burundi	Liver cancer due to NASH	9(6 to 14)	15(9 to 24)	0.64(0.03 to 1.47)	0.39(0.24 to 0.59)	0.34(0.21 to 0.55)	-0.13(-0.43 to 0.31)

Burundi	Liver cancer due to other causes	12(7 to 20)	18(12 to 27)	0.46(-0.15 to 1.4)	0.29(0.19 to 0.44)	0.23(0.15 to 0.35)	-0.21(-0.51 to 0.2)
C??te d'Ivoire	Liver cancer	331(247 to 426)	534(384 to 738)	0.61(0.15 to 1.2)	7.46(5.69 to 9.39)	4.73(3.5 to 6.43)	-0.37(-0.53 to -0.15)
C??te d'Ivoire	Liver cancer due to alcohol use	57(34 to 87)	103(62 to 160)	0.79(0.26 to 1.54)	1.44(0.88 to 2.21)	1(0.62 to 1.55)	-0.31(-0.5 to -0.04)
C??te d'Ivoire	Liver cancer due to hepatitis B	190(133 to 254)	279(186 to 399)	0.47(0.01 to 1.08)	3.93(2.82 to 5.26)	2.21(1.49 to 3.19)	-0.44(-0.6 to -0.23)
C??te d'Ivoire	Liver cancer due to hepatitis C	41(25 to 61)	74(44 to 111)	0.79(0.3 to 1.39)	1.23(0.8 to 1.8)	0.84(0.53 to 1.26)	-0.32(-0.49 to -0.1)
C??te d'Ivoire	Liver cancer due to NASH	20(13 to 30)	44(28 to 67)	1.17(0.52 to 1.99)	0.53(0.34 to 0.79)	0.44(0.29 to 0.69)	-0.16(-0.39 to 0.11)
C??te d'Ivoire	Liver cancer due to other causes	23(15 to 33)	35(22 to 50)	0.52(0.03 to 1.16)	0.33(0.21 to 0.47)	0.23(0.14 to 0.35)	-0.3(-0.5 to -0.04)
Cabo Verde	Liver cancer	3(2 to 3)	50(41 to 61)	18.41(14.39 to 23.6)	1.09(0.94 to 1.27)	11.56(9.52 to 13.94)	9.58(7.4 to 12.48)
Cabo Verde	Liver cancer due to alcohol use	0(0 to 1)	9(6 to 13)	21.25(15.99 to 28.24)	0.18(0.12 to 0.26)	2.23(1.5 to 3.07)	11.38(8.63 to 15.02)
Cabo Verde	Liver cancer due to hepatitis B	1(1 to 2)	24(18 to 31)	19.41(14.79 to 25.55)	0.52(0.4 to 0.65)	5.27(3.95 to 6.83)	9.2(6.99 to 12.11)
Cabo Verde	Liver cancer due to hepatitis C	1(0 to 1)	9(6 to 13)	14.77(11.11 to 20.32)	0.24(0.16 to 0.33)	2.22(1.46 to 3.17)	8.35(6.13 to 11.63)
Cabo Verde	Liver cancer due to NASH	0(0 to 0)	5(4 to 8)	21.91(16.19 to 30.2)	0.1(0.06 to 0.14)	1.27(0.87 to 1.87)	12.31(8.98 to 17.09)
Cabo Verde	Liver cancer due to other causes	0(0 to 0)	3(2 to 4)	12.49(8.97 to 17.39)	0.06(0.05 to 0.09)	0.57(0.39 to	8.03(5.82 to 11.24)

						0.82)	
Cambodia	Liver cancer	622(486 to 805)	1144(887 to 1413)	0.84(0.24 to 1.63)	10.86(8.82 to 13.11)	9.2(7.26 to 11.26)	-0.15(-0.38 to 0.19)
Cambodia	Liver cancer due to alcohol use	54(34 to 80)	182(115 to 264)	2.4(1.43 to 3.74)	1.18(0.75 to 1.79)	1.52(0.97 to 2.19)	0.29(-0.07 to 0.8)
Cambodia	Liver cancer due to hepatitis B	241(181 to 316)	410(291 to 573)	0.7(0.17 to 1.37)	4(2.95 to 5.38)	2.99(2.11 to 4.16)	-0.25(-0.47 to 0.02)
Cambodia	Liver cancer due to hepatitis C	181(125 to 243)	392(274 to 525)	1.17(0.54 to 2.15)	4.27(3.04 to 5.55)	3.54(2.54 to 4.63)	-0.17(-0.4 to 0.19)
Cambodia	Liver cancer due to NASH	31(21 to 45)	74(49 to 109)	1.4(0.64 to 2.46)	0.62(0.41 to 0.88)	0.62(0.41 to 0.91)	0.01(-0.28 to 0.45)
Cambodia	Liver cancer due to other causes	115(49 to 247)	86(47 to 153)	-0.26(-0.71 to 0.7)	0.79(0.43 to 1.48)	0.54(0.31 to 0.92)	-0.32(-0.69 to 0.32)
Cameroon	Liver cancer	30(21 to 45)	85(60 to 116)	1.82(0.76 to 3.23)	0.65(0.45 to 0.97)	0.65(0.47 to 0.87)	0.01(-0.35 to 0.48)
Cameroon	Liver cancer due to alcohol use	5(3 to 9)	16(10 to 25)	2.1(0.84 to 3.73)	0.12(0.07 to 0.21)	0.14(0.09 to 0.21)	0.14(-0.3 to 0.73)
Cameroon	Liver cancer due to hepatitis B	16(10 to 25)	43(29 to 62)	1.73(0.62 to 3.21)	0.32(0.21 to 0.5)	0.3(0.2 to 0.43)	-0.05(-0.42 to 0.44)
Cameroon	Liver cancer due to hepatitis C	5(3 to 7)	11(7 to 17)	1.47(0.64 to 2.56)	0.12(0.07 to 0.18)	0.11(0.07 to 0.16)	-0.07(-0.37 to 0.31)
Cameroon	Liver cancer due to NASH	2(1 to 4)	8(5 to 12)	2.13(1.03 to 3.61)	0.06(0.03 to 0.09)	0.07(0.04 to 0.1)	0.15(-0.25 to 0.67)
Cameroon	Liver cancer due to other causes	2(1 to 3)	7(4 to 10)	2.26(1.14 to 3.68)	0.03(0.02 to 0.05)	0.03(0.02 to 0.05)	0.1(-0.29 to 0.58)

Canada	Liver cancer	657(628 to 682)	3108(2397 to 3997)	3.73(2.65 to 5.12)	2.05(1.96 to 2.13)	4.75(3.62 to 6.11)	1.31(0.77 to 1.99)
Canada	Liver cancer due to alcohol use	370(318 to 420)	1742(1224 to 2341)	3.71(2.57 to 5.11)	1.14(0.98 to 1.3)	2.64(1.85 to 3.53)	1.31(0.74 to 2)
Canada	Liver cancer due to hepatitis B	55(38 to 79)	214(131 to 333)	2.88(1.93 to 4.15)	0.17(0.12 to 0.25)	0.36(0.23 to 0.55)	1.05(0.54 to 1.69)
Canada	Liver cancer due to hepatitis C	116(80 to 157)	578(361 to 852)	4(2.83 to 5.43)	0.36(0.25 to 0.48)	0.83(0.52 to 1.22)	1.33(0.79 to 2)
Canada	Liver cancer due to NASH	67(48 to 93)	394(258 to 576)	4.86(3.42 to 6.6)	0.21(0.15 to 0.28)	0.58(0.39 to 0.83)	1.8(1.13 to 2.62)
Canada	Liver cancer due to other causes	49(38 to 63)	181(118 to 266)	2.71(1.78 to 3.93)	0.17(0.13 to 0.22)	0.33(0.23 to 0.47)	0.97(0.52 to 1.58)
Central African Republic	Liver cancer	47(32 to 65)	74(46 to 111)	0.59(0.17 to 1.15)	3.55(2.5 to 4.86)	3.18(2.08 to 4.66)	-0.1(-0.33 to 0.2)
Central African Republic	Liver cancer due to alcohol use	7(4 to 11)	11(6 to 19)	0.6(0.15 to 1.29)	0.57(0.33 to 0.89)	0.5(0.28 to 0.87)	-0.11(-0.36 to 0.25)
Central African Republic	Liver cancer due to hepatitis B	15(9 to 22)	22(13 to 37)	0.54(0.09 to 1.16)	0.99(0.61 to 1.48)	0.81(0.46 to 1.31)	-0.19(-0.41 to 0.11)
Central African Republic	Liver cancer due to hepatitis C	17(10 to 25)	28(16 to 43)	0.67(0.2 to 1.3)	1.55(1.01 to 2.25)	1.46(0.9 to 2.17)	-0.06(-0.3 to 0.26)
Central African Republic	Liver cancer due to NASH	3(2 to 4)	5(3 to 8)	0.82(0.31 to 1.55)	0.22(0.13 to 0.34)	0.22(0.13 to 0.36)	0.01(-0.27 to 0.38)
Central African Republic	Liver cancer due to other causes	6(3 to 10)	8(5 to 13)	0.36(-0.13 to 1.13)	0.22(0.13 to 0.33)	0.18(0.11 to 0.3)	-0.15(-0.41 to 0.2)

Chad	Liver cancer	182(141 to 234)	317(237 to 412)	0.74(0.32 to 1.33)	6.16(4.75 to 7.91)	5.31(4.05 to 6.84)	-0.14(-0.34 to 0.14)
Chad	Liver cancer due to alcohol use	26(15 to 40)	46(28 to 71)	0.79(0.29 to 1.48)	0.91(0.54 to 1.43)	0.85(0.52 to 1.3)	-0.06(-0.31 to 0.29)
Chad	Liver cancer due to hepatitis B	97(68 to 130)	169(119 to 229)	0.75(0.29 to 1.38)	3.2(2.26 to 4.35)	2.66(1.87 to 3.63)	-0.17(-0.38 to 0.11)
Chad	Liver cancer due to hepatitis C	34(21 to 49)	53(32 to 78)	0.55(0.19 to 1.08)	1.28(0.81 to 1.81)	1.07(0.68 to 1.54)	-0.16(-0.35 to 0.11)
Chad	Liver cancer due to NASH	13(8 to 19)	24(15 to 36)	0.89(0.4 to 1.52)	0.45(0.29 to 0.66)	0.44(0.28 to 0.66)	-0.02(-0.26 to 0.29)
Chad	Liver cancer due to other causes	13(9 to 18)	25(17 to 35)	0.93(0.4 to 1.7)	0.31(0.21 to 0.44)	0.28(0.18 to 0.41)	-0.1(-0.32 to 0.21)
Chile	Liver cancer	215(201 to 231)	714(554 to 899)	2.32(1.53 to 3.19)	2.14(1.99 to 2.3)	2.98(2.31 to 3.75)	0.4(0.07 to 0.77)
Chile	Liver cancer due to alcohol use	67(49 to 88)	226(148 to 319)	2.36(1.52 to 3.41)	0.67(0.48 to 0.87)	0.93(0.61 to 1.32)	0.4(0.05 to 0.83)
Chile	Liver cancer due to hepatitis B	45(32 to 63)	118(75 to 182)	1.64(0.97 to 2.46)	0.43(0.3 to 0.59)	0.5(0.32 to 0.76)	0.17(-0.12 to 0.52)
Chile	Liver cancer due to hepatitis C	70(51 to 89)	252(168 to 352)	2.62(1.8 to 3.62)	0.72(0.52 to 0.92)	1.04(0.69 to 1.45)	0.44(0.13 to 0.84)
Chile	Liver cancer due to NASH	17(12 to 23)	74(48 to 111)	3.46(2.41 to 4.89)	0.17(0.12 to 0.24)	0.31(0.2 to 0.46)	0.82(0.4 to 1.39)
Chile	Liver cancer due to other causes	17(13 to 22)	43(28 to 64)	1.58(0.94 to 2.36)	0.15(0.11 to 0.2)	0.2(0.14 to 0.29)	0.32(0.01 to 0.69)

China	Liver cancer	236825(199322 to 280116)	210462(174832 to 251195)	-0.11(-0.32 to 0.15)	25.71(21.73 to 30.35)	10.46(8.74 to 12.42)	-0.59(-0.68 to -0.48)
China	Liver cancer due to alcohol use	17554(12726 to 23390)	19188(14315 to 25625)	0.09(-0.15 to 0.41)	1.95(1.44 to 2.59)	0.92(0.69 to 1.22)	-0.53(-0.63 to -0.39)
China	Liver cancer due to hepatitis B	158410(129499 to 191318)	135028(108924 to 164320)	-0.15(-0.36 to 0.13)	16.48(13.52 to 19.86)	6.63(5.36 to 8.07)	-0.6(-0.7 to -0.47)
China	Liver cancer due to hepatitis C	35447(28972 to 43242)	34036(27796 to 40829)	-0.04(-0.25 to 0.21)	4.53(3.76 to 5.48)	1.75(1.44 to 2.09)	-0.61(-0.69 to -0.51)
China	Liver cancer due to NASH	8809(6898 to 10830)	10034(7813 to 12509)	0.14(-0.1 to 0.44)	1.01(0.8 to 1.24)	0.51(0.4 to 0.63)	-0.5(-0.61 to -0.38)
China	Liver cancer due to other causes	16605(13560 to 20527)	12176(9859 to 15011)	-0.27(-0.42 to -0.08)	1.72(1.41 to 2.14)	0.64(0.53 to 0.78)	-0.63(-0.7 to -0.53)
Colombia	Liver cancer	509(483 to 535)	1318(1002 to 1690)	1.59(0.98 to 2.33)	2.84(2.68 to 2.98)	2.51(1.91 to 3.22)	-0.11(-0.32 to 0.14)
Colombia	Liver cancer due to alcohol use	143(104 to 184)	417(277 to 603)	1.91(1.15 to 2.82)	0.82(0.6 to 1.06)	0.8(0.53 to 1.15)	-0.03(-0.28 to 0.27)
Colombia	Liver cancer due to hepatitis B	115(86 to 152)	232(146 to 349)	1.03(0.5 to 1.68)	0.57(0.42 to 0.78)	0.44(0.28 to 0.67)	-0.23(-0.42 to 0.01)
Colombia	Liver cancer due to hepatitis C	177(134 to 220)	489(337 to 684)	1.76(1.12 to 2.58)	1.09(0.83 to 1.34)	0.92(0.63 to 1.29)	-0.15(-0.35 to 0.1)
Colombia	Liver cancer due to NASH	30(21 to 43)	102(65 to 155)	2.39(1.52 to 3.57)	0.17(0.12 to 0.25)	0.19(0.12 to 0.3)	0.11(-0.17 to 0.49)
Colombia	Liver cancer due to other causes	44(34 to 55)	77(52 to 109)	0.75(0.29 to 1.32)	0.18(0.14 to 0.23)	0.16(0.11 to 0.22)	-0.11(-0.33 to 0.16)

Comoros	Liver cancer	7(4 to 12)	14(9 to 21)	0.91(0.3 to 2.67)	3.09(1.69 to 5.16)	2.79(1.94 to 4.33)	-0.09(-0.37 to 0.6)
Comoros	Liver cancer due to alcohol use	1(1 to 3)	3(1 to 5)	0.92(0.3 to 2.46)	0.63(0.3 to 1.31)	0.56(0.31 to 1.09)	-0.11(-0.4 to 0.53)
Comoros	Liver cancer due to hepatitis B	2(1 to 5)	4(2 to 8)	0.81(0.17 to 3.03)	1(0.45 to 1.85)	0.82(0.47 to 1.42)	-0.17(-0.46 to 0.7)
Comoros	Liver cancer due to hepatitis C	2(1 to 3)	3(2 to 5)	1.1(0.46 to 2.43)	0.79(0.45 to 1.27)	0.75(0.48 to 1.15)	-0.05(-0.32 to 0.47)
Comoros	Liver cancer due to NASH	1(0 to 2)	2(1 to 3)	1.27(0.55 to 3.05)	0.4(0.22 to 0.68)	0.42(0.25 to 0.66)	0.05(-0.27 to 0.73)
Comoros	Liver cancer due to other causes	1(0 to 1)	1(1 to 2)	0.49(-0.08 to 2.02)	0.27(0.13 to 0.43)	0.24(0.15 to 0.36)	-0.11(-0.41 to 0.69)
Congo	Liver cancer	46(34 to 63)	77(55 to 107)	0.68(0.22 to 1.37)	3.97(2.99 to 5.38)	2.89(2.11 to 3.94)	-0.27(-0.46 to 0)
Congo	Liver cancer due to alcohol use	7(4 to 11)	13(7 to 20)	0.9(0.34 to 1.89)	0.61(0.35 to 0.95)	0.48(0.3 to 0.74)	-0.2(-0.43 to 0.17)
Congo	Liver cancer due to hepatitis B	13(8 to 20)	21(13 to 32)	0.59(0.07 to 1.36)	1(0.63 to 1.53)	0.65(0.41 to 0.99)	-0.35(-0.55 to -0.05)
Congo	Liver cancer due to hepatitis C	19(12 to 27)	32(21 to 47)	0.71(0.24 to 1.37)	1.88(1.32 to 2.62)	1.38(0.95 to 1.97)	-0.27(-0.45 to -0.02)
Congo	Liver cancer due to NASH	3(2 to 4)	6(4 to 9)	1.1(0.44 to 2.01)	0.26(0.17 to 0.4)	0.23(0.14 to 0.36)	-0.11(-0.37 to 0.22)
Congo	Liver cancer due to other causes	5(3 to 7)	6(4 to 9)	0.23(-0.23 to 0.92)	0.22(0.15 to 0.31)	0.15(0.09 to 0.22)	-0.33(-0.54 to -0.05)

Cook Islands	Liver cancer	2(1 to 2)	3(2 to 3)	0.6(0.22 to 1.06)	13.25(11.01 to 15.61)	11.37(9.15 to 13.93)	-0.14(-0.34 to 0.1)
Cook Islands	Liver cancer due to alcohol use	0(0 to 0)	0(0 to 1)	1.29(0.69 to 2.06)	1.56(0.96 to 2.25)	1.84(1.18 to 2.68)	0.18(-0.11 to 0.57)
Cook Islands	Liver cancer due to hepatitis B	1(1 to 1)	1(1 to 2)	0.39(0.02 to 0.89)	6.94(5.22 to 9.07)	5.5(4.07 to 7.32)	-0.21(-0.42 to 0.08)
Cook Islands	Liver cancer due to hepatitis C	0(0 to 0)	1(0 to 1)	0.62(0.27 to 1.09)	2.96(1.97 to 4.05)	2.27(1.52 to 3.21)	-0.23(-0.39 to -0.02)
Cook Islands	Liver cancer due to NASH	0(0 to 0)	0(0 to 0)	1.16(0.63 to 1.83)	1.2(0.83 to 1.7)	1.32(0.87 to 1.85)	0.1(-0.15 to 0.44)
Cook Islands	Liver cancer due to other causes	0(0 to 0)	0(0 to 0)	0.22(-0.11 to 0.64)	0.58(0.39 to 0.83)	0.43(0.28 to 0.63)	-0.26(-0.45 to -0.01)
Costa Rica	Liver cancer	96(89 to 102)	261(202 to 331)	1.73(1.09 to 2.51)	5.42(5.02 to 5.8)	5.14(3.97 to 6.51)	-0.05(-0.27 to 0.22)
Costa Rica	Liver cancer due to alcohol use	32(24 to 40)	91(61 to 129)	1.86(1.17 to 2.74)	1.83(1.34 to 2.34)	1.78(1.2 to 2.53)	-0.03(-0.26 to 0.27)
Costa Rica	Liver cancer due to hepatitis B	17(13 to 23)	40(26 to 61)	1.36(0.78 to 2.08)	0.89(0.64 to 1.23)	0.78(0.5 to 1.18)	-0.13(-0.34 to 0.14)
Costa Rica	Liver cancer due to hepatitis C	34(25 to 43)	93(63 to 129)	1.76(1.14 to 2.56)	2.01(1.51 to 2.56)	1.84(1.24 to 2.54)	-0.08(-0.29 to 0.18)
Costa Rica	Liver cancer due to NASH	7(5 to 9)	23(15 to 35)	2.48(1.6 to 3.57)	0.38(0.26 to 0.53)	0.46(0.3 to 0.69)	0.21(-0.09 to 0.59)
Costa Rica	Liver cancer due to other causes	6(5 to 8)	14(9 to 20)	1.15(0.6 to 1.84)	0.3(0.22 to 0.41)	0.28(0.19 to 0.4)	-0.08(-0.3 to 0.2)

Croatia	Liver cancer	200(185 to 216)	321(252 to 404)	0.6(0.24 to 1.07)	3.13(2.9 to 3.37)	3.73(2.92 to 4.75)	0.19(-0.08 to 0.55)
Croatia	Liver cancer due to alcohol use	87(67 to 107)	152(107 to 203)	0.74(0.33 to 1.28)	1.34(1.05 to 1.63)	1.75(1.22 to 2.35)	0.3(-0.01 to 0.7)
Croatia	Liver cancer due to hepatitis B	48(34 to 64)	65(42 to 97)	0.36(0.01 to 0.79)	0.74(0.53 to 0.99)	0.82(0.54 to 1.22)	0.11(-0.16 to 0.47)
Croatia	Liver cancer due to hepatitis C	40(27 to 55)	61(39 to 89)	0.54(0.2 to 0.99)	0.63(0.43 to 0.87)	0.67(0.43 to 0.96)	0.05(-0.17 to 0.34)
Croatia	Liver cancer due to NASH	17(12 to 23)	31(20 to 46)	0.85(0.39 to 1.45)	0.27(0.18 to 0.37)	0.34(0.22 to 0.51)	0.28(-0.03 to 0.69)
Croatia	Liver cancer due to other causes	9(6 to 12)	11(7 to 17)	0.31(0 to 0.69)	0.15(0.11 to 0.2)	0.16(0.11 to 0.22)	0.07(-0.17 to 0.36)
Cuba	Liver cancer	659(624 to 690)	442(355 to 548)	-0.33(-0.46 to -0.18)	6.35(6.01 to 6.65)	2.34(1.89 to 2.9)	-0.63(-0.71 to -0.55)
Cuba	Liver cancer due to alcohol use	214(159 to 271)	165(113 to 226)	-0.23(-0.4 to -0.03)	2.06(1.53 to 2.61)	0.87(0.59 to 1.19)	-0.58(-0.67 to -0.47)
Cuba	Liver cancer due to hepatitis B	186(140 to 245)	107(73 to 155)	-0.43(-0.55 to -0.28)	1.8(1.34 to 2.35)	0.58(0.4 to 0.84)	-0.68(-0.75 to -0.6)
Cuba	Liver cancer due to hepatitis C	158(111 to 210)	101(66 to 144)	-0.36(-0.49 to -0.23)	1.53(1.08 to 2.03)	0.52(0.34 to 0.75)	-0.66(-0.73 to -0.59)
Cuba	Liver cancer due to NASH	57(40 to 78)	45(29 to 68)	-0.21(-0.38 to 0.02)	0.54(0.39 to 0.75)	0.23(0.15 to 0.36)	-0.57(-0.66 to -0.44)
Cuba	Liver cancer due to other causes	44(32 to 59)	23(15 to 34)	-0.47(-0.59 to -0.32)	0.42(0.31 to 0.57)	0.14(0.1 to 0.2)	-0.67(-0.73 to -0.58)
Cyprus	Liver cancer	25(21 to 29)	70(60 to 82)	1.8(1.24 to 2.6)	3.15(2.67 to 3.65)	3.69(3.16 to	0.17(-0.05 to 0.49)

						4.32)	
Cyprus	Liver cancer due to alcohol use	9(6 to 13)	27(19 to 35)	1.87(1.18 to 2.89)	1.11(0.78 to 1.47)	1.36(0.97 to 1.81)	0.22(-0.06 to 0.61)
Cyprus	Liver cancer due to hepatitis B	4(2 to 6)	9(6 to 13)	1.31(0.8 to 2)	0.46(0.31 to 0.67)	0.47(0.32 to 0.67)	0.01(-0.2 to 0.31)
Cyprus	Liver cancer due to hepatitis C	9(6 to 12)	27(19 to 34)	1.91(1.3 to 2.85)	1.22(0.88 to 1.6)	1.41(1.04 to 1.82)	0.16(-0.07 to 0.49)
Cyprus	Liver cancer due to NASH	1(1 to 2)	4(3 to 7)	2.26(1.51 to 3.34)	0.17(0.12 to 0.25)	0.23(0.15 to 0.34)	0.33(0.05 to 0.71)
Cyprus	Liver cancer due to other causes	1(1 to 2)	4(2 to 5)	1.45(0.9 to 2.1)	0.19(0.13 to 0.25)	0.22(0.16 to 0.3)	0.18(-0.07 to 0.5)
Czechia	Liver cancer	596(566 to 624)	615(504 to 755)	0.03(-0.17 to 0.29)	4.32(4.1 to 4.52)	2.96(2.42 to 3.65)	-0.31(-0.45 to -0.14)
Czechia	Liver cancer due to alcohol use	275(220 to 328)	302(223 to 395)	0.1(-0.14 to 0.38)	1.97(1.57 to 2.34)	1.43(1.05 to 1.87)	-0.28(-0.43 to -0.09)
Czechia	Liver cancer due to hepatitis B	131(93 to 181)	108(71 to 162)	-0.18(-0.37 to 0.05)	0.97(0.7 to 1.34)	0.56(0.38 to 0.82)	-0.42(-0.55 to -0.26)
Czechia	Liver cancer due to hepatitis C	133(91 to 178)	141(93 to 205)	0.06(-0.16 to 0.33)	0.95(0.66 to 1.27)	0.65(0.42 to 0.94)	-0.32(-0.45 to -0.15)
Czechia	Liver cancer due to NASH	38(26 to 54)	46(29 to 68)	0.21(-0.04 to 0.54)	0.27(0.19 to 0.38)	0.21(0.14 to 0.32)	-0.21(-0.37 to 0.01)
Czechia	Liver cancer due to other causes	19(14 to 27)	18(12 to 26)	-0.09(-0.28 to 0.17)	0.16(0.12 to 0.21)	0.11(0.08 to 0.15)	-0.3(-0.44 to -0.12)

Democratic People's Republic of Korea	Liver cancer	2589(1965 to 3410)	3353(2492 to 4402)	0.3(-0.12 to 0.82)	14.62(11.35 to 18.97)	10.23(7.66 to 13.36)	-0.3(-0.51 to -0.04)
Democratic People's Republic of Korea	Liver cancer due to alcohol use	183(108 to 298)	271(155 to 435)	0.48(0 to 1.13)	1.05(0.65 to 1.66)	0.82(0.48 to 1.3)	-0.22(-0.47 to 0.09)
Democratic People's Republic of Korea	Liver cancer due to hepatitis B	1646(1185 to 2253)	2014(1394 to 2795)	0.22(-0.21 to 0.82)	8.76(6.38 to 11.82)	6.05(4.19 to 8.45)	-0.31(-0.54 to 0)
Democratic People's Republic of Korea	Liver cancer due to hepatitis C	477(299 to 690)	717(461 to 1019)	0.5(0.1 to 1.03)	3.21(2.07 to 4.53)	2.27(1.48 to 3.21)	-0.29(-0.48 to -0.06)
Democratic People's Republic of Korea	Liver cancer due to NASH	110(71 to 170)	159(98 to 245)	0.44(-0.01 to 1)	0.68(0.43 to 1.03)	0.49(0.31 to 0.76)	-0.28(-0.49 to -0.02)
Democratic People's Republic of Korea	Liver cancer due to other causes	173(110 to 252)	193(119 to 290)	0.12(-0.24 to 0.59)	0.92(0.6 to 1.33)	0.6(0.38 to 0.88)	-0.34(-0.54 to -0.07)
Democratic Republic of the Congo	Liver cancer	456(367 to 564)	870(659 to 1142)	0.91(0.35 to 1.66)	2.47(2.02 to 2.96)	2.14(1.65 to 2.79)	-0.13(-0.37 to 0.21)

Democratic Republic of the Congo	Liver cancer due to alcohol use	52(33 to 76)	103(61 to 157)	0.99(0.39 to 1.92)	0.33(0.21 to 0.47)	0.28(0.17 to 0.42)	-0.15(-0.4 to 0.22)
Democratic Republic of the Congo	Liver cancer due to hepatitis B	122(85 to 170)	239(156 to 355)	0.96(0.34 to 1.87)	0.61(0.42 to 0.87)	0.5(0.32 to 0.76)	-0.19(-0.43 to 0.16)
Democratic Republic of the Congo	Liver cancer due to hepatitis C	171(124 to 226)	352(245 to 484)	1.05(0.44 to 1.95)	1.19(0.9 to 1.52)	1.07(0.76 to 1.43)	-0.11(-0.34 to 0.23)
Democratic Republic of the Congo	Liver cancer due to NASH	24(16 to 36)	56(37 to 86)	1.32(0.6 to 2.36)	0.15(0.1 to 0.22)	0.15(0.1 to 0.23)	-0.01(-0.28 to 0.4)
Democratic Republic of the Congo	Liver cancer due to other causes	86(50 to 149)	120(64 to 217)	0.39(-0.3 to 1.59)	0.18(0.12 to 0.26)	0.15(0.09 to 0.23)	-0.19(-0.5 to 0.25)
Denmark	Liver cancer	191(180 to 201)	434(341 to 553)	1.27(0.79 to 1.94)	2.41(2.28 to 2.54)	3.98(3.11 to 5.1)	0.65(0.29 to 1.13)
Denmark	Liver cancer due to alcohol use	77(58 to 96)	175(118 to 243)	1.26(0.72 to 1.98)	0.97(0.74 to 1.2)	1.58(1.06 to 2.23)	0.63(0.25 to 1.17)
Denmark	Liver cancer due to hepatitis B	25(17 to 36)	59(36 to 91)	1.36(0.8 to 2.07)	0.34(0.24 to 0.48)	0.61(0.38 to 0.93)	0.77(0.34 to 1.31)
Denmark	Liver cancer due to hepatitis C	68(50 to 88)	152(104 to 215)	1.25(0.75 to 1.85)	0.82(0.6 to 1.05)	1.32(0.9 to 1.86)	0.62(0.26 to 1.06)
Denmark	Liver cancer due to NASH	11(7 to 16)	27(17 to 41)	1.49(0.89 to 2.27)	0.13(0.09 to 0.19)	0.24(0.15 to 0.36)	0.82(0.38 to 1.4)
Denmark	Liver cancer due to other causes	10(7 to 14)	21(13 to 31)	1.09(0.56 to 1.73)	0.16(0.12 to 0.2)	0.24(0.16 to 0.33)	0.53(0.16 to 0.96)

Djibouti	Liver cancer	5(3 to 9)	20(12 to 34)	2.91(1.68 to 4.68)	3.15(2.06 to 5.44)	3.22(2.07 to 5.23)	0.02(-0.27 to 0.42)
Djibouti	Liver cancer due to alcohol use	1(0 to 2)	5(2 to 9)	3.39(1.99 to 5.49)	0.74(0.37 to 1.58)	0.78(0.42 to 1.49)	0.06(-0.26 to 0.51)
Djibouti	Liver cancer due to hepatitis B	2(1 to 4)	7(4 to 12)	2.76(1.48 to 4.88)	0.99(0.54 to 1.93)	0.96(0.53 to 1.69)	-0.04(-0.35 to 0.4)
Djibouti	Liver cancer due to hepatitis C	1(1 to 2)	4(2 to 7)	3.31(2.02 to 5.09)	0.79(0.48 to 1.3)	0.8(0.49 to 1.29)	0.02(-0.26 to 0.37)
Djibouti	Liver cancer due to NASH	0(0 to 1)	2(1 to 4)	3.76(2.24 to 6)	0.37(0.2 to 0.63)	0.43(0.24 to 0.74)	0.17(-0.16 to 0.62)
Djibouti	Liver cancer due to other causes	1(0 to 1)	2(1 to 3)	1.62(0.53 to 3.17)	0.26(0.16 to 0.42)	0.25(0.15 to 0.41)	-0.05(-0.36 to 0.38)
Dominica	Liver cancer	6(5 to 7)	3(2 to 4)	-0.54(-0.64 to -0.41)	8.59(7.5 to 9.72)	3.18(2.58 to 3.95)	-0.63(-0.71 to -0.53)
Dominica	Liver cancer due to alcohol use	2(1 to 3)	1(1 to 1)	-0.49(-0.61 to -0.34)	2.82(2.02 to 3.71)	1.15(0.79 to 1.59)	-0.59(-0.69 to -0.47)
Dominica	Liver cancer due to hepatitis B	2(1 to 2)	1(0 to 1)	-0.56(-0.66 to -0.42)	2.45(1.78 to 3.3)	0.83(0.56 to 1.18)	-0.66(-0.74 to -0.56)
Dominica	Liver cancer due to hepatitis C	2(1 to 2)	1(0 to 1)	-0.58(-0.67 to -0.47)	2.03(1.43 to 2.76)	0.69(0.46 to 0.99)	-0.66(-0.73 to -0.56)
Dominica	Liver cancer due to NASH	1(0 to 1)	0(0 to 0)	-0.48(-0.6 to -0.31)	0.71(0.49 to 0.99)	0.31(0.21 to 0.44)	-0.57(-0.67 to -0.44)
Dominica	Liver cancer due to other causes	0(0 to 1)	0(0 to 0)	-0.6(-0.69 to -0.47)	0.58(0.41 to 0.79)	0.2(0.14 to 0.28)	-0.65(-0.73 to -0.54)

Dominican Republic	Liver cancer	155(136 to 176)	435(294 to 660)	1.8(0.82 to 3.36)	3.91(3.38 to 4.45)	4.64(3.16 to 6.99)	0.19(-0.22 to 0.85)
Dominican Republic	Liver cancer due to alcohol use	47(34 to 62)	151(90 to 255)	2.2(1.01 to 4.13)	1.26(0.91 to 1.65)	1.63(0.97 to 2.76)	0.29(-0.18 to 1.09)
Dominican Republic	Liver cancer due to hepatitis B	51(38 to 66)	127(74 to 215)	1.51(0.56 to 3.12)	1.17(0.87 to 1.54)	1.3(0.77 to 2.18)	0.11(-0.3 to 0.83)
Dominican Republic	Liver cancer due to hepatitis C	30(21 to 41)	87(55 to 139)	1.9(0.91 to 3.28)	0.88(0.62 to 1.18)	0.98(0.62 to 1.55)	0.11(-0.26 to 0.62)
Dominican Republic	Liver cancer due to NASH	11(8 to 15)	38(23 to 59)	2.54(1.32 to 4.27)	0.28(0.2 to 0.4)	0.41(0.25 to 0.64)	0.46(-0.03 to 1.16)
Dominican Republic	Liver cancer due to other causes	17(13 to 21)	31(21 to 46)	0.87(0.27 to 1.7)	0.31(0.24 to 0.4)	0.32(0.21 to 0.48)	0.03(-0.29 to 0.46)
Ecuador	Liver cancer	155(139 to 172)	505(400 to 653)	2.26(1.49 to 3.22)	2.82(2.5 to 3.13)	3.4(2.72 to 4.37)	0.21(-0.07 to 0.57)
Ecuador	Liver cancer due to alcohol use	37(26 to 50)	149(99 to 216)	3.04(2.08 to 4.39)	0.72(0.5 to 0.98)	1.01(0.68 to 1.45)	0.41(0.08 to 0.87)
Ecuador	Liver cancer due to hepatitis B	77(62 to 93)	215(152 to 291)	1.81(1.11 to 2.7)	1.35(1.07 to 1.65)	1.42(1 to 1.92)	0.05(-0.2 to 0.38)
Ecuador	Liver cancer due to hepatitis C	11(7 to 16)	41(24 to 62)	2.64(1.68 to 3.76)	0.23(0.15 to 0.34)	0.29(0.18 to 0.44)	0.26(-0.06 to 0.66)
Ecuador	Liver cancer due to NASH	15(10 to 21)	61(40 to 89)	3.12(1.99 to 4.45)	0.29(0.2 to 0.41)	0.43(0.28 to 0.61)	0.47(0.08 to 0.96)
Ecuador	Liver cancer due to other causes	15(12 to 20)	39(26 to 55)	1.53(0.87 to 2.33)	0.23(0.17 to 0.31)	0.25(0.17 to 0.36)	0.11(-0.17 to 0.45)
Egypt	Liver cancer	5143(4090 to 6179)	13841(9844 to 19064)	1.69(0.78 to 3.25)	16.75(13.1 to 20.2)	20.92(15.09 to	0.25(-0.17 to 0.94)

						28.51)	
Egypt	Liver cancer due to alcohol use	382(225 to 598)	1219(662 to 2118)	2.19(1 to 4.14)	1.24(0.73 to 1.93)	1.79(0.99 to 3.07)	0.44(-0.08 to 1.33)
Egypt	Liver cancer due to hepatitis B	790(528 to 1155)	1880(1118 to 3028)	1.38(0.56 to 2.73)	2.29(1.52 to 3.39)	2.49(1.5 to 4.03)	0.09(-0.28 to 0.72)
Egypt	Liver cancer due to hepatitis C	3294(2484 to 4067)	8678(5840 to 12411)	1.63(0.72 to 3.16)	11.3(8.43 to 13.9)	13.64(9.44 to 19.19)	0.21(-0.2 to 0.89)
Egypt	Liver cancer due to NASH	324(212 to 485)	1271(764 to 2053)	2.92(1.55 to 5.29)	1.08(0.7 to 1.65)	1.94(1.18 to 3.07)	0.81(0.18 to 1.85)
Egypt	Liver cancer due to other causes	354(255 to 494)	794(500 to 1238)	1.25(0.47 to 2.34)	0.85(0.59 to 1.19)	1.06(0.66 to 1.67)	0.25(-0.16 to 0.84)
El Salvador	Liver cancer	103(95 to 111)	118(89 to 153)	0.14(-0.15 to 0.49)	3.37(3.1 to 3.64)	1.99(1.5 to 2.59)	-0.41(-0.56 to -0.23)
El Salvador	Liver cancer due to alcohol use	25(18 to 33)	37(24 to 53)	0.44(0.05 to 0.91)	0.86(0.62 to 1.13)	0.63(0.4 to 0.91)	-0.28(-0.47 to -0.04)
El Salvador	Liver cancer due to hepatitis B	21(15 to 27)	18(11 to 27)	-0.13(-0.38 to 0.18)	0.63(0.45 to 0.86)	0.31(0.19 to 0.47)	-0.51(-0.65 to -0.35)
El Salvador	Liver cancer due to hepatitis C	40(30 to 50)	45(30 to 63)	0.14(-0.16 to 0.51)	1.38(1.05 to 1.73)	0.75(0.5 to 1.06)	-0.45(-0.6 to -0.28)
El Salvador	Liver cancer due to NASH	7(5 to 11)	11(7 to 17)	0.44(0.04 to 0.92)	0.25(0.17 to 0.36)	0.18(0.11 to 0.28)	-0.27(-0.47 to -0.02)
El Salvador	Liver cancer due to other causes	10(8 to 12)	7(5 to 10)	-0.27(-0.47 to 0.01)	0.25(0.18 to 0.33)	0.12(0.08 to 0.17)	-0.52(-0.66 to -0.34)
Equatorial Guinea	Liver cancer	7(5 to 9)	16(9 to 24)	1.43(0.33 to 3.03)	3.03(2.27 to 3.96)	3.17(1.87 to	0.05(-0.43 to 0.69)

						4.63)	
Equatorial Guinea	Liver cancer due to alcohol use	1(0 to 1)	3(1 to 4)	2.22(0.64 to 4.52)	0.41(0.24 to 0.63)	0.57(0.29 to 0.95)	0.4(-0.28 to 1.41)
Equatorial Guinea	Liver cancer due to hepatitis B	2(1 to 3)	5(3 to 8)	1.54(0.34 to 3.32)	0.78(0.52 to 1.17)	0.75(0.4 to 1.22)	-0.04(-0.5 to 0.6)
Equatorial Guinea	Liver cancer due to hepatitis C	3(2 to 4)	6(3 to 9)	1.18(0.16 to 2.5)	1.47(1.02 to 2.03)	1.42(0.81 to 2.18)	-0.03(-0.48 to 0.52)
Equatorial Guinea	Liver cancer due to NASH	0(0 to 1)	1(1 to 2)	2.72(1.01 to 5.18)	0.19(0.11 to 0.29)	0.28(0.15 to 0.47)	0.53(-0.16 to 1.43)
Equatorial Guinea	Liver cancer due to other causes	1(0 to 1)	1(1 to 2)	0.66(-0.12 to 1.99)	0.18(0.12 to 0.26)	0.15(0.08 to 0.24)	-0.18(-0.53 to 0.35)
Eritrea	Liver cancer	35(24 to 54)	87(60 to 128)	1.46(0.71 to 2.55)	3.21(2.17 to 4.86)	3.1(2.17 to 4.41)	-0.03(-0.32 to 0.39)
Eritrea	Liver cancer due to alcohol use	7(4 to 13)	19(10 to 32)	1.57(0.76 to 2.8)	0.72(0.39 to 1.28)	0.7(0.39 to 1.17)	-0.03(-0.31 to 0.42)
Eritrea	Liver cancer due to hepatitis B	13(8 to 20)	29(17 to 45)	1.26(0.51 to 2.38)	1.01(0.62 to 1.59)	0.86(0.52 to 1.35)	-0.15(-0.41 to 0.24)
Eritrea	Liver cancer due to hepatitis C	7(4 to 12)	19(11 to 29)	1.73(0.83 to 3.26)	0.83(0.47 to 1.38)	0.87(0.54 to 1.28)	0.04(-0.29 to 0.58)
Eritrea	Liver cancer due to NASH	3(2 to 6)	10(6 to 16)	1.9(0.9 to 3.51)	0.37(0.21 to 0.63)	0.41(0.25 to 0.65)	0.1(-0.26 to 0.66)
Eritrea	Liver cancer due to other causes	5(3 to 8)	11(7 to 16)	1.13(0.34 to 2.4)	0.28(0.17 to 0.44)	0.27(0.17 to 0.4)	-0.02(-0.34 to 0.5)
Estonia	Liver cancer	42(39 to 45)	94(72 to 118)	1.26(0.74 to 1.82)	2.06(1.92 to 2.21)	3.71(2.84 to	0.8(0.39 to 1.27)

						4.71)	
Estonia	Liver cancer due to alcohol use	15(11 to 19)	42(30 to 56)	1.82(1.11 to 2.69)	0.73(0.54 to 0.91)	1.67(1.18 to 2.23)	1.3(0.71 to 2.02)
Estonia	Liver cancer due to hepatitis B	10(7 to 14)	17(11 to 25)	0.61(0.19 to 1.09)	0.52(0.37 to 0.7)	0.73(0.47 to 1.08)	0.42(0.05 to 0.85)
Estonia	Liver cancer due to hepatitis C	11(8 to 15)	24(16 to 34)	1.18(0.66 to 1.8)	0.54(0.39 to 0.72)	0.87(0.56 to 1.26)	0.62(0.22 to 1.04)
Estonia	Liver cancer due to NASH	3(2 to 4)	8(5 to 12)	1.65(0.96 to 2.53)	0.15(0.1 to 0.21)	0.29(0.19 to 0.44)	1(0.51 to 1.61)
Estonia	Liver cancer due to other causes	2(1 to 3)	3(2 to 4)	0.33(-0.11 to 0.92)	0.13(0.08 to 0.19)	0.14(0.1 to 0.21)	0.07(-0.31 to 0.7)
Eswatini	Liver cancer	18(11 to 35)	119(34 to 220)	5.68(0.15 to 15.4)	5.64(3.67 to 10.81)	18.43(5.61 to 32.97)	2.27(-0.4 to 6.71)
Eswatini	Liver cancer due to alcohol use	3(2 to 7)	27(6 to 56)	7.53(0.19 to 20.53)	1.05(0.53 to 2.5)	4.38(1.09 to 8.93)	3.2(-0.39 to 9.48)
Eswatini	Liver cancer due to hepatitis B	7(4 to 16)	54(13 to 105)	6.3(0 to 18.61)	2.08(1.14 to 4.44)	7.51(1.83 to 14.25)	2.61(-0.49 to 8.35)
Eswatini	Liver cancer due to hepatitis C	4(3 to 8)	22(7 to 41)	3.89(0.26 to 9.8)	1.69(1.03 to 2.9)	4.04(1.43 to 7.46)	1.4(-0.34 to 4.11)
Eswatini	Liver cancer due to NASH	2(1 to 3)	11(3 to 20)	5.59(0.43 to 14.37)	0.54(0.31 to 1.01)	1.75(0.57 to 3.25)	2.22(-0.24 to 6.26)
Eswatini	Liver cancer due to other causes	1(1 to 2)	5(2 to 11)	3.65(0.14 to 10.38)	0.29(0.17 to 0.51)	0.74(0.25 to 1.44)	1.57(-0.37 to 4.99)
Ethiopia	Liver cancer	642(446 to 926)	1155(930 to 1476)	0.8(0.07 to 1.81)	2.91(2.1 to 3.99)	2.73(2.21 to	-0.06(-0.41 to 0.41)

						3.47)	
Ethiopia	Liver cancer due to alcohol use	129(87 to 188)	247(183 to 331)	0.92(0.12 to 1.98)	0.65(0.44 to 0.91)	0.63(0.47 to 0.85)	-0.03(-0.42 to 0.48)
Ethiopia	Liver cancer due to hepatitis B	157(104 to 238)	277(207 to 370)	0.76(0 to 1.84)	0.66(0.44 to 0.96)	0.58(0.43 to 0.78)	-0.12(-0.48 to 0.36)
Ethiopia	Liver cancer due to hepatitis C	191(130 to 272)	384(302 to 492)	1.01(0.25 to 2.09)	1.09(0.77 to 1.51)	1.06(0.83 to 1.35)	-0.03(-0.36 to 0.44)
Ethiopia	Liver cancer due to NASH	54(36 to 80)	112(87 to 147)	1.07(0.25 to 2.23)	0.27(0.19 to 0.39)	0.28(0.21 to 0.37)	0.02(-0.34 to 0.53)
Ethiopia	Liver cancer due to other causes	111(67 to 193)	136(103 to 178)	0.23(-0.39 to 1.2)	0.24(0.16 to 0.38)	0.19(0.15 to 0.24)	-0.23(-0.57 to 0.26)
Fiji	Liver cancer	22(18 to 28)	47(36 to 61)	1.11(0.5 to 2.04)	5.72(4.62 to 7.07)	6.04(4.67 to 7.62)	0.06(-0.24 to 0.51)
Fiji	Liver cancer due to alcohol use	3(2 to 4)	6(4 to 10)	1.48(0.72 to 2.63)	0.7(0.42 to 1.07)	0.81(0.49 to 1.26)	0.16(-0.19 to 0.71)
Fiji	Liver cancer due to hepatitis B	13(9 to 16)	25(18 to 35)	0.97(0.35 to 1.86)	2.93(2.16 to 3.83)	2.96(2.12 to 4.08)	0.01(-0.29 to 0.47)
Fiji	Liver cancer due to hepatitis C	4(3 to 6)	9(6 to 14)	1.2(0.57 to 2.21)	1.34(0.88 to 1.91)	1.36(0.91 to 1.97)	0.01(-0.25 to 0.44)
Fiji	Liver cancer due to NASH	2(1 to 3)	5(3 to 7)	1.66(0.84 to 2.8)	0.48(0.31 to 0.71)	0.63(0.41 to 0.91)	0.31(-0.08 to 0.89)
Fiji	Liver cancer due to other causes	1(1 to 2)	2(2 to 3)	0.79(0.23 to 1.64)	0.27(0.18 to 0.4)	0.28(0.18 to 0.42)	0.03(-0.28 to 0.52)
Finland	Liver cancer	236(221 to 251)	670(530 to 857)	1.84(1.25 to 2.64)	3.32(3.11 to 3.53)	5.54(4.35 to	0.67(0.31 to 1.16)

						7.14)	
Finland	Liver cancer due to alcohol use	86(64 to 108)	257(176 to 359)	1.99(1.29 to 2.95)	1.2(0.9 to 1.5)	2.11(1.44 to 2.92)	0.76(0.35 to 1.32)
Finland	Liver cancer due to hepatitis B	33(23 to 47)	79(49 to 126)	1.4(0.79 to 2.21)	0.49(0.34 to 0.68)	0.76(0.49 to 1.17)	0.55(0.16 to 1.08)
Finland	Liver cancer due to hepatitis C	90(67 to 114)	258(181 to 359)	1.87(1.24 to 2.73)	1.23(0.93 to 1.56)	2(1.39 to 2.76)	0.62(0.27 to 1.1)
Finland	Liver cancer due to NASH	14(10 to 21)	46(29 to 71)	2.19(1.41 to 3.25)	0.2(0.13 to 0.29)	0.37(0.23 to 0.56)	0.86(0.41 to 1.48)
Finland	Liver cancer due to other causes	13(9 to 17)	30(19 to 46)	1.35(0.76 to 2.12)	0.2(0.15 to 0.26)	0.31(0.21 to 0.44)	0.52(0.17 to 1)
France	Liver cancer	3976(3786 to 4159)	8449(6482 to 10932)	1.13(0.63 to 1.75)	4.98(4.75 to 5.21)	6.66(5.05 to 8.72)	0.34(0.01 to 0.75)
France	Liver cancer due to alcohol use	1507(1098 to 1916)	2839(1841 to 4083)	0.88(0.41 to 1.46)	1.89(1.38 to 2.39)	2.27(1.48 to 3.32)	0.2(-0.11 to 0.58)
France	Liver cancer due to hepatitis B	555(375 to 785)	1058(646 to 1655)	0.91(0.41 to 1.58)	0.74(0.51 to 1.03)	0.96(0.6 to 1.47)	0.29(-0.04 to 0.77)
France	Liver cancer due to hepatitis C	1545(1143 to 2013)	3645(2536 to 5003)	1.36(0.8 to 2.08)	1.87(1.39 to 2.46)	2.68(1.84 to 3.71)	0.43(0.07 to 0.88)
France	Liver cancer due to NASH	195(133 to 292)	531(325 to 837)	1.72(1.03 to 2.59)	0.24(0.16 to 0.35)	0.4(0.25 to 0.61)	0.68(0.25 to 1.23)
France	Liver cancer due to other causes	174(124 to 245)	375(239 to 554)	1.16(0.62 to 1.84)	0.24(0.18 to 0.33)	0.35(0.23 to 0.51)	0.44(0.09 to 0.89)
Gabon	Liver cancer	20(14 to 25)	34(23 to 49)	0.75(0.11 to 1.65)	3.35(2.48 to 4.27)	3.27(2.15 to 4.67)	-0.02(-0.36 to 0.46)

Gabon	Liver cancer due to alcohol use	3(2 to 5)	6(3 to 11)	0.94(0.18 to 2.01)	0.56(0.33 to 0.86)	0.6(0.32 to 1.04)	0.07(-0.34 to 0.67)
Gabon	Liver cancer due to hepatitis B	5(3 to 7)	8(5 to 13)	0.8(0.12 to 1.85)	0.72(0.46 to 1.03)	0.67(0.39 to 1.09)	-0.06(-0.41 to 0.46)
Gabon	Liver cancer due to hepatitis C	9(6 to 12)	15(9 to 23)	0.7(0.1 to 1.6)	1.67(1.17 to 2.22)	1.58(1.02 to 2.33)	-0.05(-0.38 to 0.45)
Gabon	Liver cancer due to NASH	1(1 to 2)	3(2 to 4)	1.37(0.48 to 2.67)	0.21(0.13 to 0.31)	0.27(0.16 to 0.42)	0.29(-0.17 to 1.02)
Gabon	Liver cancer due to other causes	2(1 to 3)	2(1 to 3)	0.1(-0.32 to 0.79)	0.18(0.11 to 0.27)	0.14(0.09 to 0.21)	-0.22(-0.49 to 0.19)
Gambia	Liver cancer	121(89 to 162)	409(292 to 541)	2.37(1.26 to 4.01)	29.75(22.26 to 39.05)	38.21(27.53 to 49.67)	0.28(-0.15 to 0.86)
Gambia	Liver cancer due to alcohol use	19(12 to 30)	73(42 to 111)	2.78(1.44 to 4.64)	5.18(3.29 to 7.79)	7.37(4.25 to 11.17)	0.42(-0.07 to 1.09)
Gambia	Liver cancer due to hepatitis B	72(51 to 99)	229(159 to 323)	2.18(1.08 to 3.85)	17.02(12.08 to 23.08)	20.27(14.09 to 28.56)	0.19(-0.22 to 0.79)
Gambia	Liver cancer due to hepatitis C	13(8 to 19)	49(31 to 73)	2.79(1.54 to 4.49)	4.03(2.54 to 5.88)	5.47(3.43 to 8.11)	0.36(-0.07 to 0.9)
Gambia	Liver cancer due to NASH	7(5 to 11)	32(20 to 47)	3.3(1.88 to 5.3)	2.05(1.33 to 3.01)	3.2(2.05 to 4.8)	0.56(0.05 to 1.22)
Gambia	Liver cancer due to other causes	10(6 to 14)	26(18 to 36)	1.71(0.81 to 3.15)	1.46(0.93 to 2.1)	1.91(1.25 to 2.79)	0.31(-0.09 to 0.88)
Georgia	Liver cancer	68(62 to 75)	200(164 to 244)	1.92(1.36 to 2.63)	1.14(1.03 to 1.25)	3.52(2.87 to 4.32)	2.09(1.47 to 2.84)
Georgia	Liver cancer due to alcohol use	18(12 to 24)	68(47 to 91)	2.8(1.98 to 3.9)	0.29(0.2 to 0.38)	1.18(0.83 to	3.12(2.23 to 4.31)

						1.59)	
Georgia	Liver cancer due to hepatitis B	16(12 to 22)	47(32 to 67)	1.87(1.25 to 2.65)	0.26(0.19 to 0.36)	0.89(0.62 to 1.28)	2.4(1.65 to 3.37)
Georgia	Liver cancer due to hepatitis C	24(18 to 31)	65(46 to 87)	1.68(1.18 to 2.27)	0.41(0.31 to 0.52)	1.07(0.76 to 1.44)	1.62(1.11 to 2.24)
Georgia	Liver cancer due to NASH	5(3 to 7)	14(10 to 20)	2.05(1.41 to 2.88)	0.08(0.05 to 0.11)	0.24(0.17 to 0.34)	2.05(1.41 to 2.91)
Georgia	Liver cancer due to other causes	5(3 to 8)	6(4 to 9)	0.19(-0.3 to 1.04)	0.1(0.06 to 0.16)	0.14(0.09 to 0.2)	0.32(-0.26 to 1.4)
Germany	Liver cancer	3171(2931 to 3425)	9300(7292 to 11872)	1.93(1.23 to 2.76)	2.55(2.36 to 2.75)	5.07(3.96 to 6.44)	0.99(0.5 to 1.58)
Germany	Liver cancer due to alcohol use	1393(1123 to 1672)	4400(3178 to 5988)	2.16(1.35 to 3.12)	1.11(0.9 to 1.33)	2.42(1.74 to 3.3)	1.17(0.6 to 1.87)
Germany	Liver cancer due to hepatitis B	387(277 to 529)	888(556 to 1358)	1.3(0.69 to 2)	0.33(0.24 to 0.45)	0.55(0.35 to 0.83)	0.66(0.23 to 1.19)
Germany	Liver cancer due to hepatitis C	957(735 to 1207)	2725(1845 to 3793)	1.85(1.17 to 2.68)	0.74(0.57 to 0.93)	1.37(0.93 to 1.9)	0.86(0.4 to 1.4)
Germany	Liver cancer due to NASH	227(159 to 316)	773(482 to 1170)	2.4(1.52 to 3.44)	0.18(0.12 to 0.24)	0.4(0.26 to 0.6)	1.26(0.67 to 1.97)
Germany	Liver cancer due to other causes	207(152 to 281)	514(334 to 758)	1.48(0.85 to 2.24)	0.19(0.15 to 0.25)	0.33(0.23 to 0.48)	0.73(0.31 to 1.24)
Ghana	Liver cancer	393(299 to 526)	980(725 to 1260)	1.5(0.64 to 2.54)	5.83(4.49 to 7.75)	5.68(4.31 to 7.2)	-0.03(-0.36 to 0.37)
Ghana	Liver cancer due to alcohol use	71(44 to 111)	204(130 to 299)	1.89(0.79 to 3.22)	1.14(0.71 to 1.75)	1.27(0.82 to 1.85)	0.12(-0.3 to 0.64)

Ghana	Liver cancer due to hepatitis B	215(154 to 297)	515(359 to 699)	1.4(0.49 to 2.5)	3.01(2.15 to 4.13)	2.77(1.94 to 3.78)	-0.08(-0.42 to 0.33)
Ghana	Liver cancer due to hepatitis C	57(35 to 83)	128(78 to 190)	1.27(0.56 to 2.2)	1.03(0.66 to 1.49)	0.9(0.58 to 1.31)	-0.12(-0.4 to 0.23)
Ghana	Liver cancer due to NASH	24(15 to 36)	74(49 to 110)	2.11(1.09 to 3.39)	0.39(0.25 to 0.6)	0.47(0.31 to 0.7)	0.2(-0.19 to 0.7)
Ghana	Liver cancer due to other causes	27(19 to 37)	59(41 to 83)	1.21(0.52 to 2.04)	0.26(0.18 to 0.37)	0.27(0.18 to 0.38)	0.01(-0.3 to 0.36)
Greece	Liver cancer	346(324 to 368)	802(643 to 1002)	1.32(0.83 to 1.93)	2.28(2.14 to 2.42)	3.52(2.78 to 4.42)	0.54(0.2 to 0.96)
Greece	Liver cancer due to alcohol use	117(87 to 150)	274(188 to 384)	1.34(0.81 to 2.01)	0.75(0.55 to 0.96)	1.18(0.82 to 1.67)	0.58(0.22 to 1.06)
Greece	Liver cancer due to hepatitis B	121(91 to 154)	269(183 to 381)	1.22(0.7 to 1.87)	0.81(0.62 to 1.01)	1.31(0.92 to 1.84)	0.62(0.23 to 1.09)
Greece	Liver cancer due to hepatitis C	65(45 to 88)	158(104 to 229)	1.44(0.9 to 2.13)	0.42(0.3 to 0.57)	0.59(0.38 to 0.86)	0.39(0.09 to 0.75)
Greece	Liver cancer due to NASH	24(16 to 35)	63(40 to 96)	1.64(1.01 to 2.4)	0.15(0.1 to 0.22)	0.24(0.16 to 0.37)	0.58(0.21 to 1.03)
Greece	Liver cancer due to other causes	19(14 to 26)	38(24 to 57)	0.98(0.49 to 1.58)	0.15(0.11 to 0.19)	0.2(0.13 to 0.28)	0.34(0.03 to 0.73)
Greenland	Liver cancer	2(1 to 2)	4(3 to 6)	1.61(0.93 to 2.49)	4.48(3.91 to 5.18)	6.2(4.93 to 7.74)	0.38(0.05 to 0.83)
Greenland	Liver cancer due to alcohol use	1(1 to 1)	2(1 to 3)	1.73(0.98 to 2.73)	1.84(1.37 to 2.37)	2.64(1.83 to 3.62)	0.43(0.06 to 0.91)

Greenland	Liver cancer due to hepatitis B	0(0 to 0)	1(0 to 1)	1.08(0.46 to 1.92)	0.62(0.42 to 0.94)	0.78(0.48 to 1.18)	0.25(-0.08 to 0.67)
Greenland	Liver cancer due to hepatitis C	0(0 to 1)	1(1 to 2)	1.84(1.11 to 2.75)	1.19(0.82 to 1.63)	1.65(1.09 to 2.39)	0.38(0.06 to 0.79)
Greenland	Liver cancer due to NASH	0(0 to 0)	0(0 to 1)	2.08(1.26 to 3.16)	0.4(0.28 to 0.58)	0.63(0.41 to 0.91)	0.56(0.17 to 1.05)
Greenland	Liver cancer due to other causes	0(0 to 0)	0(0 to 1)	1.02(0.42 to 1.82)	0.42(0.3 to 0.56)	0.51(0.33 to 0.75)	0.22(-0.11 to 0.62)
Grenada	Liver cancer	6(6 to 7)	3(3 to 4)	-0.48(-0.56 to -0.39)	8.92(7.97 to 9.89)	2.96(2.61 to 3.36)	-0.67(-0.72 to -0.61)
Grenada	Liver cancer due to alcohol use	2(2 to 3)	1(1 to 2)	-0.4(-0.5 to -0.27)	2.99(2.17 to 3.89)	1.13(0.83 to 1.45)	-0.62(-0.69 to -0.55)
Grenada	Liver cancer due to hepatitis B	2(1 to 2)	1(1 to 1)	-0.5(-0.59 to -0.4)	2.57(1.91 to 3.42)	0.74(0.52 to 1.02)	-0.71(-0.76 to -0.66)
Grenada	Liver cancer due to hepatitis C	2(1 to 2)	1(0 to 1)	-0.57(-0.64 to -0.48)	2.09(1.45 to 2.8)	0.65(0.44 to 0.89)	-0.69(-0.74 to -0.63)
Grenada	Liver cancer due to NASH	0(0 to 1)	0(0 to 0)	-0.43(-0.53 to -0.3)	0.65(0.45 to 0.92)	0.26(0.18 to 0.38)	-0.6(-0.67 to -0.51)
Grenada	Liver cancer due to other causes	0(0 to 1)	0(0 to 0)	-0.56(-0.64 to -0.46)	0.62(0.44 to 0.83)	0.19(0.13 to 0.25)	-0.7(-0.75 to -0.63)
Guam	Liver cancer	3(3 to 4)	11(9 to 14)	2.74(1.87 to 3.84)	3.75(3.21 to 4.37)	5.89(4.8 to 7.19)	0.57(0.22 to 1.01)
Guam	Liver cancer due to alcohol use	0(0 to 1)	1(1 to 2)	3.11(2.05 to 4.54)	0.44(0.28 to 0.65)	0.73(0.45 to 1.09)	0.65(0.23 to 1.2)

Guam	Liver cancer due to hepatitis B	2(1 to 2)	7(5 to 9)	2.8(1.86 to 4.08)	1.98(1.56 to 2.53)	3.49(2.66 to 4.45)	0.76(0.36 to 1.33)
Guam	Liver cancer due to hepatitis C	1(0 to 1)	2(1 to 3)	2.35(1.57 to 3.32)	0.82(0.55 to 1.13)	0.91(0.59 to 1.34)	0.11(-0.14 to 0.41)
Guam	Liver cancer due to NASH	0(0 to 0)	1(1 to 2)	3.33(2.27 to 4.68)	0.35(0.24 to 0.5)	0.56(0.38 to 0.83)	0.61(0.22 to 1.1)
Guam	Liver cancer due to other causes	0(0 to 0)	0(0 to 1)	1.57(0.94 to 2.34)	0.16(0.11 to 0.22)	0.2(0.14 to 0.29)	0.27(-0.03 to 0.64)
Guatemala	Liver cancer	344(305 to 388)	498(394 to 628)	0.45(0.12 to 0.88)	9.33(8.27 to 10.5)	4.47(3.55 to 5.6)	-0.52(-0.63 to -0.38)
Guatemala	Liver cancer due to alcohol use	82(57 to 112)	124(81 to 180)	0.51(0.13 to 1.02)	2.27(1.6 to 3.06)	1.13(0.73 to 1.64)	-0.51(-0.63 to -0.34)
Guatemala	Liver cancer due to hepatitis B	74(54 to 100)	90(60 to 131)	0.21(-0.08 to 0.59)	1.73(1.21 to 2.39)	0.74(0.48 to 1.1)	-0.57(-0.68 to -0.44)
Guatemala	Liver cancer due to hepatitis C	132(99 to 167)	200(142 to 273)	0.51(0.16 to 0.97)	4.03(3.07 to 5)	1.9(1.37 to 2.58)	-0.53(-0.63 to -0.4)
Guatemala	Liver cancer due to NASH	23(16 to 32)	44(28 to 64)	0.88(0.42 to 1.52)	0.65(0.45 to 0.91)	0.39(0.25 to 0.59)	-0.4(-0.54 to -0.19)
Guatemala	Liver cancer due to other causes	32(25 to 42)	41(29 to 56)	0.27(-0.05 to 0.65)	0.64(0.47 to 0.86)	0.31(0.21 to 0.43)	-0.52(-0.64 to -0.37)
Guinea	Liver cancer	1032(859 to 1243)	1881(1308 to 2447)	0.82(0.21 to 1.51)	29.91(24.86 to 35.83)	32.17(22.33 to 41.9)	0.08(-0.28 to 0.49)
Guinea	Liver cancer due to alcohol use	160(103 to 237)	313(181 to 476)	0.96(0.23 to 1.8)	4.81(3.11 to 7.15)	5.74(3.32 to 8.62)	0.19(-0.25 to 0.71)

Guinea	Liver cancer due to hepatitis B	567(434 to 731)	1038(690 to 1420)	0.83(0.19 to 1.57)	16.25(12.38 to 20.89)	17(11.25 to 23.24)	0.05(-0.32 to 0.47)
Guinea	Liver cancer due to hepatitis C	165(106 to 234)	272(165 to 405)	0.65(0.16 to 1.25)	5.35(3.51 to 7.46)	5.38(3.29 to 8)	0.01(-0.28 to 0.36)
Guinea	Liver cancer due to NASH	67(44 to 97)	137(87 to 208)	1.03(0.42 to 1.81)	2.08(1.38 to 3.01)	2.52(1.59 to 3.89)	0.21(-0.14 to 0.67)
Guinea	Liver cancer due to other causes	72(50 to 102)	122(79 to 178)	0.69(0.11 to 1.49)	1.42(1.01 to 1.99)	1.52(0.99 to 2.27)	0.07(-0.26 to 0.46)
Guinea-Bissau	Liver cancer	35(25 to 51)	48(34 to 66)	0.36(-0.05 to 0.92)	8.07(5.85 to 11.34)	6.12(4.45 to 8.28)	-0.24(-0.46 to 0.06)
Guinea-Bissau	Liver cancer due to alcohol use	6(3 to 9)	8(5 to 12)	0.38(-0.05 to 1.03)	1.38(0.83 to 2.21)	1.09(0.67 to 1.66)	-0.21(-0.45 to 0.13)
Guinea-Bissau	Liver cancer due to hepatitis B	19(13 to 29)	25(17 to 36)	0.29(-0.12 to 0.87)	4.23(2.86 to 6.06)	2.9(1.98 to 4.08)	-0.32(-0.53 to -0.02)
Guinea-Bissau	Liver cancer due to hepatitis C	5(3 to 9)	8(5 to 12)	0.4(0.02 to 0.93)	1.49(0.9 to 2.33)	1.24(0.75 to 1.89)	-0.17(-0.38 to 0.14)
Guinea-Bissau	Liver cancer due to NASH	2(1 to 4)	4(2 to 6)	0.7(0.2 to 1.38)	0.58(0.36 to 0.91)	0.57(0.35 to 0.87)	-0.02(-0.3 to 0.35)
Guinea-Bissau	Liver cancer due to other causes	2(2 to 4)	3(2 to 5)	0.36(-0.03 to 0.97)	0.39(0.24 to 0.59)	0.33(0.2 to 0.52)	-0.16(-0.4 to 0.19)
Guyana	Liver cancer	26(22 to 30)	19(15 to 24)	-0.27(-0.46 to -0.03)	6.52(5.61 to 7.45)	3.03(2.36 to 3.84)	-0.54(-0.65 to -0.4)
Guyana	Liver cancer due to alcohol use	9(6 to 11)	7(4 to 9)	-0.26(-0.45 to -0.01)	2.29(1.65 to 3.03)	1.05(0.71 to 1.46)	-0.54(-0.66 to -0.4)
Guyana	Liver cancer due to hepatitis B	8(6 to 11)	6(4 to 8)	-0.31(-0.51 to -0.07)	1.84(1.32 to 2.52)	0.82(0.54 to	-0.56(-0.67 to -0.41)

						1.18)	
Guyana	Liver cancer due to hepatitis C	5(3 to 7)	4(2 to 6)	-0.24(-0.44 to -0.02)	1.46(1 to 2.03)	0.67(0.43 to 0.96)	-0.54(-0.65 to -0.4)
Guyana	Liver cancer due to NASH	2(1 to 3)	2(1 to 3)	-0.08(-0.32 to 0.24)	0.48(0.33 to 0.69)	0.28(0.18 to 0.41)	-0.42(-0.56 to -0.23)
Guyana	Liver cancer due to other causes	2(2 to 3)	1(1 to 2)	-0.35(-0.53 to -0.12)	0.45(0.32 to 0.61)	0.21(0.14 to 0.3)	-0.53(-0.66 to -0.37)
Haiti	Liver cancer	220(137 to 300)	290(176 to 432)	0.32(-0.06 to 0.83)	6.65(4.16 to 9.25)	4.14(2.49 to 6.26)	-0.38(-0.55 to -0.15)
Haiti	Liver cancer due to alcohol use	68(39 to 107)	92(49 to 158)	0.36(-0.05 to 0.91)	2.11(1.21 to 3.37)	1.37(0.74 to 2.31)	-0.35(-0.54 to -0.09)
Haiti	Liver cancer due to hepatitis B	69(40 to 108)	92(51 to 153)	0.34(-0.08 to 0.89)	1.91(1.13 to 3.04)	1.19(0.66 to 2.02)	-0.38(-0.57 to -0.13)
Haiti	Liver cancer due to hepatitis C	49(26 to 79)	61(33 to 99)	0.25(-0.11 to 0.74)	1.69(0.95 to 2.74)	0.98(0.53 to 1.56)	-0.42(-0.59 to -0.2)
Haiti	Liver cancer due to NASH	14(7 to 22)	19(10 to 32)	0.4(-0.01 to 0.99)	0.45(0.25 to 0.74)	0.29(0.15 to 0.48)	-0.35(-0.53 to -0.08)
Haiti	Liver cancer due to other causes	21(12 to 35)	26(15 to 40)	0.22(-0.16 to 0.73)	0.5(0.28 to 0.8)	0.3(0.17 to 0.47)	-0.4(-0.57 to -0.17)
Honduras	Liver cancer	231(92 to 333)	879(408 to 1299)	2.8(1.9 to 4.39)	11.06(4.16 to 16.11)	14.8(6.8 to 21.7)	0.34(0.02 to 0.95)
Honduras	Liver cancer due to alcohol use	60(19 to 100)	252(104 to 414)	3.21(2.13 to 5.43)	2.96(0.91 to 4.92)	4.22(1.75 to 6.85)	0.43(0.07 to 1.21)
Honduras	Liver cancer due to hepatitis B	51(19 to 85)	158(64 to 273)	2.09(1.22 to 3.42)	2.21(0.75 to 3.78)	2.46(0.97 to	0.11(-0.19 to 0.62)

						4.31)	
Honduras	Liver cancer due to hepatitis C	86(29 to 137)	343(140 to 558)	2.98(2.05 to 4.82)	4.48(1.48 to 7.06)	6.06(2.5 to 9.75)	0.35(0.04 to 1)
Honduras	Liver cancer due to NASH	15(5 to 26)	73(28 to 127)	3.85(2.57 to 6.13)	0.73(0.22 to 1.3)	1.24(0.47 to 2.19)	0.71(0.27 to 1.56)
Honduras	Liver cancer due to other causes	19(9 to 29)	54(23 to 91)	1.82(1.01 to 2.92)	0.69(0.28 to 1.15)	0.82(0.33 to 1.39)	0.19(-0.13 to 0.7)
Hungary	Liver cancer	952(909 to 993)	486(400 to 592)	-0.49(-0.58 to -0.37)	6.47(6.18 to 6.74)	2.58(2.11 to 3.16)	-0.6(-0.67 to -0.51)
Hungary	Liver cancer due to alcohol use	426(340 to 505)	209(149 to 277)	-0.51(-0.61 to -0.38)	2.84(2.28 to 3.37)	1.09(0.78 to 1.45)	-0.62(-0.7 to -0.52)
Hungary	Liver cancer due to hepatitis B	209(148 to 287)	102(69 to 150)	-0.51(-0.61 to -0.39)	1.46(1.06 to 1.97)	0.59(0.4 to 0.84)	-0.6(-0.68 to -0.49)
Hungary	Liver cancer due to hepatitis C	220(153 to 292)	119(78 to 168)	-0.46(-0.56 to -0.34)	1.49(1.05 to 1.95)	0.59(0.39 to 0.84)	-0.6(-0.68 to -0.51)
Hungary	Liver cancer due to NASH	66(46 to 93)	41(28 to 58)	-0.39(-0.52 to -0.21)	0.45(0.32 to 0.63)	0.21(0.14 to 0.3)	-0.54(-0.64 to -0.41)
Hungary	Liver cancer due to other causes	31(21 to 42)	15(10 to 22)	-0.51(-0.62 to -0.39)	0.23(0.17 to 0.31)	0.1(0.07 to 0.14)	-0.55(-0.64 to -0.43)
Iceland	Liver cancer	6(5 to 6)	19(16 to 22)	2.29(1.77 to 2.91)	2.03(1.86 to 2.23)	3.52(3.06 to 4.05)	0.74(0.46 to 1.06)
Iceland	Liver cancer due to alcohol use	2(1 to 2)	7(5 to 9)	2.82(2.11 to 3.68)	0.64(0.46 to 0.85)	1.29(0.94 to 1.68)	1.02(0.64 to 1.46)
Iceland	Liver cancer due to hepatitis B	1(1 to 1)	3(2 to 4)	2.07(1.55 to 2.69)	0.35(0.24 to 0.48)	0.58(0.4 to	0.68(0.4 to 1.03)

						0.83)	
Iceland	Liver cancer due to hepatitis C	2(2 to 3)	7(5 to 9)	2.08(1.56 to 2.71)	0.76(0.57 to 0.97)	1.2(0.87 to 1.59)	0.58(0.32 to 0.9)
Iceland	Liver cancer due to NASH	0(0 to 1)	1(1 to 2)	2.5(1.84 to 3.38)	0.13(0.09 to 0.19)	0.24(0.16 to 0.35)	0.84(0.5 to 1.31)
Iceland	Liver cancer due to other causes	0(0 to 1)	1(1 to 1)	1.42(0.95 to 1.92)	0.15(0.12 to 0.2)	0.21(0.15 to 0.28)	0.4(0.14 to 0.67)
India	Liver cancer	11777(9852 to 13689)	29968(25244 to 35612)	1.54(1.04 to 2.1)	2.54(2.09 to 2.97)	2.61(2.2 to 3.1)	0.03(-0.17 to 0.25)
India	Liver cancer due to alcohol use	2841(2184 to 3559)	8285(6535 to 10413)	1.92(1.29 to 2.68)	0.65(0.49 to 0.8)	0.72(0.57 to 0.9)	0.12(-0.12 to 0.41)
India	Liver cancer due to hepatitis B	5032(4036 to 6059)	10910(8722 to 13397)	1.17(0.71 to 1.69)	0.99(0.78 to 1.19)	0.9(0.72 to 1.11)	-0.09(-0.28 to 0.13)
India	Liver cancer due to hepatitis C	1935(1517 to 2422)	5842(4521 to 7313)	2.02(1.42 to 2.68)	0.51(0.4 to 0.63)	0.55(0.43 to 0.69)	0.08(-0.13 to 0.31)
India	Liver cancer due to NASH	1111(879 to 1367)	3339(2656 to 4188)	2(1.44 to 2.65)	0.27(0.21 to 0.33)	0.3(0.24 to 0.38)	0.13(-0.08 to 0.37)
India	Liver cancer due to other causes	857(704 to 1087)	1592(1284 to 1997)	0.86(0.53 to 1.21)	0.13(0.1 to 0.15)	0.13(0.1 to 0.16)	0.03(-0.13 to 0.22)
Indonesia	Liver cancer	2472(2137 to 2837)	4504(3796 to 5158)	0.82(0.45 to 1.26)	2.43(2.1 to 2.8)	2.23(1.9 to 2.51)	-0.08(-0.27 to 0.12)
Indonesia	Liver cancer due to alcohol use	407(319 to 504)	844(649 to 1073)	1.07(0.61 to 1.68)	0.42(0.33 to 0.51)	0.4(0.31 to 0.5)	-0.04(-0.25 to 0.24)
Indonesia	Liver cancer due to hepatitis B	604(492 to 721)	993(767 to 1255)	0.65(0.29 to 1.09)	0.5(0.41 to 0.6)	0.43(0.33 to 0.53)	-0.15(-0.33 to 0.07)

Indonesia	Liver cancer due to hepatitis C	1015(856 to 1211)	2002(1634 to 2333)	0.97(0.54 to 1.43)	1.16(0.98 to 1.39)	1.08(0.89 to 1.23)	-0.07(-0.27 to 0.14)
Indonesia	Liver cancer due to NASH	214(172 to 261)	444(350 to 539)	1.08(0.63 to 1.59)	0.21(0.17 to 0.26)	0.22(0.18 to 0.27)	0.05(-0.16 to 0.29)
Indonesia	Liver cancer due to other causes	233(179 to 311)	220(179 to 267)	-0.05(-0.33 to 0.27)	0.14(0.11 to 0.18)	0.1(0.08 to 0.12)	-0.29(-0.46 to -0.08)
Iran (Islamic Republic of)	Liver cancer	1068(881 to 1245)	2722(2475 to 2991)	1.55(1.04 to 2.24)	4.26(3.45 to 5.11)	3.85(3.5 to 4.25)	-0.1(-0.3 to 0.17)
Iran (Islamic Republic of)	Liver cancer due to alcohol use	115(83 to 154)	279(211 to 364)	1.43(1.03 to 2.11)	0.47(0.34 to 0.6)	0.41(0.3 to 0.53)	-0.13(-0.28 to 0.1)
Iran (Islamic Republic of)	Liver cancer due to hepatitis B	466(384 to 550)	1072(918 to 1245)	1.3(0.92 to 1.86)	1.67(1.37 to 1.98)	1.42(1.2 to 1.66)	-0.15(-0.3 to 0.07)
Iran (Islamic Republic of)	Liver cancer due to hepatitis C	258(198 to 340)	764(649 to 891)	1.96(1.14 to 2.97)	1.26(0.96 to 1.66)	1.16(0.98 to 1.35)	-0.08(-0.34 to 0.26)
Iran (Islamic Republic of)	Liver cancer due to NASH	116(88 to 150)	389(326 to 461)	2.35(1.46 to 3.45)	0.52(0.39 to 0.69)	0.57(0.48 to 0.68)	0.1(-0.21 to 0.48)
Iran (Islamic Republic of)	Liver cancer due to other causes	112(92 to 135)	218(186 to 253)	0.94(0.57 to 1.39)	0.35(0.28 to 0.44)	0.3(0.25 to 0.35)	-0.15(-0.36 to 0.09)
Iraq	Liver cancer	353(273 to 439)	1407(1069 to 1783)	2.98(1.8 to 4.59)	4.38(3.37 to 5.43)	6.09(4.71 to 7.57)	0.39(-0.01 to 0.96)
Iraq	Liver cancer due to alcohol use	31(19 to 48)	130(78 to 198)	3.14(1.82 to 5)	0.41(0.25 to 0.64)	0.59(0.35 to 0.89)	0.43(-0.02 to 1.06)
Iraq	Liver cancer due to hepatitis B	136(96 to 187)	525(359 to 745)	2.86(1.61 to 4.63)	1.59(1.1 to 2.22)	2.04(1.38 to 2.88)	0.28(-0.11 to 0.82)

Iraq	Liver cancer due to hepatitis C	120(82 to 166)	490(330 to 682)	3.08(1.88 to 4.87)	1.65(1.14 to 2.26)	2.37(1.64 to 3.28)	0.43(0.02 to 1.02)
Iraq	Liver cancer due to NASH	32(21 to 47)	152(97 to 227)	3.76(2.31 to 5.9)	0.42(0.27 to 0.61)	0.69(0.45 to 1)	0.66(0.16 to 1.39)
Iraq	Liver cancer due to other causes	34(24 to 45)	111(75 to 161)	2.25(1.28 to 3.58)	0.31(0.21 to 0.43)	0.41(0.27 to 0.6)	0.32(-0.08 to 0.85)
Ireland	Liver cancer	60(56 to 64)	295(227 to 374)	3.91(2.78 to 5.3)	1.46(1.36 to 1.56)	4(3.07 to 5.1)	1.73(1.1 to 2.52)
Ireland	Liver cancer due to alcohol use	21(16 to 27)	110(75 to 155)	4.19(2.86 to 5.84)	0.51(0.38 to 0.65)	1.48(1 to 2.08)	1.91(1.15 to 2.84)
Ireland	Liver cancer due to hepatitis B	8(6 to 12)	37(23 to 58)	3.6(2.47 to 5.11)	0.21(0.14 to 0.29)	0.54(0.34 to 0.82)	1.6(0.94 to 2.42)
Ireland	Liver cancer due to hepatitis C	23(17 to 29)	111(75 to 154)	3.89(2.75 to 5.32)	0.55(0.42 to 0.68)	1.46(0.99 to 2.03)	1.65(1.05 to 2.43)
Ireland	Liver cancer due to NASH	4(3 to 6)	21(13 to 33)	4.64(3.23 to 6.48)	0.09(0.06 to 0.13)	0.28(0.18 to 0.43)	2.13(1.36 to 3.14)
Ireland	Liver cancer due to other causes	4(3 to 6)	15(10 to 22)	2.63(1.71 to 3.72)	0.11(0.08 to 0.14)	0.24(0.16 to 0.34)	1.18(0.66 to 1.8)
Israel	Liver cancer	122(109 to 135)	337(263 to 429)	1.77(1.13 to 2.62)	2.49(2.23 to 2.74)	2.97(2.31 to 3.79)	0.2(-0.08 to 0.56)
Israel	Liver cancer due to alcohol use	28(19 to 39)	89(57 to 130)	2.14(1.33 to 3.22)	0.57(0.39 to 0.78)	0.78(0.5 to 1.16)	0.37(0.02 to 0.85)
Israel	Liver cancer due to hepatitis B	20(14 to 29)	51(33 to 78)	1.58(0.91 to 2.42)	0.41(0.29 to 0.59)	0.48(0.31 to 0.73)	0.15(-0.15 to 0.55)
Israel	Liver cancer due to hepatitis C	56(42 to 70)	148(105 to 204)	1.66(1.07 to 2.44)	1.14(0.87 to 1.42)	1.27(0.9 to 1.74)	0.11(-0.14 to 0.44)

Israel	Liver cancer due to NASH	9(6 to 14)	27(17 to 41)	1.98(1.24 to 2.97)	0.18(0.12 to 0.27)	0.24(0.15 to 0.36)	0.28(-0.03 to 0.69)
Israel	Liver cancer due to other causes	9(6 to 12)	22(15 to 32)	1.5(0.9 to 2.27)	0.18(0.13 to 0.24)	0.21(0.14 to 0.3)	0.16(-0.11 to 0.55)
Italy	Liver cancer	5982(5811 to 6130)	7928(6450 to 9653)	0.33(0.08 to 0.61)	6.77(6.58 to 6.94)	5.97(4.81 to 7.37)	-0.12(-0.28 to 0.09)
Italy	Liver cancer due to alcohol use	1673(1448 to 1897)	2059(1556 to 2673)	0.23(-0.05 to 0.57)	1.88(1.63 to 2.13)	1.6(1.2 to 2.1)	-0.15(-0.35 to 0.09)
Italy	Liver cancer due to hepatitis B	761(637 to 906)	849(633 to 1101)	0.12(-0.11 to 0.4)	0.91(0.78 to 1.08)	0.75(0.56 to 0.98)	-0.18(-0.35 to 0.04)
Italy	Liver cancer due to hepatitis C	3109(2833 to 3394)	4438(3568 to 5463)	0.43(0.17 to 0.71)	3.45(3.14 to 3.77)	3.14(2.5 to 3.92)	-0.09(-0.25 to 0.1)
Italy	Liver cancer due to NASH	270(226 to 319)	373(287 to 475)	0.38(0.14 to 0.66)	0.3(0.25 to 0.35)	0.27(0.21 to 0.34)	-0.11(-0.27 to 0.06)
Italy	Liver cancer due to other causes	170(145 to 198)	210(164 to 263)	0.23(0.03 to 0.46)	0.23(0.2 to 0.27)	0.22(0.17 to 0.27)	-0.07(-0.21 to 0.09)
Jamaica	Liver cancer	70(65 to 75)	77(61 to 96)	0.1(-0.14 to 0.41)	3.89(3.64 to 4.15)	2.61(2.07 to 3.25)	-0.33(-0.48 to -0.15)
Jamaica	Liver cancer due to alcohol use	22(17 to 29)	26(17 to 35)	0.15(-0.12 to 0.46)	1.25(0.93 to 1.61)	0.87(0.6 to 1.21)	-0.3(-0.46 to -0.11)
Jamaica	Liver cancer due to hepatitis B	20(15 to 27)	21(14 to 30)	0.03(-0.23 to 0.36)	1.17(0.86 to 1.53)	0.71(0.48 to 1)	-0.39(-0.54 to -0.21)
Jamaica	Liver cancer due to hepatitis C	16(11 to 21)	18(12 to 25)	0.08(-0.17 to 0.37)	0.9(0.62 to 1.17)	0.59(0.38 to 0.85)	-0.35(-0.5 to -0.17)
Jamaica	Liver cancer due to NASH	6(4 to 8)	8(5 to 11)	0.37(0.02 to 0.8)	0.3(0.21 to 0.43)	0.25(0.17 to 0.38)	-0.16(-0.37 to 0.11)

Jamaica	Liver cancer due to other causes	5(4 to 7)	5(4 to 7)	0(-0.24 to 0.29)	0.27(0.2 to 0.37)	0.18(0.12 to 0.26)	-0.33(-0.5 to -0.13)
Japan	Liver cancer	24465(23601 to 25148)	46660(37995 to 55120)	0.91(0.58 to 1.22)	14.22(13.71 to 14.62)	12.71(10.51 to 14.98)	-0.11(-0.25 to 0.05)
Japan	Liver cancer due to alcohol use	3199(2708 to 3704)	5040(3874 to 6362)	0.58(0.28 to 0.93)	1.84(1.56 to 2.13)	1.48(1.14 to 1.88)	-0.2(-0.34 to -0.03)
Japan	Liver cancer due to hepatitis B	3752(3266 to 4325)	4876(3780 to 6179)	0.3(0.05 to 0.58)	2.2(1.92 to 2.52)	1.65(1.3 to 2.08)	-0.25(-0.38 to -0.1)
Japan	Liver cancer due to hepatitis C	15780(14838 to 16676)	33312(26824 to 39444)	1.11(0.73 to 1.47)	9.14(8.59 to 9.66)	8.6(7.06 to 10.25)	-0.06(-0.21 to 0.1)
Japan	Liver cancer due to NASH	973(824 to 1148)	2195(1661 to 2802)	1.26(0.81 to 1.74)	0.57(0.48 to 0.67)	0.56(0.44 to 0.71)	-0.01(-0.17 to 0.17)
Japan	Liver cancer due to other causes	761(652 to 879)	1236(937 to 1549)	0.62(0.33 to 0.96)	0.47(0.41 to 0.54)	0.42(0.34 to 0.5)	-0.11(-0.23 to 0.03)
Jordan	Liver cancer	40(32 to 49)	154(124 to 191)	2.85(1.82 to 4.34)	3.01(2.36 to 3.78)	2.46(1.98 to 3.04)	-0.18(-0.41 to 0.14)
Jordan	Liver cancer due to alcohol use	4(2 to 6)	16(10 to 25)	3.41(1.99 to 5.46)	0.3(0.18 to 0.45)	0.27(0.16 to 0.42)	-0.1(-0.39 to 0.33)
Jordan	Liver cancer due to hepatitis B	16(12 to 22)	57(40 to 78)	2.45(1.47 to 3.95)	1.1(0.78 to 1.51)	0.8(0.56 to 1.13)	-0.27(-0.48 to 0.05)
Jordan	Liver cancer due to hepatitis C	12(8 to 17)	48(33 to 66)	3(1.83 to 4.71)	1.1(0.72 to 1.51)	0.89(0.61 to 1.2)	-0.19(-0.42 to 0.17)
Jordan	Liver cancer due to NASH	4(2 to 6)	19(13 to 28)	4.12(2.62 to 6.27)	0.31(0.2 to 0.48)	0.33(0.22 to 0.49)	0.06(-0.25 to 0.52)

Jordan	Liver cancer due to other causes	4(3 to 5)	13(10 to 18)	2.31(1.48 to 3.43)	0.21(0.14 to 0.3)	0.17(0.12 to 0.24)	-0.19(-0.4 to 0.13)
Kazakhstan	Liver cancer	450(419 to 482)	1101(946 to 1272)	1.44(1.08 to 1.86)	3.48(3.24 to 3.71)	6.3(5.43 to 7.24)	0.81(0.55 to 1.11)
Kazakhstan	Liver cancer due to alcohol use	137(99 to 175)	355(249 to 465)	1.58(1.15 to 2.11)	1.07(0.78 to 1.36)	2.01(1.43 to 2.63)	0.88(0.56 to 1.25)
Kazakhstan	Liver cancer due to hepatitis B	119(87 to 161)	264(182 to 376)	1.21(0.83 to 1.64)	0.88(0.63 to 1.18)	1.42(0.98 to 1.99)	0.62(0.35 to 0.92)
Kazakhstan	Liver cancer due to hepatitis C	140(102 to 181)	356(247 to 483)	1.54(1.15 to 1.98)	1.14(0.84 to 1.46)	2.14(1.5 to 2.86)	0.87(0.59 to 1.19)
Kazakhstan	Liver cancer due to NASH	25(18 to 36)	82(55 to 119)	2.25(1.67 to 2.92)	0.2(0.14 to 0.29)	0.49(0.33 to 0.72)	1.41(1 to 1.92)
Kazakhstan	Liver cancer due to other causes	28(18 to 41)	44(30 to 60)	0.56(-0.02 to 1.41)	0.18(0.12 to 0.26)	0.25(0.17 to 0.34)	0.34(-0.12 to 0.98)
Kenya	Liver cancer	219(151 to 384)	663(432 to 982)	2.03(1.41 to 2.86)	2.48(1.7 to 4.37)	2.9(1.92 to 4.25)	0.17(-0.06 to 0.45)
Kenya	Liver cancer due to alcohol use	54(33 to 100)	167(102 to 262)	2.09(1.43 to 2.93)	0.66(0.41 to 1.22)	0.76(0.47 to 1.21)	0.15(-0.09 to 0.45)
Kenya	Liver cancer due to hepatitis B	64(40 to 114)	200(123 to 306)	2.13(1.44 to 3.02)	0.66(0.41 to 1.17)	0.75(0.46 to 1.16)	0.13(-0.11 to 0.43)
Kenya	Liver cancer due to hepatitis C	44(30 to 76)	133(87 to 195)	2.04(1.45 to 2.78)	0.59(0.41 to 1.01)	0.7(0.47 to 1.01)	0.19(-0.04 to 0.45)
Kenya	Liver cancer due to NASH	28(20 to 48)	96(63 to 142)	2.37(1.69 to 3.25)	0.35(0.24 to 0.59)	0.45(0.3 to 0.65)	0.3(0.05 to 0.6)

Kenya	Liver cancer due to other causes	28(21 to 44)	67(46 to 98)	1.37(0.74 to 2.14)	0.22(0.16 to 0.36)	0.24(0.16 to 0.35)	0.09(-0.14 to 0.38)
Kiribati	Liver cancer	6(5 to 7)	9(7 to 12)	0.58(0.11 to 1.19)	13.92(10.8 to 17.27)	11.69(9.09 to 14.66)	-0.16(-0.4 to 0.16)
Kiribati	Liver cancer due to alcohol use	1(0 to 1)	1(1 to 1)	0.63(0.08 to 1.37)	1.37(0.82 to 2.1)	1.17(0.7 to 1.74)	-0.14(-0.42 to 0.25)
Kiribati	Liver cancer due to hepatitis B	4(3 to 5)	5(4 to 7)	0.53(0.05 to 1.15)	7.75(5.67 to 10.44)	6.08(4.38 to 8.17)	-0.21(-0.45 to 0.1)
Kiribati	Liver cancer due to hepatitis C	1(1 to 2)	2(1 to 2)	0.61(0.12 to 1.28)	3.07(2.06 to 4.33)	2.72(1.75 to 3.93)	-0.11(-0.37 to 0.24)
Kiribati	Liver cancer due to NASH	0(0 to 1)	1(1 to 1)	0.89(0.33 to 1.65)	1.07(0.69 to 1.61)	1.11(0.73 to 1.65)	0.03(-0.26 to 0.46)
Kiribati	Liver cancer due to other causes	0(0 to 0)	1(0 to 1)	0.67(0.19 to 1.34)	0.66(0.45 to 0.93)	0.6(0.4 to 0.89)	-0.09(-0.34 to 0.27)
Kuwait	Liver cancer	18(16 to 20)	66(52 to 82)	2.61(1.82 to 3.53)	2.78(2.47 to 3.1)	2.75(2.17 to 3.42)	-0.01(-0.22 to 0.24)
Kuwait	Liver cancer due to alcohol use	2(1 to 2)	6(4 to 10)	2.74(1.79 to 3.99)	0.28(0.18 to 0.42)	0.28(0.17 to 0.43)	-0.02(-0.27 to 0.29)
Kuwait	Liver cancer due to hepatitis B	8(6 to 10)	24(17 to 34)	1.97(1.25 to 2.86)	1.05(0.78 to 1.37)	0.84(0.56 to 1.22)	-0.2(-0.4 to 0.03)
Kuwait	Liver cancer due to hepatitis C	5(3 to 6)	21(15 to 29)	3.34(2.29 to 4.51)	0.96(0.69 to 1.22)	1.03(0.73 to 1.39)	0.08(-0.17 to 0.36)
Kuwait	Liver cancer due to NASH	2(1 to 2)	9(6 to 14)	4.2(2.87 to 5.82)	0.31(0.21 to 0.44)	0.42(0.27 to 0.63)	0.36(0.01 to 0.78)
Kuwait	Liver cancer due to other causes	2(1 to 2)	5(4 to 7)	1.78(1.14 to 2.51)	0.18(0.14 to 0.25)	0.18(0.13 to	-0.03(-0.24 to 0.23)

						0.25)	
Kyrgyzstan	Liver cancer	40(36 to 45)	114(97 to 132)	1.84(1.34 to 2.37)	1.27(1.16 to 1.38)	2.54(2.16 to 2.91)	1(0.68 to 1.35)
Kyrgyzstan	Liver cancer due to alcohol use	10(7 to 13)	34(24 to 45)	2.54(1.83 to 3.42)	0.32(0.23 to 0.43)	0.77(0.53 to 1.02)	1.38(0.93 to 1.95)
Kyrgyzstan	Liver cancer due to hepatitis B	10(7 to 13)	27(19 to 38)	1.85(1.3 to 2.48)	0.3(0.21 to 0.4)	0.54(0.37 to 0.76)	0.82(0.47 to 1.19)
Kyrgyzstan	Liver cancer due to hepatitis C	14(10 to 18)	39(28 to 51)	1.84(1.33 to 2.41)	0.47(0.35 to 0.59)	0.95(0.68 to 1.24)	1.01(0.66 to 1.4)
Kyrgyzstan	Liver cancer due to NASH	2(1 to 3)	7(5 to 10)	2.33(1.64 to 3.14)	0.07(0.05 to 0.1)	0.16(0.11 to 0.24)	1.33(0.87 to 1.9)
Kyrgyzstan	Liver cancer due to other causes	5(3 to 9)	6(5 to 9)	0.28(-0.3 to 1.56)	0.11(0.06 to 0.17)	0.12(0.08 to 0.16)	0.1(-0.36 to 0.92)
Lao People's Democratic Republic	Liver cancer	220(150 to 300)	305(223 to 395)	0.39(-0.11 to 1.13)	9.99(6.95 to 13.51)	6.72(5.06 to 8.54)	-0.33(-0.55 to -0.01)
Lao People's Democratic Republic	Liver cancer due to alcohol use	43(25 to 69)	70(43 to 106)	0.63(0.04 to 1.52)	2.01(1.2 to 3.14)	1.6(1.02 to 2.38)	-0.2(-0.48 to 0.2)
Lao People's Democratic Republic	Liver cancer due to hepatitis B	101(63 to 150)	132(89 to 186)	0.31(-0.2 to 1.1)	4.27(2.7 to 6.3)	2.62(1.76 to 3.67)	-0.39(-0.62 to -0.05)
Lao People's Democratic Republic	Liver cancer due to hepatitis C	52(30 to 82)	69(43 to 98)	0.31(-0.11 to 0.94)	2.71(1.61 to 4.1)	1.77(1.15 to 2.48)	-0.35(-0.55 to -0.06)

Lao People's Democratic Republic	Liver cancer due to NASH	14(8 to 23)	23(14 to 34)	0.58(0.02 to 1.39)	0.69(0.4 to 1.09)	0.53(0.34 to 0.79)	-0.22(-0.48 to 0.15)
Lao People's Democratic Republic	Liver cancer due to other causes	9(5 to 14)	11(7 to 16)	0.25(-0.28 to 1.02)	0.31(0.18 to 0.5)	0.21(0.13 to 0.3)	-0.34(-0.58 to -0.01)
Latvia	Liver cancer	58(54 to 62)	101(84 to 122)	0.74(0.42 to 1.08)	1.65(1.54 to 1.77)	2.68(2.22 to 3.26)	0.62(0.32 to 0.95)
Latvia	Liver cancer due to alcohol use	21(15 to 26)	44(32 to 58)	1.12(0.68 to 1.65)	0.57(0.43 to 0.72)	1.15(0.84 to 1.52)	1.01(0.59 to 1.53)
Latvia	Liver cancer due to hepatitis B	14(10 to 19)	19(12 to 27)	0.31(0.03 to 0.65)	0.41(0.3 to 0.55)	0.56(0.38 to 0.81)	0.37(0.09 to 0.72)
Latvia	Liver cancer due to hepatitis C	16(11 to 21)	27(18 to 37)	0.71(0.36 to 1.1)	0.43(0.31 to 0.57)	0.64(0.43 to 0.89)	0.48(0.18 to 0.81)
Latvia	Liver cancer due to NASH	4(3 to 6)	8(5 to 13)	1.02(0.54 to 1.57)	0.12(0.08 to 0.16)	0.21(0.14 to 0.31)	0.79(0.4 to 1.27)
Latvia	Liver cancer due to other causes	3(2 to 5)	3(2 to 5)	0.02(-0.32 to 0.5)	0.12(0.08 to 0.19)	0.12(0.08 to 0.16)	-0.03(-0.4 to 0.65)
Lebanon	Liver cancer	79(64 to 97)	192(143 to 266)	1.43(0.69 to 2.61)	3.43(2.84 to 4.2)	3.67(2.74 to 5.09)	0.07(-0.26 to 0.58)
Lebanon	Liver cancer due to alcohol use	10(6 to 15)	23(13 to 38)	1.33(0.55 to 2.61)	0.43(0.27 to 0.65)	0.44(0.26 to 0.73)	0.01(-0.33 to 0.51)
Lebanon	Liver cancer due to hepatitis B	43(32 to 55)	92(64 to 132)	1.16(0.47 to 2.18)	1.78(1.34 to 2.28)	1.76(1.23 to 2.52)	-0.01(-0.32 to 0.45)
Lebanon	Liver cancer due to hepatitis C	16(10 to 24)	46(29 to 72)	1.85(0.84 to 3.68)	0.78(0.5 to 1.14)	0.88(0.55 to 1.38)	0.13(-0.25 to 0.79)

Lebanon	Liver cancer due to NASH	6(4 to 8)	20(13 to 32)	2.56(1.23 to 4.65)	0.26(0.17 to 0.38)	0.38(0.24 to 0.6)	0.46(-0.07 to 1.28)
Lebanon	Liver cancer due to other causes	5(3 to 6)	11(7 to 17)	1.36(0.59 to 2.52)	0.18(0.12 to 0.25)	0.21(0.14 to 0.32)	0.18(-0.2 to 0.72)
Lesotho	Liver cancer	52(32 to 110)	195(78 to 309)	2.75(-0.11 to 7.21)	5.13(3.17 to 10.64)	14.44(6.05 to 22.7)	1.81(-0.29 to 4.98)
Lesotho	Liver cancer due to alcohol use	10(4 to 24)	44(15 to 75)	3.64(-0.11 to 9.59)	0.95(0.45 to 2.32)	3.37(1.15 to 5.71)	2.55(-0.29 to 6.91)
Lesotho	Liver cancer due to hepatitis B	21(11 to 50)	84(28 to 144)	2.96(-0.24 to 8.59)	1.93(1.02 to 4.51)	5.6(1.97 to 9.55)	1.9(-0.42 to 5.8)
Lesotho	Liver cancer due to hepatitis C	14(8 to 26)	42(19 to 71)	1.92(-0.02 to 5.03)	1.58(0.94 to 2.84)	3.57(1.68 to 5.91)	1.27(-0.2 to 3.59)
Lesotho	Liver cancer due to NASH	4(2 to 8)	16(7 to 28)	3.02(0.16 to 7.66)	0.42(0.23 to 0.82)	1.3(0.56 to 2.2)	2.07(-0.05 to 5.38)
Lesotho	Liver cancer due to other causes	3(2 to 5)	9(4 to 15)	2.04(-0.12 to 5.55)	0.25(0.14 to 0.47)	0.6(0.26 to 1.04)	1.38(-0.29 to 4.07)
Liberia	Liver cancer	78(61 to 101)	107(75 to 154)	0.38(-0.05 to 0.98)	6.78(5.37 to 8.83)	4.92(3.6 to 7.24)	-0.27(-0.49 to 0.02)
Liberia	Liver cancer due to alcohol use	14(9 to 22)	18(11 to 30)	0.3(-0.11 to 0.95)	1.26(0.78 to 1.95)	0.93(0.56 to 1.51)	-0.26(-0.49 to 0.1)
Liberia	Liver cancer due to hepatitis B	40(29 to 54)	55(36 to 87)	0.37(-0.07 to 1.1)	3.44(2.51 to 4.62)	2.29(1.51 to 3.54)	-0.33(-0.54 to -0.02)
Liberia	Liver cancer due to hepatitis C	13(8 to 19)	17(10 to 26)	0.31(-0.06 to 0.86)	1.24(0.81 to 1.75)	0.96(0.57 to 1.44)	-0.23(-0.43 to 0.07)
Liberia	Liver cancer due to NASH	6(4 to 8)	10(6 to 15)	0.73(0.2 to 1.53)	0.52(0.34 to 0.77)	0.5(0.31 to	-0.04(-0.32 to 0.39)

						0.77)	
Liberia	Liver cancer due to other causes	5(3 to 7)	7(4 to 10)	0.37(-0.08 to 0.96)	0.32(0.22 to 0.45)	0.24(0.15 to 0.37)	-0.24(-0.45 to 0.06)
Libya	Liver cancer	100(76 to 130)	271(200 to 374)	1.71(0.73 to 3.08)	5.18(3.9 to 6.71)	5.18(3.88 to 7.05)	0(-0.35 to 0.51)
Libya	Liver cancer due to alcohol use	9(5 to 14)	25(15 to 40)	1.83(0.81 to 3.33)	0.48(0.29 to 0.75)	0.49(0.28 to 0.8)	0.04(-0.33 to 0.58)
Libya	Liver cancer due to hepatitis B	40(27 to 56)	105(70 to 156)	1.6(0.62 to 3.08)	1.94(1.33 to 2.71)	1.8(1.2 to 2.64)	-0.08(-0.41 to 0.43)
Libya	Liver cancer due to hepatitis C	31(21 to 46)	87(57 to 128)	1.76(0.75 to 3.14)	1.82(1.19 to 2.64)	1.83(1.2 to 2.66)	0.01(-0.35 to 0.51)
Libya	Liver cancer due to NASH	10(7 to 15)	34(23 to 54)	2.35(1.17 to 4.13)	0.57(0.36 to 0.87)	0.7(0.45 to 1.09)	0.23(-0.2 to 0.86)
Libya	Liver cancer due to other causes	9(6 to 13)	20(13 to 30)	1.13(0.35 to 2.2)	0.37(0.25 to 0.55)	0.36(0.23 to 0.55)	-0.05(-0.39 to 0.42)
Lithuania	Liver cancer	74(69 to 79)	172(136 to 211)	1.33(0.85 to 1.86)	1.66(1.56 to 1.78)	3.2(2.5 to 3.95)	0.92(0.52 to 1.37)
Lithuania	Liver cancer due to alcohol use	26(19 to 33)	79(55 to 102)	2(1.26 to 2.82)	0.58(0.43 to 0.72)	1.46(1.03 to 1.9)	1.51(0.88 to 2.2)
Lithuania	Liver cancer due to hepatitis B	18(13 to 25)	33(22 to 49)	0.79(0.38 to 1.24)	0.42(0.31 to 0.56)	0.69(0.46 to 1.01)	0.65(0.25 to 1.07)
Lithuania	Liver cancer due to hepatitis C	20(14 to 26)	43(28 to 58)	1.14(0.68 to 1.63)	0.44(0.31 to 0.57)	0.71(0.47 to 0.98)	0.63(0.28 to 1.01)
Lithuania	Liver cancer due to NASH	5(4 to 7)	13(8 to 19)	1.47(0.93 to 2.12)	0.11(0.08 to 0.16)	0.22(0.14 to 0.32)	0.94(0.5 to 1.45)

Lithuania	Liver cancer due to other causes	4(3 to 6)	5(3 to 8)	0.23(-0.18 to 0.85)	0.12(0.07 to 0.17)	0.12(0.09 to 0.17)	0.07(-0.3 to 0.74)
Luxembourg	Liver cancer	16(15 to 17)	39(30 to 51)	1.45(0.92 to 2.15)	2.95(2.73 to 3.2)	4.03(3.14 to 5.25)	0.36(0.07 to 0.76)
Luxembourg	Liver cancer due to alcohol use	7(5 to 8)	15(10 to 21)	1.34(0.75 to 2.09)	1.2(0.9 to 1.49)	1.59(1.08 to 2.22)	0.33(-0.01 to 0.76)
Luxembourg	Liver cancer due to hepatitis B	2(1 to 3)	5(3 to 7)	1.32(0.79 to 2)	0.39(0.27 to 0.56)	0.51(0.33 to 0.77)	0.31(0.01 to 0.71)
Luxembourg	Liver cancer due to hepatitis C	6(4 to 7)	15(10 to 21)	1.59(1.02 to 2.31)	1.02(0.75 to 1.32)	1.43(0.98 to 2.06)	0.4(0.1 to 0.81)
Luxembourg	Liver cancer due to NASH	1(1 to 1)	3(2 to 4)	1.78(1.16 to 2.67)	0.16(0.11 to 0.24)	0.25(0.16 to 0.38)	0.54(0.2 to 1.03)
Luxembourg	Liver cancer due to other causes	1(1 to 1)	2(1 to 3)	1.39(0.87 to 2.04)	0.18(0.14 to 0.23)	0.24(0.16 to 0.33)	0.34(0.06 to 0.68)
Madagascar	Liver cancer	164(118 to 274)	285(193 to 430)	0.74(0.23 to 1.46)	2.88(1.99 to 5.02)	2.44(1.66 to 3.72)	-0.15(-0.4 to 0.18)
Madagascar	Liver cancer due to alcohol use	36(18 to 74)	63(34 to 116)	0.76(0.21 to 1.65)	0.7(0.35 to 1.48)	0.59(0.32 to 1.07)	-0.15(-0.41 to 0.24)
Madagascar	Liver cancer due to hepatitis B	58(37 to 101)	99(60 to 164)	0.72(0.14 to 1.47)	0.96(0.58 to 1.72)	0.72(0.42 to 1.23)	-0.24(-0.48 to 0.08)
Madagascar	Liver cancer due to hepatitis C	31(19 to 50)	56(34 to 87)	0.85(0.29 to 1.62)	0.65(0.4 to 1.05)	0.61(0.39 to 0.93)	-0.06(-0.33 to 0.31)
Madagascar	Liver cancer due to NASH	17(10 to 27)	33(20 to 52)	0.97(0.36 to 1.77)	0.32(0.2 to 0.54)	0.31(0.19 to 0.49)	-0.03(-0.31 to 0.37)

Madagascar	Liver cancer due to other causes	23(14 to 37)	34(22 to 51)	0.48(0 to 1.25)	0.26(0.17 to 0.39)	0.21(0.13 to 0.31)	-0.19(-0.42 to 0.13)
Malawi	Liver cancer	160(119 to 222)	241(187 to 303)	0.5(0.07 to 1.12)	3.25(2.46 to 4.6)	2.82(2.23 to 3.54)	-0.13(-0.38 to 0.2)
Malawi	Liver cancer due to alcohol use	28(16 to 49)	49(32 to 71)	0.74(0.13 to 1.57)	0.73(0.42 to 1.26)	0.7(0.46 to 1)	-0.03(-0.36 to 0.4)
Malawi	Liver cancer due to hepatitis B	46(31 to 71)	65(44 to 91)	0.42(-0.05 to 1.09)	0.99(0.65 to 1.59)	0.74(0.5 to 1.07)	-0.25(-0.5 to 0.08)
Malawi	Liver cancer due to hepatitis C	25(16 to 39)	42(27 to 58)	0.63(0.18 to 1.29)	0.73(0.46 to 1.08)	0.68(0.45 to 0.93)	-0.08(-0.32 to 0.26)
Malawi	Liver cancer due to NASH	14(9 to 21)	25(16 to 36)	0.77(0.25 to 1.47)	0.37(0.24 to 0.56)	0.36(0.24 to 0.52)	-0.01(-0.29 to 0.36)
Malawi	Liver cancer due to other causes	47(27 to 76)	60(40 to 87)	0.28(-0.22 to 1.07)	0.43(0.29 to 0.62)	0.34(0.24 to 0.47)	-0.21(-0.47 to 0.14)
Malaysia	Liver cancer	523(448 to 612)	1641(1243 to 2134)	2.14(1.24 to 3.33)	5.51(4.7 to 6.44)	6.08(4.65 to 7.83)	0.1(-0.2 to 0.5)
Malaysia	Liver cancer due to alcohol use	65(42 to 95)	221(136 to 344)	2.42(1.36 to 3.81)	0.72(0.46 to 1.08)	0.83(0.5 to 1.28)	0.15(-0.2 to 0.61)
Malaysia	Liver cancer due to hepatitis B	321(255 to 390)	951(677 to 1261)	1.96(1.05 to 3.12)	3.21(2.52 to 3.94)	3.38(2.42 to 4.47)	0.05(-0.26 to 0.46)
Malaysia	Liver cancer due to hepatitis C	80(51 to 114)	252(159 to 378)	2.14(1.34 to 3.33)	0.97(0.62 to 1.36)	1.03(0.67 to 1.53)	0.07(-0.21 to 0.45)
Malaysia	Liver cancer due to NASH	41(29 to 59)	175(113 to 262)	3.23(2.04 to 5)	0.47(0.32 to 0.68)	0.69(0.45 to 1.01)	0.46(0.06 to 1.05)
Malaysia	Liver cancer due to other causes	16(12 to 22)	42(27 to 61)	1.55(0.84 to 2.51)	0.15(0.1 to 0.21)	0.15(0.1 to	0.04(-0.26 to 0.43)

						0.22)	
Maldives	Liver cancer	6(4 to 9)	17(13 to 21)	1.81(0.77 to 3.39)	6.8(4.73 to 10.09)	5.47(4.31 to 6.7)	-0.2(-0.47 to 0.23)
Maldives	Liver cancer due to alcohol use	1(1 to 2)	4(2 to 5)	1.97(0.83 to 3.74)	1.42(0.81 to 2.45)	1.23(0.8 to 1.79)	-0.13(-0.45 to 0.33)
Maldives	Liver cancer due to hepatitis B	3(2 to 5)	8(5 to 10)	1.58(0.58 to 3.24)	2.96(1.83 to 4.75)	2.12(1.49 to 2.89)	-0.28(-0.54 to 0.12)
Maldives	Liver cancer due to hepatitis C	1(1 to 2)	4(3 to 5)	1.95(0.81 to 3.76)	1.76(1 to 2.92)	1.44(0.95 to 2.01)	-0.18(-0.46 to 0.27)
Maldives	Liver cancer due to NASH	0(0 to 1)	1(1 to 2)	2.88(1.33 to 5.35)	0.48(0.28 to 0.83)	0.53(0.35 to 0.79)	0.1(-0.3 to 0.72)
Maldives	Liver cancer due to other causes	0(0 to 0)	1(0 to 1)	1.25(0.43 to 2.55)	0.19(0.11 to 0.33)	0.15(0.1 to 0.22)	-0.19(-0.48 to 0.24)
Mali	Liver cancer	696(571 to 838)	1358(997 to 1803)	0.95(0.41 to 1.66)	15.12(12.43 to 18.25)	14.46(10.75 to 18.98)	-0.04(-0.31 to 0.28)
Mali	Liver cancer due to alcohol use	105(64 to 156)	225(137 to 348)	1.15(0.47 to 2.05)	2.37(1.48 to 3.51)	2.49(1.51 to 3.77)	0.05(-0.29 to 0.48)
Mali	Liver cancer due to hepatitis B	286(203 to 375)	549(356 to 798)	0.92(0.32 to 1.67)	6.01(4.25 to 7.96)	5.42(3.46 to 7.81)	-0.1(-0.37 to 0.24)
Mali	Liver cancer due to hepatitis C	210(141 to 293)	409(263 to 588)	0.94(0.43 to 1.57)	5.14(3.54 to 6.98)	4.9(3.25 to 6.81)	-0.05(-0.29 to 0.24)
Mali	Liver cancer due to NASH	37(25 to 54)	90(57 to 138)	1.42(0.67 to 2.31)	0.86(0.58 to 1.26)	1(0.64 to 1.51)	0.16(-0.19 to 0.58)
Mali	Liver cancer due to other causes	58(38 to 85)	86(58 to 125)	0.49(-0.05 to 1.27)	0.73(0.51 to 1.06)	0.65(0.42 to 0.95)	-0.11(-0.38 to 0.22)

Malta	Liver cancer	7(6 to 8)	22(19 to 26)	2.22(1.63 to 2.91)	1.61(1.46 to 1.76)	2.5(2.11 to 2.95)	0.55(0.28 to 0.89)
Malta	Liver cancer due to alcohol use	2(2 to 3)	8(6 to 11)	2.59(1.83 to 3.5)	0.51(0.37 to 0.67)	0.88(0.62 to 1.18)	0.72(0.36 to 1.16)
Malta	Liver cancer due to hepatitis B	1(1 to 2)	3(2 to 4)	1.72(1.18 to 2.38)	0.24(0.17 to 0.35)	0.36(0.25 to 0.52)	0.49(0.19 to 0.86)
Malta	Liver cancer due to hepatitis C	3(2 to 3)	9(6 to 12)	2.17(1.59 to 2.91)	0.64(0.48 to 0.81)	0.91(0.66 to 1.22)	0.43(0.17 to 0.75)
Malta	Liver cancer due to NASH	0(0 to 1)	2(1 to 2)	2.79(1.94 to 3.79)	0.1(0.07 to 0.14)	0.17(0.12 to 0.25)	0.76(0.39 to 1.23)
Malta	Liver cancer due to other causes	0(0 to 1)	1(1 to 2)	1.39(0.9 to 1.97)	0.12(0.09 to 0.16)	0.18(0.13 to 0.24)	0.45(0.2 to 0.77)
Marshall Islands	Liver cancer	2(2 to 3)	4(3 to 5)	0.9(0.37 to 1.62)	11.66(8.89 to 15.25)	10.22(7.38 to 13.84)	-0.12(-0.36 to 0.2)
Marshall Islands	Liver cancer due to alcohol use	0(0 to 0)	0(0 to 1)	1.05(0.42 to 1.95)	1.37(0.79 to 2.2)	1.3(0.77 to 2.07)	-0.06(-0.33 to 0.31)
Marshall Islands	Liver cancer due to hepatitis B	1(1 to 2)	2(2 to 3)	0.9(0.32 to 1.66)	6.36(4.49 to 9.01)	5.38(3.59 to 7.84)	-0.15(-0.39 to 0.17)
Marshall Islands	Liver cancer due to hepatitis C	0(0 to 1)	1(0 to 1)	0.77(0.25 to 1.47)	2.6(1.64 to 3.93)	2.24(1.36 to 3.33)	-0.14(-0.38 to 0.16)
Marshall Islands	Liver cancer due to NASH	0(0 to 0)	0(0 to 0)	1.16(0.51 to 2.05)	0.82(0.52 to 1.28)	0.86(0.55 to 1.31)	0.04(-0.25 to 0.44)
Marshall Islands	Liver cancer due to other causes	0(0 to 0)	0(0 to 0)	0.71(0.22 to 1.4)	0.51(0.32 to 0.75)	0.44(0.27 to 0.67)	-0.13(-0.37 to 0.22)

Mauritania	Liver cancer	72(58 to 88)	86(63 to 114)	0.2(-0.15 to 0.63)	6.91(5.59 to 8.42)	4.1(3.03 to 5.29)	-0.41(-0.57 to -0.2)
Mauritania	Liver cancer due to alcohol use	10(6 to 15)	12(7 to 19)	0.25(-0.12 to 0.73)	0.98(0.61 to 1.5)	0.62(0.37 to 0.95)	-0.37(-0.55 to -0.14)
Mauritania	Liver cancer due to hepatitis B	39(29 to 50)	43(29 to 61)	0.1(-0.27 to 0.55)	3.61(2.72 to 4.65)	1.93(1.3 to 2.72)	-0.47(-0.63 to -0.27)
Mauritania	Liver cancer due to hepatitis C	13(8 to 18)	16(10 to 23)	0.23(-0.1 to 0.65)	1.35(0.84 to 1.93)	0.83(0.53 to 1.19)	-0.39(-0.54 to -0.18)
Mauritania	Liver cancer due to NASH	6(4 to 9)	10(6 to 15)	0.61(0.12 to 1.23)	0.62(0.42 to 0.91)	0.5(0.32 to 0.73)	-0.2(-0.42 to 0.08)
Mauritania	Liver cancer due to other causes	4(3 to 6)	6(3 to 8)	0.26(-0.16 to 0.8)	0.34(0.22 to 0.49)	0.22(0.14 to 0.33)	-0.35(-0.56 to -0.09)
Mauritius	Liver cancer	14(13 to 15)	33(25 to 43)	1.45(0.85 to 2.26)	1.81(1.68 to 1.94)	1.93(1.49 to 2.49)	0.07(-0.18 to 0.41)
Mauritius	Liver cancer due to alcohol use	3(2 to 4)	8(5 to 12)	1.82(1.02 to 2.98)	0.38(0.26 to 0.52)	0.45(0.29 to 0.67)	0.17(-0.14 to 0.66)
Mauritius	Liver cancer due to hepatitis B	6(4 to 7)	11(8 to 16)	1.06(0.5 to 1.81)	0.7(0.53 to 0.88)	0.65(0.44 to 0.93)	-0.06(-0.31 to 0.27)
Mauritius	Liver cancer due to hepatitis C	3(2 to 4)	9(6 to 13)	1.67(1.04 to 2.47)	0.5(0.36 to 0.64)	0.54(0.35 to 0.77)	0.08(-0.17 to 0.39)
Mauritius	Liver cancer due to NASH	1(1 to 2)	4(2 to 6)	2.23(1.4 to 3.28)	0.16(0.11 to 0.23)	0.23(0.15 to 0.34)	0.37(0.03 to 0.83)
Mauritius	Liver cancer due to other causes	1(0 to 1)	1(1 to 1)	0.63(0.17 to 1.17)	0.07(0.05 to 0.09)	0.07(0.05 to 0.09)	-0.02(-0.25 to 0.28)

Mexico	Liver cancer	970(935 to 997)	3973(3436 to 4550)	3.1(2.54 to 3.71)	2.24(2.14 to 2.3)	3.46(2.99 to 3.96)	0.55(0.33 to 0.77)
Mexico	Liver cancer due to alcohol use	250(217 to 284)	1223(994 to 1483)	3.89(3.17 to 4.7)	0.6(0.52 to 0.68)	1.06(0.86 to 1.28)	0.77(0.51 to 1.06)
Mexico	Liver cancer due to hepatitis B	142(121 to 168)	480(378 to 610)	2.37(1.88 to 2.9)	0.3(0.25 to 0.35)	0.4(0.31 to 0.51)	0.34(0.15 to 0.54)
Mexico	Liver cancer due to hepatitis C	383(344 to 420)	1595(1337 to 1895)	3.17(2.61 to 3.79)	0.98(0.88 to 1.07)	1.42(1.19 to 1.68)	0.45(0.26 to 0.66)
Mexico	Liver cancer due to NASH	78(68 to 91)	404(324 to 493)	4.16(3.43 to 4.96)	0.19(0.16 to 0.22)	0.35(0.28 to 0.43)	0.9(0.64 to 1.19)
Mexico	Liver cancer due to other causes	117(104 to 134)	271(225 to 322)	1.33(0.94 to 1.74)	0.18(0.16 to 0.2)	0.23(0.19 to 0.27)	0.3(0.11 to 0.48)
Micronesia (Federated States of)	Liver cancer	5(4 to 7)	8(5 to 12)	0.44(-0.08 to 1.15)	11.16(8.3 to 14.48)	10.37(6.93 to 14.51)	-0.07(-0.39 to 0.35)
Micronesia (Federated States of)	Liver cancer due to alcohol use	1(0 to 1)	1(1 to 2)	0.49(-0.08 to 1.27)	1.34(0.78 to 2.14)	1.29(0.72 to 2.03)	-0.04(-0.38 to 0.44)
Micronesia (Federated States of)	Liver cancer due to hepatitis B	3(2 to 4)	4(3 to 7)	0.43(-0.13 to 1.18)	5.93(4.06 to 8.21)	5.35(3.33 to 7.86)	-0.1(-0.44 to 0.35)
Micronesia (Federated States of)	Liver cancer due to hepatitis C	1(1 to 2)	1(1 to 2)	0.36(-0.12 to 1.03)	2.5(1.56 to 3.74)	2.29(1.37 to 3.46)	-0.09(-0.38 to 0.34)
Micronesia (Federated States of)	Liver cancer due to NASH	0(0 to 1)	1(0 to 1)	0.68(0.08 to 1.59)	0.89(0.56 to 1.39)	1.01(0.6 to 1.56)	0.13(-0.24 to 0.73)

Micronesia (Federated States of)	Liver cancer due to other causes	0(0 to 0)	0(0 to 1)	0.27(-0.19 to 0.87)	0.49(0.3 to 0.73)	0.44(0.25 to 0.73)	-0.1(-0.41 to 0.36)
Monaco	Liver cancer	2(2 to 3)	8(6 to 10)	2.49(1.61 to 3.67)	3.47(2.71 to 4.29)	9.04(7.13 to 11.28)	1.61(0.93 to 2.55)
Monaco	Liver cancer due to alcohol use	1(1 to 1)	3(2 to 4)	2.59(1.67 to 3.85)	1.18(0.79 to 1.64)	3.2(2.19 to 4.44)	1.7(0.97 to 2.71)
Monaco	Liver cancer due to hepatitis B	0(0 to 0)	1(1 to 1)	2.16(1.3 to 3.4)	0.56(0.36 to 0.83)	1.35(0.88 to 1.97)	1.41(0.75 to 2.42)
Monaco	Liver cancer due to hepatitis C	1(1 to 1)	3(2 to 4)	2.47(1.58 to 3.75)	1.26(0.85 to 1.74)	3.28(2.27 to 4.41)	1.61(0.91 to 2.55)
Monaco	Liver cancer due to NASH	0(0 to 0)	1(0 to 1)	2.88(1.81 to 4.38)	0.24(0.15 to 0.37)	0.72(0.47 to 1.1)	1.96(1.16 to 3.08)
Monaco	Liver cancer due to other causes	0(0 to 0)	0(0 to 1)	2.32(1.45 to 3.53)	0.23(0.16 to 0.31)	0.5(0.34 to 0.71)	1.2(0.65 to 1.92)
Mongolia	Liver cancer	680(544 to 827)	2311(1767 to 2994)	2.4(1.47 to 3.73)	64.22(51.72 to 77.51)	105.22(82.57 to 131.46)	0.64(0.22 to 1.21)
Mongolia	Liver cancer due to alcohol use	179(112 to 256)	714(468 to 1046)	2.99(1.79 to 4.54)	16.95(10.71 to 23.76)	31.82(21.31 to 44.72)	0.88(0.34 to 1.6)
Mongolia	Liver cancer due to hepatitis B	239(167 to 330)	715(470 to 1044)	1.99(1.1 to 3.26)	21.55(14.91 to 29.84)	27.28(18.01 to 39.1)	0.27(-0.08 to 0.76)
Mongolia	Liver cancer due to hepatitis C	206(138 to 288)	657(435 to 925)	2.19(1.35 to 3.41)	20.42(13.9 to 28.35)	35.02(24.73 to 46.77)	0.71(0.28 to 1.35)
Mongolia	Liver cancer due to NASH	33(21 to 50)	146(93 to 220)	3.38(2.14 to 5.06)	3.27(2.12 to 4.9)	7.62(4.88 to 11.42)	1.33(0.69 to 2.21)

Mongolia	Liver cancer due to other causes	23(15 to 34)	79(51 to 121)	2.47(1.43 to 3.96)	2.03(1.31 to 3.09)	3.47(2.19 to 5.27)	0.71(0.2 to 1.42)
Montenegro	Liver cancer	38(31 to 44)	58(47 to 72)	0.54(0.17 to 1.16)	6.05(4.99 to 7)	5.97(4.82 to 7.31)	-0.01(-0.25 to 0.39)
Montenegro	Liver cancer due to alcohol use	16(12 to 21)	25(18 to 34)	0.55(0.13 to 1.25)	2.55(1.88 to 3.3)	2.48(1.76 to 3.34)	-0.03(-0.29 to 0.39)
Montenegro	Liver cancer due to hepatitis B	9(6 to 13)	12(8 to 18)	0.33(-0.01 to 0.85)	1.44(1.01 to 2.03)	1.31(0.88 to 1.9)	-0.09(-0.31 to 0.26)
Montenegro	Liver cancer due to hepatitis C	9(6 to 12)	14(10 to 21)	0.69(0.27 to 1.35)	1.4(0.94 to 1.92)	1.46(0.99 to 2.05)	0.04(-0.22 to 0.45)
Montenegro	Liver cancer due to NASH	3(2 to 4)	5(3 to 7)	0.87(0.37 to 1.65)	0.42(0.28 to 0.6)	0.49(0.34 to 0.72)	0.18(-0.13 to 0.66)
Montenegro	Liver cancer due to other causes	1(1 to 2)	2(1 to 3)	0.3(-0.05 to 0.77)	0.24(0.17 to 0.33)	0.22(0.15 to 0.32)	-0.07(-0.29 to 0.24)
Morocco	Liver cancer	249(183 to 307)	632(481 to 778)	1.54(0.81 to 2.53)	1.92(1.38 to 2.37)	2.16(1.65 to 2.63)	0.13(-0.19 to 0.55)
Morocco	Liver cancer due to alcohol use	27(16 to 42)	73(44 to 112)	1.69(0.87 to 2.89)	0.21(0.12 to 0.32)	0.25(0.15 to 0.38)	0.19(-0.18 to 0.71)
Morocco	Liver cancer due to hepatitis B	100(70 to 138)	230(154 to 327)	1.3(0.58 to 2.35)	0.71(0.49 to 0.99)	0.72(0.48 to 1.02)	0.01(-0.29 to 0.44)
Morocco	Liver cancer due to hepatitis C	81(53 to 114)	216(144 to 297)	1.66(0.94 to 2.72)	0.7(0.46 to 0.95)	0.8(0.53 to 1.08)	0.14(-0.16 to 0.57)
Morocco	Liver cancer due to NASH	22(14 to 33)	72(47 to 108)	2.3(1.28 to 3.7)	0.18(0.11 to 0.27)	0.26(0.17 to 0.38)	0.45(0.02 to 1.02)

Morocco	Liver cancer due to other causes	19(13 to 26)	40(26 to 59)	1.16(0.55 to 2.01)	0.12(0.08 to 0.17)	0.13(0.09 to 0.19)	0.09(-0.21 to 0.51)
Mozambique	Liver cancer	133(94 to 214)	441(303 to 585)	2.32(0.52 to 4.31)	2.1(1.51 to 3.39)	3.67(2.55 to 4.85)	0.75(-0.2 to 1.82)
Mozambique	Liver cancer due to alcohol use	25(14 to 48)	95(54 to 139)	2.82(0.46 to 5.71)	0.44(0.25 to 0.86)	0.91(0.52 to 1.33)	1.08(-0.21 to 2.61)
Mozambique	Liver cancer due to hepatitis B	56(35 to 99)	186(117 to 266)	2.35(0.37 to 4.73)	0.84(0.52 to 1.47)	1.44(0.91 to 2.07)	0.72(-0.32 to 1.9)
Mozambique	Liver cancer due to hepatitis C	20(12 to 32)	55(33 to 82)	1.75(0.49 to 3.31)	0.41(0.25 to 0.66)	0.62(0.38 to 0.92)	0.51(-0.17 to 1.34)
Mozambique	Liver cancer due to NASH	13(8 to 20)	42(27 to 63)	2.32(0.79 to 4.25)	0.24(0.15 to 0.37)	0.42(0.27 to 0.63)	0.77(-0.04 to 1.77)
Mozambique	Liver cancer due to other causes	20(12 to 32)	62(38 to 97)	2.17(0.7 to 4.61)	0.18(0.11 to 0.27)	0.28(0.19 to 0.41)	0.59(-0.09 to 1.45)
Myanmar	Liver cancer	643(458 to 925)	1868(1543 to 2237)	1.9(0.98 to 3.23)	2.78(2.01 to 3.95)	4.11(3.46 to 4.88)	0.48(0.04 to 1.09)
Myanmar	Liver cancer due to alcohol use	111(67 to 188)	376(241 to 549)	2.4(1.24 to 4.09)	0.49(0.3 to 0.81)	0.83(0.54 to 1.19)	0.68(0.14 to 1.48)
Myanmar	Liver cancer due to hepatitis B	249(156 to 382)	649(460 to 901)	1.61(0.73 to 2.89)	1(0.63 to 1.54)	1.32(0.93 to 1.84)	0.32(-0.1 to 0.92)
Myanmar	Liver cancer due to hepatitis C	182(108 to 291)	548(373 to 745)	2.01(1.1 to 3.4)	0.9(0.57 to 1.39)	1.3(0.9 to 1.73)	0.44(0.04 to 1.1)
Myanmar	Liver cancer due to NASH	53(32 to 85)	194(130 to 283)	2.65(1.49 to 4.41)	0.25(0.15 to 0.4)	0.44(0.3 to 0.64)	0.8(0.25 to 1.61)
Myanmar	Liver cancer due to other causes	49(29 to 84)	103(69 to 151)	1.1(0.16 to 2.48)	0.13(0.09 to 0.22)	0.21(0.14 to	0.55(-0.07 to 1.41)

						0.3)	
Namibia	Liver cancer	14(8 to 25)	48(36 to 63)	2.48(0.86 to 5.24)	1.86(1.16 to 3.3)	3.28(2.5 to 4.2)	0.76(-0.04 to 2.06)
Namibia	Liver cancer due to alcohol use	3(1 to 6)	11(7 to 17)	3.37(1.11 to 7.29)	0.36(0.16 to 0.8)	0.8(0.51 to 1.15)	1.26(0.1 to 3.32)
Namibia	Liver cancer due to hepatitis B	5(3 to 10)	17(11 to 25)	2.46(0.65 to 6)	0.64(0.33 to 1.31)	1.07(0.71 to 1.55)	0.69(-0.18 to 2.3)
Namibia	Liver cancer due to hepatitis C	4(2 to 7)	12(8 to 17)	2.02(0.85 to 3.9)	0.61(0.38 to 0.99)	0.94(0.63 to 1.3)	0.56(-0.03 to 1.46)
Namibia	Liver cancer due to NASH	1(1 to 2)	4(3 to 6)	2.57(1.07 to 5)	0.16(0.09 to 0.29)	0.3(0.19 to 0.44)	0.83(0.08 to 2.01)
Namibia	Liver cancer due to other causes	1(1 to 2)	3(2 to 4)	1.98(0.78 to 4.02)	0.1(0.06 to 0.18)	0.16(0.11 to 0.24)	0.61(-0.05 to 1.69)
Nauru	Liver cancer	0(0 to 1)	0(0 to 1)	0.01(-0.27 to 0.43)	10.54(7.58 to 13.38)	9.17(6.5 to 12.59)	-0.13(-0.36 to 0.19)
Nauru	Liver cancer due to alcohol use	0(0 to 0)	0(0 to 0)	0.08(-0.27 to 0.6)	1.26(0.75 to 1.93)	1.2(0.68 to 1.85)	-0.05(-0.33 to 0.34)
Nauru	Liver cancer due to hepatitis B	0(0 to 0)	0(0 to 0)	-0.02(-0.31 to 0.43)	5.66(3.81 to 7.69)	4.66(3.12 to 6.77)	-0.18(-0.41 to 0.17)
Nauru	Liver cancer due to hepatitis C	0(0 to 0)	0(0 to 0)	-0.05(-0.33 to 0.32)	2.29(1.47 to 3.27)	1.99(1.24 to 3.04)	-0.13(-0.35 to 0.16)
Nauru	Liver cancer due to NASH	0(0 to 0)	0(0 to 0)	0.18(-0.16 to 0.66)	0.85(0.55 to 1.25)	0.89(0.57 to 1.38)	0.05(-0.23 to 0.44)
Nauru	Liver cancer due to other causes	0(0 to 0)	0(0 to 0)	0.03(-0.24 to 0.37)	0.47(0.3 to 0.71)	0.43(0.26 to 0.69)	-0.09(-0.34 to 0.22)

Nepal	Liver cancer	179(137 to 232)	460(331 to 662)	1.57(0.71 to 2.57)	1.8(1.4 to 2.27)	2.13(1.54 to 3.05)	0.18(-0.2 to 0.63)
Nepal	Liver cancer due to alcohol use	41(28 to 59)	125(74 to 208)	2.04(0.91 to 3.44)	0.46(0.31 to 0.67)	0.58(0.34 to 0.95)	0.25(-0.22 to 0.85)
Nepal	Liver cancer due to hepatitis B	50(33 to 71)	110(68 to 173)	1.21(0.41 to 2.13)	0.46(0.31 to 0.65)	0.47(0.29 to 0.74)	0.02(-0.35 to 0.43)
Nepal	Liver cancer due to hepatitis C	49(32 to 72)	148(98 to 232)	2.03(1.06 to 3.19)	0.61(0.41 to 0.87)	0.75(0.51 to 1.14)	0.23(-0.16 to 0.7)
Nepal	Liver cancer due to NASH	13(9 to 20)	45(28 to 68)	2.41(1.33 to 3.88)	0.15(0.1 to 0.22)	0.21(0.13 to 0.33)	0.46(0 to 1.04)
Nepal	Liver cancer due to other causes	26(16 to 44)	32(22 to 47)	0.24(-0.28 to 1.18)	0.12(0.08 to 0.18)	0.12(0.08 to 0.17)	0(-0.34 to 0.53)
Netherlands	Liver cancer	284(269 to 298)	1074(836 to 1350)	2.78(1.96 to 3.75)	1.46(1.39 to 1.54)	3.33(2.58 to 4.19)	1.27(0.77 to 1.87)
Netherlands	Liver cancer due to alcohol use	115(91 to 140)	440(312 to 597)	2.82(1.94 to 3.89)	0.58(0.46 to 0.7)	1.33(0.94 to 1.82)	1.3(0.77 to 1.94)
Netherlands	Liver cancer due to hepatitis B	38(27 to 53)	132(84 to 203)	2.48(1.62 to 3.47)	0.2(0.15 to 0.28)	0.47(0.31 to 0.7)	1.29(0.74 to 1.98)
Netherlands	Liver cancer due to hepatitis C	91(68 to 117)	355(247 to 495)	2.9(2.07 to 3.87)	0.45(0.34 to 0.58)	1.03(0.71 to 1.43)	1.26(0.77 to 1.85)
Netherlands	Liver cancer due to NASH	20(14 to 29)	88(56 to 132)	3.4(2.35 to 4.67)	0.1(0.07 to 0.14)	0.26(0.17 to 0.39)	1.64(1.01 to 2.44)
Netherlands	Liver cancer due to other causes	20(15 to 25)	59(38 to 84)	2.01(1.22 to 2.85)	0.13(0.1 to 0.16)	0.24(0.17 to 0.32)	0.89(0.44 to 1.4)

New Zealand	Liver cancer	92(85 to 99)	361(297 to 437)	2.92(2.2 to 3.78)	2.41(2.24 to 2.59)	4.96(4.09 to 6.02)	1.05(0.67 to 1.51)
New Zealand	Liver cancer due to alcohol use	38(32 to 42)	138(107 to 176)	2.66(1.88 to 3.58)	0.98(0.84 to 1.1)	1.88(1.46 to 2.39)	0.92(0.5 to 1.42)
New Zealand	Liver cancer due to hepatitis B	17(14 to 20)	58(43 to 75)	2.41(1.7 to 3.29)	0.45(0.38 to 0.54)	0.88(0.66 to 1.14)	0.93(0.52 to 1.42)
New Zealand	Liver cancer due to hepatitis C	25(21 to 28)	108(87 to 134)	3.42(2.63 to 4.31)	0.63(0.55 to 0.73)	1.39(1.12 to 1.72)	1.21(0.82 to 1.66)
New Zealand	Liver cancer due to NASH	7(6 to 9)	39(31 to 49)	4.35(3.43 to 5.47)	0.19(0.16 to 0.22)	0.52(0.41 to 0.65)	1.76(1.29 to 2.36)
New Zealand	Liver cancer due to other causes	6(5 to 6)	18(15 to 22)	2.16(1.63 to 2.75)	0.16(0.14 to 0.18)	0.29(0.24 to 0.35)	0.8(0.51 to 1.12)
Nicaragua	Liver cancer	50(44 to 56)	165(133 to 202)	2.3(1.58 to 3.17)	3.04(2.65 to 3.48)	3.79(3.09 to 4.63)	0.24(-0.04 to 0.58)
Nicaragua	Liver cancer due to alcohol use	13(9 to 18)	51(34 to 71)	2.88(1.89 to 4.27)	0.86(0.58 to 1.15)	1.17(0.79 to 1.64)	0.36(0.01 to 0.85)
Nicaragua	Liver cancer due to hepatitis B	10(7 to 14)	26(17 to 38)	1.55(0.91 to 2.34)	0.55(0.39 to 0.79)	0.54(0.35 to 0.79)	-0.02(-0.26 to 0.27)
Nicaragua	Liver cancer due to hepatitis C	18(13 to 23)	62(44 to 84)	2.53(1.75 to 3.43)	1.22(0.89 to 1.56)	1.52(1.1 to 2.02)	0.25(-0.03 to 0.58)
Nicaragua	Liver cancer due to NASH	3(2 to 4)	14(9 to 20)	3.26(2.23 to 4.5)	0.2(0.14 to 0.29)	0.32(0.21 to 0.46)	0.61(0.23 to 1.09)
Nicaragua	Liver cancer due to other causes	6(5 to 7)	12(8 to 16)	1.03(0.53 to 1.6)	0.21(0.16 to 0.28)	0.23(0.16 to 0.31)	0.06(-0.18 to 0.36)

Niger	Liver cancer	20(16 to 25)	51(37 to 68)	1.54(0.82 to 2.44)	0.67(0.52 to 0.83)	0.61(0.45 to 0.8)	-0.09(-0.34 to 0.21)
Niger	Liver cancer due to alcohol use	3(2 to 4)	7(4 to 11)	1.6(0.81 to 2.7)	0.1(0.06 to 0.16)	0.1(0.06 to 0.15)	-0.06(-0.33 to 0.34)
Niger	Liver cancer due to hepatitis B	11(8 to 15)	27(18 to 38)	1.38(0.68 to 2.35)	0.36(0.26 to 0.47)	0.3(0.2 to 0.42)	-0.15(-0.38 to 0.16)
Niger	Liver cancer due to hepatitis C	3(2 to 5)	9(5 to 13)	1.7(1 to 2.62)	0.13(0.09 to 0.19)	0.13(0.08 to 0.18)	-0.04(-0.27 to 0.27)
Niger	Liver cancer due to NASH	1(1 to 2)	4(2 to 5)	1.98(1.12 to 3.04)	0.04(0.03 to 0.07)	0.05(0.03 to 0.07)	0.06(-0.23 to 0.43)
Niger	Liver cancer due to other causes	1(1 to 2)	4(3 to 6)	2.04(1.14 to 3.28)	0.03(0.02 to 0.04)	0.03(0.02 to 0.05)	0.03(-0.25 to 0.38)
Nigeria	Liver cancer	1559(1184 to 1976)	2786(2157 to 3527)	0.79(0.3 to 1.46)	3.3(2.46 to 4.16)	3.25(2.56 to 4.04)	-0.02(-0.28 to 0.36)
Nigeria	Liver cancer due to alcohol use	279(196 to 385)	575(429 to 760)	1.06(0.45 to 1.89)	0.65(0.46 to 0.89)	0.73(0.55 to 0.95)	0.13(-0.19 to 0.6)
Nigeria	Liver cancer due to hepatitis B	651(469 to 859)	1119(829 to 1493)	0.72(0.23 to 1.41)	1.34(0.96 to 1.79)	1.23(0.91 to 1.62)	-0.09(-0.34 to 0.29)
Nigeria	Liver cancer due to hepatitis C	307(216 to 419)	558(421 to 718)	0.82(0.31 to 1.56)	0.78(0.56 to 1.06)	0.75(0.58 to 0.95)	-0.04(-0.3 to 0.36)
Nigeria	Liver cancer due to NASH	125(90 to 168)	269(201 to 346)	1.15(0.54 to 1.98)	0.3(0.22 to 0.4)	0.34(0.26 to 0.43)	0.16(-0.16 to 0.62)
Nigeria	Liver cancer due to other causes	197(151 to 249)	265(174 to 363)	0.35(-0.14 to 0.9)	0.23(0.18 to 0.29)	0.19(0.14 to 0.25)	-0.16(-0.39 to 0.13)
Niue	Liver cancer	0(0 to 0)	0(0 to 0)	-0.09(-0.33 to 0.25)	8.1(6.43 to 10.14)	7.37(5.65 to	-0.09(-0.34 to 0.25)

						9.46)	
Niue	Liver cancer due to alcohol use	0(0 to 0)	0(0 to 0)	0.06(-0.26 to 0.52)	0.99(0.61 to 1.48)	1.02(0.64 to 1.54)	0.03(-0.28 to 0.47)
Niue	Liver cancer due to hepatitis B	0(0 to 0)	0(0 to 0)	-0.11(-0.37 to 0.26)	4.25(3.08 to 5.58)	3.69(2.61 to 5.06)	-0.13(-0.4 to 0.23)
Niue	Liver cancer due to hepatitis C	0(0 to 0)	0(0 to 0)	-0.17(-0.4 to 0.12)	1.81(1.21 to 2.58)	1.55(1.01 to 2.25)	-0.14(-0.37 to 0.16)
Niue	Liver cancer due to NASH	0(0 to 0)	0(0 to 0)	0.12(-0.19 to 0.55)	0.68(0.46 to 1.02)	0.79(0.51 to 1.15)	0.16(-0.16 to 0.6)
Niue	Liver cancer due to other causes	0(0 to 0)	0(0 to 0)	-0.19(-0.43 to 0.14)	0.37(0.24 to 0.53)	0.32(0.2 to 0.48)	-0.12(-0.39 to 0.26)
North Macedonia	Liver cancer	163(146 to 179)	270(213 to 344)	0.66(0.27 to 1.14)	8.65(7.72 to 9.49)	8.39(6.65 to 10.65)	-0.03(-0.25 to 0.24)
North Macedonia	Liver cancer due to alcohol use	59(43 to 76)	102(68 to 143)	0.72(0.27 to 1.25)	3.13(2.31 to 4)	3.06(2.07 to 4.31)	-0.02(-0.27 to 0.27)
North Macedonia	Liver cancer due to hepatitis B	44(32 to 61)	63(41 to 95)	0.42(0.07 to 0.87)	2.25(1.6 to 3.06)	1.96(1.3 to 2.89)	-0.13(-0.34 to 0.14)
North Macedonia	Liver cancer due to hepatitis C	41(28 to 56)	73(48 to 105)	0.76(0.35 to 1.29)	2.3(1.6 to 3.07)	2.32(1.56 to 3.27)	0.01(-0.22 to 0.3)
North Macedonia	Liver cancer due to NASH	11(8 to 16)	23(15 to 34)	1.06(0.56 to 1.73)	0.62(0.43 to 0.87)	0.73(0.48 to 1.07)	0.19(-0.11 to 0.59)
North Macedonia	Liver cancer due to other causes	7(5 to 9)	10(6 to 15)	0.41(0.05 to 0.88)	0.35(0.26 to 0.48)	0.32(0.21 to 0.47)	-0.09(-0.3 to 0.18)
Northern Mariana	Liver cancer	1(1 to 2)	5(4 to 6)	2.08(1.23 to 3.22)	6.8(5.35 to 8.4)	7.97(6.58 to	0.17(-0.09 to 0.52)

Islands						9.7)	
Northern Mariana Islands	Liver cancer due to alcohol use	0(0 to 0)	1(0 to 1)	2.87(1.68 to 4.47)	0.75(0.46 to 1.12)	0.98(0.63 to 1.43)	0.3(-0.02 to 0.72)
Northern Mariana Islands	Liver cancer due to hepatitis B	1(1 to 1)	3(2 to 4)	1.96(1.06 to 3.23)	3.59(2.59 to 4.74)	4.47(3.39 to 5.79)	0.25(-0.09 to 0.66)
Northern Mariana Islands	Liver cancer due to hepatitis C	0(0 to 0)	1(0 to 1)	2.3(1.44 to 3.41)	1.5(0.99 to 2.08)	1.45(0.93 to 2.02)	-0.03(-0.24 to 0.22)
Northern Mariana Islands	Liver cancer due to NASH	0(0 to 0)	0(0 to 1)	2.34(1.37 to 3.65)	0.67(0.46 to 0.96)	0.78(0.53 to 1.13)	0.18(-0.1 to 0.53)
Northern Mariana Islands	Liver cancer due to other causes	0(0 to 0)	0(0 to 0)	0.97(0.32 to 1.8)	0.3(0.2 to 0.43)	0.28(0.19 to 0.4)	-0.06(-0.28 to 0.23)
Norway	Liver cancer	105(100 to 110)	262(218 to 316)	1.48(1.05 to 2.01)	1.64(1.57 to 1.71)	2.91(2.42 to 3.51)	0.77(0.46 to 1.15)
Norway	Liver cancer due to alcohol use	33(29 to 38)	90(70 to 113)	1.7(1.15 to 2.39)	0.51(0.45 to 0.58)	0.99(0.76 to 1.25)	0.94(0.53 to 1.43)
Norway	Liver cancer due to hepatitis B	16(14 to 19)	36(28 to 47)	1.23(0.81 to 1.73)	0.28(0.24 to 0.33)	0.45(0.35 to 0.58)	0.59(0.28 to 0.95)
Norway	Liver cancer due to hepatitis C	43(38 to 48)	104(84 to 126)	1.43(1.03 to 1.87)	0.62(0.55 to 0.69)	1.08(0.87 to 1.32)	0.74(0.46 to 1.06)
Norway	Liver cancer due to NASH	7(6 to 8)	18(14 to 22)	1.67(1.21 to 2.15)	0.1(0.08 to 0.11)	0.19(0.15 to 0.24)	0.96(0.63 to 1.32)
Norway	Liver cancer due to other causes	7(6 to 8)	15(12 to 18)	1.17(0.84 to 1.56)	0.13(0.12 to 0.15)	0.2(0.17 to 0.25)	0.5(0.28 to 0.77)
Oman	Liver cancer	31(22 to 41)	98(79 to 122)	2.19(1.25 to 3.62)	4.25(2.94 to 5.65)	5.27(4.47 to	0.24(-0.12 to 0.85)

						6.3)	
Oman	Liver cancer due to alcohol use	3(2 to 4)	9(6 to 15)	2.35(1.27 to 4.05)	0.41(0.24 to 0.65)	0.54(0.34 to 0.84)	0.31(-0.12 to 1.07)
Oman	Liver cancer due to hepatitis B	13(8 to 20)	42(29 to 58)	2.14(1.15 to 3.8)	1.63(1.02 to 2.39)	1.76(1.26 to 2.47)	0.08(-0.26 to 0.63)
Oman	Liver cancer due to hepatitis C	10(6 to 14)	27(18 to 37)	1.87(0.99 to 3.23)	1.62(1.04 to 2.33)	1.93(1.35 to 2.54)	0.19(-0.18 to 0.79)
Oman	Liver cancer due to NASH	2(1 to 3)	11(7 to 15)	4.17(2.54 to 6.85)	0.32(0.18 to 0.52)	0.66(0.45 to 0.97)	1.07(0.39 to 2.16)
Oman	Liver cancer due to other causes	3(2 to 4)	9(7 to 12)	1.93(1.18 to 3.02)	0.27(0.18 to 0.39)	0.37(0.27 to 0.51)	0.38(0 to 0.94)
Pakistan	Liver cancer	1976(1345 to 2622)	3884(3101 to 4736)	0.97(0.42 to 1.87)	3.27(2.16 to 4.42)	3.27(2.56 to 4.02)	0(-0.27 to 0.46)
Pakistan	Liver cancer due to alcohol use	303(189 to 424)	616(457 to 833)	1.03(0.41 to 2.23)	0.52(0.32 to 0.72)	0.52(0.39 to 0.7)	0.01(-0.29 to 0.59)
Pakistan	Liver cancer due to hepatitis B	333(220 to 463)	725(555 to 944)	1.18(0.51 to 2.37)	0.51(0.33 to 0.72)	0.51(0.39 to 0.67)	0.01(-0.3 to 0.56)
Pakistan	Liver cancer due to hepatitis C	1034(662 to 1434)	1869(1400 to 2382)	0.81(0.29 to 1.66)	1.86(1.18 to 2.58)	1.79(1.31 to 2.24)	-0.04(-0.3 to 0.43)
Pakistan	Liver cancer due to NASH	141(93 to 199)	319(240 to 409)	1.27(0.59 to 2.4)	0.23(0.15 to 0.34)	0.27(0.2 to 0.35)	0.16(-0.17 to 0.72)
Pakistan	Liver cancer due to other causes	165(125 to 216)	355(272 to 468)	1.15(0.54 to 2.1)	0.16(0.12 to 0.21)	0.18(0.14 to 0.23)	0.12(-0.17 to 0.59)
Palau	Liver cancer	1(1 to 1)	2(2 to 3)	1.31(0.52 to 2.55)	9.64(6.66 to 13.3)	9.97(7.68 to	0.03(-0.31 to 0.57)

						12.94)	
Palau	Liver cancer due to alcohol use	0(0 to 0)	0(0 to 0)	1.5(0.63 to 2.96)	1.25(0.7 to 2.06)	1.36(0.84 to 2.06)	0.09(-0.29 to 0.7)
Palau	Liver cancer due to hepatitis B	1(0 to 1)	1(1 to 2)	1.25(0.45 to 2.53)	5.82(3.75 to 8.3)	5.84(4.24 to 7.86)	0(-0.34 to 0.56)
Palau	Liver cancer due to hepatitis C	0(0 to 0)	0(0 to 0)	1.22(0.48 to 2.39)	1.59(0.96 to 2.41)	1.59(1.03 to 2.27)	0(-0.32 to 0.48)
Palau	Liver cancer due to NASH	0(0 to 0)	0(0 to 0)	1.91(0.92 to 3.52)	0.64(0.38 to 1.02)	0.86(0.54 to 1.28)	0.33(-0.1 to 1)
Palau	Liver cancer due to other causes	0(0 to 0)	0(0 to 0)	1.01(0.31 to 2)	0.33(0.2 to 0.51)	0.32(0.21 to 0.49)	-0.01(-0.35 to 0.49)
Palestine	Liver cancer	72(53 to 96)	149(125 to 180)	1.07(0.48 to 1.91)	8.42(6.28 to 11.14)	6.56(5.52 to 7.88)	-0.22(-0.45 to 0.09)
Palestine	Liver cancer due to alcohol use	6(3 to 9)	13(8 to 20)	1.19(0.52 to 2.19)	0.73(0.42 to 1.12)	0.6(0.37 to 0.9)	-0.18(-0.43 to 0.19)
Palestine	Liver cancer due to hepatitis B	25(17 to 37)	54(40 to 72)	1.16(0.53 to 2.09)	2.74(1.85 to 3.99)	2.06(1.49 to 2.81)	-0.25(-0.47 to 0.07)
Palestine	Liver cancer due to hepatitis C	28(19 to 40)	55(39 to 73)	0.95(0.34 to 1.75)	3.61(2.47 to 5)	2.79(2.04 to 3.63)	-0.23(-0.47 to 0.09)
Palestine	Liver cancer due to NASH	6(4 to 10)	15(10 to 21)	1.28(0.56 to 2.29)	0.78(0.47 to 1.22)	0.69(0.47 to 1)	-0.11(-0.4 to 0.26)
Palestine	Liver cancer due to other causes	6(4 to 9)	11(8 to 16)	0.92(0.33 to 1.76)	0.56(0.35 to 0.86)	0.41(0.29 to 0.58)	-0.26(-0.49 to 0.07)
Panama	Liver cancer	50(46 to 54)	121(92 to 155)	1.42(0.83 to 2.2)	3.27(3.01 to 3.52)	2.93(2.23 to 3.77)	-0.1(-0.32 to 0.18)

Panama	Liver cancer due to alcohol use	15(11 to 20)	42(28 to 61)	1.75(1.04 to 2.67)	1.02(0.75 to 1.31)	1.02(0.68 to 1.47)	0(-0.26 to 0.34)
Panama	Liver cancer due to hepatitis B	10(7 to 13)	19(12 to 28)	0.92(0.4 to 1.61)	0.6(0.42 to 0.83)	0.45(0.29 to 0.68)	-0.24(-0.45 to 0.02)
Panama	Liver cancer due to hepatitis C	18(13 to 23)	43(29 to 60)	1.4(0.81 to 2.16)	1.23(0.92 to 1.57)	1.04(0.7 to 1.48)	-0.16(-0.37 to 0.11)
Panama	Liver cancer due to NASH	3(2 to 5)	10(6 to 15)	2.07(1.26 to 3.12)	0.21(0.15 to 0.3)	0.24(0.15 to 0.36)	0.13(-0.16 to 0.51)
Panama	Liver cancer due to other causes	4(3 to 5)	7(5 to 10)	0.88(0.41 to 1.46)	0.21(0.16 to 0.29)	0.18(0.12 to 0.25)	-0.16(-0.37 to 0.12)
Papua New Guinea	Liver cancer	25(19 to 32)	65(50 to 85)	1.64(0.92 to 2.66)	1.4(1.09 to 1.78)	1.53(1.19 to 1.96)	0.09(-0.21 to 0.48)
Papua New Guinea	Liver cancer due to alcohol use	3(2 to 5)	9(5 to 14)	1.88(0.99 to 3.21)	0.18(0.11 to 0.28)	0.21(0.13 to 0.34)	0.2(-0.17 to 0.74)
Papua New Guinea	Liver cancer due to hepatitis B	13(9 to 18)	33(23 to 46)	1.57(0.81 to 2.58)	0.66(0.46 to 0.91)	0.69(0.47 to 0.96)	0.05(-0.25 to 0.45)
Papua New Guinea	Liver cancer due to hepatitis C	6(4 to 8)	15(10 to 23)	1.63(0.91 to 2.63)	0.41(0.26 to 0.58)	0.44(0.29 to 0.64)	0.08(-0.2 to 0.44)
Papua New Guinea	Liver cancer due to NASH	1(1 to 2)	4(3 to 7)	2.06(1.21 to 3.21)	0.09(0.06 to 0.14)	0.11(0.07 to 0.18)	0.26(-0.08 to 0.69)
Papua New Guinea	Liver cancer due to other causes	2(1 to 3)	5(3 to 7)	1.52(0.92 to 2.32)	0.07(0.05 to 0.09)	0.07(0.05 to 0.1)	0.09(-0.17 to 0.46)
Paraguay	Liver cancer	52(45 to 60)	112(84 to 146)	1.14(0.56 to 1.91)	2.32(2 to 2.66)	2.05(1.54 to 2.67)	-0.11(-0.35 to 0.2)

Paraguay	Liver cancer due to alcohol use	18(12 to 23)	38(24 to 55)	1.17(0.53 to 2.04)	0.8(0.55 to 1.04)	0.7(0.44 to 1.01)	-0.12(-0.38 to 0.22)
Paraguay	Liver cancer due to hepatitis B	10(7 to 13)	21(13 to 32)	1.18(0.54 to 2.08)	0.4(0.27 to 0.56)	0.36(0.22 to 0.56)	-0.09(-0.35 to 0.29)
Paraguay	Liver cancer due to hepatitis C	18(13 to 24)	40(27 to 56)	1.2(0.62 to 1.98)	0.87(0.63 to 1.12)	0.77(0.52 to 1.05)	-0.12(-0.35 to 0.19)
Paraguay	Liver cancer due to NASH	3(2 to 4)	6(4 to 9)	1.42(0.76 to 2.35)	0.11(0.08 to 0.17)	0.11(0.07 to 0.17)	-0.01(-0.28 to 0.36)
Paraguay	Liver cancer due to other causes	4(3 to 6)	7(4 to 10)	0.51(0.07 to 1)	0.14(0.1 to 0.19)	0.11(0.07 to 0.16)	-0.19(-0.42 to 0.1)
Peru	Liver cancer	719(619 to 829)	831(618 to 1103)	0.16(-0.18 to 0.61)	5.84(5.02 to 6.72)	2.6(1.93 to 3.45)	-0.55(-0.68 to -0.38)
Peru	Liver cancer due to alcohol use	216(151 to 291)	279(180 to 395)	0.29(-0.09 to 0.8)	1.87(1.31 to 2.52)	0.88(0.57 to 1.26)	-0.53(-0.67 to -0.35)
Peru	Liver cancer due to hepatitis B	345(270 to 436)	349(237 to 495)	0.01(-0.3 to 0.43)	2.7(2.06 to 3.43)	1.08(0.73 to 1.54)	-0.6(-0.72 to -0.43)
Peru	Liver cancer due to hepatitis C	45(27 to 67)	60(36 to 96)	0.35(-0.07 to 0.93)	0.41(0.25 to 0.6)	0.19(0.11 to 0.3)	-0.53(-0.67 to -0.34)
Peru	Liver cancer due to NASH	50(35 to 73)	79(49 to 115)	0.56(0.08 to 1.24)	0.44(0.29 to 0.63)	0.25(0.15 to 0.36)	-0.43(-0.61 to -0.2)
Peru	Liver cancer due to other causes	63(47 to 81)	64(42 to 91)	0.02(-0.27 to 0.44)	0.42(0.3 to 0.58)	0.2(0.13 to 0.28)	-0.53(-0.67 to -0.35)
Philippines	Liver cancer	3167(2422 to 3895)	5301(4244 to 6574)	0.67(0.22 to 1.42)	9.64(7.27 to 11.92)	6.38(5.16 to 7.84)	-0.34(-0.52 to -0.04)

Philippines	Liver cancer due to alcohol use	651(445 to 871)	1235(928 to 1630)	0.9(0.33 to 1.92)	2.09(1.43 to 2.77)	1.52(1.15 to 1.99)	-0.28(-0.49 to 0.11)
Philippines	Liver cancer due to hepatitis B	1653(1229 to 2057)	2458(1903 to 3172)	0.49(0.07 to 1.19)	4.53(3.29 to 5.74)	2.73(2.11 to 3.52)	-0.4(-0.57 to -0.11)
Philippines	Liver cancer due to hepatitis C	547(400 to 703)	1022(806 to 1295)	0.87(0.4 to 1.67)	2.07(1.54 to 2.62)	1.42(1.13 to 1.78)	-0.31(-0.48 to -0.04)
Philippines	Liver cancer due to NASH	196(147 to 252)	398(311 to 506)	1.04(0.53 to 1.86)	0.65(0.49 to 0.84)	0.52(0.41 to 0.65)	-0.21(-0.41 to 0.11)
Philippines	Liver cancer due to other causes	120(98 to 147)	187(147 to 235)	0.55(0.22 to 1.06)	0.29(0.23 to 0.37)	0.2(0.16 to 0.25)	-0.3(-0.46 to -0.05)
Poland	Liver cancer	3007(2892 to 3101)	1349(1131 to 1599)	-0.55(-0.62 to -0.46)	6.95(6.66 to 7.17)	1.95(1.63 to 2.31)	-0.72(-0.77 to -0.66)
Poland	Liver cancer due to alcohol use	1094(966 to 1222)	608(490 to 739)	-0.44(-0.55 to -0.32)	2.49(2.2 to 2.78)	0.86(0.7 to 1.05)	-0.65(-0.72 to -0.57)
Poland	Liver cancer due to hepatitis B	699(595 to 816)	252(194 to 325)	-0.64(-0.71 to -0.56)	1.61(1.38 to 1.86)	0.38(0.3 to 0.49)	-0.76(-0.81 to -0.71)
Poland	Liver cancer due to hepatitis C	857(743 to 974)	333(269 to 409)	-0.61(-0.67 to -0.54)	2.02(1.76 to 2.27)	0.46(0.37 to 0.56)	-0.77(-0.81 to -0.73)
Poland	Liver cancer due to NASH	238(202 to 280)	111(88 to 139)	-0.53(-0.61 to -0.44)	0.56(0.47 to 0.65)	0.16(0.12 to 0.19)	-0.72(-0.76 to -0.67)
Poland	Liver cancer due to other causes	120(103 to 138)	44(36 to 54)	-0.63(-0.69 to -0.57)	0.29(0.25 to 0.33)	0.08(0.07 to 0.1)	-0.7(-0.75 to -0.66)
Portugal	Liver cancer	255(241 to 269)	1058(825 to 1339)	3.16(2.23 to 4.28)	1.86(1.77 to 1.96)	4.9(3.79 to 6.26)	1.63(1.03 to 2.35)

Portugal	Liver cancer due to alcohol use	98(74 to 122)	421(286 to 591)	3.3(2.28 to 4.57)	0.69(0.53 to 0.86)	1.96(1.34 to 2.78)	1.83(1.13 to 2.68)
Portugal	Liver cancer due to hepatitis B	36(25 to 50)	137(84 to 213)	2.8(1.83 to 3.97)	0.28(0.2 to 0.38)	0.74(0.46 to 1.14)	1.65(0.96 to 2.45)
Portugal	Liver cancer due to hepatitis C	92(69 to 117)	388(268 to 539)	3.23(2.29 to 4.39)	0.66(0.51 to 0.83)	1.64(1.11 to 2.29)	1.49(0.92 to 2.17)
Portugal	Liver cancer due to NASH	14(10 to 20)	66(41 to 100)	3.75(2.6 to 5.15)	0.1(0.07 to 0.14)	0.29(0.18 to 0.44)	1.86(1.15 to 2.81)
Portugal	Liver cancer due to other causes	15(11 to 20)	46(29 to 70)	2.07(1.27 to 3.03)	0.13(0.1 to 0.17)	0.26(0.18 to 0.37)	1.01(0.51 to 1.64)
Puerto Rico	Liver cancer	241(225 to 254)	188(141 to 244)	-0.22(-0.41 to 0.01)	6.57(6.15 to 6.93)	2.76(2.06 to 3.59)	-0.58(-0.69 to -0.45)
Puerto Rico	Liver cancer due to alcohol use	84(62 to 107)	66(43 to 94)	-0.22(-0.43 to 0.03)	2.29(1.69 to 2.92)	0.95(0.62 to 1.37)	-0.58(-0.7 to -0.45)
Puerto Rico	Liver cancer due to hepatitis B	64(46 to 86)	44(28 to 66)	-0.31(-0.49 to -0.08)	1.76(1.29 to 2.35)	0.71(0.46 to 1.06)	-0.59(-0.7 to -0.47)
Puerto Rico	Liver cancer due to hepatitis C	54(37 to 73)	44(28 to 64)	-0.19(-0.38 to 0.04)	1.48(1.03 to 1.96)	0.58(0.37 to 0.85)	-0.61(-0.7 to -0.49)
Puerto Rico	Liver cancer due to NASH	24(17 to 34)	25(16 to 38)	0.04(-0.23 to 0.38)	0.66(0.46 to 0.92)	0.35(0.23 to 0.52)	-0.47(-0.61 to -0.29)
Puerto Rico	Liver cancer due to other causes	14(10 to 19)	10(6 to 14)	-0.31(-0.49 to -0.09)	0.38(0.27 to 0.52)	0.16(0.11 to 0.23)	-0.57(-0.68 to -0.43)
Qatar	Liver cancer	15(11 to 19)	108(76 to 147)	6.4(3.83 to 10.15)	16.14(12.14 to 21.04)	17.39(12.83 to 22.91)	0.08(-0.25 to 0.54)

Qatar	Liver cancer due to alcohol use	2(1 to 2)	12(6 to 19)	6.74(3.81 to 11.48)	1.75(1.01 to 2.8)	1.89(1.08 to 3.06)	0.08(-0.3 to 0.68)
Qatar	Liver cancer due to hepatitis B	6(4 to 9)	44(28 to 67)	5.92(3.32 to 10.1)	5.26(3.43 to 7.9)	4.73(2.91 to 7.33)	-0.1(-0.42 to 0.33)
Qatar	Liver cancer due to hepatitis C	4(3 to 6)	31(19 to 46)	6.34(3.97 to 9.8)	6.25(4.02 to 8.87)	7.09(4.79 to 9.84)	0.13(-0.21 to 0.6)
Qatar	Liver cancer due to NASH	2(1 to 2)	15(9 to 23)	8.44(5.28 to 13.19)	2.04(1.26 to 3.16)	2.84(1.77 to 4.32)	0.39(-0.04 to 1.01)
Qatar	Liver cancer due to other causes	1(1 to 1)	7(4 to 10)	6(3.7 to 9.49)	0.84(0.52 to 1.27)	0.85(0.5 to 1.35)	0.01(-0.32 to 0.43)
Republic of Korea	Liver cancer	3548(2935 to 4339)	20367(16678 to 24504)	4.74(3.28 to 6.63)	10.99(9.18 to 13.42)	22.8(18.72 to 27.32)	1.07(0.57 to 1.74)
Republic of Korea	Liver cancer due to alcohol use	447(285 to 662)	3603(2377 to 5240)	7.06(4.74 to 10.07)	1.44(0.93 to 2.12)	3.98(2.6 to 5.79)	1.76(1.04 to 2.71)
Republic of Korea	Liver cancer due to hepatitis B	2354(1878 to 2935)	11449(8885 to 14578)	3.86(2.52 to 5.6)	6.83(5.43 to 8.45)	12.82(9.97 to 16.31)	0.88(0.38 to 1.52)
Republic of Korea	Liver cancer due to hepatitis C	476(314 to 679)	3549(2361 to 4874)	6.45(4.35 to 9.32)	1.81(1.23 to 2.55)	3.98(2.69 to 5.45)	1.19(0.61 to 1.97)
Republic of Korea	Liver cancer due to NASH	148(98 to 213)	1119(731 to 1650)	6.55(4.32 to 9.62)	0.53(0.36 to 0.76)	1.25(0.83 to 1.83)	1.37(0.72 to 2.27)
Republic of Korea	Liver cancer due to other causes	122(82 to 174)	647(423 to 945)	4.28(2.76 to 6.45)	0.38(0.25 to 0.54)	0.76(0.52 to 1.09)	1.02(0.48 to 1.74)
Republic of Moldova	Liver cancer	73(68 to 78)	132(111 to 155)	0.8(0.53 to 1.1)	1.65(1.54 to 1.76)	2.3(1.95 to 2.7)	0.4(0.19 to 0.63)
Republic of Moldova	Liver cancer due to alcohol use	37(30 to 43)	65(51 to 81)	0.77(0.47 to 1.12)	0.81(0.68 to 0.95)	1.12(0.88 to	0.37(0.14 to 0.63)

						1.39)	
Republic of Moldova	Liver cancer due to hepatitis B	15(11 to 21)	24(16 to 35)	0.61(0.32 to 0.97)	0.32(0.23 to 0.44)	0.43(0.3 to 0.61)	0.33(0.1 to 0.6)
Republic of Moldova	Liver cancer due to hepatitis C	14(10 to 19)	30(20 to 43)	1.13(0.76 to 1.53)	0.33(0.24 to 0.45)	0.52(0.35 to 0.73)	0.55(0.29 to 0.82)
Republic of Moldova	Liver cancer due to NASH	3(2 to 5)	9(6 to 12)	1.52(1 to 2.15)	0.08(0.06 to 0.11)	0.15(0.1 to 0.22)	0.88(0.51 to 1.32)
Republic of Moldova	Liver cancer due to other causes	4(2 to 7)	4(3 to 6)	-0.05(-0.41 to 0.53)	0.1(0.06 to 0.15)	0.09(0.07 to 0.12)	-0.06(-0.39 to 0.49)
Romania	Liver cancer	483(433 to 543)	1064(865 to 1294)	1.2(0.75 to 1.7)	1.75(1.56 to 1.95)	3.02(2.45 to 3.7)	0.73(0.38 to 1.12)
Romania	Liver cancer due to alcohol use	197(147 to 247)	480(343 to 628)	1.44(0.91 to 2.03)	0.69(0.52 to 0.86)	1.34(0.95 to 1.76)	0.93(0.52 to 1.42)
Romania	Liver cancer due to hepatitis B	119(84 to 161)	209(140 to 306)	0.76(0.37 to 1.22)	0.43(0.31 to 0.57)	0.65(0.44 to 0.94)	0.52(0.2 to 0.93)
Romania	Liver cancer due to hepatitis C	118(81 to 159)	261(175 to 365)	1.22(0.77 to 1.73)	0.43(0.31 to 0.57)	0.69(0.46 to 0.97)	0.59(0.27 to 0.95)
Romania	Liver cancer due to NASH	30(21 to 43)	82(54 to 120)	1.73(1.14 to 2.41)	0.11(0.08 to 0.16)	0.22(0.15 to 0.32)	1.02(0.57 to 1.49)
Romania	Liver cancer due to other causes	20(15 to 27)	33(22 to 47)	0.62(0.25 to 1.11)	0.08(0.06 to 0.1)	0.12(0.09 to 0.16)	0.45(0.15 to 0.83)
Russian Federation	Liver cancer	3044(2910 to 3207)	6598(5591 to 7845)	1.17(0.87 to 1.52)	1.72(1.64 to 1.8)	2.91(2.46 to 3.44)	0.7(0.46 to 0.97)
Russian Federation	Liver cancer due to alcohol use	1015(880 to 1155)	2472(1982 to 3077)	1.44(1.05 to 1.9)	0.55(0.48 to 0.63)	1.06(0.85 to	0.92(0.62 to 1.29)

						1.32)	
Russian Federation	Liver cancer due to hepatitis B	837(717 to 982)	1527(1188 to 1986)	0.83(0.53 to 1.15)	0.46(0.4 to 0.54)	0.7(0.55 to 0.91)	0.51(0.27 to 0.79)
Russian Federation	Liver cancer due to hepatitis C	767(657 to 878)	1757(1418 to 2117)	1.29(1.01 to 1.62)	0.43(0.37 to 0.49)	0.74(0.6 to 0.89)	0.71(0.49 to 0.95)
Russian Federation	Liver cancer due to NASH	207(176 to 245)	548(437 to 671)	1.65(1.3 to 2.05)	0.12(0.1 to 0.14)	0.24(0.19 to 0.29)	1.03(0.76 to 1.32)
Russian Federation	Liver cancer due to other causes	219(181 to 277)	293(220 to 382)	0.34(0.1 to 0.58)	0.15(0.12 to 0.2)	0.17(0.12 to 0.25)	0.15(-0.13 to 0.45)
Rwanda	Liver cancer	158(114 to 220)	279(212 to 369)	0.77(0.1 to 1.73)	5.05(3.67 to 6.79)	4.4(3.48 to 5.65)	-0.13(-0.42 to 0.29)
Rwanda	Liver cancer due to alcohol use	47(29 to 72)	83(55 to 123)	0.78(0.13 to 1.77)	1.6(1.03 to 2.38)	1.37(0.94 to 1.96)	-0.14(-0.43 to 0.28)
Rwanda	Liver cancer due to hepatitis B	47(31 to 71)	82(53 to 118)	0.73(0 to 1.8)	1.38(0.91 to 2.05)	1.11(0.73 to 1.61)	-0.19(-0.51 to 0.26)
Rwanda	Liver cancer due to hepatitis C	30(18 to 48)	55(37 to 79)	0.85(0.21 to 1.83)	1.15(0.7 to 1.75)	1.05(0.72 to 1.48)	-0.08(-0.37 to 0.35)
Rwanda	Liver cancer due to NASH	15(9 to 24)	31(20 to 45)	1.01(0.26 to 2.17)	0.53(0.32 to 0.83)	0.53(0.34 to 0.76)	-0.01(-0.34 to 0.47)
Rwanda	Liver cancer due to other causes	19(12 to 31)	29(19 to 43)	0.5(-0.26 to 1.63)	0.39(0.24 to 0.6)	0.34(0.23 to 0.48)	-0.13(-0.49 to 0.38)
Saint Kitts and Nevis	Liver cancer	5(4 to 5)	2(2 to 3)	-0.48(-0.58 to -0.35)	12.71(11.56 to 14.03)	3.73(3.12 to 4.44)	-0.71(-0.76 to -0.64)
Saint Kitts and Nevis	Liver cancer due to alcohol use	2(1 to 2)	1(1 to 1)	-0.44(-0.56 to -0.29)	3.92(2.84 to 5.13)	1.28(0.9 to	-0.67(-0.73 to -0.6)

						1.76)	
Saint Kitts and Nevis	Liver cancer due to hepatitis B	1(1 to 2)	1(0 to 1)	-0.45(-0.59 to -0.28)	3.78(2.82 to 4.91)	1.02(0.69 to 1.45)	-0.73(-0.79 to -0.66)
Saint Kitts and Nevis	Liver cancer due to hepatitis C	1(1 to 2)	1(0 to 1)	-0.57(-0.66 to -0.46)	3.06(2.15 to 4.07)	0.84(0.56 to 1.18)	-0.73(-0.77 to -0.67)
Saint Kitts and Nevis	Liver cancer due to NASH	0(0 to 1)	0(0 to 0)	-0.46(-0.58 to -0.28)	1.09(0.77 to 1.53)	0.37(0.26 to 0.53)	-0.66(-0.73 to -0.57)
Saint Kitts and Nevis	Liver cancer due to other causes	0(0 to 0)	0(0 to 0)	-0.56(-0.67 to -0.4)	0.86(0.62 to 1.18)	0.22(0.15 to 0.32)	-0.74(-0.8 to -0.66)
Saint Lucia	Liver cancer	5(5 to 6)	5(4 to 6)	-0.14(-0.3 to 0.04)	6.05(5.55 to 6.61)	2.16(1.8 to 2.59)	-0.64(-0.71 to -0.57)
Saint Lucia	Liver cancer due to alcohol use	2(1 to 2)	2(1 to 2)	-0.07(-0.25 to 0.16)	2.2(1.67 to 2.79)	0.84(0.61 to 1.1)	-0.62(-0.69 to -0.53)
Saint Lucia	Liver cancer due to hepatitis B	1(1 to 2)	1(1 to 2)	-0.2(-0.35 to -0.02)	1.64(1.22 to 2.16)	0.54(0.38 to 0.75)	-0.67(-0.73 to -0.6)
Saint Lucia	Liver cancer due to hepatitis C	1(1 to 2)	1(1 to 1)	-0.18(-0.33 to -0.01)	1.37(0.98 to 1.82)	0.45(0.3 to 0.64)	-0.67(-0.73 to -0.6)
Saint Lucia	Liver cancer due to NASH	0(0 to 1)	0(0 to 1)	0.04(-0.17 to 0.3)	0.45(0.31 to 0.62)	0.19(0.13 to 0.27)	-0.57(-0.65 to -0.47)
Saint Lucia	Liver cancer due to other causes	0(0 to 1)	0(0 to 0)	-0.33(-0.48 to -0.16)	0.4(0.29 to 0.54)	0.13(0.09 to 0.18)	-0.67(-0.74 to -0.59)
Saint Vincent and the Grenadines	Liver cancer	5(5 to 6)	4(3 to 5)	-0.23(-0.35 to -0.1)	7.15(6.57 to 7.76)	2.97(2.57 to 3.44)	-0.58(-0.65 to -0.51)

Saint Vincent and the Grenadines	Liver cancer due to alcohol use	2(1 to 2)	2(1 to 2)	-0.08(-0.24 to 0.12)	2.29(1.65 to 2.96)	1.13(0.83 to 1.45)	-0.51(-0.59 to -0.41)
Saint Vincent and the Grenadines	Liver cancer due to hepatitis B	2(1 to 2)	1(1 to 1)	-0.3(-0.42 to -0.15)	2.16(1.6 to 2.85)	0.81(0.59 to 1.09)	-0.62(-0.69 to -0.55)
Saint Vincent and the Grenadines	Liver cancer due to hepatitis C	1(1 to 2)	1(1 to 1)	-0.33(-0.44 to -0.22)	1.68(1.19 to 2.23)	0.6(0.41 to 0.84)	-0.64(-0.7 to -0.59)
Saint Vincent and the Grenadines	Liver cancer due to NASH	0(0 to 1)	0(0 to 0)	-0.12(-0.28 to 0.09)	0.54(0.38 to 0.77)	0.26(0.18 to 0.37)	-0.51(-0.6 to -0.41)
Saint Vincent and the Grenadines	Liver cancer due to other causes	0(0 to 1)	0(0 to 0)	-0.42(-0.53 to -0.29)	0.48(0.36 to 0.63)	0.18(0.12 to 0.24)	-0.64(-0.7 to -0.56)
Samoa	Liver cancer	6(4 to 7)	8(6 to 10)	0.39(-0.01 to 0.88)	6.01(4.76 to 7.6)	4.99(3.82 to 6.29)	-0.17(-0.4 to 0.1)
Samoa	Liver cancer due to alcohol use	1(0 to 1)	1(1 to 1)	0.37(-0.02 to 0.92)	0.79(0.47 to 1.21)	0.65(0.39 to 0.99)	-0.17(-0.4 to 0.14)
Samoa	Liver cancer due to hepatitis B	3(2 to 4)	4(3 to 6)	0.37(-0.06 to 0.94)	3.32(2.38 to 4.37)	2.7(1.94 to 3.68)	-0.19(-0.43 to 0.13)
Samoa	Liver cancer due to hepatitis C	1(1 to 1)	1(1 to 2)	0.4(0.03 to 0.87)	1.18(0.77 to 1.73)	0.99(0.63 to 1.45)	-0.16(-0.37 to 0.1)
Samoa	Liver cancer due to NASH	0(0 to 1)	1(0 to 1)	0.56(0.12 to 1.17)	0.47(0.31 to 0.71)	0.44(0.29 to 0.65)	-0.06(-0.31 to 0.27)
Samoa	Liver cancer due to other causes	0(0 to 0)	0(0 to 0)	0.28(-0.09 to 0.8)	0.24(0.16 to 0.35)	0.2(0.13 to 0.3)	-0.17(-0.4 to 0.14)

San Marino	Liver cancer	1(1 to 1)	2(1 to 3)	1.67(0.92 to 2.69)	2.21(1.85 to 2.62)	3.22(2.45 to 4.29)	0.46(0.05 to 1.02)
San Marino	Liver cancer due to alcohol use	0(0 to 0)	1(0 to 1)	1.72(0.89 to 2.81)	0.8(0.58 to 1.06)	1.22(0.8 to 1.76)	0.53(0.05 to 1.18)
San Marino	Liver cancer due to hepatitis B	0(0 to 0)	0(0 to 0)	1.39(0.69 to 2.38)	0.32(0.22 to 0.48)	0.46(0.29 to 0.7)	0.42(0 to 1.03)
San Marino	Liver cancer due to hepatitis C	0(0 to 0)	1(0 to 1)	1.74(0.96 to 2.79)	0.77(0.55 to 1.03)	1.08(0.73 to 1.55)	0.4(0.01 to 0.96)
San Marino	Liver cancer due to NASH	0(0 to 0)	0(0 to 0)	2.03(1.15 to 3.33)	0.13(0.09 to 0.2)	0.21(0.14 to 0.34)	0.62(0.15 to 1.34)
San Marino	Liver cancer due to other causes	0(0 to 0)	0(0 to 0)	1.24(0.63 to 2.03)	0.19(0.14 to 0.24)	0.25(0.17 to 0.34)	0.33(-0.02 to 0.78)
Sao Tome and Principe	Liver cancer	2(1 to 2)	3(2 to 4)	0.86(0.23 to 1.67)	2.44(1.89 to 2.96)	2.64(1.77 to 3.48)	0.08(-0.28 to 0.52)
Sao Tome and Principe	Liver cancer due to alcohol use	0(0 to 0)	1(0 to 1)	1.21(0.39 to 2.21)	0.42(0.27 to 0.59)	0.56(0.33 to 0.87)	0.35(-0.13 to 0.93)
Sao Tome and Principe	Liver cancer due to hepatitis B	1(1 to 1)	2(1 to 2)	0.85(0.17 to 1.82)	1.23(0.87 to 1.58)	1.21(0.76 to 1.73)	-0.01(-0.36 to 0.45)
Sao Tome and Principe	Liver cancer due to hepatitis C	0(0 to 0)	0(0 to 1)	0.54(0.06 to 1.2)	0.47(0.31 to 0.65)	0.47(0.28 to 0.72)	0(-0.31 to 0.39)
Sao Tome and Principe	Liver cancer due to NASH	0(0 to 0)	0(0 to 0)	1.1(0.42 to 2.04)	0.2(0.14 to 0.3)	0.26(0.16 to 0.39)	0.28(-0.12 to 0.88)
Sao Tome and Principe	Liver cancer due to other causes	0(0 to 0)	0(0 to 0)	0.66(0.15 to 1.45)	0.12(0.09 to 0.17)	0.13(0.09 to 0.19)	0.07(-0.25 to 0.52)

Saudi Arabia	Liver cancer	291(212 to 373)	855(645 to 1124)	1.94(0.92 to 3.57)	5.03(3.66 to 6.43)	5.29(4.17 to 6.69)	0.05(-0.3 to 0.6)
Saudi Arabia	Liver cancer due to alcohol use	22(13 to 36)	64(37 to 102)	1.85(0.82 to 3.58)	0.41(0.24 to 0.65)	0.43(0.25 to 0.68)	0.05(-0.33 to 0.67)
Saudi Arabia	Liver cancer due to hepatitis B	127(85 to 177)	334(225 to 477)	1.63(0.64 to 3.3)	1.96(1.28 to 2.79)	1.66(1.11 to 2.39)	-0.15(-0.44 to 0.36)
Saudi Arabia	Liver cancer due to hepatitis C	96(62 to 134)	284(188 to 399)	1.97(0.94 to 3.42)	1.91(1.25 to 2.64)	2.11(1.46 to 2.88)	0.11(-0.26 to 0.66)
Saudi Arabia	Liver cancer due to NASH	28(17 to 42)	123(81 to 187)	3.44(1.89 to 5.9)	0.52(0.32 to 0.78)	0.83(0.55 to 1.24)	0.6(0.06 to 1.46)
Saudi Arabia	Liver cancer due to other causes	18(13 to 26)	51(32 to 77)	1.78(0.84 to 3.15)	0.24(0.15 to 0.35)	0.26(0.16 to 0.39)	0.06(-0.3 to 0.63)
Senegal	Liver cancer	77(61 to 92)	163(120 to 208)	1.12(0.51 to 1.9)	2.23(1.78 to 2.65)	2.07(1.55 to 2.6)	-0.07(-0.33 to 0.27)
Senegal	Liver cancer due to alcohol use	10(6 to 14)	20(12 to 30)	1.12(0.45 to 1.98)	0.3(0.19 to 0.44)	0.28(0.17 to 0.41)	-0.08(-0.36 to 0.29)
Senegal	Liver cancer due to hepatitis B	46(36 to 57)	96(67 to 128)	1.07(0.42 to 1.87)	1.33(1.03 to 1.65)	1.18(0.84 to 1.57)	-0.12(-0.39 to 0.21)
Senegal	Liver cancer due to hepatitis C	6(4 to 9)	13(8 to 21)	1.2(0.54 to 2.05)	0.21(0.13 to 0.31)	0.2(0.12 to 0.31)	-0.05(-0.32 to 0.3)
Senegal	Liver cancer due to NASH	7(5 to 11)	19(12 to 28)	1.56(0.77 to 2.64)	0.24(0.16 to 0.34)	0.26(0.17 to 0.39)	0.11(-0.23 to 0.54)
Senegal	Liver cancer due to other causes	8(5 to 11)	15(10 to 21)	0.93(0.25 to 1.82)	0.15(0.1 to 0.2)	0.15(0.1 to 0.22)	0(-0.31 to 0.41)

Serbia	Liver cancer	682(568 to 789)	862(678 to 1087)	0.26(-0.04 to 0.66)	5.99(5 to 6.87)	5.42(4.28 to 6.82)	-0.09(-0.31 to 0.19)
Serbia	Liver cancer due to alcohol use	245(174 to 318)	328(222 to 455)	0.34(-0.01 to 0.82)	2.1(1.52 to 2.72)	2.02(1.36 to 2.79)	-0.04(-0.28 to 0.28)
Serbia	Liver cancer due to hepatitis B	165(114 to 231)	161(101 to 247)	-0.02(-0.28 to 0.33)	1.4(0.97 to 1.93)	1.09(0.7 to 1.65)	-0.22(-0.42 to 0.05)
Serbia	Liver cancer due to hepatitis C	212(145 to 294)	288(194 to 405)	0.36(0 to 0.81)	1.93(1.37 to 2.65)	1.76(1.22 to 2.43)	-0.09(-0.31 to 0.21)
Serbia	Liver cancer due to NASH	38(25 to 57)	61(39 to 91)	0.6(0.15 to 1.18)	0.36(0.23 to 0.52)	0.38(0.25 to 0.56)	0.06(-0.21 to 0.44)
Serbia	Liver cancer due to other causes	22(15 to 31)	24(15 to 36)	0.08(-0.21 to 0.46)	0.2(0.15 to 0.28)	0.17(0.11 to 0.24)	-0.18(-0.38 to 0.09)
Seychelles	Liver cancer	4(4 to 5)	6(5 to 7)	0.44(0.16 to 0.78)	7.69(6.67 to 8.9)	5.56(4.68 to 6.58)	-0.28(-0.41 to -0.12)
Seychelles	Liver cancer due to alcohol use	1(1 to 1)	2(1 to 2)	0.78(0.36 to 1.28)	1.59(1.05 to 2.3)	1.42(0.94 to 2)	-0.11(-0.31 to 0.13)
Seychelles	Liver cancer due to hepatitis B	2(1 to 2)	2(2 to 3)	0.34(0.04 to 0.74)	3.26(2.46 to 4.3)	2.07(1.51 to 2.79)	-0.37(-0.5 to -0.19)
Seychelles	Liver cancer due to hepatitis C	1(1 to 1)	1(1 to 2)	0.27(0.03 to 0.55)	1.91(1.32 to 2.6)	1.32(0.9 to 1.79)	-0.31(-0.43 to -0.16)
Seychelles	Liver cancer due to NASH	0(0 to 1)	1(0 to 1)	0.6(0.27 to 0.98)	0.69(0.49 to 0.97)	0.59(0.41 to 0.83)	-0.15(-0.33 to 0.04)
Seychelles	Liver cancer due to other causes	0(0 to 0)	0(0 to 0)	0.3(0.05 to 0.62)	0.22(0.15 to 0.32)	0.16(0.11 to 0.22)	-0.29(-0.43 to -0.12)
Sierra Leone	Liver cancer	116(87 to 150)	176(130 to 235)	0.52(0.07 to 1.15)	5.83(4.43 to 7.54)	4.62(3.5 to [illegible]	-0.21(-0.44 to 0.1)

						6.03)	
Sierra Leone	Liver cancer due to alcohol use	21(13 to 32)	30(18 to 46)	0.46(0 to 1.1)	1.08(0.69 to 1.66)	0.86(0.53 to 1.31)	-0.2(-0.45 to 0.13)
Sierra Leone	Liver cancer due to hepatitis B	60(41 to 81)	88(60 to 124)	0.47(0 to 1.14)	2.94(2.04 to 4.01)	2.14(1.48 to 3.07)	-0.27(-0.49 to 0.04)
Sierra Leone	Liver cancer due to hepatitis C	20(12 to 29)	30(18 to 43)	0.5(0.06 to 1.1)	1.09(0.7 to 1.59)	0.93(0.58 to 1.34)	-0.15(-0.39 to 0.18)
Sierra Leone	Liver cancer due to NASH	8(5 to 12)	15(10 to 23)	0.87(0.3 to 1.63)	0.43(0.27 to 0.63)	0.43(0.28 to 0.65)	0.01(-0.29 to 0.4)
Sierra Leone	Liver cancer due to other causes	8(5 to 12)	14(9 to 20)	0.7(0.13 to 1.54)	0.28(0.19 to 0.41)	0.26(0.17 to 0.39)	-0.08(-0.37 to 0.32)
Singapore	Liver cancer	189(178 to 201)	881(707 to 1110)	3.65(2.71 to 4.91)	8.44(7.9 to 8.99)	11.5(9.24 to 14.41)	0.36(0.1 to 0.72)
Singapore	Liver cancer due to alcohol use	14(9 to 20)	70(41 to 113)	4.16(2.87 to 5.7)	0.62(0.4 to 0.91)	0.91(0.53 to 1.46)	0.46(0.1 to 0.89)
Singapore	Liver cancer due to hepatitis B	123(105 to 142)	499(364 to 652)	3.06(2.17 to 4.17)	5.23(4.39 to 6.1)	6.36(4.61 to 8.31)	0.22(-0.05 to 0.54)
Singapore	Liver cancer due to hepatitis C	41(27 to 56)	246(156 to 355)	5.05(3.8 to 6.78)	2.02(1.37 to 2.74)	3.32(2.11 to 4.72)	0.65(0.31 to 1.1)
Singapore	Liver cancer due to NASH	7(4 to 10)	42(25 to 63)	5.33(3.85 to 7.19)	0.32(0.21 to 0.48)	0.56(0.34 to 0.87)	0.77(0.36 to 1.29)
Singapore	Liver cancer due to other causes	6(4 to 8)	24(15 to 37)	3.22(2.11 to 4.58)	0.25(0.18 to 0.35)	0.34(0.22 to 0.51)	0.35(0.02 to 0.75)
Slovakia	Liver cancer	272(255 to 293)	311(242 to 395)	0.14(-0.12 to 0.48)	4.55(4.27 to 4.87)	3.41(2.66 to	-0.25(-0.42 to -0.03)

						4.33)	
Slovakia	Liver cancer due to alcohol use	121(94 to 145)	143(99 to 194)	0.18(-0.12 to 0.57)	2(1.57 to 2.4)	1.53(1.06 to 2.09)	-0.23(-0.43 to 0.01)
Slovakia	Liver cancer due to hepatitis B	65(47 to 87)	62(39 to 93)	-0.05(-0.3 to 0.26)	1.09(0.79 to 1.46)	0.7(0.45 to 1.04)	-0.36(-0.52 to -0.14)
Slovakia	Liver cancer due to hepatitis C	60(42 to 81)	73(47 to 107)	0.22(-0.07 to 0.58)	1(0.7 to 1.34)	0.79(0.51 to 1.14)	-0.21(-0.4 to 0.01)
Slovakia	Liver cancer due to NASH	17(12 to 23)	24(15 to 35)	0.39(0.05 to 0.82)	0.28(0.2 to 0.39)	0.26(0.17 to 0.38)	-0.09(-0.31 to 0.2)
Slovakia	Liver cancer due to other causes	10(7 to 13)	10(6 to 15)	0.03(-0.23 to 0.33)	0.17(0.13 to 0.22)	0.13(0.09 to 0.19)	-0.21(-0.4 to 0.01)
Slovenia	Liver cancer	71(55 to 91)	223(171 to 286)	2.14(1.14 to 3.48)	2.91(2.23 to 3.72)	5.3(4.04 to 6.83)	0.82(0.24 to 1.61)
Slovenia	Liver cancer due to alcohol use	28(19 to 39)	89(60 to 125)	2.19(1.17 to 3.67)	1.13(0.77 to 1.56)	2.12(1.42 to 3.01)	0.87(0.27 to 1.73)
Slovenia	Liver cancer due to hepatitis B	18(11 to 26)	47(30 to 71)	1.68(0.76 to 2.95)	0.72(0.47 to 1.07)	1.21(0.76 to 1.81)	0.67(0.11 to 1.46)
Slovenia	Liver cancer due to hepatitis C	18(12 to 27)	60(39 to 86)	2.34(1.29 to 3.63)	0.73(0.48 to 1.08)	1.33(0.86 to 1.92)	0.82(0.25 to 1.55)
Slovenia	Liver cancer due to NASH	5(3 to 8)	20(12 to 32)	3.01(1.71 to 4.76)	0.21(0.13 to 0.31)	0.46(0.29 to 0.7)	1.22(0.52 to 2.18)
Slovenia	Liver cancer due to other causes	3(2 to 4)	7(4 to 10)	1.56(0.74 to 2.64)	0.12(0.08 to 0.17)	0.18(0.12 to 0.27)	0.58(0.11 to 1.19)
Solomon Islands	Liver cancer	11(8 to 14)	19(15 to 24)	0.77(0.24 to 1.51)	6.67(4.95 to 8.63)	5.37(4.3 to	-0.2(-0.42 to 0.14)

						6.55)	
Solomon Islands	Liver cancer due to alcohol use	1(1 to 2)	2(1 to 3)	0.88(0.28 to 1.82)	0.72(0.43 to 1.17)	0.62(0.38 to 0.94)	-0.14(-0.4 to 0.28)
Solomon Islands	Liver cancer due to hepatitis B	6(5 to 9)	11(8 to 14)	0.67(0.17 to 1.38)	3.72(2.61 to 4.97)	2.75(2.06 to 3.61)	-0.26(-0.47 to 0.05)
Solomon Islands	Liver cancer due to hepatitis C	2(1 to 3)	3(2 to 4)	0.85(0.29 to 1.7)	1.32(0.8 to 1.94)	1.15(0.77 to 1.61)	-0.13(-0.37 to 0.24)
Solomon Islands	Liver cancer due to NASH	1(0 to 1)	2(1 to 2)	1.13(0.43 to 2.18)	0.49(0.31 to 0.78)	0.48(0.32 to 0.69)	-0.02(-0.31 to 0.41)
Solomon Islands	Liver cancer due to other causes	1(1 to 1)	2(1 to 3)	0.83(0.26 to 1.58)	0.41(0.27 to 0.58)	0.36(0.25 to 0.49)	-0.12(-0.37 to 0.23)
Somalia	Liver cancer	98(59 to 170)	240(150 to 425)	1.44(0.65 to 2.68)	3.53(2.21 to 6.12)	3.33(2.15 to 5.91)	-0.06(-0.35 to 0.36)
Somalia	Liver cancer due to alcohol use	19(10 to 41)	44(24 to 86)	1.35(0.57 to 2.54)	0.76(0.41 to 1.63)	0.68(0.37 to 1.31)	-0.11(-0.38 to 0.3)
Somalia	Liver cancer due to hepatitis B	38(21 to 74)	90(51 to 167)	1.36(0.52 to 2.63)	1.21(0.67 to 2.31)	1.09(0.62 to 2.02)	-0.1(-0.4 to 0.36)
Somalia	Liver cancer due to hepatitis C	19(10 to 32)	48(27 to 85)	1.57(0.73 to 2.77)	0.86(0.49 to 1.43)	0.87(0.51 to 1.56)	0.01(-0.29 to 0.45)
Somalia	Liver cancer due to NASH	10(5 to 17)	25(15 to 44)	1.6(0.75 to 2.96)	0.4(0.22 to 0.68)	0.41(0.24 to 0.71)	0.01(-0.3 to 0.48)
Somalia	Liver cancer due to other causes	13(7 to 22)	33(19 to 55)	1.53(0.55 to 3.08)	0.29(0.16 to 0.48)	0.27(0.16 to 0.46)	-0.04(-0.37 to 0.43)
South Africa	Liver cancer	1306(866 to 2211)	2600(2291 to 2946)	0.99(0.08 to 2.18)	5.79(3.76 to 9.81)	5.6(4.94 to	-0.03(-0.47 to 0.56)

						6.35)	
South Africa	Liver cancer due to alcohol use	257(150 to 487)	541(442 to 660)	1.11(0.04 to 2.66)	1.19(0.69 to 2.25)	1.18(0.96 to 1.42)	-0.01(-0.51 to 0.74)
South Africa	Liver cancer due to hepatitis B	518(334 to 909)	995(831 to 1183)	0.92(-0.04 to 2.19)	2.07(1.3 to 3.7)	1.96(1.64 to 2.32)	-0.05(-0.53 to 0.58)
South Africa	Liver cancer due to hepatitis C	317(203 to 492)	630(534 to 751)	0.99(0.24 to 2.01)	1.61(1.02 to 2.49)	1.51(1.29 to 1.81)	-0.06(-0.41 to 0.44)
South Africa	Liver cancer due to NASH	129(83 to 211)	299(252 to 357)	1.33(0.34 to 2.63)	0.59(0.37 to 0.98)	0.67(0.57 to 0.81)	0.14(-0.34 to 0.8)
South Africa	Liver cancer due to other causes	87(62 to 134)	134(112 to 159)	0.54(-0.04 to 1.2)	0.33(0.22 to 0.51)	0.27(0.23 to 0.32)	-0.16(-0.49 to 0.23)
South Sudan	Liver cancer	78(49 to 141)	110(65 to 192)	0.41(0.01 to 1.01)	3.03(1.88 to 5.53)	2.74(1.64 to 4.77)	-0.1(-0.34 to 0.25)
South Sudan	Liver cancer due to alcohol use	17(8 to 38)	23(11 to 47)	0.36(-0.06 to 0.97)	0.7(0.34 to 1.57)	0.62(0.31 to 1.29)	-0.11(-0.38 to 0.26)
South Sudan	Liver cancer due to hepatitis B	26(15 to 52)	38(20 to 73)	0.47(0 to 1.18)	0.98(0.54 to 1.98)	0.86(0.45 to 1.63)	-0.12(-0.39 to 0.27)
South Sudan	Liver cancer due to hepatitis C	16(9 to 27)	23(12 to 39)	0.42(0 to 0.97)	0.74(0.43 to 1.26)	0.68(0.39 to 1.15)	-0.08(-0.33 to 0.25)
South Sudan	Liver cancer due to NASH	8(5 to 15)	13(7 to 23)	0.55(0.09 to 1.21)	0.36(0.2 to 0.63)	0.36(0.2 to 0.61)	0(-0.27 to 0.39)
South Sudan	Liver cancer due to other causes	10(6 to 16)	13(8 to 20)	0.26(-0.22 to 0.89)	0.25(0.15 to 0.41)	0.21(0.13 to 0.34)	-0.14(-0.39 to 0.21)
Spain	Liver cancer	2132(2023 to 2225)	5411(4207 to 6881)	1.54(0.97 to 2.25)	3.92(3.72 to 4.09)	6.01(4.63 to	0.53(0.18 to 0.99)

						7.72)	
Spain	Liver cancer due to alcohol use	667(470 to 865)	1509(969 to 2161)	1.26(0.68 to 1.98)	1.21(0.86 to 1.56)	1.73(1.12 to 2.49)	0.43(0.06 to 0.89)
Spain	Liver cancer due to hepatitis B	241(167 to 339)	579(362 to 899)	1.41(0.79 to 2.21)	0.47(0.33 to 0.65)	0.74(0.47 to 1.14)	0.58(0.17 to 1.1)
Spain	Liver cancer due to hepatitis C	1041(823 to 1278)	2839(2022 to 3751)	1.73(1.11 to 2.49)	1.88(1.49 to 2.3)	2.97(2.07 to 3.98)	0.58(0.22 to 1.03)
Spain	Liver cancer due to NASH	101(68 to 147)	300(186 to 456)	1.96(1.22 to 2.87)	0.18(0.13 to 0.27)	0.32(0.2 to 0.49)	0.73(0.29 to 1.28)
Spain	Liver cancer due to other causes	81(59 to 112)	184(116 to 270)	1.25(0.69 to 1.94)	0.17(0.13 to 0.23)	0.25(0.17 to 0.37)	0.46(0.13 to 0.9)
Sri Lanka	Liver cancer	222(194 to 254)	711(512 to 953)	2.2(1.21 to 3.4)	2.06(1.81 to 2.35)	2.8(2.06 to 3.72)	0.36(-0.06 to 0.85)
Sri Lanka	Liver cancer due to alcohol use	43(29 to 62)	180(110 to 276)	3.15(1.8 to 5)	0.42(0.28 to 0.59)	0.69(0.43 to 1.05)	0.65(0.11 to 1.39)
Sri Lanka	Liver cancer due to hepatitis B	99(76 to 126)	264(170 to 386)	1.66(0.8 to 2.74)	0.85(0.64 to 1.1)	1.01(0.67 to 1.47)	0.19(-0.18 to 0.69)
Sri Lanka	Liver cancer due to hepatitis C	50(33 to 68)	173(110 to 259)	2.47(1.45 to 3.9)	0.53(0.37 to 0.71)	0.72(0.46 to 1.04)	0.34(-0.04 to 0.87)
Sri Lanka	Liver cancer due to NASH	18(13 to 26)	68(42 to 108)	2.71(1.56 to 4.26)	0.18(0.12 to 0.26)	0.28(0.17 to 0.43)	0.52(0.06 to 1.12)
Sri Lanka	Liver cancer due to other causes	11(8 to 18)	25(15 to 38)	1.23(0.52 to 2.23)	0.08(0.06 to 0.12)	0.11(0.07 to 0.16)	0.3(-0.08 to 0.77)
Sudan	Liver cancer	330(195 to 495)	674(393 to 1048)	1.04(0.39 to 1.97)	3.53(2.06 to 5.36)	3.7(2.19 to	0.05(-0.27 to 0.54)

						5.76)	
Sudan	Liver cancer due to alcohol use	42(21 to 81)	91(47 to 171)	1.16(0.41 to 2.49)	0.46(0.23 to 0.87)	0.53(0.28 to 1)	0.15(-0.25 to 0.86)
Sudan	Liver cancer due to hepatitis B	127(75 to 198)	247(133 to 407)	0.94(0.25 to 2.02)	1.3(0.76 to 2.03)	1.24(0.68 to 2.05)	-0.04(-0.36 to 0.5)
Sudan	Liver cancer due to hepatitis C	95(51 to 147)	182(102 to 292)	0.92(0.32 to 1.78)	1.12(0.61 to 1.73)	1.12(0.64 to 1.78)	0(-0.3 to 0.44)
Sudan	Liver cancer due to NASH	32(16 to 52)	86(49 to 138)	1.71(0.82 to 2.97)	0.36(0.18 to 0.59)	0.5(0.29 to 0.8)	0.4(-0.05 to 1.01)
Sudan	Liver cancer due to other causes	35(20 to 51)	68(42 to 104)	0.97(0.36 to 1.79)	0.29(0.16 to 0.43)	0.3(0.17 to 0.48)	0.04(-0.28 to 0.44)
Suriname	Liver cancer	17(15 to 19)	16(12 to 19)	-0.09(-0.27 to 0.15)	6.45(5.76 to 7.18)	2.6(2.09 to 3.23)	-0.6(-0.68 to -0.49)
Suriname	Liver cancer due to alcohol use	5(4 to 7)	5(4 to 8)	-0.01(-0.23 to 0.27)	2.14(1.54 to 2.8)	0.91(0.64 to 1.27)	-0.58(-0.67 to -0.46)
Suriname	Liver cancer due to hepatitis B	5(4 to 7)	4(3 to 6)	-0.18(-0.37 to 0.05)	1.92(1.42 to 2.56)	0.71(0.49 to 1)	-0.63(-0.71 to -0.52)
Suriname	Liver cancer due to hepatitis C	4(2 to 5)	3(2 to 5)	-0.09(-0.29 to 0.15)	1.45(1.01 to 1.96)	0.56(0.38 to 0.8)	-0.61(-0.69 to -0.51)
Suriname	Liver cancer due to NASH	1(1 to 2)	1(1 to 2)	0.13(-0.13 to 0.48)	0.5(0.34 to 0.72)	0.25(0.17 to 0.36)	-0.51(-0.62 to -0.36)
Suriname	Liver cancer due to other causes	1(1 to 2)	1(1 to 1)	-0.22(-0.41 to 0.03)	0.44(0.32 to 0.59)	0.17(0.12 to 0.24)	-0.61(-0.7 to -0.5)
Sweden	Liver cancer	351(332 to 369)	595(501 to 697)	0.7(0.43 to 1.01)	2.41(2.29 to 2.53)	3.04(2.56 to 3.59)	0.26(0.06 to 0.5)

Sweden	Liver cancer due to alcohol use	140(121 to 158)	263(209 to 327)	0.88(0.55 to 1.33)	0.95(0.83 to 1.08)	1.35(1.07 to 1.69)	0.42(0.16 to 0.76)
Sweden	Liver cancer due to hepatitis B	21(16 to 27)	34(24 to 45)	0.63(0.35 to 0.97)	0.16(0.13 to 0.21)	0.2(0.15 to 0.27)	0.23(0.02 to 0.5)
Sweden	Liver cancer due to hepatitis C	141(120 to 163)	217(172 to 266)	0.54(0.32 to 0.79)	0.92(0.78 to 1.07)	1.04(0.82 to 1.28)	0.13(-0.04 to 0.31)
Sweden	Liver cancer due to NASH	26(22 to 32)	46(36 to 57)	0.75(0.47 to 1.05)	0.17(0.14 to 0.21)	0.22(0.18 to 0.28)	0.29(0.09 to 0.51)
Sweden	Liver cancer due to other causes	23(20 to 27)	35(27 to 43)	0.49(0.27 to 0.73)	0.2(0.18 to 0.23)	0.22(0.18 to 0.27)	0.11(-0.04 to 0.29)
Switzerland	Liver cancer	266(251 to 281)	959(738 to 1244)	2.6(1.76 to 3.75)	2.64(2.48 to 2.78)	5.79(4.44 to 7.59)	1.19(0.66 to 1.9)
Switzerland	Liver cancer due to alcohol use	117(90 to 142)	397(266 to 560)	2.38(1.5 to 3.5)	1.16(0.89 to 1.4)	2.4(1.61 to 3.41)	1.07(0.52 to 1.77)
Switzerland	Liver cancer due to hepatitis B	35(24 to 51)	126(78 to 195)	2.58(1.66 to 3.82)	0.38(0.26 to 0.53)	0.84(0.53 to 1.29)	1.24(0.66 to 2.02)
Switzerland	Liver cancer due to hepatitis C	88(63 to 115)	340(229 to 478)	2.88(2.01 to 4.14)	0.83(0.6 to 1.09)	1.93(1.29 to 2.73)	1.33(0.79 to 2.07)
Switzerland	Liver cancer due to NASH	13(9 to 20)	55(34 to 87)	3.11(2.13 to 4.42)	0.13(0.09 to 0.18)	0.32(0.2 to 0.5)	1.51(0.9 to 2.31)
Switzerland	Liver cancer due to other causes	12(9 to 17)	41(25 to 62)	2.32(1.44 to 3.37)	0.15(0.11 to 0.2)	0.3(0.19 to 0.42)	0.99(0.5 to 1.6)
Syrian Arab Republic	Liver cancer	259(196 to 326)	535(400 to 711)	1.07(0.4 to 2.17)	4.89(3.64 to 6.23)	4.5(3.42 to 5.9)	-0.08(-0.38 to 0.41)
Syrian Arab Republic	Liver cancer due to alcohol use	23(14 to 35)	49(28 to 78)	1.15(0.38 to 2.45)	0.45(0.27 to 0.69)	0.41(0.23 to 0.64)	-0.1(-0.43 to 0.43)

Syrian Arab Republic	Liver cancer due to hepatitis B	105(75 to 145)	194(129 to 282)	0.85(0.22 to 1.88)	1.8(1.24 to 2.54)	1.47(0.98 to 2.12)	-0.18(-0.46 to 0.26)
Syrian Arab Republic	Liver cancer due to hepatitis C	83(54 to 118)	186(124 to 264)	1.24(0.5 to 2.43)	1.8(1.18 to 2.55)	1.73(1.18 to 2.42)	-0.04(-0.36 to 0.47)
Syrian Arab Republic	Liver cancer due to NASH	24(16 to 36)	64(40 to 98)	1.63(0.73 to 3.16)	0.49(0.31 to 0.76)	0.56(0.36 to 0.85)	0.15(-0.24 to 0.82)
Syrian Arab Republic	Liver cancer due to other causes	24(17 to 33)	42(28 to 62)	0.77(0.21 to 1.59)	0.34(0.23 to 0.5)	0.33(0.22 to 0.48)	-0.04(-0.35 to 0.43)
Taiwan (Province of China)	Liver cancer	2118(2030 to 2208)	3356(2609 to 4335)	0.58(0.22 to 1.04)	12.26(11.74 to 12.77)	8.65(6.73 to 11.15)	-0.29(-0.45 to -0.09)
Taiwan (Province of China)	Liver cancer due to alcohol use	207(135 to 302)	308(183 to 482)	0.49(0.1 to 1.01)	1.21(0.8 to 1.76)	0.77(0.46 to 1.21)	-0.36(-0.53 to -0.14)
Taiwan (Province of China)	Liver cancer due to hepatitis B	1199(1020 to 1372)	1464(1044 to 2003)	0.22(-0.07 to 0.58)	6.67(5.66 to 7.69)	3.84(2.75 to 5.24)	-0.42(-0.56 to -0.26)
Taiwan (Province of China)	Liver cancer due to hepatitis C	501(365 to 646)	1159(796 to 1593)	1.31(0.77 to 2.02)	3.16(2.32 to 3.99)	2.9(1.97 to 4)	-0.08(-0.29 to 0.19)
Taiwan (Province of China)	Liver cancer due to NASH	103(72 to 148)	266(170 to 404)	1.59(0.9 to 2.5)	0.62(0.43 to 0.89)	0.68(0.43 to 1.03)	0.1(-0.19 to 0.47)
Taiwan (Province of China)	Liver cancer due to other causes	108(79 to 146)	159(102 to 241)	0.48(0.07 to 0.96)	0.6(0.44 to 0.83)	0.46(0.3 to 0.66)	-0.25(-0.45 to 0)
Tajikistan	Liver cancer	34(30 to 38)	174(139 to 219)	4.16(3.01 to 5.51)	1.04(0.94 to 1.15)	3.7(2.96 to 4.66)	2.56(1.79 to 3.46)

Tajikistan	Liver cancer due to alcohol use	6(4 to 8)	38(25 to 55)	5.66(4 to 7.84)	0.21(0.14 to 0.29)	0.82(0.53 to 1.2)	2.93(2 to 4.16)
Tajikistan	Liver cancer due to hepatitis B	6(4 to 8)	38(27 to 54)	5.48(3.92 to 7.63)	0.18(0.13 to 0.25)	0.62(0.42 to 0.88)	2.36(1.57 to 3.34)
Tajikistan	Liver cancer due to hepatitis C	13(10 to 16)	75(55 to 101)	4.91(3.53 to 6.58)	0.48(0.37 to 0.59)	1.86(1.39 to 2.44)	2.91(2.03 to 3.97)
Tajikistan	Liver cancer due to NASH	1(1 to 2)	9(6 to 14)	6.48(4.51 to 8.85)	0.05(0.03 to 0.07)	0.22(0.14 to 0.32)	3.7(2.54 to 5.15)
Tajikistan	Liver cancer due to other causes	8(6 to 12)	13(9 to 20)	0.57(0.08 to 1.49)	0.12(0.09 to 0.17)	0.17(0.12 to 0.26)	0.44(0 to 1.13)
Thailand	Liver cancer	7869(6830 to 9009)	24828(18287 to 33055)	2.16(1.27 to 3.34)	20.81(18.13 to 23.74)	24.18(17.89 to 32.01)	0.16(-0.16 to 0.6)
Thailand	Liver cancer due to alcohol use	1892(1295 to 2620)	7426(4788 to 11075)	2.92(1.73 to 4.52)	5.2(3.54 to 7.14)	7.16(4.64 to 10.56)	0.38(-0.02 to 0.94)
Thailand	Liver cancer due to hepatitis B	3988(3146 to 4910)	10264(6987 to 14643)	1.57(0.79 to 2.61)	9.72(7.66 to 12.07)	9.97(6.88 to 14.14)	0.03(-0.28 to 0.42)
Thailand	Liver cancer due to hepatitis C	1220(816 to 1710)	4113(2573 to 6175)	2.37(1.43 to 3.64)	3.79(2.6 to 5.22)	4.06(2.57 to 6.04)	0.07(-0.22 to 0.46)
Thailand	Liver cancer due to NASH	520(356 to 741)	2375(1491 to 3588)	3.57(2.26 to 5.48)	1.5(1.03 to 2.14)	2.34(1.48 to 3.51)	0.56(0.13 to 1.2)
Thailand	Liver cancer due to other causes	249(171 to 339)	651(395 to 1006)	1.62(0.81 to 2.65)	0.62(0.41 to 0.86)	0.65(0.4 to 0.98)	0.05(-0.25 to 0.45)
Timor-Leste	Liver cancer	23(15 to 33)	51(33 to 72)	1.19(0.43 to 2.27)	7.47(5.02 to 10.41)	6.19(4.18 to 8.57)	-0.17(-0.43 to 0.19)

Timor-Leste	Liver cancer due to alcohol use	4(2 to 6)	10(5 to 17)	1.72(0.79 to 3.06)	1.33(0.75 to 2.12)	1.24(0.67 to 2.03)	-0.07(-0.36 to 0.35)
Timor-Leste	Liver cancer due to hepatitis B	12(7 to 18)	22(12 to 33)	0.84(0.11 to 1.8)	3.25(2.01 to 5.05)	2.49(1.38 to 3.82)	-0.23(-0.51 to 0.14)
Timor-Leste	Liver cancer due to hepatitis C	5(3 to 8)	13(8 to 20)	1.56(0.74 to 2.8)	2.08(1.3 to 3.15)	1.73(1.12 to 2.52)	-0.17(-0.41 to 0.16)
Timor-Leste	Liver cancer due to NASH	2(1 to 2)	4(2 to 6)	1.63(0.72 to 2.89)	0.56(0.34 to 0.89)	0.52(0.31 to 0.79)	-0.08(-0.37 to 0.3)
Timor-Leste	Liver cancer due to other causes	1(1 to 2)	2(1 to 3)	0.85(-0.03 to 2.03)	0.24(0.15 to 0.38)	0.21(0.13 to 0.31)	-0.15(-0.47 to 0.29)
Togo	Liver cancer	87(68 to 110)	192(144 to 252)	1.22(0.56 to 2.1)	6.4(5.11 to 8.12)	4.95(3.83 to 6.37)	-0.23(-0.45 to 0.07)
Togo	Liver cancer due to alcohol use	13(8 to 21)	32(20 to 49)	1.38(0.62 to 2.44)	1.1(0.68 to 1.66)	0.88(0.56 to 1.32)	-0.19(-0.44 to 0.15)
Togo	Liver cancer due to hepatitis B	47(35 to 62)	100(70 to 139)	1.13(0.43 to 2.08)	3.24(2.34 to 4.31)	2.31(1.64 to 3.19)	-0.29(-0.51 to 0.01)
Togo	Liver cancer due to hepatitis C	14(9 to 20)	31(19 to 45)	1.25(0.62 to 2.15)	1.26(0.83 to 1.76)	1.01(0.65 to 1.42)	-0.2(-0.41 to 0.1)
Togo	Liver cancer due to NASH	6(4 to 9)	17(11 to 26)	1.79(0.99 to 2.87)	0.49(0.33 to 0.72)	0.48(0.32 to 0.75)	-0.01(-0.28 to 0.36)
Togo	Liver cancer due to other causes	7(5 to 9)	13(8 to 18)	0.91(0.3 to 1.68)	0.32(0.22 to 0.44)	0.27(0.18 to 0.38)	-0.16(-0.41 to 0.15)
Tokelau	Liver cancer	0(0 to 0)	0(0 to 0)	-0.13(-0.38 to 0.21)	8.36(6.2 to 10.95)	7.32(5.29 to 9.9)	-0.12(-0.37 to 0.21)

Tokelau	Liver cancer due to alcohol use	0(0 to 0)	0(0 to 0)	-0.07(-0.34 to 0.31)	0.91(0.53 to 1.42)	0.86(0.49 to 1.31)	-0.06(-0.32 to 0.3)
Tokelau	Liver cancer due to hepatitis B	0(0 to 0)	0(0 to 0)	-0.09(-0.37 to 0.27)	4.3(2.9 to 6.05)	3.8(2.5 to 5.41)	-0.12(-0.39 to 0.23)
Tokelau	Liver cancer due to hepatitis C	0(0 to 0)	0(0 to 0)	-0.25(-0.47 to 0.06)	2.05(1.31 to 2.92)	1.63(1.06 to 2.31)	-0.21(-0.44 to 0.12)
Tokelau	Liver cancer due to NASH	0(0 to 0)	0(0 to 0)	0.02(-0.29 to 0.48)	0.68(0.43 to 1.03)	0.72(0.46 to 1.07)	0.06(-0.27 to 0.53)
Tokelau	Liver cancer due to other causes	0(0 to 0)	0(0 to 0)	-0.24(-0.48 to 0.1)	0.41(0.26 to 0.63)	0.32(0.2 to 0.48)	-0.22(-0.47 to 0.12)
Tonga	Liver cancer	14(9 to 18)	20(14 to 26)	0.44(0.03 to 1.01)	23.33(16.2 to 30.14)	24.33(17.65 to 31.9)	0.04(-0.25 to 0.44)
Tonga	Liver cancer due to alcohol use	2(1 to 3)	3(1 to 4)	0.58(0.1 to 1.25)	2.82(1.58 to 4.4)	3.23(1.89 to 5.01)	0.14(-0.2 to 0.61)
Tonga	Liver cancer due to hepatitis B	8(5 to 11)	11(7 to 15)	0.35(-0.06 to 0.91)	13.12(8.52 to 18.26)	13.05(8.89 to 18.45)	-0.01(-0.31 to 0.4)
Tonga	Liver cancer due to hepatitis C	2(2 to 4)	4(2 to 5)	0.51(0.11 to 1.02)	4.66(2.95 to 6.94)	4.77(3.02 to 7.04)	0.02(-0.24 to 0.36)
Tonga	Liver cancer due to NASH	1(1 to 2)	2(1 to 3)	0.84(0.34 to 1.54)	1.82(1.1 to 2.78)	2.34(1.47 to 3.47)	0.29(-0.05 to 0.76)
Tonga	Liver cancer due to other causes	1(0 to 1)	1(0 to 1)	0.38(-0.01 to 0.97)	0.91(0.56 to 1.37)	0.94(0.58 to 1.45)	0.03(-0.26 to 0.46)
Trinidad and Tobago	Liver cancer	57(53 to 60)	46(35 to 61)	-0.18(-0.4 to 0.08)	6.66(6.23 to 7.09)	2.51(1.9 to 3.29)	-0.62(-0.72 to -0.5)
Trinidad and Tobago	Liver cancer due to alcohol use	18(13 to 23)	16(10 to 23)	-0.11(-0.36 to 0.2)	2.14(1.57 to 2.74)	0.86(0.56 to	-0.6(-0.71 to -0.46)

						1.23)	
Trinidad and Tobago	Liver cancer due to hepatitis B	17(12 to 22)	12(8 to 18)	-0.28(-0.48 to -0.04)	1.88(1.39 to 2.49)	0.64(0.42 to 0.95)	-0.66(-0.75 to -0.54)
Trinidad and Tobago	Liver cancer due to hepatitis C	13(9 to 17)	10(6 to 15)	-0.19(-0.4 to 0.07)	1.59(1.11 to 2.09)	0.57(0.36 to 0.83)	-0.64(-0.73 to -0.54)
Trinidad and Tobago	Liver cancer due to NASH	5(4 to 7)	5(3 to 8)	0(-0.26 to 0.33)	0.59(0.42 to 0.84)	0.27(0.18 to 0.41)	-0.54(-0.67 to -0.39)
Trinidad and Tobago	Liver cancer due to other causes	4(3 to 6)	3(2 to 4)	-0.33(-0.52 to -0.09)	0.46(0.34 to 0.61)	0.17(0.11 to 0.24)	-0.64(-0.74 to -0.51)
Tunisia	Liver cancer	94(73 to 121)	240(170 to 336)	1.55(0.69 to 2.9)	1.87(1.45 to 2.37)	1.94(1.38 to 2.69)	0.03(-0.31 to 0.58)
Tunisia	Liver cancer due to alcohol use	10(5 to 17)	27(14 to 50)	1.73(0.67 to 3.43)	0.2(0.11 to 0.33)	0.22(0.11 to 0.39)	0.09(-0.32 to 0.77)
Tunisia	Liver cancer due to hepatitis B	31(21 to 45)	67(40 to 104)	1.15(0.35 to 2.36)	0.58(0.39 to 0.83)	0.52(0.31 to 0.8)	-0.1(-0.43 to 0.41)
Tunisia	Liver cancer due to hepatitis C	41(28 to 56)	115(77 to 168)	1.8(0.84 to 3.45)	0.88(0.61 to 1.18)	0.95(0.64 to 1.37)	0.08(-0.29 to 0.71)
Tunisia	Liver cancer due to NASH	6(4 to 9)	19(11 to 30)	2.4(1.2 to 4.44)	0.11(0.07 to 0.18)	0.15(0.09 to 0.25)	0.35(-0.11 to 1.15)
Tunisia	Liver cancer due to other causes	6(5 to 9)	12(7 to 18)	0.83(0.21 to 1.75)	0.1(0.07 to 0.14)	0.1(0.06 to 0.15)	-0.02(-0.33 to 0.44)
Turkey	Liver cancer	1349(1080 to 1640)	2768(2204 to 3413)	1.05(0.52 to 1.85)	3.72(2.97 to 4.47)	3.21(2.55 to 3.95)	-0.14(-0.36 to 0.19)
Turkey	Liver cancer due to alcohol use	157(101 to 234)	351(219 to 532)	1.24(0.6 to 2.2)	0.46(0.29 to 0.68)	0.4(0.25 to	-0.11(-0.37 to 0.25)

						0.61)	
Turkey	Liver cancer due to hepatitis B	678(515 to 861)	1156(844 to 1572)	0.71(0.2 to 1.41)	1.77(1.33 to 2.26)	1.29(0.94 to 1.76)	-0.27(-0.48 to 0.03)
Turkey	Liver cancer due to hepatitis C	309(200 to 439)	772(528 to 1032)	1.5(0.8 to 2.65)	0.95(0.61 to 1.33)	0.92(0.63 to 1.23)	-0.02(-0.29 to 0.41)
Turkey	Liver cancer due to NASH	105(69 to 156)	315(211 to 456)	2(1.07 to 3.3)	0.31(0.2 to 0.46)	0.37(0.25 to 0.54)	0.21(-0.15 to 0.73)
Turkey	Liver cancer due to other causes	101(71 to 139)	173(118 to 244)	0.72(0.22 to 1.36)	0.24(0.16 to 0.34)	0.21(0.15 to 0.29)	-0.12(-0.36 to 0.2)
Turkmenistan	Liver cancer	25(23 to 29)	234(184 to 298)	8.24(6.01 to 11.04)	1.17(1.09 to 1.27)	5.55(4.38 to 7.01)	3.75(2.66 to 5.07)
Turkmenistan	Liver cancer due to alcohol use	5(3 to 6)	69(45 to 99)	14.06(10.21 to 18.89)	0.25(0.17 to 0.34)	1.68(1.11 to 2.38)	5.74(4.04 to 7.8)
Turkmenistan	Liver cancer due to hepatitis B	6(5 to 8)	67(45 to 96)	10.29(7.53 to 14.06)	0.27(0.2 to 0.37)	1.45(0.99 to 2.05)	4.35(3.06 to 6.15)
Turkmenistan	Liver cancer due to hepatitis C	8(6 to 10)	67(43 to 94)	7.67(5.56 to 10.27)	0.44(0.34 to 0.55)	1.71(1.12 to 2.35)	2.84(1.94 to 3.96)
Turkmenistan	Liver cancer due to NASH	1(1 to 2)	16(10 to 24)	12.81(9.03 to 17.65)	0.07(0.04 to 0.09)	0.4(0.26 to 0.6)	5.14(3.48 to 7.25)
Turkmenistan	Liver cancer due to other causes	6(5 to 9)	15(11 to 20)	1.51(0.81 to 2.6)	0.14(0.11 to 0.2)	0.31(0.23 to 0.42)	1.28(0.7 to 2.09)
Tuvalu	Liver cancer	1(1 to 1)	1(1 to 1)	0.19(-0.13 to 0.69)	10.4(7.88 to 13.47)	8.47(6.24 to 11.32)	-0.19(-0.41 to 0.15)
Tuvalu	Liver cancer due to alcohol use	0(0 to 0)	0(0 to 0)	0.29(-0.1 to 0.9)	1.15(0.67 to 1.79)	1.02(0.6 to 1.61)	-0.11(-0.37 to 0.29)

Tuvalu	Liver cancer due to hepatitis B	0(0 to 1)	0(0 to 1)	0.16(-0.18 to 0.7)	5.57(3.88 to 7.64)	4.46(3.1 to 6.41)	-0.2(-0.43 to 0.16)
Tuvalu	Liver cancer due to hepatitis C	0(0 to 0)	0(0 to 0)	0.16(-0.16 to 0.64)	2.44(1.55 to 3.62)	1.87(1.16 to 2.73)	-0.23(-0.44 to 0.06)
Tuvalu	Liver cancer due to NASH	0(0 to 0)	0(0 to 0)	0.46(0.04 to 1.06)	0.77(0.49 to 1.19)	0.75(0.47 to 1.13)	-0.03(-0.31 to 0.38)
Tuvalu	Liver cancer due to other causes	0(0 to 0)	0(0 to 0)	0.09(-0.25 to 0.57)	0.48(0.31 to 0.72)	0.37(0.23 to 0.56)	-0.24(-0.48 to 0.1)
Uganda	Liver cancer	318(255 to 388)	963(744 to 1217)	2.03(1.24 to 3.23)	4.48(3.6 to 5.42)	6.06(4.81 to 7.51)	0.35(0.01 to 0.84)
Uganda	Liver cancer due to alcohol use	99(65 to 136)	287(195 to 410)	1.9(1.13 to 3.11)	1.49(1 to 2.03)	1.97(1.34 to 2.74)	0.32(-0.02 to 0.82)
Uganda	Liver cancer due to hepatitis B	102(70 to 144)	298(201 to 426)	1.93(1.08 to 3.34)	1.36(0.94 to 1.95)	1.63(1.1 to 2.36)	0.2(-0.12 to 0.73)
Uganda	Liver cancer due to hepatitis C	53(34 to 76)	165(107 to 234)	2.11(1.28 to 3.21)	0.9(0.59 to 1.25)	1.31(0.87 to 1.85)	0.46(0.1 to 0.93)
Uganda	Liver cancer due to NASH	27(19 to 40)	99(68 to 147)	2.62(1.61 to 4.07)	0.43(0.29 to 0.62)	0.7(0.46 to 1.05)	0.63(0.21 to 1.19)
Uganda	Liver cancer due to other causes	36(24 to 51)	113(76 to 165)	2.12(1.14 to 3.61)	0.3(0.21 to 0.43)	0.45(0.31 to 0.65)	0.49(0.08 to 1.06)
Ukraine	Liver cancer	685(651 to 720)	1930(1641 to 2285)	1.82(1.37 to 2.34)	1(0.94 to 1.07)	2.67(2.27 to 3.19)	1.66(1.21 to 2.19)
Ukraine	Liver cancer due to alcohol use	202(174 to 230)	688(553 to 840)	2.4(1.79 to 3.08)	0.28(0.24 to 0.31)	0.91(0.73 to 1.12)	2.28(1.69 to 2.94)

Ukraine	Liver cancer due to hepatitis B	163(138 to 193)	466(372 to 579)	1.85(1.35 to 2.44)	0.24(0.2 to 0.27)	0.68(0.55 to 0.86)	1.91(1.37 to 2.54)
Ukraine	Liver cancer due to hepatitis C	205(178 to 234)	534(436 to 650)	1.6(1.2 to 2.08)	0.29(0.25 to 0.33)	0.68(0.56 to 0.84)	1.37(1 to 1.8)
Ukraine	Liver cancer due to NASH	52(43 to 62)	155(124 to 188)	1.99(1.54 to 2.53)	0.07(0.06 to 0.09)	0.21(0.17 to 0.25)	1.83(1.4 to 2.36)
Ukraine	Liver cancer due to other causes	62(45 to 84)	87(65 to 115)	0.41(-0.07 to 1.11)	0.13(0.09 to 0.18)	0.19(0.13 to 0.28)	0.44(-0.15 to 1.42)
United Arab Emirates	Liver cancer	21(11 to 37)	219(93 to 496)	9.7(4.8 to 16.88)	4.28(2.15 to 8.14)	4.73(2.01 to 10.82)	0.1(-0.36 to 0.71)
United Arab Emirates	Liver cancer due to alcohol use	2(1 to 5)	23(7 to 61)	9.51(4.45 to 16.32)	0.54(0.21 to 1.22)	0.58(0.19 to 1.57)	0.08(-0.41 to 0.72)
United Arab Emirates	Liver cancer due to hepatitis B	10(5 to 20)	115(48 to 264)	10.19(4.82 to 18.39)	1.55(0.71 to 3.18)	1.73(0.69 to 4.15)	0.12(-0.37 to 0.75)
United Arab Emirates	Liver cancer due to hepatitis C	5(2 to 9)	46(17 to 113)	8.84(4.49 to 14.84)	1.53(0.72 to 3.09)	1.58(0.63 to 3.86)	0.03(-0.39 to 0.56)
United Arab Emirates	Liver cancer due to NASH	2(1 to 3)	22(9 to 54)	12.42(6.27 to 20.81)	0.43(0.2 to 0.87)	0.61(0.24 to 1.52)	0.43(-0.17 to 1.2)
United Arab Emirates	Liver cancer due to other causes	2(1 to 3)	13(6 to 30)	6.69(3.1 to 12.44)	0.24(0.13 to 0.48)	0.24(0.1 to 0.55)	-0.02(-0.41 to 0.49)
United Kingdom	Liver cancer	1787(1722 to 1836)	6201(5143 to 7417)	2.47(1.89 to 3.15)	2.04(1.97 to 2.1)	5.08(4.2 to 6.11)	1.49(1.05 to 1.98)
United Kingdom	Liver cancer due to alcohol use	654(580 to 727)	2296(1802 to 2881)	2.51(1.82 to 3.33)	0.73(0.65 to 0.81)	1.88(1.47 to 2.37)	1.57(1.05 to 2.18)

United Kingdom	Liver cancer due to hepatitis B	246(205 to 293)	804(610 to 1031)	2.27(1.68 to 2.99)	0.31(0.26 to 0.36)	0.75(0.57 to 0.95)	1.43(0.99 to 1.98)
United Kingdom	Liver cancer due to hepatitis C	669(597 to 747)	2341(1911 to 2840)	2.5(1.95 to 3.1)	0.73(0.65 to 0.81)	1.79(1.46 to 2.17)	1.45(1.06 to 1.88)
United Kingdom	Liver cancer due to NASH	113(95 to 133)	453(355 to 568)	3.02(2.39 to 3.75)	0.12(0.11 to 0.14)	0.36(0.28 to 0.45)	1.88(1.42 to 2.42)
United Kingdom	Liver cancer due to other causes	105(92 to 119)	308(248 to 379)	1.93(1.46 to 2.42)	0.15(0.13 to 0.17)	0.31(0.25 to 0.37)	1.04(0.73 to 1.39)
United Republic of Tanzania	Liver cancer	255(203 to 314)	600(464 to 765)	1.36(0.77 to 2.07)	2.03(1.67 to 2.48)	2.25(1.79 to 2.8)	0.11(-0.14 to 0.41)
United Republic of Tanzania	Liver cancer due to alcohol use	57(37 to 83)	147(96 to 214)	1.58(0.91 to 2.42)	0.53(0.35 to 0.77)	0.62(0.41 to 0.91)	0.18(-0.12 to 0.53)
United Republic of Tanzania	Liver cancer due to hepatitis B	71(50 to 100)	163(111 to 235)	1.29(0.67 to 2.09)	0.56(0.39 to 0.81)	0.57(0.38 to 0.82)	0.01(-0.25 to 0.34)
United Republic of Tanzania	Liver cancer due to hepatitis C	46(30 to 63)	114(77 to 159)	1.51(0.96 to 2.23)	0.48(0.33 to 0.65)	0.54(0.36 to 0.74)	0.11(-0.12 to 0.4)
United Republic of Tanzania	Liver cancer due to NASH	25(18 to 36)	72(49 to 102)	1.85(1.18 to 2.75)	0.24(0.17 to 0.34)	0.3(0.2 to 0.43)	0.27(-0.01 to 0.64)
United Republic of Tanzania	Liver cancer due to other causes	56(34 to 89)	104(63 to 171)	0.87(0.1 to 2.07)	0.21(0.15 to 0.29)	0.21(0.15 to 0.3)	0.01(-0.29 to 0.41)
United States of America	Liver cancer	6874(6639 to 7029)	27895(22785 to 33513)	3.06(2.32 to 3.87)	2.22(2.15 to 2.27)	5.23(4.28 to 6.29)	1.35(0.92 to 1.83)
United States of America	Liver cancer due to alcohol use	2022(1789 to 2247)	8928(6833 to 11477)	3.42(2.47 to 4.52)	0.65(0.57 to 0.72)	1.66(1.27 to 2.15)	1.57(1.01 to 2.22)

United States of America	Liver cancer due to hepatitis B	965(839 to 1110)	3443(2613 to 4474)	2.57(1.84 to 3.37)	0.32(0.28 to 0.37)	0.69(0.53 to 0.89)	1.14(0.71 to 1.62)
United States of America	Liver cancer due to hepatitis C	2485(2235 to 2716)	10409(8344 to 12654)	3.19(2.47 to 3.95)	0.78(0.71 to 0.86)	1.88(1.51 to 2.28)	1.4(0.98 to 1.83)
United States of America	Liver cancer due to NASH	661(569 to 767)	2791(2184 to 3465)	3.22(2.53 to 3.99)	0.2(0.18 to 0.24)	0.51(0.4 to 0.63)	1.48(1.07 to 1.93)
United States of America	Liver cancer due to other causes	741(657 to 825)	2325(1857 to 2896)	2.14(1.63 to 2.73)	0.26(0.23 to 0.29)	0.49(0.39 to 0.6)	0.86(0.57 to 1.19)
United States Virgin Islands	Liver cancer	3(3 to 4)	5(4 to 5)	0.32(0 to 0.73)	4.07(3.35 to 4.76)	2.49(2.06 to 2.95)	-0.39(-0.54 to -0.2)
United States Virgin Islands	Liver cancer due to alcohol use	1(1 to 1)	2(1 to 2)	0.55(0.13 to 1.1)	1.26(0.89 to 1.73)	0.86(0.61 to 1.18)	-0.32(-0.5 to -0.08)
United States Virgin Islands	Liver cancer due to hepatitis B	1(1 to 1)	1(1 to 2)	0.06(-0.23 to 0.44)	1.18(0.81 to 1.63)	0.66(0.46 to 0.94)	-0.44(-0.59 to -0.23)
United States Virgin Islands	Liver cancer due to hepatitis C	1(0 to 1)	1(1 to 1)	0.34(0 to 0.74)	0.94(0.64 to 1.32)	0.52(0.35 to 0.72)	-0.44(-0.58 to -0.28)
United States Virgin Islands	Liver cancer due to NASH	0(0 to 0)	1(0 to 1)	0.71(0.27 to 1.34)	0.41(0.27 to 0.58)	0.3(0.21 to 0.43)	-0.26(-0.44 to 0.01)
United States Virgin Islands	Liver cancer due to other causes	0(0 to 0)	0(0 to 0)	-0.06(-0.31 to 0.28)	0.28(0.19 to 0.38)	0.14(0.1 to 0.2)	-0.48(-0.61 to -0.31)
Uruguay	Liver cancer	53(47 to 60)	120(94 to 153)	1.26(0.7 to 1.96)	1.36(1.2 to 1.53)	2.28(1.77 to 2.9)	0.67(0.25 to 1.21)
Uruguay	Liver cancer due to alcohol use	15(11 to 21)	36(24 to 52)	1.35(0.68 to 2.18)	0.39(0.27 to 0.53)	0.7(0.45 to 1)	0.79(0.27 to 1.44)
Uruguay	Liver cancer due to hepatitis B	11(7 to 15)	21(14 to 32)	0.98(0.43 to 1.7)	0.28(0.2 to 0.4)	0.43(0.29 to	0.54(0.1 to 1.11)

						0.66)	
Uruguay	Liver cancer due to hepatitis C	19(14 to 25)	44(30 to 60)	1.31(0.73 to 2)	0.48(0.34 to 0.62)	0.78(0.52 to 1.09)	0.64(0.24 to 1.14)
Uruguay	Liver cancer due to NASH	4(3 to 6)	12(8 to 18)	1.81(1.1 to 2.81)	0.11(0.07 to 0.16)	0.22(0.14 to 0.33)	1.01(0.5 to 1.73)
Uruguay	Liver cancer due to other causes	4(3 to 5)	7(4 to 10)	0.85(0.38 to 1.37)	0.1(0.08 to 0.14)	0.14(0.1 to 0.21)	0.41(0.07 to 0.79)
Uzbekistan	Liver cancer	115(99 to 134)	1346(1098 to 1615)	10.73(8.11 to 13.95)	0.91(0.82 to 1)	6.34(5.27 to 7.46)	5.96(4.62 to 7.48)
Uzbekistan	Liver cancer due to alcohol use	23(16 to 30)	377(252 to 524)	15.73(12.02 to 19.66)	0.21(0.14 to 0.27)	1.78(1.21 to 2.42)	7.66(6.01 to 9.55)
Uzbekistan	Liver cancer due to hepatitis B	27(20 to 36)	387(271 to 548)	13.23(9.96 to 16.93)	0.22(0.16 to 0.29)	1.51(1.05 to 2.13)	5.98(4.57 to 7.6)
Uzbekistan	Liver cancer due to hepatitis C	36(27 to 44)	411(275 to 564)	10.56(8.18 to 13.31)	0.34(0.26 to 0.42)	2.26(1.61 to 3)	5.7(4.54 to 6.99)
Uzbekistan	Liver cancer due to NASH	6(4 to 8)	97(65 to 142)	15.98(11.87 to 21.12)	0.05(0.04 to 0.08)	0.51(0.35 to 0.73)	8.58(6.66 to 11.04)
Uzbekistan	Liver cancer due to other causes	24(14 to 39)	74(50 to 103)	2.11(0.66 to 5.14)	0.1(0.06 to 0.16)	0.29(0.2 to 0.39)	1.9(0.66 to 3.96)
Vanuatu	Liver cancer	6(4 to 10)	16(11 to 23)	1.57(0.73 to 2.87)	9.22(5.88 to 13.77)	9.05(6.21 to 12.85)	-0.02(-0.33 to 0.45)
Vanuatu	Liver cancer due to alcohol use	1(0 to 1)	2(1 to 3)	1.79(0.86 to 3.26)	1.11(0.56 to 1.94)	1.16(0.63 to 1.93)	0.05(-0.29 to 0.57)
Vanuatu	Liver cancer due to hepatitis B	4(2 to 6)	9(6 to 14)	1.47(0.59 to 2.74)	5.11(3.09 to 7.91)	4.86(3.1 to 7.2)	-0.05(-0.38 to 0.44)

Vanuatu	Liver cancer due to hepatitis C	1(1 to 2)	3(2 to 5)	1.61(0.79 to 2.91)	2(1.15 to 3.32)	1.92(1.16 to 2.88)	-0.04(-0.32 to 0.41)
Vanuatu	Liver cancer due to NASH	0(0 to 1)	1(1 to 2)	2.12(1.15 to 3.68)	0.63(0.35 to 1.04)	0.74(0.45 to 1.14)	0.17(-0.19 to 0.72)
Vanuatu	Liver cancer due to other causes	0(0 to 0)	1(0 to 1)	1.53(0.72 to 2.8)	0.37(0.21 to 0.6)	0.38(0.22 to 0.58)	0.02(-0.31 to 0.56)
Venezuela (Bolivarian Republic of)	Liver cancer	617(588 to 646)	654(502 to 855)	0.06(-0.2 to 0.37)	6.36(6.04 to 6.68)	2.29(1.77 to 2.97)	-0.64(-0.73 to -0.54)
Venezuela (Bolivarian Republic of)	Liver cancer due to alcohol use	206(152 to 258)	233(154 to 334)	0.13(-0.15 to 0.51)	2.16(1.57 to 2.72)	0.81(0.54 to 1.15)	-0.63(-0.72 to -0.5)
Venezuela (Bolivarian Republic of)	Liver cancer due to hepatitis B	111(79 to 153)	102(64 to 159)	-0.08(-0.32 to 0.23)	1.04(0.71 to 1.47)	0.34(0.22 to 0.53)	-0.67(-0.75 to -0.56)
Venezuela (Bolivarian Republic of)	Liver cancer due to hepatitis C	215(160 to 270)	227(152 to 319)	0.06(-0.2 to 0.36)	2.36(1.77 to 2.97)	0.81(0.55 to 1.13)	-0.66(-0.74 to -0.56)
Venezuela (Bolivarian Republic of)	Liver cancer due to NASH	42(30 to 59)	54(36 to 85)	0.29(-0.05 to 0.71)	0.44(0.31 to 0.62)	0.19(0.13 to 0.3)	-0.56(-0.67 to -0.43)
Venezuela (Bolivarian Republic of)	Liver cancer due to other causes	43(33 to 56)	37(24 to 53)	-0.15(-0.37 to 0.11)	0.36(0.26 to 0.49)	0.13(0.09 to 0.19)	-0.64(-0.73 to -0.52)
Viet Nam	Liver cancer	1500(1119 to 1893)	2335(1787 to 2945)	0.56(0.06 to 1.3)	3.7(2.77 to 4.63)	2.61(2.03 to 3.26)	-0.3(-0.52 to 0.06)
Viet Nam	Liver cancer due to alcohol use	271(165 to 419)	558(338 to 825)	1.06(0.37 to 2.16)	0.67(0.41 to 1.04)	0.63(0.38 to 0.92)	-0.07(-0.39 to 0.44)

Viet Nam	Liver cancer due to hepatitis B	705(482 to 968)	953(648 to 1341)	0.35(-0.11 to 1.03)	1.69(1.15 to 2.32)	0.99(0.68 to 1.41)	-0.41(-0.61 to -0.11)
Viet Nam	Liver cancer due to hepatitis C	347(192 to 542)	536(308 to 842)	0.54(0.06 to 1.39)	0.91(0.52 to 1.41)	0.65(0.38 to 1.01)	-0.29(-0.51 to 0.11)
Viet Nam	Liver cancer due to NASH	118(73 to 177)	206(129 to 314)	0.74(0.16 to 1.74)	0.3(0.19 to 0.46)	0.24(0.16 to 0.37)	-0.2(-0.45 to 0.25)
Viet Nam	Liver cancer due to other causes	59(41 to 85)	83(55 to 122)	0.4(0.03 to 0.92)	0.13(0.08 to 0.19)	0.1(0.07 to 0.14)	-0.24(-0.45 to 0.09)
Yemen	Liver cancer	156(98 to 229)	416(301 to 569)	1.67(0.87 to 2.93)	3.19(2.03 to 4.67)	3.2(2.32 to 4.33)	0(-0.28 to 0.42)
Yemen	Liver cancer due to alcohol use	9(5 to 17)	28(16 to 47)	1.95(1 to 3.43)	0.2(0.1 to 0.35)	0.22(0.12 to 0.37)	0.13(-0.23 to 0.66)
Yemen	Liver cancer due to hepatitis B	56(32 to 91)	139(88 to 211)	1.5(0.68 to 2.81)	1.03(0.61 to 1.69)	0.96(0.59 to 1.46)	-0.07(-0.36 to 0.39)
Yemen	Liver cancer due to hepatitis C	64(37 to 103)	175(115 to 252)	1.71(0.91 to 2.92)	1.5(0.87 to 2.35)	1.51(1.02 to 2.15)	0(-0.28 to 0.42)
Yemen	Liver cancer due to NASH	10(6 to 17)	34(21 to 53)	2.2(1.18 to 3.76)	0.23(0.12 to 0.39)	0.27(0.17 to 0.43)	0.17(-0.19 to 0.7)
Yemen	Liver cancer due to other causes	16(9 to 26)	41(27 to 59)	1.58(0.73 to 2.93)	0.23(0.13 to 0.36)	0.24(0.15 to 0.35)	0.05(-0.27 to 0.57)
Zambia	Liver cancer	76(56 to 108)	195(149 to 247)	1.57(0.71 to 2.76)	2.37(1.78 to 3.43)	2.75(2.14 to 3.41)	0.16(-0.25 to 0.66)
Zambia	Liver cancer due to alcohol use	17(10 to 29)	48(31 to 67)	1.86(0.7 to 3.3)	0.62(0.37 to 1.09)	0.76(0.51 to 1.07)	0.22(-0.28 to 0.85)

Zambia	Liver cancer due to hepatitis B	23(15 to 35)	62(42 to 87)	1.67(0.7 to 2.97)	0.68(0.45 to 1.06)	0.75(0.51 to 1.07)	0.09(-0.31 to 0.62)
Zambia	Liver cancer due to hepatitis C	13(9 to 20)	36(23 to 51)	1.67(0.78 to 2.83)	0.56(0.36 to 0.84)	0.65(0.43 to 0.91)	0.17(-0.22 to 0.62)
Zambia	Liver cancer due to NASH	8(5 to 12)	24(15 to 35)	1.95(0.96 to 3.36)	0.29(0.19 to 0.44)	0.37(0.24 to 0.53)	0.27(-0.16 to 0.83)
Zambia	Liver cancer due to other causes	15(8 to 25)	26(18 to 37)	0.8(-0.02 to 2.15)	0.22(0.14 to 0.32)	0.22(0.15 to 0.31)	0.01(-0.34 to 0.48)
Zimbabwe	Liver cancer	512(397 to 796)	1032(760 to 1405)	1.01(0.37 to 1.8)	11.76(9.21 to 17.91)	13.64(10.27 to 18.13)	0.16(-0.21 to 0.6)
Zimbabwe	Liver cancer due to alcohol use	84(47 to 162)	153(91 to 252)	0.81(0.22 to 1.58)	1.98(1.14 to 3.8)	2.07(1.25 to 3.38)	0.05(-0.29 to 0.48)
Zimbabwe	Liver cancer due to hepatitis B	223(150 to 380)	400(265 to 612)	0.79(0.21 to 1.55)	4.65(3.11 to 7.83)	4.58(3.01 to 7.03)	-0.02(-0.33 to 0.38)
Zimbabwe	Liver cancer due to hepatitis C	139(95 to 202)	325(215 to 462)	1.33(0.61 to 2.27)	3.69(2.59 to 5.26)	5.02(3.37 to 7.07)	0.36(-0.05 to 0.91)
Zimbabwe	Liver cancer due to NASH	37(24 to 61)	89(57 to 133)	1.37(0.62 to 2.39)	0.91(0.57 to 1.5)	1.25(0.8 to 1.9)	0.37(-0.06 to 0.94)
Zimbabwe	Liver cancer due to other causes	28(19 to 42)	65(43 to 96)	1.32(0.56 to 2.35)	0.53(0.35 to 0.83)	0.72(0.46 to 1.1)	0.36(-0.09 to 0.94)

Table S6. The disability-adjusted life years (DALYs) of liver cancer and underlying etiologies between 1990 and 2019 at national level, both sexes

regions	Causes	Cases in 1990	Cases in 2019	Change in absolute number (95% UI)	ASRs in 1990	ASRs in 2019	change in ASR per 100 000 population (95% UI)
Afghanistan	Liver cancer	25402(19222 to 32534)	47404(35299 to 63438)	0.87(0.33 to 1.64)	324.06(247.55 to 417.52)	271.95(207.43 to 351.12)	-0.16(-0.39 to 0.17)
Afghanistan	Liver cancer due to alcohol use	1852(1073 to 2883)	3013(1828 to 4773)	0.63(0.11 to 1.38)	24.53(14.49 to 37.66)	21.25(12.96 to 32.72)	-0.13(-0.38 to 0.21)
Afghanistan	Liver cancer due to hepatitis B	10924(7641 to 15023)	21929(15280 to 31243)	1.01(0.38 to 1.96)	137(96.52 to 187.8)	111.25(77.86 to 157.05)	-0.19(-0.42 to 0.16)
Afghanistan	Liver cancer due to hepatitis C	8136(5302 to 11578)	12474(8130 to 17989)	0.53(0.05 to 1.24)	110.39(74.53 to 154.58)	92.07(61.13 to 130.59)	-0.17(-0.4 to 0.17)
Afghanistan	Liver cancer due to NASH	1793(1176 to 2712)	3692(2361 to 5531)	1.06(0.41 to 2.1)	23.15(15.01 to 34.67)	22.47(14.32 to 33.14)	-0.03(-0.31 to 0.39)
Afghanistan	Liver cancer due to other causes	2697(1852 to 3812)	6296(4390 to 8905)	1.33(0.6 to 2.43)	28.99(19.5 to 41.55)	24.91(16.94 to 35.9)	-0.14(-0.4 to 0.24)
Albania	Liver cancer	6082(5662 to 6511)	6592(4753 to 8852)	0.08(-0.22 to 0.46)	271.62(253.28 to 290.59)	160.49(116.87 to 213.76)	-0.41(-0.57 to -0.2)
Albania	Liver cancer due to alcohol use	1692(1209 to 2204)	2473(1551 to 3713)	0.46(0.02 to 1.03)	78.59(56.9 to 102.21)	57.73(36.41 to 85.09)	-0.27(-0.49 to 0.01)
Albania	Liver cancer due to hepatitis B	2140(1643 to 2726)	1786(1132 to 2673)	-0.17(-0.42 to 0.14)	88.88(66.86 to 115.37)	46.12(30.09 to 68.18)	-0.48(-0.63 to -0.29)
Albania	Liver cancer due to hepatitis C	1440(995 to 1931)	1567(977 to 2348)	0.09(-0.23 to 0.48)	70.84(49.72 to 94.13)	36.02(22.59 to 54.01)	-0.49(-0.63 to -0.31)

Albania	Liver cancer due to NASH	396(287 to 549)	493(304 to 768)	0.24(-0.12 to 0.72)	18.33(13 to 25.58)	11.77(7.35 to 18.15)	-0.36(-0.54 to -0.1)
Albania	Liver cancer due to other causes	414(326 to 515)	274(183 to 404)	-0.34(-0.53 to -0.1)	14.98(11.5 to 19.21)	8.83(6.34 to 12.19)	-0.41(-0.56 to -0.23)
Algeria	Liver cancer	6209(5046 to 7556)	17664(13736 to 22423)	1.84(1.11 to 2.79)	42.72(34.66 to 52.73)	49.64(38.67 to 62.81)	0.16(-0.15 to 0.56)
Algeria	Liver cancer due to alcohol use	473(282 to 728)	1696(984 to 2619)	2.59(1.48 to 4.1)	3.67(2.24 to 5.58)	4.94(2.9 to 7.68)	0.34(-0.06 to 0.92)
Algeria	Liver cancer due to hepatitis B	2435(1768 to 3220)	6916(4820 to 9537)	1.84(1.01 to 3.01)	16.71(12.01 to 22.22)	18.21(12.75 to 25.33)	0.09(-0.23 to 0.53)
Algeria	Liver cancer due to hepatitis C	1674(1125 to 2354)	5421(3680 to 7536)	2.24(1.31 to 3.41)	14.15(9.84 to 19.16)	16.56(11.5 to 22.91)	0.17(-0.15 to 0.55)
Algeria	Liver cancer due to NASH	439(292 to 660)	1766(1151 to 2622)	3.03(1.81 to 4.66)	3.36(2.22 to 4.95)	5.14(3.32 to 7.72)	0.53(0.06 to 1.11)
Algeria	Liver cancer due to other causes	1188(884 to 1567)	1864(1382 to 2464)	0.57(0.11 to 1.16)	4.83(3.64 to 6.24)	4.78(3.55 to 6.38)	-0.01(-0.26 to 0.32)
American Samoa	Liver cancer	42(31 to 52)	99(79 to 125)	1.37(0.77 to 2.24)	150.18(113.56 to 185.64)	189.82(153.06 to 237.59)	0.26(-0.06 to 0.71)
American Samoa	Liver cancer due to alcohol use	4(2 to 6)	10(6 to 16)	1.55(0.86 to 2.6)	15.63(9.29 to 24.21)	19.8(12.32 to 29.57)	0.27(-0.07 to 0.73)
American Samoa	Liver cancer due to hepatitis B	25(17 to 33)	58(44 to 79)	1.34(0.68 to 2.28)	84.69(58.51 to 112.3)	109.31(82.71 to 145.44)	0.29(-0.06 to 0.8)
American Samoa	Liver cancer due to hepatitis C	6(4 to 9)	15(10 to 22)	1.45(0.87 to 2.3)	27.95(18.06 to 39.74)	31.67(20.68 to 44.94)	0.13(-0.13 to 0.5)
American Samoa	Liver cancer due to NASH	4(2 to 5)	10(7 to 14)	1.78(1.04 to 2.82)	14.11(9.18 to 20.36)	19.94(13.76 to	0.41(0.06 to 0.9)

						28.26)	
American Samoa	Liver cancer due to other causes	3(2 to 4)	5(3 to 7)	0.73(0.26 to 1.35)	7.8(5.45 to 10.91)	9.09(6.18 to 12.99)	0.17(-0.15 to 0.61)
Andorra	Liver cancer	119(86 to 164)	282(210 to 371)	1.37(0.54 to 2.5)	208.5(153 to 284.92)	207.57(155.78 to 273.08)	0(-0.35 to 0.47)
Andorra	Liver cancer due to alcohol use	51(34 to 76)	115(76 to 161)	1.24(0.46 to 2.42)	87.37(58.63 to 130.57)	83.73(55.63 to 117.54)	-0.04(-0.37 to 0.45)
Andorra	Liver cancer due to hepatitis B	19(12 to 30)	41(25 to 63)	1.09(0.36 to 2.2)	32.55(19.54 to 49.93)	30.82(19.18 to 47.12)	-0.05(-0.39 to 0.43)
Andorra	Liver cancer due to hepatitis C	36(22 to 55)	95(61 to 136)	1.66(0.71 to 2.93)	64.44(40.61 to 97.73)	67.87(43.96 to 97.65)	0.05(-0.31 to 0.55)
Andorra	Liver cancer due to NASH	6(4 to 10)	18(11 to 28)	1.93(0.88 to 3.41)	10.72(6.6 to 16.92)	13.04(7.99 to 20.62)	0.22(-0.21 to 0.8)
Andorra	Liver cancer due to other causes	7(4 to 10)	14(9 to 21)	1.02(0.3 to 2.14)	13.42(9.11 to 18.79)	12.11(8.02 to 17.65)	-0.1(-0.4 to 0.37)
Angola	Liver cancer	4636(3371 to 6286)	11277(8099 to 15215)	1.43(0.5 to 2.74)	73.17(56.99 to 93.74)	66.04(51.49 to 84)	-0.1(-0.36 to 0.28)
Angola	Liver cancer due to alcohol use	390(229 to 616)	1309(813 to 1959)	2.36(1.23 to 4.1)	9.06(5.42 to 14.18)	10.46(6.66 to 15.49)	0.15(-0.22 to 0.7)
Angola	Liver cancer due to hepatitis B	1174(815 to 1646)	2956(1951 to 4213)	1.52(0.61 to 2.81)	19.86(13.62 to 28.09)	16.7(11.16 to 24.08)	-0.16(-0.43 to 0.23)
Angola	Liver cancer due to hepatitis C	1211(823 to 1664)	2991(2033 to 4130)	1.47(0.7 to 2.54)	30.2(21.16 to 40.94)	26.12(18.64 to 35.18)	-0.14(-0.38 to 0.21)
Angola	Liver cancer due to NASH	179(115 to 273)	583(380 to 880)	2.25(1.11 to 3.89)	3.75(2.41 to 5.64)	4.2(2.78 to 6.33)	0.12(-0.23 to 0.6)

Angola	Liver cancer due to other causes	1681(903 to 2971)	3438(1797 to 5963)	1.05(-0.19 to 3.56)	10.3(6.37 to 17.14)	8.56(5.07 to 13.88)	-0.17(-0.61 to 0.61)
Antigua and Barbuda	Liver cancer	102(92 to 112)	67(56 to 79)	-0.34(-0.46 to -0.2)	197.08(178.57 to 218.41)	64.64(54.4 to 76.32)	-0.67(-0.73 to -0.6)
Antigua and Barbuda	Liver cancer due to alcohol use	31(22 to 41)	23(16 to 31)	-0.26(-0.4 to -0.08)	60.84(42.89 to 80.93)	22.1(15.44 to 29.76)	-0.64(-0.71 to -0.55)
Antigua and Barbuda	Liver cancer due to hepatitis B	35(27 to 46)	20(14 to 28)	-0.43(-0.54 to -0.29)	69.49(52.69 to 91.61)	18.6(13.51 to 25.62)	-0.73(-0.78 to -0.67)
Antigua and Barbuda	Liver cancer due to hepatitis C	20(14 to 28)	13(9 to 19)	-0.36(-0.48 to -0.22)	38.61(25.8 to 53.09)	13.03(8.77 to 18.75)	-0.66(-0.72 to -0.59)
Antigua and Barbuda	Liver cancer due to NASH	7(5 to 10)	6(4 to 8)	-0.21(-0.38 to 0)	13.73(9.75 to 19.49)	5.69(3.91 to 8.06)	-0.59(-0.67 to -0.48)
Antigua and Barbuda	Liver cancer due to other causes	8(6 to 10)	5(3 to 7)	-0.39(-0.51 to -0.23)	14.41(10.73 to 19.04)	5.23(3.81 to 6.94)	-0.64(-0.71 to -0.54)
Argentina	Liver cancer	11515(10058 to 13058)	25035(23210 to 27109)	1.17(0.86 to 1.57)	35.14(30.73 to 39.92)	47.62(44.11 to 51.38)	0.35(0.16 to 0.6)
Argentina	Liver cancer due to alcohol use	3826(2722 to 5059)	8342(6064 to 10709)	1.18(0.79 to 1.69)	11.62(8.28 to 15.31)	15.66(11.36 to 20.15)	0.35(0.1 to 0.67)
Argentina	Liver cancer due to hepatitis B	2260(1574 to 3206)	4778(3368 to 6712)	1.11(0.74 to 1.57)	6.93(4.85 to 9.75)	9.28(6.56 to 12.98)	0.34(0.1 to 0.63)
Argentina	Liver cancer due to hepatitis C	3327(2362 to 4513)	7923(5755 to 10189)	1.38(1.04 to 1.86)	10.27(7.35 to 13.8)	14.69(10.64 to 18.95)	0.43(0.22 to 0.7)
Argentina	Liver cancer due to NASH	744(512 to 1093)	2107(1458 to 2932)	1.83(1.36 to 2.52)	2.29(1.6 to 3.38)	3.92(2.72 to 5.43)	0.71(0.42 to 1.12)

Argentina	Liver cancer due to other causes	1359(1111 to 1669)	1885(1450 to 2426)	0.39(0.17 to 0.63)	4.02(3.29 to 4.94)	4.06(3.18 to 5.18)	0.01(-0.12 to 0.16)
Armenia	Liver cancer	882(804 to 955)	6424(5263 to 7736)	6.28(4.88 to 7.99)	29.76(27.57 to 32.02)	158.11(129.01 to 190.25)	4.31(3.27 to 5.54)
Armenia	Liver cancer due to alcohol use	172(119 to 231)	1935(1321 to 2623)	10.25(7.91 to 13.11)	6.19(4.38 to 8.26)	46.57(32.39 to 62.49)	6.52(4.98 to 8.42)
Armenia	Liver cancer due to hepatitis B	202(153 to 262)	1527(1024 to 2181)	6.55(5.02 to 8.28)	6.71(5.03 to 8.74)	38.33(26.04 to 53.64)	4.71(3.58 to 6.05)
Armenia	Liver cancer due to hepatitis C	234(169 to 299)	2215(1549 to 3004)	8.45(6.69 to 10.53)	8.86(6.57 to 11.13)	53.05(37.48 to 70.79)	4.99(3.92 to 6.25)
Armenia	Liver cancer due to NASH	37(26 to 52)	463(313 to 677)	11.35(8.66 to 14.56)	1.39(0.97 to 1.97)	11.34(7.76 to 16.37)	7.16(5.45 to 9.36)
Armenia	Liver cancer due to other causes	236(166 to 294)	285(187 to 409)	0.21(-0.29 to 1.12)	6.6(4.71 to 8.17)	8.82(5.68 to 13.1)	0.34(-0.24 to 1.38)
Australia	Liver cancer	9351(8994 to 9762)	37190(33939 to 40789)	2.98(2.59 to 3.37)	48.99(47.07 to 51.25)	98.86(90.16 to 108.43)	1.02(0.82 to 1.22)
Australia	Liver cancer due to alcohol use	3972(3066 to 4816)	14765(10976 to 18441)	2.72(2.26 to 3.2)	20.45(15.88 to 24.85)	38.84(28.95 to 48.57)	0.9(0.68 to 1.14)
Australia	Liver cancer due to hepatitis B	1664(1185 to 2237)	5500(3794 to 7872)	2.3(1.85 to 2.77)	8.86(6.34 to 11.96)	15.83(11.25 to 22.2)	0.79(0.55 to 1.04)
Australia	Liver cancer due to hepatitis C	2438(1757 to 3225)	11236(8049 to 14778)	3.61(3.16 to 4.15)	12.54(9.04 to 16.63)	28.2(20.05 to 37.4)	1.25(1.03 to 1.52)
Australia	Liver cancer due to NASH	650(464 to 906)	3753(2626 to 5123)	4.77(3.81 to 5.83)	3.35(2.38 to 4.59)	9.64(6.94 to 13.05)	1.88(1.4 to 2.4)

Australia	Liver cancer due to other causes	627(511 to 783)	1935(1418 to 2589)	2.09(1.57 to 2.57)	3.79(3.17 to 4.63)	6.35(4.92 to 8.17)	0.67(0.43 to 0.9)
Austria	Liver cancer	8767(8326 to 9217)	16169(14540 to 18031)	0.84(0.65 to 1.08)	81.09(76.92 to 85.39)	100.7(90.21 to 112.42)	0.24(0.11 to 0.4)
Austria	Liver cancer due to alcohol use	4760(3953 to 5555)	8774(7084 to 10583)	0.84(0.6 to 1.12)	43.78(36.26 to 51.08)	54.26(43.93 to 65.37)	0.24(0.07 to 0.43)
Austria	Liver cancer due to hepatitis B	791(553 to 1112)	1205(780 to 1841)	0.52(0.27 to 0.79)	7.91(5.53 to 11.09)	8.28(5.53 to 12.35)	0.05(-0.12 to 0.24)
Austria	Liver cancer due to hepatitis C	2377(1716 to 3120)	4659(3306 to 6390)	0.96(0.73 to 1.21)	20.63(14.86 to 27.24)	27.43(19.17 to 37.62)	0.33(0.17 to 0.51)
Austria	Liver cancer due to NASH	343(238 to 495)	757(506 to 1131)	1.21(0.87 to 1.6)	3.01(2.12 to 4.33)	4.56(3.12 to 6.75)	0.51(0.28 to 0.78)
Austria	Liver cancer due to other causes	495(380 to 646)	774(540 to 1082)	0.56(0.31 to 0.86)	5.75(4.6 to 7.22)	6.16(4.66 to 8.16)	0.07(-0.07 to 0.25)
Azerbaijan	Liver cancer	1606(1448 to 1788)	9875(7589 to 12820)	5.15(3.66 to 7.28)	27.48(24.91 to 30.41)	100.31(77.43 to 131.68)	2.65(1.78 to 4)
Azerbaijan	Liver cancer due to alcohol use	281(191 to 384)	2629(1641 to 3998)	8.35(5.73 to 12.34)	5.52(3.81 to 7.45)	26.39(16.69 to 39.52)	3.78(2.45 to 5.95)
Azerbaijan	Liver cancer due to hepatitis B	366(275 to 485)	2871(1936 to 4165)	6.83(4.84 to 9.71)	6.46(4.75 to 8.57)	26.06(17.64 to 37.28)	3.03(2 to 4.53)
Azerbaijan	Liver cancer due to hepatitis C	392(284 to 509)	3003(1981 to 4347)	6.65(4.66 to 9.42)	8.06(5.94 to 10.3)	32.96(21.89 to 47.85)	3.09(2 to 4.68)
Azerbaijan	Liver cancer due to NASH	69(48 to 96)	691(454 to 1031)	9.04(6.47 to 12.9)	1.36(0.93 to 1.96)	7.48(4.86 to 11.47)	4.51(3.07 to 6.68)

Azerbaijan	Liver cancer due to other causes	498(409 to 617)	682(480 to 1054)	0.37(-0.06 to 1.1)	6.08(5.01 to 7.49)	7.41(5.28 to 11.9)	0.22(-0.15 to 0.89)
Bahamas	Liver cancer	378(331 to 424)	338(274 to 419)	-0.11(-0.3 to 0.15)	219.32(192.3 to 245.65)	81.78(66.85 to 100.95)	-0.63(-0.71 to -0.52)
Bahamas	Liver cancer due to alcohol use	128(94 to 168)	109(74 to 153)	-0.15(-0.34 to 0.13)	78.42(56.5 to 101.85)	26.42(18.18 to 36.72)	-0.66(-0.74 to -0.56)
Bahamas	Liver cancer due to hepatitis B	136(103 to 175)	116(82 to 160)	-0.15(-0.35 to 0.12)	73.05(55.11 to 96.46)	26.71(19.09 to 36.87)	-0.63(-0.72 to -0.52)
Bahamas	Liver cancer due to hepatitis C	57(39 to 79)	58(37 to 83)	0.02(-0.2 to 0.3)	37.09(25.24 to 51.53)	14.77(9.55 to 20.8)	-0.6(-0.69 to -0.49)
Bahamas	Liver cancer due to NASH	26(18 to 35)	29(20 to 44)	0.15(-0.12 to 0.51)	15.19(10.7 to 21.08)	7.34(4.97 to 10.6)	-0.52(-0.63 to -0.37)
Bahamas	Liver cancer due to other causes	31(24 to 40)	25(18 to 35)	-0.19(-0.37 to 0.05)	15.57(11.59 to 20.53)	6.54(4.72 to 8.87)	-0.58(-0.67 to -0.46)
Bahrain	Liver cancer	251(210 to 297)	1079(825 to 1414)	3.29(2.16 to 4.94)	127.57(106.75 to 150.4)	101.97(79.86 to 129.47)	-0.2(-0.4 to 0.08)
Bahrain	Liver cancer due to alcohol use	26(16 to 40)	111(64 to 175)	3.19(1.94 to 5)	14.82(9.13 to 22.42)	10.65(6.4 to 16.6)	-0.28(-0.48 to 0.01)
Bahrain	Liver cancer due to hepatitis B	109(82 to 143)	461(311 to 650)	3.22(2.04 to 5.03)	46.3(33.86 to 63.41)	33.62(23.33 to 48.1)	-0.27(-0.47 to 0)
Bahrain	Liver cancer due to hepatitis C	70(49 to 99)	308(199 to 445)	3.4(2.26 to 4.94)	45.15(31.45 to 60.63)	37.16(25.53 to 50.26)	-0.18(-0.39 to 0.1)
Bahrain	Liver cancer due to NASH	23(16 to 32)	125(81 to 194)	4.48(2.95 to 6.64)	13.04(8.84 to 19.17)	13.8(9.23 to 21.08)	0.06(-0.22 to 0.45)

Bahrain	Liver cancer due to other causes	23(18 to 30)	75(50 to 111)	2.26(1.42 to 3.37)	8.25(5.93 to 11.57)	6.74(4.74 to 9.62)	-0.18(-0.37 to 0.06)
Bangladesh	Liver cancer	66010(50909 to 83163)	99187(78469 to 123484)	0.5(0.11 to 1.13)	93.62(75 to 114.65)	70.52(55.7 to 87.15)	-0.25(-0.44 to 0.04)
Bangladesh	Liver cancer due to alcohol use	9444(6250 to 13692)	18163(11565 to 26252)	0.92(0.36 to 1.71)	18.02(12 to 26.02)	13.38(8.5 to 19.35)	-0.26(-0.48 to 0.04)
Bangladesh	Liver cancer due to hepatitis B	22390(15747 to 30622)	27161(19204 to 37704)	0.21(-0.15 to 0.78)	29.53(21.26 to 40.01)	18.04(12.74 to 25.06)	-0.39(-0.56 to -0.14)
Bangladesh	Liver cancer due to hepatitis C	14420(10002 to 19949)	33128(23055 to 44348)	1.3(0.65 to 2.19)	29.21(20.45 to 39.91)	25.21(17.73 to 33.63)	-0.14(-0.37 to 0.19)
Bangladesh	Liver cancer due to NASH	3946(2675 to 5617)	7342(4998 to 10460)	0.86(0.27 to 1.78)	5.86(4.05 to 8.25)	5.18(3.47 to 7.45)	-0.12(-0.37 to 0.24)
Bangladesh	Liver cancer due to other causes	15811(10608 to 23210)	13393(9797 to 17620)	-0.15(-0.45 to 0.27)	11.01(7.76 to 15.79)	8.72(6.41 to 11.53)	-0.21(-0.47 to 0.18)
Barbados	Liver cancer	196(147 to 252)	295(243 to 355)	0.51(0.08 to 1.21)	72.93(54.13 to 93.22)	65.21(53.3 to 79.03)	-0.11(-0.36 to 0.31)
Barbados	Liver cancer due to alcohol use	61(38 to 93)	109(74 to 146)	0.77(0.19 to 1.77)	22.24(13.82 to 34.05)	22.28(15.42 to 29.69)	0(-0.32 to 0.57)
Barbados	Liver cancer due to hepatitis B	57(37 to 85)	74(52 to 105)	0.3(-0.12 to 1.02)	22.26(14.1 to 32.73)	16.65(11.84 to 23.14)	-0.25(-0.49 to 0.16)
Barbados	Liver cancer due to hepatitis C	39(25 to 58)	60(39 to 85)	0.54(0.13 to 1.14)	13.3(8.28 to 20.21)	12.01(8.02 to 17.08)	-0.1(-0.33 to 0.26)
Barbados	Liver cancer due to NASH	16(11 to 24)	29(20 to 42)	0.77(0.3 to 1.51)	5.67(3.71 to 8.4)	6.06(4.15 to 8.67)	0.07(-0.21 to 0.53)

Barbados	Liver cancer due to other causes	23(16 to 32)	24(16 to 33)	0.04(-0.24 to 0.45)	9.47(6.92 to 13.33)	8.2(5.78 to 11.24)	-0.13(-0.34 to 0.18)
Belarus	Liver cancer	4290(4024 to 4544)	9071(6450 to 12404)	1.11(0.51 to 1.87)	34.77(32.58 to 36.88)	62.5(44.93 to 85.19)	0.8(0.28 to 1.44)
Belarus	Liver cancer due to alcohol use	1468(1099 to 1831)	3897(2434 to 5819)	1.66(0.81 to 2.81)	11.16(8.42 to 13.92)	25.06(15.47 to 37.47)	1.24(0.52 to 2.22)
Belarus	Liver cancer due to hepatitis B	1035(768 to 1399)	2038(1236 to 3213)	0.97(0.33 to 1.81)	8.09(6.11 to 10.77)	14.35(8.7 to 22.2)	0.77(0.2 to 1.54)
Belarus	Liver cancer due to hepatitis C	961(672 to 1274)	1974(1236 to 2979)	1.05(0.52 to 1.69)	7.31(5.18 to 9.6)	12.35(7.8 to 18.42)	0.69(0.25 to 1.22)
Belarus	Liver cancer due to NASH	248(173 to 356)	604(376 to 931)	1.43(0.79 to 2.29)	1.92(1.36 to 2.7)	3.98(2.51 to 6.01)	1.07(0.51 to 1.82)
Belarus	Liver cancer due to other causes	577(449 to 693)	558(404 to 758)	-0.03(-0.31 to 0.36)	6.28(4.88 to 7.52)	6.75(4.99 to 8.88)	0.08(-0.25 to 0.56)
Belgium	Liver cancer	9361(8908 to 9814)	16999(15459 to 18522)	0.82(0.64 to 1.02)	64.22(61.3 to 67.4)	84.28(76.44 to 92.36)	0.31(0.18 to 0.45)
Belgium	Liver cancer due to alcohol use	3521(2661 to 4358)	6797(5080 to 8610)	0.93(0.68 to 1.18)	23.63(17.96 to 29.21)	33.53(25.17 to 41.88)	0.42(0.23 to 0.61)
Belgium	Liver cancer due to hepatitis B	1398(996 to 1916)	2316(1577 to 3330)	0.66(0.45 to 0.88)	10.29(7.48 to 13.82)	12.89(9.17 to 18.11)	0.25(0.1 to 0.42)
Belgium	Liver cancer due to hepatitis C	3373(2514 to 4263)	6027(4514 to 7722)	0.79(0.6 to 1)	21.88(16.49 to 27.82)	27.67(20.67 to 35.93)	0.26(0.13 to 0.42)
Belgium	Liver cancer due to NASH	512(352 to 735)	1026(708 to 1492)	1.01(0.73 to 1.35)	3.38(2.35 to 4.81)	4.87(3.39 to 7.01)	0.44(0.24 to 0.69)

Belgium	Liver cancer due to other causes	557(430 to 734)	833(593 to 1152)	0.49(0.28 to 0.72)	5.03(4.1 to 6.2)	5.33(4.12 to 6.93)	0.06(-0.08 to 0.2)
Belize	Liver cancer	157(142 to 172)	234(199 to 271)	0.49(0.23 to 0.77)	152.98(138.86 to 167.31)	77.57(66.16 to 89.66)	-0.49(-0.58 to -0.4)
Belize	Liver cancer due to alcohol use	42(31 to 56)	74(53 to 98)	0.74(0.41 to 1.11)	45.19(32.48 to 59.31)	25.74(18.54 to 33.94)	-0.43(-0.53 to -0.31)
Belize	Liver cancer due to hepatitis B	50(39 to 64)	78(58 to 104)	0.55(0.25 to 0.91)	47.97(35.98 to 62)	23.73(17.55 to 31.94)	-0.51(-0.6 to -0.4)
Belize	Liver cancer due to hepatitis C	30(21 to 41)	40(26 to 57)	0.32(0.08 to 0.59)	33.01(23.14 to 44.59)	14.65(9.73 to 20.85)	-0.56(-0.64 to -0.47)
Belize	Liver cancer due to NASH	12(8 to 16)	21(14 to 28)	0.77(0.4 to 1.2)	11.97(8.4 to 16.48)	7.11(4.93 to 9.86)	-0.41(-0.52 to -0.27)
Belize	Liver cancer due to other causes	23(18 to 27)	22(17 to 29)	-0.02(-0.22 to 0.21)	14.84(11.61 to 18.61)	6.33(4.74 to 8.39)	-0.57(-0.65 to -0.48)
Benin	Liver cancer	4413(3545 to 5572)	8053(5733 to 11095)	0.82(0.27 to 1.58)	179.58(144.02 to 231.64)	128.18(93.89 to 174.06)	-0.29(-0.49 to 0.02)
Benin	Liver cancer due to alcohol use	616(396 to 947)	1216(735 to 1940)	0.98(0.36 to 1.88)	29.72(18.84 to 45.83)	23.19(14.21 to 36.49)	-0.22(-0.46 to 0.14)
Benin	Liver cancer due to hepatitis B	2242(1660 to 2959)	4019(2713 to 5746)	0.79(0.21 to 1.68)	95.6(69.73 to 127.45)	62.31(42.57 to 87.27)	-0.35(-0.55 to -0.04)
Benin	Liver cancer due to hepatitis C	573(363 to 824)	1012(622 to 1500)	0.77(0.26 to 1.54)	29.03(18.27 to 41.63)	21.18(13.03 to 30.24)	-0.27(-0.47 to 0.03)
Benin	Liver cancer due to NASH	258(173 to 374)	635(415 to 962)	1.46(0.7 to 2.56)	12.01(7.88 to 17.71)	11.63(7.7 to 18.13)	-0.03(-0.32 to 0.39)

Benin	Liver cancer due to other causes	724(486 to 1058)	1171(759 to 1756)	0.62(0 to 1.53)	13.23(9.55 to 17.85)	9.87(6.6 to 14.53)	-0.25(-0.49 to 0.06)
Bermuda	Liver cancer	110(99 to 120)	55(45 to 67)	-0.5(-0.6 to -0.38)	172.93(156.14 to 189.53)	45.46(37.15 to 56.14)	-0.74(-0.79 to -0.67)
Bermuda	Liver cancer due to alcohol use	42(32 to 53)	21(15 to 29)	-0.49(-0.6 to -0.35)	65.85(49.22 to 82.87)	16.98(11.91 to 23.01)	-0.74(-0.8 to -0.67)
Bermuda	Liver cancer due to hepatitis B	32(23 to 43)	14(10 to 21)	-0.55(-0.65 to -0.42)	49.14(35.81 to 65.81)	12.63(8.71 to 17.6)	-0.74(-0.8 to -0.67)
Bermuda	Liver cancer due to hepatitis C	20(13 to 27)	10(7 to 15)	-0.47(-0.58 to -0.33)	31.87(21.57 to 43.93)	7.92(4.96 to 11.68)	-0.75(-0.8 to -0.68)
Bermuda	Liver cancer due to NASH	9(6 to 13)	5(4 to 8)	-0.41(-0.54 to -0.23)	14.44(10.2 to 20.17)	4.27(2.93 to 6.16)	-0.7(-0.77 to -0.62)
Bermuda	Liver cancer due to other causes	7(5 to 9)	3(2 to 4)	-0.58(-0.69 to -0.46)	11.63(8.94 to 15.19)	3.66(2.69 to 4.91)	-0.69(-0.76 to -0.59)
Bhutan	Liver cancer	201(124 to 301)	465(313 to 664)	1.31(0.53 to 2.71)	61.71(40.07 to 91.75)	77.74(52.99 to 110.69)	0.26(-0.15 to 0.92)
Bhutan	Liver cancer due to alcohol use	48(27 to 81)	125(71 to 197)	1.63(0.7 to 3.09)	17.07(9.81 to 28.7)	21.4(12.23 to 33.76)	0.25(-0.18 to 0.91)
Bhutan	Liver cancer due to hepatitis B	66(37 to 108)	133(78 to 211)	1.01(0.26 to 2.3)	19.55(11.1 to 32.07)	20.83(12.08 to 32.72)	0.07(-0.32 to 0.7)
Bhutan	Liver cancer due to hepatitis C	41(23 to 65)	123(76 to 190)	2.03(1.03 to 3.62)	15.94(9.22 to 24.73)	22.15(13.64 to 34.04)	0.39(-0.03 to 1.05)
Bhutan	Liver cancer due to NASH	12(7 to 21)	40(24 to 64)	2.23(1.12 to 4.21)	4.25(2.49 to 6.91)	6.86(4.22 to 10.84)	0.61(0.08 to 1.46)

Bhutan	Liver cancer due to other causes	34(12 to 61)	43(28 to 64)	0.25(-0.32 to 2.47)	4.89(2.15 to 8.02)	6.49(4.32 to 9.57)	0.33(-0.22 to 2.01)
Bolivia (Plurinational State of)	Liver cancer	4924(3708 to 6508)	10580(7826 to 13986)	1.15(0.51 to 2.02)	130.51(98.22 to 171.56)	113.25(83.6 to 148.79)	-0.13(-0.38 to 0.22)
Bolivia (Plurinational State of)	Liver cancer due to alcohol use	1086(666 to 1607)	2923(1897 to 4286)	1.69(0.9 to 2.84)	32.95(20.39 to 49.39)	32.67(21.45 to 47.81)	-0.01(-0.29 to 0.39)
Bolivia (Plurinational State of)	Liver cancer due to hepatitis B	2419(1740 to 3271)	4852(3357 to 6791)	1.01(0.38 to 1.94)	65.11(46.53 to 88.07)	51.17(35.34 to 70.8)	-0.21(-0.45 to 0.14)
Bolivia (Plurinational State of)	Liver cancer due to hepatitis C	280(149 to 484)	692(373 to 1161)	1.47(0.75 to 2.53)	9.14(4.97 to 15.46)	8.2(4.55 to 13.7)	-0.1(-0.36 to 0.27)
Bolivia (Plurinational State of)	Liver cancer due to NASH	308(188 to 523)	875(539 to 1384)	1.84(0.96 to 3.17)	9.35(5.63 to 15.85)	10.03(6.22 to 15.83)	0.07(-0.25 to 0.56)
Bolivia (Plurinational State of)	Liver cancer due to other causes	831(565 to 1231)	1237(855 to 1691)	0.49(-0.04 to 1.24)	13.96(9.18 to 21)	11.19(7.54 to 15.73)	-0.2(-0.46 to 0.15)
Bosnia and Herzegovina	Liver cancer	6255(5793 to 6722)	10344(8111 to 13063)	0.65(0.29 to 1.09)	141.53(131.88 to 151.39)	176.45(138.37 to 222.84)	0.25(-0.02 to 0.58)
Bosnia and Herzegovina	Liver cancer due to alcohol use	2010(1412 to 2634)	3679(2504 to 5198)	0.83(0.4 to 1.4)	45.57(33.03 to 58.95)	60.91(41.25 to 85.64)	0.34(0.02 to 0.75)
Bosnia and Herzegovina	Liver cancer due to hepatitis B	2086(1532 to 2782)	2480(1624 to 3746)	0.19(-0.11 to 0.54)	44.53(32.93 to 58.98)	44.42(29.68 to 65.3)	0(-0.24 to 0.27)
Bosnia and Herzegovina	Liver cancer due to hepatitis C	1480(998 to 2030)	2873(1882 to 4129)	0.94(0.47 to 1.54)	35.46(24.41 to 47.63)	47.44(31.33 to 67.96)	0.34(0.03 to 0.71)

Bosnia and Herzegovina	Liver cancer due to NASH	380(259 to 544)	916(603 to 1374)	1.41(0.79 to 2.19)	9.04(6.2 to 12.97)	15.54(10.34 to 23.01)	0.72(0.28 to 1.25)
Bosnia and Herzegovina	Liver cancer due to other causes	299(221 to 396)	395(251 to 600)	0.32(-0.03 to 0.75)	6.92(5.24 to 9.16)	8.15(5.52 to 11.75)	0.18(-0.11 to 0.53)
Botswana	Liver cancer	180(87 to 418)	711(477 to 1006)	2.95(0.68 to 7.4)	26.49(12.75 to 60.85)	40.98(28.78 to 55.8)	0.55(-0.33 to 2.27)
Botswana	Liver cancer due to alcohol use	30(11 to 88)	138(80 to 222)	3.65(0.79 to 10.19)	4.69(1.71 to 13.63)	8.47(5.04 to 13.38)	0.81(-0.29 to 3.28)
Botswana	Liver cancer due to hepatitis B	79(33 to 213)	323(205 to 490)	3.09(0.55 to 8.54)	10.8(4.51 to 28.79)	16.55(10.7 to 24.85)	0.53(-0.4 to 2.53)
Botswana	Liver cancer due to hepatitis C	41(20 to 83)	137(83 to 204)	2.34(0.68 to 5.48)	7.18(3.47 to 14.25)	9.64(5.97 to 14.02)	0.34(-0.31 to 1.59)
Botswana	Liver cancer due to NASH	13(6 to 29)	61(36 to 96)	3.53(1.18 to 8.41)	2.1(0.92 to 4.57)	3.79(2.32 to 5.73)	0.81(-0.12 to 2.68)
Botswana	Liver cancer due to other causes	16(10 to 29)	51(33 to 73)	2.1(0.75 to 4.21)	1.73(0.93 to 3.32)	2.53(1.65 to 3.66)	0.47(-0.24 to 1.63)
Brazil	Liver cancer	52792(50965 to 54479)	139930(132618 to 147341)	1.65(1.49 to 1.82)	51.99(49.92 to 53.63)	58.86(55.66 to 61.99)	0.13(0.07 to 0.2)
Brazil	Liver cancer due to alcohol use	13214(11475 to 15181)	42725(36702 to 48757)	2.23(2.01 to 2.48)	13.82(11.97 to 15.84)	17.65(15.21 to 20.12)	0.28(0.2 to 0.37)
Brazil	Liver cancer due to hepatitis B	13529(11768 to 15333)	29069(24784 to 33829)	1.15(1 to 1.33)	12.37(10.68 to 14.11)	11.9(10.16 to 13.81)	-0.04(-0.1 to 0.03)
Brazil	Liver cancer due to hepatitis C	15888(14002 to 17854)	49167(42836 to 55644)	2.09(1.91 to 2.3)	17.75(15.76 to 19.82)	20.7(18.06 to 23.36)	0.17(0.1 to 0.24)

Brazil	Liver cancer due to NASH	2703(2336 to 3106)	8536(7295 to 9997)	2.16(1.94 to 2.38)	2.75(2.37 to 3.18)	3.59(3.07 to 4.21)	0.3(0.22 to 0.38)
Brazil	Liver cancer due to other causes	7459(6737 to 8333)	10434(9187 to 11847)	0.4(0.22 to 0.6)	5.3(4.77 to 5.88)	5.02(4.38 to 5.74)	-0.05(-0.17 to 0.08)
Brunei Darussalam	Liver cancer	285(232 to 345)	1079(903 to 1295)	2.79(1.94 to 3.95)	238.18(193.59 to 286.5)	284.15(241.01 to 336.53)	0.19(-0.06 to 0.53)
Brunei Darussalam	Liver cancer due to alcohol use	24(15 to 36)	92(56 to 147)	2.85(1.84 to 4.29)	22.17(13.45 to 33.67)	25.45(15.68 to 38.84)	0.15(-0.14 to 0.52)
Brunei Darussalam	Liver cancer due to hepatitis B	167(128 to 216)	618(468 to 800)	2.69(1.74 to 3.95)	120.93(89.98 to 158.24)	144.05(108.35 to 184.61)	0.19(-0.09 to 0.58)
Brunei Darussalam	Liver cancer due to hepatitis C	67(43 to 97)	271(181 to 382)	3.04(2.17 to 4.21)	73.53(49.01 to 103.93)	86.49(60.49 to 117.48)	0.18(-0.06 to 0.5)
Brunei Darussalam	Liver cancer due to NASH	11(7 to 17)	49(31 to 73)	3.33(2.26 to 4.71)	11.26(7.34 to 16.96)	14.84(9.83 to 21.79)	0.32(0.01 to 0.73)
Brunei Darussalam	Liver cancer due to other causes	15(11 to 21)	50(34 to 70)	2.25(1.42 to 3.21)	10.29(6.98 to 14.64)	13.31(9.13 to 18.54)	0.29(0.01 to 0.68)
Bulgaria	Liver cancer	20457(19078 to 21897)	14782(11719 to 18455)	-0.28(-0.44 to -0.09)	163.88(152.99 to 175.76)	117.83(92.99 to 148.19)	-0.28(-0.44 to -0.09)
Bulgaria	Liver cancer due to alcohol use	8326(6310 to 10386)	6795(4892 to 9243)	-0.18(-0.38 to 0.06)	64.64(49.18 to 80.66)	52.18(36.96 to 70.95)	-0.19(-0.39 to 0.04)
Bulgaria	Liver cancer due to hepatitis B	5716(4201 to 7687)	3398(2262 to 5081)	-0.41(-0.54 to -0.24)	47.71(35.62 to 62.8)	30.27(20.4 to 44.37)	-0.37(-0.51 to -0.19)
Bulgaria	Liver cancer due to hepatitis C	4301(2893 to 5972)	3063(1976 to 4523)	-0.29(-0.45 to -0.09)	33.45(22.7 to 45.54)	22.13(14.38 to 32.6)	-0.34(-0.48 to -0.15)

Bulgaria	Liver cancer due to NASH	1330(910 to 1870)	1032(676 to 1522)	-0.22(-0.4 to 0)	10.66(7.51 to 14.72)	7.87(5.19 to 11.4)	-0.26(-0.44 to -0.05)
Bulgaria	Liver cancer due to other causes	785(573 to 1062)	494(323 to 710)	-0.37(-0.52 to -0.17)	7.43(5.7 to 9.66)	5.37(3.78 to 7.36)	-0.28(-0.44 to -0.07)
Burkina Faso	Liver cancer	5769(4383 to 7532)	10107(7409 to 13700)	0.75(0.22 to 1.46)	85.03(67.62 to 104.25)	64.53(48.58 to 82.4)	-0.24(-0.45 to 0.01)
Burkina Faso	Liver cancer due to alcohol use	495(307 to 729)	884(557 to 1287)	0.79(0.21 to 1.57)	10.86(6.76 to 15.93)	9.05(5.7 to 13.19)	-0.17(-0.44 to 0.18)
Burkina Faso	Liver cancer due to hepatitis B	1898(1383 to 2498)	3171(2183 to 4422)	0.67(0.16 to 1.36)	35.53(25.66 to 46.56)	24.76(17.33 to 33.99)	-0.3(-0.52 to -0.03)
Burkina Faso	Liver cancer due to hepatitis C	834(548 to 1169)	1217(790 to 1704)	0.46(0.04 to 1.05)	19.45(13.06 to 26.75)	13.4(8.89 to 18.44)	-0.31(-0.5 to -0.06)
Burkina Faso	Liver cancer due to NASH	230(155 to 332)	509(325 to 725)	1.21(0.56 to 2.11)	4.89(3.28 to 7.08)	4.74(3.03 to 6.9)	-0.03(-0.29 to 0.35)
Burkina Faso	Liver cancer due to other causes	2312(1324 to 3795)	4326(2481 to 7045)	0.87(-0.03 to 2.42)	14.29(9.11 to 22.09)	12.57(8.08 to 19.24)	-0.12(-0.49 to 0.46)
Burundi	Liver cancer	3222(2287 to 4379)	4829(3313 to 7429)	0.5(-0.09 to 1.28)	104.42(74.76 to 138.99)	78.61(53.72 to 123.25)	-0.25(-0.53 to 0.15)
Burundi	Liver cancer due to alcohol use	781(488 to 1161)	1065(593 to 1912)	0.36(-0.18 to 1.17)	31.58(19.9 to 46.44)	21.3(11.77 to 38.22)	-0.33(-0.59 to 0.04)
Burundi	Liver cancer due to hepatitis B	914(602 to 1303)	1518(932 to 2573)	0.66(-0.02 to 1.65)	31.08(20.31 to 44.72)	23.63(13.93 to 39.63)	-0.24(-0.55 to 0.21)
Burundi	Liver cancer due to hepatitis C	450(260 to 706)	732(428 to 1157)	0.63(0.03 to 1.52)	19.26(11.25 to 29.85)	16.2(9.65 to 25.12)	-0.16(-0.46 to 0.27)
Burundi	Liver cancer due to NASH	256(158 to 395)	437(269 to 717)	0.7(0.03 to 1.62)	9.84(6.1 to 15.14)	8.31(5.07 to	-0.16(-0.47 to 0.29)

						13.64)	
Burundi	Liver cancer due to other causes	821(486 to 1383)	1077(690 to 1638)	0.31(-0.31 to 1.48)	12.66(8.06 to 19.61)	9.17(6.22 to 13.62)	-0.28(-0.59 to 0.18)
C??te d'Ivoire	Liver cancer	11312(8325 to 14808)	17290(12133 to 23836)	0.53(0.07 to 1.1)	204.36(153.69 to 261.23)	125.38(90.88 to 172.46)	-0.39(-0.56 to -0.17)
C??te d'Ivoire	Liver cancer due to alcohol use	1670(981 to 2508)	2909(1738 to 4590)	0.74(0.22 to 1.49)	37.03(22.32 to 56.41)	24.83(15.02 to 38.99)	-0.33(-0.53 to -0.06)
C??te d'Ivoire	Liver cancer due to hepatitis B	6393(4450 to 8628)	9378(6228 to 13385)	0.47(-0.01 to 1.11)	113.61(79.87 to 152.27)	63.37(42.47 to 90.53)	-0.44(-0.62 to -0.22)
C??te d'Ivoire	Liver cancer due to hepatitis C	1109(666 to 1696)	1875(1109 to 2873)	0.69(0.21 to 1.31)	28.04(17.6 to 41.09)	18.35(11.15 to 27.58)	-0.35(-0.52 to -0.14)
C??te d'Ivoire	Liver cancer due to NASH	599(394 to 859)	1251(788 to 1874)	1.09(0.42 to 1.94)	12.98(8.49 to 19.08)	10.49(6.72 to 16.03)	-0.19(-0.43 to 0.1)
C??te d'Ivoire	Liver cancer due to other causes	1542(1017 to 2247)	1876(1210 to 2676)	0.22(-0.22 to 0.84)	12.7(8.85 to 17.75)	8.34(5.43 to 12.19)	-0.34(-0.53 to -0.1)
Cabo Verde	Liver cancer	68(59 to 78)	1411(1128 to 1745)	19.8(15.34 to 25.54)	28.25(24.39 to 32.6)	303.59(245.48 to 372.44)	9.75(7.43 to 12.67)
Cabo Verde	Liver cancer due to alcohol use	10(7 to 14)	246(164 to 351)	24.12(17.73 to 32.86)	4.35(2.89 to 6.22)	55.92(37.19 to 78.28)	11.85(8.97 to 15.97)
Cabo Verde	Liver cancer due to hepatitis B	32(26 to 39)	765(573 to 1009)	22.9(17.19 to 30.49)	14.17(11.15 to 17.71)	155.31(117.04 to 204.2)	9.96(7.41 to 13.37)
Cabo Verde	Liver cancer due to hepatitis C	12(8 to 17)	191(126 to 270)	15.07(11.28 to 20.62)	5.13(3.5 to 7.11)	46.32(30.13 to 65.76)	8.03(5.88 to 11.12)
Cabo Verde	Liver cancer due to NASH	5(4 to 7)	126(87 to 178)	23.41(17.33 to 31.48)	2.2(1.53 to 3.14)	29.03(19.73 to	12.17(8.84 to 16.64)

						41.44)	
Cabo Verde	Liver cancer due to other causes	9(7 to 12)	83(59 to 112)	8.24(5.36 to 12.35)	2.39(1.84 to 3.13)	17.01(11.91 to 23.32)	6.11(4.28 to 8.53)
Cambodia	Liver cancer	31806(22069 to 49657)	40159(30496 to 51295)	0.26(-0.3 to 1.03)	371.64(291.05 to 482.64)	284.56(217.91 to 357.03)	-0.23(-0.5 to 0.1)
Cambodia	Liver cancer due to alcohol use	1556(990 to 2321)	5028(3137 to 7475)	2.23(1.27 to 3.55)	31.29(20.03 to 46.78)	39.13(24.69 to 57.2)	0.25(-0.11 to 0.75)
Cambodia	Liver cancer due to hepatitis B	9474(7115 to 12211)	14228(10130 to 19567)	0.5(-0.01 to 1.15)	135.47(101.51 to 177.27)	96.16(68.82 to 133.31)	-0.29(-0.51 to 0)
Cambodia	Liver cancer due to hepatitis C	4972(3353 to 6699)	10002(6703 to 13664)	1.01(0.4 to 1.92)	105.43(73.51 to 141.11)	82.43(56.92 to 109.92)	-0.22(-0.44 to 0.14)
Cambodia	Liver cancer due to NASH	1015(680 to 1494)	2182(1428 to 3206)	1.15(0.42 to 2.15)	17.21(11.66 to 24.97)	16.3(10.79 to 23.96)	-0.05(-0.35 to 0.37)
Cambodia	Liver cancer due to other causes	14788(6723 to 30575)	8718(4530 to 15787)	-0.41(-0.8 to 0.51)	82.24(41.3 to 161.02)	50.55(26.79 to 90.62)	-0.39(-0.78 to 0.5)
Cameroon	Liver cancer	933(654 to 1399)	2744(1910 to 3799)	1.94(0.81 to 3.44)	17.38(12.08 to 26.38)	17.73(12.52 to 24.34)	0.02(-0.36 to 0.52)
Cameroon	Liver cancer due to alcohol use	146(80 to 253)	462(276 to 730)	2.17(0.84 to 3.94)	3.07(1.73 to 5.37)	3.5(2.13 to 5.45)	0.14(-0.32 to 0.75)
Cameroon	Liver cancer due to hepatitis B	502(327 to 797)	1443(927 to 2083)	1.88(0.68 to 3.44)	9.16(5.84 to 14.53)	8.89(5.87 to 12.7)	-0.03(-0.42 to 0.5)
Cameroon	Liver cancer due to hepatitis C	116(69 to 184)	288(172 to 444)	1.48(0.63 to 2.72)	2.66(1.57 to 4.18)	2.45(1.49 to 3.67)	-0.08(-0.38 to 0.32)
Cameroon	Liver cancer due to NASH	67(41 to 107)	218(136 to 335)	2.23(1.06 to 3.87)	1.4(0.85 to 2.23)	1.6(1 to 2.52)	0.14(-0.26 to 0.69)

Cameroon	Liver cancer due to other causes	101(71 to 142)	333(221 to 468)	2.29(1.16 to 3.83)	1.1(0.75 to 1.56)	1.28(0.85 to 1.8)	0.17(-0.23 to 0.69)
Canada	Liver cancer	14874(14254 to 15463)	56804(50595 to 63104)	2.82(2.4 to 3.25)	47.3(45.37 to 49.21)	91.02(81.09 to 100.87)	0.92(0.71 to 1.14)
Canada	Liver cancer due to alcohol use	8449(7276 to 9597)	32450(26074 to 38593)	2.84(2.3 to 3.35)	26.41(22.74 to 29.96)	50.95(41.09 to 60.33)	0.93(0.67 to 1.19)
Canada	Liver cancer due to hepatitis B	1398(985 to 1992)	4263(2852 to 6299)	2.05(1.55 to 2.58)	4.48(3.14 to 6.43)	7.5(5.2 to 10.87)	0.68(0.44 to 0.92)
Canada	Liver cancer due to hepatitis C	2371(1602 to 3285)	9885(6656 to 13756)	3.17(2.71 to 3.73)	7.42(5.02 to 10.24)	14.81(10.09 to 20.52)	1(0.78 to 1.26)
Canada	Liver cancer due to NASH	1369(997 to 1893)	6629(4675 to 9567)	3.84(3.14 to 4.64)	4.26(3.13 to 5.86)	10.23(7.35 to 14.37)	1.4(1.07 to 1.78)
Canada	Liver cancer due to other causes	1287(1025 to 1608)	3577(2652 to 4859)	1.78(1.31 to 2.23)	4.74(3.87 to 5.8)	7.52(5.9 to 9.41)	0.59(0.39 to 0.81)
Central African Republic	Liver cancer	1785(1196 to 2563)	2681(1676 to 4149)	0.5(0.08 to 1.08)	104.65(72.63 to 146.78)	89.47(57.27 to 133.87)	-0.14(-0.36 to 0.15)
Central African Republic	Liver cancer due to alcohol use	203(113 to 334)	328(174 to 587)	0.61(0.14 to 1.3)	15.53(8.76 to 24.86)	13.48(7.43 to 23.26)	-0.13(-0.37 to 0.22)
Central African Republic	Liver cancer due to hepatitis B	533(332 to 786)	817(450 to 1374)	0.53(0.06 to 1.21)	31.78(19.59 to 47.45)	25.26(14.21 to 41.39)	-0.21(-0.44 to 0.11)
Central African Republic	Liver cancer due to hepatitis C	475(290 to 728)	790(457 to 1258)	0.66(0.19 to 1.3)	38.91(24.42 to 57.9)	35.53(21.46 to 53.92)	-0.09(-0.34 to 0.25)
Central African Republic	Liver cancer due to NASH	81(49 to 130)	148(82 to 254)	0.83(0.3 to 1.62)	5.82(3.53 to 9.2)	5.69(3.26 to 9.55)	-0.02(-0.3 to 0.35)
Central African	Liver cancer due to other	493(271 to 819)	598(344 to 972)	0.21(-0.3 to 1.04)	12.61(7.42 to 19.68)	9.51(5.68 to	-0.25(-0.52 to 0.15)

Republic	causes					15.27)	
Chad	Liver cancer	5652(4392 to 7247)	10260(7570 to 13390)	0.82(0.36 to 1.43)	169.24(130.8 to 217.85)	141.62(105.92 to 185.48)	-0.16(-0.37 to 0.12)
Chad	Liver cancer due to alcohol use	688(404 to 1071)	1257(756 to 1956)	0.83(0.31 to 1.55)	23.39(13.58 to 36.36)	21.2(12.95 to 32.57)	-0.09(-0.35 to 0.26)
Chad	Liver cancer due to hepatitis B	2962(2093 to 4030)	5461(3803 to 7486)	0.84(0.34 to 1.54)	92.62(64.76 to 125.77)	75.42(52.97 to 102.96)	-0.19(-0.4 to 0.11)
Chad	Liver cancer due to hepatitis C	835(521 to 1208)	1328(818 to 1976)	0.59(0.19 to 1.16)	29.68(18.66 to 42.77)	24.13(14.75 to 35.63)	-0.19(-0.38 to 0.08)
Chad	Liver cancer due to NASH	335(220 to 495)	664(423 to 977)	0.98(0.45 to 1.72)	11.18(7.26 to 16.7)	10.65(6.73 to 16.08)	-0.05(-0.29 to 0.29)
Chad	Liver cancer due to other causes	831(526 to 1243)	1551(1057 to 2210)	0.86(0.24 to 1.85)	12.37(8.49 to 16.87)	10.21(6.86 to 14.27)	-0.17(-0.38 to 0.14)
Chile	Liver cancer	5644(5230 to 6089)	15838(14565 to 17228)	1.81(1.48 to 2.18)	53.59(49.78 to 57.74)	66.96(61.63 to 72.7)	0.25(0.1 to 0.41)
Chile	Liver cancer due to alcohol use	1691(1248 to 2180)	5044(3625 to 6572)	1.98(1.56 to 2.49)	16.36(11.99 to 21.24)	20.9(15.1 to 27.13)	0.28(0.1 to 0.5)
Chile	Liver cancer due to hepatitis B	1294(949 to 1785)	2927(2039 to 4140)	1.26(0.9 to 1.67)	11.86(8.58 to 16.58)	12.46(8.74 to 17.38)	0.05(-0.1 to 0.22)
Chile	Liver cancer due to hepatitis C	1638(1171 to 2114)	5196(3716 to 6836)	2.17(1.78 to 2.64)	16.32(11.66 to 20.91)	21.43(15.35 to 28.13)	0.31(0.16 to 0.5)
Chile	Liver cancer due to NASH	399(281 to 562)	1538(1068 to 2196)	2.85(2.24 to 3.55)	3.89(2.72 to 5.48)	6.41(4.49 to 9.11)	0.65(0.41 to 0.93)
Chile	Liver cancer due to other	622(508 to 767)	1134(839 to 1525)	0.82(0.47 to 1.22)	5.16(4.13 to 6.51)	5.76(4.39 to	0.12(-0.09 to 0.35)

	causes					7.57)	
China	Liver cancer	7577768(6419833 to 8981021)	5325461(4425687 to 6374588)	-0.3(-0.46 to -0.08)	769.11(653.21 to 913.07)	264.31(220.69 to 315.14)	-0.66(-0.73 to -0.55)
China	Liver cancer due to alcohol use	508949(374286 to 687646)	451771(329264 to 590301)	-0.11(-0.32 to 0.16)	54.03(40.02 to 72.29)	21.36(15.73 to 27.62)	-0.6(-0.69 to -0.49)
China	Liver cancer due to hepatitis B	5264531(4341621 to 6354239)	3597725(2908492 to 4437040)	-0.32(-0.48 to -0.08)	520.57(430.64 to 628.29)	177.67(144.15 to 218.32)	-0.66(-0.74 to -0.54)
China	Liver cancer due to hepatitis C	909455(744826 to 1114928)	714427(580678 to 856767)	-0.21(-0.38 to -0.01)	105.93(87.73 to 128.71)	35.11(28.85 to 41.74)	-0.67(-0.74 to -0.58)
China	Liver cancer due to NASH	256934(205032 to 320337)	229176(183154 to 283928)	-0.11(-0.29 to 0.13)	27.27(21.81 to 33.95)	11.33(9.13 to 14.01)	-0.58(-0.67 to -0.48)
China	Liver cancer due to other causes	637899(527607 to 769921)	332362(267901 to 403087)	-0.48(-0.59 to -0.34)	61.31(50.56 to 74.38)	18.83(15.64 to 22.29)	-0.69(-0.75 to -0.62)
Colombia	Liver cancer	14848(14130 to 15586)	30991(23416 to 40346)	1.09(0.58 to 1.72)	73.45(69.76 to 77.12)	59.85(45.22 to 77.53)	-0.19(-0.38 to 0.06)
Colombia	Liver cancer due to alcohol use	3850(2790 to 4945)	9627(6269 to 13750)	1.5(0.81 to 2.34)	20.64(14.84 to 26.45)	18.37(11.97 to 26.22)	-0.11(-0.35 to 0.18)
Colombia	Liver cancer due to hepatitis B	3714(2875 to 4733)	6204(3959 to 9326)	0.67(0.22 to 1.22)	16.88(12.69 to 22.31)	11.82(7.52 to 17.75)	-0.3(-0.48 to -0.08)
Colombia	Liver cancer due to hepatitis C	4311(3218 to 5395)	10190(6882 to 14502)	1.36(0.79 to 2.09)	24.72(18.67 to 30.87)	19.33(13.05 to 27.46)	-0.22(-0.41 to 0.02)
Colombia	Liver cancer due to NASH	811(581 to 1120)	2258(1442 to 3419)	1.79(1.06 to 2.76)	4.2(2.95 to 5.96)	4.31(2.74 to 6.51)	0.02(-0.23 to 0.38)

Colombia	Liver cancer due to other causes	2163(1781 to 2618)	2713(1930 to 3648)	0.25(-0.09 to 0.72)	7.01(5.66 to 8.64)	6.02(4.35 to 8.06)	-0.14(-0.36 to 0.15)
Comoros	Liver cancer	231(110 to 384)	403(262 to 632)	0.74(0.13 to 2.81)	84.8(42.23 to 144.32)	74.96(50.13 to 117.54)	-0.12(-0.41 to 0.81)
Comoros	Liver cancer due to alcohol use	37(16 to 78)	71(38 to 143)	0.91(0.25 to 2.88)	16.26(7.02 to 34.04)	14.16(7.55 to 27.81)	-0.13(-0.42 to 0.67)
Comoros	Liver cancer due to hepatitis B	77(29 to 146)	140(77 to 244)	0.81(0.12 to 3.55)	29.81(12.03 to 57.01)	24.68(13.86 to 42.8)	-0.17(-0.48 to 0.93)
Comoros	Liver cancer due to hepatitis C	40(20 to 66)	82(50 to 126)	1.04(0.38 to 2.71)	18.16(9.65 to 29.72)	16.93(10.3 to 26.17)	-0.07(-0.36 to 0.61)
Comoros	Liver cancer due to NASH	23(11 to 41)	52(32 to 82)	1.22(0.45 to 3.54)	9.85(4.84 to 17.11)	10.14(6.21 to 15.98)	0.03(-0.3 to 0.92)
Comoros	Liver cancer due to other causes	53(25 to 85)	58(35 to 88)	0.1(-0.39 to 1.41)	10.73(5.16 to 16.9)	9.05(5.58 to 13.72)	-0.16(-0.48 to 0.78)
Congo	Liver cancer	1632(1172 to 2211)	2485(1750 to 3420)	0.52(0.08 to 1.19)	112.88(82.91 to 155.27)	76.71(54.88 to 105.05)	-0.32(-0.51 to -0.04)
Congo	Liver cancer due to alcohol use	190(109 to 313)	361(206 to 572)	0.9(0.3 to 1.91)	16.2(9.4 to 26.23)	12.37(7.35 to 19.55)	-0.24(-0.47 to 0.16)
Congo	Liver cancer due to hepatitis B	468(300 to 704)	732(473 to 1114)	0.57(0.02 to 1.4)	31.8(20.2 to 48.85)	19.74(12.68 to 30.34)	-0.38(-0.58 to -0.07)
Congo	Liver cancer due to hepatitis C	506(330 to 748)	854(546 to 1289)	0.69(0.18 to 1.36)	46.07(31.1 to 66.24)	32.01(21.22 to 46.72)	-0.31(-0.49 to -0.05)
Congo	Liver cancer due to NASH	85(53 to 131)	177(105 to 281)	1.08(0.39 to 2.05)	6.83(4.27 to 10.53)	5.81(3.5 to 9.01)	-0.15(-0.41 to 0.2)
Congo	Liver cancer due to other	383(224 to 609)	361(232 to 547)	-0.06(-0.45 to 0.58)	11.99(7.69 to 17.56)	6.78(4.45 to	-0.43(-0.64 to -0.13)

	causes					10.08)	
Cook Islands	Liver cancer	51(41 to 61)	71(55 to 87)	0.4(0.04 to 0.86)	360.57(296.86 to 431.52)	295.21(230.25 to 366.11)	-0.18(-0.39 to 0.09)
Cook Islands	Liver cancer due to alcohol use	5(3 to 8)	11(7 to 17)	1.08(0.52 to 1.82)	40.61(24.6 to 59.51)	46.03(28.6 to 68.37)	0.13(-0.16 to 0.54)
Cook Islands	Liver cancer due to hepatitis B	30(22 to 39)	37(27 to 49)	0.24(-0.11 to 0.71)	204.79(154.33 to 268.41)	157.28(114.42 to 210.44)	-0.23(-0.45 to 0.08)
Cook Islands	Liver cancer due to hepatitis C	8(5 to 12)	12(8 to 18)	0.44(0.1 to 0.9)	66.92(43.31 to 93.94)	48.56(31.75 to 70.03)	-0.27(-0.44 to -0.06)
Cook Islands	Liver cancer due to NASH	4(3 to 6)	8(5 to 11)	0.86(0.37 to 1.48)	29.84(20.75 to 42.27)	30.96(20.17 to 44.07)	0.04(-0.22 to 0.38)
Cook Islands	Liver cancer due to other causes	3(2 to 4)	3(2 to 4)	-0.05(-0.33 to 0.31)	18.41(12.81 to 25.66)	12.38(7.87 to 17.76)	-0.33(-0.52 to -0.08)
Costa Rica	Liver cancer	2510(2343 to 2679)	6199(4745 to 7945)	1.47(0.87 to 2.2)	133.48(123.93 to 142.67)	120.73(92.6 to 154.27)	-0.1(-0.31 to 0.17)
Costa Rica	Liver cancer due to alcohol use	803(590 to 1020)	2154(1438 to 3086)	1.68(1.01 to 2.53)	44.7(32.7 to 56.95)	41.63(27.79 to 59.87)	-0.07(-0.3 to 0.22)
Costa Rica	Liver cancer due to hepatitis B	527(399 to 690)	1123(727 to 1701)	1.13(0.59 to 1.8)	25.69(18.81 to 34.38)	21.49(14.02 to 32.32)	-0.16(-0.37 to 0.1)
Costa Rica	Liver cancer due to hepatitis C	769(573 to 991)	1986(1317 to 2809)	1.58(0.98 to 2.37)	44.46(33.2 to 57.51)	38.77(25.67 to 54.88)	-0.13(-0.33 to 0.14)
Costa Rica	Liver cancer due to NASH	167(119 to 227)	530(344 to 794)	2.18(1.38 to 3.21)	8.95(6.24 to 12.52)	10.36(6.76 to 15.52)	0.16(-0.14 to 0.53)
Costa Rica	Liver cancer due to other	243(198 to 294)	407(281 to 583)	0.67(0.23 to 1.24)	9.7(7.6 to 12.27)	8.48(6 to 11.93)	-0.13(-0.33 to 0.14)

	causes						
Croatia	Liver cancer	4848(4479 to 5236)	6534(5050 to 8391)	0.35(0.02 to 0.76)	75.16(69.57 to 80.96)	81.58(62.78 to 105.41)	0.09(-0.18 to 0.42)
Croatia	Liver cancer due to alcohol use	2102(1601 to 2605)	3137(2176 to 4265)	0.49(0.12 to 0.99)	31.95(24.44 to 39.29)	38.05(26.32 to 52.21)	0.19(-0.1 to 0.58)
Croatia	Liver cancer due to hepatitis B	1279(928 to 1740)	1471(952 to 2205)	0.15(-0.15 to 0.53)	19.8(14.6 to 26.56)	19.95(13.26 to 29.57)	0.01(-0.25 to 0.35)
Croatia	Liver cancer due to hepatitis C	862(566 to 1203)	1112(702 to 1649)	0.29(0 to 0.69)	13.28(8.85 to 18.35)	12.77(7.98 to 19.04)	-0.04(-0.26 to 0.25)
Croatia	Liver cancer due to NASH	368(258 to 515)	570(369 to 862)	0.55(0.15 to 1.05)	5.75(4.07 to 7.96)	6.74(4.43 to 10.02)	0.17(-0.13 to 0.56)
Croatia	Liver cancer due to other causes	236(174 to 315)	244(156 to 359)	0.03(-0.22 to 0.34)	4.37(3.39 to 5.56)	4.06(2.85 to 5.57)	-0.07(-0.28 to 0.19)
Cuba	Liver cancer	16383(15561 to 17189)	9734(7739 to 12139)	-0.41(-0.53 to -0.27)	157.96(150.08 to 165.86)	53.44(42.7 to 66.31)	-0.66(-0.73 to -0.58)
Cuba	Liver cancer due to alcohol use	5100(3770 to 6508)	3617(2490 to 4997)	-0.29(-0.45 to -0.1)	49.3(36.29 to 63.1)	19.31(13.25 to 26.73)	-0.61(-0.7 to -0.5)
Cuba	Liver cancer due to hepatitis B	5254(4044 to 6725)	2637(1789 to 3819)	-0.5(-0.61 to -0.37)	50.4(38.59 to 65.13)	14.82(10.2 to 21.29)	-0.71(-0.77 to -0.63)
Cuba	Liver cancer due to hepatitis C	3413(2368 to 4494)	1983(1269 to 2902)	-0.42(-0.54 to -0.29)	33.06(22.95 to 43.88)	10.36(6.61 to 15.19)	-0.69(-0.75 to -0.61)
Cuba	Liver cancer due to NASH	1312(939 to 1773)	926(601 to 1397)	-0.29(-0.46 to -0.09)	12.51(8.96 to 17.07)	4.97(3.25 to 7.44)	-0.6(-0.69 to -0.49)
Cuba	Liver cancer due to other	1303(1004 to 1681)	570(381 to 815)	-0.56(-0.66 to -0.43)	12.69(9.76 to 16.31)	3.99(2.91 to	-0.69(-0.75 to -0.6)

	causes					5.39)	
Cyprus	Liver cancer	607(500 to 714)	1319(1138 to 1521)	1.17(0.76 to 1.75)	74.12(61.76 to 86.47)	69.91(60.41 to 80.27)	-0.06(-0.23 to 0.18)
Cyprus	Liver cancer due to alcohol use	225(157 to 301)	513(375 to 671)	1.28(0.76 to 2.01)	26.47(18.65 to 35.37)	26.34(19.24 to 34.55)	0(-0.23 to 0.3)
Cyprus	Liver cancer due to hepatitis B	104(71 to 150)	184(126 to 265)	0.77(0.37 to 1.28)	12.74(8.73 to 18.17)	10.12(6.87 to 14.45)	-0.21(-0.38 to 0.02)
Cyprus	Liver cancer due to hepatitis C	204(141 to 276)	470(335 to 609)	1.3(0.84 to 1.96)	25.58(18.17 to 33.98)	24.6(17.9 to 31.74)	-0.04(-0.23 to 0.22)
Cyprus	Liver cancer due to NASH	31(20 to 44)	79(52 to 116)	1.57(1.02 to 2.39)	3.76(2.56 to 5.26)	4.13(2.77 to 6.01)	0.1(-0.13 to 0.42)
Cyprus	Liver cancer due to other causes	43(33 to 57)	73(52 to 102)	0.69(0.34 to 1.12)	5.56(4.28 to 7.18)	4.72(3.52 to 6.36)	-0.15(-0.32 to 0.07)
Czechia	Liver cancer	14809(14023 to 15516)	13268(10734 to 16441)	-0.1(-0.28 to 0.13)	109.93(104.14 to 115.42)	68.4(55.03 to 85.08)	-0.38(-0.5 to -0.21)
Czechia	Liver cancer due to alcohol use	6788(5410 to 8079)	6552(4726 to 8632)	-0.03(-0.24 to 0.22)	49.47(39.17 to 58.8)	32.64(23.57 to 43.07)	-0.34(-0.48 to -0.17)
Czechia	Liver cancer due to hepatitis B	3618(2641 to 4946)	2600(1727 to 3857)	-0.28(-0.45 to -0.07)	27.62(20.43 to 37.43)	14.66(9.93 to 21.43)	-0.47(-0.59 to -0.31)
Czechia	Liver cancer due to hepatitis C	2966(2011 to 4033)	2772(1764 to 4113)	-0.07(-0.26 to 0.18)	21.33(14.62 to 28.66)	13.33(8.64 to 19.49)	-0.38(-0.5 to -0.21)
Czechia	Liver cancer due to NASH	859(600 to 1241)	914(609 to 1388)	0.06(-0.17 to 0.36)	6.25(4.4 to 8.9)	4.56(3.1 to 6.8)	-0.27(-0.43 to -0.07)
Czechia	Liver cancer due to other causes	578(444 to 762)	431(292 to 615)	-0.25(-0.41 to -0.04)	5.25(4.21 to 6.59)	3.21(2.34 to 4.24)	-0.39(-0.51 to -0.22)

Democratic People's Republic of Korea	Liver cancer	83295(62368 to 111514)	99151(71505 to 134805)	0.19(-0.22 to 0.73)	437.07(332.88 to 577.51)	299.93(216.26 to 405.84)	-0.31(-0.55 to -0.02)
Democratic People's Republic of Korea	Liver cancer due to alcohol use	5457(3170 to 9267)	7565(4193 to 12209)	0.39(-0.1 to 1.03)	29.55(17.69 to 48)	22.62(12.69 to 36)	-0.23(-0.5 to 0.1)
Democratic People's Republic of Korea	Liver cancer due to hepatitis B	55211(38995 to 76021)	64006(43224 to 91694)	0.16(-0.27 to 0.77)	280.73(201.59 to 382.89)	191.79(129.84 to 270.45)	-0.32(-0.56 to 0.03)
Democratic People's Republic of Korea	Liver cancer due to hepatitis C	12518(7563 to 18529)	17232(10872 to 24805)	0.38(-0.02 to 0.88)	76.12(48 to 109.78)	52.83(33.78 to 75.72)	-0.31(-0.5 to -0.07)
Democratic People's Republic of Korea	Liver cancer due to NASH	3205(2044 to 5104)	4242(2550 to 6772)	0.32(-0.12 to 0.88)	18.03(11.61 to 27.66)	12.9(7.88 to 20.44)	-0.28(-0.51 to 0)
Democratic People's Republic of Korea	Liver cancer due to other causes	6903(4510 to 9852)	6106(3711 to 9210)	-0.12(-0.43 to 0.3)	32.64(21.52 to 46.36)	19.79(12.72 to 28.93)	-0.39(-0.6 to -0.12)
Democratic Republic of the Congo	Liver cancer	19838(15250 to 26206)	33433(23337 to 46906)	0.69(0.09 to 1.48)	73.3(59.19 to 88.98)	61.97(46.76 to 81.46)	-0.15(-0.4 to 0.18)

Democratic Republic of the Congo	Liver cancer due to alcohol use	1485(937 to 2205)	3061(1786 to 4798)	1.06(0.41 to 2.04)	8.45(5.35 to 12.25)	7.42(4.41 to 11.32)	-0.12(-0.39 to 0.27)
Democratic Republic of the Congo	Liver cancer due to hepatitis B	4442(3114 to 6093)	8992(5957 to 13243)	1.02(0.33 to 2.04)	19.34(13.29 to 26.91)	16.02(10.47 to 24.15)	-0.17(-0.44 to 0.21)
Democratic Republic of the Congo	Liver cancer due to hepatitis C	4740(3388 to 6324)	9730(6709 to 13644)	1.05(0.42 to 1.99)	28.75(21.23 to 37.43)	25.71(17.91 to 35.19)	-0.11(-0.36 to 0.27)
Democratic Republic of the Congo	Liver cancer due to NASH	754(500 to 1100)	1765(1123 to 2687)	1.34(0.59 to 2.41)	3.93(2.61 to 5.77)	3.89(2.52 to 6.01)	-0.01(-0.31 to 0.42)
Democratic Republic of the Congo	Liver cancer due to other causes	8416(5069 to 13902)	9886(4483 to 18750)	0.17(-0.5 to 1.5)	12.83(8.52 to 19.71)	8.92(4.7 to 15.57)	-0.31(-0.65 to 0.33)
Denmark	Liver cancer	3378(3214 to 3553)	7743(6956 to 8536)	1.29(1.04 to 1.55)	45.68(43.42 to 48.08)	75.48(67.87 to 83.25)	0.65(0.47 to 0.85)
Denmark	Liver cancer due to alcohol use	1381(1067 to 1702)	3144(2307 to 4017)	1.28(0.95 to 1.61)	18.21(14.13 to 22.49)	29.74(21.85 to 37.98)	0.63(0.4 to 0.88)
Denmark	Liver cancer due to hepatitis B	506(352 to 700)	1162(779 to 1674)	1.3(0.98 to 1.66)	7.35(5.19 to 10.19)	12.66(8.71 to 17.84)	0.72(0.49 to 0.98)
Denmark	Liver cancer due to hepatitis C	1108(808 to 1435)	2575(1861 to 3423)	1.32(1.05 to 1.61)	14.15(10.27 to 18.34)	23.58(16.9 to 31.67)	0.67(0.47 to 0.87)
Denmark	Liver cancer due to NASH	177(121 to 258)	456(308 to 663)	1.58(1.18 to 2.04)	2.27(1.57 to 3.29)	4.27(2.97 to 6.16)	0.88(0.6 to 1.21)
Denmark	Liver cancer due to other causes	206(156 to 273)	406(291 to 561)	0.97(0.65 to 1.28)	3.7(2.99 to 4.6)	5.22(3.98 to 6.78)	0.41(0.2 to 0.63)

Djibouti	Liver cancer	193(125 to 334)	658(382 to 1107)	2.41(1.28 to 4.09)	87.06(55.78 to 153.4)	86.67(53.59 to 144.37)	0(-0.31 to 0.42)
Djibouti	Liver cancer due to alcohol use	31(14 to 68)	130(65 to 257)	3.22(1.77 to 5.41)	19.17(9.31 to 42.16)	19.72(10.26 to 38.07)	0.03(-0.3 to 0.51)
Djibouti	Liver cancer due to hepatitis B	66(36 to 130)	240(124 to 430)	2.62(1.33 to 4.85)	30.07(16.27 to 58.91)	28.53(15.36 to 50.81)	-0.05(-0.37 to 0.44)
Djibouti	Liver cancer due to hepatitis C	25(14 to 43)	105(57 to 183)	3.15(1.84 to 5.15)	18.04(10.57 to 30.17)	17.91(10.4 to 30.29)	-0.01(-0.29 to 0.39)
Djibouti	Liver cancer due to NASH	15(8 to 26)	69(36 to 123)	3.54(2.01 to 5.96)	9.04(4.99 to 15.46)	10.4(5.83 to 18.4)	0.15(-0.2 to 0.65)
Djibouti	Liver cancer due to other causes	55(34 to 92)	114(65 to 185)	1.06(0.11 to 2.49)	10.75(6.92 to 16.82)	10.11(6.01 to 16.19)	-0.06(-0.41 to 0.45)
Dominica	Liver cancer	151(131 to 171)	70(56 to 88)	-0.54(-0.64 to -0.4)	221.57(191.36 to 253.56)	80.73(63.89 to 100.87)	-0.64(-0.72 to -0.53)
Dominica	Liver cancer due to alcohol use	48(34 to 64)	25(17 to 35)	-0.48(-0.61 to -0.32)	70.49(49.86 to 93.94)	28.16(19.09 to 39.16)	-0.6(-0.7 to -0.47)
Dominica	Liver cancer due to hepatitis B	46(34 to 61)	20(14 to 29)	-0.56(-0.67 to -0.42)	71.96(53.03 to 97.08)	24.13(16.4 to 33.75)	-0.66(-0.75 to -0.56)
Dominica	Liver cancer due to hepatitis C	32(22 to 44)	13(9 to 20)	-0.58(-0.67 to -0.45)	44.64(30.69 to 61.78)	14.83(9.59 to 21.8)	-0.67(-0.74 to -0.58)
Dominica	Liver cancer due to NASH	12(8 to 17)	6(4 to 9)	-0.48(-0.6 to -0.31)	16.91(11.93 to 23.3)	7.1(4.78 to 10.27)	-0.58(-0.68 to -0.45)
Dominica	Liver cancer due to other causes	12(9 to 16)	5(3 to 7)	-0.6(-0.69 to -0.48)	17.58(12.9 to 23.64)	6.52(4.58 to 8.95)	-0.63(-0.72 to -0.5)

Dominican Republic	Liver cancer	4918(4318 to 5568)	12028(7956 to 18762)	1.45(0.59 to 2.86)	108.03(94.5 to 122.34)	123.21(81.83 to 190.9)	0.14(-0.27 to 0.81)
Dominican Republic	Liver cancer due to alcohol use	1296(938 to 1721)	3918(2271 to 6891)	2.02(0.87 to 3.93)	32.52(23.47 to 43.23)	41.05(24.26 to 70.98)	0.26(-0.22 to 1.07)
Dominican Republic	Liver cancer due to hepatitis B	1735(1323 to 2201)	3988(2298 to 6730)	1.3(0.4 to 2.81)	36.46(27.44 to 47.21)	39.24(22.5 to 66.3)	0.08(-0.34 to 0.78)
Dominican Republic	Liver cancer due to hepatitis C	732(499 to 1005)	1982(1189 to 3227)	1.71(0.78 to 3.07)	19.73(13.68 to 27.11)	21.5(13.11 to 35.01)	0.09(-0.29 to 0.62)
Dominican Republic	Liver cancer due to NASH	297(210 to 411)	950(567 to 1513)	2.2(1.04 to 3.93)	7.02(4.98 to 9.89)	9.91(5.99 to 15.73)	0.41(-0.09 to 1.17)
Dominican Republic	Liver cancer due to other causes	858(691 to 1048)	1191(810 to 1684)	0.39(-0.06 to 0.99)	12.3(9.87 to 15.22)	11.5(7.88 to 16.34)	-0.06(-0.35 to 0.29)
Ecuador	Liver cancer	4566(4115 to 5018)	12453(9780 to 16102)	1.73(1.09 to 2.56)	73.81(66.19 to 81.37)	80.6(63.52 to 104.2)	0.09(-0.16 to 0.43)
Ecuador	Liver cancer due to alcohol use	930(650 to 1245)	3393(2245 to 4906)	2.65(1.75 to 3.88)	17.08(11.99 to 22.97)	22.38(14.78 to 32.22)	0.31(0 to 0.75)
Ecuador	Liver cancer due to hepatitis B	2310(1909 to 2739)	5658(3992 to 7597)	1.45(0.83 to 2.24)	37.37(30.2 to 44.87)	35.97(25.47 to 48.52)	-0.04(-0.28 to 0.28)
Ecuador	Liver cancer due to hepatitis C	250(156 to 368)	826(494 to 1273)	2.31(1.43 to 3.38)	4.87(3.05 to 7.17)	5.68(3.4 to 8.65)	0.16(-0.14 to 0.54)
Ecuador	Liver cancer due to NASH	359(257 to 491)	1301(865 to 1897)	2.62(1.62 to 3.85)	6.47(4.55 to 8.98)	8.71(5.82 to 12.76)	0.35(-0.02 to 0.8)
Ecuador	Liver cancer due to other causes	716(574 to 884)	1274(947 to 1708)	0.78(0.3 to 1.32)	8.01(6.2 to 10.13)	7.86(5.8 to 10.53)	-0.02(-0.25 to 0.26)
Egypt	Liver cancer	157326(129738 to 187905)	388986(274355 to 541114)	1.47(0.66 to 2.79)	457.68(366.19 to 548.17)	537.85(382.38 to	0.18(-0.22 to 0.84)

						742.42)	
Egypt	Liver cancer due to alcohol use	10983(6429 to 17349)	34334(18728 to 59913)	2.13(0.95 to 4.06)	33.66(19.89 to 52.63)	47.16(25.93 to 81.71)	0.4(-0.12 to 1.27)
Egypt	Liver cancer due to hepatitis B	26479(18135 to 37989)	59485(35298 to 92500)	1.25(0.46 to 2.56)	71.7(47.94 to 105.69)	74.42(44.46 to 117.79)	0.04(-0.32 to 0.63)
Egypt	Liver cancer due to hepatitis C	90846(67951 to 114018)	231262(151517 to 333273)	1.55(0.64 to 3)	288.88(218.04 to 357.98)	333.1(224.95 to 473.1)	0.15(-0.25 to 0.82)
Egypt	Liver cancer due to NASH	9382(6272 to 13833)	34863(20698 to 57504)	2.72(1.41 to 4.99)	28.36(18.67 to 42.67)	48.58(29.25 to 77.8)	0.71(0.12 to 1.76)
Egypt	Liver cancer due to other causes	19635(13650 to 29259)	29042(18730 to 43478)	0.48(-0.11 to 1.31)	35.08(25.71 to 47.9)	34.59(22.41 to 52.19)	-0.01(-0.34 to 0.46)
El Salvador	Liver cancer	2976(2778 to 3192)	2789(2100 to 3650)	-0.06(-0.31 to 0.24)	89.97(83.39 to 96.69)	47.57(35.66 to 62.47)	-0.47(-0.61 to -0.3)
El Salvador	Liver cancer due to alcohol use	682(482 to 905)	861(554 to 1261)	0.26(-0.09 to 0.69)	22.33(15.85 to 29.58)	14.84(9.5 to 21.66)	-0.34(-0.52 to -0.11)
El Salvador	Liver cancer due to hepatitis B	677(514 to 874)	500(319 to 761)	-0.26(-0.48 to 0)	19.41(14.49 to 25.69)	8.5(5.36 to 13.06)	-0.56(-0.69 to -0.41)
El Salvador	Liver cancer due to hepatitis C	968(718 to 1222)	960(634 to 1350)	-0.01(-0.27 to 0.32)	32.77(24.51 to 41.45)	16.36(10.79 to 22.94)	-0.5(-0.63 to -0.33)
El Salvador	Liver cancer due to NASH	204(148 to 281)	244(153 to 373)	0.2(-0.15 to 0.63)	6.3(4.41 to 8.84)	4.12(2.57 to 6.34)	-0.35(-0.53 to -0.11)
El Salvador	Liver cancer due to other causes	445(368 to 529)	224(154 to 311)	-0.5(-0.65 to -0.31)	9.16(7.31 to 11.41)	3.75(2.58 to 5.26)	-0.59(-0.71 to -0.44)
Equatorial Guinea	Liver cancer	248(180 to 323)	533(302 to 839)	1.15(0.2 to 2.66)	88.1(64.07 to 116.09)	81.83(46.68 to	-0.07(-0.5 to 0.52)

						122.74)	
Equatorial Guinea	Liver cancer due to alcohol use	24(14 to 38)	74(37 to 126)	2.07(0.54 to 4.37)	11.05(6.46 to 17.25)	14.11(7.09 to 24.02)	0.28(-0.35 to 1.19)
Equatorial Guinea	Liver cancer due to hepatitis B	68(45 to 97)	175(93 to 287)	1.58(0.36 to 3.46)	25.21(16.47 to 37.4)	22.38(11.77 to 36.5)	-0.11(-0.54 to 0.51)
Equatorial Guinea	Liver cancer due to hepatitis C	75(48 to 109)	151(82 to 242)	1.01(0.08 to 2.33)	36.55(24.42 to 52.31)	31.74(17.71 to 49.79)	-0.13(-0.54 to 0.4)
Equatorial Guinea	Liver cancer due to NASH	11(7 to 18)	41(22 to 68)	2.61(0.98 to 5.14)	4.93(2.93 to 7.81)	6.8(3.75 to 11.3)	0.38(-0.25 to 1.26)
Equatorial Guinea	Liver cancer due to other causes	69(42 to 109)	92(46 to 169)	0.34(-0.37 to 1.6)	10.36(6.85 to 14.99)	6.79(3.75 to 11.36)	-0.34(-0.65 to 0.18)
Eritrea	Liver cancer	1299(871 to 1964)	2956(1992 to 4479)	1.28(0.51 to 2.36)	90.6(60.37 to 138.45)	83.8(57.57 to 122.24)	-0.07(-0.36 to 0.34)
Eritrea	Liver cancer due to alcohol use	219(117 to 399)	546(298 to 956)	1.49(0.68 to 2.7)	19.37(10.45 to 34.95)	18.05(10.12 to 30.75)	-0.07(-0.36 to 0.38)
Eritrea	Liver cancer due to hepatitis B	455(281 to 722)	1024(603 to 1641)	1.25(0.48 to 2.43)	31.18(19.15 to 49.21)	26.15(15.57 to 40.92)	-0.16(-0.44 to 0.24)
Eritrea	Liver cancer due to hepatitis C	196(103 to 341)	509(305 to 786)	1.6(0.75 to 3.1)	19.75(10.66 to 34.11)	19.54(11.94 to 29.19)	-0.01(-0.33 to 0.52)
Eritrea	Liver cancer due to NASH	106(61 to 175)	297(183 to 472)	1.8(0.81 to 3.4)	9.31(5.4 to 15.78)	9.84(6.13 to 15.89)	0.06(-0.3 to 0.62)
Eritrea	Liver cancer due to other causes	323(196 to 509)	580(362 to 917)	0.8(0 to 2.15)	10.99(6.74 to 16.94)	10.23(6.66 to 15.36)	-0.07(-0.43 to 0.48)
Estonia	Liver cancer	1084(1004 to 1166)	1962(1487 to 2498)	0.81(0.39 to 1.29)	54.94(50.58 to 59.17)	84.7(64.42 to 108.76)	0.54(0.18 to 0.97)

Estonia	Liver cancer due to alcohol use	384(283 to 480)	889(629 to 1194)	1.32(0.72 to 2.05)	18.74(13.92 to 23.46)	37.65(26.41 to 51.66)	1.01(0.48 to 1.65)
Estonia	Liver cancer due to hepatitis B	297(216 to 402)	395(248 to 593)	0.33(-0.02 to 0.75)	15.11(11.27 to 20.05)	18.95(12.14 to 28.17)	0.25(-0.09 to 0.66)
Estonia	Liver cancer due to hepatitis C	253(174 to 340)	456(291 to 660)	0.8(0.36 to 1.34)	12.21(8.46 to 16.36)	17.66(11.25 to 25.67)	0.45(0.09 to 0.86)
Estonia	Liver cancer due to NASH	70(49 to 100)	152(97 to 232)	1.17(0.6 to 1.88)	3.46(2.42 to 4.88)	6.14(3.93 to 9.47)	0.78(0.32 to 1.34)
Estonia	Liver cancer due to other causes	81(52 to 109)	70(47 to 102)	-0.13(-0.44 to 0.39)	5.42(3.34 to 7.37)	4.3(3.12 to 5.82)	-0.21(-0.5 to 0.36)
Eswatini	Liver cancer	564(352 to 1121)	3861(1052 to 7406)	5.85(0.12 to 16.39)	155.18(98.26 to 303.44)	534.72(151.32 to 1000.58)	2.45(-0.42 to 7.48)
Eswatini	Liver cancer due to alcohol use	93(46 to 226)	810(188 to 1680)	7.71(0.17 to 22.23)	28.16(14.08 to 66.86)	121.51(29.28 to 251.56)	3.31(-0.4 to 10.03)
Eswatini	Liver cancer due to hepatitis B	257(144 to 556)	1937(441 to 3839)	6.54(0.01 to 19.93)	64.72(35.76 to 141.25)	244.6(57.08 to 474.87)	2.78(-0.49 to 9.23)
Eswatini	Liver cancer due to hepatitis C	116(68 to 206)	585(189 to 1127)	4.02(0.22 to 10.52)	39.23(23.32 to 68.87)	97.57(32.34 to 187.53)	1.49(-0.36 to 4.55)
Eswatini	Liver cancer due to NASH	47(27 to 88)	319(94 to 615)	5.78(0.39 to 15.72)	13.74(7.78 to 26.28)	46.53(14.41 to 88.12)	2.39(-0.27 to 6.94)
Eswatini	Liver cancer due to other causes	50(32 to 84)	211(72 to 413)	3.19(0.03 to 9.27)	9.33(5.59 to 16.45)	24.51(8.15 to 48.51)	1.63(-0.37 to 5.27)
Ethiopia	Liver cancer	24737(16471 to 38489)	36981(29047 to 46872)	0.49(-0.19 to 1.5)	82(56.98 to 117.09)	69.81(55.12 to 88.58)	-0.15(-0.51 to 0.32)

Ethiopia	Liver cancer due to alcohol use	3606(2380 to 5327)	6465(4713 to 8846)	0.79(0.01 to 1.88)	16.75(11.14 to 24.16)	15.26(11.19 to 20.88)	-0.09(-0.47 to 0.43)
Ethiopia	Liver cancer due to hepatitis B	5434(3557 to 8322)	9148(6808 to 12144)	0.68(-0.09 to 1.8)	20.01(13.25 to 29.88)	16.61(12.29 to 22.25)	-0.17(-0.54 to 0.33)
Ethiopia	Liver cancer due to hepatitis C	5183(3386 to 7577)	9282(7144 to 12015)	0.79(0.06 to 1.83)	25.78(17.49 to 36.53)	23.29(17.94 to 29.7)	-0.1(-0.44 to 0.38)
Ethiopia	Liver cancer due to NASH	1613(1038 to 2470)	3040(2351 to 3993)	0.88(0.07 to 2.09)	6.98(4.62 to 10.29)	6.58(5.11 to 8.7)	-0.06(-0.43 to 0.46)
Ethiopia	Liver cancer due to other causes	8902(5071 to 16119)	9046(6547 to 12659)	0.02(-0.53 to 1.04)	12.48(7.73 to 21.32)	8.07(6.12 to 10.62)	-0.35(-0.68 to 0.18)
Fiji	Liver cancer	711(573 to 892)	1414(1062 to 1846)	0.99(0.39 to 1.84)	157.57(126.95 to 196.23)	166.77(126.43 to 214.9)	0.06(-0.25 to 0.53)
Fiji	Liver cancer due to alcohol use	74(44 to 113)	176(102 to 281)	1.39(0.63 to 2.54)	18.15(11.18 to 27.94)	21.16(12.62 to 33.21)	0.17(-0.19 to 0.72)
Fiji	Liver cancer due to hepatitis B	422(315 to 543)	790(558 to 1099)	0.87(0.29 to 1.71)	87.2(65.3 to 113.08)	89.06(63.78 to 122.76)	0.02(-0.29 to 0.47)
Fiji	Liver cancer due to hepatitis C	112(69 to 168)	237(147 to 354)	1.11(0.5 to 2.15)	31.07(19.77 to 45.07)	31.14(20.08 to 45.61)	0(-0.28 to 0.46)
Fiji	Liver cancer due to NASH	51(33 to 76)	127(79 to 192)	1.48(0.68 to 2.66)	12.15(7.89 to 17.98)	15.78(10.15 to 23.42)	0.3(-0.1 to 0.87)
Fiji	Liver cancer due to other causes	52(38 to 73)	85(57 to 122)	0.63(0.12 to 1.4)	9.01(6.24 to 12.74)	9.64(6.5 to 13.71)	0.07(-0.24 to 0.57)
Finland	Liver cancer	4587(4306 to 4881)	9501(8649 to 10443)	1.07(0.85 to 1.33)	67.08(62.95 to 71.44)	85.82(78.22 to 94.53)	0.28(0.14 to 0.45)
Finland	Liver cancer due to alcohol use	1718(1301 to 2136)	3778(2801 to 4788)	1.2(0.9 to 1.59)	24.77(18.63 to 30.86)	33.2(25.02 to	0.34(0.16 to 0.57)

						42.05)	
Finland	Liver cancer due to hepatitis B	751(535 to 1041)	1279(860 to 1890)	0.7(0.39 to 1.03)	11.58(8.29 to 15.76)	13.51(9.57 to 18.93)	0.17(-0.02 to 0.37)
Finland	Liver cancer due to hepatitis C	1581(1177 to 2025)	3372(2458 to 4414)	1.13(0.88 to 1.44)	22.24(16.41 to 28.8)	28.19(20.69 to 36.83)	0.27(0.12 to 0.44)
Finland	Liver cancer due to NASH	256(175 to 375)	610(414 to 887)	1.38(0.98 to 1.84)	3.63(2.53 to 5.25)	5.29(3.68 to 7.6)	0.46(0.22 to 0.73)
Finland	Liver cancer due to other causes	281(211 to 373)	463(322 to 652)	0.65(0.36 to 1.01)	4.87(3.82 to 6.21)	5.64(4.29 to 7.33)	0.16(0 to 0.36)
France	Liver cancer	91051(86775 to 95187)	155465(136112 to 176599)	0.71(0.48 to 0.95)	119.18(113.71 to 124.6)	132.36(116.22 to 150.91)	0.11(-0.03 to 0.27)
France	Liver cancer due to alcohol use	34888(25438 to 44241)	53974(37675 to 71884)	0.55(0.3 to 0.83)	45.28(33.17 to 57.39)	45.72(32.01 to 61)	0.01(-0.15 to 0.19)
France	Liver cancer due to hepatitis B	14338(9901 to 20228)	21937(14247 to 32085)	0.53(0.3 to 0.81)	19.84(13.89 to 27.74)	21.14(14.3 to 30.43)	0.07(-0.09 to 0.26)
France	Liver cancer due to hepatitis C	33141(24172 to 43784)	63013(46686 to 80864)	0.9(0.62 to 1.23)	41.86(30.36 to 54.9)	50.31(36.98 to 65.29)	0.2(0.03 to 0.41)
France	Liver cancer due to NASH	4161(2859 to 6199)	9158(6122 to 13675)	1.2(0.81 to 1.65)	5.28(3.66 to 7.78)	7.45(5.09 to 11.05)	0.41(0.16 to 0.7)
France	Liver cancer due to other causes	4523(3359 to 6157)	7384(5180 to 10296)	0.63(0.37 to 0.93)	6.93(5.39 to 9.02)	7.73(5.69 to 10.36)	0.12(-0.05 to 0.29)
Gabon	Liver cancer	661(477 to 880)	1038(690 to 1441)	0.57(0 to 1.41)	94.91(68.7 to 123.24)	86.04(57.22 to 120.6)	-0.09(-0.42 to 0.37)
Gabon	Liver cancer due to alcohol use	90(50 to 138)	174(92 to 306)	0.94(0.14 to 2.02)	15.03(8.48 to 22.88)	15.34(8.07 to 27.13)	0.02(-0.37 to 0.6)

Gabon	Liver cancer due to hepatitis B	159(104 to 227)	279(163 to 437)	0.75(0.05 to 1.84)	23.03(14.9 to 33)	20.56(11.97 to 32.47)	-0.11(-0.45 to 0.4)
Gabon	Liver cancer due to hepatitis C	232(157 to 316)	388(237 to 581)	0.67(0.06 to 1.59)	40.53(27.94 to 54.43)	36.57(22.81 to 54.51)	-0.1(-0.43 to 0.39)
Gabon	Liver cancer due to NASH	33(21 to 50)	78(46 to 118)	1.36(0.45 to 2.64)	5.37(3.41 to 8.1)	6.63(3.97 to 10.16)	0.24(-0.23 to 0.93)
Gabon	Liver cancer due to other causes	147(77 to 246)	120(70 to 196)	-0.19(-0.54 to 0.4)	10.95(6.46 to 17.14)	6.95(4.28 to 10.88)	-0.37(-0.61 to 0.02)
Gambia	Liver cancer	4268(3049 to 5721)	13506(9531 to 18084)	2.16(1.13 to 3.74)	876.47(636.2 to 1170.09)	1116.06(789.18 to 1480.33)	0.27(-0.15 to 0.91)
Gambia	Liver cancer due to alcohol use	577(349 to 896)	2144(1222 to 3356)	2.72(1.36 to 4.79)	142.87(89.09 to 221.36)	201.49(114.57 to 311.95)	0.41(-0.1 to 1.14)
Gambia	Liver cancer due to hepatitis B	2439(1685 to 3412)	7760(5371 to 11068)	2.18(1.05 to 4.03)	523.14(368.42 to 720.49)	627.55(428.18 to 889.39)	0.2(-0.23 to 0.85)
Gambia	Liver cancer due to hepatitis C	345(204 to 523)	1274(765 to 1936)	2.69(1.39 to 4.48)	95.67(58 to 142.41)	130.23(79.25 to 195.59)	0.36(-0.09 to 0.99)
Gambia	Liver cancer due to NASH	213(136 to 318)	903(570 to 1356)	3.23(1.76 to 5.42)	52.58(33.51 to 79.21)	82.59(51.09 to 126.76)	0.57(0.04 to 1.3)
Gambia	Liver cancer due to other causes	695(448 to 1026)	1425(933 to 2045)	1.05(0.23 to 2.38)	62.2(41.19 to 89.45)	74.2(50.84 to 103.39)	0.19(-0.21 to 0.8)
Georgia	Liver cancer	1817(1635 to 2020)	5131(4162 to 6347)	1.82(1.2 to 2.6)	30.05(26.88 to 33.46)	96.41(77.82 to 119.79)	2.21(1.47 to 3.12)
Georgia	Liver cancer due to alcohol use	447(302 to 602)	1739(1212 to 2417)	2.89(2.03 to 4.08)	7.04(4.84 to 9.46)	31.82(21.95 to 44.05)	3.52(2.49 to 4.9)

Georgia	Liver cancer due to hepatitis B	452(330 to 620)	1380(946 to 1981)	2.05(1.39 to 2.9)	7.19(5.3 to 9.72)	27.96(19.43 to 39.39)	2.89(2.01 to 4)
Georgia	Liver cancer due to hepatitis C	556(403 to 726)	1475(1020 to 2055)	1.65(1.13 to 2.28)	9(6.66 to 11.56)	25.52(17.58 to 35.59)	1.83(1.24 to 2.54)
Georgia	Liver cancer due to NASH	110(76 to 158)	333(226 to 476)	2.03(1.38 to 2.91)	1.8(1.27 to 2.51)	6.06(4.15 to 8.58)	2.37(1.61 to 3.35)
Georgia	Liver cancer due to other causes	252(151 to 361)	203(137 to 288)	-0.19(-0.55 to 0.57)	5.01(2.95 to 7.27)	5.06(3.36 to 7.36)	0.01(-0.47 to 1.1)
Germany	Liver cancer	66749(61838 to 71961)	149719(137484 to 161942)	1.24(0.98 to 1.51)	56.4(52.46 to 60.73)	89.01(81.81 to 96.21)	0.58(0.4 to 0.77)
Germany	Liver cancer due to alcohol use	29743(23937 to 35631)	71612(57951 to 85613)	1.41(1.05 to 1.78)	24.67(19.83 to 29.54)	42.23(34.13 to 50.86)	0.71(0.46 to 0.98)
Germany	Liver cancer due to hepatitis B	9288(6747 to 12826)	16020(10844 to 23195)	0.72(0.43 to 1.07)	8.3(6.14 to 11.35)	10.74(7.55 to 15.34)	0.29(0.09 to 0.53)
Germany	Liver cancer due to hepatitis C	18364(14040 to 23462)	41231(30215 to 53410)	1.25(0.95 to 1.53)	14.64(11.17 to 18.71)	22.56(16.38 to 30.02)	0.54(0.34 to 0.74)
Germany	Liver cancer due to NASH	4438(3133 to 6149)	11850(8320 to 16519)	1.67(1.25 to 2.11)	3.59(2.56 to 4.95)	6.66(4.78 to 9.28)	0.85(0.58 to 1.15)
Germany	Liver cancer due to other causes	4917(3769 to 6365)	9006(6430 to 12419)	0.83(0.54 to 1.13)	5.21(4.23 to 6.45)	6.82(5.27 to 8.85)	0.31(0.12 to 0.49)
Ghana	Liver cancer	12925(9829 to 17194)	31258(22668 to 40048)	1.42(0.6 to 2.41)	161.23(122.42 to 216.86)	156.63(115.87 to 201.04)	-0.03(-0.37 to 0.38)
Ghana	Liver cancer due to alcohol use	1997(1250 to 3163)	5707(3546 to 8513)	1.86(0.78 to 3.24)	29.32(18.26 to 45.67)	32.43(20.57 to 47.67)	0.11(-0.31 to 0.62)

Ghana	Liver cancer due to hepatitis B	7028(5001 to 9665)	16912(11674 to 22776)	1.41(0.5 to 2.58)	87.57(62.6 to 122.06)	81.12(56.48 to 110.38)	-0.07(-0.43 to 0.36)
Ghana	Liver cancer due to hepatitis C	1465(898 to 2216)	3232(1929 to 4849)	1.21(0.48 to 2.14)	23.62(14.89 to 34.51)	20.29(12.26 to 30.24)	-0.14(-0.41 to 0.21)
Ghana	Liver cancer due to NASH	675(435 to 998)	2067(1379 to 3041)	2.06(1.05 to 3.39)	9.65(6.16 to 14.59)	11.51(7.69 to 17.26)	0.19(-0.2 to 0.69)
Ghana	Liver cancer due to other causes	1760(1252 to 2470)	3341(2171 to 4845)	0.9(0.19 to 1.9)	11.08(8.09 to 14.92)	11.27(7.74 to 15.58)	0.02(-0.29 to 0.42)
Greece	Liver cancer	7198(6824 to 7639)	14337(13193 to 15515)	0.99(0.82 to 1.18)	48.63(46.13 to 51.46)	70.32(65.11 to 75.99)	0.45(0.32 to 0.58)
Greece	Liver cancer due to alcohol use	2395(1763 to 3026)	4903(3651 to 6326)	1.05(0.81 to 1.33)	15.51(11.51 to 19.55)	23.12(16.64 to 30)	0.49(0.32 to 0.68)
Greece	Liver cancer due to hepatitis B	2748(2114 to 3428)	5307(3972 to 6906)	0.93(0.7 to 1.18)	18.84(14.67 to 23.25)	28.39(21.97 to 35.89)	0.51(0.33 to 0.7)
Greece	Liver cancer due to hepatitis C	1169(795 to 1575)	2425(1671 to 3367)	1.07(0.83 to 1.38)	7.58(5.22 to 10.11)	10.11(6.93 to 14.17)	0.33(0.18 to 0.5)
Greece	Liver cancer due to NASH	442(302 to 644)	1003(691 to 1445)	1.27(0.93 to 1.64)	2.87(2 to 4.12)	4.32(3 to 6.16)	0.5(0.29 to 0.76)
Greece	Liver cancer due to other causes	444(337 to 587)	699(474 to 976)	0.58(0.32 to 0.83)	3.83(3.1 to 4.78)	4.38(3.32 to 5.69)	0.14(-0.02 to 0.31)
Greenland	Liver cancer	54(45 to 64)	118(90 to 153)	1.2(0.6 to 2.01)	125.83(108.08 to 147.12)	157.81(122.13 to 201.59)	0.25(-0.07 to 0.7)
Greenland	Liver cancer due to alcohol use	22(16 to 29)	52(34 to 73)	1.34(0.64 to 2.31)	51.85(38.14 to 67.5)	67.45(45.49 to 93.11)	0.3(-0.06 to 0.77)
Greenland	Liver cancer due to hepatitis B	10(6 to 15)	17(10 to 26)	0.74(0.19 to 1.5)	19.62(13.12 to 29.3)	22.11(13.83 to	0.13(-0.19 to 0.57)

						34.27)	
Greenland	Liver cancer due to hepatitis C	11(7 to 16)	29(18 to 43)	1.54(0.83 to 2.42)	30.18(20.1 to 42.38)	38.62(24.74 to 56.22)	0.28(-0.04 to 0.68)
Greenland	Liver cancer due to NASH	4(3 to 6)	11(7 to 16)	1.66(0.92 to 2.73)	10.23(6.99 to 14.96)	14.79(9.67 to 21.77)	0.45(0.07 to 0.96)
Greenland	Liver cancer due to other causes	7(5 to 9)	10(6 to 16)	0.56(0.05 to 1.23)	13.95(10.36 to 18.28)	14.85(9.56 to 22.41)	0.06(-0.25 to 0.46)
Grenada	Liver cancer	160(143 to 178)	85(74 to 97)	-0.47(-0.55 to -0.38)	234.73(208.46 to 260.94)	74.02(64.82 to 84.47)	-0.68(-0.73 to -0.63)
Grenada	Liver cancer due to alcohol use	51(37 to 66)	32(23 to 41)	-0.38(-0.48 to -0.24)	75.74(55.09 to 99.26)	27.33(19.91 to 35.47)	-0.64(-0.7 to -0.57)
Grenada	Liver cancer due to hepatitis B	50(38 to 65)	25(18 to 34)	-0.5(-0.59 to -0.4)	76.56(57.79 to 99.84)	20.89(14.95 to 28.46)	-0.73(-0.77 to -0.67)
Grenada	Liver cancer due to hepatitis C	34(23 to 45)	16(10 to 22)	-0.54(-0.62 to -0.44)	47.16(32.17 to 64.27)	13.95(9.41 to 19.36)	-0.7(-0.75 to -0.65)
Grenada	Liver cancer due to NASH	11(8 to 15)	7(5 to 9)	-0.4(-0.51 to -0.27)	15.84(11.13 to 21.99)	5.99(4.21 to 8.33)	-0.62(-0.69 to -0.54)
Grenada	Liver cancer due to other causes	15(11 to 18)	6(5 to 8)	-0.58(-0.66 to -0.49)	19.44(14.8 to 24.95)	5.86(4.44 to 7.54)	-0.7(-0.75 to -0.64)
Guam	Liver cancer	92(77 to 110)	325(259 to 406)	2.53(1.69 to 3.66)	99.69(84.6 to 117.98)	169.45(136.43 to 209.91)	0.7(0.31 to 1.21)
Guam	Liver cancer due to alcohol use	10(6 to 14)	38(23 to 59)	2.95(1.96 to 4.37)	11.29(7.11 to 16.59)	19.61(12 to 29.73)	0.74(0.3 to 1.34)
Guam	Liver cancer due to hepatitis B	57(45 to 72)	205(156 to 262)	2.59(1.68 to 3.91)	57.49(45.28 to 72.8)	107.08(82.86 to	0.86(0.42 to 1.51)

						136.1)	
Guam	Liver cancer due to hepatitis C	13(8 to 19)	41(25 to 62)	2.2(1.43 to 3.14)	17.57(11.46 to 24.86)	21.31(13.42 to 31.68)	0.21(-0.07 to 0.56)
Guam	Liver cancer due to NASH	7(5 to 10)	28(18 to 41)	3.09(2.01 to 4.46)	8.21(5.69 to 11.82)	14.54(9.54 to 21.07)	0.77(0.3 to 1.32)
Guam	Liver cancer due to other causes	6(4 to 7)	13(9 to 18)	1.23(0.69 to 1.94)	5.14(3.8 to 6.74)	6.92(4.84 to 9.51)	0.35(0.04 to 0.74)
Guatemala	Liver cancer	10262(9053 to 11521)	13470(10540 to 17240)	0.31(0.01 to 0.73)	240.23(212.63 to 268.67)	112.63(88.33 to 143.52)	-0.53(-0.64 to -0.38)
Guatemala	Liver cancer due to alcohol use	2259(1557 to 3069)	3196(2072 to 4710)	0.42(0.07 to 0.91)	57.24(39.47 to 76.97)	28.02(18.01 to 41.52)	-0.51(-0.63 to -0.34)
Guatemala	Liver cancer due to hepatitis B	2508(1873 to 3304)	2837(1935 to 4065)	0.13(-0.14 to 0.51)	52.78(38.77 to 70.81)	21.79(14.42 to 31.99)	-0.59(-0.68 to -0.45)
Guatemala	Liver cancer due to hepatitis C	3361(2478 to 4293)	4683(3287 to 6520)	0.39(0.07 to 0.85)	92.15(69.6 to 116.04)	42.78(30.09 to 59.09)	-0.54(-0.64 to -0.4)
Guatemala	Liver cancer due to NASH	665(481 to 933)	1140(746 to 1636)	0.72(0.28 to 1.32)	16.09(11.33 to 23)	9.49(6.13 to 13.93)	-0.41(-0.56 to -0.21)
Guatemala	Liver cancer due to other causes	1469(1170 to 1819)	1613(1172 to 2171)	0.1(-0.19 to 0.5)	21.97(16.91 to 28.39)	10.56(7.5 to 14.47)	-0.52(-0.64 to -0.36)
Guinea	Liver cancer	32551(26830 to 39187)	59804(41827 to 78297)	0.84(0.25 to 1.54)	833.45(689.56 to 1014.67)	889.94(611.41 to 1163.89)	0.07(-0.3 to 0.49)
Guinea	Liver cancer due to alcohol use	4310(2774 to 6306)	8581(4926 to 13187)	0.99(0.25 to 1.85)	124.09(80.46 to 182)	147.03(84.72 to 224.75)	0.18(-0.27 to 0.7)
Guinea	Liver cancer due to hepatitis B	17500(13402 to 22461)	33423(22132 to 46228)	0.91(0.23 to 1.75)	473.11(358.14 to 609.32)	497.66(327.71 to	0.05(-0.32 to 0.51)

						691.59)	
Guinea	Liver cancer due to hepatitis C	4067(2667 to 5831)	6723(3943 to 9987)	0.65(0.15 to 1.27)	123.21(81.36 to 174.15)	122.86(72.99 to 181.41)	0(-0.31 to 0.37)
Guinea	Liver cancer due to NASH	1788(1209 to 2580)	3752(2388 to 5614)	1.1(0.46 to 1.91)	51.49(34.8 to 74.92)	62.3(39.29 to 94.66)	0.21(-0.15 to 0.68)
Guinea	Liver cancer due to other causes	4885(3191 to 7378)	7325(4514 to 11511)	0.5(-0.15 to 1.59)	61.56(44.56 to 82.58)	60.09(40.08 to 86.89)	-0.02(-0.36 to 0.46)
Guinea-Bissau	Liver cancer	1137(801 to 1660)	1533(1083 to 2152)	0.35(-0.06 to 0.92)	223.51(158.32 to 321.81)	164.47(116.77 to 225.64)	-0.26(-0.49 to 0.05)
Guinea-Bissau	Liver cancer due to alcohol use	156(92 to 260)	221(130 to 350)	0.41(-0.06 to 1.11)	35.72(20.93 to 58.55)	27.68(16.59 to 43.13)	-0.23(-0.48 to 0.14)
Guinea-Bissau	Liver cancer due to hepatitis B	623(415 to 940)	838(567 to 1210)	0.35(-0.09 to 0.96)	123.18(82.48 to 182.04)	84.12(56.94 to 121.57)	-0.32(-0.54 to 0)
Guinea-Bissau	Liver cancer due to hepatitis C	143(82 to 235)	199(112 to 317)	0.39(-0.01 to 0.95)	34.95(20.32 to 56.03)	27.82(16.39 to 43.68)	-0.2(-0.42 to 0.11)
Guinea-Bissau	Liver cancer due to NASH	66(40 to 107)	114(70 to 183)	0.73(0.21 to 1.46)	14.6(9.03 to 23.1)	13.77(8.33 to 21.52)	-0.06(-0.34 to 0.32)
Guinea-Bissau	Liver cancer due to other causes	149(93 to 234)	160(111 to 229)	0.07(-0.31 to 0.68)	15.06(9.66 to 22.86)	11.09(7.26 to 16.71)	-0.26(-0.49 to 0.07)
Guyana	Liver cancer	774(664 to 884)	543(411 to 702)	-0.3(-0.49 to -0.06)	175.46(150.7 to 200.9)	79.96(61.06 to 103)	-0.54(-0.66 to -0.39)
Guyana	Liver cancer due to alcohol use	241(169 to 321)	175(115 to 250)	-0.27(-0.48 to -0.02)	59.18(41.6 to 77.83)	26.39(17.8 to 37.17)	-0.55(-0.67 to -0.4)
Guyana	Liver cancer due to hepatitis B	268(205 to 354)	177(118 to 257)	-0.34(-0.54 to -0.09)	55.82(41.38 to 75.25)	24.54(16.41 to	-0.56(-0.68 to -0.4)

						35.36)	
Guyana	Liver cancer due to hepatitis C	127(84 to 183)	95(58 to 142)	-0.25(-0.45 to -0.01)	33.71(22.61 to 48.41)	15.17(9.46 to 22.28)	-0.55(-0.66 to -0.41)
Guyana	Liver cancer due to NASH	50(35 to 71)	45(29 to 67)	-0.11(-0.36 to 0.21)	11.95(8.22 to 17)	6.83(4.47 to 10.23)	-0.43(-0.58 to -0.22)
Guyana	Liver cancer due to other causes	87(67 to 109)	50(35 to 71)	-0.42(-0.59 to -0.22)	14.8(10.86 to 19.5)	7.03(4.93 to 9.79)	-0.52(-0.66 to -0.36)
Haiti	Liver cancer	6561(4055 to 8907)	8490(5110 to 12665)	0.29(-0.09 to 0.84)	175.19(109.15 to 239.62)	107.22(64.8 to 160.1)	-0.39(-0.56 to -0.15)
Haiti	Liver cancer due to alcohol use	1825(1054 to 2847)	2454(1335 to 4199)	0.34(-0.08 to 0.94)	53.06(30.86 to 83.88)	33.64(18.04 to 57.78)	-0.37(-0.56 to -0.1)
Haiti	Liver cancer due to hepatitis B	2159(1262 to 3254)	2903(1634 to 4827)	0.34(-0.09 to 0.93)	55.6(33.08 to 86.03)	34.08(18.9 to 56.62)	-0.39(-0.58 to -0.13)
Haiti	Liver cancer due to hepatitis C	1219(649 to 2016)	1507(793 to 2465)	0.24(-0.12 to 0.74)	38.4(21.18 to 62.68)	22.11(11.88 to 35.89)	-0.42(-0.59 to -0.2)
Haiti	Liver cancer due to NASH	370(200 to 589)	514(265 to 859)	0.39(-0.04 to 1.01)	10.81(5.89 to 17.2)	6.95(3.54 to 11.52)	-0.36(-0.55 to -0.09)
Haiti	Liver cancer due to other causes	988(533 to 1893)	1112(675 to 1780)	0.13(-0.27 to 0.67)	17.32(9.63 to 29.07)	10.44(6.11 to 16.45)	-0.4(-0.58 to -0.15)
Honduras	Liver cancer	6584(2829 to 9336)	22759(10486 to 34296)	2.46(1.59 to 3.8)	283.36(112.98 to 410)	359.62(166.22 to 538.16)	0.27(-0.04 to 0.82)
Honduras	Liver cancer due to alcohol use	1592(527 to 2621)	6393(2623 to 10721)	3.02(1.96 to 4.91)	74.22(23.8 to 122.73)	102.68(42.11 to 170.33)	0.38(0.03 to 1.07)
Honduras	Liver cancer due to hepatitis B	1653(656 to 2706)	4659(1907 to 8077)	1.82(0.99 to 3.06)	65.35(23.62 to 107.96)	68.38(27.53 to	0.05(-0.26 to 0.51)

						117.79)	
Honduras	Liver cancer due to hepatitis C	2115(737 to 3383)	8085(3260 to 13327)	2.82(1.88 to 4.59)	103.3(35.02 to 165.42)	135.41(54.48 to 221.81)	0.31(0 to 0.93)
Honduras	Liver cancer due to NASH	409(151 to 686)	1824(706 to 3164)	3.46(2.24 to 5.44)	17.96(6.13 to 31.39)	29.09(11.24 to 50.45)	0.62(0.18 to 1.36)
Honduras	Liver cancer due to other causes	816(456 to 1142)	1798(827 to 2936)	1.2(0.49 to 2.13)	22.52(10.6 to 34.79)	24.06(10.41 to 39.84)	0.07(-0.23 to 0.49)
Hungary	Liver cancer	23517(22482 to 24521)	10732(8725 to 13195)	-0.54(-0.63 to -0.43)	163.51(156.48 to 170.69)	61.26(49.57 to 75.43)	-0.63(-0.7 to -0.53)
Hungary	Liver cancer due to alcohol use	10419(8372 to 12449)	4647(3332 to 6246)	-0.55(-0.65 to -0.44)	70.69(56.61 to 84.13)	25.54(18.15 to 34.35)	-0.64(-0.71 to -0.55)
Hungary	Liver cancer due to hepatitis B	5791(4233 to 7827)	2540(1718 to 3677)	-0.56(-0.65 to -0.45)	41.81(31.3 to 55.7)	15.78(10.95 to 22.37)	-0.62(-0.71 to -0.52)
Hungary	Liver cancer due to hepatitis C	4897(3405 to 6615)	2354(1499 to 3393)	-0.52(-0.61 to -0.4)	32.96(23.08 to 44.24)	12.37(7.92 to 17.96)	-0.62(-0.7 to -0.54)
Hungary	Liver cancer due to NASH	1515(1070 to 2125)	823(558 to 1203)	-0.46(-0.58 to -0.3)	10.44(7.53 to 14.44)	4.48(3.03 to 6.55)	-0.57(-0.67 to -0.44)
Hungary	Liver cancer due to other causes	894(666 to 1160)	368(251 to 515)	-0.59(-0.68 to -0.48)	7.61(5.94 to 9.57)	3.1(2.27 to 4.06)	-0.59(-0.68 to -0.49)
Iceland	Liver cancer	121(112 to 132)	328(290 to 369)	1.7(1.33 to 2.13)	44.57(40.86 to 48.21)	64.25(56.89 to 72.22)	0.44(0.24 to 0.67)
Iceland	Liver cancer due to alcohol use	39(29 to 51)	123(90 to 159)	2.17(1.68 to 2.77)	14.13(10.33 to 18.38)	23.73(17.57 to 30.71)	0.68(0.42 to 1.01)
Iceland	Liver cancer due to hepatitis B	22(16 to 31)	56(39 to 78)	1.48(1.11 to 1.92)	8.52(6.12 to 11.95)	11.69(8.27 to	0.37(0.16 to 0.61)

						16.23)	
Iceland	Liver cancer due to hepatitis C	43(32 to 54)	109(78 to 146)	1.56(1.19 to 1.99)	15.25(11.44 to 19.56)	20.24(14.4 to 27.11)	0.33(0.13 to 0.55)
Iceland	Liver cancer due to NASH	8(5 to 11)	22(15 to 32)	1.9(1.4 to 2.55)	2.69(1.87 to 3.82)	4.16(2.88 to 6.07)	0.55(0.28 to 0.89)
Iceland	Liver cancer due to other causes	10(8 to 13)	18(13 to 25)	0.84(0.49 to 1.23)	3.98(3.19 to 4.95)	4.43(3.18 to 5.85)	0.11(-0.1 to 0.35)
India	Liver cancer	375337(321165 to 428499)	843856(712239 to 1002252)	1.25(0.85 to 1.71)	68.55(57.3 to 79.21)	69.24(58.62 to 82.07)	0.01(-0.18 to 0.22)
India	Liver cancer due to alcohol use	79027(60430 to 99203)	217814(170504 to 270698)	1.76(1.18 to 2.48)	16.24(12.31 to 20.41)	18.18(14.28 to 22.58)	0.12(-0.12 to 0.41)
India	Liver cancer due to hepatitis B	161909(131846 to 191508)	330206(268857 to 402951)	1.04(0.64 to 1.5)	28.59(23 to 34.17)	25.99(21.11 to 31.87)	-0.09(-0.27 to 0.11)
India	Liver cancer due to hepatitis C	50299(39315 to 62768)	140423(109064 to 179042)	1.79(1.22 to 2.38)	11.48(9.07 to 14.24)	12.38(9.62 to 15.67)	0.08(-0.15 to 0.3)
India	Liver cancer due to NASH	31343(25246 to 38281)	86138(68322 to 107158)	1.75(1.24 to 2.35)	6.48(5.17 to 7.95)	7.29(5.78 to 9)	0.12(-0.09 to 0.36)
India	Liver cancer due to other causes	52759(43774 to 62653)	69275(56257 to 83750)	0.31(0.03 to 0.63)	5.76(4.89 to 6.72)	5.4(4.39 to 6.55)	-0.06(-0.24 to 0.14)
Indonesia	Liver cancer	85228(71754 to 99970)	126128(105690 to 145908)	0.48(0.15 to 0.91)	68.22(58.33 to 77.23)	56.62(47.98 to 64.79)	-0.17(-0.34 to 0.05)
Indonesia	Liver cancer due to alcohol use	11512(9127 to 14216)	22203(16865 to 28019)	0.93(0.48 to 1.48)	10.72(8.47 to 13.43)	9.78(7.53 to 12.26)	-0.09(-0.3 to 0.16)
Indonesia	Liver cancer due to hepatitis B	21475(17674 to 25481)	30952(23856 to 38562)	0.44(0.1 to 0.88)	15.75(13.01 to 18.87)	12.29(9.55 to 15.27)	-0.22(-0.39 to 0)

Indonesia	Liver cancer due to hepatitis C	26458(22133 to 31825)	47858(39063 to 56927)	0.81(0.41 to 1.29)	26.89(22.54 to 32.36)	23.15(19.11 to 27.1)	-0.14(-0.32 to 0.09)
Indonesia	Liver cancer due to NASH	6493(5209 to 7836)	11773(9384 to 14473)	0.81(0.42 to 1.3)	5.55(4.45 to 6.77)	5.31(4.3 to 6.41)	-0.04(-0.24 to 0.2)
Indonesia	Liver cancer due to other causes	19290(13576 to 29824)	13343(10855 to 16147)	-0.31(-0.59 to 0.05)	9.32(6.74 to 13.89)	6.08(4.93 to 7.42)	-0.35(-0.6 to -0.03)
Iran (Islamic Republic of)	Liver cancer	30563(25874 to 35118)	61489(56204 to 67247)	1.01(0.66 to 1.51)	102.74(84.91 to 120.06)	81.29(74.16 to 88.83)	-0.21(-0.36 to 0)
Iran (Islamic Republic of)	Liver cancer due to alcohol use	2916(2104 to 3924)	6182(4617 to 8122)	1.12(0.75 to 1.68)	10.77(7.81 to 14.16)	8.51(6.36 to 11.17)	-0.21(-0.35 to 0)
Iran (Islamic Republic of)	Liver cancer due to hepatitis B	13750(11543 to 16192)	27500(23711 to 31557)	1(0.67 to 1.45)	44.15(36.72 to 51.62)	34.13(29.3 to 39.32)	-0.23(-0.36 to -0.04)
Iran (Islamic Republic of)	Liver cancer due to hepatitis C	6141(4760 to 8011)	14287(12024 to 16932)	1.33(0.74 to 2.11)	25.82(19.9 to 33.62)	20.4(17.12 to 23.9)	-0.21(-0.42 to 0.07)
Iran (Islamic Republic of)	Liver cancer due to NASH	2968(2309 to 3704)	7755(6523 to 9082)	1.61(0.99 to 2.41)	11.39(8.68 to 14.57)	10.73(9.04 to 12.62)	-0.06(-0.3 to 0.26)
Iran (Islamic Republic of)	Liver cancer due to other causes	4787(3878 to 5989)	5764(5019 to 6596)	0.2(-0.03 to 0.54)	10.62(8.67 to 12.78)	7.52(6.58 to 8.61)	-0.29(-0.41 to -0.14)
Iraq	Liver cancer	10455(8058 to 13089)	37464(27898 to 48530)	2.58(1.49 to 4.07)	115.26(88.65 to 144.67)	144.76(109.52 to 184.24)	0.26(-0.13 to 0.79)
Iraq	Liver cancer due to alcohol use	823(483 to 1269)	3292(1960 to 5073)	3(1.71 to 4.78)	10.27(6.06 to 15.8)	13.73(8.28 to 20.97)	0.34(-0.09 to 0.91)
Iraq	Liver cancer due to hepatitis B	4275(3004 to 5788)	15409(10463 to 22010)	2.6(1.4 to 4.34)	46.36(32.19 to 64.12)	54.65(36.99 to 78.03)	0.18(-0.2 to 0.73)
Iraq	Liver cancer due to hepatitis C	2946(1970 to 4153)	11410(7499 to 16381)	2.87(1.69 to 4.64)	38.01(25.69 to 53.03)	49.73(33.18 to	0.31(-0.08 to 0.88)

						70.11)	
Iraq	Liver cancer due to NASH	849(569 to 1248)	3767(2383 to 5744)	3.43(2 to 5.5)	10.16(6.72 to 15.05)	15.29(9.67 to 22.71)	0.5(0.04 to 1.18)
Iraq	Liver cancer due to other causes	1561(1164 to 2106)	3586(2555 to 5004)	1.3(0.57 to 2.31)	10.46(7.43 to 14.2)	11.35(7.82 to 16.32)	0.08(-0.23 to 0.51)
Ireland	Liver cancer	1335(1248 to 1428)	5201(4638 to 5747)	2.89(2.4 to 3.41)	33.42(31.26 to 35.67)	72.42(64.47 to 80.2)	1.17(0.89 to 1.45)
Ireland	Liver cancer due to alcohol use	476(356 to 603)	1991(1484 to 2521)	3.18(2.53 to 3.88)	11.7(8.71 to 14.71)	27.31(20.27 to 34.6)	1.33(0.96 to 1.72)
Ireland	Liver cancer due to hepatitis B	205(144 to 285)	737(505 to 1069)	2.59(2.06 to 3.17)	5.38(3.86 to 7.47)	10.87(7.58 to 15.49)	1.02(0.72 to 1.35)
Ireland	Liver cancer due to hepatitis C	459(343 to 583)	1822(1335 to 2363)	2.97(2.43 to 3.57)	11.24(8.43 to 14.18)	24.53(17.95 to 31.62)	1.18(0.9 to 1.51)
Ireland	Liver cancer due to NASH	78(54 to 114)	356(247 to 517)	3.55(2.82 to 4.38)	1.91(1.35 to 2.71)	4.87(3.4 to 6.92)	1.55(1.14 to 2)
Ireland	Liver cancer due to other causes	117(94 to 144)	294(214 to 392)	1.51(1.1 to 1.95)	3.19(2.59 to 3.87)	4.85(3.7 to 6.25)	0.52(0.29 to 0.76)
Israel	Liver cancer	2827(2545 to 3117)	6789(6181 to 7431)	1.4(1.12 to 1.74)	58.37(52.61 to 64.63)	62.02(56.39 to 67.85)	0.06(-0.06 to 0.21)
Israel	Liver cancer due to alcohol use	662(457 to 902)	1829(1279 to 2436)	1.76(1.34 to 2.28)	13.6(9.4 to 18.48)	16.58(11.6 to 22.08)	0.22(0.03 to 0.46)
Israel	Liver cancer due to hepatitis B	524(370 to 743)	1164(815 to 1654)	1.22(0.92 to 1.61)	11.18(7.87 to 15.68)	11.27(7.92 to 16.02)	0.01(-0.14 to 0.19)
Israel	Liver cancer due to hepatitis C	1190(891 to 1504)	2761(2072 to 3448)	1.32(1.02 to 1.69)	24.46(18.26 to 30.98)	24.46(18.36 to 30.74)	0(-0.12 to 0.16)

Israel	Liver cancer due to NASH	199(134 to 288)	517(354 to 753)	1.6(1.16 to 2.08)	4.02(2.75 to 5.83)	4.64(3.21 to 6.68)	0.15(-0.03 to 0.37)
Israel	Liver cancer due to other causes	253(196 to 326)	518(391 to 685)	1.05(0.79 to 1.38)	5.11(3.93 to 6.64)	5.07(3.89 to 6.57)	-0.01(-0.13 to 0.15)
Italy	Liver cancer	129610(126360 to 132338)	131543(119652 to 141949)	0.01(-0.07 to 0.09)	151.35(147.5 to 154.49)	106.25(97.08 to 114.48)	-0.3(-0.35 to -0.25)
Italy	Liver cancer due to alcohol use	37029(31930 to 42240)	35249(29559 to 40990)	-0.05(-0.15 to 0.05)	42.66(36.74 to 48.58)	28.8(24.01 to 33.58)	-0.33(-0.39 to -0.26)
Italy	Liver cancer due to hepatitis B	18281(15432 to 21749)	15465(12765 to 18745)	-0.15(-0.24 to -0.07)	22.67(19.28 to 26.72)	14.44(12 to 17.28)	-0.36(-0.42 to -0.3)
Italy	Liver cancer due to hepatitis C	64501(58354 to 70834)	71136(62707 to 78795)	0.1(0.02 to 0.18)	72.91(66.05 to 79.9)	53.9(47.26 to 60.26)	-0.26(-0.31 to -0.21)
Italy	Liver cancer due to NASH	5557(4665 to 6560)	5929(4885 to 7115)	0.07(-0.03 to 0.16)	6.34(5.36 to 7.45)	4.55(3.77 to 5.42)	-0.28(-0.34 to -0.23)
Italy	Liver cancer due to other causes	4241(3676 to 4862)	3764(3180 to 4425)	-0.11(-0.19 to -0.04)	6.77(6 to 7.61)	4.56(4.01 to 5.14)	-0.33(-0.38 to -0.28)
Jamaica	Liver cancer	1793(1683 to 1914)	1943(1518 to 2455)	0.08(-0.18 to 0.4)	100.75(94.34 to 108.05)	66.04(51.55 to 83.74)	-0.34(-0.5 to -0.15)
Jamaica	Liver cancer due to alcohol use	539(399 to 694)	618(418 to 866)	0.15(-0.13 to 0.48)	30.92(22.77 to 39.99)	21.04(14.21 to 29.53)	-0.32(-0.49 to -0.13)
Jamaica	Liver cancer due to hepatitis B	586(450 to 750)	605(416 to 843)	0.03(-0.24 to 0.37)	33.74(25.73 to 43.58)	20.28(13.85 to 28.32)	-0.4(-0.55 to -0.2)
Jamaica	Liver cancer due to hepatitis C	354(245 to 474)	377(240 to 559)	0.06(-0.2 to 0.38)	20(13.74 to 26.99)	12.71(8.12 to 18.9)	-0.36(-0.52 to -0.17)

Jamaica	Liver cancer due to NASH	129(92 to 181)	178(118 to 259)	0.38(0 to 0.82)	7.18(5.07 to 10.16)	6(3.97 to 8.74)	-0.16(-0.39 to 0.11)
Jamaica	Liver cancer due to other causes	185(147 to 230)	166(116 to 230)	-0.1(-0.33 to 0.18)	8.9(6.86 to 11.35)	6(4.26 to 8.22)	-0.33(-0.49 to -0.12)
Japan	Liver cancer	504223(490496 to 514912)	557065(499746 to 597191)	0.1(0.01 to 0.17)	292.18(284.23 to 298.37)	175.22(160.86 to 186.6)	-0.4(-0.44 to -0.37)
Japan	Liver cancer due to alcohol use	67761(57798 to 79457)	64895(54264 to 76150)	-0.04(-0.15 to 0.08)	38.93(33.26 to 45.59)	21.5(17.81 to 25.43)	-0.45(-0.49 to -0.4)
Japan	Liver cancer due to hepatitis B	88514(77191 to 101665)	67539(57254 to 80625)	-0.24(-0.32 to -0.15)	52.24(45.78 to 59.66)	26.52(22.68 to 31.08)	-0.49(-0.53 to -0.45)
Japan	Liver cancer due to hepatitis C	311963(292281 to 330173)	383625(337521 to 418060)	0.23(0.1 to 0.32)	178.83(167.59 to 189.22)	112.76(101.23 to 122.52)	-0.37(-0.41 to -0.33)
Japan	Liver cancer due to NASH	18941(16033 to 22158)	25075(20001 to 30390)	0.32(0.13 to 0.52)	10.96(9.31 to 12.8)	7.33(6.01 to 8.79)	-0.33(-0.39 to -0.27)
Japan	Liver cancer due to other causes	17045(14701 to 19522)	15930(13295 to 18794)	-0.07(-0.19 to 0.06)	11.21(9.79 to 12.66)	7.11(6.24 to 8)	-0.37(-0.42 to -0.32)
Jordan	Liver cancer	1186(965 to 1451)	3751(3002 to 4726)	2.16(1.33 to 3.42)	74.14(59.01 to 91.82)	52.36(42.17 to 65.27)	-0.29(-0.49 to 0)
Jordan	Liver cancer due to alcohol use	97(58 to 147)	384(221 to 609)	2.95(1.64 to 4.91)	7.06(4.25 to 10.72)	5.76(3.4 to 9.11)	-0.18(-0.46 to 0.2)
Jordan	Liver cancer due to hepatitis B	513(386 to 677)	1542(1079 to 2111)	2.01(1.1 to 3.32)	30.04(21.94 to 40.82)	19.57(13.71 to 27.19)	-0.35(-0.54 to -0.05)
Jordan	Liver cancer due to hepatitis C	295(193 to 415)	1004(665 to 1384)	2.4(1.38 to 3.82)	23.46(15.4 to 32.56)	16.3(11 to 22.39)	-0.31(-0.51 to -0.01)
Jordan	Liver cancer due to NASH	100(67 to 147)	423(281 to 625)	3.24(2.05 to 5.1)	7.11(4.62 to 10.73)	6.41(4.21 to 9.48)	-0.1(-0.36 to 0.29)

Jordan	Liver cancer due to other causes	181(139 to 232)	399(299 to 518)	1.2(0.64 to 1.99)	6.46(4.74 to 8.67)	4.33(3.13 to 5.87)	-0.33(-0.5 to -0.1)
Kazakhstan	Liver cancer	12960(11965 to 13953)	29811(25700 to 34545)	1.3(0.95 to 1.71)	95.14(88.27 to 102.14)	162.18(140.05 to 187.56)	0.7(0.45 to 1)
Kazakhstan	Liver cancer due to alcohol use	3785(2721 to 4911)	9423(6561 to 12487)	1.49(1.08 to 1.98)	28.47(20.56 to 36.58)	51.07(35.99 to 67.2)	0.79(0.49 to 1.15)
Kazakhstan	Liver cancer due to hepatitis B	3676(2753 to 4862)	7982(5590 to 11098)	1.17(0.77 to 1.59)	26.12(19.36 to 34.67)	41.35(29.17 to 57.76)	0.58(0.31 to 0.88)
Kazakhstan	Liver cancer due to hepatitis C	3548(2553 to 4654)	8778(6009 to 11957)	1.47(1.08 to 1.92)	27.66(20.11 to 35.83)	49.61(34.5 to 67.45)	0.79(0.51 to 1.1)
Kazakhstan	Liver cancer due to NASH	669(467 to 946)	2107(1429 to 3052)	2.15(1.56 to 2.82)	5.11(3.55 to 7.28)	11.88(8.11 to 16.89)	1.32(0.92 to 1.82)
Kazakhstan	Liver cancer due to other causes	1282(851 to 1697)	1520(1053 to 2067)	0.19(-0.27 to 1)	7.78(5.24 to 10.19)	8.27(5.71 to 11.25)	0.06(-0.32 to 0.74)
Kenya	Liver cancer	7063(5151 to 12285)	22017(14800 to 32461)	2.12(1.4 to 3.06)	66.24(45.58 to 117.89)	81.93(55.39 to 119.77)	0.24(-0.03 to 0.57)
Kenya	Liver cancer due to alcohol use	1476(892 to 2738)	5007(3041 to 7895)	2.39(1.59 to 3.34)	16.79(10.11 to 31.13)	20.62(12.67 to 32.22)	0.23(-0.06 to 0.56)
Kenya	Liver cancer due to hepatitis B	2121(1333 to 3806)	7277(4633 to 10997)	2.43(1.6 to 3.42)	19.71(12.33 to 35.4)	24.05(15.23 to 36.11)	0.22(-0.07 to 0.55)
Kenya	Liver cancer due to hepatitis C	1094(738 to 1906)	3698(2410 to 5547)	2.38(1.63 to 3.31)	13.44(9.13 to 23.35)	17.02(11.3 to 25.19)	0.27(0.01 to 0.59)
Kenya	Liver cancer due to NASH	795(533 to 1398)	2964(1949 to 4487)	2.73(1.88 to 3.78)	8.52(5.78 to 14.77)	11.85(7.94 to 17.7)	0.39(0.09 to 0.75)

Kenya	Liver cancer due to other causes	1577(1181 to 2227)	3072(2191 to 4349)	0.95(0.32 to 1.75)	7.78(5.98 to 12.52)	8.39(5.74 to 12.18)	0.08(-0.19 to 0.43)
Kiribati	Liver cancer	197(151 to 249)	315(240 to 402)	0.6(0.11 to 1.22)	423.71(325.23 to 531.34)	353.67(273.08 to 446.53)	-0.17(-0.41 to 0.15)
Kiribati	Liver cancer due to alcohol use	17(10 to 27)	28(16 to 44)	0.65(0.1 to 1.4)	39.56(23.47 to 62.39)	33.54(19.69 to 50.57)	-0.15(-0.43 to 0.24)
Kiribati	Liver cancer due to hepatitis B	124(91 to 163)	193(139 to 260)	0.56(0.06 to 1.21)	253.91(184.65 to 336.14)	202.8(146.53 to 273.21)	-0.2(-0.46 to 0.13)
Kiribati	Liver cancer due to hepatitis C	30(19 to 44)	47(29 to 70)	0.6(0.12 to 1.29)	77.16(50.7 to 111.92)	65.89(41.98 to 95.04)	-0.15(-0.39 to 0.19)
Kiribati	Liver cancer due to NASH	13(9 to 19)	25(16 to 37)	0.9(0.32 to 1.67)	29.85(19.61 to 43.79)	30.31(20.29 to 44.55)	0.02(-0.28 to 0.42)
Kiribati	Liver cancer due to other causes	13(10 to 18)	21(15 to 30)	0.6(0.13 to 1.18)	23.22(16.22 to 31.97)	21.14(14.51 to 30.19)	-0.09(-0.34 to 0.25)
Kuwait	Liver cancer	554(495 to 620)	1447(1108 to 1841)	1.61(1 to 2.34)	68.4(60.73 to 76.79)	51.33(39.8 to 65.2)	-0.25(-0.42 to -0.04)
Kuwait	Liver cancer due to alcohol use	45(29 to 67)	139(81 to 217)	2.06(1.24 to 3.15)	6.81(4.33 to 10.05)	5.47(3.26 to 8.58)	-0.2(-0.41 to 0.09)
Kuwait	Liver cancer due to hepatitis B	258(206 to 317)	613(419 to 859)	1.37(0.75 to 2.13)	28.49(21.95 to 36.76)	18.33(12.17 to 26.25)	-0.36(-0.52 to -0.16)
Kuwait	Liver cancer due to hepatitis C	122(83 to 164)	392(262 to 554)	2.22(1.43 to 3.17)	20.19(14.13 to 26.46)	16.55(11.2 to 22.95)	-0.18(-0.38 to 0.06)
Kuwait	Liver cancer due to NASH	47(33 to 66)	178(116 to 264)	2.77(1.8 to 3.96)	6.81(4.58 to 9.8)	6.99(4.51 to 10.43)	0.03(-0.24 to 0.36)

Kuwait	Liver cancer due to other causes	81(66 to 99)	125(92 to 167)	0.54(0.18 to 0.97)	6.11(4.83 to 7.63)	3.99(2.96 to 5.41)	-0.35(-0.48 to -0.18)
Kyrgyzstan	Liver cancer	1173(1016 to 1323)	3184(2713 to 3686)	1.72(1.2 to 2.35)	34.1(30.62 to 37.51)	64.24(54.85 to 74.39)	0.88(0.56 to 1.23)
Kyrgyzstan	Liver cancer due to alcohol use	251(176 to 335)	915(631 to 1228)	2.65(1.92 to 3.61)	8.16(5.73 to 10.78)	19.07(13.36 to 25.47)	1.34(0.88 to 1.9)
Kyrgyzstan	Liver cancer due to hepatitis B	273(202 to 362)	854(611 to 1170)	2.13(1.48 to 2.92)	8.36(6.08 to 11.07)	15.67(11.11 to 21.7)	0.87(0.49 to 1.32)
Kyrgyzstan	Liver cancer due to hepatitis C	324(234 to 419)	953(663 to 1257)	1.95(1.38 to 2.57)	10.77(7.9 to 13.81)	21.22(15.02 to 27.69)	0.97(0.61 to 1.36)
Kyrgyzstan	Liver cancer due to NASH	51(36 to 74)	180(126 to 261)	2.52(1.74 to 3.4)	1.66(1.15 to 2.42)	3.84(2.65 to 5.6)	1.31(0.85 to 1.87)
Kyrgyzstan	Liver cancer due to other causes	274(144 to 396)	281(203 to 385)	0.03(-0.44 to 1.24)	5.15(2.87 to 7.23)	4.45(3.25 to 5.92)	-0.14(-0.5 to 0.73)
Lao People's Democratic Republic	Liver cancer	6825(4547 to 9695)	9133(6566 to 12046)	0.34(-0.18 to 1.16)	281.66(191.88 to 392.48)	180.51(131.94 to 234.38)	-0.36(-0.59 to 0)
Lao People's Democratic Republic	Liver cancer due to alcohol use	1230(720 to 2015)	1970(1194 to 3039)	0.6(0 to 1.51)	54.19(31.67 to 87.91)	41.34(25.48 to 62.7)	-0.24(-0.52 to 0.18)
Lao People's Democratic Republic	Liver cancer due to hepatitis B	3332(2080 to 5018)	4324(2892 to 6071)	0.3(-0.22 to 1.14)	131.78(82.24 to 196.56)	78.32(52.62 to 110.88)	-0.41(-0.64 to -0.04)
Lao People's Democratic Republic	Liver cancer due to hepatitis C	1389(760 to 2192)	1743(1069 to 2526)	0.25(-0.18 to 0.89)	65.63(37.61 to 101.68)	40.16(25.24 to 57.22)	-0.39(-0.59 to -0.09)

Lao People's Democratic Republic	Liver cancer due to NASH	411(234 to 675)	631(392 to 936)	0.53(-0.06 to 1.38)	17.98(10.4 to 29.38)	13.21(8.25 to 19.55)	-0.27(-0.53 to 0.1)
Lao People's Democratic Republic	Liver cancer due to other causes	463(257 to 879)	465(313 to 659)	0.01(-0.49 to 0.77)	12.08(6.82 to 21)	7.49(5.01 to 10.62)	-0.38(-0.65 to 0.02)
Latvia	Liver cancer	1508(1399 to 1621)	2258(1857 to 2782)	0.5(0.22 to 0.82)	44.4(40.7 to 48.04)	66.22(54.18 to 81.88)	0.49(0.21 to 0.83)
Latvia	Liver cancer due to alcohol use	524(388 to 661)	985(723 to 1300)	0.88(0.47 to 1.37)	14.69(10.95 to 18.55)	27.91(20.29 to 37.21)	0.9(0.48 to 1.42)
Latvia	Liver cancer due to hepatitis B	404(299 to 549)	477(323 to 693)	0.18(-0.08 to 0.5)	11.85(8.84 to 15.98)	15.8(10.88 to 22.54)	0.33(0.05 to 0.7)
Latvia	Liver cancer due to hepatitis C	355(247 to 479)	533(354 to 750)	0.5(0.2 to 0.85)	9.81(6.87 to 13.13)	13.75(8.93 to 19.52)	0.4(0.12 to 0.74)
Latvia	Liver cancer due to NASH	99(68 to 140)	174(115 to 262)	0.77(0.36 to 1.26)	2.79(1.95 to 3.93)	4.78(3.18 to 7.04)	0.72(0.34 to 1.2)
Latvia	Liver cancer due to other causes	127(79 to 172)	89(62 to 123)	-0.3(-0.55 to 0.15)	5.26(3.12 to 7.13)	3.98(2.9 to 5.34)	-0.24(-0.54 to 0.41)
Lebanon	Liver cancer	2175(1745 to 2681)	3748(2769 to 5175)	0.72(0.18 to 1.47)	87.82(70.92 to 108.32)	71.78(53.28 to 98.96)	-0.18(-0.44 to 0.18)
Lebanon	Liver cancer due to alcohol use	253(154 to 380)	453(262 to 753)	0.79(0.17 to 1.74)	10.6(6.56 to 15.76)	8.69(4.97 to 14.44)	-0.18(-0.46 to 0.25)
Lebanon	Liver cancer due to hepatitis B	1224(922 to 1585)	2031(1422 to 2859)	0.66(0.12 to 1.42)	48.69(36.91 to 62.7)	38.77(27.04 to 54.69)	-0.2(-0.46 to 0.16)
Lebanon	Liver cancer due to hepatitis C	376(226 to 562)	701(421 to 1105)	0.86(0.22 to 1.93)	16.76(10.59 to 24.77)	13.48(8.06 to 21.3)	-0.2(-0.47 to 0.21)

Lebanon	Liver cancer due to NASH	137(90 to 203)	322(206 to 498)	1.34(0.5 to 2.69)	5.92(3.91 to 8.68)	6.14(3.95 to 9.53)	0.04(-0.32 to 0.6)
Lebanon	Liver cancer due to other causes	184(128 to 266)	242(167 to 348)	0.32(-0.11 to 0.94)	5.84(4.09 to 8.13)	4.69(3.23 to 6.71)	-0.2(-0.43 to 0.14)
Lesotho	Liver cancer	1547(916 to 3378)	6071(2316 to 9819)	2.92(-0.14 to 7.95)	141.04(84.44 to 303.72)	410.68(162.44 to 658.81)	1.91(-0.33 to 5.45)
Lesotho	Liver cancer due to alcohol use	271(126 to 670)	1310(421 to 2237)	3.84(-0.1 to 10.55)	25.61(11.96 to 62.77)	92.87(30.37 to 155.81)	2.63(-0.32 to 7.44)
Lesotho	Liver cancer due to hepatitis B	694(356 to 1626)	2855(943 to 4999)	3.11(-0.25 to 9.47)	60.14(31.21 to 141.68)	178.63(59.46 to 307.63)	1.97(-0.44 to 6.26)
Lesotho	Liver cancer due to hepatitis C	358(200 to 682)	1107(464 to 1901)	2.09(-0.03 to 5.56)	36.5(20.99 to 68.27)	85.9(38.24 to 146.39)	1.35(-0.23 to 3.95)
Lesotho	Liver cancer due to NASH	113(59 to 223)	478(191 to 837)	3.24(0.14 to 8.58)	10.66(5.66 to 21.18)	33.89(14 to 57.83)	2.18(-0.1 to 5.88)
Lesotho	Liver cancer due to other causes	111(68 to 202)	322(139 to 550)	1.89(-0.15 to 5.18)	8.12(4.75 to 15.09)	19.4(8.22 to 33.21)	1.39(-0.31 to 4.13)
Liberia	Liver cancer	2353(1830 to 3045)	3369(2328 to 4980)	0.43(-0.01 to 1.08)	185.5(144.77 to 241.91)	129.81(92.15 to 188.24)	-0.3(-0.51 to 0)
Liberia	Liver cancer due to alcohol use	371(229 to 579)	514(301 to 842)	0.39(-0.08 to 1.14)	31.88(19.82 to 49.43)	22.97(13.69 to 37.44)	-0.28(-0.51 to 0.08)
Liberia	Liver cancer due to hepatitis B	1216(884 to 1615)	1844(1188 to 2846)	0.52(-0.01 to 1.34)	98.84(71.55 to 132.42)	65.57(42.58 to 103.01)	-0.34(-0.56 to 0.01)
Liberia	Liver cancer due to hepatitis C	315(197 to 459)	422(249 to 664)	0.34(-0.07 to 0.93)	28.45(18.01 to 40.65)	21.08(12.47 to 32.22)	-0.26(-0.47 to 0.04)

Liberia	Liver cancer due to NASH	147(98 to 216)	273(173 to 421)	0.86(0.27 to 1.79)	12.65(8.43 to 18.33)	11.84(7.51 to 18.34)	-0.06(-0.35 to 0.38)
Liberia	Liver cancer due to other causes	304(188 to 516)	316(213 to 459)	0.04(-0.39 to 0.71)	13.68(9.12 to 20.16)	8.35(5.48 to 12.31)	-0.39(-0.59 to -0.11)
Libya	Liver cancer	2955(2249 to 3820)	7261(5259 to 10264)	1.46(0.57 to 2.67)	135.1(101.96 to 176.39)	126.13(93.34 to 175.16)	-0.07(-0.4 to 0.41)
Libya	Liver cancer due to alcohol use	233(139 to 367)	645(375 to 1057)	1.77(0.73 to 3.26)	11.95(7.16 to 18.68)	11.9(6.89 to 19.43)	0(-0.37 to 0.53)
Libya	Liver cancer due to hepatitis B	1272(883 to 1738)	3196(2109 to 4710)	1.51(0.52 to 2.96)	56.45(38.43 to 77.88)	49.82(32.89 to 74.06)	-0.12(-0.45 to 0.39)
Libya	Liver cancer due to hepatitis C	754(483 to 1110)	1978(1238 to 2995)	1.62(0.62 to 3.01)	40.81(26.43 to 60.1)	38.59(24.74 to 57.72)	-0.05(-0.41 to 0.43)
Libya	Liver cancer due to NASH	268(171 to 407)	841(552 to 1333)	2.14(0.99 to 3.82)	13.49(8.49 to 20.43)	15.56(10.01 to 24.64)	0.15(-0.25 to 0.74)
Libya	Liver cancer due to other causes	428(292 to 637)	602(400 to 892)	0.41(-0.1 to 1.19)	12.4(8.31 to 17.9)	10.26(7.08 to 14.88)	-0.17(-0.45 to 0.21)
Lithuania	Liver cancer	1875(1745 to 2010)	3732(2927 to 4609)	0.99(0.56 to 1.46)	43.15(39.83 to 46.44)	75.76(59.52 to 93.69)	0.76(0.38 to 1.19)
Lithuania	Liver cancer due to alcohol use	647(471 to 807)	1707(1209 to 2251)	1.64(0.97 to 2.38)	14.4(10.56 to 17.91)	33.74(23.92 to 44.6)	1.34(0.74 to 2.02)
Lithuania	Liver cancer due to hepatitis B	511(379 to 683)	806(532 to 1190)	0.58(0.19 to 1.01)	11.72(8.77 to 15.48)	18.16(12.09 to 26.43)	0.55(0.16 to 0.98)
Lithuania	Liver cancer due to hepatitis C	441(310 to 581)	830(540 to 1144)	0.88(0.47 to 1.35)	9.7(6.85 to 12.75)	15.02(9.68 to 20.86)	0.55(0.21 to 0.92)

Lithuania	Liver cancer due to NASH	119(83 to 166)	256(167 to 381)	1.16(0.66 to 1.73)	2.66(1.88 to 3.69)	4.89(3.22 to 7.28)	0.84(0.39 to 1.35)
Lithuania	Liver cancer due to other causes	158(98 to 216)	132(92 to 187)	-0.16(-0.46 to 0.36)	4.68(2.76 to 6.45)	3.95(3 to 5.13)	-0.16(-0.45 to 0.48)
Luxembourg	Liver cancer	357(332 to 386)	738(599 to 924)	1.06(0.69 to 1.55)	68.15(63.45 to 73.29)	78.63(63.92 to 98.59)	0.15(-0.05 to 0.42)
Luxembourg	Liver cancer due to alcohol use	150(113 to 185)	297(207 to 402)	0.98(0.55 to 1.53)	28.04(21.33 to 34.53)	31.44(21.81 to 42.49)	0.12(-0.12 to 0.43)
Luxembourg	Liver cancer due to hepatitis B	52(36 to 74)	100(65 to 150)	0.92(0.54 to 1.4)	10.31(7.27 to 14.38)	11.19(7.37 to 16.58)	0.09(-0.13 to 0.36)
Luxembourg	Liver cancer due to hepatitis C	116(84 to 152)	256(178 to 354)	1.21(0.82 to 1.73)	21.17(15.51 to 27.91)	25.97(17.93 to 36.26)	0.23(0.01 to 0.51)
Luxembourg	Liver cancer due to NASH	19(13 to 27)	45(30 to 66)	1.39(0.92 to 1.97)	3.48(2.45 to 5)	4.69(3.15 to 6.86)	0.35(0.09 to 0.67)
Luxembourg	Liver cancer due to other causes	21(16 to 27)	40(28 to 57)	0.9(0.52 to 1.37)	5.15(4.14 to 6.33)	5.34(3.81 to 7.23)	0.04(-0.18 to 0.29)
Madagascar	Liver cancer	5814(4277 to 9354)	9707(6694 to 14402)	0.67(0.19 to 1.38)	82.75(59.45 to 138.9)	66.8(45.3 to 100.51)	-0.19(-0.43 to 0.14)
Madagascar	Liver cancer due to alcohol use	989(512 to 2051)	1816(971 to 3315)	0.84(0.24 to 1.72)	18.05(9.28 to 37.3)	15(8.14 to 27.3)	-0.17(-0.43 to 0.24)
Madagascar	Liver cancer due to hepatitis B	1985(1314 to 3445)	3472(2135 to 5628)	0.75(0.18 to 1.51)	29.8(19.08 to 52.86)	22.05(13.14 to 36.64)	-0.26(-0.5 to 0.07)
Madagascar	Liver cancer due to hepatitis C	791(484 to 1283)	1523(911 to 2377)	0.92(0.34 to 1.78)	15.32(9.47 to 24.85)	13.96(8.59 to 21.39)	-0.09(-0.36 to 0.29)
Madagascar	Liver cancer due to NASH	486(310 to 776)	986(592 to 1566)	1.03(0.37 to 1.89)	8.27(5.11 to 13.55)	7.78(4.69 to	-0.06(-0.35 to 0.33)

						12.35)	
Madagascar	Liver cancer due to other causes	1563(923 to 2486)	1911(1243 to 2859)	0.22(-0.25 to 1.09)	11.32(7.28 to 17.16)	8.01(5.33 to 11.77)	-0.29(-0.52 to 0.06)
Malawi	Liver cancer	7348(5031 to 10466)	9302(7136 to 12045)	0.27(-0.16 to 0.88)	102.04(77.12 to 142.11)	80.46(63.34 to 101.04)	-0.21(-0.45 to 0.09)
Malawi	Liver cancer due to alcohol use	796(449 to 1433)	1319(848 to 1913)	0.66(0.07 to 1.49)	18.87(10.73 to 33.29)	17.35(11.29 to 24.96)	-0.08(-0.4 to 0.36)
Malawi	Liver cancer due to hepatitis B	1520(1021 to 2384)	2106(1420 to 3019)	0.39(-0.1 to 1.06)	30.12(19.99 to 47.6)	21.48(14.26 to 30.75)	-0.29(-0.53 to 0.05)
Malawi	Liver cancer due to hepatitis C	663(407 to 1021)	997(634 to 1400)	0.5(0.08 to 1.12)	17.09(10.83 to 25.97)	14.49(9.32 to 20.16)	-0.15(-0.38 to 0.18)
Malawi	Liver cancer due to NASH	403(267 to 609)	674(437 to 981)	0.67(0.18 to 1.39)	9.21(5.95 to 13.95)	8.43(5.5 to 12.32)	-0.08(-0.35 to 0.28)
Malawi	Liver cancer due to other causes	3965(2066 to 7076)	4207(2908 to 5898)	0.06(-0.42 to 0.92)	26.76(15.62 to 44.7)	18.71(13.28 to 25.36)	-0.3(-0.59 to 0.16)
Malaysia	Liver cancer	15342(13087 to 17935)	43385(32755 to 56853)	1.83(1.01 to 2.92)	146.1(125 to 171.23)	151.38(115.04 to 197.82)	0.04(-0.26 to 0.43)
Malaysia	Liver cancer due to alcohol use	1733(1133 to 2540)	5525(3386 to 8662)	2.19(1.16 to 3.62)	18(11.65 to 26.49)	19.65(12.13 to 30.39)	0.09(-0.25 to 0.54)
Malaysia	Liver cancer due to hepatitis B	9883(7825 to 12023)	26745(19075 to 35777)	1.71(0.87 to 2.77)	90.27(71.75 to 109.76)	90.42(64.58 to 120.12)	0(-0.31 to 0.39)
Malaysia	Liver cancer due to hepatitis C	1924(1205 to 2766)	5632(3564 to 8674)	1.93(1.16 to 3.03)	21.58(13.62 to 30.82)	21.42(13.62 to 31.89)	-0.01(-0.27 to 0.37)
Malaysia	Liver cancer due to NASH	1088(748 to 1533)	4193(2672 to 6341)	2.85(1.74 to 4.47)	11.27(7.76 to 16.17)	15.38(9.87 to	0.36(-0.02 to 0.93)

						23.08)	
Malaysia	Liver cancer due to other causes	713(549 to 917)	1290(868 to 1852)	0.81(0.3 to 1.51)	4.98(3.66 to 6.6)	4.51(3.07 to 6.44)	-0.1(-0.34 to 0.22)
Maldives	Liver cancer	178(118 to 279)	444(344 to 552)	1.5(0.53 to 3.02)	172.49(116.74 to 262.31)	126.24(98.62 to 155.18)	-0.27(-0.54 to 0.13)
Maldives	Liver cancer due to alcohol use	33(18 to 60)	89(57 to 132)	1.73(0.66 to 3.45)	34.4(19.25 to 60.34)	27.68(17.72 to 40.41)	-0.2(-0.5 to 0.26)
Maldives	Liver cancer due to hepatitis B	90(55 to 151)	222(160 to 294)	1.46(0.48 to 3.18)	82.16(50.46 to 134.56)	54.92(39.12 to 73.21)	-0.33(-0.59 to 0.09)
Maldives	Liver cancer due to hepatitis C	33(17 to 60)	80(52 to 114)	1.47(0.48 to 2.96)	38.32(21.22 to 67.1)	28.06(18.39 to 39.44)	-0.27(-0.53 to 0.15)
Maldives	Liver cancer due to NASH	10(6 to 19)	35(23 to 51)	2.33(1 to 4.5)	11.2(6.43 to 19.49)	10.96(7.15 to 16.42)	-0.02(-0.39 to 0.55)
Maldives	Liver cancer due to other causes	12(7 to 21)	18(13 to 25)	0.46(-0.12 to 1.42)	6.42(3.83 to 11.25)	4.61(3.32 to 6.45)	-0.28(-0.55 to 0.11)
Mali	Liver cancer	23956(19666 to 28682)	43691(31736 to 58788)	0.82(0.27 to 1.5)	445.32(363.38 to 537.01)	408.97(298.64 to 547.03)	-0.08(-0.35 to 0.25)
Mali	Liver cancer due to alcohol use	3058(1861 to 4613)	6561(3932 to 10215)	1.15(0.45 to 2.1)	66.05(40.51 to 98.92)	68.05(41.23 to 106.52)	0.03(-0.3 to 0.46)
Mali	Liver cancer due to hepatitis B	9447(6877 to 12255)	18379(11731 to 26849)	0.95(0.32 to 1.76)	187.25(135.07 to 244.78)	166.99(107.37 to 245.22)	-0.11(-0.39 to 0.25)
Mali	Liver cancer due to hepatitis C	5768(3818 to 8183)	11061(6965 to 16327)	0.92(0.39 to 1.58)	131.08(88.05 to 181.99)	122.21(78.52 to 176.53)	-0.07(-0.32 to 0.24)
Mali	Liver cancer due to NASH	1091(742 to 1592)	2647(1645 to 4097)	1.43(0.65 to 2.38)	23.38(15.98 to 33.67)	26.68(16.91 to	0.14(-0.21 to 0.57)

						41.18)	
Mali	Liver cancer due to other causes	4592(2980 to 6881)	5042(3304 to 7501)	0.1(-0.35 to 0.87)	37.57(26.38 to 52.11)	25.05(16.67 to 36.26)	-0.33(-0.57 to 0)
Malta	Liver cancer	160(146 to 173)	414(361 to 471)	1.59(1.21 to 2.02)	37.55(34.42 to 40.67)	50(43.85 to 56.83)	0.33(0.14 to 0.56)
Malta	Liver cancer due to alcohol use	52(37 to 66)	153(111 to 202)	1.95(1.42 to 2.56)	11.91(8.58 to 15.26)	17.69(12.84 to 22.86)	0.49(0.22 to 0.79)
Malta	Liver cancer due to hepatitis B	27(19 to 38)	58(40 to 86)	1.17(0.8 to 1.6)	6.34(4.49 to 8.9)	8.04(5.63 to 11.32)	0.27(0.06 to 0.52)
Malta	Liver cancer due to hepatitis C	58(43 to 75)	150(108 to 200)	1.58(1.16 to 2.09)	13.53(10.03 to 17.27)	16.79(12.15 to 22.52)	0.24(0.04 to 0.47)
Malta	Liver cancer due to NASH	9(6 to 13)	28(19 to 41)	2.08(1.49 to 2.79)	2.14(1.44 to 3.1)	3.27(2.26 to 4.65)	0.53(0.25 to 0.89)
Malta	Liver cancer due to other causes	13(11 to 17)	23(17 to 32)	0.73(0.4 to 1.11)	3.64(2.91 to 4.48)	4.22(3.21 to 5.44)	0.16(-0.04 to 0.38)
Marshall Islands	Liver cancer	64(50 to 82)	124(86 to 172)	0.95(0.38 to 1.69)	325.73(249.51 to 422.87)	288.32(205.15 to 393.78)	-0.11(-0.36 to 0.22)
Marshall Islands	Liver cancer due to alcohol use	6(4 to 10)	14(8 to 23)	1.15(0.47 to 2.1)	36.56(20.93 to 57.31)	34.41(20.02 to 56.15)	-0.06(-0.34 to 0.33)
Marshall Islands	Liver cancer due to hepatitis B	39(29 to 52)	76(51 to 110)	0.95(0.35 to 1.74)	191.16(137.43 to 265.35)	165.22(111.26 to 236.35)	-0.14(-0.39 to 0.21)
Marshall Islands	Liver cancer due to hepatitis C	10(6 to 15)	18(10 to 29)	0.89(0.32 to 1.66)	60.97(37.88 to 93.76)	52.22(31.08 to 78.59)	-0.14(-0.38 to 0.18)
Marshall Islands	Liver cancer due to NASH	4(3 to 6)	9(5 to 14)	1.25(0.57 to 2.2)	20.99(13.65 to 31.97)	22.09(13.97 to	0.05(-0.26 to 0.47)

						34.22)	
Marshall Islands	Liver cancer due to other causes	4(3 to 6)	7(4 to 10)	0.59(0.12 to 1.22)	16.05(10.41 to 22.75)	14.38(9.07 to 21.27)	-0.1(-0.36 to 0.25)
Mauritania	Liver cancer	2179(1760 to 2682)	2478(1714 to 3396)	0.14(-0.24 to 0.59)	188.88(150.59 to 232.6)	105.08(74.57 to 139.79)	-0.44(-0.61 to -0.23)
Mauritania	Liver cancer due to alcohol use	267(161 to 416)	318(186 to 495)	0.19(-0.2 to 0.7)	25.29(15.48 to 39.09)	14.81(8.57 to 23.05)	-0.41(-0.6 to -0.17)
Mauritania	Liver cancer due to hepatitis B	1212(915 to 1561)	1310(847 to 1847)	0.08(-0.31 to 0.57)	105.01(78.39 to 134.76)	53.49(35.09 to 74.88)	-0.49(-0.67 to -0.28)
Mauritania	Liver cancer due to hepatitis C	311(188 to 452)	364(219 to 538)	0.17(-0.17 to 0.61)	31.17(19.21 to 45.2)	17.93(11.09 to 26.04)	-0.42(-0.58 to -0.21)
Mauritania	Liver cancer due to NASH	163(110 to 234)	254(160 to 372)	0.56(0.04 to 1.22)	15.3(10.28 to 22.05)	11.58(7.36 to 16.94)	-0.24(-0.48 to 0.06)
Mauritania	Liver cancer due to other causes	226(161 to 312)	233(145 to 348)	0.03(-0.39 to 0.61)	12.1(8.54 to 16.38)	7.27(4.57 to 10.8)	-0.4(-0.61 to -0.13)
Mauritius	Liver cancer	381(352 to 408)	793(603 to 1030)	1.08(0.56 to 1.8)	47.11(43.53 to 50.59)	45.63(35.11 to 59.2)	-0.03(-0.27 to 0.3)
Mauritius	Liver cancer due to alcohol use	73(51 to 101)	187(115 to 281)	1.55(0.8 to 2.58)	9.52(6.5 to 13.01)	10.33(6.48 to 15.43)	0.09(-0.22 to 0.53)
Mauritius	Liver cancer due to hepatitis B	169(135 to 209)	301(201 to 428)	0.78(0.27 to 1.45)	19.83(15.52 to 24.81)	17.08(11.54 to 23.95)	-0.14(-0.37 to 0.18)
Mauritius	Liver cancer due to hepatitis C	80(55 to 105)	189(121 to 282)	1.37(0.8 to 2.09)	11.01(7.65 to 14.34)	10.86(7.1 to 15.9)	-0.01(-0.24 to 0.28)
Mauritius	Liver cancer due to NASH	30(22 to 42)	85(55 to 129)	1.8(1.05 to 2.75)	3.93(2.79 to 5.57)	4.92(3.21 to	0.25(-0.07 to 0.67)

						7.41)	
Mauritius	Liver cancer due to other causes	28(23 to 33)	31(21 to 42)	0.09(-0.21 to 0.47)	2.82(2.26 to 3.42)	2.44(1.8 to 3.21)	-0.13(-0.33 to 0.14)
Mexico	Liver cancer	27882(26999 to 28855)	97973(84151 to 111931)	2.51(2 to 3.07)	56.05(54.31 to 57.51)	82.76(71.12 to 94.63)	0.48(0.27 to 0.7)
Mexico	Liver cancer due to alcohol use	6508(5664 to 7417)	29901(23793 to 36911)	3.59(2.87 to 4.37)	14.65(12.72 to 16.65)	25.12(20.1 to 30.94)	0.71(0.45 to 1)
Mexico	Liver cancer due to hepatitis B	4477(3821 to 5193)	13544(10765 to 16711)	2.03(1.56 to 2.55)	8.57(7.23 to 10.11)	10.93(8.69 to 13.56)	0.28(0.08 to 0.49)
Mexico	Liver cancer due to hepatitis C	9017(8075 to 9948)	35486(29513 to 42435)	2.94(2.39 to 3.58)	21.48(19.3 to 23.66)	30.54(25.48 to 36.54)	0.42(0.23 to 0.65)
Mexico	Liver cancer due to NASH	2054(1783 to 2361)	9558(7860 to 11659)	3.65(2.97 to 4.42)	4.39(3.78 to 5.11)	8.08(6.64 to 9.88)	0.84(0.58 to 1.14)
Mexico	Liver cancer due to other causes	5825(5208 to 6614)	9484(7861 to 11260)	0.63(0.3 to 1.02)	6.96(6.3 to 7.72)	8.08(6.67 to 9.66)	0.16(-0.05 to 0.4)
Micronesia (Federated States of)	Liver cancer	171(123 to 230)	245(151 to 367)	0.43(-0.12 to 1.18)	315.59(230.11 to 415.7)	290.57(185.95 to 424.89)	-0.08(-0.42 to 0.38)
Micronesia (Federated States of)	Liver cancer due to alcohol use	18(11 to 29)	28(14 to 47)	0.53(-0.06 to 1.38)	36.18(21.2 to 58.14)	34.6(18.79 to 56.24)	-0.04(-0.4 to 0.45)
Micronesia (Federated States of)	Liver cancer due to hepatitis B	103(70 to 143)	146(87 to 229)	0.42(-0.15 to 1.22)	181.65(123.25 to 251.79)	163.83(97.89 to 252.29)	-0.1(-0.46 to 0.39)

Micronesia (Federated States of)	Liver cancer due to hepatitis C	27(16 to 42)	38(20 to 63)	0.4(-0.12 to 1.07)	59.03(35.81 to 90.37)	52.72(29.74 to 83.22)	-0.11(-0.41 to 0.32)
Micronesia (Federated States of)	Liver cancer due to NASH	12(7 to 18)	20(11 to 33)	0.69(0.07 to 1.63)	22.99(14.42 to 35.48)	25.55(14.77 to 41.09)	0.11(-0.27 to 0.71)
Micronesia (Federated States of)	Liver cancer due to other causes	11(7 to 16)	12(7 to 21)	0.13(-0.28 to 0.63)	15.73(9.85 to 22.89)	13.86(8.02 to 22.9)	-0.12(-0.43 to 0.28)
Monaco	Liver cancer	45(35 to 57)	140(110 to 175)	2.09(1.26 to 3.23)	76.52(59.87 to 94.85)	171.86(133.37 to 219.55)	1.25(0.65 to 2.08)
Monaco	Liver cancer due to alcohol use	16(11 to 22)	51(35 to 71)	2.18(1.33 to 3.37)	26.06(17.7 to 36.62)	61.63(41.84 to 85.79)	1.37(0.7 to 2.31)
Monaco	Liver cancer due to hepatitis B	7(5 to 11)	20(13 to 30)	1.76(0.97 to 2.88)	13.95(9.28 to 20.56)	28.81(18.5 to 41.83)	1.07(0.49 to 1.96)
Monaco	Liver cancer due to hepatitis C	17(11 to 23)	52(35 to 70)	2.11(1.29 to 3.29)	24.99(16.38 to 35.13)	57.84(39.39 to 79.22)	1.31(0.67 to 2.23)
Monaco	Liver cancer due to NASH	3(2 to 5)	11(7 to 17)	2.49(1.52 to 3.88)	4.96(3.15 to 7.64)	13(8.54 to 19.68)	1.62(0.88 to 2.66)
Monaco	Liver cancer due to other causes	2(2 to 4)	7(4 to 10)	1.77(1.03 to 2.78)	6.57(4.77 to 8.8)	10.57(7.44 to 14.91)	0.61(0.19 to 1.13)
Mongolia	Liver cancer	19131(15136 to 23427)	65007(48959 to 85363)	2.4(1.43 to 3.79)	1726.2(1371.23 to 2109.52)	2558.12(1961.96 to 3278.63)	0.48(0.08 to 1.04)
Mongolia	Liver cancer due to alcohol use	4915(3027 to 7088)	19948(12804 to 29861)	3.06(1.82 to 4.73)	450.94(280.39 to 646.63)	786.56(516.06 to 1129.99)	0.74(0.23 to 1.42)
Mongolia	Liver cancer due to hepatitis B	7302(5178 to 10026)	22386(14476 to 33100)	2.07(1.11 to 3.41)	633.01(443.92 to 876.85)	763.2(508.16 to 1105.78)	0.21(-0.15 to 0.7)

Mongolia	Liver cancer due to hepatitis C	5292(3483 to 7497)	16530(10580 to 23937)	2.12(1.26 to 3.33)	501.73(334.19 to 709.19)	752.55(511.89 to 1044.07)	0.5(0.11 to 1.08)
Mongolia	Liver cancer due to NASH	884(566 to 1339)	3787(2396 to 5766)	3.28(2.03 to 4.99)	81.75(52.21 to 123.99)	167.2(106.94 to 249.06)	1.05(0.48 to 1.81)
Mongolia	Liver cancer due to other causes	737(509 to 1084)	2355(1495 to 3578)	2.2(1.25 to 3.56)	58.77(38.32 to 88.01)	88.6(57.26 to 133.02)	0.51(0.06 to 1.14)
Montenegro	Liver cancer	1005(827 to 1169)	1396(1130 to 1715)	0.39(0.06 to 0.92)	156.93(129.85 to 181.73)	146.43(119.21 to 179.4)	-0.07(-0.29 to 0.28)
Montenegro	Liver cancer due to alcohol use	413(298 to 532)	592(417 to 794)	0.43(0.05 to 1.07)	64.4(46.76 to 82.77)	60.12(42.74 to 80.01)	-0.07(-0.31 to 0.33)
Montenegro	Liver cancer due to hepatitis B	277(194 to 381)	331(222 to 482)	0.2(-0.1 to 0.65)	42.33(29.91 to 58)	36.73(25.19 to 52.33)	-0.13(-0.34 to 0.18)
Montenegro	Liver cancer due to hepatitis C	200(132 to 284)	313(203 to 454)	0.57(0.18 to 1.16)	31.78(21.15 to 44.64)	31.65(20.82 to 45.25)	0(-0.25 to 0.37)
Montenegro	Liver cancer due to NASH	64(43 to 90)	108(73 to 158)	0.7(0.26 to 1.38)	10.1(6.86 to 14.13)	11.34(7.79 to 16.25)	0.12(-0.16 to 0.57)
Montenegro	Liver cancer due to other causes	51(39 to 67)	52(35 to 74)	0.01(-0.25 to 0.33)	8.31(6.43 to 10.75)	6.58(4.76 to 9.07)	-0.21(-0.39 to 0)
Morocco	Liver cancer	6504(4929 to 8029)	15336(11513 to 19411)	1.36(0.67 to 2.29)	45.03(33.45 to 55.34)	48.56(36.92 to 60.46)	0.08(-0.23 to 0.51)
Morocco	Liver cancer due to alcohol use	648(383 to 1014)	1720(996 to 2691)	1.66(0.82 to 2.83)	4.73(2.78 to 7.36)	5.53(3.31 to 8.54)	0.17(-0.2 to 0.68)
Morocco	Liver cancer due to hepatitis B	2784(1999 to 3790)	6144(4118 to 8833)	1.21(0.49 to 2.26)	18.46(13.08 to 25.48)	18.19(12.24 to 25.9)	-0.01(-0.32 to 0.44)

Morocco	Liver cancer due to hepatitis C	1837(1168 to 2593)	4687(3051 to 6524)	1.55(0.83 to 2.59)	14.39(9.34 to 19.92)	15.91(10.5 to 21.72)	0.11(-0.2 to 0.54)
Morocco	Liver cancer due to NASH	520(334 to 780)	1628(1034 to 2462)	2.13(1.14 to 3.46)	3.86(2.4 to 5.84)	5.38(3.46 to 8.05)	0.39(-0.04 to 0.99)
Morocco	Liver cancer due to other causes	715(515 to 981)	1157(781 to 1668)	0.62(0.11 to 1.32)	3.59(2.52 to 4.86)	3.56(2.41 to 5.09)	-0.01(-0.27 to 0.37)
Mozambique	Liver cancer	4803(3352 to 7400)	16351(11107 to 22347)	2.4(0.68 to 4.65)	57.88(41.43 to 92.52)	101.55(69.38 to 135.39)	0.75(-0.2 to 1.81)
Mozambique	Liver cancer due to alcohol use	665(363 to 1289)	2575(1483 to 3824)	2.87(0.47 to 5.78)	10.7(5.87 to 20.77)	22.5(12.87 to 33.12)	1.1(-0.2 to 2.68)
Mozambique	Liver cancer due to hepatitis B	1811(1150 to 3181)	6380(3958 to 9343)	2.52(0.42 to 5.12)	24.1(15.23 to 42.77)	42.24(26.4 to 60.28)	0.75(-0.29 to 2.02)
Mozambique	Liver cancer due to hepatitis C	500(293 to 816)	1364(771 to 2053)	1.73(0.47 to 3.28)	8.88(5.3 to 14.08)	13.32(7.95 to 19.87)	0.5(-0.19 to 1.35)
Mozambique	Liver cancer due to NASH	349(222 to 541)	1183(751 to 1780)	2.39(0.79 to 4.38)	5.53(3.49 to 8.66)	9.79(6.33 to 14.63)	0.77(-0.04 to 1.8)
Mozambique	Liver cancer due to other causes	1478(842 to 2633)	4848(2787 to 8120)	2.28(0.39 to 5.82)	8.66(5.4 to 13.57)	13.69(8.76 to 20.93)	0.58(-0.17 to 1.85)
Myanmar	Liver cancer	20367(14397 to 30561)	53941(43687 to 66342)	1.65(0.67 to 2.93)	74.59(53.56 to 110.35)	110.14(90.4 to 133.65)	0.48(-0.02 to 1.11)
Myanmar	Liver cancer due to alcohol use	2911(1780 to 4896)	9695(6176 to 14279)	2.33(1.14 to 3.97)	12.19(7.52 to 20.3)	20.22(12.99 to 29.48)	0.66(0.09 to 1.43)
Myanmar	Liver cancer due to hepatitis B	7651(4955 to 11833)	19566(13922 to 27188)	1.56(0.58 to 2.83)	28.41(18.44 to 43.49)	37.75(26.95 to 51.72)	0.33(-0.16 to 0.96)

Myanmar	Liver cancer due to hepatitis C	4489(2648 to 7169)	13040(8653 to 18080)	1.9(0.98 to 3.2)	20.14(12.34 to 31.34)	28.65(19.55 to 38.88)	0.42(-0.01 to 1.01)
Myanmar	Liver cancer due to NASH	1425(881 to 2269)	5035(3372 to 7311)	2.53(1.24 to 4.36)	5.89(3.69 to 9.37)	10.58(7.15 to 15.32)	0.8(0.19 to 1.63)
Myanmar	Liver cancer due to other causes	3891(2142 to 7653)	6605(4238 to 9628)	0.7(-0.2 to 2.22)	7.96(4.74 to 14.51)	12.96(8.35 to 18.92)	0.63(-0.18 to 1.95)
Namibia	Liver cancer	409(250 to 750)	1438(1029 to 1928)	2.52(0.82 to 5.48)	50.57(30.59 to 92.54)	89.39(65.2 to 118.25)	0.77(-0.07 to 2.21)
Namibia	Liver cancer due to alcohol use	72(33 to 168)	324(201 to 488)	3.5(1.1 to 7.82)	9.4(4.34 to 22.06)	21.26(13.36 to 31.61)	1.26(0.08 to 3.46)
Namibia	Liver cancer due to hepatitis B	158(81 to 329)	572(363 to 820)	2.61(0.69 to 6.51)	19.33(9.82 to 40.35)	33.16(21.46 to 47.25)	0.72(-0.2 to 2.49)
Namibia	Liver cancer due to hepatitis C	102(61 to 171)	305(192 to 447)	1.98(0.79 to 3.95)	14.14(8.56 to 23.34)	21.65(13.9 to 31.12)	0.53(-0.07 to 1.51)
Namibia	Liver cancer due to NASH	31(17 to 57)	112(69 to 166)	2.59(1.03 to 5.33)	4.04(2.23 to 7.44)	7.34(4.61 to 10.97)	0.82(0.04 to 2.15)
Namibia	Liver cancer due to other causes	45(27 to 71)	125(83 to 186)	1.79(0.64 to 3.86)	3.66(2.25 to 6.01)	5.98(3.97 to 8.78)	0.64(-0.04 to 1.81)
Nauru	Liver cancer	16(11 to 22)	16(10 to 24)	0.01(-0.27 to 0.44)	298.17(208.98 to 392.54)	256.69(177.05 to 363.21)	-0.14(-0.37 to 0.2)
Nauru	Liver cancer due to alcohol use	2(1 to 3)	2(1 to 3)	0.1(-0.26 to 0.66)	33.76(19.92 to 53.4)	31.51(17.21 to 50.17)	-0.07(-0.34 to 0.34)
Nauru	Liver cancer due to hepatitis B	10(6 to 14)	10(6 to 16)	-0.01(-0.3 to 0.46)	173.27(115.45 to 239.64)	142.42(91.8 to 213.37)	-0.18(-0.42 to 0.19)

Nauru	Liver cancer due to hepatitis C	2(1 to 3)	2(1 to 3)	-0.01(-0.3 to 0.37)	53.31(33.74 to 79.37)	45.67(27.09 to 70.21)	-0.14(-0.37 to 0.15)
Nauru	Liver cancer due to NASH	1(1 to 2)	1(1 to 2)	0.22(-0.14 to 0.69)	21.76(14.02 to 32.33)	22.61(13.9 to 35.93)	0.04(-0.24 to 0.44)
Nauru	Liver cancer due to other causes	1(1 to 2)	1(1 to 2)	-0.05(-0.28 to 0.26)	16.07(10.24 to 23.8)	14.47(9.38 to 22.84)	-0.1(-0.32 to 0.19)
Nepal	Liver cancer	6764(4928 to 9456)	12973(9421 to 18219)	0.92(0.3 to 1.74)	50.47(38.19 to 65.37)	54.37(39.46 to 76.65)	0.08(-0.26 to 0.48)
Nepal	Liver cancer due to alcohol use	1123(747 to 1640)	3108(1805 to 5272)	1.77(0.73 to 3.07)	11.47(7.74 to 16.32)	13.63(8.02 to 23.06)	0.19(-0.26 to 0.74)
Nepal	Liver cancer due to hepatitis B	1657(1108 to 2435)	3275(2035 to 5132)	0.98(0.29 to 1.86)	13.62(9.12 to 19.68)	13.01(8.17 to 20.4)	-0.04(-0.39 to 0.35)
Nepal	Liver cancer due to hepatitis C	1261(793 to 1901)	3428(2246 to 5425)	1.72(0.81 to 2.77)	13.84(8.96 to 20.06)	15.95(10.64 to 24.86)	0.15(-0.23 to 0.59)
Nepal	Liver cancer due to NASH	384(249 to 596)	1141(731 to 1735)	1.97(1 to 3.3)	3.61(2.39 to 5.58)	4.91(3.2 to 7.44)	0.36(-0.09 to 0.92)
Nepal	Liver cancer due to other causes	2339(1338 to 4179)	2021(1337 to 3422)	-0.14(-0.58 to 0.92)	7.93(4.94 to 13.38)	6.86(4.56 to 11.57)	-0.13(-0.54 to 0.85)
Netherlands	Liver cancer	6142(5847 to 6451)	18697(17186 to 20224)	2.04(1.81 to 2.33)	32.94(31.37 to 34.58)	61.6(56.93 to 66.65)	0.87(0.72 to 1.03)
Netherlands	Liver cancer due to alcohol use	2517(1994 to 3032)	7827(6049 to 9648)	2.11(1.77 to 2.47)	13.06(10.34 to 15.74)	24.81(19.28 to 30.41)	0.9(0.71 to 1.12)
Netherlands	Liver cancer due to hepatitis B	951(684 to 1310)	2589(1811 to 3648)	1.72(1.39 to 2.13)	5.25(3.81 to 7.25)	9.71(7.05 to 13.22)	0.85(0.64 to 1.09)
Netherlands	Liver cancer due to hepatitis C	1765(1302 to 2273)	5712(4178 to 7424)	2.24(1.91 to 2.62)	9(6.69 to 11.67)	17.34(12.61 to	0.93(0.73 to 1.15)

						22.47)	
Netherlands	Liver cancer due to NASH	400(281 to 559)	1444(1002 to 2020)	2.61(2.11 to 3.15)	2.06(1.46 to 2.87)	4.59(3.27 to 6.39)	1.23(0.94 to 1.56)
Netherlands	Liver cancer due to other causes	509(408 to 633)	1125(827 to 1487)	1.21(0.85 to 1.53)	3.58(2.98 to 4.25)	5.15(4.1 to 6.38)	0.44(0.25 to 0.63)
New Zealand	Liver cancer	2132(1988 to 2290)	6465(5989 to 6969)	2.03(1.72 to 2.38)	57.31(53.26 to 61.58)	94.37(87.43 to 101.74)	0.65(0.48 to 0.83)
New Zealand	Liver cancer due to alcohol use	869(749 to 987)	2512(2178 to 2863)	1.89(1.55 to 2.28)	23.12(19.92 to 26.4)	35.85(31.21 to 40.89)	0.55(0.37 to 0.76)
New Zealand	Liver cancer due to hepatitis B	445(375 to 530)	1175(979 to 1425)	1.64(1.33 to 2.01)	12.2(10.24 to 14.57)	18.93(15.91 to 22.65)	0.55(0.38 to 0.75)
New Zealand	Liver cancer due to hepatitis C	499(427 to 581)	1764(1507 to 2045)	2.54(2.16 to 2.96)	13.04(11.16 to 15.2)	23.79(20.32 to 27.58)	0.82(0.63 to 1.04)
New Zealand	Liver cancer due to NASH	155(131 to 181)	646(547 to 761)	3.16(2.73 to 3.65)	4.07(3.45 to 4.7)	9.14(7.78 to 10.63)	1.25(1.02 to 1.5)
New Zealand	Liver cancer due to other causes	165(148 to 183)	368(316 to 424)	1.23(1.01 to 1.47)	4.88(4.4 to 5.39)	6.67(5.88 to 7.59)	0.37(0.25 to 0.5)
Nicaragua	Liver cancer	1542(1371 to 1719)	4293(3415 to 5359)	1.78(1.17 to 2.52)	81.08(70.94 to 92.01)	91.54(73.23 to 113.19)	0.13(-0.12 to 0.44)
Nicaragua	Liver cancer due to alcohol use	360(247 to 477)	1292(872 to 1823)	2.58(1.68 to 3.84)	22.19(15.28 to 29.7)	28.2(19.1 to 39.78)	0.27(-0.05 to 0.72)
Nicaragua	Liver cancer due to hepatitis B	353(261 to 461)	794(538 to 1139)	1.25(0.67 to 1.99)	16.93(12.19 to 23.19)	15.26(10.1 to 22.15)	-0.1(-0.32 to 0.17)
Nicaragua	Liver cancer due to hepatitis C	446(318 to 577)	1446(1004 to 1961)	2.24(1.47 to 3.13)	28.89(20.74 to 37.54)	33.34(23.66 to	0.15(-0.11 to 0.45)

						45.01)	
Nicaragua	Liver cancer due to NASH	93(68 to 128)	347(233 to 503)	2.72(1.78 to 3.89)	5.1(3.59 to 7.14)	7.46(4.96 to 10.66)	0.46(0.11 to 0.89)
Nicaragua	Liver cancer due to other causes	289(240 to 353)	415(307 to 543)	0.43(0.08 to 0.86)	7.98(6.33 to 10.01)	7.28(5.36 to 9.68)	-0.09(-0.29 to 0.14)
Niger	Liver cancer	657(518 to 812)	1662(1193 to 2240)	1.53(0.8 to 2.52)	18.17(14.03 to 22.72)	16.28(11.83 to 21.83)	-0.1(-0.36 to 0.22)
Niger	Liver cancer due to alcohol use	81(48 to 125)	207(120 to 321)	1.54(0.74 to 2.66)	2.66(1.61 to 4.02)	2.45(1.43 to 3.76)	-0.08(-0.36 to 0.31)
Niger	Liver cancer due to hepatitis B	371(268 to 490)	880(586 to 1235)	1.37(0.63 to 2.37)	10.23(7.39 to 13.63)	8.65(5.76 to 12.04)	-0.15(-0.4 to 0.19)
Niger	Liver cancer due to hepatitis C	84(53 to 124)	220(132 to 328)	1.62(0.92 to 2.59)	3.04(1.95 to 4.44)	2.87(1.77 to 4.21)	-0.06(-0.3 to 0.26)
Niger	Liver cancer due to NASH	34(23 to 51)	100(62 to 152)	1.92(1.06 to 3.07)	1.1(0.71 to 1.65)	1.15(0.72 to 1.73)	0.05(-0.25 to 0.42)
Niger	Liver cancer due to other causes	87(62 to 123)	255(174 to 369)	1.94(0.94 to 3.42)	1.14(0.83 to 1.53)	1.16(0.8 to 1.67)	0.02(-0.29 to 0.43)
Nigeria	Liver cancer	55148(42516 to 68871)	84514(64020 to 109500)	0.53(0.11 to 1.09)	91.59(69.35 to 115.48)	81.23(63.22 to 103.86)	-0.11(-0.35 to 0.22)
Nigeria	Liver cancer due to alcohol use	7351(5196 to 10139)	14753(10646 to 20052)	1.01(0.42 to 1.89)	16.03(11.29 to 22.1)	17.09(12.51 to 22.7)	0.07(-0.24 to 0.54)
Nigeria	Liver cancer due to hepatitis B	20428(15129 to 26789)	33241(24270 to 44300)	0.63(0.12 to 1.34)	38.11(28.07 to 49.93)	32.32(23.88 to 42.64)	-0.15(-0.4 to 0.21)
Nigeria	Liver cancer due to hepatitis C	7392(5222 to 10036)	13574(9918 to 18079)	0.84(0.31 to 1.59)	17.26(12.3 to 23.22)	16.42(12.25 to	-0.05(-0.32 to 0.35)

						21.37)	
Nigeria	Liver cancer due to NASH	3331(2393 to 4444)	7004(5089 to 9464)	1.1(0.48 to 1.99)	7.02(5 to 9.42)	7.83(5.83 to 10.31)	0.12(-0.2 to 0.56)
Nigeria	Liver cancer due to other causes	16646(12414 to 22521)	15941(9243 to 22236)	-0.04(-0.48 to 0.41)	13.17(10.01 to 17.2)	7.56(5.18 to 10.11)	-0.43(-0.63 to -0.2)
Niue	Liver cancer	5(4 to 6)	4(3 to 5)	-0.12(-0.38 to 0.25)	227.34(176.8 to 293.3)	197.67(147.15 to 258.54)	-0.13(-0.39 to 0.24)
Niue	Liver cancer due to alcohol use	1(0 to 1)	1(0 to 1)	0.03(-0.29 to 0.49)	26.56(15.94 to 40.86)	26.15(15.99 to 39.86)	-0.02(-0.32 to 0.42)
Niue	Liver cancer due to hepatitis B	3(2 to 3)	2(2 to 3)	-0.15(-0.43 to 0.23)	129.89(94.24 to 171.61)	107.54(73.98 to 150.91)	-0.17(-0.45 to 0.21)
Niue	Liver cancer due to hepatitis C	1(1 to 1)	1(0 to 1)	-0.17(-0.39 to 0.16)	41.52(27.34 to 60.09)	34.29(21.61 to 50.4)	-0.17(-0.4 to 0.13)
Niue	Liver cancer due to NASH	0(0 to 1)	0(0 to 1)	0.11(-0.22 to 0.58)	17.2(11.61 to 25.48)	19.16(12.48 to 28.08)	0.11(-0.21 to 0.6)
Niue	Liver cancer due to other causes	0(0 to 0)	0(0 to 0)	-0.27(-0.49 to 0.04)	12.18(8.38 to 17.85)	10.53(6.89 to 15.78)	-0.14(-0.41 to 0.26)
North Macedonia	Liver cancer	4370(3922 to 4775)	6573(5118 to 8447)	0.5(0.15 to 0.96)	222.5(199.42 to 243.14)	202.54(158.3 to 259.74)	-0.09(-0.31 to 0.18)
North Macedonia	Liver cancer due to alcohol use	1537(1118 to 1989)	2460(1642 to 3470)	0.6(0.18 to 1.13)	78.29(57.77 to 100.48)	73.47(49.55 to 103.55)	-0.06(-0.3 to 0.24)
North Macedonia	Liver cancer due to hepatitis B	1314(958 to 1768)	1701(1126 to 2555)	0.29(-0.03 to 0.7)	64.62(46.83 to 86.37)	53.51(35.86 to 78.52)	-0.17(-0.37 to 0.08)
North Macedonia	Liver cancer due to hepatitis C	1006(678 to 1373)	1631(1035 to 2390)	0.62(0.24 to 1.11)	53.05(36.28 to 72.1)	49.98(32.7 to	-0.06(-0.27 to 0.22)

						72.05)	
North Macedonia	Liver cancer due to NASH	283(194 to 396)	527(342 to 790)	0.86(0.39 to 1.48)	14.82(10.3 to 20.64)	16.44(10.87 to 24.22)	0.11(-0.17 to 0.47)
North Macedonia	Liver cancer due to other causes	230(175 to 298)	254(168 to 382)	0.11(-0.19 to 0.48)	11.73(9.07 to 15.05)	9.13(6.34 to 12.97)	-0.22(-0.41 to 0.02)
Northern Mariana Islands	Liver cancer	51(36 to 66)	128(101 to 164)	1.54(0.79 to 2.64)	186.24(143.04 to 234.85)	210.02(169.68 to 261.41)	0.13(-0.17 to 0.51)
Northern Mariana Islands	Liver cancer due to alcohol use	5(3 to 7)	15(9 to 24)	2.32(1.23 to 3.91)	19.8(12.02 to 30.19)	24.63(15.39 to 37.54)	0.24(-0.08 to 0.68)
Northern Mariana Islands	Liver cancer due to hepatitis B	33(22 to 45)	80(59 to 108)	1.43(0.67 to 2.64)	107(76.19 to 142.94)	126.9(95.04 to 167.04)	0.19(-0.15 to 0.65)
Northern Mariana Islands	Liver cancer due to hepatitis C	6(4 to 9)	18(10 to 27)	1.93(1.09 to 3.06)	33.72(21.75 to 47.94)	31.58(20.18 to 44.85)	-0.06(-0.27 to 0.2)
Northern Mariana Islands	Liver cancer due to NASH	4(2 to 6)	11(7 to 16)	1.77(0.86 to 2.98)	16.43(10.95 to 23.56)	18.52(12.13 to 27.22)	0.13(-0.16 to 0.5)
Northern Mariana Islands	Liver cancer due to other causes	3(2 to 4)	5(3 to 7)	0.49(-0.01 to 1.15)	9.28(6.36 to 13.39)	8.4(5.82 to 11.85)	-0.1(-0.32 to 0.2)
Norway	Liver cancer	2248(2159 to 2328)	4811(4357 to 5437)	1.14(0.93 to 1.45)	38.11(36.81 to 39.34)	56.53(51.36 to 64.17)	0.48(0.34 to 0.69)
Norway	Liver cancer due to alcohol use	710(623 to 802)	1666(1385 to 1977)	1.35(1.08 to 1.75)	11.7(10.27 to 13.25)	19.18(15.93 to 22.8)	0.64(0.45 to 0.93)

Norway	Liver cancer due to hepatitis B	399(339 to 465)	745(605 to 921)	0.87(0.67 to 1.17)	7.49(6.41 to 8.78)	9.76(7.96 to 12)	0.3(0.17 to 0.52)
Norway	Liver cancer due to hepatitis C	835(741 to 935)	1793(1536 to 2083)	1.15(0.94 to 1.41)	13.01(11.49 to 14.58)	19.63(16.72 to 22.86)	0.51(0.36 to 0.7)
Norway	Liver cancer due to NASH	133(112 to 157)	311(257 to 378)	1.34(1.1 to 1.62)	2.12(1.8 to 2.47)	3.55(2.96 to 4.27)	0.68(0.51 to 0.88)
Norway	Liver cancer due to other causes	170(152 to 189)	295(250 to 350)	0.73(0.56 to 0.93)	3.8(3.4 to 4.18)	4.41(3.8 to 5.11)	0.16(0.05 to 0.28)
Oman	Liver cancer	961(684 to 1292)	2692(2074 to 3442)	1.8(0.98 to 3.05)	110.97(77.39 to 149.25)	113.84(93.79 to 139.55)	0.03(-0.28 to 0.51)
Oman	Liver cancer due to alcohol use	78(44 to 125)	247(149 to 392)	2.16(1.13 to 3.83)	10.52(5.91 to 16.8)	11.98(7.39 to 18.73)	0.14(-0.23 to 0.78)
Oman	Liver cancer due to hepatitis B	435(275 to 630)	1289(871 to 1802)	1.96(0.98 to 3.64)	46.74(29.21 to 69.08)	44.46(30.82 to 62.51)	-0.05(-0.35 to 0.47)
Oman	Liver cancer due to hepatitis C	248(152 to 369)	615(400 to 877)	1.49(0.72 to 2.72)	37.07(23.17 to 54.66)	35.17(23.43 to 47.33)	-0.05(-0.33 to 0.4)
Oman	Liver cancer due to NASH	57(33 to 90)	255(171 to 373)	3.5(2.08 to 5.87)	7.66(4.41 to 12.45)	12.64(8.68 to 18.39)	0.65(0.13 to 1.5)
Oman	Liver cancer due to other causes	144(105 to 186)	286(222 to 370)	0.99(0.46 to 1.77)	8.99(6.27 to 12.34)	9.59(7.36 to 12.71)	0.07(-0.19 to 0.45)
Pakistan	Liver cancer	64587(47309 to 84080)	129034(104785 to 157625)	1(0.48 to 1.9)	91.77(63.93 to 122.11)	91.09(73.72 to 112.15)	-0.01(-0.27 to 0.47)
Pakistan	Liver cancer due to alcohol use	8729(5532 to 12276)	18564(13698 to 25145)	1.13(0.48 to 2.36)	14.2(8.94 to 19.97)	14.37(10.7 to 19.28)	0.01(-0.29 to 0.6)
Pakistan	Liver cancer due to hepatitis B	11514(7998 to 15717)	26132(20247 to 33716)	1.27(0.6 to 2.44)	16.43(11.2 to 22.84)	16.69(12.92 to	0.02(-0.29 to 0.55)

						21.62)	
Pakistan	Liver cancer due to hepatitis C	27260(18021 to 37356)	51440(39685 to 66366)	0.89(0.35 to 1.86)	46.2(30.14 to 63.65)	43.65(33.54 to 55.86)	-0.06(-0.31 to 0.42)
Pakistan	Liver cancer due to NASH	4167(2835 to 5771)	10032(7670 to 12842)	1.41(0.7 to 2.65)	6.27(4.16 to 8.78)	7.23(5.6 to 9.42)	0.15(-0.17 to 0.71)
Pakistan	Liver cancer due to other causes	12916(9411 to 19103)	22865(17491 to 30014)	0.77(0.12 to 1.63)	8.68(6.62 to 11.62)	9.14(7.15 to 11.67)	0.05(-0.27 to 0.52)
Palau	Liver cancer	32(22 to 44)	71(52 to 95)	1.25(0.44 to 2.55)	279.31(191.65 to 391.63)	287.38(216.5 to 375.81)	0.03(-0.33 to 0.59)
Palau	Liver cancer due to alcohol use	4(2 to 6)	9(5 to 15)	1.51(0.61 to 3.05)	34.02(18.76 to 56.03)	36.72(22 to 56.31)	0.08(-0.29 to 0.69)
Palau	Liver cancer due to hepatitis B	21(14 to 31)	46(33 to 64)	1.17(0.37 to 2.49)	180.63(117.6 to 258.62)	181.57(130.47 to 246.7)	0.01(-0.36 to 0.58)
Palau	Liver cancer due to hepatitis C	4(2 to 6)	8(5 to 13)	1.28(0.49 to 2.5)	37.32(21.76 to 57.49)	36.74(22.92 to 53.75)	-0.02(-0.34 to 0.48)
Palau	Liver cancer due to NASH	2(1 to 3)	5(3 to 8)	1.91(0.88 to 3.5)	16.52(9.81 to 26.71)	21.93(13.54 to 33.66)	0.33(-0.12 to 1.06)
Palau	Liver cancer due to other causes	1(1 to 2)	2(1 to 4)	0.74(0.11 to 1.57)	10.82(6.73 to 16.75)	10.42(6.76 to 15.57)	-0.04(-0.36 to 0.42)
Palestine	Liver cancer	1912(1383 to 2566)	3818(3194 to 4616)	1(0.43 to 1.8)	201.58(146.24 to 271.12)	145.71(121.09 to 175.8)	-0.28(-0.49 to 0.03)
Palestine	Liver cancer due to alcohol use	148(85 to 237)	328(204 to 496)	1.23(0.54 to 2.32)	16.92(9.77 to 26.48)	13.5(8.3 to 20.23)	-0.2(-0.45 to 0.17)
Palestine	Liver cancer due to hepatitis B	730(490 to 1047)	1565(1160 to 2058)	1.14(0.5 to 2.09)	73.98(49.67 to 107.81)	53.29(39.16 to 70.91)	-0.28(-0.49 to 0.03)

Palestine	Liver cancer due to hepatitis C	645(416 to 923)	1213(833 to 1614)	0.88(0.28 to 1.7)	76.89(50.78 to 109.77)	54.13(38.48 to 71.23)	-0.3(-0.52 to 0.01)
Palestine	Liver cancer due to NASH	159(98 to 242)	347(237 to 493)	1.18(0.49 to 2.16)	17.61(10.65 to 27.24)	14.2(9.58 to 20.72)	-0.19(-0.44 to 0.17)
Palestine	Liver cancer due to other causes	230(153 to 330)	364(273 to 466)	0.58(0.11 to 1.29)	16.18(10.2 to 23.97)	10.59(7.56 to 14.59)	-0.35(-0.55 to -0.06)
Panama	Liver cancer	1393(1284 to 1493)	2959(2238 to 3838)	1.12(0.6 to 1.82)	85.21(78.19 to 91.96)	71.56(54.02 to 92.85)	-0.16(-0.37 to 0.12)
Panama	Liver cancer due to alcohol use	401(298 to 517)	1006(669 to 1456)	1.51(0.85 to 2.36)	25.88(19.01 to 33.36)	24.36(16.24 to 35.25)	-0.06(-0.3 to 0.26)
Panama	Liver cancer due to hepatitis B	313(236 to 410)	544(351 to 810)	0.74(0.26 to 1.38)	17.93(13.12 to 24.21)	13.01(8.4 to 19.38)	-0.27(-0.48 to -0.01)
Panama	Liver cancer due to hepatitis C	421(310 to 540)	921(617 to 1324)	1.19(0.65 to 1.9)	28.19(20.89 to 36.31)	22.34(14.92 to 32.18)	-0.21(-0.4 to 0.05)
Panama	Liver cancer due to NASH	85(61 to 116)	232(149 to 345)	1.73(1.01 to 2.67)	5.24(3.71 to 7.35)	5.61(3.59 to 8.34)	0.07(-0.22 to 0.43)
Panama	Liver cancer due to other causes	173(142 to 209)	256(183 to 351)	0.48(0.12 to 0.97)	7.96(6.4 to 9.95)	6.22(4.45 to 8.48)	-0.22(-0.41 to 0.02)
Papua New Guinea	Liver cancer	734(571 to 938)	1906(1451 to 2507)	1.6(0.89 to 2.58)	34.44(26.79 to 44.22)	36.8(28.2 to 47.91)	0.07(-0.23 to 0.47)
Papua New Guinea	Liver cancer due to alcohol use	77(45 to 122)	220(129 to 358)	1.86(0.97 to 3.19)	4.17(2.47 to 6.57)	4.91(2.95 to 8.02)	0.18(-0.19 to 0.71)
Papua New Guinea	Liver cancer due to hepatitis B	371(267 to 512)	949(660 to 1333)	1.56(0.78 to 2.62)	17.11(12.14 to 23.8)	17.55(12.17 to 24.48)	0.03(-0.28 to 0.44)

Papua New Guinea	Liver cancer due to hepatitis C	138(86 to 204)	357(221 to 550)	1.59(0.85 to 2.64)	8.52(5.4 to 12.24)	9.05(5.8 to 13.35)	0.06(-0.24 to 0.45)
Papua New Guinea	Liver cancer due to NASH	37(23 to 57)	110(70 to 166)	2.01(1.21 to 3.15)	2.02(1.27 to 3.15)	2.49(1.59 to 3.78)	0.23(-0.11 to 0.68)
Papua New Guinea	Liver cancer due to other causes	112(82 to 149)	270(200 to 364)	1.42(0.78 to 2.26)	2.62(1.98 to 3.42)	2.8(2.06 to 3.82)	0.07(-0.19 to 0.4)
Paraguay	Liver cancer	1454(1267 to 1650)	2788(2080 to 3682)	0.92(0.4 to 1.63)	58.86(51.02 to 67.49)	49.05(36.64 to 64.71)	-0.17(-0.39 to 0.14)
Paraguay	Liver cancer due to alcohol use	451(316 to 589)	929(592 to 1343)	1.06(0.44 to 1.91)	19.82(13.88 to 25.91)	16.56(10.56 to 23.99)	-0.16(-0.41 to 0.17)
Paraguay	Liver cancer due to hepatitis B	295(215 to 405)	589(367 to 888)	1(0.4 to 1.85)	11.51(8.14 to 16.03)	9.83(6.14 to 14.83)	-0.15(-0.4 to 0.22)
Paraguay	Liver cancer due to hepatitis C	429(308 to 556)	891(589 to 1274)	1.08(0.51 to 1.84)	19.56(13.95 to 25.38)	16.36(10.85 to 22.99)	-0.16(-0.39 to 0.13)
Paraguay	Liver cancer due to NASH	64(45 to 91)	144(91 to 219)	1.24(0.6 to 2.12)	2.74(1.88 to 3.94)	2.56(1.61 to 3.87)	-0.07(-0.32 to 0.29)
Paraguay	Liver cancer due to other causes	215(174 to 281)	236(160 to 355)	0.1(-0.25 to 0.5)	5.22(4.11 to 6.68)	3.73(2.51 to 5.53)	-0.29(-0.5 to -0.01)
Peru	Liver cancer	21147(18234 to 24374)	21307(15616 to 28226)	0.01(-0.3 to 0.42)	154.92(132.8 to 178.43)	65.78(48.15 to 86.91)	-0.58(-0.7 to -0.4)
Peru	Liver cancer due to alcohol use	5496(3833 to 7482)	6456(4151 to 9358)	0.17(-0.18 to 0.65)	45.45(31.48 to 61.68)	20.29(13 to 29.21)	-0.55(-0.69 to -0.38)
Peru	Liver cancer due to hepatitis B	10480(8325 to 12949)	9587(6494 to 13371)	-0.09(-0.37 to 0.29)	75.59(59.09 to 95.03)	29.1(19.72 to 40.7)	-0.62(-0.73 to -0.45)

Peru	Liver cancer due to hepatitis C	1026(628 to 1575)	1216(719 to 1996)	0.19(-0.18 to 0.69)	8.86(5.36 to 13.62)	3.83(2.24 to 6.36)	-0.57(-0.7 to -0.39)
Peru	Liver cancer due to NASH	1271(886 to 1809)	1726(1076 to 2538)	0.36(-0.07 to 0.98)	10.16(6.96 to 14.58)	5.38(3.35 to 7.91)	-0.47(-0.64 to -0.23)
Peru	Liver cancer due to other causes	2874(2295 to 3551)	2322(1578 to 3298)	-0.19(-0.44 to 0.17)	14.87(11.44 to 18.98)	7.18(4.87 to 10.2)	-0.52(-0.66 to -0.3)
Philippines	Liver cancer	103486(79199 to 125076)	162553(130421 to 200450)	0.57(0.14 to 1.22)	276.14(207.37 to 336.12)	179.84(145.63 to 219.79)	-0.35(-0.53 to -0.06)
Philippines	Liver cancer due to alcohol use	19225(13166 to 25295)	35341(26042 to 46825)	0.84(0.29 to 1.8)	56.62(38.39 to 74.69)	40.67(30.17 to 53.48)	-0.28(-0.5 to 0.1)
Philippines	Liver cancer due to hepatitis B	58084(43764 to 71889)	82147(64367 to 103206)	0.41(0.02 to 1.03)	144.22(106.01 to 181.64)	85.65(67.28 to 107.74)	-0.41(-0.58 to -0.13)
Philippines	Liver cancer due to hepatitis C	14129(10381 to 18361)	25798(20152 to 32607)	0.83(0.37 to 1.61)	47.29(34.55 to 60.51)	32.64(25.89 to 41.24)	-0.31(-0.48 to -0.02)
Philippines	Liver cancer due to NASH	5765(4438 to 7304)	11185(8589 to 14180)	0.94(0.45 to 1.7)	16.72(12.54 to 21.45)	13.11(10.34 to 16.43)	-0.22(-0.41 to 0.1)
Philippines	Liver cancer due to other causes	6283(5247 to 7374)	8083(6683 to 9648)	0.29(0.05 to 0.58)	11.29(9.3 to 13.48)	7.78(6.42 to 9.35)	-0.31(-0.44 to -0.14)
Poland	Liver cancer	73470(71248 to 75481)	30145(25069 to 36234)	-0.59(-0.66 to -0.51)	168.87(163.59 to 173.47)	45.91(38.3 to 54.92)	-0.73(-0.77 to -0.67)
Poland	Liver cancer due to alcohol use	26787(23604 to 30050)	13766(11081 to 17051)	-0.49(-0.58 to -0.36)	60.65(53.62 to 67.98)	20.27(16.29 to 25.14)	-0.67(-0.73 to -0.59)
Poland	Liver cancer due to hepatitis B	19324(16629 to 22461)	6312(4926 to 8121)	-0.67(-0.73 to -0.6)	44.63(38.6 to 51.5)	10.21(7.99 to 13.04)	-0.77(-0.81 to -0.72)

Poland	Liver cancer due to hepatitis C	18509(15952 to 21154)	6657(5241 to 8148)	-0.64(-0.7 to -0.57)	42.45(36.67 to 48.16)	9.45(7.47 to 11.56)	-0.78(-0.81 to -0.74)
Poland	Liver cancer due to NASH	5303(4527 to 6177)	2280(1806 to 2883)	-0.57(-0.64 to -0.49)	12.24(10.47 to 14.19)	3.36(2.67 to 4.21)	-0.73(-0.77 to -0.67)
Poland	Liver cancer due to other causes	3547(3114 to 4005)	1130(929 to 1358)	-0.68(-0.73 to -0.63)	8.9(7.86 to 9.94)	2.62(2.17 to 3.17)	-0.71(-0.75 to -0.66)
Portugal	Liver cancer	6296(5982 to 6653)	22220(20359 to 24242)	2.53(2.18 to 2.9)	47.85(45.5 to 50.54)	112.18(102.14 to 122.47)	1.34(1.1 to 1.59)
Portugal	Liver cancer due to alcohol use	2419(1831 to 3000)	9060(6714 to 11472)	2.75(2.3 to 3.26)	17.63(13.4 to 21.65)	45.19(33.33 to 57.69)	1.56(1.24 to 1.92)
Portugal	Liver cancer due to hepatitis B	1030(734 to 1434)	3281(2217 to 4809)	2.19(1.74 to 2.64)	8.25(5.99 to 11.3)	18.92(13.09 to 27.06)	1.29(0.98 to 1.62)
Portugal	Liver cancer due to hepatitis C	2047(1522 to 2668)	7536(5439 to 9828)	2.68(2.29 to 3.1)	14.78(11.12 to 19.07)	34.82(24.71 to 46.34)	1.36(1.1 to 1.64)
Portugal	Liver cancer due to NASH	319(223 to 451)	1296(883 to 1876)	3.07(2.49 to 3.7)	2.36(1.7 to 3.27)	6.23(4.36 to 8.82)	1.64(1.26 to 2.08)
Portugal	Liver cancer due to other causes	482(390 to 602)	1047(741 to 1461)	1.17(0.75 to 1.63)	4.83(4.04 to 5.78)	7.02(5.35 to 9.13)	0.45(0.19 to 0.73)
Puerto Rico	Liver cancer	5767(5413 to 6099)	4030(2963 to 5281)	-0.3(-0.48 to -0.09)	158.53(149.03 to 167.77)	64.29(47.29 to 84.7)	-0.59(-0.7 to -0.46)
Puerto Rico	Liver cancer due to alcohol use	1988(1465 to 2529)	1422(912 to 2063)	-0.28(-0.49 to -0.04)	54.27(39.99 to 69.24)	21.92(14.19 to 31.82)	-0.6(-0.71 to -0.46)
Puerto Rico	Liver cancer due to hepatitis B	1728(1297 to 2264)	1064(678 to 1579)	-0.38(-0.55 to -0.18)	48.1(36.26 to 63.01)	18.64(12.04 to 27.45)	-0.61(-0.72 to -0.49)

Puerto Rico	Liver cancer due to hepatitis C	1134(759 to 1536)	826(519 to 1233)	-0.27(-0.45 to -0.06)	30.99(20.79 to 41.68)	11.83(7.37 to 17.87)	-0.62(-0.71 to -0.51)
Puerto Rico	Liver cancer due to NASH	535(380 to 735)	498(319 to 749)	-0.07(-0.32 to 0.24)	14.56(10.37 to 19.9)	7.47(4.73 to 11.35)	-0.49(-0.63 to -0.31)
Puerto Rico	Liver cancer due to other causes	382(289 to 503)	220(144 to 321)	-0.42(-0.57 to -0.24)	10.61(8.02 to 14.04)	4.44(3.1 to 6.27)	-0.58(-0.69 to -0.45)
Qatar	Liver cancer	428(319 to 576)	2663(1844 to 3661)	5.22(2.98 to 8.67)	361.9(274.31 to 471.5)	287.67(206.42 to 382.88)	-0.21(-0.47 to 0.16)
Qatar	Liver cancer due to alcohol use	42(23 to 66)	288(157 to 470)	5.92(3.13 to 10.3)	40.05(22.99 to 64.72)	33.78(18.73 to 54.41)	-0.16(-0.47 to 0.33)
Qatar	Liver cancer due to hepatitis B	200(134 to 292)	1213(766 to 1832)	5.06(2.73 to 8.91)	131.37(88.01 to 193.85)	93.09(58.13 to 145.28)	-0.29(-0.55 to 0.08)
Qatar	Liver cancer due to hepatitis C	108(69 to 163)	668(394 to 1005)	5.17(3.03 to 8.36)	126.48(80.99 to 181.1)	102.35(66.43 to 145.97)	-0.19(-0.44 to 0.16)
Qatar	Liver cancer due to NASH	41(26 to 62)	323(189 to 513)	6.8(4.08 to 10.96)	42.84(26.42 to 66.02)	43.07(26.62 to 67.52)	0.01(-0.32 to 0.47)
Qatar	Liver cancer due to other causes	36(26 to 51)	172(109 to 263)	3.73(2.11 to 6.2)	21.14(13.77 to 31.04)	15.4(9.5 to 24.01)	-0.27(-0.5 to 0.05)
Republic of Korea	Liver cancer	105415(86559 to 129055)	348014(309268 to 389220)	2.3(1.57 to 3.26)	297.39(245.74 to 363.22)	390.81(348.58 to 435.93)	0.31(0.03 to 0.68)
Republic of Korea	Liver cancer due to alcohol use	12391(7827 to 18905)	59641(39874 to 83711)	3.81(2.55 to 5.38)	36.87(23.55 to 54.84)	65.8(44.69 to 91.92)	0.78(0.36 to 1.33)
Republic of Korea	Liver cancer due to hepatitis B	73452(58539 to 92187)	210262(172834 to 249480)	1.86(1.17 to 2.75)	197.86(157.98 to 246.78)	237.05(196.11 to 279.13)	0.2(-0.08 to 0.57)

Republic of Korea	Liver cancer due to hepatitis C	11716(7555 to 16935)	50729(34099 to 68972)	3.33(2.2 to 4.83)	39.3(26.15 to 55.55)	56(38.15 to 75.45)	0.43(0.09 to 0.85)
Republic of Korea	Liver cancer due to NASH	3844(2531 to 5543)	16527(11292 to 24141)	3.3(2.16 to 4.91)	12.17(8.06 to 17.43)	18.39(12.66 to 26.78)	0.51(0.14 to 0.99)
Republic of Korea	Liver cancer due to other causes	4013(2820 to 5635)	10855(7191 to 15551)	1.71(0.95 to 2.67)	11.19(7.85 to 15.54)	13.56(9.56 to 18.57)	0.21(-0.08 to 0.57)
Republic of Moldova	Liver cancer	2039(1878 to 2205)	3162(2669 to 3748)	0.55(0.32 to 0.81)	44.65(41.14 to 48.33)	56.99(48.51 to 67.46)	0.28(0.09 to 0.48)
Republic of Moldova	Liver cancer due to alcohol use	968(797 to 1130)	1549(1214 to 1952)	0.6(0.33 to 0.92)	21.01(17.43 to 24.4)	27.03(21.11 to 33.89)	0.29(0.07 to 0.55)
Republic of Moldova	Liver cancer due to hepatitis B	443(327 to 595)	652(450 to 936)	0.47(0.2 to 0.82)	9.54(7.06 to 12.77)	12.12(8.55 to 16.94)	0.27(0.04 to 0.55)
Republic of Moldova	Liver cancer due to hepatitis C	345(234 to 478)	649(432 to 939)	0.88(0.55 to 1.23)	7.69(5.3 to 10.49)	11.13(7.44 to 15.86)	0.45(0.2 to 0.72)
Republic of Moldova	Liver cancer due to NASH	88(62 to 124)	192(133 to 280)	1.19(0.74 to 1.74)	1.96(1.41 to 2.73)	3.44(2.4 to 4.96)	0.75(0.41 to 1.17)
Republic of Moldova	Liver cancer due to other causes	195(110 to 277)	120(90 to 157)	-0.39(-0.62 to 0.1)	4.45(2.49 to 6.33)	3.26(2.57 to 4.02)	-0.27(-0.54 to 0.33)
Romania	Liver cancer	12798(11425 to 14441)	25403(20515 to 31171)	0.98(0.57 to 1.42)	46.08(41.35 to 51.57)	77.58(62.72 to 95.66)	0.68(0.33 to 1.07)
Romania	Liver cancer due to alcohol use	5048(3738 to 6367)	11499(8099 to 15132)	1.28(0.77 to 1.86)	17.61(13.25 to 22.14)	33.93(23.84 to 44.8)	0.93(0.51 to 1.43)
Romania	Liver cancer due to hepatitis B	3488(2514 to 4703)	5625(3799 to 8185)	0.61(0.25 to 1.04)	12.6(9.29 to 16.73)	18.72(12.76 to 26.87)	0.49(0.16 to 0.9)
Romania	Liver cancer due to hepatitis C	2743(1883 to 3779)	5575(3620 to 7901)	1.03(0.61 to 1.51)	9.74(6.83 to 13.2)	15.59(9.99 to	0.6(0.27 to 0.99)

						22.1)	
Romania	Liver cancer due to NASH	728(510 to 1042)	1795(1184 to 2636)	1.47(0.9 to 2.1)	2.63(1.89 to 3.66)	5.28(3.53 to 7.67)	1.01(0.55 to 1.5)
Romania	Liver cancer due to other causes	791(641 to 975)	909(637 to 1272)	0.15(-0.1 to 0.47)	3.5(2.9 to 4.18)	4.05(3.13 to 5.25)	0.16(-0.05 to 0.42)
Russian Federation	Liver cancer	87239(83328 to 92381)	167776(141791 to 203267)	0.92(0.65 to 1.26)	50.37(48.18 to 53.16)	77.7(65.63 to 93.46)	0.54(0.33 to 0.8)
Russian Federation	Liver cancer due to alcohol use	27187(23436 to 31249)	62656(49250 to 80046)	1.3(0.92 to 1.77)	14.69(12.68 to 16.93)	27.54(21.53 to 35.27)	0.88(0.57 to 1.25)
Russian Federation	Liver cancer due to hepatitis B	25508(21834 to 29768)	44070(34086 to 57446)	0.73(0.45 to 1.08)	14.34(12.3 to 16.63)	21.19(16.48 to 27.44)	0.48(0.24 to 0.78)
Russian Federation	Liver cancer due to hepatitis C	18353(15501 to 21424)	38784(31503 to 47723)	1.11(0.82 to 1.44)	10.04(8.57 to 11.58)	16.51(13.38 to 20.2)	0.64(0.42 to 0.9)
Russian Federation	Liver cancer due to NASH	5278(4503 to 6228)	12717(10264 to 15751)	1.41(1.08 to 1.8)	2.96(2.55 to 3.48)	5.72(4.66 to 7.04)	0.93(0.66 to 1.25)
Russian Federation	Liver cancer due to other causes	10914(9796 to 12649)	9549(7141 to 12565)	-0.13(-0.3 to 0.06)	8.35(7.53 to 9.77)	6.74(4.75 to 9.65)	-0.19(-0.39 to 0.05)
Rwanda	Liver cancer	5418(3814 to 7857)	9081(6633 to 12203)	0.68(-0.04 to 1.7)	141.83(102.15 to 198.69)	120.17(91.34 to 157.75)	-0.15(-0.47 to 0.31)
Rwanda	Liver cancer due to alcohol use	1317(813 to 2049)	2358(1537 to 3518)	0.79(0.08 to 1.9)	42.07(26.36 to 64.34)	35.39(23.4 to 51.94)	-0.16(-0.47 to 0.32)
Rwanda	Liver cancer due to hepatitis B	1611(1028 to 2441)	2814(1791 to 4144)	0.75(-0.04 to 1.95)	42.89(27.81 to 65.45)	34.29(22.07 to 49.94)	-0.2(-0.54 to 0.31)
Rwanda	Liver cancer due to hepatitis C	785(447 to 1281)	1414(900 to 2074)	0.8(0.13 to 1.88)	27.1(15.93 to 42.78)	23.73(15.81 to	-0.12(-0.42 to 0.33)

						33.81)	
Rwanda	Liver cancer due to NASH	440(262 to 710)	876(550 to 1308)	0.99(0.16 to 2.33)	13.5(8.09 to 21.5)	12.9(8.29 to 18.7)	-0.04(-0.4 to 0.5)
Rwanda	Liver cancer due to other causes	1264(714 to 2147)	1619(982 to 2556)	0.28(-0.47 to 1.59)	16.27(9.92 to 26.13)	13.86(8.98 to 20.8)	-0.15(-0.58 to 0.54)
Saint Kitts and Nevis	Liver cancer	118(107 to 130)	64(50 to 78)	-0.46(-0.58 to -0.32)	327.88(296.71 to 362.83)	90.6(72.18 to 109.76)	-0.72(-0.78 to -0.65)
Saint Kitts and Nevis	Liver cancer due to alcohol use	36(26 to 47)	21(15 to 30)	-0.4(-0.54 to -0.23)	98.1(70.7 to 129.24)	30.31(21.16 to 42.14)	-0.69(-0.75 to -0.62)
Saint Kitts and Nevis	Liver cancer due to hepatitis B	37(28 to 48)	21(14 to 30)	-0.44(-0.6 to -0.26)	111.27(85.43 to 143.11)	27.69(18.32 to 38.97)	-0.75(-0.82 to -0.67)
Saint Kitts and Nevis	Liver cancer due to hepatitis C	25(17 to 34)	12(7 to 18)	-0.53(-0.64 to -0.41)	67.1(46.55 to 90.06)	17.87(11.67 to 25.93)	-0.73(-0.78 to -0.67)
Saint Kitts and Nevis	Liver cancer due to NASH	10(7 to 14)	6(4 to 8)	-0.42(-0.56 to -0.23)	25.7(18.46 to 35.83)	8.33(5.67 to 12.15)	-0.68(-0.75 to -0.59)
Saint Kitts and Nevis	Liver cancer due to other causes	10(7 to 12)	4(3 to 6)	-0.57(-0.7 to -0.41)	25.71(19.06 to 34.03)	6.4(4.21 to 9.02)	-0.75(-0.82 to -0.67)
Saint Lucia	Liver cancer	143(131 to 155)	115(94 to 138)	-0.2(-0.34 to -0.02)	156.27(143.32 to 170.13)	53.59(44.22 to 64.62)	-0.66(-0.72 to -0.58)
Saint Lucia	Liver cancer due to alcohol use	49(37 to 63)	44(31 to 58)	-0.11(-0.28 to 0.12)	54.99(41.23 to 70.51)	20.16(14.61 to 26.6)	-0.63(-0.7 to -0.54)
Saint Lucia	Liver cancer due to hepatitis B	45(35 to 57)	33(24 to 46)	-0.25(-0.4 to -0.08)	48.45(37.09 to 62.95)	15.35(10.9 to 20.96)	-0.68(-0.74 to -0.61)
Saint Lucia	Liver cancer due to hepatitis C	26(18 to 36)	20(13 to 30)	-0.22(-0.36 to -0.05)	29.93(20.76 to 40.79)	9.47(6.18 to	-0.68(-0.74 to -0.61)

						13.68)	
Saint Lucia	Liver cancer due to NASH	10(7 to 13)	9(6 to 14)	-0.02(-0.23 to 0.23)	10.6(7.54 to 14.86)	4.39(2.99 to 6.32)	-0.59(-0.67 to -0.48)
Saint Lucia	Liver cancer due to other causes	14(11 to 17)	8(5 to 11)	-0.44(-0.56 to -0.29)	12.3(9.34 to 16.2)	4.21(3.06 to 5.6)	-0.66(-0.73 to -0.57)
Saint Vincent and the Grenadines	Liver cancer	139(128 to 151)	105(89 to 122)	-0.25(-0.37 to -0.11)	186.24(170.63 to 202.52)	77.04(65.79 to 89.59)	-0.59(-0.65 to -0.51)
Saint Vincent and the Grenadines	Liver cancer due to alcohol use	41(30 to 54)	39(28 to 51)	-0.06(-0.23 to 0.14)	57.23(41.72 to 74)	28.1(20.48 to 36.61)	-0.51(-0.59 to -0.4)
Saint Vincent and the Grenadines	Liver cancer due to hepatitis B	48(37 to 61)	32(23 to 44)	-0.32(-0.44 to -0.17)	63.96(49.13 to 82.8)	23.84(17.3 to 31.85)	-0.63(-0.69 to -0.55)
Saint Vincent and the Grenadines	Liver cancer due to hepatitis C	26(18 to 36)	18(12 to 25)	-0.32(-0.43 to -0.2)	36.86(25.41 to 49.86)	13.17(8.83 to 18.49)	-0.64(-0.7 to -0.58)
Saint Vincent and the Grenadines	Liver cancer due to NASH	10(7 to 13)	8(6 to 12)	-0.12(-0.28 to 0.09)	12.81(9.15 to 17.78)	6.24(4.34 to 8.7)	-0.51(-0.6 to -0.4)
Saint Vincent and the Grenadines	Liver cancer due to other causes	14(11 to 17)	7(5 to 10)	-0.5(-0.6 to -0.37)	15.39(11.9 to 19.45)	5.69(4.15 to 7.57)	-0.63(-0.7 to -0.55)
Samoa	Liver cancer	166(126 to 216)	228(167 to 300)	0.37(-0.06 to 0.93)	169.39(129.92 to 218.89)	139.92(104.25 to 182.01)	-0.17(-0.42 to 0.14)
Samoa	Liver cancer due to alcohol use	19(11 to 31)	27(16 to 42)	0.39(-0.04 to 1.01)	20.97(12.28 to 33.66)	17.32(10.45 to 26.5)	-0.17(-0.42 to 0.17)

Samoa	Liver cancer due to hepatitis B	99(72 to 132)	137(96 to 190)	0.38(-0.1 to 1.01)	99.56(71.69 to 133.68)	81.51(57.38 to 112.33)	-0.18(-0.45 to 0.18)
Samoa	Liver cancer due to hepatitis C	25(15 to 38)	34(21 to 52)	0.38(0 to 0.86)	28.1(17.64 to 42.75)	23.09(14.38 to 34.36)	-0.18(-0.39 to 0.1)
Samoa	Liver cancer due to NASH	11(7 to 17)	17(11 to 26)	0.55(0.06 to 1.22)	12(7.86 to 18.16)	11.15(7.06 to 16.53)	-0.07(-0.35 to 0.32)
Samoa	Liver cancer due to other causes	11(8 to 16)	12(8 to 18)	0.09(-0.29 to 0.62)	8.77(6.11 to 12.31)	6.85(4.38 to 9.95)	-0.22(-0.47 to 0.14)
San Marino	Liver cancer	15(12 to 18)	35(22 to 51)	1.33(0.44 to 2.64)	47.58(39.25 to 57.64)	62.99(40.31 to 93.44)	0.32(-0.18 to 1.1)
San Marino	Liver cancer due to alcohol use	6(4 to 8)	13(8 to 21)	1.4(0.43 to 2.79)	17.01(12.08 to 23.05)	23.63(13.19 to 38.4)	0.39(-0.18 to 1.24)
San Marino	Liver cancer due to hepatitis B	2(2 to 3)	5(3 to 8)	1.11(0.25 to 2.36)	7.67(5.2 to 11.21)	9.88(5.57 to 16.6)	0.29(-0.25 to 1.09)
San Marino	Liver cancer due to hepatitis C	5(3 to 7)	12(7 to 18)	1.43(0.53 to 2.78)	14.8(10.16 to 20.03)	19.65(11.4 to 30.97)	0.33(-0.18 to 1.08)
San Marino	Liver cancer due to NASH	1(1 to 1)	2(1 to 4)	1.72(0.66 to 3.28)	2.6(1.73 to 3.84)	4.02(2.16 to 6.89)	0.55(-0.05 to 1.49)
San Marino	Liver cancer due to other causes	1(1 to 2)	2(1 to 3)	0.77(0.14 to 1.62)	5.5(4.16 to 7.15)	5.8(3.93 to 8.26)	0.06(-0.3 to 0.53)
Sao Tome and Principe	Liver cancer	50(37 to 62)	96(64 to 132)	0.92(0.25 to 1.86)	68.05(50.88 to 83.61)	72.22(48.42 to 97.12)	0.06(-0.31 to 0.55)
Sao Tome and Principe	Liver cancer due to alcohol use	7(5 to 11)	17(10 to 27)	1.34(0.43 to 2.48)	10.76(6.86 to 15.76)	14.28(8.25 to 22.57)	0.33(-0.17 to 0.96)

Sao Tome and Principe	Liver cancer due to hepatitis B	26(18 to 34)	53(33 to 76)	1(0.23 to 2.21)	36.8(25.27 to 47.85)	36.52(22.72 to 52.62)	-0.01(-0.39 to 0.54)
Sao Tome and Principe	Liver cancer due to hepatitis C	7(4 to 9)	11(6 to 16)	0.6(0.09 to 1.31)	10.37(6.86 to 14.4)	10.21(6.12 to 15.91)	-0.02(-0.33 to 0.4)
Sao Tome and Principe	Liver cancer due to NASH	3(2 to 5)	7(5 to 11)	1.27(0.51 to 2.38)	4.9(3.31 to 7.16)	6.28(3.94 to 9.42)	0.28(-0.13 to 0.87)
Sao Tome and Principe	Liver cancer due to other causes	7(5 to 9)	9(6 to 12)	0.27(-0.18 to 1.01)	5.23(3.72 to 6.95)	4.93(3.38 to 6.89)	-0.06(-0.35 to 0.43)
Saudi Arabia	Liver cancer	9381(6631 to 12437)	21129(15693 to 28447)	1.25(0.41 to 2.57)	138.86(98.04 to 182.98)	107.93(82.84 to 140.16)	-0.22(-0.49 to 0.22)
Saudi Arabia	Liver cancer due to alcohol use	650(369 to 1035)	1569(880 to 2566)	1.41(0.47 to 2.99)	10.81(6.16 to 17.66)	9.02(5.29 to 14.78)	-0.17(-0.48 to 0.35)
Saudi Arabia	Liver cancer due to hepatitis B	4426(2882 to 6234)	9288(6180 to 13367)	1.1(0.28 to 2.56)	60.23(38.87 to 85.56)	39.14(26.22 to 57.01)	-0.35(-0.6 to 0.07)
Saudi Arabia	Liver cancer due to hepatitis C	2621(1654 to 3861)	6173(3945 to 8845)	1.36(0.46 to 2.65)	46.46(29.37 to 66.88)	38.37(25.54 to 53.55)	-0.17(-0.46 to 0.26)
Saudi Arabia	Liver cancer due to NASH	808(483 to 1245)	2771(1811 to 4169)	2.43(1.16 to 4.5)	13.2(7.85 to 20.56)	15.62(10.4 to 23.87)	0.18(-0.23 to 0.88)
Saudi Arabia	Liver cancer due to other causes	876(627 to 1177)	1328(856 to 2006)	0.52(0.02 to 1.24)	8.15(5.51 to 11.56)	5.78(3.73 to 8.7)	-0.29(-0.52 to 0.05)
Senegal	Liver cancer	2555(1985 to 3052)	5087(3705 to 6566)	0.99(0.37 to 1.8)	61.04(47.92 to 72.51)	55.68(40.9 to 71.47)	-0.09(-0.35 to 0.26)
Senegal	Liver cancer due to alcohol use	254(157 to 375)	535(321 to 809)	1.11(0.41 to 2.01)	7.51(4.79 to 11.12)	6.82(4.13 to 10.3)	-0.09(-0.38 to 0.29)

Senegal	Liver cancer due to hepatitis B	1424(1101 to 1761)	2964(2060 to 3991)	1.08(0.41 to 1.94)	36.97(28.47 to 45.99)	32.7(22.89 to 44.12)	-0.12(-0.4 to 0.24)
Senegal	Liver cancer due to hepatitis C	149(87 to 223)	320(188 to 486)	1.15(0.51 to 2.08)	4.72(2.83 to 7.01)	4.39(2.56 to 6.81)	-0.07(-0.35 to 0.29)
Senegal	Liver cancer due to NASH	195(132 to 274)	492(308 to 710)	1.52(0.73 to 2.68)	5.66(3.79 to 8.02)	6.18(3.93 to 9.04)	0.09(-0.24 to 0.55)
Senegal	Liver cancer due to other causes	534(364 to 770)	777(493 to 1135)	0.46(-0.16 to 1.38)	6.18(4.34 to 8.52)	5.58(3.67 to 7.92)	-0.1(-0.42 to 0.32)
Serbia	Liver cancer	17796(15003 to 20597)	19076(14945 to 24381)	0.07(-0.2 to 0.42)	152.09(129.46 to 174.88)	125.07(97.12 to 160.47)	-0.18(-0.38 to 0.09)
Serbia	Liver cancer due to alcohol use	6324(4459 to 8236)	7347(4896 to 10257)	0.16(-0.15 to 0.56)	52.67(37.88 to 67.86)	46.91(31.56 to 65.83)	-0.11(-0.34 to 0.2)
Serbia	Liver cancer due to hepatitis B	4726(3311 to 6531)	3958(2495 to 6131)	-0.16(-0.38 to 0.14)	39.84(28.73 to 53.59)	28.37(18.38 to 43.17)	-0.29(-0.47 to -0.05)
Serbia	Liver cancer due to hepatitis C	5101(3419 to 7153)	5931(3937 to 8402)	0.16(-0.15 to 0.59)	44.21(30.78 to 60.56)	37.09(24.75 to 52.77)	-0.16(-0.37 to 0.11)
Serbia	Liver cancer due to NASH	949(622 to 1389)	1276(814 to 1933)	0.34(-0.03 to 0.85)	8.45(5.65 to 12.2)	8.19(5.3 to 12.16)	-0.03(-0.28 to 0.3)
Serbia	Liver cancer due to other causes	697(513 to 951)	564(358 to 835)	-0.19(-0.41 to 0.08)	6.93(5.33 to 8.89)	4.5(3.08 to 6.3)	-0.35(-0.52 to -0.14)
Seychelles	Liver cancer	117(102 to 135)	168(139 to 204)	0.43(0.13 to 0.79)	208.36(180.35 to 242)	142.83(118.89 to 170.14)	-0.31(-0.45 to -0.15)
Seychelles	Liver cancer due to alcohol use	24(15 to 34)	42(27 to 61)	0.79(0.37 to 1.32)	42.17(27.41 to 61.25)	35.9(23.45 to 51.18)	-0.15(-0.35 to 0.09)

Seychelles	Liver cancer due to hepatitis B	55(42 to 71)	73(54 to 99)	0.34(0.03 to 0.74)	97.73(74.47 to 127.17)	59.49(43.85 to 79.37)	-0.39(-0.53 to -0.21)
Seychelles	Liver cancer due to hepatitis C	25(17 to 34)	31(21 to 45)	0.27(0.04 to 0.57)	44(29.61 to 60.56)	28.45(19.08 to 39.69)	-0.35(-0.47 to -0.21)
Seychelles	Liver cancer due to NASH	10(7 to 14)	15(11 to 22)	0.59(0.26 to 1)	17.14(11.88 to 24.06)	13.68(9.44 to 19.6)	-0.2(-0.38 to -0.01)
Seychelles	Liver cancer due to other causes	5(3 to 6)	6(4 to 8)	0.23(-0.03 to 0.5)	7.32(5.24 to 9.92)	5.31(3.81 to 7.13)	-0.27(-0.42 to -0.1)
Sierra Leone	Liver cancer	3598(2662 to 4697)	5640(4080 to 7624)	0.57(0.07 to 1.26)	159.56(118.15 to 207.5)	124.54(91.76 to 167.01)	-0.22(-0.46 to 0.11)
Sierra Leone	Liver cancer due to alcohol use	544(343 to 851)	823(486 to 1257)	0.51(0.01 to 1.19)	27.45(17.43 to 42.68)	21.51(12.83 to 32.75)	-0.22(-0.47 to 0.14)
Sierra Leone	Liver cancer due to hepatitis B	1813(1221 to 2475)	2858(1935 to 4024)	0.58(0.04 to 1.34)	84.05(56.47 to 114.46)	61.64(42.38 to 88.35)	-0.27(-0.51 to 0.08)
Sierra Leone	Liver cancer due to hepatitis C	471(291 to 716)	727(426 to 1058)	0.54(0.07 to 1.26)	24.86(15.53 to 37.5)	20.6(12.53 to 29.58)	-0.17(-0.41 to 0.19)
Sierra Leone	Liver cancer due to NASH	209(134 to 312)	412(267 to 627)	0.98(0.35 to 1.86)	10.34(6.57 to 15.48)	10.31(6.62 to 15.68)	0(-0.31 to 0.41)
Sierra Leone	Liver cancer due to other causes	561(342 to 927)	819(514 to 1245)	0.46(-0.12 to 1.46)	12.87(8.59 to 19.19)	10.47(6.9 to 15.53)	-0.19(-0.47 to 0.22)
Singapore	Liver cancer	4856(4569 to 5165)	14221(12717 to 15846)	1.93(1.64 to 2.26)	200.22(188.31 to 212.56)	180.04(160.92 to 200.58)	-0.1(-0.19 to 0)
Singapore	Liver cancer due to alcohol use	330(214 to 482)	1127(702 to 1693)	2.41(1.82 to 3.08)	14.17(9.17 to 21.04)	14.1(8.85 to 21.19)	-0.01(-0.16 to 0.18)

Singapore	Liver cancer due to hepatitis B	3305(2880 to 3738)	8575(6925 to 10338)	1.59(1.26 to 1.94)	130.65(112.19 to 149.68)	106.41(85.57 to 128.25)	-0.19(-0.28 to -0.08)
Singapore	Liver cancer due to hepatitis C	895(595 to 1235)	3511(2299 to 4892)	2.93(2.39 to 3.5)	41.33(27.81 to 56.94)	45.52(30.07 to 63.06)	0.1(-0.04 to 0.26)
Singapore	Liver cancer due to NASH	148(98 to 221)	601(389 to 882)	3.06(2.38 to 3.86)	6.65(4.4 to 10.03)	7.85(5.13 to 11.6)	0.18(0 to 0.4)
Singapore	Liver cancer due to other causes	178(137 to 227)	408(265 to 589)	1.29(0.83 to 1.79)	7.41(5.67 to 9.59)	6.15(4.3 to 8.43)	-0.17(-0.3 to -0.02)
Slovakia	Liver cancer	7030(6573 to 7591)	7161(5509 to 9175)	0.02(-0.23 to 0.33)	118.81(111.05 to 128.06)	81.09(61.95 to 103.54)	-0.32(-0.48 to -0.11)
Slovakia	Liver cancer due to alcohol use	3073(2402 to 3716)	3293(2240 to 4567)	0.07(-0.21 to 0.43)	51.4(39.83 to 62.13)	36.09(24.73 to 49.75)	-0.3(-0.48 to -0.06)
Slovakia	Liver cancer due to hepatitis B	1863(1384 to 2483)	1581(1007 to 2362)	-0.15(-0.39 to 0.13)	31.96(24.06 to 42.22)	18.86(12.28 to 27.62)	-0.41(-0.57 to -0.2)
Slovakia	Liver cancer due to hepatitis C	1384(941 to 1898)	1527(974 to 2284)	0.1(-0.17 to 0.44)	23(15.65 to 31.52)	16.53(10.6 to 24.48)	-0.28(-0.45 to -0.06)
Slovakia	Liver cancer due to NASH	402(279 to 564)	501(322 to 753)	0.25(-0.07 to 0.66)	6.72(4.68 to 9.41)	5.62(3.67 to 8.29)	-0.16(-0.38 to 0.11)
Slovakia	Liver cancer due to other causes	308(239 to 396)	258(169 to 370)	-0.16(-0.37 to 0.09)	5.72(4.55 to 7.17)	3.99(2.83 to 5.42)	-0.3(-0.47 to -0.09)
Slovenia	Liver cancer	1776(1341 to 2286)	4608(3489 to 5980)	1.59(0.75 to 2.75)	73.22(55.42 to 94.41)	117.24(88.45 to 153.02)	0.6(0.08 to 1.33)
Slovenia	Liver cancer due to alcohol use	693(465 to 959)	1887(1249 to 2689)	1.72(0.82 to 3.03)	28.25(19.05 to 39.31)	47.29(30.94 to 67.31)	0.67(0.12 to 1.48)

Slovenia	Liver cancer due to hepatitis B	494(326 to 739)	1092(680 to 1636)	1.21(0.44 to 2.3)	20.48(13.57 to 30.56)	30.04(18.93 to 44.57)	0.47(-0.04 to 1.17)
Slovenia	Liver cancer due to hepatitis C	398(255 to 594)	1104(698 to 1618)	1.77(0.87 to 2.93)	16.12(10.37 to 24.01)	26.14(16.27 to 38.9)	0.62(0.1 to 1.34)
Slovenia	Liver cancer due to NASH	115(71 to 173)	378(238 to 579)	2.29(1.2 to 3.79)	4.7(2.96 to 7.04)	9.21(5.77 to 14.03)	0.96(0.32 to 1.83)
Slovenia	Liver cancer due to other causes	77(54 to 109)	146(92 to 217)	0.91(0.31 to 1.68)	3.67(2.66 to 4.99)	4.56(3.13 to 6.42)	0.24(-0.09 to 0.68)
Solomon Islands	Liver cancer	400(304 to 511)	708(556 to 879)	0.77(0.24 to 1.47)	202.44(151.77 to 261.14)	160.39(126.72 to 197.96)	-0.21(-0.44 to 0.11)
Solomon Islands	Liver cancer due to alcohol use	31(18 to 49)	58(35 to 89)	0.89(0.27 to 1.81)	19.51(11.49 to 31.61)	16.34(9.89 to 24.58)	-0.16(-0.43 to 0.25)
Solomon Islands	Liver cancer due to hepatitis B	230(164 to 303)	390(292 to 513)	0.69(0.17 to 1.42)	117.82(83.55 to 156.43)	86.35(64.67 to 113.42)	-0.27(-0.49 to 0.04)
Solomon Islands	Liver cancer due to hepatitis C	49(28 to 77)	89(55 to 129)	0.82(0.24 to 1.69)	33.3(19.53 to 50.23)	27.6(17.86 to 39.21)	-0.17(-0.41 to 0.19)
Solomon Islands	Liver cancer due to NASH	25(16 to 39)	54(35 to 79)	1.11(0.39 to 2.24)	13.96(8.87 to 21.26)	13.25(8.89 to 19.28)	-0.05(-0.35 to 0.39)
Solomon Islands	Liver cancer due to other causes	64(42 to 96)	117(80 to 159)	0.82(0.09 to 1.78)	17.84(12.2 to 24.86)	16.84(11.94 to 22.14)	-0.06(-0.35 to 0.33)
Somalia	Liver cancer	3573(2115 to 6146)	8797(5678 to 15164)	1.46(0.66 to 2.69)	99.19(60.26 to 171.88)	92.82(59.85 to 163.59)	-0.06(-0.35 to 0.38)
Somalia	Liver cancer due to alcohol use	561(284 to 1215)	1307(696 to 2600)	1.33(0.56 to 2.58)	20.07(10.36 to 43.66)	17.85(9.46 to 33.94)	-0.11(-0.38 to 0.3)

Somalia	Liver cancer due to hepatitis B	1334(738 to 2555)	3234(1883 to 6309)	1.42(0.62 to 2.74)	36.94(20.37 to 70.93)	33.71(19.46 to 64.61)	-0.09(-0.38 to 0.38)
Somalia	Liver cancer due to hepatitis C	516(264 to 915)	1304(735 to 2361)	1.53(0.68 to 2.76)	20.38(11.01 to 34.72)	20.25(11.69 to 35.59)	-0.01(-0.31 to 0.45)
Somalia	Liver cancer due to NASH	292(154 to 509)	758(446 to 1333)	1.6(0.75 to 3)	10.12(5.51 to 17.37)	10.07(5.85 to 17.69)	0(-0.31 to 0.48)
Somalia	Liver cancer due to other causes	871(457 to 1534)	2194(1296 to 3705)	1.52(0.4 to 3.61)	11.69(6.38 to 19.36)	10.94(6.64 to 17.56)	-0.06(-0.41 to 0.47)
South Africa	Liver cancer	41490(28400 to 71744)	77899(68313 to 88173)	0.88(0.01 to 1.96)	165.06(110.83 to 284.9)	154.72(135.89 to 175.35)	-0.06(-0.49 to 0.49)
South Africa	Liver cancer due to alcohol use	7544(4450 to 14358)	15412(12401 to 18893)	1.04(0 to 2.5)	32.46(19.14 to 62.01)	31.5(25.46 to 38.34)	-0.03(-0.53 to 0.67)
South Africa	Liver cancer due to hepatitis B	18629(12394 to 32643)	34023(28378 to 40602)	0.83(-0.08 to 2.02)	68.47(44.74 to 120.47)	62.97(52.89 to 74.31)	-0.08(-0.54 to 0.52)
South Africa	Liver cancer due to hepatitis C	7961(5274 to 12429)	15418(12906 to 18502)	0.94(0.2 to 1.87)	37.15(24.23 to 58.17)	34.19(28.75 to 40.91)	-0.08(-0.43 to 0.38)
South Africa	Liver cancer due to NASH	3802(2521 to 6229)	8325(6929 to 10006)	1.19(0.26 to 2.34)	15.52(10.05 to 25.44)	17.14(14.37 to 20.57)	0.1(-0.36 to 0.71)
South Africa	Liver cancer due to other causes	3553(2653 to 5360)	4721(3900 to 5669)	0.33(-0.15 to 0.85)	11.46(8.26 to 17.71)	8.92(7.42 to 10.61)	-0.22(-0.51 to 0.1)
South Sudan	Liver cancer	2697(1699 to 4685)	3716(2187 to 6319)	0.38(-0.05 to 1.03)	84.6(52.66 to 155.28)	73.57(42.71 to 128.66)	-0.13(-0.39 to 0.24)
South Sudan	Liver cancer due to alcohol use	460(222 to 1031)	625(288 to 1316)	0.36(-0.09 to 1.02)	18.32(8.9 to 41.27)	15.47(7.25 to 31.94)	-0.16(-0.42 to 0.25)

South Sudan	Liver cancer due to hepatitis B	861(479 to 1691)	1289(652 to 2439)	0.5(-0.02 to 1.31)	29.39(16.07 to 59.21)	25.27(13.1 to 48.02)	-0.14(-0.42 to 0.29)
South Sudan	Liver cancer due to hepatitis C	399(226 to 700)	576(301 to 1007)	0.44(-0.03 to 1.1)	16.98(9.69 to 29.42)	15.32(8.26 to 26.69)	-0.1(-0.38 to 0.26)
South Sudan	Liver cancer due to NASH	231(130 to 400)	366(191 to 653)	0.59(0.06 to 1.36)	8.83(4.89 to 15.53)	8.67(4.55 to 15.28)	-0.02(-0.32 to 0.42)
South Sudan	Liver cancer due to other causes	745(428 to 1232)	859(533 to 1363)	0.15(-0.35 to 0.94)	11.07(6.77 to 17.23)	8.84(5.7 to 13.36)	-0.2(-0.5 to 0.2)
Spain	Liver cancer	48406(46187 to 50420)	97661(88053 to 107340)	1.02(0.82 to 1.23)	91.83(87.81 to 95.56)	116.38(105.03 to 128.56)	0.27(0.14 to 0.41)
Spain	Liver cancer due to alcohol use	15434(10951 to 20068)	28190(19584 to 37626)	0.83(0.57 to 1.1)	28.74(20.58 to 37.37)	33.99(23.72 to 45.46)	0.18(0.02 to 0.37)
Spain	Liver cancer due to hepatitis B	6281(4444 to 8671)	11798(7930 to 16934)	0.88(0.61 to 1.22)	12.76(9.3 to 17.22)	15.8(10.92 to 22.51)	0.24(0.05 to 0.45)
Spain	Liver cancer due to hepatitis C	22297(17564 to 27645)	48902(37655 to 60373)	1.19(0.94 to 1.46)	40.94(32.43 to 50.46)	54.97(41.91 to 68.27)	0.34(0.19 to 0.5)
Spain	Liver cancer due to NASH	2188(1521 to 3189)	5168(3517 to 7574)	1.36(1 to 1.75)	4.07(2.88 to 5.83)	5.93(4.1 to 8.69)	0.46(0.24 to 0.68)
Spain	Liver cancer due to other causes	2205(1677 to 2864)	3603(2512 to 5023)	0.63(0.36 to 0.9)	5.32(4.29 to 6.61)	5.7(4.31 to 7.41)	0.07(-0.08 to 0.23)
Sri Lanka	Liver cancer	6404(5559 to 7282)	17362(12378 to 23522)	1.71(0.88 to 2.79)	53.09(46.39 to 60.25)	67.03(48.23 to 90.38)	0.26(-0.13 to 0.75)
Sri Lanka	Liver cancer due to alcohol use	1124(743 to 1592)	4290(2629 to 6690)	2.82(1.53 to 4.55)	10.04(6.64 to 14.12)	15.99(9.89 to 24.89)	0.59(0.06 to 1.3)
Sri Lanka	Liver cancer due to hepatitis B	3031(2360 to 3806)	7030(4557 to 10373)	1.32(0.58 to 2.29)	23.76(18.28 to 30.2)	26.83(17.58 to	0.13(-0.23 to 0.61)

						38.96)	
Sri Lanka	Liver cancer due to hepatitis C	1172(787 to 1621)	3686(2262 to 5591)	2.15(1.2 to 3.46)	11.35(7.69 to 15.56)	14.42(8.98 to 21.36)	0.27(-0.11 to 0.79)
Sri Lanka	Liver cancer due to NASH	476(336 to 661)	1543(947 to 2424)	2.24(1.19 to 3.61)	4.23(3.01 to 5.92)	6.01(3.75 to 9.39)	0.42(-0.01 to 1)
Sri Lanka	Liver cancer due to other causes	601(442 to 995)	814(544 to 1236)	0.35(-0.1 to 1.01)	3.71(2.75 to 5.84)	3.77(2.54 to 5.81)	0.02(-0.29 to 0.43)
Sudan	Liver cancer	9414(5699 to 13628)	18311(11386 to 28668)	0.95(0.35 to 1.77)	89.22(52.73 to 131.83)	88.53(53.8 to 140.42)	-0.01(-0.31 to 0.46)
Sudan	Liver cancer due to alcohol use	1050(543 to 2009)	2235(1173 to 4203)	1.13(0.38 to 2.48)	11.04(5.62 to 20.82)	12.22(6.34 to 22.95)	0.11(-0.28 to 0.79)
Sudan	Liver cancer due to hepatitis B	3743(2241 to 5830)	7190(3960 to 11708)	0.92(0.23 to 1.96)	35.72(20.93 to 55.72)	32.71(17.61 to 53.17)	-0.08(-0.39 to 0.42)
Sudan	Liver cancer due to hepatitis C	2261(1245 to 3509)	4171(2334 to 6769)	0.84(0.25 to 1.68)	24.95(13.77 to 38.35)	23.67(13.39 to 38.58)	-0.05(-0.36 to 0.36)
Sudan	Liver cancer due to NASH	803(423 to 1293)	2118(1255 to 3423)	1.64(0.76 to 2.88)	8.31(4.38 to 13.51)	11.1(6.43 to 18.26)	0.34(-0.1 to 0.97)
Sudan	Liver cancer due to other causes	1556(898 to 2617)	2597(1726 to 3831)	0.67(0.07 to 1.56)	9.2(5.17 to 13.55)	8.83(5.53 to 13.84)	-0.04(-0.33 to 0.35)
Suriname	Liver cancer	486(423 to 549)	415(329 to 527)	-0.15(-0.33 to 0.09)	172.83(152.58 to 194.38)	67.45(53.77 to 84.97)	-0.61(-0.69 to -0.51)
Suriname	Liver cancer due to alcohol use	146(104 to 192)	140(97 to 199)	-0.05(-0.26 to 0.23)	54.57(38.82 to 71.66)	22.57(15.77 to 31.78)	-0.59(-0.68 to -0.47)
Suriname	Liver cancer due to hepatitis B	171(129 to 222)	132(90 to 187)	-0.23(-0.41 to 0.01)	57.83(43.69 to 75.54)	20.86(14.43 to	-0.64(-0.72 to -0.53)

						29.32)	
Suriname	Liver cancer due to hepatitis C	85(57 to 119)	75(48 to 109)	-0.12(-0.32 to 0.11)	32.99(22.39 to 45.06)	12.44(8.17 to 18.12)	-0.62(-0.71 to -0.53)
Suriname	Liver cancer due to NASH	33(23 to 46)	36(24 to 52)	0.07(-0.18 to 0.4)	12.27(8.63 to 17.13)	5.9(3.99 to 8.5)	-0.52(-0.63 to -0.37)
Suriname	Liver cancer due to other causes	51(38 to 65)	33(24 to 46)	-0.34(-0.51 to -0.11)	15.17(11.37 to 19.79)	5.68(4.05 to 7.7)	-0.63(-0.71 to -0.5)
Sweden	Liver cancer	7830(7445 to 8189)	12390(11461 to 13272)	0.58(0.45 to 0.7)	58.88(56.05 to 61.76)	69.29(63.83 to 74.31)	0.18(0.07 to 0.27)
Sweden	Liver cancer due to alcohol use	3194(2778 to 3623)	5661(4884 to 6459)	0.77(0.59 to 0.96)	23.5(20.58 to 26.64)	31.43(27.05 to 35.84)	0.34(0.19 to 0.48)
Sweden	Liver cancer due to hepatitis B	547(428 to 693)	807(616 to 1048)	0.48(0.32 to 0.62)	4.7(3.72 to 5.92)	5.27(4.12 to 6.81)	0.12(0 to 0.24)
Sweden	Liver cancer due to hepatitis C	2916(2476 to 3408)	4225(3500 to 4954)	0.45(0.33 to 0.58)	20.5(17.3 to 24.08)	21.96(18.13 to 26.2)	0.07(-0.02 to 0.17)
Sweden	Liver cancer due to NASH	557(465 to 671)	905(746 to 1093)	0.62(0.47 to 0.78)	4.04(3.4 to 4.81)	4.86(4.06 to 5.82)	0.2(0.09 to 0.32)
Sweden	Liver cancer due to other causes	616(530 to 708)	793(676 to 925)	0.29(0.17 to 0.4)	6.14(5.44 to 6.91)	5.77(5.01 to 6.62)	-0.06(-0.14 to 0.03)
Switzerland	Liver cancer	5331(5043 to 5596)	15402(13811 to 17165)	1.89(1.57 to 2.26)	55.76(52.8 to 58.58)	98.91(88.39 to 110.57)	0.77(0.57 to 1)
Switzerland	Liver cancer due to alcohol use	2356(1806 to 2838)	6360(4684 to 8151)	1.7(1.3 to 2.13)	24.25(18.6 to 29.37)	40.38(29.73 to 51.94)	0.67(0.42 to 0.94)
Switzerland	Liver cancer due to hepatitis B	807(568 to 1135)	2265(1515 to 3239)	1.81(1.41 to 2.29)	8.96(6.34 to 12.55)	15.95(10.89 to 22.56)	0.78(0.54 to 1.08)

Switzerland	Liver cancer due to hepatitis C	1628(1165 to 2140)	5193(3698 to 6882)	2.19(1.82 to 2.62)	16.18(11.67 to 21.34)	31.38(22.07 to 42.23)	0.94(0.71 to 1.21)
Switzerland	Liver cancer due to NASH	255(173 to 362)	858(579 to 1274)	2.37(1.88 to 2.92)	2.56(1.76 to 3.64)	5.31(3.63 to 7.92)	1.07(0.77 to 1.42)
Switzerland	Liver cancer due to other causes	285(215 to 376)	727(503 to 1020)	1.55(1.13 to 2)	3.82(3.03 to 4.79)	5.89(4.42 to 7.81)	0.54(0.31 to 0.78)
Syrian Arab Republic	Liver cancer	7547(5842 to 9452)	13187(9723 to 17776)	0.75(0.19 to 1.66)	123.3(93.07 to 155.76)	100.49(75.29 to 134.4)	-0.18(-0.45 to 0.24)
Syrian Arab Republic	Liver cancer due to alcohol use	592(356 to 891)	1184(683 to 1920)	1(0.27 to 2.2)	10.95(6.61 to 16.75)	9.09(5.25 to 14.52)	-0.17(-0.47 to 0.33)
Syrian Arab Republic	Liver cancer due to hepatitis B	3311(2384 to 4440)	5364(3604 to 7758)	0.62(0.07 to 1.54)	51.33(36.69 to 71.17)	38.02(25.6 to 54.88)	-0.26(-0.51 to 0.14)
Syrian Arab Republic	Liver cancer due to hepatitis C	1980(1272 to 2845)	3975(2579 to 5807)	1.01(0.33 to 2.11)	38.95(25.35 to 55.7)	32.95(22 to 46.75)	-0.15(-0.43 to 0.3)
Syrian Arab Republic	Liver cancer due to NASH	638(409 to 949)	1442(916 to 2285)	1.26(0.46 to 2.57)	11.38(7.28 to 17.07)	11.49(7.36 to 17.67)	0.01(-0.33 to 0.61)
Syrian Arab Republic	Liver cancer due to other causes	1025(752 to 1389)	1223(842 to 1752)	0.19(-0.2 to 0.77)	10.69(7.56 to 14.98)	8.94(6.25 to 12.76)	-0.16(-0.42 to 0.22)
Taiwan (Province of China)	Liver cancer	62600(60085 to 65333)	66868(51941 to 86722)	0.07(-0.18 to 0.39)	346.27(332.07 to 360.69)	176.69(137.41 to 228.24)	-0.49(-0.61 to -0.34)
Taiwan (Province of China)	Liver cancer due to alcohol use	5692(3691 to 8263)	6181(3620 to 9834)	0.09(-0.2 to 0.49)	32.3(21.08 to 46.9)	15.6(9.31 to 24.62)	-0.52(-0.64 to -0.34)

Taiwan (Province of China)	Liver cancer due to hepatitis B	37962(33003 to 42842)	32747(23656 to 43958)	-0.14(-0.36 to 0.12)	203.67(175.95 to 230.79)	88.51(64.53 to 117.74)	-0.57(-0.67 to -0.44)
Taiwan (Province of China)	Liver cancer due to hepatitis C	12546(9046 to 16496)	19755(13235 to 27598)	0.57(0.18 to 1.05)	74.93(54.94 to 97.01)	49.46(33.22 to 69.09)	-0.34(-0.5 to -0.15)
Taiwan (Province of China)	Liver cancer due to NASH	2823(1991 to 3994)	4850(3106 to 7443)	0.72(0.25 to 1.31)	16.08(11.29 to 22.83)	12.52(8.05 to 18.98)	-0.22(-0.43 to 0.04)
Taiwan (Province of China)	Liver cancer due to other causes	3578(2693 to 4714)	3334(2180 to 4974)	-0.07(-0.33 to 0.23)	19.28(14.52 to 25.81)	10.59(7.46 to 15.1)	-0.45(-0.59 to -0.28)
Tajikistan	Liver cancer	1152(1019 to 1307)	5299(4223 to 6660)	3.6(2.57 to 4.85)	29.22(26.42 to 32.56)	91.86(73.83 to 115.95)	2.14(1.47 to 2.96)
Tajikistan	Liver cancer due to alcohol use	149(99 to 209)	1082(705 to 1602)	6.26(4.39 to 8.75)	5.29(3.51 to 7.37)	20.1(13.12 to 28.6)	2.8(1.9 to 4.01)
Tajikistan	Liver cancer due to hepatitis B	164(120 to 219)	1343(934 to 1852)	7.2(5.14 to 9.88)	5.15(3.66 to 6.99)	18.69(12.98 to 25.97)	2.63(1.73 to 3.7)
Tajikistan	Liver cancer due to hepatitis C	308(237 to 384)	2002(1402 to 2712)	5.5(3.95 to 7.48)	11.17(8.67 to 13.89)	41.44(30.42 to 54.53)	2.71(1.88 to 3.76)
Tajikistan	Liver cancer due to NASH	33(23 to 46)	281(189 to 404)	7.62(5.32 to 10.39)	1.11(0.77 to 1.58)	5.1(3.45 to 7.3)	3.59(2.47 to 4.98)
Tajikistan	Liver cancer due to other causes	498(396 to 626)	591(431 to 1008)	0.19(-0.15 to 1.09)	6.51(5.32 to 7.99)	6.54(4.81 to 10.25)	0.01(-0.27 to 0.64)
Thailand	Liver cancer	239204(205959 to 275829)	632922(460924 to 847115)	1.65(0.87 to 2.69)	575.32(500.14 to 659.92)	615.25(450.05 to 818.04)	0.07(-0.24 to 0.49)

Thailand	Liver cancer due to alcohol use	53637(35761 to 75647)	184807(117524 to 280303)	2.45(1.39 to 3.87)	136.53(92.89 to 188.43)	176.35(112.83 to 265.29)	0.29(-0.1 to 0.85)
Thailand	Liver cancer due to hepatitis B	131273(103715 to 160160)	288508(195941 to 413617)	1.2(0.51 to 2.09)	295.19(232.66 to 363.68)	282.94(194.16 to 400.24)	-0.04(-0.33 to 0.33)
Thailand	Liver cancer due to hepatitis C	30527(20095 to 43126)	87757(53600 to 134944)	1.87(1.04 to 3)	85.83(57.65 to 120.05)	84.85(52.91 to 128.56)	-0.01(-0.29 to 0.36)
Thailand	Liver cancer due to NASH	14337(9960 to 20126)	54634(33797 to 83372)	2.81(1.69 to 4.44)	37.09(25.36 to 52.63)	53.31(33.24 to 80.99)	0.44(0.02 to 1.03)
Thailand	Liver cancer due to other causes	9429(6751 to 12305)	17217(10569 to 26888)	0.83(0.24 to 1.6)	20.68(14.63 to 27.16)	17.81(11.44 to 26.94)	-0.14(-0.37 to 0.19)
Timor-Leste	Liver cancer	772(497 to 1094)	1424(893 to 2016)	0.85(0.1 to 1.87)	205.39(135.52 to 288.52)	161.87(103.41 to 229.54)	-0.21(-0.49 to 0.18)
Timor-Leste	Liver cancer due to alcohol use	113(62 to 188)	269(139 to 442)	1.38(0.5 to 2.73)	34.91(19.36 to 56.86)	31.33(16.43 to 50.86)	-0.1(-0.41 to 0.33)
Timor-Leste	Liver cancer due to hepatitis B	408(253 to 648)	653(341 to 1014)	0.6(-0.11 to 1.52)	97.95(61.01 to 152.84)	72.19(38.27 to 111.82)	-0.26(-0.57 to 0.14)
Timor-Leste	Liver cancer due to hepatitis C	139(78 to 218)	312(188 to 471)	1.24(0.49 to 2.46)	49.09(29.56 to 75.44)	38.69(24.15 to 57.31)	-0.21(-0.46 to 0.14)
Timor-Leste	Liver cancer due to NASH	47(28 to 76)	106(62 to 163)	1.25(0.38 to 2.43)	14.25(8.5 to 22.47)	12.45(7.36 to 18.84)	-0.13(-0.43 to 0.27)
Timor-Leste	Liver cancer due to other causes	64(34 to 115)	83(48 to 117)	0.31(-0.49 to 1.46)	9.19(5.25 to 14.46)	7.21(4.28 to 10.51)	-0.21(-0.61 to 0.29)
Togo	Liver cancer	2867(2272 to 3617)	6085(4495 to 8034)	1.12(0.47 to 1.98)	174.3(137.2 to 223.78)	132.88(100.37 to 173.59)	-0.24(-0.46 to 0.06)

Togo	Liver cancer due to alcohol use	379(231 to 590)	913(551 to 1413)	1.41(0.6 to 2.57)	27.96(17.06 to 43.64)	22.39(13.82 to 33.74)	-0.2(-0.46 to 0.16)
Togo	Liver cancer due to hepatitis B	1542(1150 to 2043)	3324(2310 to 4619)	1.16(0.42 to 2.12)	93.46(68.81 to 125.92)	67.52(47.46 to 94.03)	-0.28(-0.51 to 0.04)
Togo	Liver cancer due to hepatitis C	356(227 to 515)	794(472 to 1159)	1.23(0.59 to 2.13)	28.74(18.49 to 40.96)	22.24(13.76 to 31.88)	-0.23(-0.44 to 0.07)
Togo	Liver cancer due to NASH	170(115 to 247)	471(307 to 708)	1.77(0.93 to 2.86)	12.03(8.01 to 17.92)	11.63(7.54 to 17.99)	-0.03(-0.31 to 0.34)
Togo	Liver cancer due to other causes	420(293 to 596)	583(403 to 812)	0.39(-0.09 to 1.11)	12.12(8.84 to 16.16)	9.1(6.25 to 12.76)	-0.25(-0.47 to 0.04)
Tokelau	Liver cancer	3(2 to 4)	3(2 to 4)	-0.12(-0.4 to 0.25)	233.06(170.24 to 309.33)	199.55(139.16 to 275.1)	-0.14(-0.42 to 0.22)
Tokelau	Liver cancer due to alcohol use	0(0 to 0)	0(0 to 0)	-0.06(-0.34 to 0.33)	24.04(13.41 to 38.02)	21.98(12.12 to 34.68)	-0.09(-0.35 to 0.29)
Tokelau	Liver cancer due to hepatitis B	2(1 to 2)	2(1 to 2)	-0.08(-0.39 to 0.33)	129.75(87.56 to 183.32)	113.19(72.51 to 162.88)	-0.13(-0.42 to 0.25)
Tokelau	Liver cancer due to hepatitis C	1(0 to 1)	0(0 to 1)	-0.25(-0.49 to 0.08)	48.25(29.89 to 70.62)	36.39(23.37 to 52.91)	-0.25(-0.48 to 0.07)
Tokelau	Liver cancer due to NASH	0(0 to 0)	0(0 to 0)	0.02(-0.32 to 0.51)	17.53(10.98 to 27.03)	17.78(10.99 to 26.65)	0.01(-0.31 to 0.5)
Tokelau	Liver cancer due to other causes	0(0 to 0)	0(0 to 0)	-0.29(-0.53 to 0.01)	13.49(8.83 to 20.01)	10.21(6.45 to 15.29)	-0.24(-0.51 to 0.09)
Tonga	Liver cancer	406(280 to 536)	567(402 to 763)	0.4(-0.02 to 1.01)	655.95(453.35 to 861.63)	684.06(487.15 to 913.98)	0.04(-0.26 to 0.5)

Tonga	Liver cancer due to alcohol use	45(24 to 72)	70(40 to 110)	0.55(0.07 to 1.23)	75.07(40.87 to 119)	86.11(49.44 to 134.72)	0.15(-0.2 to 0.64)
Tonga	Liver cancer due to hepatitis B	250(163 to 343)	333(223 to 471)	0.33(-0.1 to 0.95)	395.03(258.33 to 543.35)	396.3(266.92 to 560.3)	0(-0.32 to 0.46)
Tonga	Liver cancer due to hepatitis C	63(39 to 95)	89(54 to 135)	0.42(0.04 to 0.93)	110.25(68.36 to 166.73)	112.23(67.93 to 169.45)	0.02(-0.25 to 0.38)
Tonga	Liver cancer due to NASH	28(17 to 44)	49(30 to 73)	0.75(0.26 to 1.46)	46.86(28.33 to 72.57)	60.05(36.71 to 88.63)	0.28(-0.08 to 0.8)
Tonga	Liver cancer due to other causes	20(13 to 29)	26(16 to 39)	0.27(-0.11 to 0.82)	28.73(18.17 to 42.9)	29.37(18.27 to 44.17)	0.02(-0.28 to 0.46)
Trinidad and Tobago	Liver cancer	1516(1427 to 1612)	1158(859 to 1544)	-0.24(-0.44 to 0.02)	170.55(160.09 to 181.27)	63.3(47.15 to 84.27)	-0.63(-0.73 to -0.5)
Trinidad and Tobago	Liver cancer due to alcohol use	456(339 to 594)	393(253 to 569)	-0.14(-0.38 to 0.17)	52.64(38.49 to 68.69)	20.78(13.41 to 30.04)	-0.61(-0.71 to -0.47)
Trinidad and Tobago	Liver cancer due to hepatitis B	498(383 to 639)	334(216 to 493)	-0.33(-0.52 to -0.09)	54.17(40.99 to 70.67)	18.34(11.95 to 26.87)	-0.66(-0.76 to -0.54)
Trinidad and Tobago	Liver cancer due to hepatitis C	292(202 to 392)	229(141 to 349)	-0.22(-0.42 to 0.04)	35.13(24.28 to 47.04)	12.28(7.7 to 18.35)	-0.65(-0.74 to -0.54)
Trinidad and Tobago	Liver cancer due to NASH	123(89 to 169)	117(74 to 180)	-0.05(-0.32 to 0.28)	14.1(10.05 to 19.42)	6.43(4.14 to 9.76)	-0.54(-0.68 to -0.39)
Trinidad and Tobago	Liver cancer due to other causes	146(116 to 183)	85(55 to 125)	-0.42(-0.59 to -0.22)	14.51(11.1 to 18.62)	5.46(3.68 to 7.79)	-0.62(-0.73 to -0.5)
Tunisia	Liver cancer	2597(2058 to 3273)	5321(3684 to 7618)	1.05(0.32 to 2.1)	46.55(36.27 to 59.05)	41.54(28.94 to 59.06)	-0.11(-0.41 to 0.36)

Tunisia	Liver cancer due to alcohol use	250(138 to 430)	624(314 to 1144)	1.5(0.52 to 3.07)	4.75(2.64 to 8.15)	4.8(2.42 to 8.85)	0.01(-0.38 to 0.65)
Tunisia	Liver cancer due to hepatitis B	922(637 to 1293)	1746(1034 to 2717)	0.89(0.17 to 1.93)	16.08(11.03 to 22.74)	13.16(7.87 to 20.38)	-0.18(-0.49 to 0.27)
Tunisia	Liver cancer due to hepatitis C	968(665 to 1331)	2252(1465 to 3363)	1.33(0.52 to 2.71)	19.26(13.35 to 26.07)	17.84(11.66 to 26.56)	-0.07(-0.39 to 0.45)
Tunisia	Liver cancer due to NASH	138(90 to 212)	382(224 to 616)	1.77(0.77 to 3.35)	2.61(1.7 to 4.03)	3.01(1.77 to 4.9)	0.16(-0.25 to 0.81)
Tunisia	Liver cancer due to other causes	319(219 to 460)	317(207 to 472)	-0.01(-0.4 to 0.59)	3.85(2.72 to 5.4)	2.72(1.82 to 3.92)	-0.29(-0.54 to 0.07)
Turkey	Liver cancer	38384(30628 to 46902)	58921(46456 to 73230)	0.54(0.12 to 1.15)	96.29(76.81 to 116.3)	66.43(52.76 to 82.49)	-0.31(-0.49 to -0.04)
Turkey	Liver cancer due to alcohol use	4018(2523 to 6040)	7560(4730 to 11475)	0.88(0.35 to 1.74)	11.04(7.06 to 16.35)	8.47(5.26 to 12.91)	-0.23(-0.45 to 0.1)
Turkey	Liver cancer due to hepatitis B	20327(15434 to 26041)	28363(20876 to 38019)	0.4(-0.02 to 1)	49.33(37.44 to 63.1)	30.89(22.72 to 41.54)	-0.37(-0.56 to -0.11)
Turkey	Liver cancer due to hepatitis C	7295(4611 to 10570)	13087(8616 to 17915)	0.79(0.29 to 1.63)	20.77(13.34 to 29.58)	15.15(10.03 to 20.62)	-0.27(-0.47 to 0.06)
Turkey	Liver cancer due to NASH	2674(1790 to 3889)	5779(3865 to 8330)	1.16(0.48 to 2.07)	7.19(4.73 to 10.58)	6.62(4.45 to 9.55)	-0.08(-0.36 to 0.31)
Turkey	Liver cancer due to other causes	4069(3012 to 5483)	4133(3008 to 5588)	0.02(-0.28 to 0.4)	7.97(5.76 to 10.84)	5.31(3.97 to 6.91)	-0.33(-0.51 to -0.11)
Turkmenistan	Liver cancer	867(792 to 946)	7299(5740 to 9325)	7.42(5.53 to 9.77)	32.44(30.33 to 34.7)	160.51(126.73 to 204.64)	3.95(2.88 to 5.33)
Turkmenistan	Liver cancer due to alcohol use	121(85 to 163)	2033(1330 to 2899)	15.82(11.56 to 21.17)	6.16(4.3 to 8.3)	46.01(30.52 to 65.17)	6.47(4.58 to 8.8)

Turkmenistan	Liver cancer due to hepatitis B	165(125 to 213)	2266(1560 to 3216)	12.75(9.43 to 17.5)	7.37(5.51 to 9.73)	46.63(32.12 to 66.35)	5.33(3.8 to 7.48)
Turkmenistan	Liver cancer due to hepatitis C	185(135 to 236)	1832(1174 to 2568)	8.88(6.44 to 11.82)	9.98(7.47 to 12.49)	43.36(28.6 to 60.59)	3.34(2.31 to 4.57)
Turkmenistan	Liver cancer due to NASH	30(21 to 41)	479(313 to 702)	14.91(10.47 to 20.51)	1.52(1.06 to 2.14)	10.92(7.19 to 16.22)	6.19(4.25 to 8.59)
Turkmenistan	Liver cancer due to other causes	366(302 to 441)	690(548 to 878)	0.88(0.44 to 1.53)	7.41(6.18 to 8.83)	13.6(10.7 to 17.28)	0.83(0.43 to 1.41)
Tuvalu	Liver cancer	22(17 to 28)	26(19 to 35)	0.17(-0.17 to 0.68)	293.76(220.25 to 379.35)	238.33(173.39 to 325.61)	-0.19(-0.42 to 0.16)
Tuvalu	Liver cancer due to alcohol use	2(1 to 4)	3(2 to 5)	0.27(-0.11 to 0.91)	30.85(18.07 to 47.72)	27.09(15.42 to 43.32)	-0.12(-0.38 to 0.3)
Tuvalu	Liver cancer due to hepatitis B	13(9 to 18)	15(10 to 22)	0.17(-0.19 to 0.73)	168.94(119.06 to 232.76)	137.64(93.9 to 198.14)	-0.19(-0.44 to 0.21)
Tuvalu	Liver cancer due to hepatitis C	4(2 to 6)	4(3 to 7)	0.1(-0.22 to 0.57)	57.84(35.82 to 88.32)	42.89(26.04 to 64.21)	-0.26(-0.47 to 0.04)
Tuvalu	Liver cancer due to NASH	1(1 to 2)	2(1 to 3)	0.4(-0.02 to 1.01)	19.84(12.75 to 30.28)	18.92(12.13 to 28.96)	-0.05(-0.33 to 0.34)
Tuvalu	Liver cancer due to other causes	1(1 to 2)	1(1 to 2)	-0.05(-0.35 to 0.41)	16.29(10.71 to 24.03)	11.79(7.65 to 17.72)	-0.28(-0.51 to 0.06)
Uganda	Liver cancer	10860(8575 to 13356)	33433(25255 to 43035)	2.08(1.24 to 3.35)	126.37(100.82 to 154.47)	169.93(131.54 to 213.42)	0.34(-0.01 to 0.87)
Uganda	Liver cancer due to alcohol use	2815(1824 to 3914)	8443(5565 to 12267)	2(1.15 to 3.4)	40.13(26.18 to 55.52)	52.47(35.47 to 74.78)	0.31(-0.04 to 0.85)

Uganda	Liver cancer due to hepatitis B	3389(2346 to 4747)	10581(7066 to 15226)	2.12(1.17 to 3.73)	41.93(28.78 to 59.6)	51.59(34.56 to 75.01)	0.23(-0.12 to 0.82)
Uganda	Liver cancer due to hepatitis C	1373(867 to 1988)	4307(2709 to 6186)	2.14(1.25 to 3.37)	21.11(13.5 to 30.07)	30.26(19.47 to 43.3)	0.43(0.05 to 0.94)
Uganda	Liver cancer due to NASH	769(516 to 1119)	2911(1943 to 4352)	2.79(1.67 to 4.55)	10.75(7.16 to 15.87)	17.45(11.8 to 25.94)	0.62(0.18 to 1.27)
Uganda	Liver cancer due to other causes	2514(1654 to 3715)	7191(4559 to 11018)	1.86(0.72 to 3.7)	12.45(8.74 to 17.01)	18.16(12.58 to 25.82)	0.46(0.01 to 1.1)
Ukraine	Liver cancer	17421(16382 to 18361)	46740(38901 to 55218)	1.68(1.22 to 2.22)	26.66(24.6 to 28.47)	69.89(57.74 to 83.04)	1.62(1.13 to 2.23)
Ukraine	Liver cancer due to alcohol use	4879(4182 to 5567)	16480(13070 to 20437)	2.38(1.78 to 3.11)	6.72(5.78 to 7.64)	22.72(17.93 to 28.26)	2.38(1.78 to 3.13)
Ukraine	Liver cancer due to hepatitis B	4340(3683 to 5098)	12887(10055 to 16273)	1.97(1.42 to 2.64)	6.28(5.38 to 7.29)	20.15(15.77 to 25.16)	2.21(1.59 to 2.96)
Ukraine	Liver cancer due to hepatitis C	4433(3798 to 5097)	11146(8958 to 13728)	1.51(1.11 to 1.97)	6.12(5.28 to 6.98)	14.68(11.86 to 18.11)	1.4(1.01 to 1.85)
Ukraine	Liver cancer due to NASH	1163(976 to 1382)	3433(2734 to 4281)	1.95(1.46 to 2.51)	1.64(1.39 to 1.93)	4.9(3.91 to 6.11)	1.99(1.48 to 2.58)
Ukraine	Liver cancer due to other causes	2605(1934 to 3187)	2792(2059 to 3723)	0.07(-0.29 to 0.68)	5.91(4.23 to 7.33)	7.45(5.19 to 10.74)	0.26(-0.23 to 1.13)
United Arab Emirates	Liver cancer	721(406 to 1229)	7537(3259 to 16843)	9.45(4.57 to 17.06)	106.26(53.57 to 202.91)	114.72(47.87 to 262.27)	0.08(-0.38 to 0.68)
United Arab Emirates	Liver cancer due to alcohol use	69(28 to 144)	747(237 to 2007)	9.88(4.59 to 17.33)	13.08(5.15 to 29.24)	13.89(4.5 to 37.59)	0.06(-0.42 to 0.71)
United Arab Emirates	Liver cancer due to hepatitis B	382(197 to 704)	4189(1757 to 9507)	9.96(4.64 to 18.35)	43.17(20.56 to 88.88)	47.72(19.4 to	0.11(-0.39 to 0.76)

						113.57)	
United Arab Emirates	Liver cancer due to hepatitis C	135(68 to 264)	1409(521 to 3537)	9.43(4.73 to 16.25)	32.85(15.32 to 66.29)	32.73(12.46 to 79.78)	0(-0.42 to 0.53)
United Arab Emirates	Liver cancer due to NASH	52(27 to 93)	712(280 to 1705)	12.64(6.28 to 21.53)	9.74(4.57 to 19.28)	13.62(5.45 to 33.27)	0.4(-0.2 to 1.2)
United Arab Emirates	Liver cancer due to other causes	83(56 to 117)	481(213 to 1053)	4.81(2.11 to 9.85)	7.42(4.56 to 12.87)	6.76(3.32 to 14.42)	-0.09(-0.43 to 0.41)
United Kingdom	Liver cancer	36234(35174 to 37210)	99126(93075 to 104503)	1.74(1.59 to 1.87)	44.13(42.92 to 45.29)	87.74(82.89 to 92.4)	0.99(0.88 to 1.09)
United Kingdom	Liver cancer due to alcohol use	13300(11720 to 14857)	36819(32290 to 41376)	1.77(1.58 to 1.94)	15.68(13.76 to 17.47)	32.05(28.19 to 36.16)	1.04(0.91 to 1.18)
United Kingdom	Liver cancer due to hepatitis B	5675(4756 to 6752)	14663(12194 to 17520)	1.58(1.42 to 1.74)	7.5(6.33 to 8.88)	14.63(12.26 to 17.29)	0.95(0.83 to 1.07)
United Kingdom	Liver cancer due to hepatitis C	12531(11123 to 14099)	35044(30906 to 39630)	1.8(1.65 to 1.93)	14.27(12.56 to 16.08)	28.67(25.18 to 32.52)	1.01(0.91 to 1.1)
United Kingdom	Liver cancer due to NASH	2159(1838 to 2551)	6984(5868 to 8219)	2.23(2.06 to 2.4)	2.51(2.15 to 2.92)	5.94(5.02 to 6.95)	1.37(1.25 to 1.48)
United Kingdom	Liver cancer due to other causes	2570(2291 to 2860)	5615(4867 to 6434)	1.19(1.05 to 1.32)	4.17(3.8 to 4.56)	6.45(5.68 to 7.23)	0.55(0.42 to 0.66)
United Republic of Tanzania	Liver cancer	10649(8108 to 14042)	22684(16597 to 30526)	1.13(0.45 to 2.01)	59.09(47.25 to 73.03)	62.79(48.52 to 80.1)	0.06(-0.21 to 0.39)
United Republic of Tanzania	Liver cancer due to alcohol use	1531(975 to 2223)	3917(2531 to 5796)	1.56(0.84 to 2.43)	13.28(8.62 to 19.32)	15.33(9.9 to 22.52)	0.15(-0.15 to 0.53)

United Republic of Tanzania	Liver cancer due to hepatitis B	2443(1763 to 3301)	5557(3773 to 7983)	1.27(0.59 to 2.25)	16.94(11.96 to 23.58)	16.86(11.4 to 24.42)	0(-0.28 to 0.35)
United Republic of Tanzania	Liver cancer due to hepatitis C	1145(743 to 1631)	2779(1826 to 3927)	1.43(0.84 to 2.12)	10.77(7.2 to 14.86)	11.67(7.8 to 16.34)	0.08(-0.16 to 0.38)
United Republic of Tanzania	Liver cancer due to NASH	705(489 to 991)	1962(1334 to 2813)	1.78(1.05 to 2.78)	5.79(4.05 to 8.11)	7.21(4.9 to 10.36)	0.24(-0.05 to 0.64)
United Republic of Tanzania	Liver cancer due to other causes	4825(2937 to 7675)	8470(4684 to 14188)	0.76(-0.11 to 2.34)	12.3(8.37 to 17.94)	11.73(7.35 to 18.04)	-0.05(-0.45 to 0.58)
United States of America	Liver cancer	152279(148428 to 155467)	551263(491021 to 603899)	2.62(2.22 to 2.97)	51.35(50.17 to 52.4)	107.18(95.59 to 117.45)	1.09(0.86 to 1.29)
United States of America	Liver cancer due to alcohol use	44239(39191 to 49163)	176991(144023 to 211258)	3(2.43 to 3.5)	14.65(12.98 to 16.35)	33.79(27.58 to 40.41)	1.31(0.98 to 1.59)
United States of America	Liver cancer due to hepatitis B	24008(20964 to 27378)	72865(58771 to 89847)	2.04(1.61 to 2.43)	8.3(7.24 to 9.49)	15.22(12.4 to 18.54)	0.83(0.59 to 1.06)
United States of America	Liver cancer due to hepatitis C	51425(46179 to 56454)	199612(169775 to 231284)	2.88(2.46 to 3.25)	16.86(15.12 to 18.49)	37.02(31.37 to 42.8)	1.2(0.96 to 1.4)
United States of America	Liver cancer due to NASH	13378(11618 to 15462)	51959(42091 to 62315)	2.88(2.44 to 3.27)	4.3(3.74 to 4.96)	9.76(7.94 to 11.63)	1.27(1.02 to 1.49)
United States of America	Liver cancer due to other causes	19228(17317 to 21208)	49837(41803 to 58644)	1.59(1.3 to 1.87)	7.24(6.57 to 7.95)	11.39(9.75 to 13.09)	0.57(0.42 to 0.73)
United States Virgin Islands	Liver cancer	97(80 to 116)	110(89 to 131)	0.13(-0.15 to 0.49)	105.17(86.82 to 124.24)	61.81(50.03 to 75.12)	-0.41(-0.56 to -0.21)

United States Virgin Islands	Liver cancer due to alcohol use	29(20 to 39)	39(28 to 54)	0.38(-0.01 to 0.89)	31.5(22.09 to 42.58)	20.94(14.62 to 28.98)	-0.34(-0.52 to -0.09)
United States Virgin Islands	Liver cancer due to hepatitis B	35(25 to 46)	31(21 to 44)	-0.11(-0.36 to 0.23)	34.85(24.13 to 46.8)	18.62(12.96 to 26.58)	-0.47(-0.62 to -0.25)
United States Virgin Islands	Liver cancer due to hepatitis C	17(11 to 25)	21(14 to 30)	0.21(-0.09 to 0.62)	20.63(13.63 to 29.06)	11.15(7.26 to 15.73)	-0.46(-0.59 to -0.29)
United States Virgin Islands	Liver cancer due to NASH	8(6 to 12)	12(9 to 18)	0.49(0.08 to 1.07)	9.56(6.57 to 13.58)	6.85(4.69 to 9.8)	-0.28(-0.47 to -0.03)
United States Virgin Islands	Liver cancer due to other causes	9(6 to 11)	6(4 to 9)	-0.28(-0.47 to -0.02)	8.63(6.35 to 11.45)	4.26(3.04 to 5.78)	-0.51(-0.62 to -0.34)
Uruguay	Liver cancer	1279(1115 to 1445)	2660(2381 to 2945)	1.08(0.73 to 1.46)	33.93(29.69 to 38.22)	54.16(48.49 to 60.16)	0.6(0.32 to 0.89)
Uruguay	Liver cancer due to alcohol use	367(256 to 503)	824(584 to 1079)	1.24(0.78 to 1.81)	9.49(6.64 to 12.99)	16.62(11.85 to 21.82)	0.75(0.39 to 1.19)
Uruguay	Liver cancer due to hepatitis B	285(200 to 405)	534(379 to 749)	0.88(0.5 to 1.31)	7.84(5.6 to 11.06)	11.72(8.43 to 16.28)	0.5(0.19 to 0.84)
Uruguay	Liver cancer due to hepatitis C	411(290 to 541)	881(635 to 1156)	1.14(0.81 to 1.53)	10.47(7.45 to 13.76)	16.76(12.02 to 22.24)	0.6(0.34 to 0.89)
Uruguay	Liver cancer due to NASH	95(64 to 135)	248(177 to 342)	1.61(1.13 to 2.19)	2.44(1.68 to 3.47)	4.8(3.46 to 6.6)	0.97(0.61 to 1.4)
Uruguay	Liver cancer due to other causes	121(97 to 153)	173(130 to 233)	0.43(0.19 to 0.7)	3.69(3.02 to 4.51)	4.26(3.29 to 5.47)	0.16(-0.03 to 0.35)
Uzbekistan	Liver cancer	3684(3023 to 4246)	40800(33111 to 49339)	10.07(7.35 to 13.94)	24.69(21.77 to 27.1)	164.72(135.82 to 195.88)	5.67(4.26 to 7.4)
Uzbekistan	Liver cancer due to alcohol use	580(399 to 768)	10839(7106 to 15145)	17.68(13.46 to 22.23)	5.07(3.48 to 6.73)	45.2(30.37 to	7.92(6.16 to 9.93)

						62.14)	
Uzbekistan	Liver cancer due to hepatitis B	757(580 to 975)	12946(9285 to 17716)	16.1(12.38 to 20.26)	5.91(4.45 to 7.76)	45.14(32.17 to 62.59)	6.64(5.05 to 8.37)
Uzbekistan	Liver cancer due to hepatitis C	823(608 to 1045)	11163(7358 to 15385)	12.56(9.76 to 15.61)	7.48(5.62 to 9.43)	52.05(36.46 to 69.77)	5.96(4.71 to 7.37)
Uzbekistan	Liver cancer due to NASH	142(100 to 201)	2852(1901 to 4089)	19.15(14.51 to 25.13)	1.22(0.84 to 1.76)	12.38(8.44 to 17.72)	9.15(7.05 to 11.91)
Uzbekistan	Liver cancer due to other causes	1382(824 to 1879)	2999(2084 to 4151)	1.17(0.19 to 3.37)	5.01(3.13 to 6.7)	9.95(6.92 to 13.77)	0.98(0.15 to 2.7)
Vanuatu	Liver cancer	199(129 to 298)	508(340 to 730)	1.55(0.66 to 2.85)	255.08(164.52 to 385.91)	255.58(172.13 to 366.89)	0(-0.33 to 0.51)
Vanuatu	Liver cancer due to alcohol use	21(11 to 37)	58(31 to 96)	1.78(0.8 to 3.29)	29.16(14.61 to 50.69)	30.97(16.47 to 51.03)	0.06(-0.3 to 0.62)
Vanuatu	Liver cancer due to hepatitis B	125(79 to 191)	308(193 to 455)	1.47(0.55 to 2.76)	152.07(94.95 to 235.08)	148.64(93.98 to 222.78)	-0.02(-0.38 to 0.49)
Vanuatu	Liver cancer due to hepatitis C	30(17 to 50)	78(43 to 122)	1.6(0.78 to 2.91)	46.26(26.19 to 77.93)	44.81(25.47 to 68.44)	-0.03(-0.33 to 0.47)
Vanuatu	Liver cancer due to NASH	12(7 to 19)	36(21 to 56)	2.1(1.06 to 3.66)	15.88(8.88 to 26.18)	18.93(11.4 to 29.69)	0.19(-0.19 to 0.8)
Vanuatu	Liver cancer due to other causes	12(8 to 18)	28(18 to 41)	1.36(0.64 to 2.5)	11.71(6.86 to 18.09)	12.23(7.4 to 18.5)	0.04(-0.29 to 0.59)
Venezuela (Bolivarian Republic of)	Liver cancer	16961(16219 to 17771)	16042(12188 to 21213)	-0.05(-0.29 to 0.24)	161.22(153.98 to 168.72)	54.67(41.68 to 72.04)	-0.66(-0.74 to -0.56)

Venezuela (Bolivarian Republic of)	Liver cancer due to alcohol use	5446(4028 to 6878)	5659(3717 to 8158)	0.04(-0.23 to 0.39)	54.24(40.04 to 68.26)	19.05(12.56 to 27.37)	-0.65(-0.74 to -0.53)
Venezuela (Bolivarian Republic of)	Liver cancer due to hepatitis B	3465(2561 to 4620)	2848(1818 to 4429)	-0.18(-0.4 to 0.1)	29.95(21.3 to 41.06)	9.38(6 to 14.44)	-0.69(-0.77 to -0.58)
Venezuela (Bolivarian Republic of)	Liver cancer due to hepatitis C	5228(3852 to 6642)	5033(3251 to 7189)	-0.04(-0.28 to 0.26)	54.55(40.26 to 69.01)	17.4(11.39 to 24.74)	-0.68(-0.76 to -0.58)
Venezuela (Bolivarian Republic of)	Liver cancer due to NASH	1115(801 to 1537)	1281(830 to 1945)	0.15(-0.15 to 0.56)	10.76(7.54 to 15.12)	4.42(2.89 to 6.68)	-0.59(-0.7 to -0.45)
Venezuela (Bolivarian Republic of)	Liver cancer due to other causes	1706(1388 to 2072)	1222(866 to 1700)	-0.28(-0.46 to -0.05)	11.71(9.06 to 15.11)	4.43(3.16 to 6.09)	-0.62(-0.72 to -0.5)
Viet Nam	Liver cancer	40968(30330 to 51889)	59180(44445 to 75249)	0.44(-0.02 to 1.15)	95.78(70.79 to 121.55)	62.15(47.45 to 77.93)	-0.35(-0.56 to -0.04)
Viet Nam	Liver cancer due to alcohol use	6940(4265 to 10861)	13720(8226 to 20766)	0.98(0.27 to 2.05)	16.83(10.4 to 26.24)	14.55(8.75 to 21.71)	-0.14(-0.44 to 0.31)
Viet Nam	Liver cancer due to hepatitis B	20379(13764 to 27825)	26193(17803 to 37140)	0.29(-0.16 to 0.96)	47.06(31.92 to 64.46)	25.76(17.56 to 36.03)	-0.45(-0.64 to -0.17)
Viet Nam	Liver cancer due to hepatitis C	8035(4467 to 12743)	11612(6250 to 18674)	0.45(-0.01 to 1.23)	20.28(11.36 to 31.85)	13.16(7.39 to 20.87)	-0.35(-0.56 to 0)
Viet Nam	Liver cancer due to NASH	2883(1817 to 4389)	4661(2875 to 7175)	0.62(0.06 to 1.53)	7.07(4.41 to 10.65)	5.17(3.26 to 7.89)	-0.27(-0.51 to 0.13)
Viet Nam	Liver cancer due to other causes	2730(2021 to 3664)	2995(2234 to 4118)	0.1(-0.19 to 0.45)	4.54(3.26 to 6.22)	3.53(2.67 to 4.81)	-0.22(-0.42 to 0.03)

Yemen	Liver cancer	4594(2898 to 6896)	11670(8384 to 16108)	1.54(0.72 to 2.82)	80.43(50.66 to 118.23)	77.98(56.25 to 107.52)	-0.03(-0.33 to 0.43)
Yemen	Liver cancer due to alcohol use	249(127 to 455)	713(406 to 1206)	1.86(0.91 to 3.34)	4.86(2.59 to 8.85)	5.27(3 to 8.98)	0.08(-0.27 to 0.62)
Yemen	Liver cancer due to hepatitis B	1702(996 to 2812)	4233(2707 to 6406)	1.49(0.63 to 2.84)	28.92(16.92 to 47.19)	26.1(16.36 to 39.58)	-0.1(-0.4 to 0.37)
Yemen	Liver cancer due to hepatitis C	1622(934 to 2570)	4243(2744 to 6224)	1.62(0.79 to 2.9)	34(20.07 to 53.39)	33.05(21.76 to 47.94)	-0.03(-0.32 to 0.41)
Yemen	Liver cancer due to NASH	278(146 to 459)	871(531 to 1399)	2.13(1.08 to 3.77)	5.48(2.92 to 9.12)	6.26(3.86 to 10.01)	0.14(-0.22 to 0.7)
Yemen	Liver cancer due to other causes	743(425 to 1311)	1610(1080 to 2276)	1.17(0.34 to 2.49)	7.17(4.14 to 11.51)	7.3(4.72 to 10.63)	0.02(-0.3 to 0.56)
Zambia	Liver cancer	3043(2153 to 4249)	6879(5129 to 8790)	1.26(0.49 to 2.47)	66.43(49.67 to 93.99)	73.59(56.01 to 92.69)	0.11(-0.26 to 0.62)
Zambia	Liver cancer due to alcohol use	454(266 to 803)	1319(832 to 1881)	1.9(0.74 to 3.39)	15.38(9.04 to 27.47)	18.74(12.12 to 26.27)	0.22(-0.27 to 0.85)
Zambia	Liver cancer due to hepatitis B	794(537 to 1194)	2170(1468 to 3020)	1.73(0.76 to 3.18)	20.29(13.36 to 31.04)	22.22(15 to 31.56)	0.09(-0.3 to 0.64)
Zambia	Liver cancer due to hepatitis C	348(214 to 538)	913(578 to 1324)	1.62(0.74 to 2.86)	12.5(7.9 to 19.1)	14.21(9.17 to 20.08)	0.14(-0.24 to 0.61)
Zambia	Liver cancer due to NASH	233(155 to 350)	684(446 to 985)	1.93(0.93 to 3.48)	7.06(4.68 to 10.71)	8.76(5.68 to 13.11)	0.24(-0.18 to 0.82)
Zambia	Liver cancer due to other causes	1213(651 to 2154)	1792(1157 to 2665)	0.48(-0.28 to 2.11)	11.2(6.99 to 17.3)	9.65(6.74 to 13.45)	-0.14(-0.52 to 0.5)
Zimbabwe	Liver cancer	15405(11751 to 24304)	32215(23313 to 45365)	1.09(0.41 to 1.95)	317.2(244.83 to 495.24)	373.68(274.31 to	0.18(-0.21 to 0.64)

						511.59)	
Zimbabwe	Liver cancer due to alcohol use	2372(1291 to 4601)	4492(2627 to 7606)	0.89(0.26 to 1.72)	52.35(29.16 to 101.42)	55.83(32.87 to 92.74)	0.07(-0.28 to 0.51)
Zimbabwe	Liver cancer due to hepatitis B	7316(4978 to 12517)	13937(9212 to 21778)	0.91(0.27 to 1.75)	140.75(93.95 to 242.19)	144.76(95.78 to 224.22)	0.03(-0.31 to 0.47)
Zimbabwe	Liver cancer due to hepatitis C	3518(2359 to 5189)	8450(5545 to 12020)	1.4(0.63 to 2.41)	84.92(58.31 to 122.91)	117.34(77.64 to 166.82)	0.38(-0.05 to 0.94)
Zimbabwe	Liver cancer due to NASH	1038(662 to 1670)	2568(1635 to 3890)	1.47(0.66 to 2.53)	22.56(14.2 to 36.7)	31.64(20.16 to 47.5)	0.4(-0.05 to 1)
Zimbabwe	Liver cancer due to other causes	1161(799 to 1723)	2767(1910 to 3873)	1.38(0.63 to 2.43)	16.62(11.04 to 25.66)	24.1(16.13 to 34.92)	0.45(-0.03 to 1.08)
