# Supplementary material for: The burden of primary liver cancer caused by specific etiologies from 1990 to 2019 at the global, regional, and national levels
Source: Cancer Med. 2022 Jan 6;11(5):1357–70. doi: 10.1002/cam4.4530 (PMC8894689; doi:10.1002/cam4.4530)
Supplement: Supplementary file 2 — Supplementary Material2 [file CAM4-11-1357-s001.pdf]

Additional file 2

The burden of primary liver cancer caused by specific etiologies from 1990 to 2019 at the Global, Regional, and National Level: results from the Global Burden of Disease Study 2019

Fig S1: Number of incident cases and age-standardised incidence rates at the global level by etiology of primary liver cancer, 1990–2019. 3

Fig S2: Age-standardized incidence rate for liver cancer, by region and etiology, 2019. 4

Fig S3: Number of DALYs and age-standardised DALYs rates at the global level by etiology of primary liver cancer, 1990–2019........5

Fig S4: Age-standardized DALYs rate for liver cancer, by region and etiology, 2019........6

Fig S5: Contribution of LCHB, LCHC, LCAU, LCNA, and LCOC to primary liver cancer incident cases, both sexes, globally and by region, 2019........7

Fig S6: Contribution of LCHB, LCHC, LCAU, LCNA, and LCOC to primary liver cancer DALYs, both sexes, globally and by region, 2019......8

Fig S7: The estimated percentage change of primary liver cancer from 1990 to 2019: (A) The percentage change in ASMR. (B) The percentage change in ASIR. (C) The percentage change in ASDR. 9

Fig S8: The global age-standardized rate of LCHB per 100 000 populations in 2019, by country and territory. (A) ASMR in 2019; (B) ASIR in 2019; (C) ASDR in 2019. 10

Fig S9: The estimated percentage change of LCHB from 1990 to 2019: (A) The percentage change in ASMR. (B) The percentage change in ASIR. (C) The percentage change in ASDR........11

Fig S10: The global age-standardized rate of LCHC per 100 000 populations in 2019, by country and territory. (A) ASMR in 2019; (B) ASIR in 2019; (C) ASDR in 2019........12

Fig S11: The estimated percentage change of LCHC from 1990 to 2019: (A) The percentage change in ASMR. (B) The percentage change in ASIR. (C) The percentage change in ASDR....................13
Fig S12: The global age-standardized rate of LCAU per 100 000 populations in 2019, by country and territory. (A) ASMR in 2019; (B) ASIR in 2019; (C) ASDR in 2019....................14
Fig S13: The estimated percentage change of LCAU from 1990 to 2019: (A) The percentage change in ASMR. (B) The percentage change in ASIR. (C) The percentage change in ASDR....................15
Fig S14: The global age-standardized rate of LCSH per 100 000 populations in 2019, by country and territory. (A) ASMR in 2019; (B) ASIR in 2019; (C) ASDR in 2019....................16
Fig S15: The estimated percentage change of LCSH from 1990 to 2019: (A) The percentage change in ASMR. (B) The percentage change in ASIR. (C) The percentage change in ASDR....................17
Fig S16: The global age-standardized rate of LCOC per 100 000 populations in 2019, by country and territory. (A) ASMR in 2019; (B) ASIR in 2019; (C) ASDR in 2019....................18
Fig S17: The estimated percentage change of LCOC from 1990 to 2019: (A) The percentage change in ASMR. (B) The percentage change in ASIR. (C) The percentage change in ASDR....................19
Fig S18: Global primary liver cancer incident cases by etiology and age for females and males, 2019....................20
Fig S19: Global primary liver cancer DALYs by etiology and age for females and males, 2019....................21
Fig S20: Age-standardised rates of primary liver cancer globally and for 21 regions by SDI, 1990-2019....................22
Fig S21: Age-standardised rates per 100 000 population by 204 countries and sociodemographic index(SDI), 2019....................23

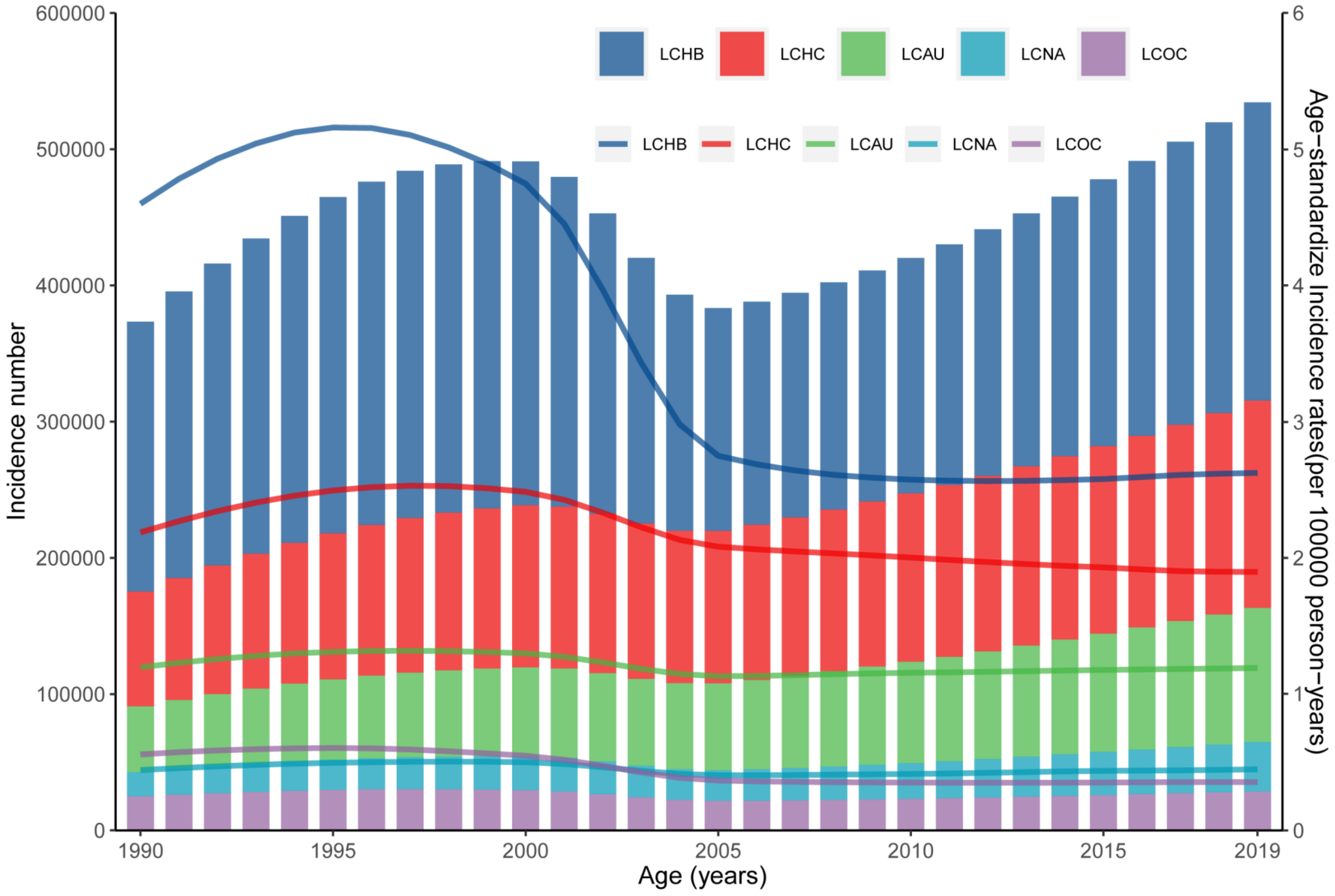

Fig S1: Number of incident cases and age-standardised incidence rates at the global level by etiology of primary liver cancer, 1990–2019.

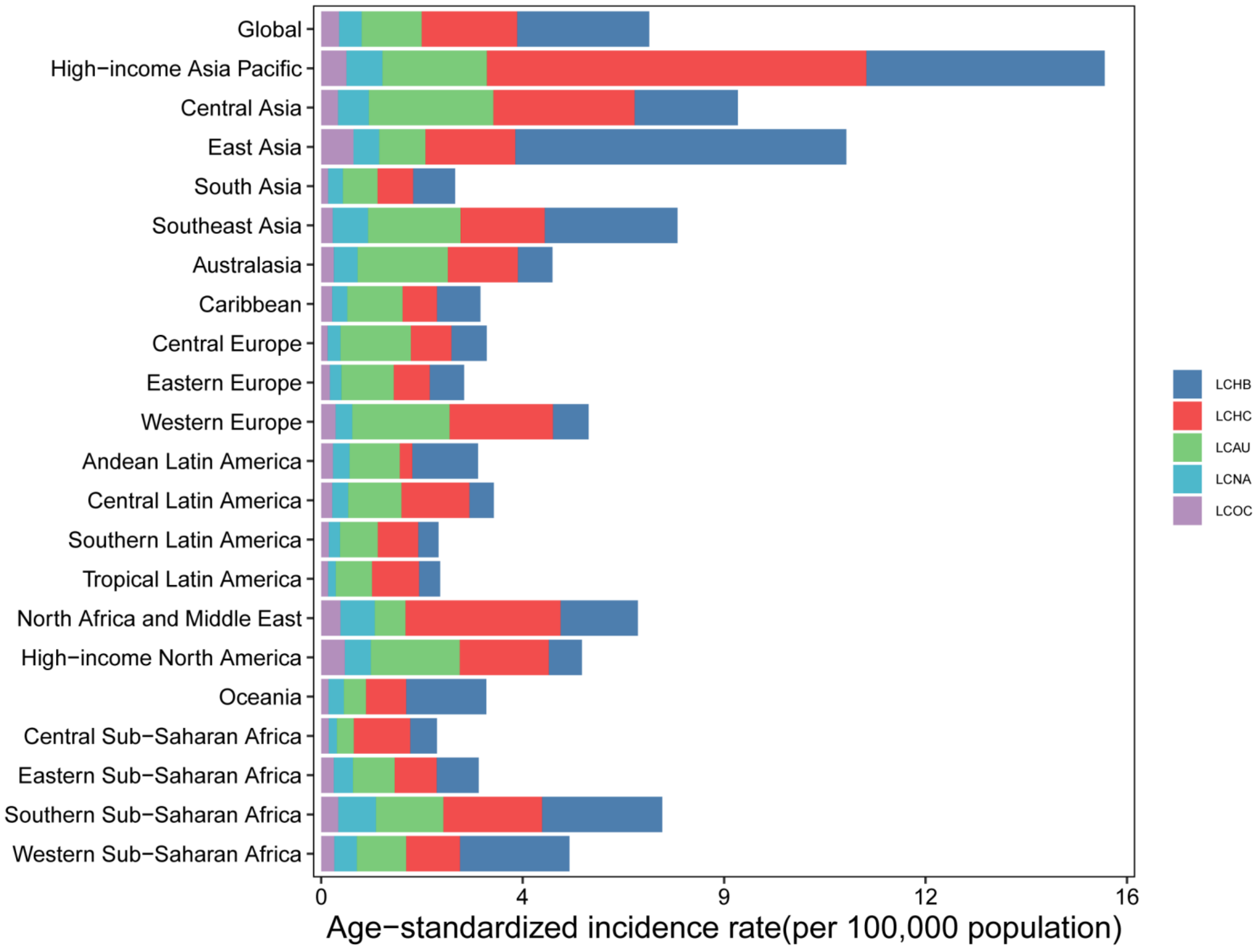

Fig S2: Age-standardized incidence rate for liver cancer, by region and etiology, 2019.

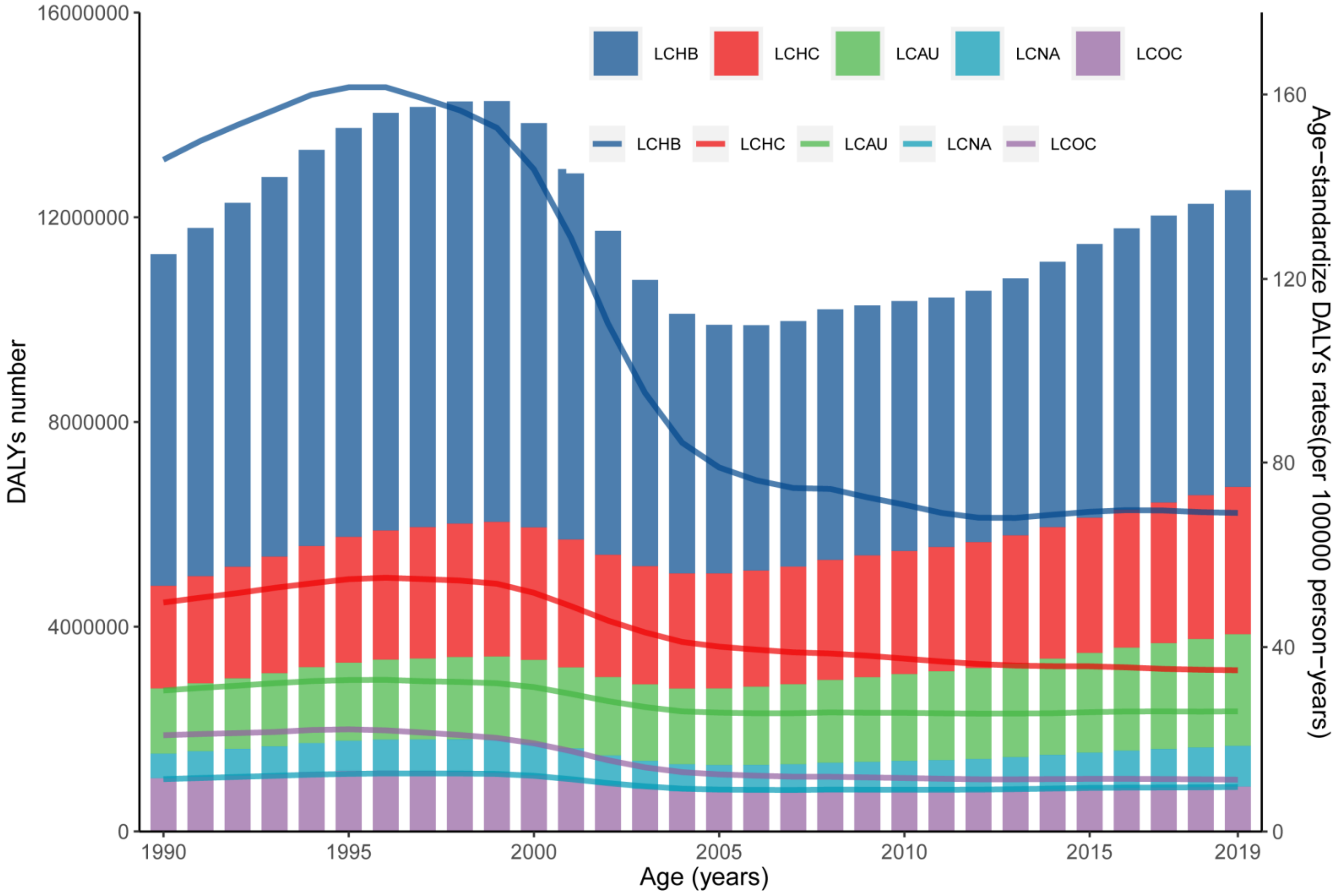

Fig S3: Number of DALYs and age-standardised DALYs rates at the global level by etiology of primary liver cancer, 1990–2019.

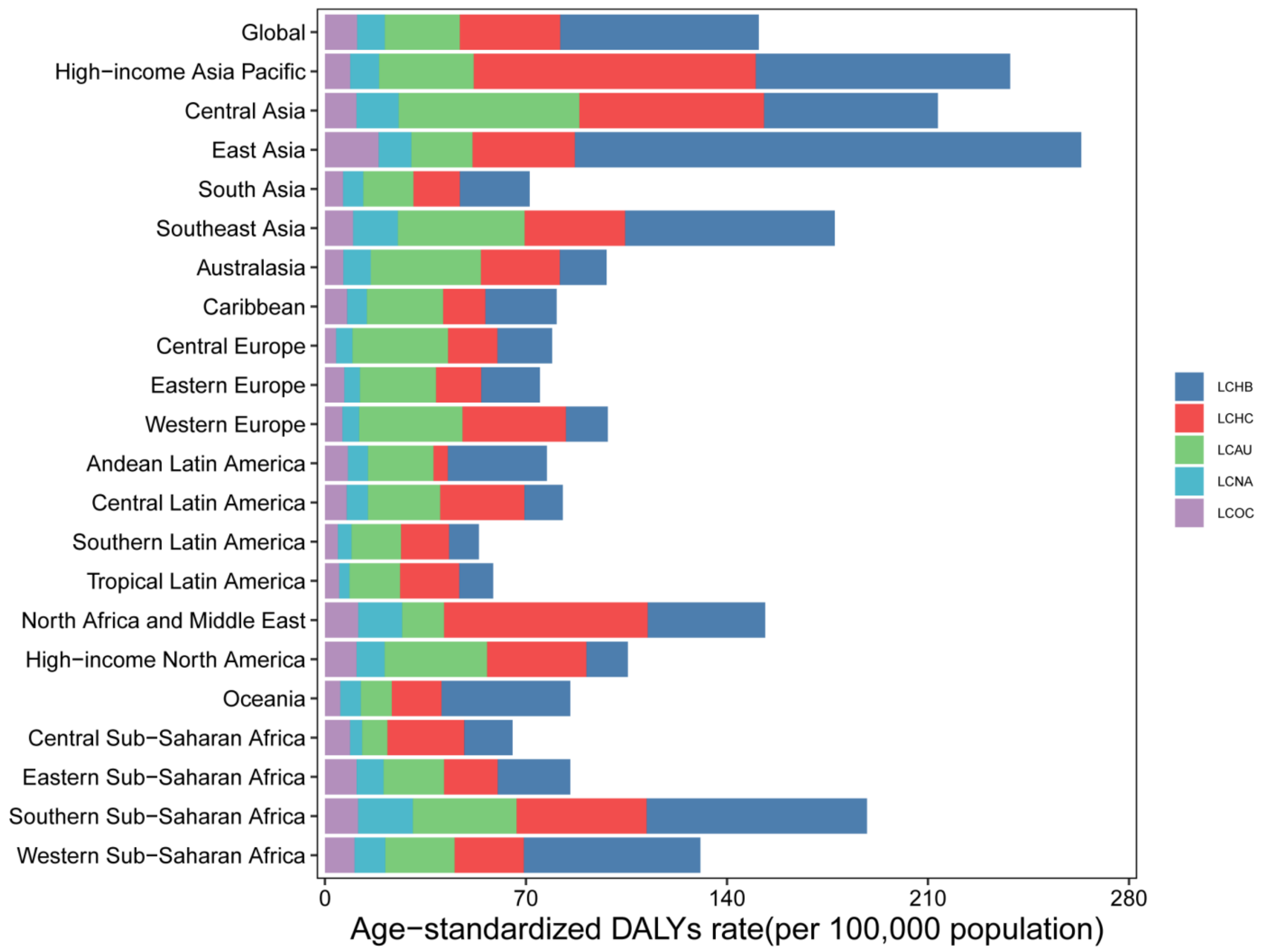

Fig S4: Age-standardized DALYs rate for liver cancer, by region and etiology, 2019.

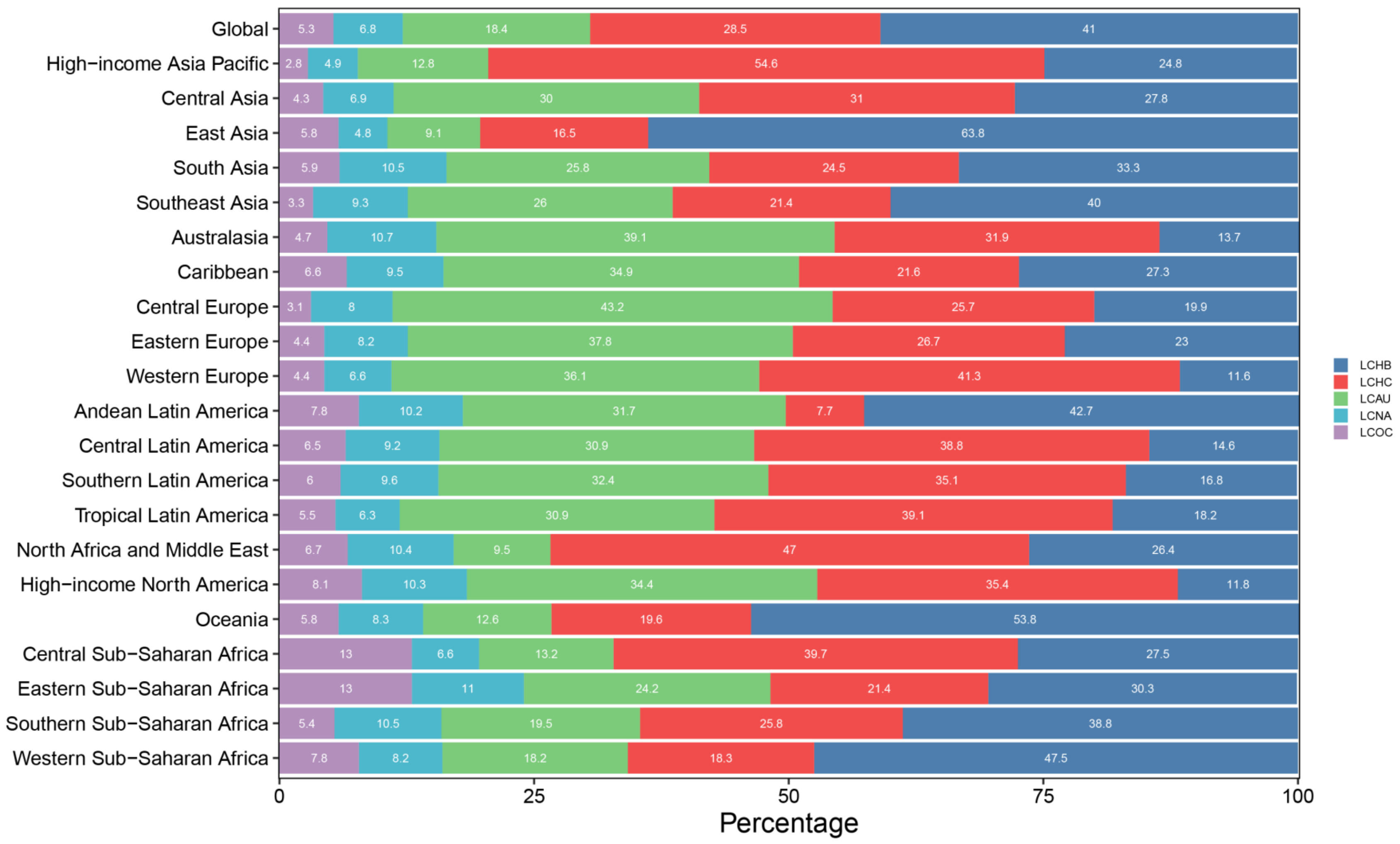

Fig S5: Contribution of LCHB, LCHC, LCAU, LCNA, and LCOC to primary liver cancer incident cases, both sexes, globally and by region, 2019

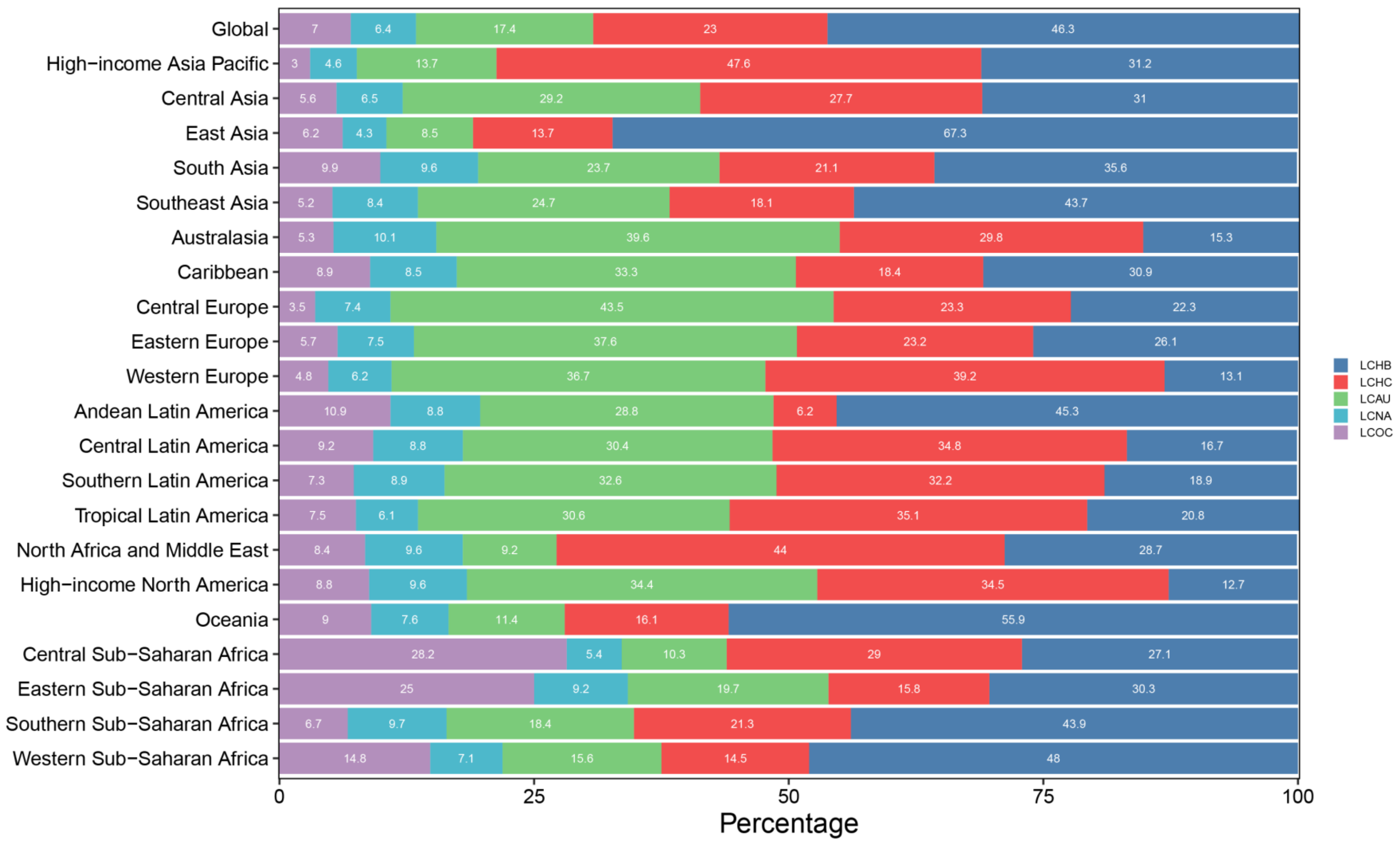

Fig S6: Contribution of LCHB, LCHC, LCAU, LCNA, and LCOC to primary liver cancer DALYs, both sexes, globally and by region, 2019

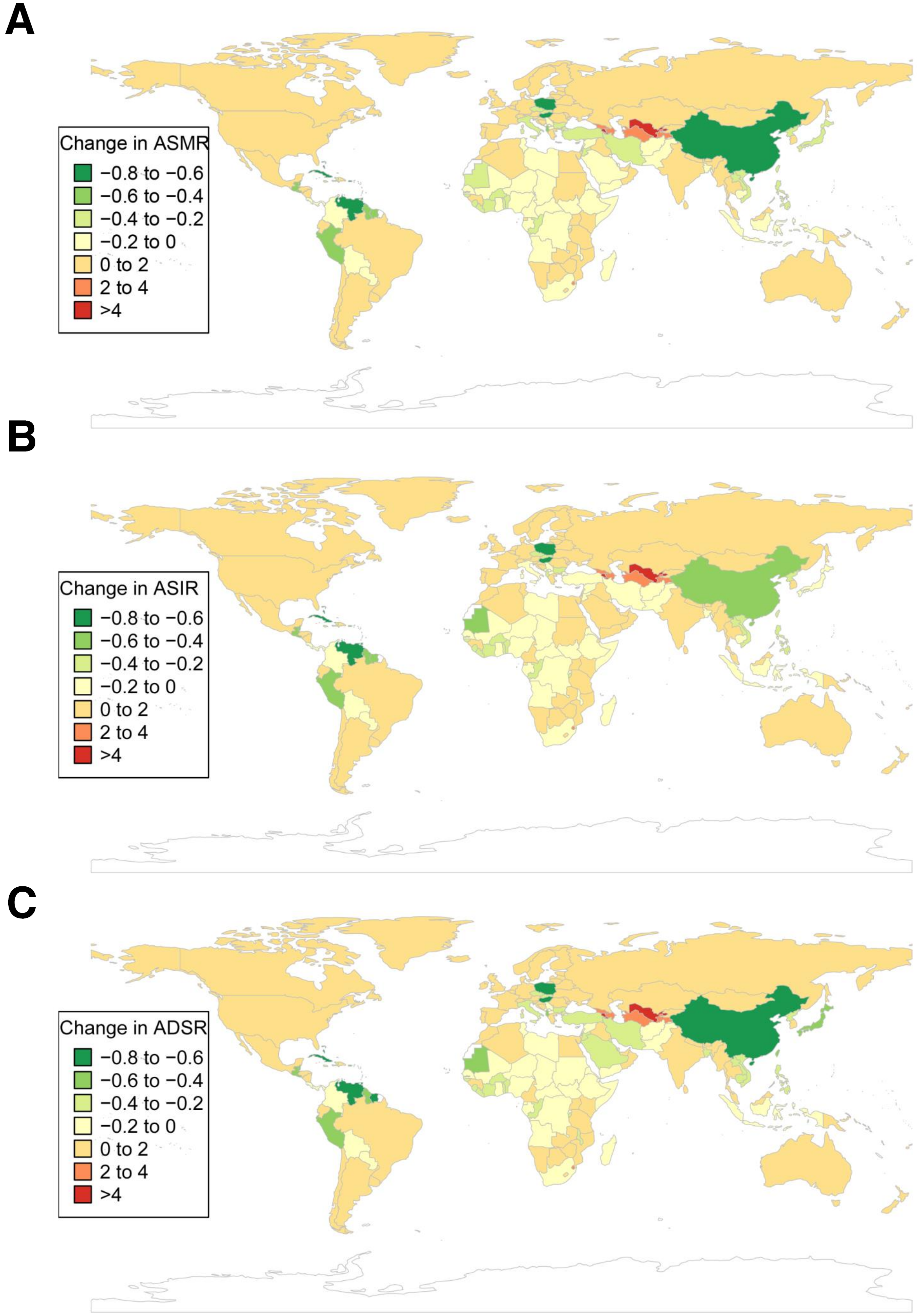

Fig.S7 The estimated percentage change of primary liver cancer from 1990 to 2019: (A) The percentage change in ASMR. (B) The percentage change in ASIR. (C) The percentage change in ASDR.

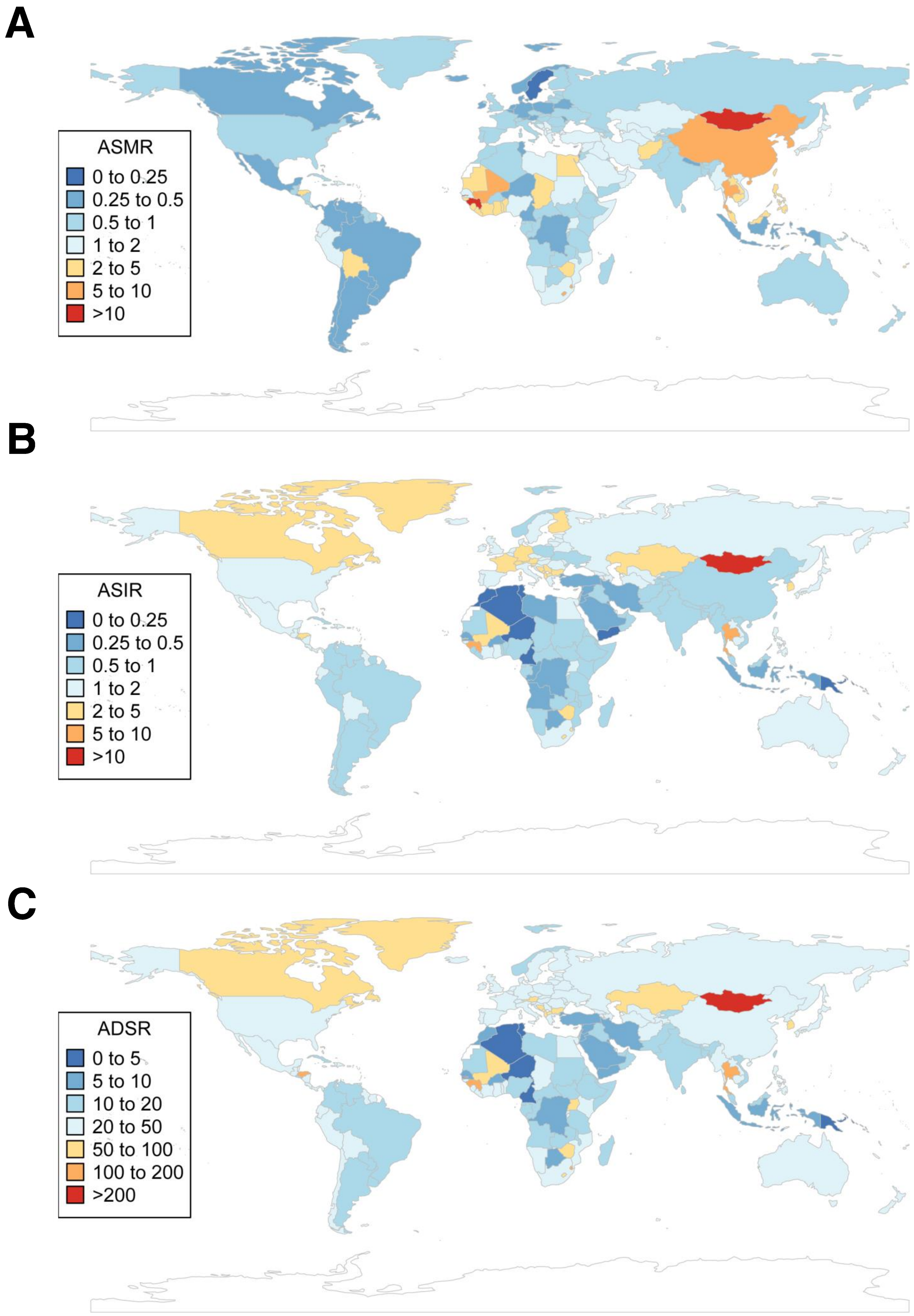

Fig.S8 The global age-standardized rate of LCHB per 100 000 populations in 2019, by country and territory. (A) ASMR in 2019; (B) ASIR in 2019; (C) ASDR in 2019.

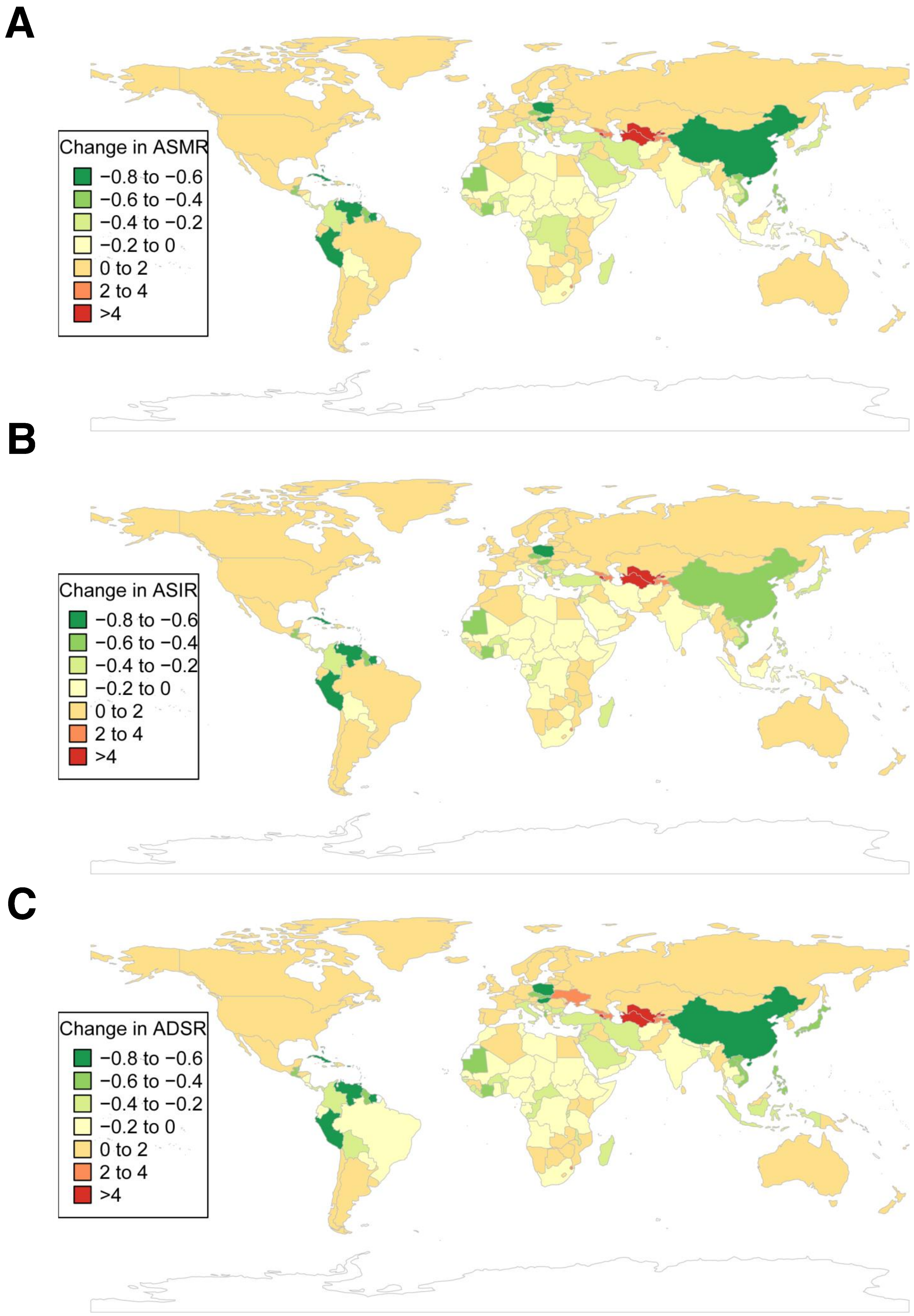

Fig. S9 The estimated percentage change of LCHB from 1990 to 2019: (A) The percentage change in ASMR. (B) The percentage change in ASIR. (C) The percentage change in ASDR.

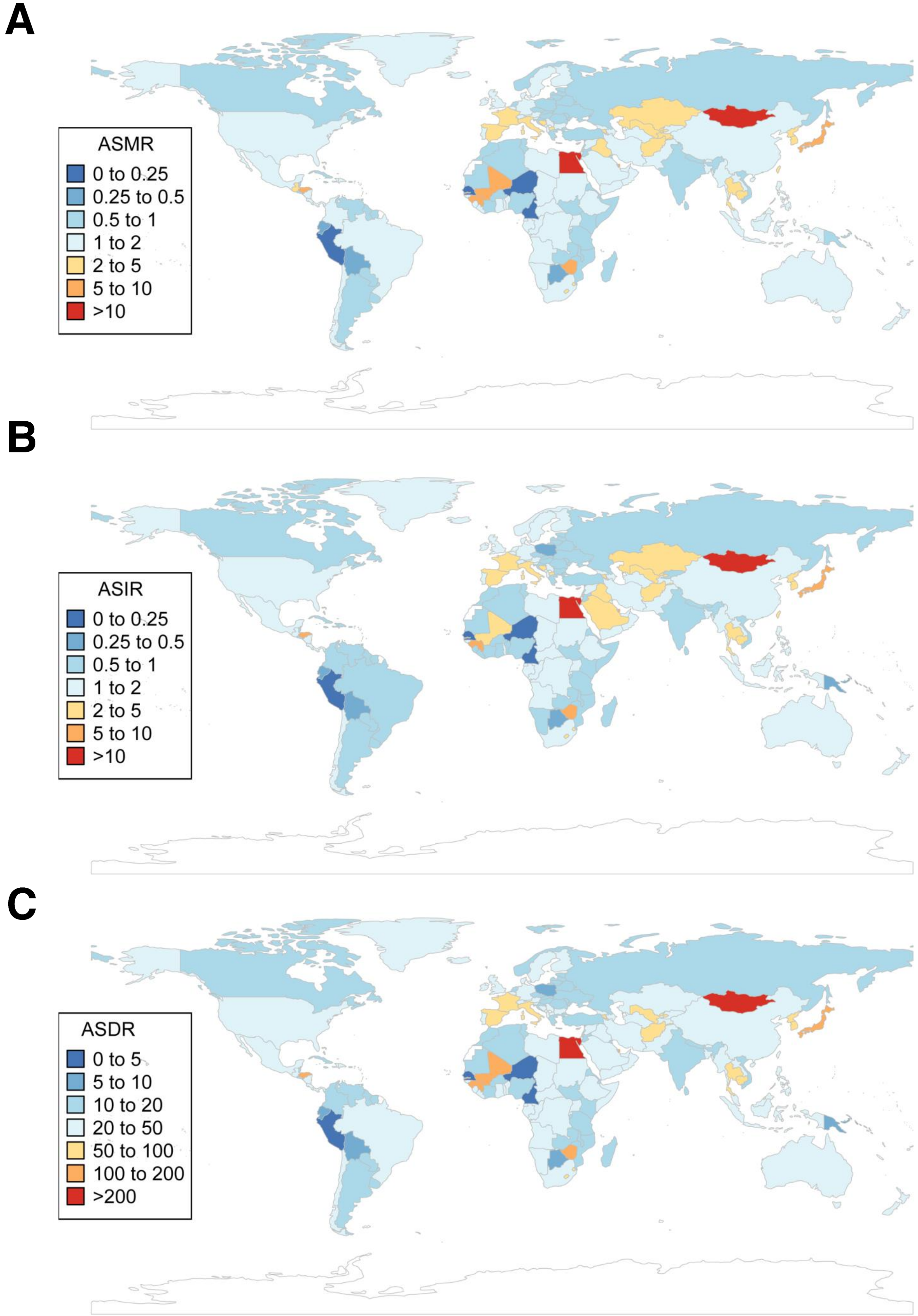

Fig.S10 The global age-standardized rate of LCHC per 100 000 populations in 2019, by country and territory. (A) ASMR in 2019; (B) ASIR in 2019; (C) ASDR in 2019.

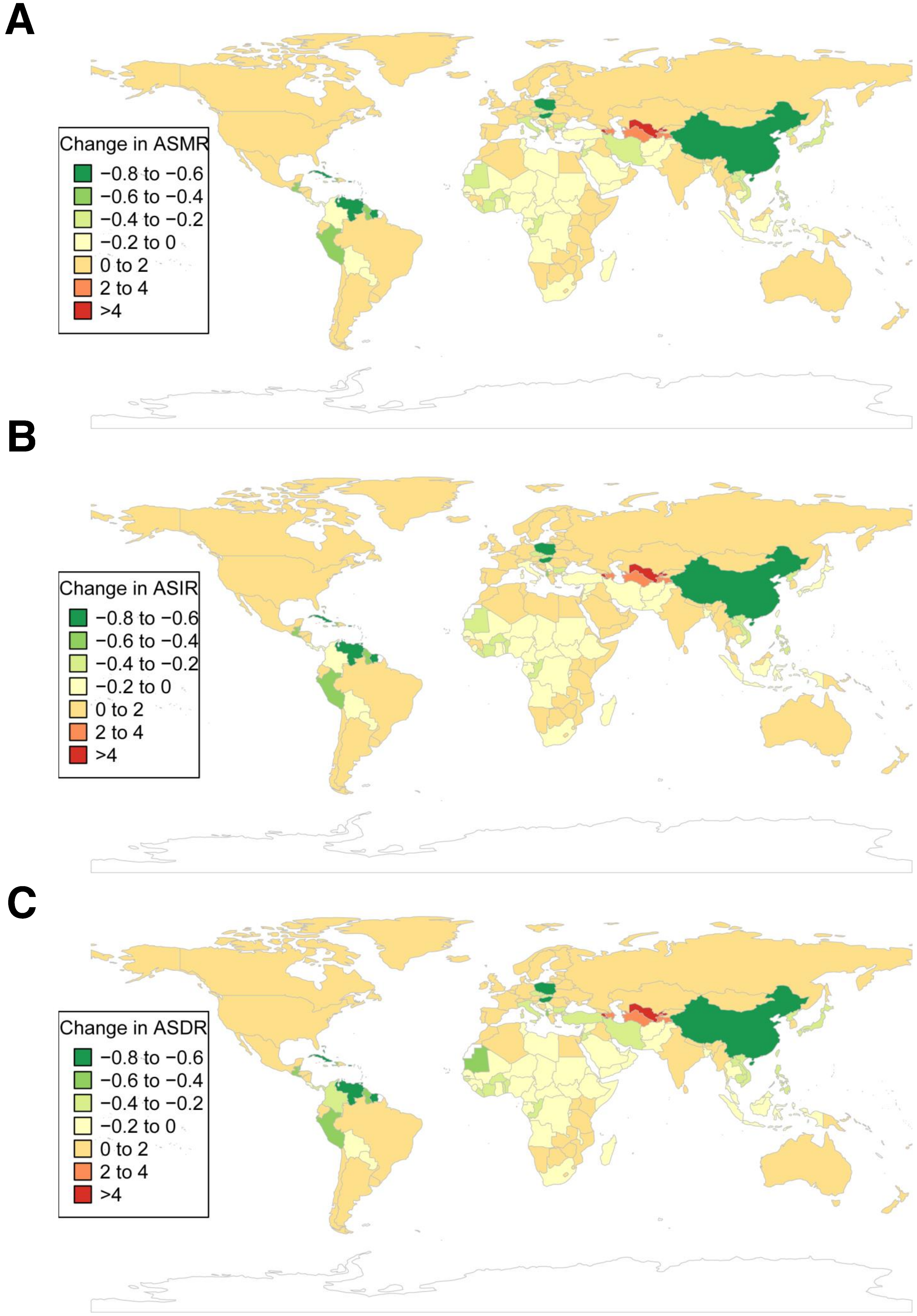

Fig.S11 The estimated percentage change of LCHC from 1990 to 2019: (A) The percentage change in ASMR. (B) The percentage change in ASIR. (C) The percentage change in ASDR.

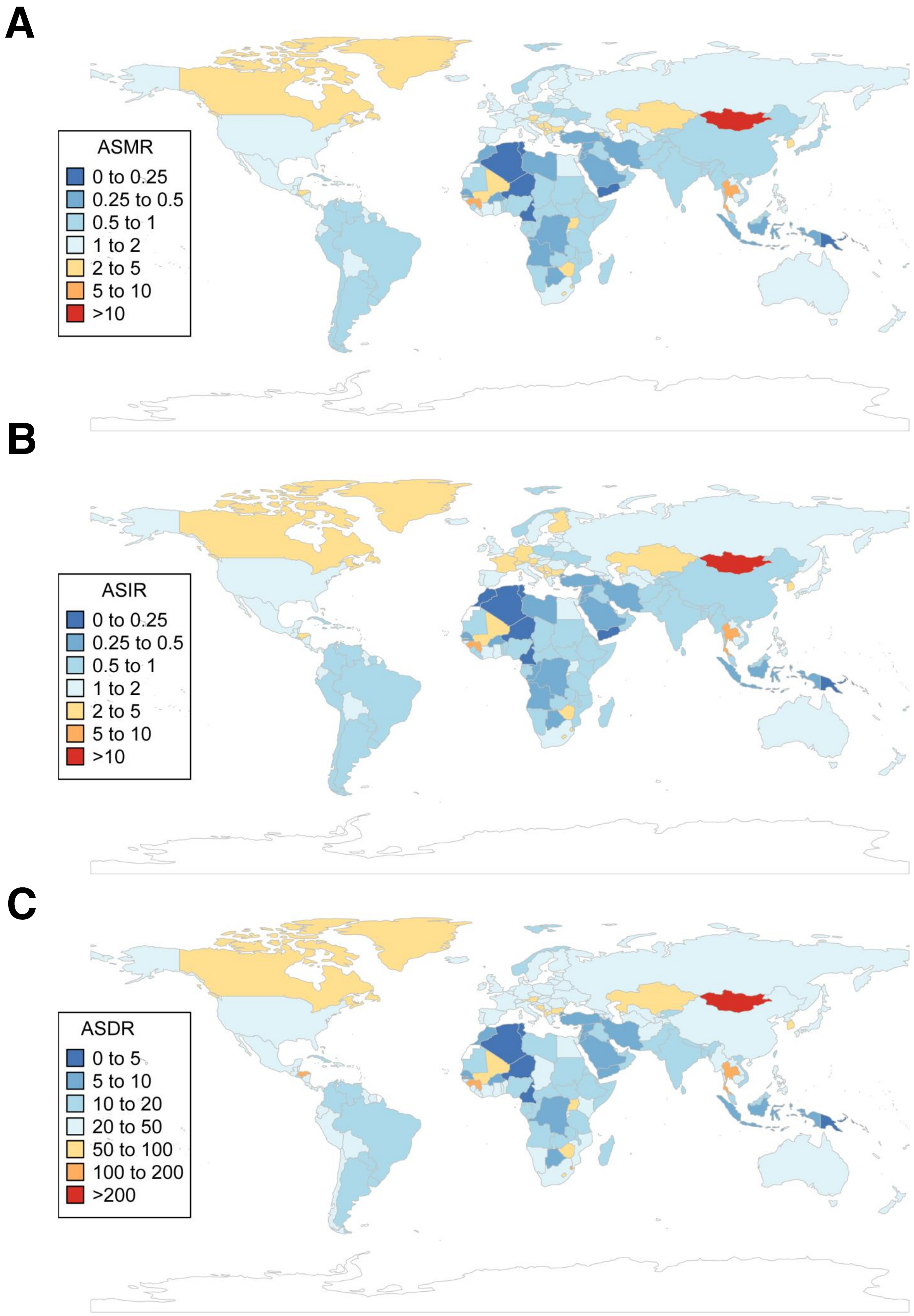

Fig.S12 The global age-standardized rate of LCAU per 100 000 populations in 2019, by country and territory. (A) ASMR in 2019; (B) ASIR in 2019; (C) ASDR in 2019.

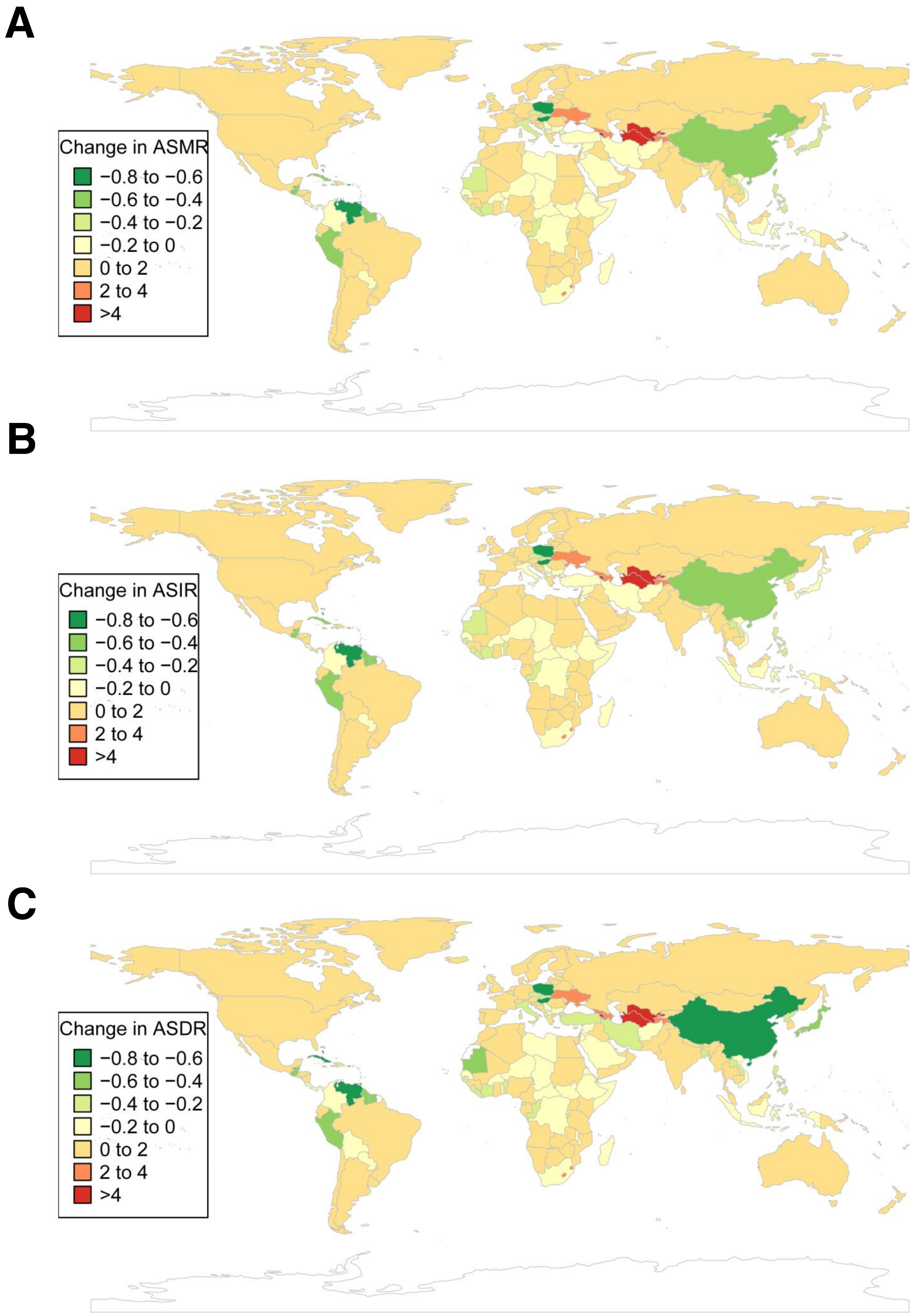

Fig.S13 The estimated percentage change of LCAU from 1990 to 2019: (A) The percentage change in ASMR. (B) The percentage change in ASIR. (C) The percentage change in ASDR.

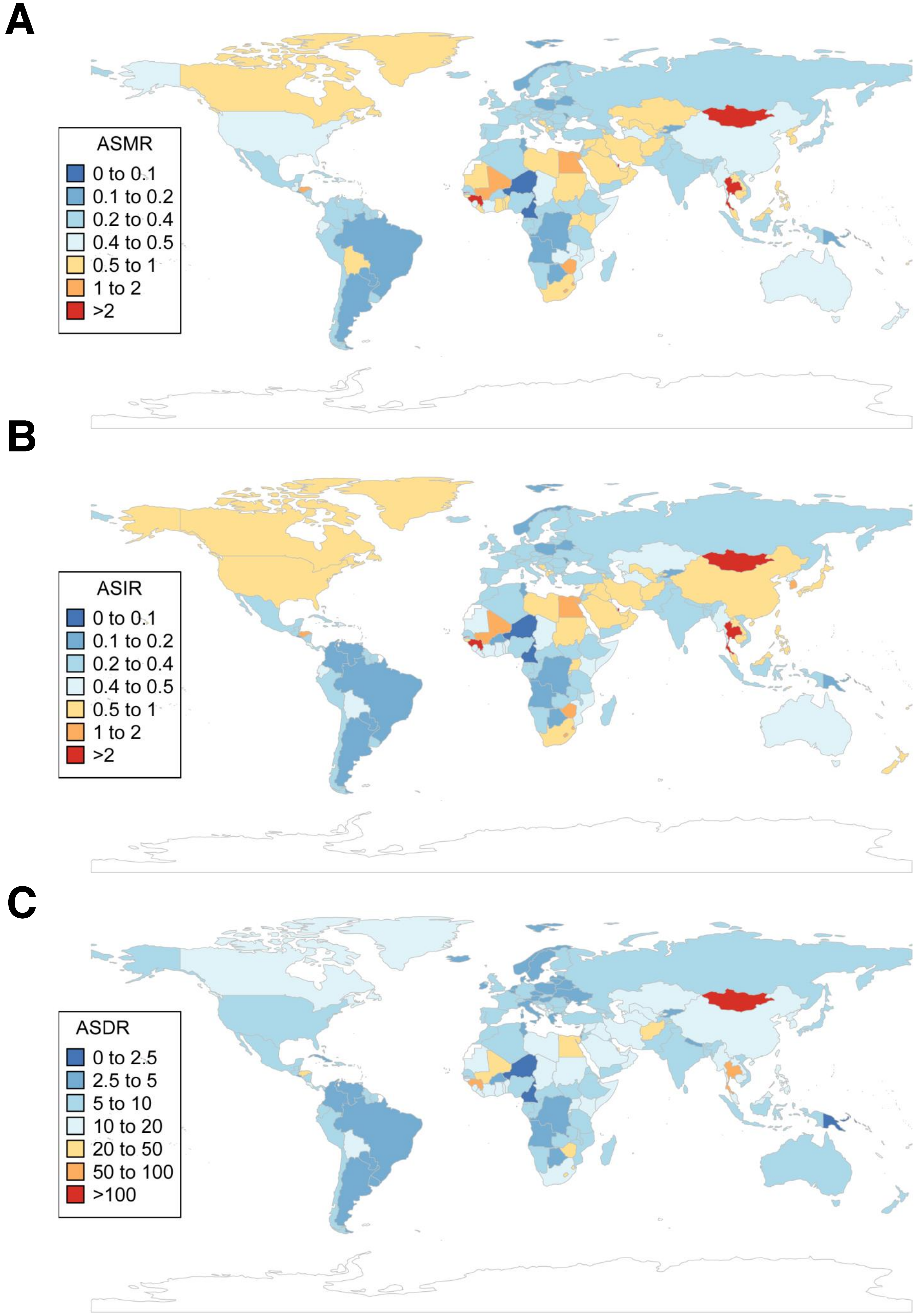

Fig. S14 The global age-standardized rate of LCNA per 100 000 populations in 2019, by country and territory. (A) ASMR in 2019; (B) ASIR in 2019; (C) ASDR in 2019.

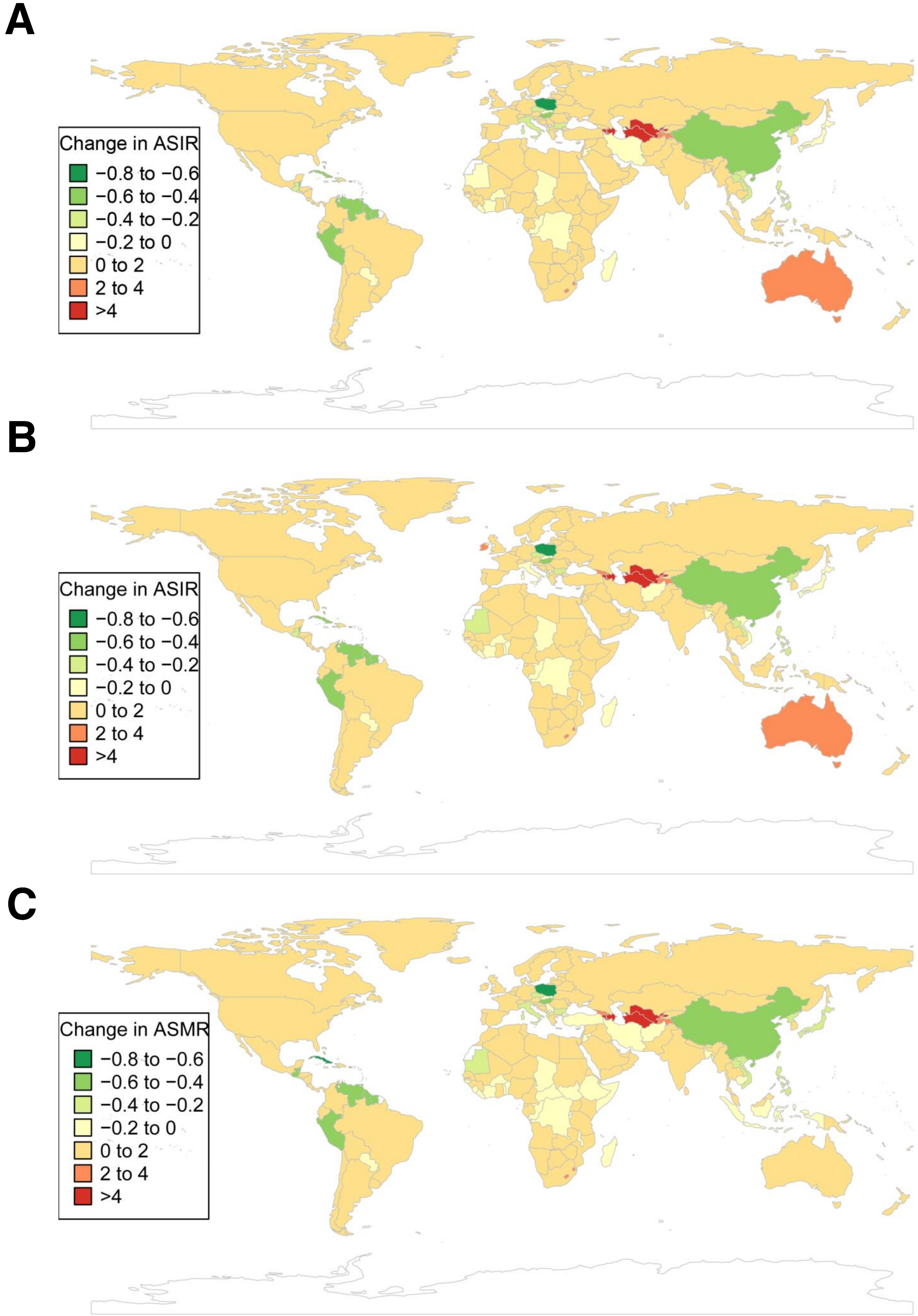

Fig.S15 The estimated percentage change of LCNA from 1990 to 2019: (A) The percentage change in ASMR. (B) The percentage change in ASIR. (C) The percentage change in ASDR.

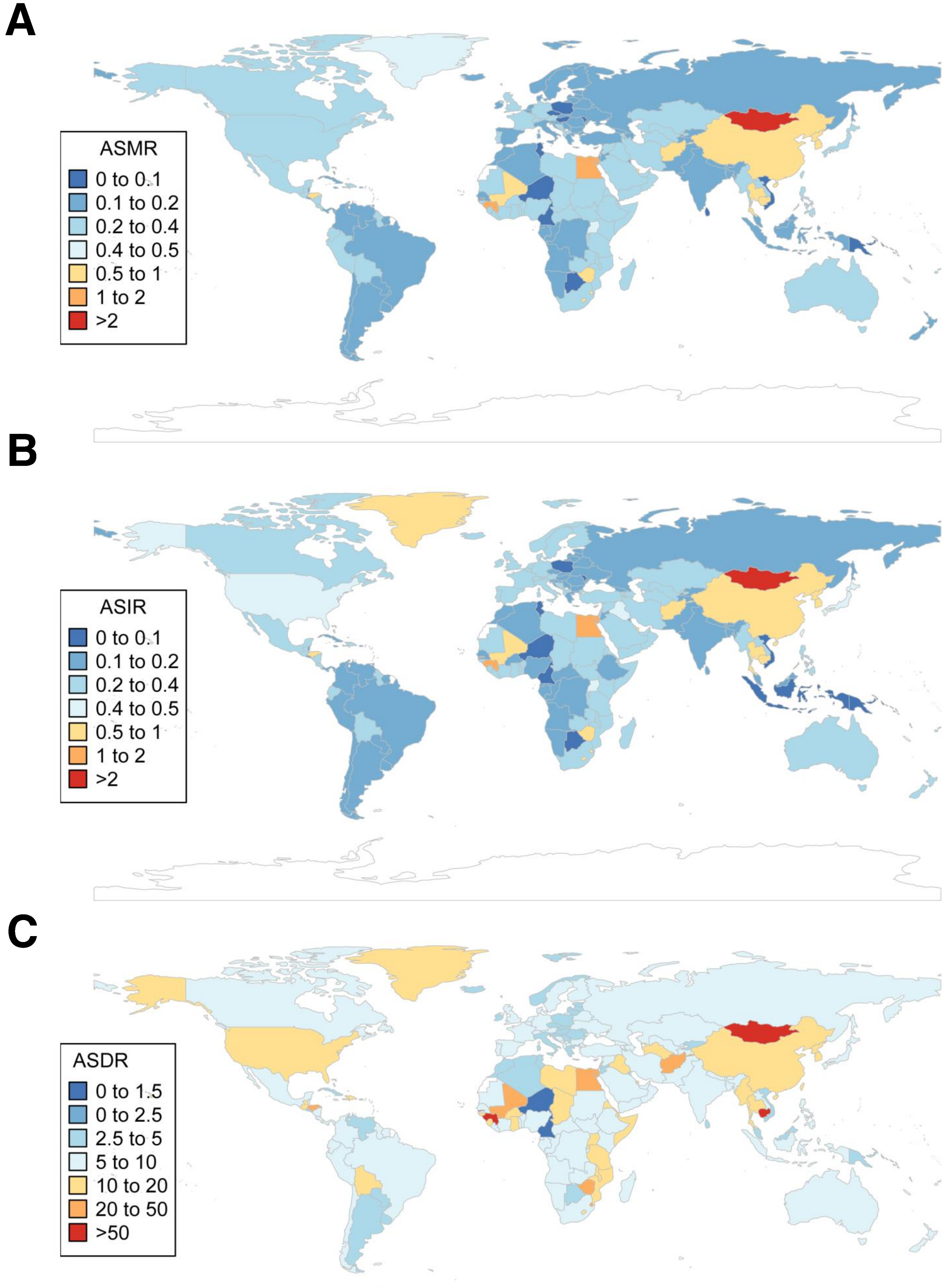

Fig. S16 The global age-standardized rate of LCOC per 100 000 populations in 2019, by country and territory. (A) ASMR in 2019; (B) ASIR in 2019; (C) ASDR in 2019.

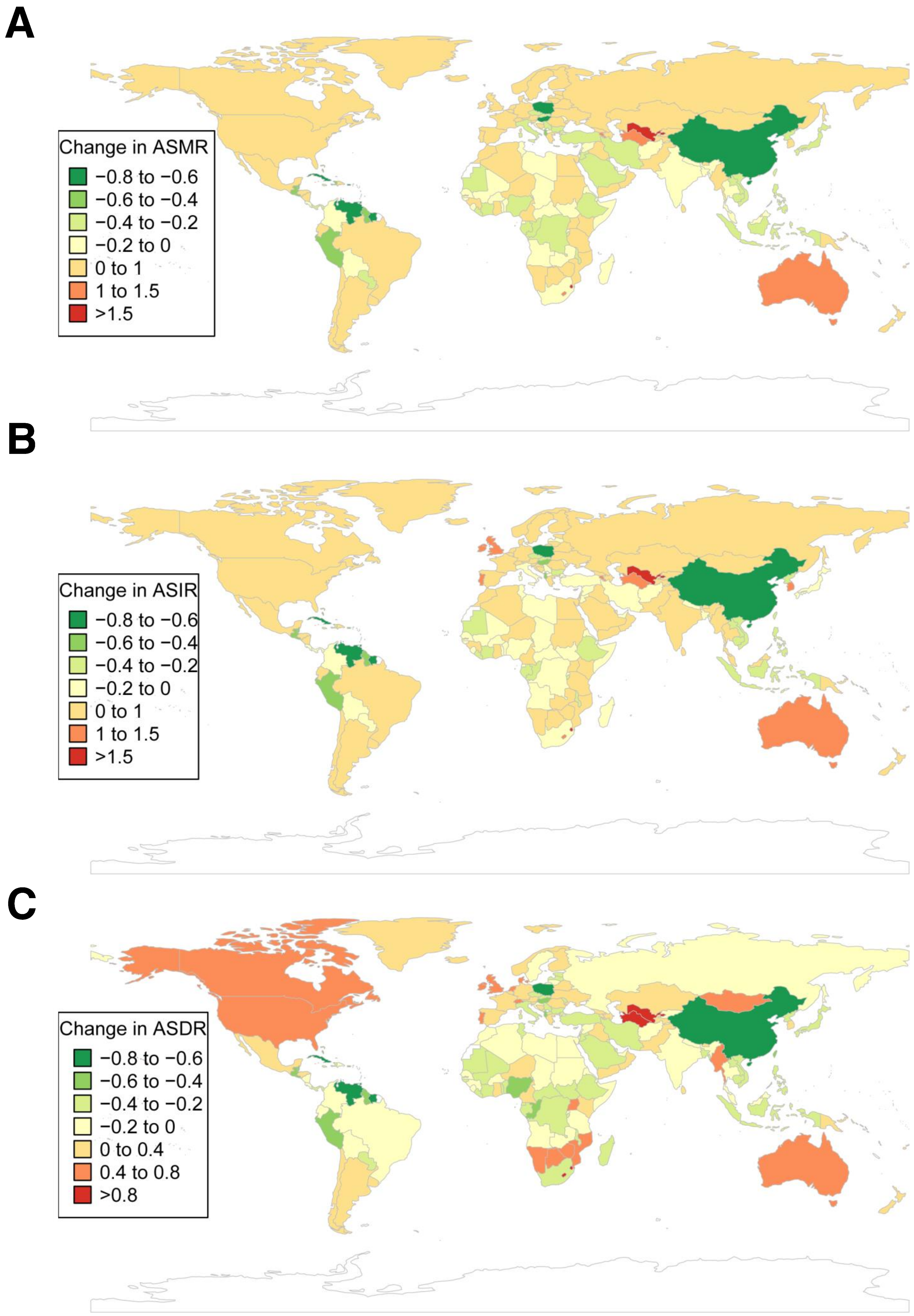

Fig.S17 The estimated percentage change of LCOC from 1990 to 2019: (A) The percentage change in ASMR. (B) The percentage change in ASIR. (C) The percentage change in ASDR.

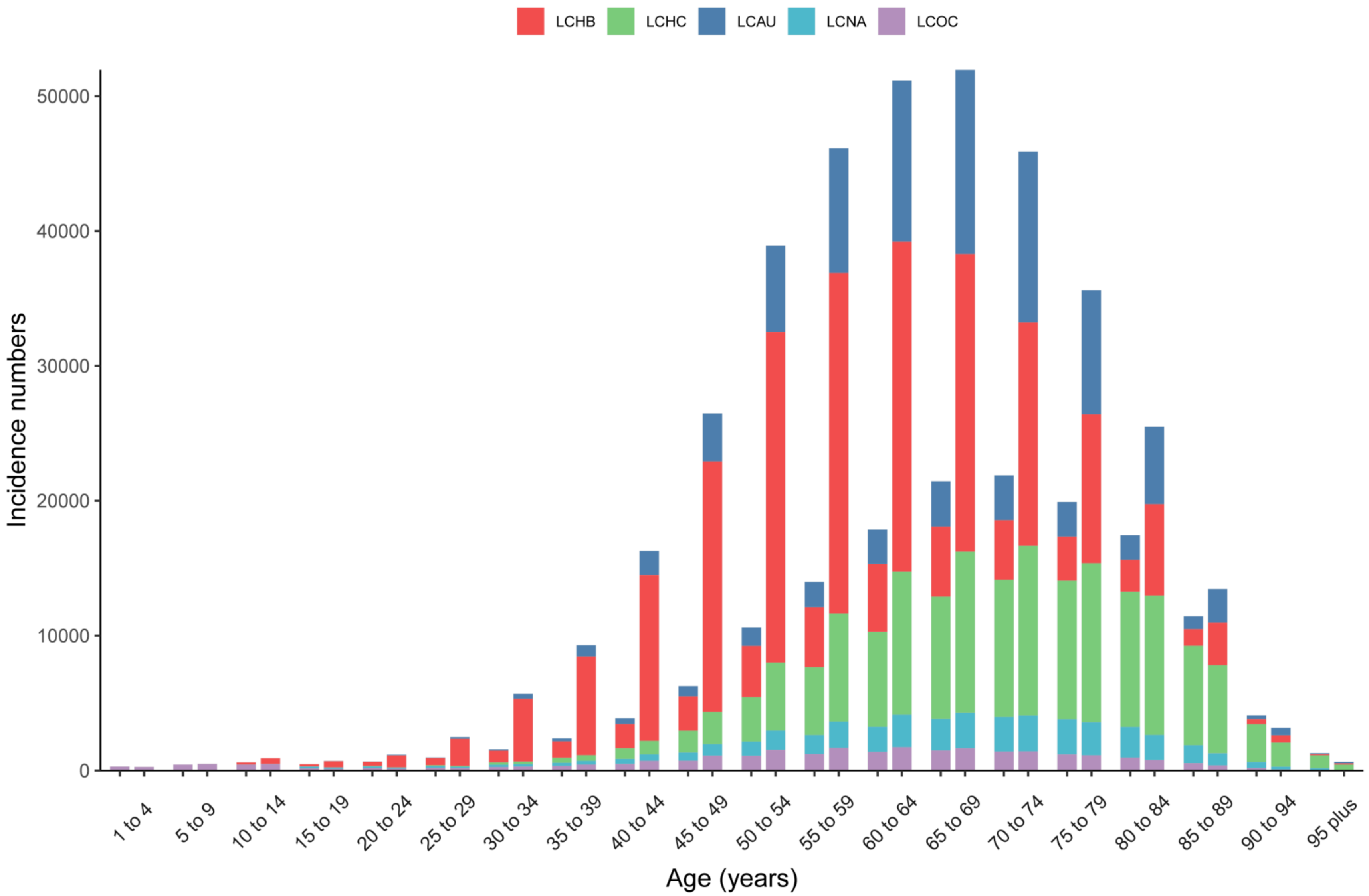

Fig.S18 Global primary liver cancer incident cases by etiology and age for females and males, 2019. For each group, the left column showed case data in female and the right column shows data in male.

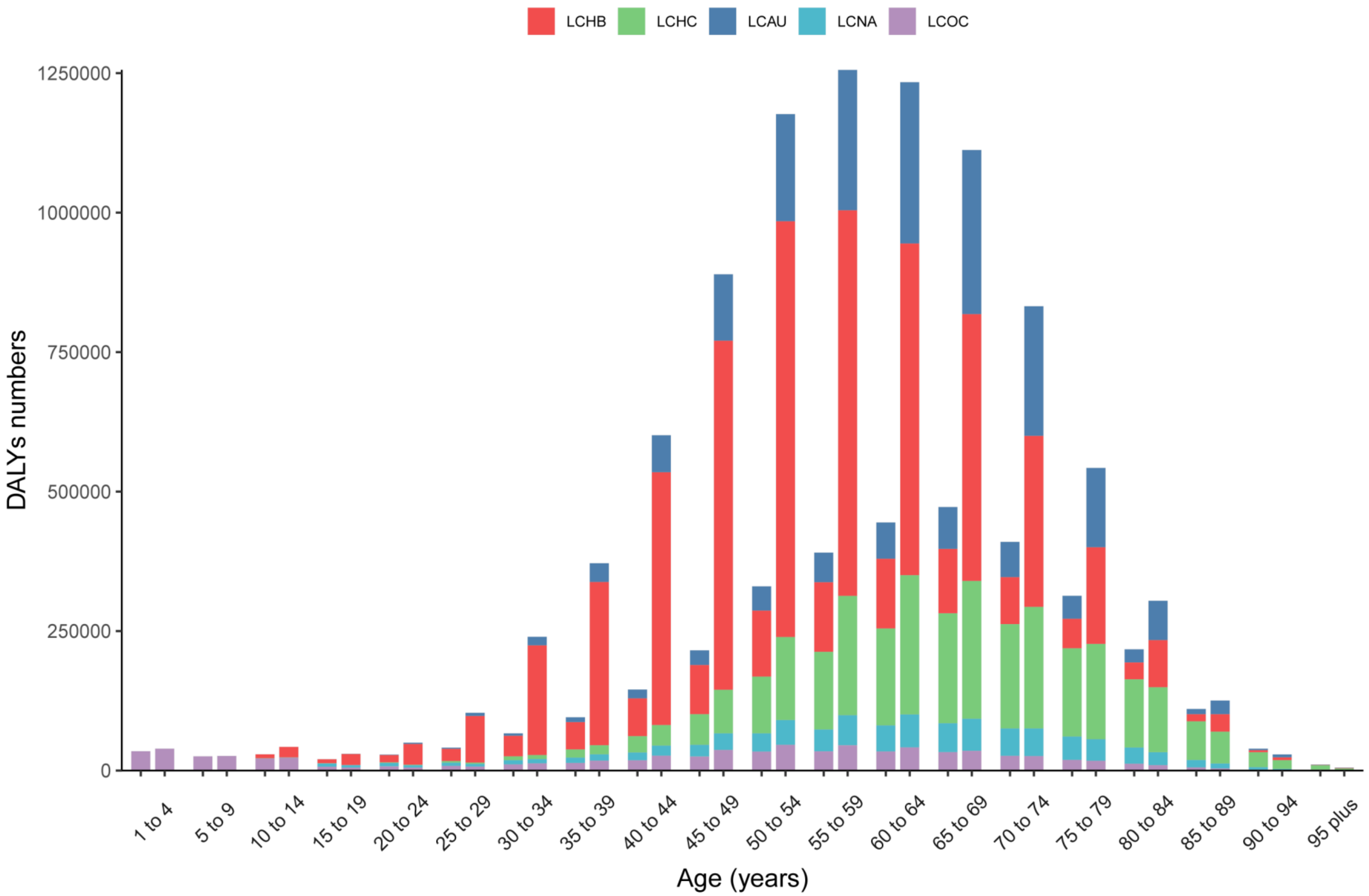

Fig.S19 Global primary liver cancer DALYs by etiology and age for females and males, 2019. For each group, the left column showed case data in female and the right column shows data in male.

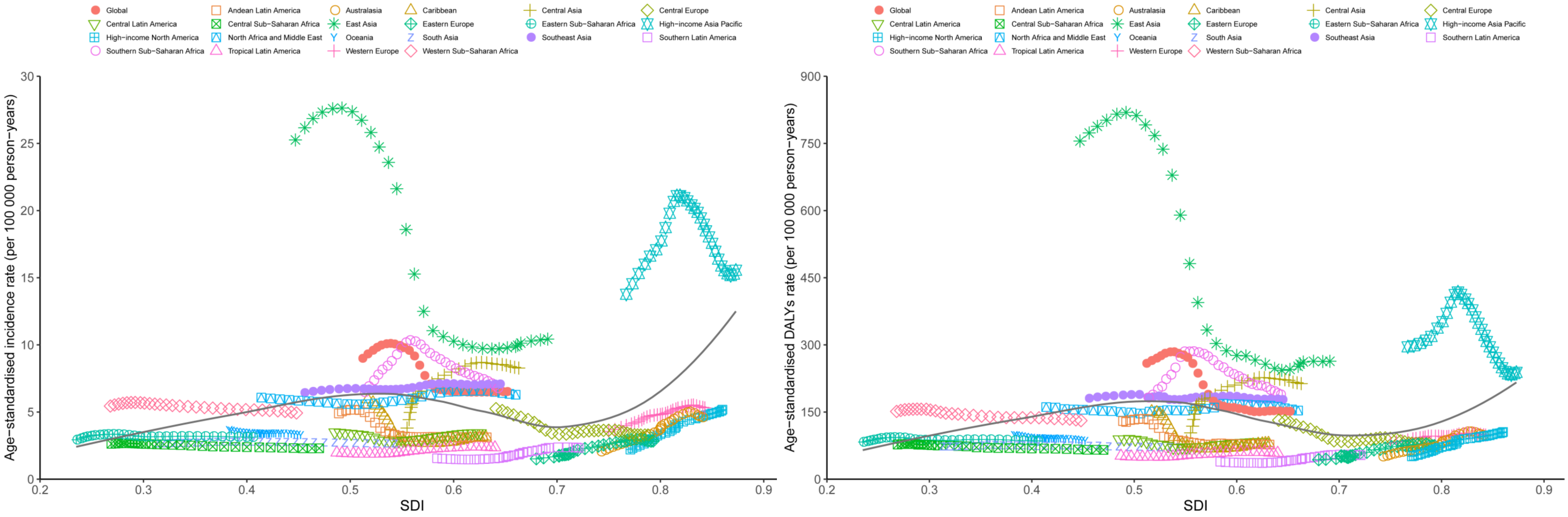

Fig S20: Age-standardised rates of primary liver cancer globally and for 21 regions by SDI, 1990-2019 (A) Age-standardised incidence rate per 100 000 population. (B) Age-standardised DALYs rate per 100 000 population

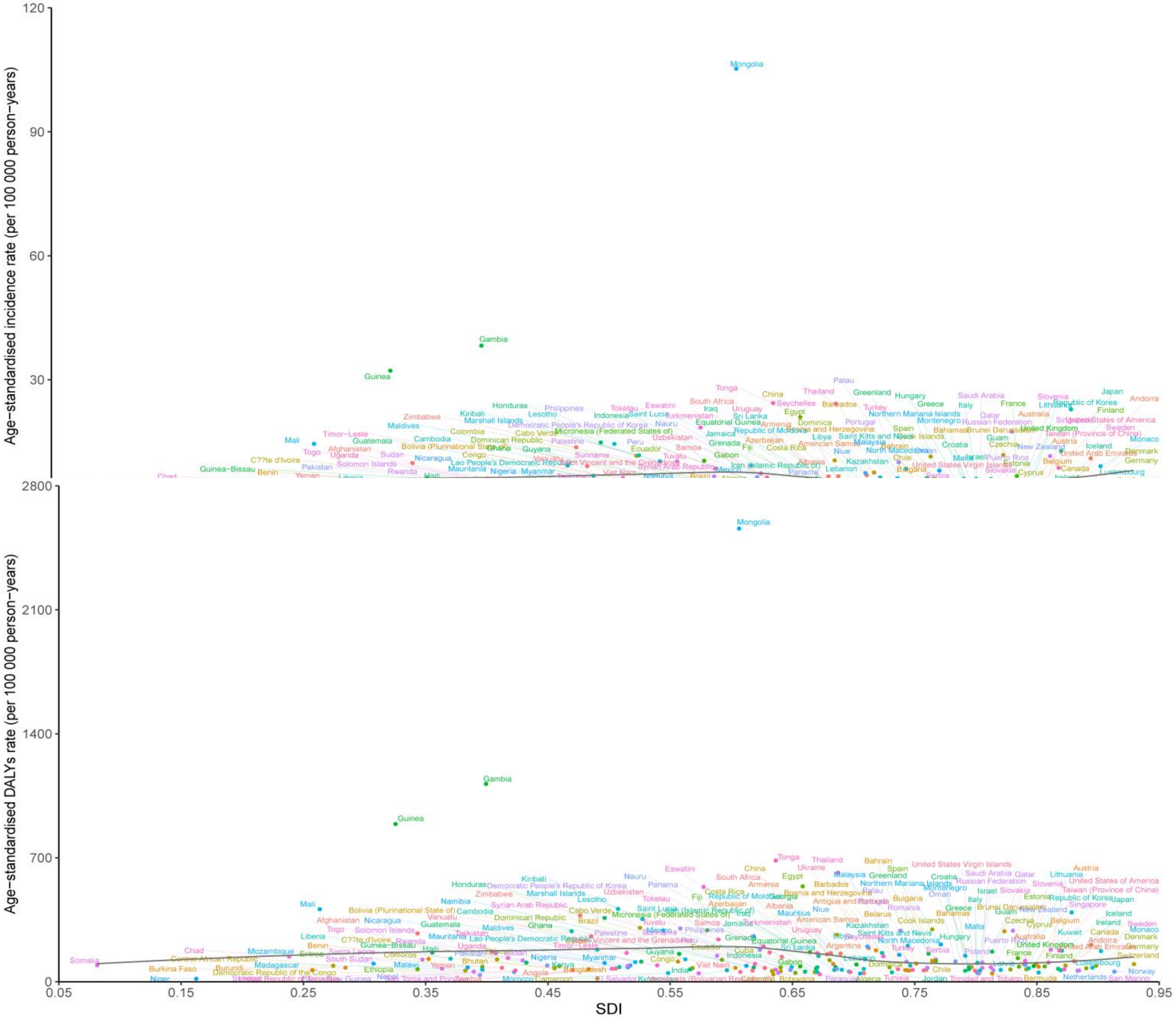

Fig S21: Age-standardised rates per 100 000 population by 204 countries and sociodemographic index(SDI), 2019. (A) Age-standardised incidence rate per 100 000 population. (B) Age-standardised DALYs rate per 100 000 population
